# Selective 1,4-syn-Addition to Cyclic 1,3-Dienes via Hybrid Palladium Catalysis

**DOI:** 10.1021/acscentsci.4c00094

**Published:** 2024-05-15

**Authors:** Yan Liang, Tiancen Bian, Komal Yadav, Qixin Zhou, Liejin Zhou, Rui Sun, Zuxiao Zhang

**Affiliations:** † Key Laboratory of the Ministry of Education for Advanced Catalysis Materials, College of Chemistry and Materials Science, Zhejiang Normal University, Jinhua 321017, China; ‡ Department of Chemistry, University of Hawai’i at Ma̅noa, Honolulu, Hawaii 96822, United States

## Abstract

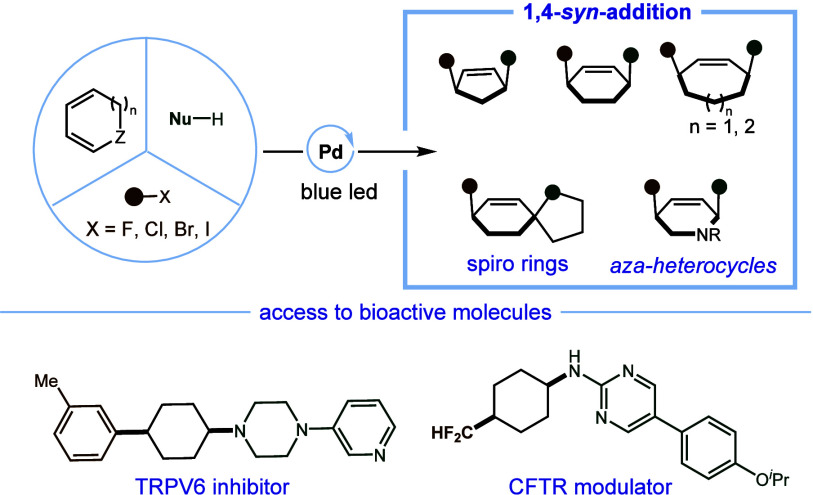

1,4-cis-Disubstituted cyclic compounds play a pivotal role in pharmaceutical development, offering enhanced potency and bioavailability. However, their stereoselective and modular synthesis remains a long-standing challenge. Here, we report an innovative strategy for accessing these structures via mild conditions employing cyclic 1,3-dienes/alkyl(aryl)halides and amines. This procedure exhibits a wide substrate scope that tolerates various functional groups. The utility of this method is demonstrated in the efficient synthesis of a TRPV6 inhibitor, CFTR modulator, and other bioactive molecules. Combined experimental and computational studies suggest that the hybrid palladium-catalyzed radical-polar crossover mechanism is crucial for achieving exceptional 1,4-syn-addition selectivity (dr > 20:1).

## Abstract 2

Via hybrid palladium catalysis, we pioneered a redox neutral 1,4-syn-addition to 1,3-cyclic dienes for constructing diverse cyclic compounds, unlocking quick access to bioactive molecules.

## Introduction

**Figure 1:**
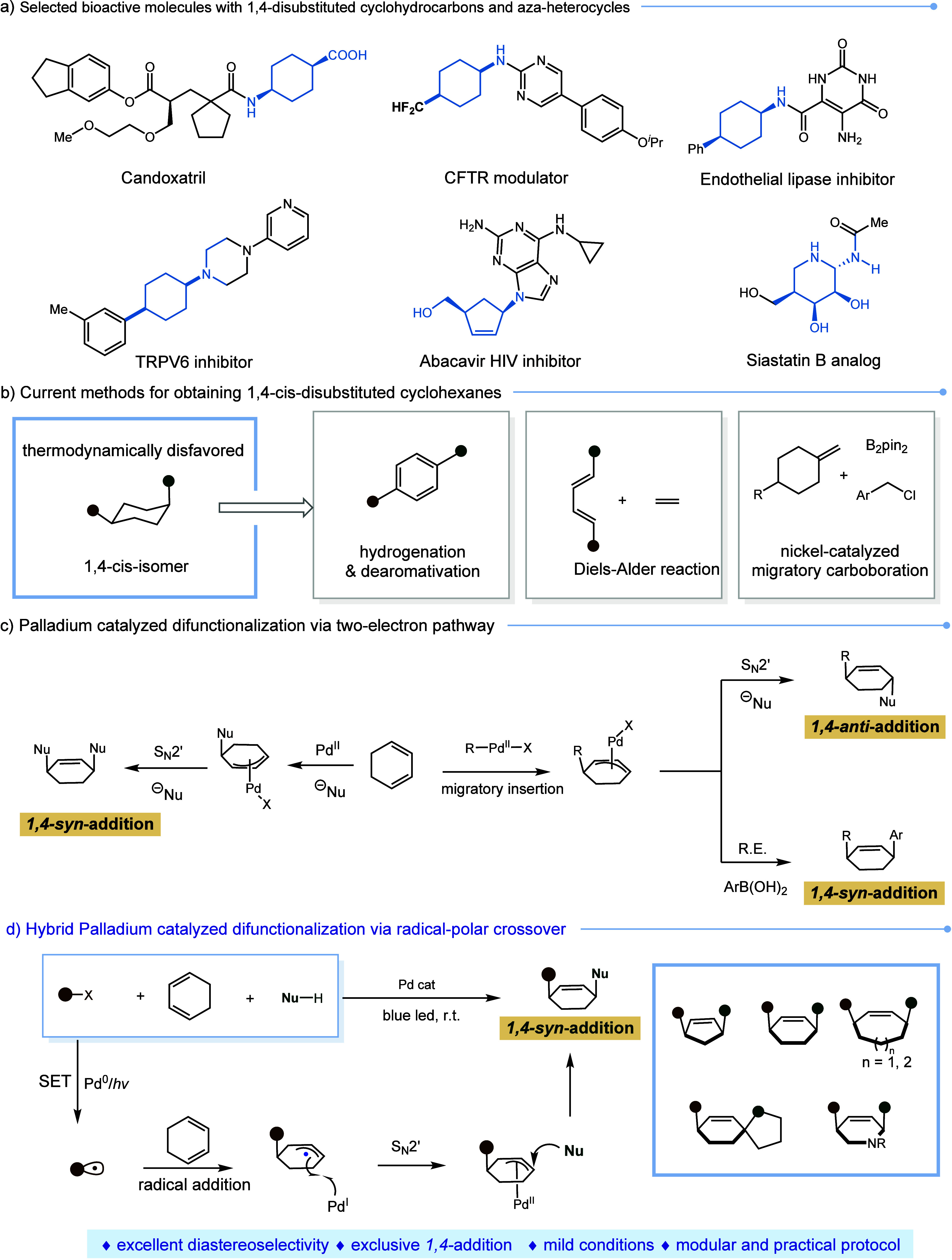
Application and synthesis of 1,4-cis-disubstituted cyclic frameworks.

Contemporary drug discovery endeavors have increasingly focused on saturated compounds due to their intricate three-dimensional geometries, which often impart superior bioactivities and physical properties compared to their planar bioisosteres.^1^ Given that a substantial majority of small-molecule pharmaceuticals feature at least one ring system, the development of efficient synthetic methodologies for stereospecific construction of saturated rings has garnered significant attention.^2^ The 1,4-cis-disubstituted cyclic framework represents a pivotal structural motif within a wide spectrum of pharmaceutical molecules, including notable examples such as candoxatril,^3^ a CFTR modulator,^4^ an endothelial lipase inhibitor,^5^ TRPV6 inhibitor,^6^ abacavir,^7^ and a siastatin B analog^8^ (Figure 1a). Considerable effort has been devoted to the selective construction of cyclic structures with energetically unfavorable 1,4-cis substitutions. However, the available methods are still limited to selective hydrogenation^9^ and dearomatization of arenes^10^ and the Diels–Alder reaction.^11^

Multicomponent reactions, facilitating the rapid assembly of multifunctional molecules with structural diversity from readily accessible starting materials, stand out as highly efficient and practical synthetic strategies, characterized by their atom- and step-economical nature.^12^ Recently, the Yin research group introduced an elegant approach for accessing thermodynamically disfavored substituted cyclohexanes through nickel-catalyzed migration functionalization of alkenes with a preinstalled substitution.^13^ On the other hand, the transition metal-catalyzed difunctionalization of conjugated dienes has offered a dependable platform for the preparation of polysubstituted alkenes in a stereoselective manner.^14^ As an illustration, the Bäckvall group has elegantly devised a palladium-catalyzed 1,4-syn-difunctionalization of 1,3-dienes under oxidative conditions. The remarkable diastereoselectivity observed in this process is attributed to the dual nucleophilic anti-attack mechanism (Figure 1c).^15^ Additionally, the Larock group developed a three-component coupling of aryl halides, 1,3-cyclohexadiene, and boronic acids to provide 1,4-syn-addition products (Figure 1c).^16^ However, these methods still suffered from limited substrate scope. While using a nonstabilized carbon nucleophile, the redox neutral transformation usually favors 1,4-trans isomer products or mixtures (Figure 1c), as they typically involve syn-migratory insertion and S_N_2′ substitution, particularly with amines as nucleophiles.^17^ Given the prevalence of 1,4-cis-difunctionalized cyclic scaffolds and the existing limitations in current synthetic approaches, a new strategy for the modular synthesis of these thermodynamically disfavored isomers in a highly stereoselective and efficient manner is highly desirable. Such an approach would significantly enrich the toolkit of organic synthetic chemists and expand the compound library available for drug discovery purposes.

The major challenge in achieving a general redox-neutral 1,4-syn-addition to cyclic 1,3-dienes involves reversing the conventional syn-migratory insertion of the R-Pd(II)-X complex, as the S_N_2′ preferred anti-attack mode in the presence of amines complicates matters. Recent advancements in hybrid palladium catalysis have demonstrated notable reactivity to reduce carbon–halogen (C–X) bonds and generate carbon-centered radicals. The formed Pd(I) species exhibit a pronounced affinity for engaging with subsequent carbon radicals, resulting in the formation of Pd(II) intermediates amenable to classic palladium chemistry.^18^ Thus, we envisioned that the hybrid palladium catalysis would enable a formal stepwise anti-migratory insertion of cyclic 1,3-dienes since the steric effects favor the capture of the allylic carbon radical from the less hindered backside by Pd(I). Subsequently, the resulting allylic Pd(II) complex undergoes S_N_2′ nucleophilic substitution in the presence of amines, yielding 1,4-cis-carboamination products (see Figure 1d). Herein, we present a reliable and modular protocol for synthesizing 1,4-cis-substituted cyclic compounds through excited-palladium-catalyzed multicomponent reactions. This method could efficiently assemble a diverse array of amines, electrophiles, and cyclo-1,3-dienes into 1,4-syn-addition products with excellent regio- and diastereoselectivity.

## Results and Discussion

We initiated this investigation by selecting trifluoromethylated arene **S1**, cyclohexyl 1,3-diene **S2**, and morpholine **S3** as the model substrates (Table 1). Employing Pd(PPh_3_)_4_ as a catalyst at a 5 mol % loading in DMSO as the solvent led to a smooth reaction, yielding the desired product **1** in excellent diastereoselectivity and good yield (88%, Table 1). The introduction of external ligands (**L1-L4**) proved to be ineffective and resulted in decreased yields. A solvent screening experiment revealed that only dimethylformamide (DMF) provided comparable yields, while other solvents led to diminished yields (Table 1, entries 2–6). Substituting Pd(PPh_3_)_4_ with other catalysts resulted in either a halted reaction or a significant decrease in yield (Table 1, entries 7–9). Notably, reducing the catalyst loading to 2.5 mol % still led to the desired product in high yield, at 86% (Table 1, entry 10). Conversely, increasing the catalyst loading to 10 mol % led to a slightly lower yield (Table 1, entry 11). Various bases were examined, with K_2_HPO_4_ identified as the optimal base for this transformation (Table 1, entries 12–16). Control experiments confirmed that both the presence of the catalyst and exposure to light were essential for the success of this reaction (Table 1, entries 17, 18).

**Table 1:**
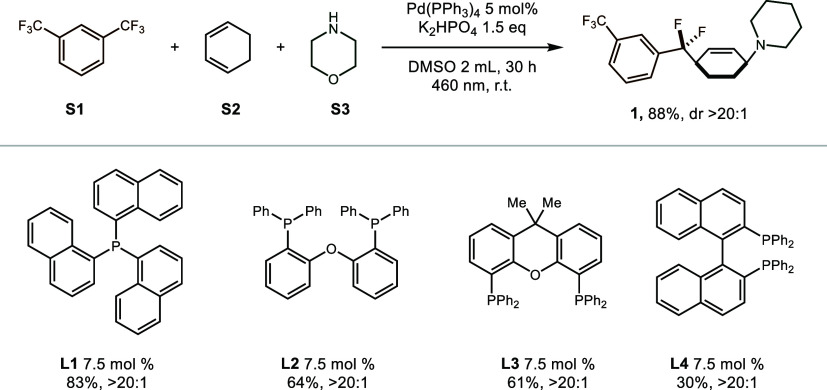
Optimization of Conditions^a^

entry	catalyst	base	solvent	yield, *dr*^b^
1	Pd(PPh_3_)_4_ 5 mol %	K_2_HPO_4_	DMSO	88%, >20:1
2	Pd(PPh_3_)_4_ 5 mol %	K_2_HPO_4_	DMF	88%, >20:1
3	Pd(PPh_3_)_4_ 5 mol %	K_2_HPO_4_	benzene	trace
4	Pd(PPh_3_)_4_ 5 mol %	K_2_HPO_4_	1,4-dioxane	trace
5	Pd(PPh_3_)_4_ 5 mol %	K_2_HPO_4_	EA	25%, >20:1
6	Pd(PPh_3_)_4_ 5 mol %	K_2_HPO_4_	DCE	trace
7	Pd(OAc)_2_ 5 mol %	K_2_HPO_4_	DMF	0
8	Pd(acac)_2_ 5 mol %	K_2_HPO_4_	DMF	0
9	Pd(PPh_3_)_2_Cl_2_ 5 mol %	K_2_HPO_4_	DMF	13%, >20:1
10	Pd(PPh_3_)_4_ 2.5 mol %	K_2_HPO_4_	DMF	86%, >20:1
11	Pd(PPh_3_)_4_ 7.5 mol %	K_2_HPO_4_	DMF	71%, >20:1
12	Pd(PPh_3_)_4_ 5 mol %	NaOAc	DMF	58%, >20:1
13^c^	Pd(PPh_3_)_4_ 5 mol %	Na_2_HPO_4_	DMF	40%, >20:1
14	Pd(PPh_3_)_4_ 5 mol %	Cs_2_CO_3_	DMF	0
15	Pd(PPh_3_)_4_ 5 mol %	Na_2_CO_3_	DMF	48%, >20:1
16	Pd(PPh_3_)_4_ 5 mol %	KHCO_3_	DMF	49%, >20:1
17	no catalyst	K_2_HPO_4_	DMF	NR
18^c^	Pd(PPh_3_)_4_ 5 mol %	K_2_HPO_4_	DMF	NR

a General conditions (unless otherwise indicated): 0.15 mmol of **S1**, 0.15 mmol of **S2**, 0.1 mmol of **S3**, 5 mol % Pd (PPh_3_)_4_, 0.15 mmol of base, solvent (2 mL), 460 nm (10 W), rt, 30 h.

b Yield determined by ^1^H NMR and *dr* ratio determined by ^19^F NMR using 2,2,2-trifluoro-*N*,*N*-dimethylacetamide as an external standard.

c Without light.

**Scheme 1:**
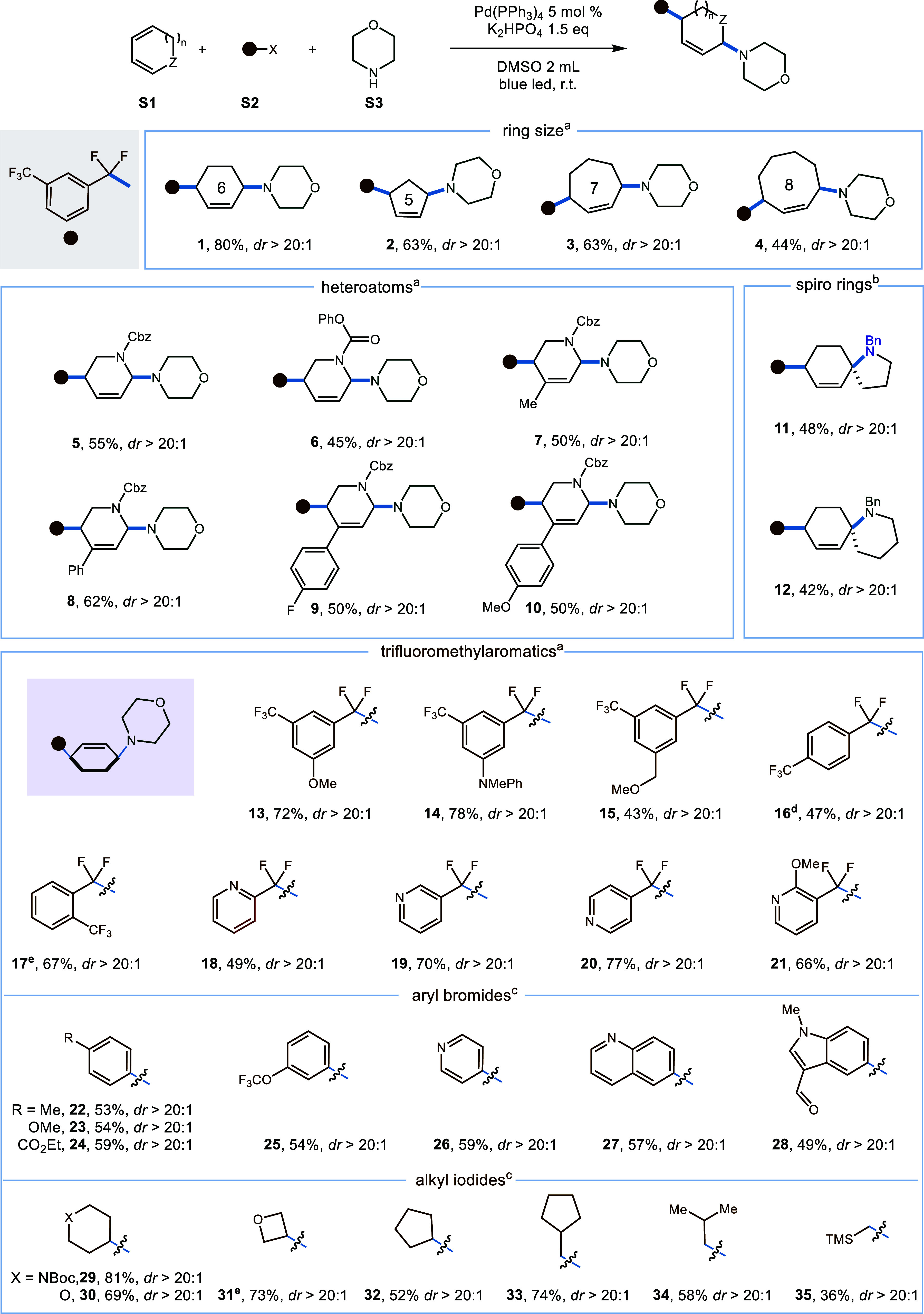
Scope of 1,3-Dienes and Electrophiles 0.15 mmol of **S1**, 0.15 mmol of **S2**, 0.1 mmol of **S3**, 5 mol % Pd(PPh_3_)_4_, 0.15 mmol of K_2_HP0_4_, DMSO (2 mL), 460 nm (10 W), 25 °C, 30 h. 0.15 mmol of **S1**, 0.1 mmol of amine-tethered diene, 5 mol % Pd(PPh_3_)_4_, 0.15 mmol of K_2_HPO_4_, DMSO (2 mL), 460 nm (10 W), 25 °C, 30 h. 0.45 mmol of Ar-Br or alkyl-l, 0.15 mmol of **S1**, 0.1 mmol of **S3**, 10 mol % Pd(PPh_3_)_4_, 0.15 mmol of K_3_P0_4_, DMSO (2 mL), 460 nm (10 W), 25 °C, 20 h. 1-(Toluene-4-sulfonyl)piperazine as nucleophile. Thiomorpholine as nucleophile.

Subsequently, we turned our attention to exploring the versatility of this hybrid palladium-catalyzed reaction, initially assessing a range of cyclic 1,3-dienes with varying ring sizes (Scheme 1). Gratifyingly, cyclic dienes featuring five- to eight-membered rings consistently delivered the desired products **1**–**4** with moderate to high yields and exceptional regio- and diastereoselectivity. Recognizing the significance of aza-heterocycles in medicinal chemistry, a diverse array of dihydropyridines was subjected to the established conditions, resulting in the formation of 1,4-cis-difunctionalized products **5**–**10** in moderate yields. Furthermore, conjugated cyclohexyldienes, equipped with preinstalled nucleophiles, demonstrated compatibility with the optimized reaction conditions, yielding diverse spirocyclic products **11** and **12** of varying sizes. Subsequently, an exploration of the scope of the electrophiles was undertaken. Given the significance of *gem*-difluoromethylene unit in pharmaceutical discovery, our primary focus gravitated toward trifluoromethylaromatics. Various substituted trifluoromethylarenes including meta, para, and ortho ditrifluoromethylated arenes emerged as amenable substrates, delivering the desired products **13**–**17** in moderate to favorable yields, alongside the medicinally relevant trifluoromethylated pyridines **18**–**21** with diverse substitution patterns. Following this, we turned our attention to aryl bromides. Both electron-rich and electron-deficient aryl bromides demonstrated the capability to yield the corresponding aryl amination products **22**–**25** with moderate efficiency, although the electron-rich aryl bromide **23** displayed a slightly reduced yield. Moreover, an array of heteroarenes, including pyridine **26**, quinoline **27**, and indole **28**, were proved to be suitable substrates for this transformation. Finally, the alkyl iodides were investigated as electrophiles in this transformation. Both secondary and primary alkyl iodides were amenable to single-electron reduction, yielding alkyl amination products **29**–**35** in moderate to good yields. Notably, in all cases, excellent regio- and diastereoselectivity were consistently observed, and the stereochemistry (**52**) was definitely confirmed by single-crystal X-ray crystallography.

A diverse range of nitrogen-based nucleophiles was systematically investigated, demonstrating the remarkable versatility of the reaction across various amine substrates. This included both primary and secondary amines, encompassing cyclic counterparts like piperidines (**36**–**39**) and piperazines (**40**–**47**), as well as thiomorpholine (**48**), pyrrolidine (**52**), acyclic secondary amines (**53** and **55**), and primary amines (**56**–**58**). These substrates participated in the three-component coupling, yielding the desired products in moderate to good yields (40% to 92%). More importantly, the medicinally relevant heteroarenes, such as furan (**45**), pyridine (**47**), thiophene (**50**), and ciprofloxacin ethyl ester (**59**), were all compatible with the standard conditions. Both primary and secondary amine functionalities were accommodated, highlighting the transformation’s versatility. Furthermore, the compatibility of free alcohol (**51**) and the NH group within the amide (**54**) underscored the reaction’s broad functional group tolerance. Notably, the nucleophilic scope was extended to carbon-, oxygen-, and sulfur-based nucleophiles. The incorporation of a carbon-based nucleophile, 2,2-dimethyl-1,3- dioxane-4,6-dione, as a coupling partner resulted in a 1,4-difluoromethyl alkylation product (**60**) with a 71% yield. Additionally, simple sodium phenoxide could serve as a nucleophile to furnish the corresponding product (**61**) in an acceptable yield, as well. Finally, the utilization of various sodium arylsulfinates (**62**–**64**) in the reactions yielded the targeted 1,4-difunctionalization products with moderate yields.

**Scheme 2:**
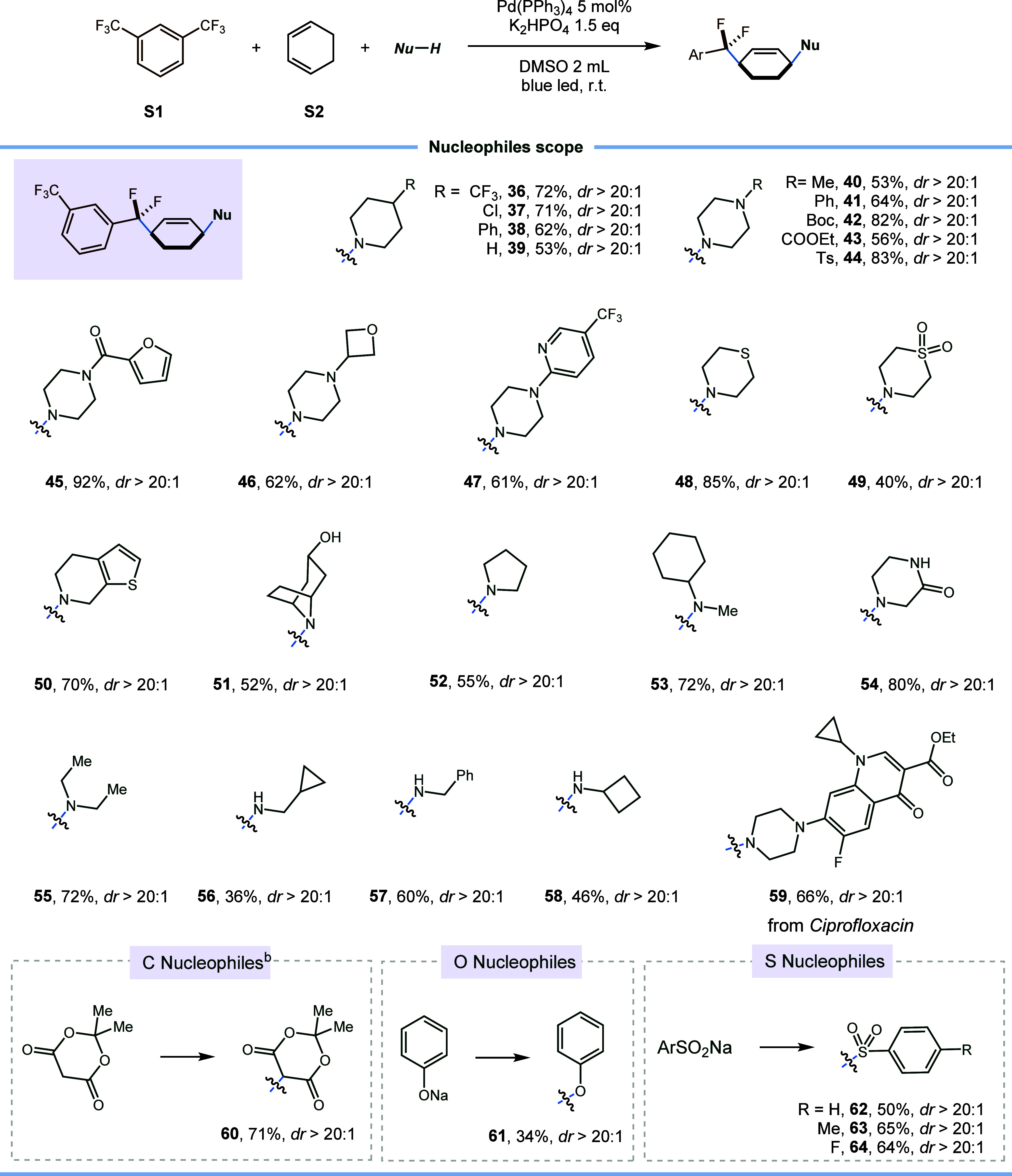
Scope of Nucleophiles 0.15 mmol of **S1**, 0.15 mmol of **S2**, 0.1 mmol of **Nu-H**, 5 mol % Pd(PPh_3_)_4_, 0.15 mmol of K_2_HPO_4_, DMSO (2 mL), 460 nm (10 W), 25 °C, 30 h. One additional equivalent of base was added.

To further highlight the versatility of this chemical transformation, we successfully synthesized a CFTR modulator (**66**) in just four steps. The key intermediate, *cis*-4-(difluoromethyl)cyclohex-2-en-1-amine (**65**), was efficiently accessed with exceptional diastereoselectivity from readily available starting materials. Additionally, the *cis*-TRPV6 inhibitor (**67**), which exhibits 10-fold activity compared to its trans isomer was synthesized via a two-step procedure from commercially available starting materials with excellent diastereoselectivity.

Moreover, this transformation allowed for the assembly of basic starting materials into biologically relevant compounds. Employing the aryl amination method, the diastereoisomer (**68**) of the inhibitor of 11β-HSD1 featuring a double bond was accessible. Subsequently, by employing palladium-catalyzed hydrogenation of the product, an analogue (**69**) of a DPP-4 inhibitor could be synthesized. Furthermore, this procedure offers a straightforward route to functionalized 1-N-iminosugars, with difluoromethylene-modified 1-N-iminosugar (**5**) being constructed via a straightforward dihydroxylation of the product. The stereochemistry (**68**, **70**) was unambiguously confirmed by single-crystal X-ray crystallography.

**Figure 2:**
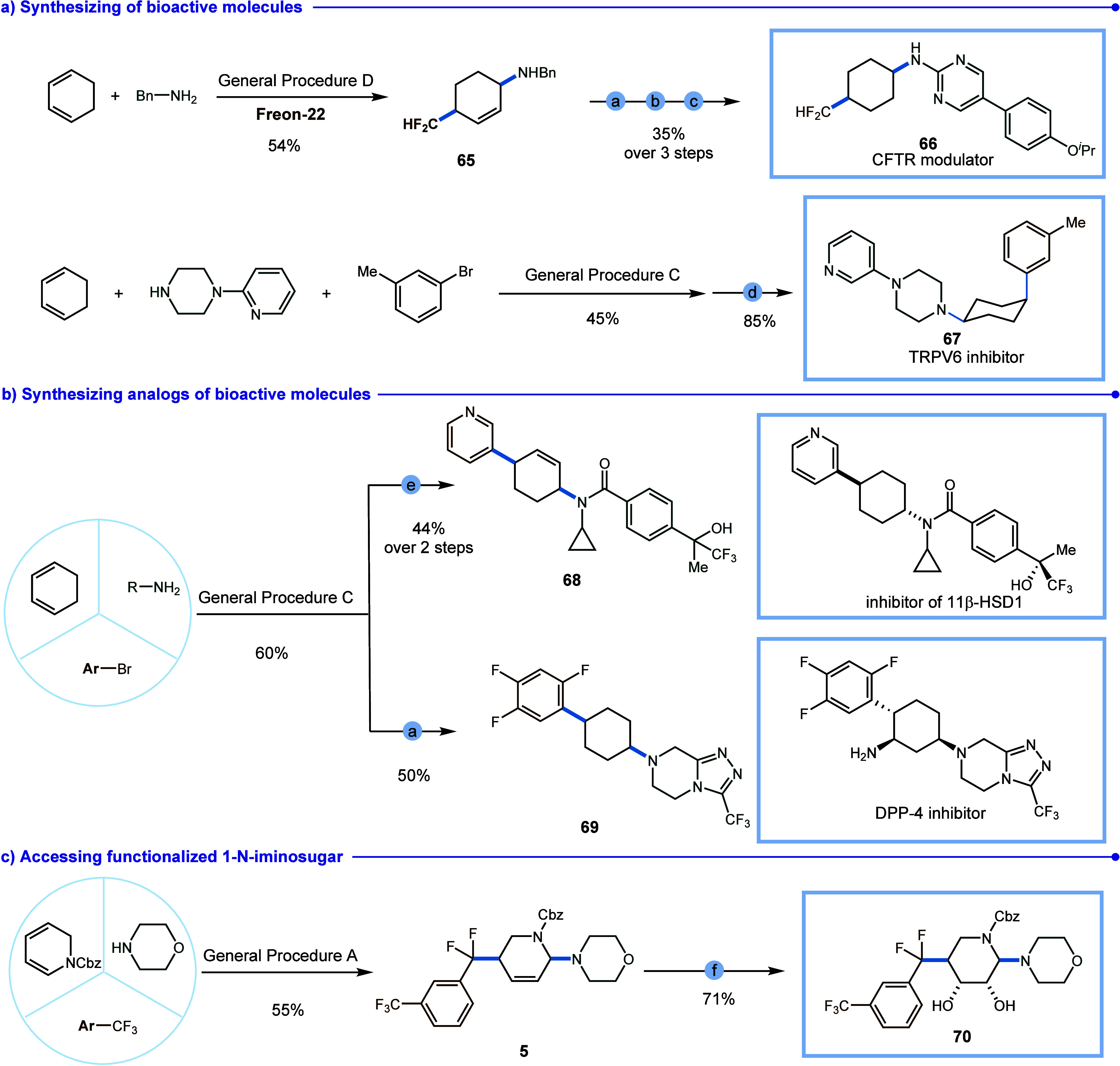
Synthetic utilities. Conditions: (a) HCOONH_4_ (5 equiv), Pd/C (10 mol %), MeOH (5 mL), 65 °C, 18 h; (b) TEA (3.2 equiv), 5-bromo-2-chloropyrimidine (1 equiv), 65 °C, 18 h; (c) 4-(trifluoromethoxy)phenylboronic acid (2 equiv), Na_2_CO_3_ (3 equiv), Pd(PPh_3_)_4_ (5 mol %), CH_3_CN/H_2_O (4:1). d) Pd/C (10 mol %), H_2_ (1 atm), MeOH (2 mL), rt, 18 h; (e) NaHCO_3_ (2.0 equiv), 4-(1,1,1-trifluoro-2-hydroxypropan-2-yl)benzoic acid (1.0 equiv), HOAt (1.3 equiv), EDCI (1.3 equiv), DMF (10 mL), 0–25 °C, 12 h. (f) NMO (2.0 equiv), K_2_OsO_4_·2H_2_O (10 mol %), t-BuOH/H_2_O (1:1), 0–25 °C, 12 h.

**Figure 3:**
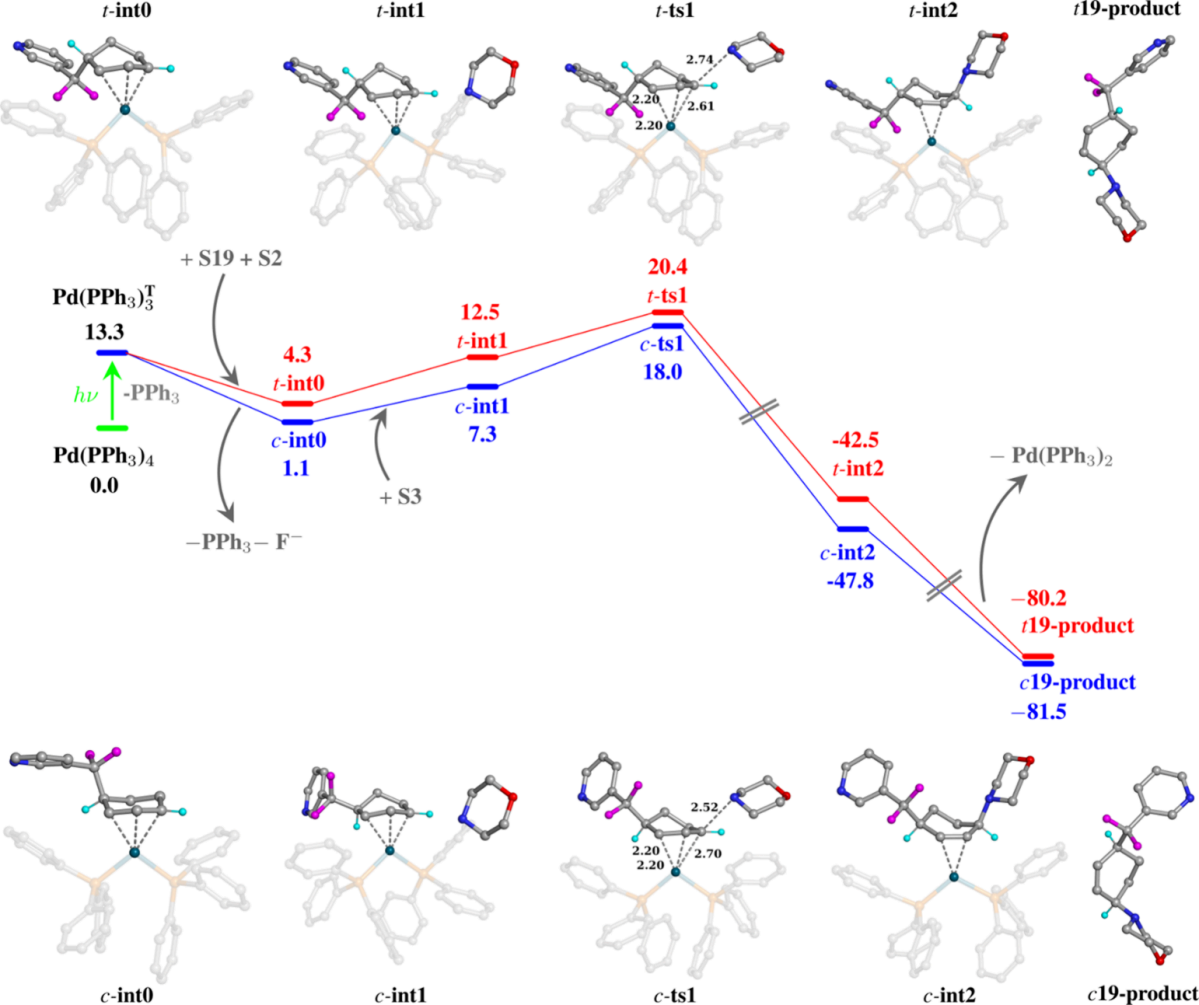
Gibbs free energy profile for the formation of cis- and trans-products via a stepwise alkene migration followed by nucleophilic substitution at the π-allylpalladium complex. Only the important H atoms (cyan color) are shown in the figure for clarity. The free energies are reported in kcal mol^–1^.

To understand the nature of the facial selectivity observed in the experiment, density functional theory (DFT) calculation was employed to calculate the energy profile of the reaction (details in the Supporting Information). As shown in Figure 3, the trifluoromethylated arene carbon radical reacts with the 1,3-diene moiety to give the allylic carbon radical, which coordinates with Pd(PPh_3_)_2_ via an π–π interaction (**int0**). The nucleophile attacks **int0** from the opposite direction of the catalyst, via a classical S_N_2′ mechanism that involves a prereaction complex (**int1**), a Walden inversion transition state (**ts1**), a postreaction complex (**int2**), and dissociated products (**product**). As the figure shows, the trans-pathway has overall higher energies than the cis-pathway with the rate-limiting step (*t*-**ts1** vs *c-***ts1**) 2.4 kcal/mol higher. According to the Curtin–Hammett principle and the Eyring–Polanyi equation, this level of difference results in a ratio of *t-***product** vs *c-***product** of 1:57, aligning closely with experimental findings.

**Figure 4:**
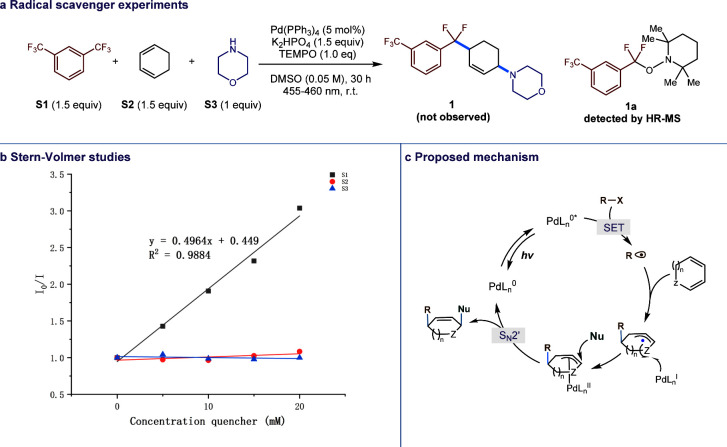
Mechanistic study.

The radical trap and Stern–Volmer quenching experiments were then performed. Control experiments utilizing TEMPO as a radical scavenger yielded no product formation with the observation of the ArCF_2_-TEMPO adduct (Figure 4a). Furthermore, Stern–Volmer quenching experiments (Figure 4b) revealed that only S1 effectively quenched the excited Pd(0) catalyst. Integrating these findings from radical scavenger experiments, Stern–Volmer results, and computational studies, we propose the following mechanism (Figure 4c). Upon exposure to blue LED irradiation, the Pd(0) catalyst is photoexcited, thus facilitating the donation of an electron to the electrophile. This electron transfer event generates a carbon radical and Pd(I) species. Subsequently, the carbon radical engages in an addition reaction with the 1,3-diene substrate to yield an allylic carbon radical. Notably, owing to steric considerations, the Pd(I) species exhibits a preference for recombination with the allylic radical from the back side. Ultimately, the nucleophile executes a nucleophilic attack on the carbon atom situated at the back of the palladium, leading to the formation of the syn-addition product.

## Conclusions

In summary, a novel strategy has been developed for the construction of 1,4-cis-substituted cyclic frameworks. This approach is enabled by a hybrid palladium-catalyzed radical-polar crossover mechanism to achieve reversed facial selectivity. Utilizing common resources such as cyclic 1,3-dienes, amines, and a diverse array of electrophiles (trifluoromethylaromatics, aryl bromides, and alkyl iodides), it realizes the synthesis of various 1,4-cis-substituted cyclic compounds with different ring sizes, spiro structures, and aza-heterocycles, maintaining remarkable diastereoselectivity. Significantly, this method offers a straightforward pathway for synthesizing biologically active compounds including pharmaceutical molecules and their derivatives.

## Supporting Information Available

Full experimental procedures, spectroscopic data, and detailed X-ray crystallographic data (PDF)
Transparent Peer Review report available (PDF)
