# Supplementary material for: Selective 1,4-syn-Addition to Cyclic 1,3-Dienes via Hybrid Palladium Catalysis
Source: ACS Cent Sci. 2024 May 15;10(6):1191–200. doi: 10.1021/acscentsci.4c00094 (PMC11212138; doi:10.1021/acscentsci.4c00094)
Supplement: Supplementary file 1 — oc4c00094_si_001.pdf [file oc4c00094_si_001.pdf]

Selective 1,4-*syn*-Addition to Cyclic 1,3-Dienes *via* Hybrid Palladium Catalysis

Yan Liang,[1,‡] Tiancen Bian,[2,‡] Komal Yadav,[2] Qixin Zhou,[1] Liejin Zhou,[1] Rui Sun[2] and Zuxiao Zhang[1,2]*

[1]Key Laboratory of the Ministry of Education for Advanced Catalysis Materials, College of Chemistry and Materials Science, Zhejiang Normal University, Jinhua, China. [2]Department of Chemistry, University of Hawai'i at Mānoa. Honolulu, HI, USA. Email: zzhang9@hawaii.edu

[‡]These authors contributed equally: Yan Liang, Tiancen Bian.

Table of Contents

I. General Information S2

II. Unsuccessful substrates S3

III. General Procedure for substrates S4

IV. General Procedure for 1,4-cis-disubstituted cyclohexenes S7

V. Detailed Optimization of Conditions S9

VI. Characterization of products S17

VII. Synthesis of drug and its analog S53

VIII. Mechanistic Experiments S59

IX. Computational Studies S66

X. Single crystal Data S96

XI. Spectral Data S123

XII. References S229

I. General Information

All chemicals and reagents were purchased from Bidepharm, Energy Chemical or J&K Scientific. All solvents were dried over 4Å molecular sieve prior use. Unless otherwise specified, all reactions were carried out under an atmosphere of N_2 in 10 mL vial sealed with parafilm. Flash column chromatography, or preparative thin-layer chromatography, was performed with Silicycle F60 (230-400 mesh) silica gel. Thin layer chromatography (TLC) analyses were performed using EMD 60 F254 TLC plates and visualized by fluorescence quenching, I_2 or $KMnO_4$ stain. All yields are averages of at least two experimental runs.

Nuclear magnetic resonance (NMR) spectra (^{1}H, ^{19}F, ^{13}C) were recorded using either a Bruker AVIII 400 or AVIII 600 MHz NMR spectrometer. ^{1}H and ^{13}C NMR chemical shifts are reported in parts per million and referenced to residual $CHCl_3$ signals in $CDCl_3$ (^{1}H: δ 7.26; ^{13}C: δ 77.2). ^{1}H NMR data are reported as follows: chemical shifts (δ ppm), multiplicity (s = singlet, d = doublet, t = triplet, q = quartet, m = multiplet, b = broad, ap = apparent), coupling constant (Hz), relative integral. Data for ^{13}C and ^{19}F NMR are reported in terms of chemical shift and multiplicity where appropriate. High-resolution mass spectrometry (HRMS) analysis was carried out using a TOF MS instrument with an APCI or ESI, EI source. Single-crystal X-ray diffraction studies were conducted at 293 K on the Bruker D8 VENTURE diffractometer equipped with a PHOTON-II detector (MoKα, λ = 0.71073 Å or CuKα, λ = 1.54178). An oil bath was used for heating when was needed. Column chromatography was performed using silica gel (300-400 mesh).

Photochemical reactions were performed by placing reaction vessel on the 10 W lamp panel (455-460 nm) See a photograph of the set-up below.

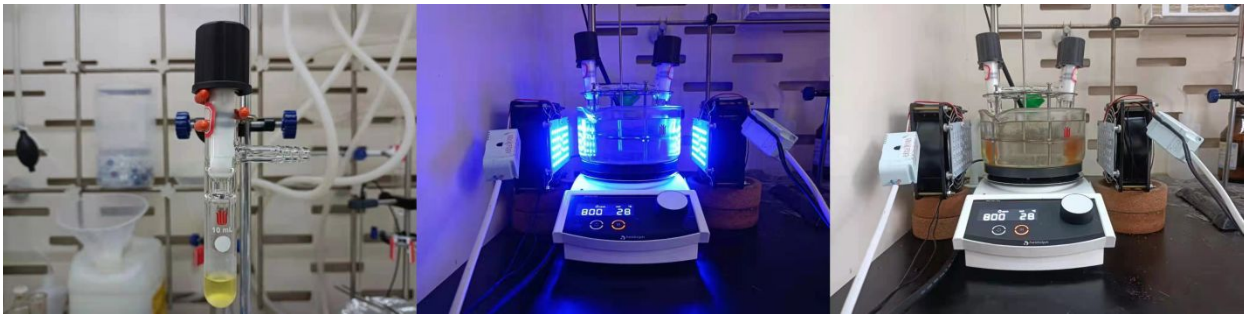

Figure S1. The reaction set up apparatus.

Ⅱ. Unsuccessful substrates

Nucleophile:

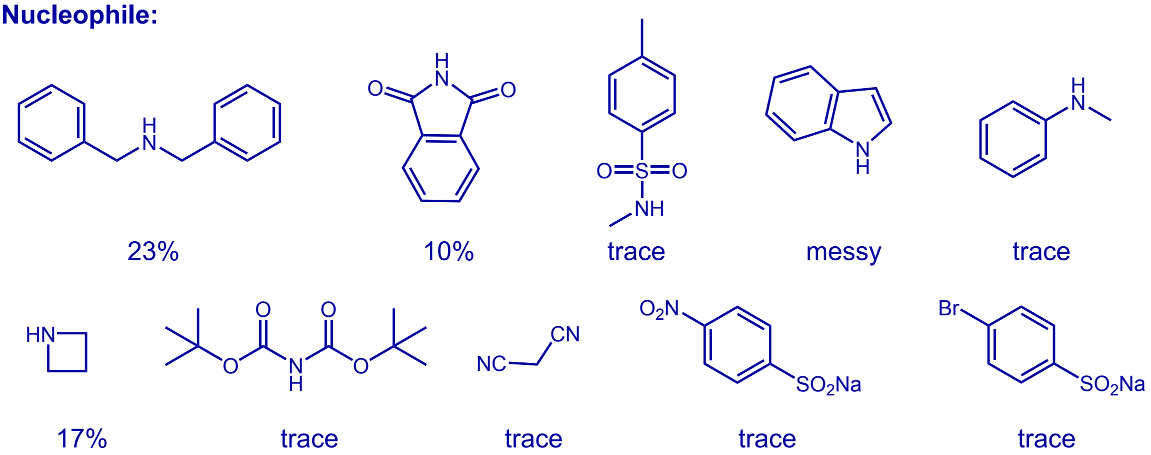

Electrophile:

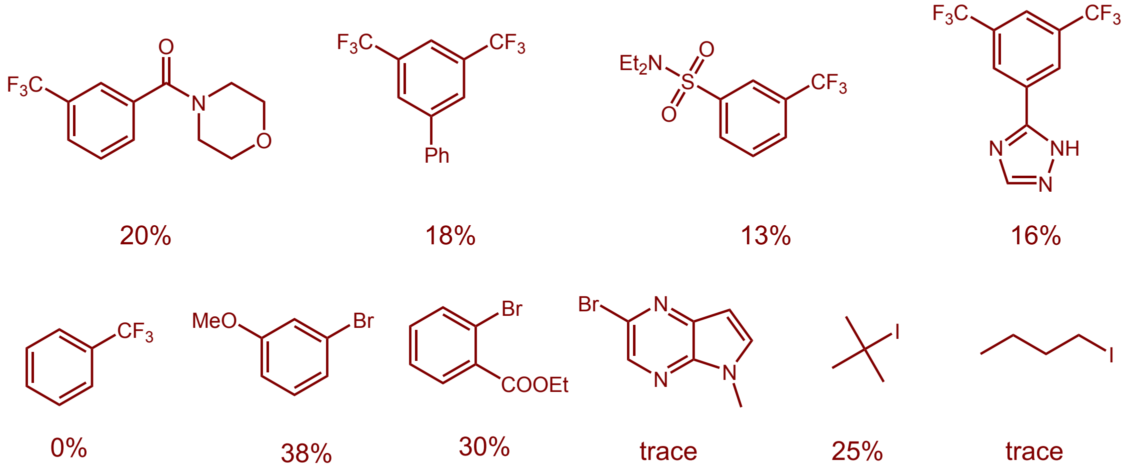

Diene:

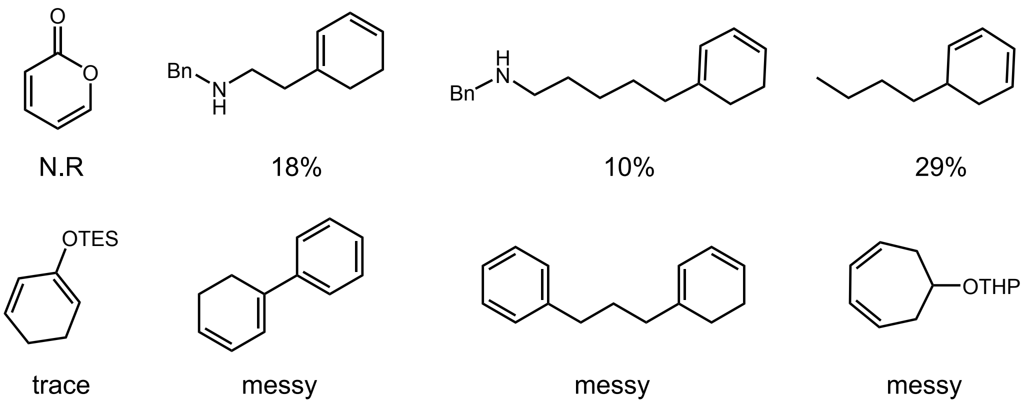

III. General Procedure

3.1 General procedure for substituted dihydropyridine S5-S10

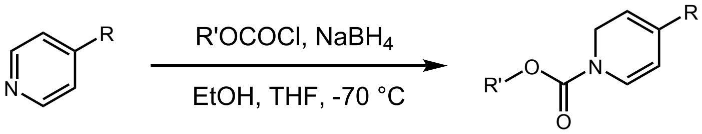

Chloroformate (20.0 mmol, 1.0 eq) in THF (10 mL) was added dropwise under nitrogen to a mixture of $NaBH_4$ (20.0 mmol, 1.0 eq), substituted pyridine (20.0 mmol, 1.0 eq) in EtOH (5 mL) and THF (40 mL) at −78 °C. The reaction was stirred at −78 °C or 3 h and then quenched by saturated aqueous NH_4Cl solution (50 ml). The mixture was extracted with DCM (3×30 ml). The combined organic layers were dried over Na_2SO_4 and concentrated under reduced pressure to give the residue, which was purified by column chromatography (petroleum ether/ethyl acetate = 100/1-10/1) to give product as oil or solid (The substrates **S5-S10** were synthesized according to published procedures). The spectral data of the substrates **S5-S8** were consisted with that reported in the literature [1–3].

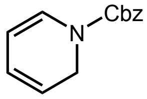

S5

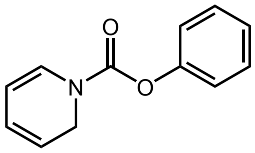

S6

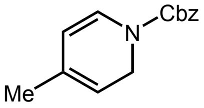

S7

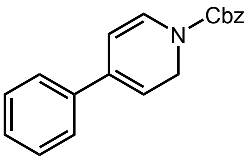

S8

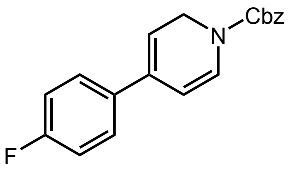

S9

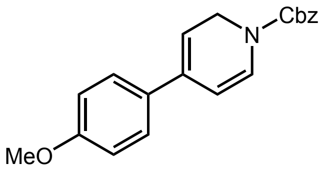

S10

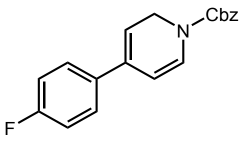

S9

^{1}H NMR (400 MHz, $CDCl_3$) δ 7.50 – 7.34 (m, 5H), 7.34 – 7.27 (m, 2H), 7.08 – 6.98 (m, 2H), 6.92 (dd, J = 36.3, 8.0 Hz, 1H), 5.77 – 5.58 (m, 1H), 5.49 (dd, J = 32.9, 8.1 Hz, 1H), 5.23 (s, 2H), 4.53 (d, J = 4.3 Hz, 2H). **^{13}C NMR** (101 MHz, $CDCl_3$) δ 162.37 (d, J = 246.7 Hz), 135.88, 134.82 (d, J = 3.5 Hz), 128.59, 128.35, 128.16, 127.38, 127.00 (d, J = 8.0 Hz), 115.34 (d, J = 21.4 Hz), 114.57, 114.08, 105.90, 67.95, 44.01 (d, J = 19.9 Hz). **^{19}F NMR** (377 MHz, $CDCl_3$) δ -114.63 – -114.84 (m).

HRMS (ESI) for $C_{19}H_{16}FNO_2$ $[M+H]^+$ m/z: calcd. 310.1238, found 310.1230.

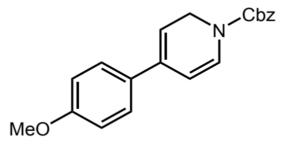

S10

^{1}H NMR (400 MHz, $CDCl_3$) δ 7.42 – 7.33 (m, 5H), 7.33 – 7.27 (m, 2H), 7.00 – 6.82 (m, 3H), 5.75 – 5.43 (m, 2H), 5.23 (s, 2H), 4.52 (d, J = 4.3 Hz, 2H), 3.81 (s, 3H). **^{13}C NMR** (101 MHz, $CDCl_3$) δ 159.21, 135.96, 131.24, 128.57, 128.30, 128.13, 127.07, 126.47, 126.30, 113.86, 113.25, 106.62 – 106.09 (m), 67.86, 55.28, 43.95.

HRMS (ESI) for $C_{20}H_{19}NO_3$ $[M+H]^+$ m/z: calcd. 322.1438, found 322.1429.

3.2 General procedure for substituted diene S11-S12

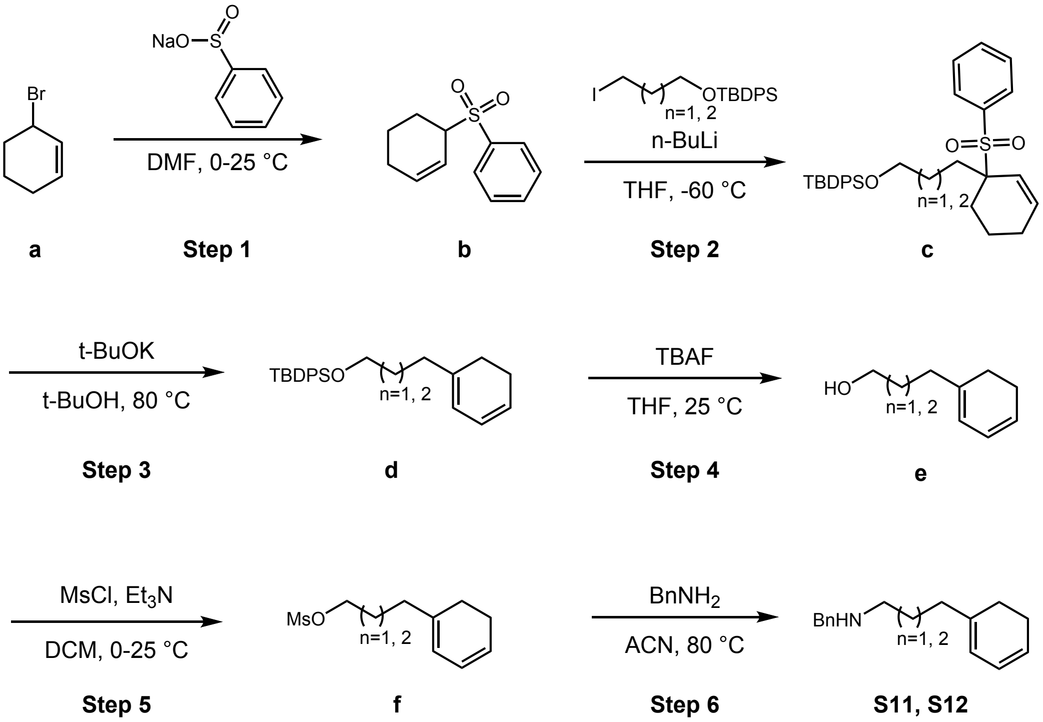

The substrates **S11** and **S12** were synthesized according to published procedures. The spectral data of the substrates were consisted with that reported in the literature4.

Note: The prepared 1,3-diene should be stored at low temperature and protected from light, and used as soon as possible.

Step 1: Synthesis of b

To a solution of 3-bromocyclohex-1-ene (4.00 g, 25.0 mmol) in DMF (40 mL) at 0 °C was added sodium benzensulfinate (8.00 g, 50.0 mmol). The reaction mixture was stirred at 25 °C for 24 h and TLC showed the reaction was complete. Then the mixture was poured into water (50 mL). The aqueous phase was extracted with EtOAc (3×60 mL) and the combined organic

phases were washed with brine (5×40 mL), dried over Na_2SO_4, filtered and concentrated under reduced pressure to give a residue, which was purified by column chromatography (petroleum ether/ethyl acetate = 50:1) to afford the product **b** as white solid (5.10 g, 91.6% yield).

Step 2: Synthesis of c

To a solution of **b** (6.00 g, 27.0 mmol) in THF (60 mL) was added n-BuLi (12.0 mL, 29.7 mmol, 2.5 M) dropwise at -60 °C and the mixture stirred at this temperature for 0.5 h. Then tert-butyl(3-iodopropoxy) diphenylsilane (12.2 g, 29.7 mmol) in THF (250 mL) was added to the above mixture dropwise at -60 °C and the cooling bath was removed. The reaction mixture was stirred for an additional hour and TLC showed the reaction was complete. Then the mixture was poured into saturated aqueous NH_4Cl solution (100 mL) and was extracted with EtOAc (3×80 mL). The combined organic phases were washed with brine (3×50 mL), dried over Na_2SO_4. filtered and concentrated under reduced pressure to give a residue, which was purified by column chromatography (petroleum ether/ethyl acetate = 100:1) to afford the product **c** as yellow oil (11.0 g, 78.0% yield).

Step 3: Synthesis of d

A mixture of **S3** (1.00 g, 2.00 mmol) and t-BuOK (500 mg, 4.40 mmol) in t-BuOH (10 mL) was stirred at 80 °C for 11 h. TLC showed the reaction was complete and quenched with brine (10 mL). The aqueous layer was extracted with EtOAc (3×30 mL). The combined organic phases were washed with brine (4×30 mL), dried over Na_2SO_4. filtered and concentrated under reduced pressure to give the crude product **d**, which was used directly in the next step (800 mg, crude).

Step 4: Synthesis of e

A solution of TBAF (5.00 mmol, 5 mL, 1 M in THF) was added to **d** (800 mg, crude) and the reaction mixture stirred at 20 °C for 12 h. The reaction mixture was quenched with brine (10 mL) and extracted with ethyl acetate (20 mL×3). The combined organic layers were dried over anhydrous Na_2SO_4, filtered and concentrated under reduced pressure to give a residue, which was purified by column chromatography (petroleum ether/EtOAc = 10:1) to afford **e** as yellow oil (193 mg, 70% yield over two steps).

Step 5: Synthesis of f

Methanesulfonyl chloride (2.50 g, 22 mmol) was added to a solution of **e** (1.38 g, 10.0 mmol) and Et_3N (3.7 mL, 27 mmol) in anhydrous CH_2Cl_2 (15 mL) dropwise at 0 °C. The mixture was warmed to room temperature and stirred for 3 hours. The mixture was concentrated under reduced pressure to give the crude product **f**, which was used directly in the next step, and the

yield was assumed to be 100%.

Step 6: Synthesis of S11

To the crude **f** was added benzylamine (5.30 g, 50 mmol) in anhydrous CH_3CN (30 mL). and the reaction mixture was stirred at 80 °C. for 12 h. TLC showed the reaction was complete and quenched with brine (40 mL). The aqueous layer was extracted with EtOAc (3×40 mL). The combined organic phases were washed with brine (3×40 mL), dried over Na_2SO_4. filtered and concentrated under reduced pressure to give the residue, which was purified by column chromatography (petroleum ether/ethyl acetate = 5/1) to give **S11** as yellow oil (1.13 g, 50% over two steps).

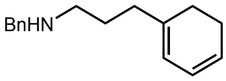

S11

^{1}H NMR (400 MHz, $CDCl_3$) δ 7.35 – 7.29 (m, 4H), 7.26 – 7.23 (m, 1H), 5.92 – 5.82 (m, 1H), 5.71 – 5.61 (m, 2H), 3.79 (s, 2H), 2.71 – 2.58 (m, 2H), 2.22 – 2.06 (m, 6H), 1.72 – 1.66 (m, 2H), 1.65 (s, 1H).

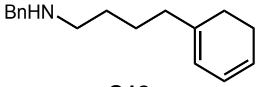

S12

The preparation of substrate **S12** is analogous to **S11**.

^{1}H NMR (600 MHz, $CDCl_3$) δ 7.36 – 7.30 (m, 4H), 7.27 – 7.23 (m, 1H), 5.90 – 5.80 (m, 1H), 5.71 – 5.57 (m, 2H), 3.79 (s, 2H), 2.64 (t, J = 7.0 Hz, 2H), 2.24 – 2.12 (m, 2H), 2.10 – 2.02 (m, 4H), 1.55 – 1.45 (m, 5H).

IV. General Procedure for 1,4-cis-disubstituted cyclohexenes

4.1 General Procedure A

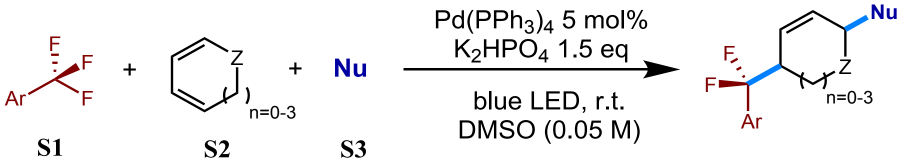

To a 10 mL Schlenk tube, containing a stirring bar, was added $Pd(PPh_3)_4$ (5.70 mg, 0.005 mmol), K_2HPO_4 (26.0 mg, 0.15 mmol), dry DMSO (2 mL), **S1** (0.15 mmol), **S2** (0.15 mmol) and **S3** (0.1 mmol) (**NOTE**: one additional equivalent base was used when **S3** was HCl salt form) consecutively in a glovebox. The mixture was then sealed, degassed by using a "freeze–pump–thaw" procedure for 3 times and then irradiated with 10 w blue LEDs (10 w, 455-460 nm) with fan at room temperature (about 25 °C) for 30 h. After the reaction completed, the mixture was quenched with brine (2 mL) and extracted with EtOAc (2×2 mL). The combined

organic layers were washed with brine (2×2 mL), dried over Na_2SO_4 and concentrated *in vacuo*. The crude material was then purified by column chromatography on silica gel using the noted solvent mixture to furnish the desired product.

4.2 General Procedure B

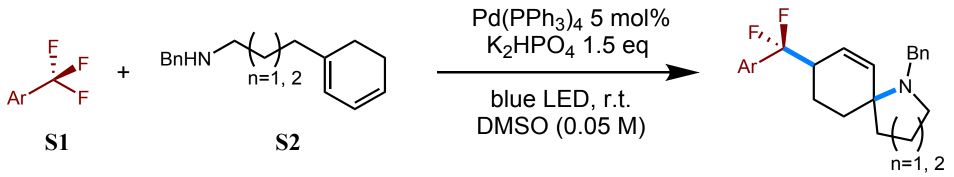

To a 10 mL Schlenk tube, containing a stirring bar, was added $Pd(PPh_3)_4$ (5.70 mg, 0.005 mmol), K_2HPO_4 (26.0 mg, 0.15 mmol), dry DMSO (2 mL), **S1** (0.15 mmol) and **S2** (0.1 mmol) consecutively in a glovebox. The mixture was then sealed, degassed by using a "freeze–pump–thaw" procedure for 3 times and then irradiated with 10 w blue LEDs (10 w, 455-460 nm) with fan at room temperature (about 25 °C) for 30 h. After the reaction completed, the mixture was quenched with brine (2 mL) and extracted with EtOAc (2×2 mL). The combined organic layers were washed with brine (2×2 mL), dried over Na_2SO_4 and concentrated in vacuo. The crude material was then purified by column chromatography on silica gel using the noted solvent mixture to furnish the desired product.

4.3 General Procedure C

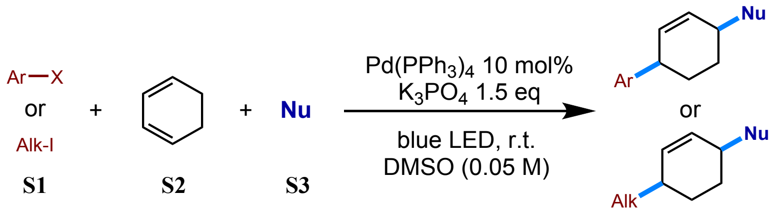

To a 10 mL Schlenk tube, containing a stirring bar, was added $Pd(PPh_3)_4$ (11.4 mg, 0.010 mmol), K_3PO_4 (31.8 mg, 0.15 mmol), dry DMSO (2 mL), **S1** (0.15 mmol), **S2** (45 uL, 0.45 mmol) and **S3** (0.1 mmol) consecutively in a glovebox. The mixture was then sealed, degassed by using a "freeze–pump–thaw" procedure for 3 times and then irradiated with 10 w blue LEDs (10 w, 455-460 nm) with fan at room temperature (about 25 °C) for 20 h. After the reaction completed, the mixture was quenched with brine (2 mL) and extracted with EtOAc (2×3 mL). The combined organic layers were washed with brine (3×2 mL), dried over $MgSO_4$ and concentrated in vacuo. The crude material was then purified by column chromatography on silica gel using the noted solvent mixture to furnish the desired product.

4.4 General Procedure D

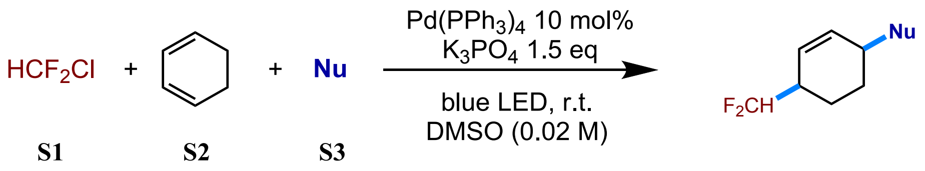

To a solution of **S3** (0.2 mmol) and K_3PO_4 (64 mg, 0.3 mmol) in dry DMSO (4 mL) was added $Pd(PPh_3)_4$ (24 mg, 0.02 mmol) in the glove box. The mixture was bubbled with HCF_2Cl balloon for 5 mins, after which **S2** (0.5 mmol) was added to the above solution. Then the reaction was sealed with parafilm and irradiated at 5 w blue LEDs (455-460 nm) with fan at room temperature (about 25 °C) for 12 h. After the reaction completed, the mixture was quenched with brine (4 mL) and extracted with EtOAc (3×4 mL). The combined organic layers were washed with brine (4×3 mL), dried over Na_2SO_4 and concentrated in vacuo. The crude material was then purified by column chromatography on silica gel using the noted solvent mixture to furnish the desired product.

V. Detailed Optimization of Conditions

Table S1. Investigation of photocatalysts.

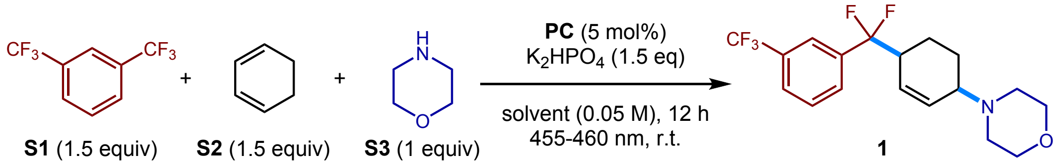

entry[a]	PC	solvent	T/°C	Time/h	**1**/yield/%[b]
1	$Pd(PPh_3)_4$	DMSO	25	12	82
2	$Pd(OAc)_2$	DMSO	25	12	0
3	$Pd(acac)_2$	DMSO	25	12	0
4	$Pd(dba)_2$	DMSO	25	12	0
5	$Pd(OPiv)_2$	DMSO	25	12	0
6	$PdCl_2(dppe)$	DMSO	25	12	13
7[c]	/	DMSO	26	12	0

[a]0.15 mmol **S1**, 0.15 mmol **S2**, 0.1 mmol **S3**,5 mol% **PC** and 0.15 mmol K_2HPO_4 in dry DMSO (2 mL) irradiated at 10 w 455-460 nm lamp panel at room temperature for 12 h. [b]Yield determined by ^{1}H-NMR using 2,2,2-Trifluoro-N,N-dimethylacetamide as an external standard. [c]Without photocatalyst.

Table S2. Investigation of solvents.

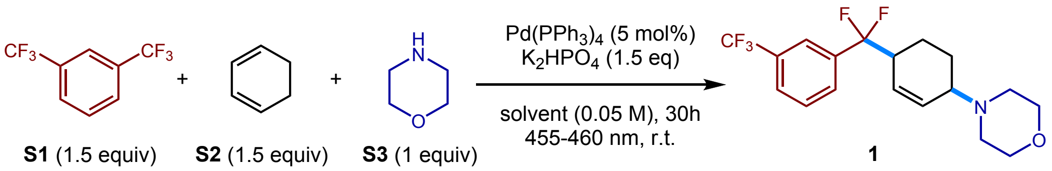

S1 (1.5 equiv) **S2** (1.5 equiv) **S3** (1 equiv) **1**

entry[a]	PC	solvent	T/°C	Time/h	**1**/yield/%[b]
1	$Pd(PPh_3)_4$	DMSO	25	30	88
2	$Pd(PPh_3)_4$	DMF	25	30	88
3	$Pd(PPh_3)_4$	Toluene	25	30	trace
4	$Pd(PPh_3)_4$	1,4-dioxane	25	30	trace
5	$Pd(PPh_3)_4$	EtOAc	25	30	25
6	$Pd(PPh_3)_4$	DCE	25	30	trace

[a]0.15 mmol **S1**, 0.15 mmol **S2**, 0.1 mmol **S3**,5 mol% $Pd(PPh_3)_4$ and 0.15 mmol K_2HPO_4 in dry solvent (2 mL) irradiated at 10 w 455-460 nm lamp panel at room temperature for 30 h. [b]Yield determined by ^{1}H-NMR using 2,2,2-Trifluoro-N,N-dimethylacetamide as an external standard.

Table S3. Investigation of bases.

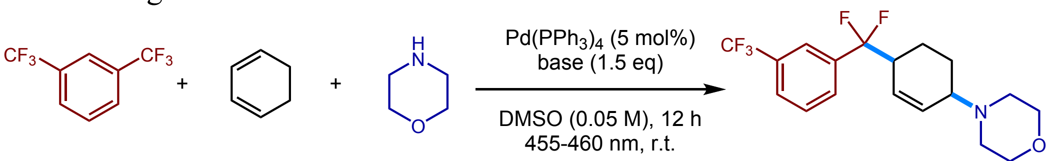

S1 (1.5 equiv) **S2** (1.5 equiv) **S3** (1 equiv) **1**

entry[a]	PC	base	T/°C	Time/h	**1**/yield/%[b]
1	$Pd(PPh_3)_4$	CH_3COONa	25	12	58
2	$Pd(PPh_3)_4$	NaH_2PO_4	25	12	40
3	$Pd(PPh_3)_4$	Cs_2CO_3	25	12	0
4	$Pd(PPh_3)_4$	Na_2CO_3	25	12	48
5	$Pd(PPh_3)_4$	$KHCO_3$	25	12	49
6	$Pd(PPh_3)_4$	K_2HPO_4	25	12	82
7[c]	$Pd(PPh_3)_4$	/	25	12	60

[a]0.15 mmol **S1**, 0.15 mmol **S2**, 0.1 mmol **S3**,5 mol% $Pd(PPh_3)_4$ and 0.15 mmol base in dry DMSO (2 mL) irradiated at 10 w 455-460 nm lamp panel at room temperature for 12 h. [b]Yield determined by ^{1}H-NMR using 2,2,2-Trifluoro-N,N-dimethylacetamide as an external standard. [c]Without base.

Table S4. Investigation of the equivalent of K_2HPO_4.

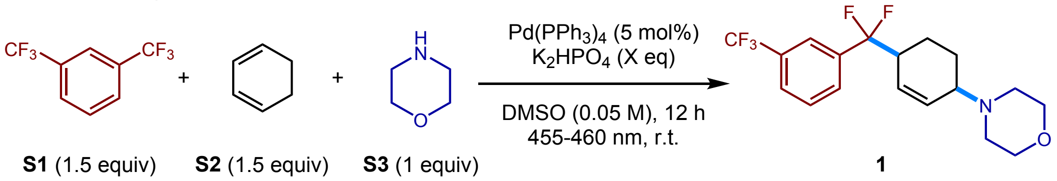

S1 (1.5 equiv) **S2** (1.5 equiv) **S3** (1 equiv) **1**

entry[a]	PC	Equivalent of K_2HPO_4	T/°C	Time/h	**1**/yield/%[b]
1	$Pd(PPh_3)_4$	1.0	25	12	68
2	$Pd(PPh_3)_4$	1.5	25	12	82
3	$Pd(PPh_3)_4$	2.0	25	12	72
4	$Pd(PPh_3)_4$	3.0	25	12	57

5	$Pd(PPh_3)_4$	4.0	25	12	75

[a]0.15 mmol **S1**, 0.15 mmol **S2**, 0.1 mmol **S3**,5 mol% $Pd(PPh_3)_4$ and X mmol K_2HPO_4 in dry DMSO (2 mL) irradiated at 10 w 455-460 nm lamp panel at room temperature for 12 h. [b]Yield determined by ^{1}H-NMR using 2,2,2-Trifluoro-N,N-dimethylacetamide as an external standard.

Table S5. Investigation of the equivalent of $Pd(PPh_3)_4$.

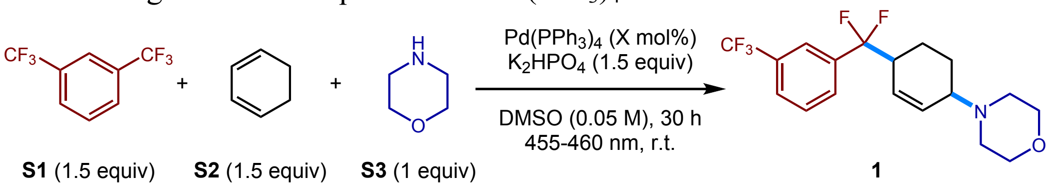

entry[a]	PC	X	T/°C	Time/h	**1**/yield/%[b]
1	$Pd(PPh_3)_4$	2.5	25	30	86
2	$Pd(PPh_3)_4$	5	25	30	88
3	$Pd(PPh_3)_4$	7.5	25	30	71
4	$Pd(PPh_3)_4$	10	25	30	78

[a]0.15 mmol **S1**, 0.15 mmol **S2**, 0.1 mmol **S3,** X mol% $Pd(PPh_3)_4$ and 0.15 mmol K_2HPO_4 in dry DMSO (2 mL) irradiated at 10 w 455-460 nm lamp panel at room temperature for 30 h. [b]Yield determined by ^{1}H-NMR using 2,2,2-Trifluoro-N,N-dimethylacetamide as an external standard.

Table S6. Investigation of the concentration.

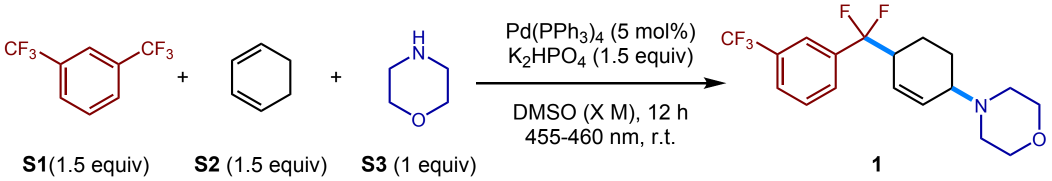

entry[a]	X	DMSO/y mL	T/°C	Time/h	**1**/yield/%[b]
1	0.1	1	25	12	44
2	0.05	2	25	12	82
3	0.03	3	25	12	73
4	0.025	4	25	12	74

[a]0.15 mmol **S1**, 0.15 mmol **S2**, 0.1 mmol **S3**,5 mol% $Pd(PPh_3)_4$ and 0.15 mmol K_2HPO_4 in dry DMSO (y mL) irradiated at 10 w 455-460 nm lamp panel at room temperature for 12 h. [b]Yield determined by ^{1}H-NMR using 2,2,2-Trifluoro-N,N-dimethylacetamide as an external standard.

Table S7. Investigation of the ratio of **S1**.

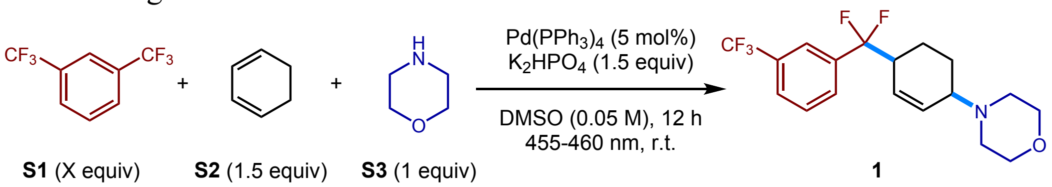

entry[a]	PC	X	T/°C	Time/h	**1**/yield/%[b]
1	$Pd(PPh_3)_4$	1	25	12	56
2	$Pd(PPh_3)_4$	1.5	25	12	82
3	$Pd(PPh_3)_4$	2	25	12	75

4	$Pd(PPh_3)_4$	3	25	12	82
5	$Pd(PPh_3)_4$	4	25	12	84

[a]0.1*X mmol **S1**, 0.15 mmol **S2**, 0.1 mmol **S3**,5 mol% $Pd(PPh_3)_4$ and 0.15 mmol K_2HPO_4 in dry DMSO (2 mL) irradiated at 10 w 455-460 nm lamp panel at room temperature for 12 h. [b]Yield determined by ^{1}H-NMR using 2,2,2-Trifluoro-N,N-dimethylacetamide as an external standard.

Table S8. Investigation of the ratio of **S2**.

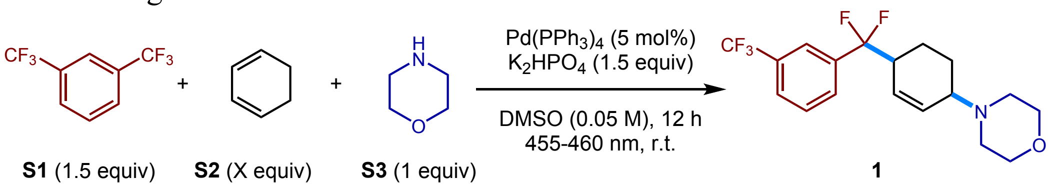

entry[a]	PC	X	T/°C	Time/h	**1**/yield/%[b]
1	$Pd(PPh_3)_4$	1	25	12	69
2	$Pd(PPh_3)_4$	1.5	25	12	82
3	$Pd(PPh_3)_4$	2	25	12	80
4	$Pd(PPh_3)_4$	3	25	12	91
5	$Pd(PPh_3)_4$	4	25	12	88

[a]0.15 mmol **S1**, 0.1*X mmol **S2**, 0.1 mmol **S3**,5 mol% $Pd(PPh_3)_4$ and 0.15 mmol K_2HPO_4 in dry DMSO (2 mL) irradiated at 10 w 455-460 nm lamp panel at room temperature for 12 h. [b]Yield determined by ^{1}H-NMR using 2,2,2-Trifluoro-N,N-dimethylacetamide as an external standard.

Table S9. Investigation of the ratio of **S3**.

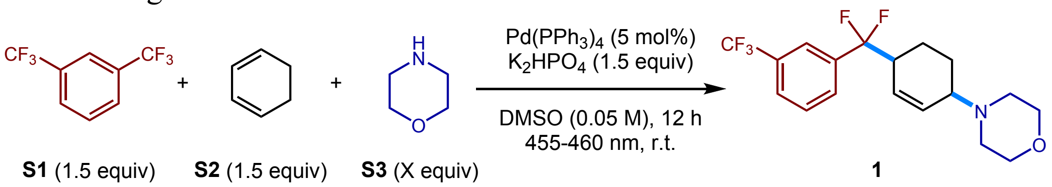

entry[a]	PC	X	T/°C	Time/h	**1**/yield/%[b]
1	$Pd(PPh_3)_4$	1	25	12	82
2	$Pd(PPh_3)_4$	2	25	12	85
3	$Pd(PPh_3)_4$	3	25	12	83
4	$Pd(PPh_3)_4$	4	25	12	76
5	$Pd(PPh_3)_4$	5	25	12	76

[a]0.15 mmol **S1**, 0.15 mmol **S2**, 0.1*X mmol **S3**,5 mol% $Pd(PPh_3)_4$ and 0.15 mmol K_2HPO_4 in dry DMSO (2 mL) irradiated at 10 w 455-460 nm lamp panel at room temperature for 12 h. [b]Yield determined by ^{1}H-NMR using 2,2,2-Trifluoro-N,N-dimethylacetamide as an external standard.

Table S10. Investigation of the light sources.

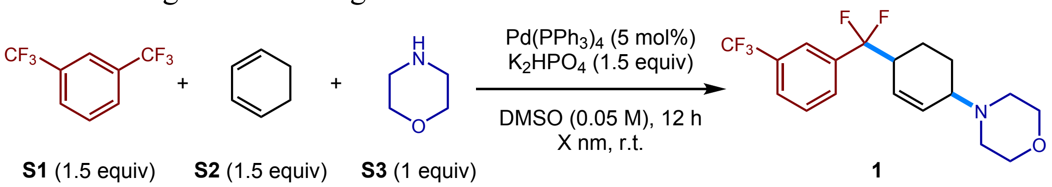

entry[a]	**PC**	Light source	T/°C	Time/h	**1**/yield/%[b]
1	$Pd(PPh_3)_4$	365-375	25	12	71
	$Pd(PPh_3)_4$	390-400	25	12	72
2	$Pd(PPh_3)_4$	420-430	25	12	73
3	$Pd(PPh_3)_4$	455-460	25	12	82
4	$Pd(PPh_3)_4$	475-485	25	12	81
5[c]	$Pd(PPh_3)_4$	dark	25	12	0

[a]0.15 mmol **S1**, 0.15 mmol **S2**, 0.1 mmol **S3**,5 mol% $Pd(PPh_3)_4$ and 0.15 mmol K_2HPO_4 in dry DMSO (2 mL) irradiated at 10 w X nm lamp panel at room temperature for 12 h. [b]Yield determined by ^{1}H-NMR using 2,2,2-Trifluoro-N,N-dimethylacetamide as an external standard.[c]Without light source.

Table S11. Investigation of the ligands.

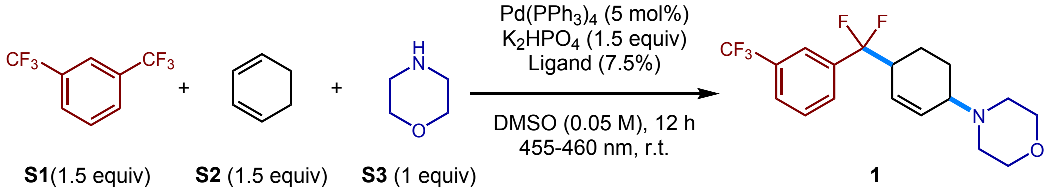

entry[a]	**PC**	Ligand	T/°C	Time/h	**1**/yield/%[b]
1	$Pd(PPh_3)_4$	Tri(*p*-tolyl)phosphine	25	12	64
2	$Pd(PPh_3)_4$	DPEphos	25	12	64
3	$Pd(PPh_3)_4$	Tri(1-naphthyl)phosphine	25	12	83
4	$Pd(PPh_3)_4$	Xantphos	25	12	61
5	$Pd(PPh_3)_4$	(R)-BINAP	25	12	30

[a]0.15 mmol **S1**, 0.15 mmol **S2**, 0.1 mmol **S3**,5 mol% $Pd(PPh_3)_4$ 0.15 mmol K_2HPO_4 and 7.5 mol% Ligand in dry DMSO (2 mL) irradiated at 10 w 455-460 nm lamp panel at room temperature for 12 h. [b]Yield determined by ^{1}H-NMR using 2,2,2-Trifluoro-N,N-dimethylacetamide as an external standard.

Table S12. Investigation of the temperature.

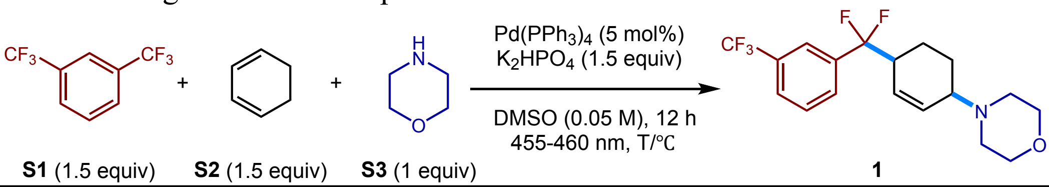

entry[a]	**PC**	T/°C	Time/h	**1**/yield/%[b]
1	$Pd(PPh_3)_4$	25	12	82
	$Pd(PPh_3)_4$	40	12	83
2	$Pd(PPh_3)_4$	60	12	73
3	$Pd(PPh_3)_4$	80	12	55
4	$Pd(PPh_3)_4$	100	12	19

[a]0.15 mmol **S1**, 0.15 mmol **S2**, 0.1 mmol **S3**,5 mol% $Pd(PPh_3)_4$ and 0.15 mmol K_2HPO_4 in dry DMSO (2 mL) irradiated at 10 w 455-460 nm lamp panel at T °C for 12 h. [b]Yield determined by ^{1}H-NMR using 2,2,2-Trifluoro-N,N-dimethylacetamide as an external standard.

Table S13. Investigation of the time.

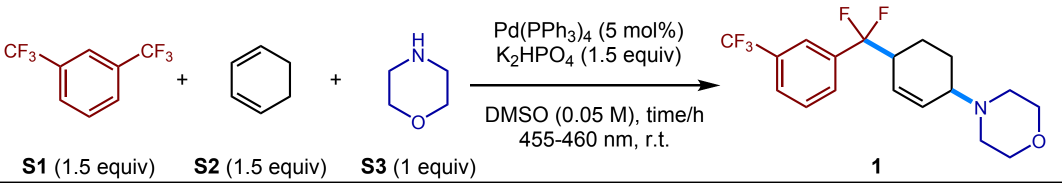

S1 (1.5 equiv) **S2** (1.5 equiv) **S3** (1 equiv) **1**

entry[a]	PC	T/°C	Time/h	**1**/yield/%[b]
1	$Pd(PPh_3)_4$	25	6	83
2	$Pd(PPh_3)_4$	25	12	77
3	$Pd(PPh_3)_4$	25	15	83
4	$Pd(PPh_3)_4$	25	24	80
5	$Pd(PPh_3)_4$	25	30	89

[a]0.15 mmol **S1**, 0.15 mmol **S2**, 0.1 mmol **S3**,5 mol% $Pd(PPh_3)_4$ and 0.15 mmol K_2HPO_4 in dry DMSO (2 mL) irradiated at 10 w 455-460 nm lamp panel at room temperature (25 °C) for t h. [b]Yield determined by ^{1}H-NMR using 2,2,2-Trifluoro-N,N-dimethylacetamide as an external standard.

Table S14. Investigation of solvents.

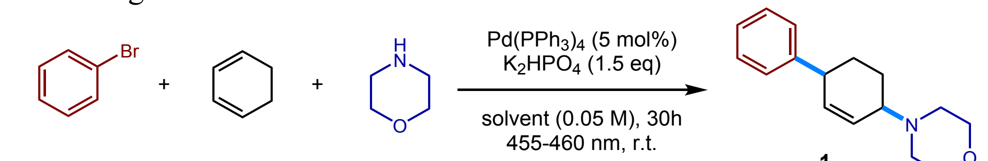

S1 (1.5 equiv) **S2** (1.5 equiv) **S3** (1 equiv) **1**

entry[a]	solvent	**1d**/yield/%[b]	entry[a]	solvent	**1**/yield/%[b]
1	DMF	trace	12	1,4-dioxane	trace
2	DME	trace	13	NMP	0
3	DMSO	34	14	EtOAc	trace
4	THF	trace	15	PhF	trace
5	DCM	trace	16	PhCl	0
6	DMA	0	17	1,3-dioxolane	trace
7	PhH	trace	18	$CHCl_3$	0
8	PhCN	0	19	HFIP	0
9	CH_3CN	trace	20	MeOH	trace
10	DCE	trace	21	2-Me THF	trace
11	MTBE	trace	22	Toluene	trace

[a]0.15 mmol **S1**, 0.15 mmol **S2**, 0.1 mmol **S3**,5 mol% $Pd(PPh_3)_4$ and 0.15 mmol K_2HPO_4 in dry solvent (2 mL) irradiated at 10 w 455-460 nm lamp panel at room temperature for 30 h. [b]Yield determined by ^{1}H-NMR using 2,2,2-Trifluoro-N,N-dimethylacetamide as an external standard.

Table S15. Investigation of bases.

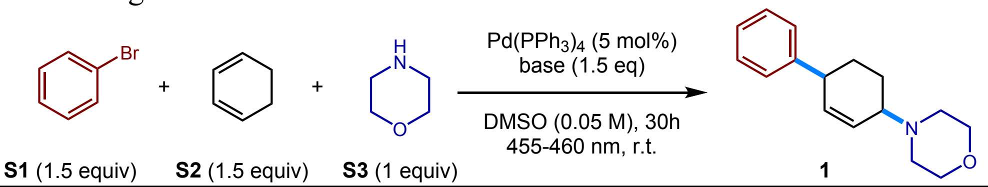

S1 (1.5 equiv) **S2** (1.5 equiv) **S3** (1 equiv) **1**

entry[a]	PC	base	T/°C	Time/h	**1**/yield/%[b]
1	$Pd(PPh_3)_4$	K_2HPO_4	25	30	33
2	$Pd(PPh_3)_4$	CH_3COONa	25	30	30
3	$Pd(PPh_3)_4$	NaH_2PO4	25	30	21
4	$Pd(PPh_3)_4$	Na_2CO_3	25	30	32

5	$Pd(PPh_3)_4$	$KHCO_3$	25	30	33
6	$Pd(PPh_3)_4$	K_2CO_3	25	30	34
7	$Pd(PPh_3)_4$	K_3PO_4	25	30	40
8	$Pd(PPh_3)_4$	$NaHCO_3$	25	30	36
9	$Pd(PPh_3)_4$	Na_2HPO_4	25	30	21
10	$Pd(PPh_3)_4$	Cs_2CO_3	25	30	24
1S3	$Pd(PPh_3)_4$	Without base	25	12	35

[a]0.15 mmol **S1**, 0.15 mmol **S2**, 0.1 mmol **S3**,5 mol% $Pd(PPh_3)_4$ and 0.15 mmol base in dry DMSO (2 mL) irradiated at 10 w 455-460 nm lamp panel at room temperature for 30 h. [b]Yield determined by ^{1}H-NMR using 2,2,2-Trifluoro-N,N-dimethylacetamide as an external standard. [c]Without base and used 10% $Pd(PPh_3)_4$, 0.45 mmol **S2.**

Table S16. Investigation of the temperature.

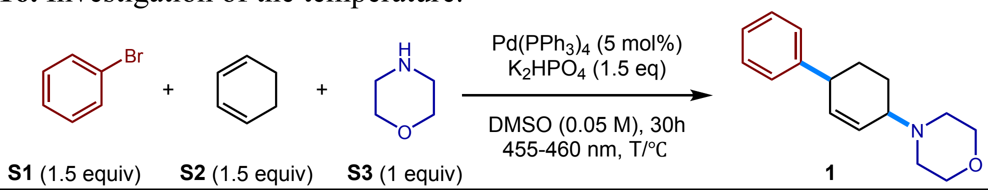

entry[a]	PC	T/°C	Time/h	**1**/yield/%[b]
1	$Pd(PPh_3)_4$	25	30	33
2	$Pd(PPh_3)_4$	40	30	34
3	$Pd(PPh_3)_4$	60	30	23
4	$Pd(PPh_3)_4$	80	30	12
5	$Pd(PPh_3)_4$	100	30	6

[a]0.15 mmol **S1**, 0.15 mmol **S2**, 0.1 mmol **S3**,5 mol% $Pd(PPh_3)_4$ and 0.15 mmol K_2HPO_4 in dry DMSO (2 mL) irradiated at 10 w 455-460 nm lamp panel at T °C for 30 h. [b]Yield determined by ^{1}H-NMR using 2,2,2-Trifluoro-N, N-dimethylacetamide as an external standard.

Table S17. Investigation of the equivalent of S1.

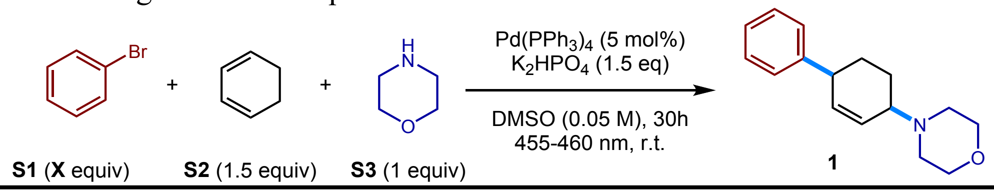

entry[a]	PC	X equiv	T/°C	Time/h	**1**/yield/%[b]
1	$Pd(PPh_3)_4$	1	25	30	32
2	$Pd(PPh_3)_4$	1.5	25	30	24
3	$Pd(PPh_3)_4$	2	25	30	22
4	$Pd(PPh_3)_4$	3	25	30	21
5	$Pd(PPh_3)_4$	4	25	30	22
6	$Pd(PPh_3)_4$	5	25	30	20

[a]0.1*X mmol **S1**, 0.15 mmol **S2**, 0.1 mmol **S3**,5 mol% $Pd(PPh_3)_4$ and 0.15 mmol K_2HPO_4 in dry DMSO (2 mL) irradiated at 10 w 455-460 nm lamp panel at room temperature for 30 h. [b]Yield determined by ^{1}H-NMR using 2,2,2-Trifluoro-N,N-dimethylacetamide as an external standard.

Table S18. Investigation of the equivalent of K_3PO_4.

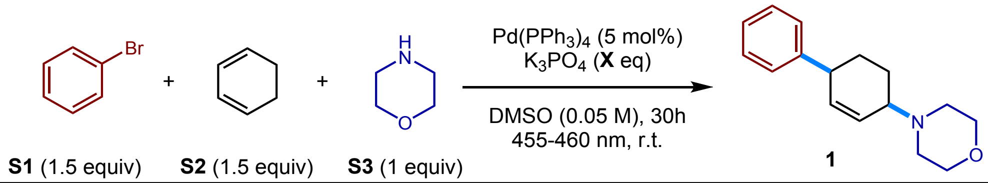

entry[a]	PC	**X** equiv	T/°C	Time/h	**1**/yield/%[b]
1	$Pd(PPh_3)_4$	1.0	25	30	37
2	$Pd(PPh_3)_4$	1.5	25	30	36
3	$Pd(PPh_3)_4$	2.0	25	30	36
4	$Pd(PPh_3)_4$	3.0	25	30	33
5	$Pd(PPh_3)_4$	4.0	25	30	31
6	$Pd(PPh_3)_4$	5.0	25	30	30

[a]0.15 mmol **S1**, 0.15 mmol **S2**, 0.1 mmol **S3**,5 mol% $Pd(PPh_3)_4$ and X mmol K_2HPO_4 in dry DMSO (2 mL) irradiated at 10 w 455-460 nm lamp panel at room temperature for 30 h. [b]Yield determined by ^{1}H-NMR using 2,2,2-Trifluoro-N,N-dimethylacetamide as an external standard.

Table S19. Investigation of the equivalent of S2.

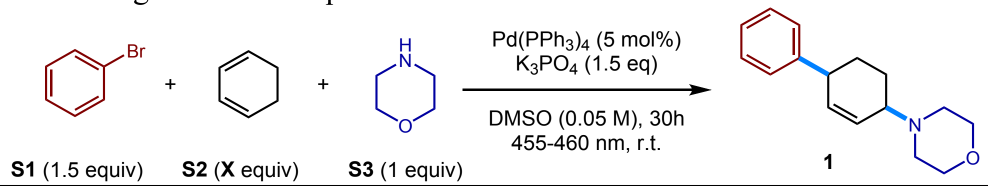

entry[a]	PC	**X** equiv	T/°C	Time/h	**1**/yield/%[b]
1	$Pd(PPh_3)_4$	1	25	30	14
2	$Pd(PPh_3)_4$	1.5	25	30	27
3	$Pd(PPh_3)_4$	2	25	30	42
4	$Pd(PPh_3)_4$	3	25	30	52
5	$Pd(PPh_3)_4$	4	25	30	60
6	$Pd(PPh_3)_4$	4.5	25	30	66
7	$Pd(PPh_3)_4$	5	25	30	66

[a]0.15 mmol **S1**, **X** mmol **S2**, 0.1 mmol **S3**,5 mol% $Pd(PPh_3)_4$ and 0.15 mmol K_3PO_4 in dry DMSO (2 mL) irradiated at 10 w 455-460 nm lamp panel at room temperature for 30 h. [b]Yield determined by ^{1}H-NMR using 2,2,2-Trifluoro-N,N-dimethylacetamide as an external standard.

Table S20. Investigation of the equivalent of S3.

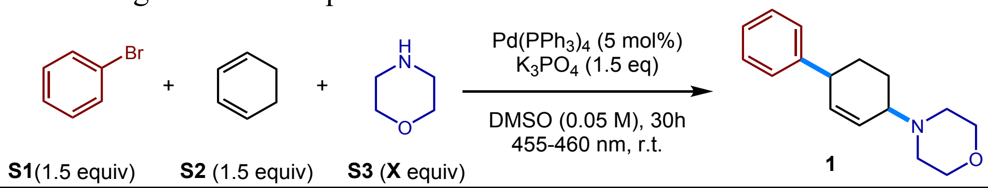

entry[a]	PC	**X** equiv	T/°C	Time/h	**1**/yield/%[b]
1	$Pd(PPh_3)_4$	1	25	30	36
2	$Pd(PPh_3)_4$	2	25	30	39
3	$Pd(PPh_3)_4$	3	25	30	40
4	$Pd(PPh_3)_4$	4	25	30	42
5	$Pd(PPh_3)_4$	5	25	30	35

[a]0.15 mmol **S1**, 0.15 mmol **S2**, 0.1***X** mmol **S3**,5 mol% $Pd(PPh_3)_4$ and 0.15 mmol K_3PO_4 in dry DMSO (2 mL) irradiated at 10 w 455-460 nm lamp panel at room temperature for 30 h.

[b]Yield determined by ^{1}H-NMR using 2,2,2-Trifluoro-N,N-dimethylacetamide as an external standard.

Table S21. Investigation of the time.

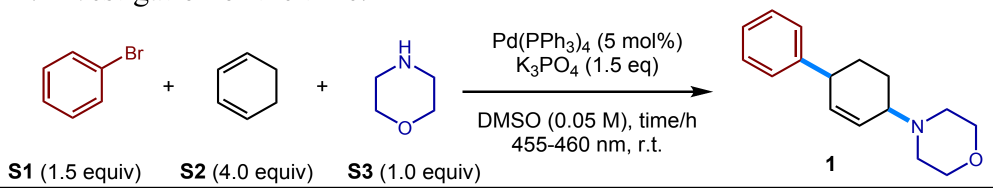

entry[a]	PC	T/°C	Time/h	**1**/yield/%[b]
1	$Pd(PPh_3)_4$	25	6	34
2	$Pd(PPh_3)_4$	25	12	52
3	$Pd(PPh_3)_4$	25	20	61
4	$Pd(PPh_3)_4$	25	25	57
5	$Pd(PPh_3)_4$	25	30	50

[a]0.15 mmol **S1**, 0.40 mmol **S2**, 0.1 mmol **S3**,5 mol% $Pd(PPh_3)_4$ and 0.15 mmol K_3PO_4 in dry DMSO (2 mL) irradiated at 10 w 455-460 nm lamp panel at room temperature for t h. [b]Yield determined by ^{1}H-NMR using 2,2,2-Trifluoro-N,N-dimethylacetamide as an external standard.

Table S22. Investigation of the equivalent of $Pd(PPh_3)_4$.

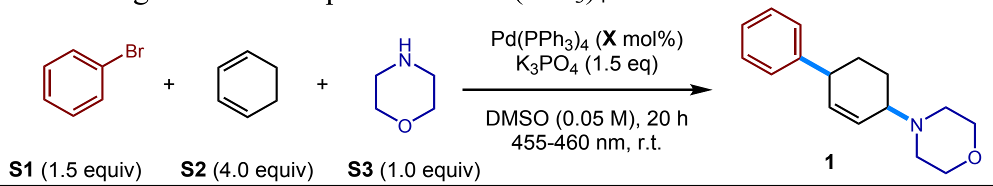

entry[a]	PC	**X** mol%	wavelength	T/°C	Time/h	**1**/yield/%[b]
1	$Pd(PPh_3)_4$	2.5	455-460 nm	25	20	30
2	$Pd(PPh_3)_4$	5	455-460 nm	25	20	65
3	$Pd(PPh_3)_4$	7.5	455-460 nm	25	20	64
4	$Pd(PPh_3)_4$	10	455-460 nm	25	20	69
5[c]	$Pd(PPh_3)_4$	10	Without light	25	20	0
6[d]	/	0	455-460 nm	25	20	0

[a]0.15 mmol **S1**, 0.40 mmol **S2**, 0.1 mmol **S3**, X mol% $Pd(PPh_3)_4$ and 0.15 mmol K_3PO_4 in dry DMSO (2 mL) irradiated at 10 w 455-460 nm lamp panel at room temperature for 20 h. [b]Yield determined by ^{1}H-NMR using 2,2,2-Trifluoro-N,N-dimethylacetamide as an external standard. [c]Without light source. [d]Without $Pd(PPh_3)_4$.

VI. Characterization of products

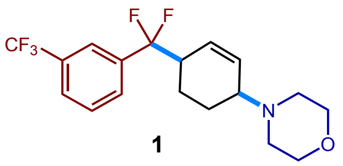

1

4-(4-(difluoro(3-(trifluoromethyl)phenyl)methyl)cyclohex-2-en-1-yl)morpholine

Prepared following general procedure A the reaction mixture was purified by column chromatography using 15% EtOAc in hexane to provide the title compound in 80% yield (29 mg) as a colorless oil.

R$_f$ = 0.45 (50% EtOAc in hexane)

^{1}H NMR (400 MHz, $CDCl_3$) δ 7.70 (d, *J* = 8.4 Hz, 2H), 7.65 (d, J = 8.0 Hz, 1H), 7.56 (t, *J* = 7.7 Hz, 1H), 5.94 (dt, *J* = 10.5, 2.6 Hz, 1H), 5.83 (dt, *J* = 10.5, 2.7 Hz, 1H), 3.64 (t, *J* = 4.6 Hz, 4H), 3.05 (s, 1H), 2.93 – 2.75 (m, 1H), 2.56 – 2.45 (m, 2H), 2.43 – 2.31 (m, 2H), 1.86 – 1.74 (m, 1H), 1.71 – 1.58 (m, 1H), 1.57 – 1.42 (m, 2H). **^{13}C NMR** (101 MHz, $CDCl_3$) δ 137.04 (t, *J* = 27.4 Hz), 133.83 – 133.29 (m), 130.79 (q, *J* = 32.9 Hz), 129.25 – 128.92 (m), 128.89, 126.94 – 126.34 (m), 125.85 – 125.47 (m), 123.72 (q, *J* = 272.4 Hz), 123.05 – 122.57 (m), 122.69 (t, *J* = 248.4 Hz), 67.26, 58.62, 49.27, 42.70 (t, *J* = 26.0 Hz), 20.33 (t, *J* = 3.4 Hz), 19.72. **^{19}F NMR** (377 MHz, $CDCl_3$) δ -62.74, -98.53 (dd, *J* = 245.8, 14.0 Hz), -100.53 (dd, *J* = 245.7, 14.4 Hz).

HRMS (ESI) for $C_{18}H_{20}F_5NO$ $[M+H]^+$ m/z: calcd. 362.1538, found 362.1540.

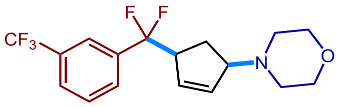

2

4-(4-(difluoro(3-(trifluoromethyl)phenyl)methyl)cyclopent-2-en-1-yl)morpholine

Prepared following general procedure A the reaction mixture was purified by column chromatography using 15% EtOAc in hexane to provide the title compound in 63% yield (22 mg) as a yellow oil.

R_f = 0.5 (50% EtOAc in hexane)

^{1}H NMR (600 MHz, $CDCl_3$) δ 7.73 – 7.65 (m, 2H), 7.64 (d, *J* = 7.9 Hz, 1H), 7.58 – 7.52 (m, 1H), 6.01 – 5.94 (m, 1H), 5.78 – 5.71 (m, 1H), 3.83 – 3.73 (m, 1H), 3.70 – 3.55 (m, 4H), 3.44 – 3.30 (m, 1H), 2.46 – 2.30 (m, 4H), 2.09 – 1.98 (m, 1H), 1.77 – 1.66 (m, 1H). **^{13}C NMR** (101 MHz, $CDCl_3$) δ 137.26 (t, *J* = 27.5 Hz), 135.36, 130.93 (q, *J* = 32.8 Hz), 129.00, 128.98 – 128.73 (m), 126.95 – 126.21 (m), 123.64 (q, *J* = 272.4 Hz), 122.74 – 122.34 (m), 121.85 (t, *J* = 245.0 Hz), 70.80, 66.96, 52.68 (t, *J* = 27.8 Hz), 49.75, 24.70 (t, *J* = 3.2 Hz). **^{19}F NMR** (377 MHz, $CDCl_3$) δ -62.80, -99.89 (dd, *J* = 246.9, 14.2 Hz), -100.79 (dd, *J* = 246.8, 14.6 Hz).

HRMS (ESI) for $C_{17}H_{18}F_5NO$ $[M+H]^+$ m/z: calcd. 348.1381, found 348.1382.

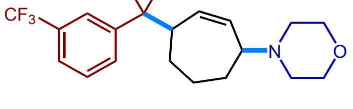

3

4-(4-(difluoro(3-(trifluoromethyl)phenyl)methyl)cyclohept-2-en-1-yl)morpholine

Prepared following general procedure A the reaction mixture was purified by column chromatography using 15% EtOAc in hexane to provide the title compound in 63% yield (24 mg) as a colorless oil.

R_f = 0.5 (50% EtOAc in hexane)

^{1}H NMR (600 MHz, $CDCl_3$) δ 7.73 (s, 1H), 7.70 (d, *J* = 7.9 Hz, 1H), 7.66 (d, *J* = 7.9 Hz, 1H), 7.56 (t, *J* = 7.8 Hz, 1H), 6.04 – 5.91 (m, 1H), 5.88 – 5.73 (m, 1H), 3.86 – 3.56 (m, 4H), 3.28 – 3.18 (m, 1H), 3.10 – 2.91 (m, 1H), 2.65 – 2.48 (m, 4H), 2.18 – 2.06 (m, 1H), 1.92 – 1.78 (m, 2H), 1.66 – 1.50 (m, 1H), 1.38 – 1.29 (m, 1H), 1.27 – 1.20 (m, 1H). **^{13}C NMR** (101 MHz, $CDCl_3$) δ 137.27 (t, *J* = 27.4 Hz), 134.98, 131.67 – 130.31 (m), 129.11, 129.03, 127.39, 126.66 (d, *J* = 4.2 Hz), 123.68 (q, *J* = 273.7 Hz), 122.67 (t, *J* = 246.6 Hz), 122.65 – 122.41 (m), 67.11, 64.85, 48.95, 47.98 (t, *J* = 25.2 Hz), 28.76, 28.34, 26.14 (t, *J* = 3.7 Hz). **^{19}F NMR** (377 MHz, $CDCl_3$) δ -62.71, -101.17 (dd, *J* = 249.9, 15.3 Hz), -102.36 (dd, *J* = 250.1, 16.0 Hz).
HRMS (ESI) for $C_{19}H_{22}F_5NO$ $[M+H]^+$ m/z: calcd. 376.1694, found 376.1700.

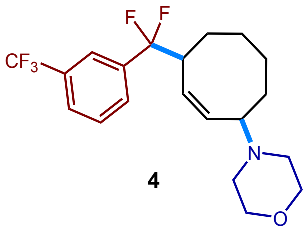

4

4-(difluoro(3-(trifluoromethyl)phenyl)methyl)cyclooct-2-en-1-yl)morpholine
Prepared following general procedure A the reaction mixture was purified by column chromatography using 15% EtOAc in hexane to provide the title compound in 44% yield (17 mg) as a yellow oil.
R$_f$ = 0.5 (50% EtOAc in hexane)
^{1}H NMR (400 MHz, CDCl3) δ 7.73 – 7.62 (m, 3H), 7.59 – 7.49 (m, 1H), 5.61 – 5.47 (m, 2H), 3.74 – 3.60 (m, 4H), 3.29 – 3.10 (m, 1H), 3.10 – 2.95 (m, 1H), 2.53 – 2.35 (m, 4H), 1.96 – 1.83 (m, 2H), 1.80 – 1.64 (m, 1H), 1.62 – 1.49 (m, 2H), 1.49 – 1.36 (m, 2H), 1.34 – 1.25 (m, 1H). **^{13}C NMR** (101 MHz, $CDCl_3$) δ 137.70 (t, *J* = 27.5 Hz), 133.35, 130.88 (q, *J* = 32.7 Hz), 128.99, 128.84 (t, *J* = 6.2 Hz), 126.83 – 126.18 (m), 123.74 (q, *J* = 272.1 Hz), 122.96 – 122.11 (m), 122.41 (d, *J* = 246.4 Hz), 67.05, 62.85, 51.32, 46.12 (t, *J* = 25.2 Hz), 32.38, 28.81 (t, *J* = 3.3 Hz), 25.58, 24.63. **^{19}F NMR** (377 MHz, $CDCl_3$) δ -62.74, -98.15 (dd, *J* = 249.4, 11.7 Hz), -105.88 (dd, *J* = 249.5, 17.9 Hz).
HRMS (ESI) for $C_{20}H_{24}F_5NO$ $[M+H]^+$ m/z: calcd. 390.1851, found 390.1852.

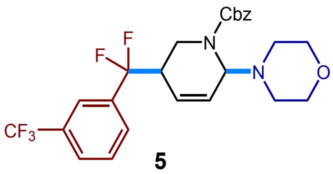

5

Benzyl-3-(difluoro(3-(trifluoromethyl)phenyl)methyl)-6-morpholino-3,6-di-hydropyridine-1(2H)-carboxylate
Prepared following general procedure A the reaction mixture was purified by column

chromatography using 15% EtOAc in hexane to provide the title compound in 55% yield (27.5 mg) as a yellow oil.

R$_f$ = 0.3 (25% EtOAc in hexane)

^{1}H NMR (600 MHz, CD_3CN) δ 7.82 – 7.76 (m, 2H), 7.72 (d, *J* = 7.9 Hz, 1H), 7.64 (t, *J* = 7.9 Hz, 1H), 7.36 – 7.19 (m, 5H), 6.02 (d, *J* = 10.5 Hz, 1H), 5.84 (d, *J* = 10.4 Hz, 1H), 5.11 – 4.94 (m, 3H), 4.07 (s, 1H), 3.44 (s, 4H), 3.19 – 3.08 (m, 1H), 2.94 (s, 1H), 2.58 – 2.31 (m, 4H).**^{13}C NMR** (151 MHz, CD_3CN) δ 156.64, 137.77, 136.88 (t, *J* = 27.1 Hz), 131.37 (q, *J* = 32.6 Hz), 130.82, 130.58, 130.45 – 130.24 (m), 129.43, 128.95, 128.79 – 128.45 (m), 128.25 (d, *J* = 4.0 Hz), 126.54 (t, *J* = 4.5 Hz), 123.44 – 123.03 (m), 124.92 (q, *J* = 271.8 Hz), 122.22 (t, *J* = 245.5 Hz), 70.53, 67.99, 67.69, 50.35, 43.88 – 42.49 (m), 40.76 – 39.06 (m). **^{19}F NMR** (377 MHz, CD_3CN) δ -63.20, -99.33 – -104.41 (m).

HRMS (ESI) for $C_{25}H_{25}F_5N_2O_3$ [M-$C_4H_8NO^-$]$^+$ m/z: calcd. 410.1174, found 410.1170.

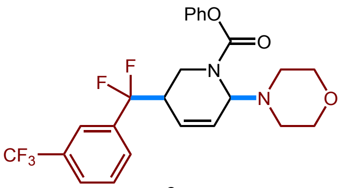

6

phenyl-3-(difluoro(3-(trifluoromethyl)phenyl)methyl)-6-morpholino-3,6-dihydropyridine-1(2H)-carboxylate

Prepared following general procedure A the reaction mixture was purified by column chromatography using 15% EtOAc in hexane to provide the title compound in 45% yield (22 mg) as a yellow oil.

R$_f$ = 0.3 (25% EtOAc in hexane)

^{1}H NMR (400 MHz, CD_3CN) δ 7.88 – 7.73 (m, 3H), 7.72 – 7.61 (m, 1H), 7.42 – 7.28 (m, 2H), 7.23 – 7.14 (m, 1H), 7.11 – 6.99 (m, 2H), 6.15 – 6.02 (m, 1H), 5.96 – 5.85 (m, 1H), 5.13 (s, 1H), 4.31 – 4.11 (m, 1H), 3.65 – 3.40 (m, 4H), 3.39 – 3.20 (m, 1H), 3.16 – 2.94 (m, 1H), 2.83 – 2.36 (m, 4H). **^{13}C NMR** (151 MHz, CD_3CN) δ 154.35, 151.37, 135.84 (t, *J* = 27.0 Hz), 130.44 (q, *J* = 32.7 Hz), 129.90, 129.57 – 129.38 (m), 129.29, 127.54 – 127.20 (m), 125.83 – 125.61 (m), 125.46, 123.96 (q, *J* = 271.7 Hz), 122.61 – 122.25 (m), 121.82, 121.75, 121.73 (t, *J* = 245.8 Hz), 69.97, 66.78, 49.38, 42.83 – 41.55 (m), 40.46 – 37.64 (m). **^{19}F NMR** (377 MHz, CD_3CN) δ -63.23, -101.57.

HRMS (ESI) for $C_{24}H_{23}F_5N_2O_3$ [M-$C_4H_8NO^-$]$^+$ m/z: calcd. 396.1017, found 396.1010.

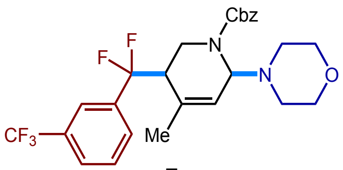

7

benzyl-3-(difluoro(3-(trifluoromethyl)phenyl)methyl)-4-methyl-6-morphol--ino-3,6-dihydropyridine-1(2H)-carboxylate

Prepared following general procedure A the reaction mixture was purified by column chromatography using 10% EtOAc in hexane to provide the title compound in 50% yield (26 mg) as a yellow oil.

R_f = 0.5 (25% EtOAc in hexane)

^{1}H NMR (400 MHz, CD_3CN) δ 7.88 – 7.81 (m, 2H), 7.80 – 7.74 (m, 1H), 7.71 – 7.63 (m, 1H), 7.37 – 7.30 (m, 3H), 7.28 – 7.23 (m, 2H), 5.76 – 5.66 (m, 1H), 5.10 – 4.96 (m, 2H), 4.92 – 4.83 (m, 1H), 3.76 (dd, J = 13.4, 5.5 Hz, 1H), 3.47 (t, J = 4.7 Hz, 4H), 3.29 – 3.12 (m, 1H), 3.02 (dd, J = 13.3, 11.6 Hz, 1H), 2.59 – 2.47 (m, 2H), 2.46 – 2.37 (m, 2H), 2.27 (s, 3H). **^{13}C NMR** (101 MHz, CD_3CN) δ 156.27, 138.16 – 137.35 (m), 135.86 – 135.28 (m), 131.42 (q, J = 32.6 Hz), 130.92, 130.26 (t, J = 6.4 Hz), 129.45, 128.96, 128.69, 128.34 – 128.04 (m), 127.13, 124.96 (q, J = 271.8 Hz), 123.31 (t, J = 248.7 Hz), 123.24 – 122.86 (m), 70.96, 67.92, 67.68, 50.47, 45.63 (t, J = 24.0 Hz), 41.17 – 40.40 (m), 23.71 – 22.02 (m). **^{19}F NMR** (377 MHz, CD_3CN) δ -63.12, -87.63 (d, J = 252.9 Hz), -104.18 (d, J = 253.4 Hz).

HRMS (ESI) for $C_{26}H_{27}F_5N_2O_3$ $[M+H]^+$ m/z: calcd. 511.2015, found 511.2018.

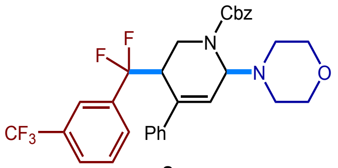

8

benzyl-3-(difluoro(3-(trifluoromethyl)phenyl)methyl)- 6-morpholino-4-phenyl-3,6-dihydropyridine-1(2H)-carboxylate

Prepared following general procedure A the reaction mixture was purified by column chromatography using 15% EtOAc in hexane to provide the title compound in 62% yield (35.5 mg) as a yellow oil.

R_f = 0.3 (25% EtOAc in hexane)

^{1}H NMR (400 MHz, $CDCl_3$) δ 7.60 – 7.51 (m, 1H), 7.41 – 7.32 (m, 7H), 7.35 – 7.29 (m, 1H), 7.19 – 7.08 (m, 3H), 7.04 – 6.95 (m, 2H), 5.86 (dd, J = 5.1, 1.8 Hz, 1H), 5.27 – 5.11 (m, 2H), 5.10 – 5.00 (m, 1H), 4.54 (ddd, J = 13.8, 6.6, 1.3 Hz, 1H), 3.93 – 3.77 (m, 1H), 3.58 (dd, J = 5.6, 3.8 Hz, 4H), 3.53 – 3.44 (m, 1H), 2.63 (dt, J = 11.5, 4.7 Hz, 2H), 2.52 (dt, J = 11.2, 4.7

Hz, 2H). **^{13}C NMR** (101 MHz, $CDCl_3$) δ 155.48, 140.05, 139.14 – 138.82 (m), 137.08 – 136.24 (m), 136.18, 130.48 (q, J = 32.8 Hz), 130.09, 129.08 – 128.70 (m), 128.57, 128.55, 128.24, 128.01, 127.82, 127.47, 127.00, 126.35, 123.52 (q, J = 272.6 Hz), 122.74 – 122.38 (m), 121.71 (t, J = 249.4 Hz), 69.69, 67.58, 67.06, 49.73, 44.36 (t, J = 25.4 Hz), 38.70 – 38.31 (m). **^{19}F NMR** (565 MHz, $CDCl_3$) δ -62.64, -87.79 (d, J = 254.6 Hz), -101.49 (d, J = 254.5 Hz).

HRMS (ESI) for $C_{31}H_{29}F_5N_2O_3$ [M-$C_4H_8NO^-$]$^+$ m/z: calcd. 486.1487, found 486.1489.

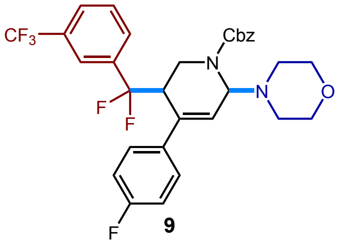

9

benzyl-3-(difluoro(3-(trifluoromethyl)phenyl)methyl)- 4-(4-fluorophenyl)-6-morpholino-3,6-dihydropyridine-1(2H)-carboxylate

Prepared following general procedure A the reaction mixture was purified by column chromatography using 10% EtOAc in hexane to provide the title compound in 50% yield (30 mg) as a yellow oil.

R$_f$ = 0.7 (25% EtOAc in hexane)

^{1}H NMR (400 MHz, CD_3CN) δ 7.75 – 7.64 (m, 1H), 7.51 (dd, J = 4.7, 1.9 Hz, 2H), 7.41 – 7.29 (m, 6H), 7.15 – 7.04 (m, 2H), 6.92 – 6.79 (m, 2H), 5.87 (dd, J = 5.1, 1.8 Hz, 1H), 5.20 – 5.06 (m, 2H), 4.99 (dd, J = 4.8, 1.7 Hz, 1H), 4.37 (dd, J = 13.8, 6.6 Hz, 1H), 4.11 – 3.93 (m, 1H), 3.50 (dq, J = 5.9, 2.7 Hz, 5H), 2.66 – 2.53 (m, 2H), 2.47 (dt, J = 11.4, 4.6 Hz, 2H). **^{13}C NMR** (151 MHz, CD_3CN) δ 162.87 (d, J = 244.4 Hz), 156.20, 138.76 (t, J = 3.3 Hz), 137.87, 137.79 (d, J = 3.3 Hz), 137.62, 137.45, 131.47, 130.84 (q, J = 32.5 Hz), 130.38, 130.10 (d, J = 8.1 Hz), 129.49, 129.02, 128.80, 127.66, 124.86 (q, J = 271.8 Hz), 123.31 – 123.02 (m), 123.18 (t, J = 248.0 Hz), 115.47 (d, J = 21.7 Hz), 70.50, 68.03, 67.63, 50.69, 44.96 (t, J = 25.0 Hz), 39.49 – 38.91 (m). **^{19}F NMR** (565 MHz, CD_3CN) δ -63.19, -89.91 (d, J = 252.4 Hz), -100.69 (d, J = 252.2 Hz), -116.69 (tt, J = 9.4, 5.4 Hz).

HRMS (ESI) for $C_{31}H_{28}F_6N_2O_3$ [M-$C_4H_8NO^-$]$^+$ m/z: calcd. 504.1393, found 504.1393.

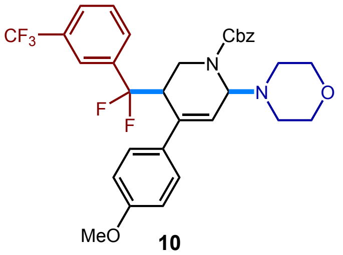

10

benzyl-3-(difluoro(3-(trifluoromethyl)phenyl)methyl)-4-(4-methoxyphenyl)-6-morpholino-3,6-dihydropyridine-1(2H)-carboxylate

Prepared following general procedure A the reaction mixture was purified by column chromatography using 10% EtOAc in hexane to provide the title compound in 50% yield (30 mg) as a yellow oil.

R$_f$ = 0.5 (25% EtOAc in hexane)

^{1}H NMR (600 MHz, CD_3CN) δ 7.69 – 7.64 (m, 1H), 7.54 – 7.47 (m, 2H), 7.43 – 7.36 (m, 5H), 7.35 – 7.32 (m, 1H), 7.05 – 6.95 (m, 2H), 6.73 – 6.63 (m, 2H), 5.82 (dd, J = 5.2, 1.7 Hz, 1H), 5.18 – 5.09 (m, 2H), 4.95 (dt, J = 5.1, 1.5 Hz, 1H), 4.45 – 4.35 (m, 1H), 4.09 – 3.95 (m, 1H), 3.80 – 3.72 (m, 1H), 3.72 (s, 3H), 3.51 – 3.45 (m, 4H), 2.59 – 2.51 (m, 2H), 2.46 – 2.38 (m, 2H). **^{13}C NMR** (151 MHz, CD_3CN) δ 159.92, 156.15, 139.12 (t, J = 3.4 Hz), 137.92, 137.63 (t, J = 26.7 Hz), 133.70, 130.73 (q, J = 32.5 Hz), 130.47 (t, J = 6.4 Hz), 130.25, 129.48, 129.31, 129.00, 128.80, 128.43, 127.55, 124.91 (q, J = 271.7 Hz), 123.41 – 123.04 (m), 123.38 (t, J = 248.4 Hz), 114.08, 70.52, 67.98, 67.61, 55.75, 50.69, 44.83 (t, J = 25.0 Hz), 39.01 (dd, J = 8.6, 5.2 Hz). **^{19}F NMR** (565 MHz, CD_3CN) δ -63.12, -88.22 (d, J = 252.9 Hz), -101.05 (d, J = 252.8 Hz).

HRMS (ESI) for $C_{32}H_{31}F_5N_2O_4$ [M-$C_4H_8NO^-$]$^+$ m/z: calcd. 516.1593, found 516.1597.

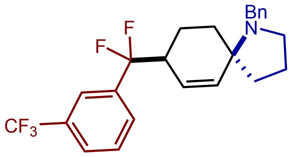

11

1-benzyl-8-(difluoro(3-(trifluoromethyl)phenyl)methyl)-1-azaspiro[4.5]dec-6-ene

Prepared following general procedure B the reaction mixture was purified by column chromatography using 5% EtOAc in hexane to provide the title compound in 48% yield (20.5 mg) as a yellow oil.

R$_f$ = 0.8 (30% EtOAc in hexane)

^{1}H NMR (600 MHz, $CDCl_3$) δ 7.84 – 7.77 (m, 1H), 7.74 – 7.68 (m, 2H), 7.56 (t, J = 7.8 Hz, 1H), 7.37 – 7.25 (m, 5H), 5.97 – 5.90 (m, 1H), 5.86 – 5.79 (m, 1H), 3.56 – 3.47 (m, 1H), 3.33 – 3.25 (m, 1H), 2.96 – 2.84 (m, 1H), 2.80 – 2.73 (m, 1H), 2.66 – 2.58 (m, 1H), 2.01 – 1.92 (m, 1H), 1.89 – 1.77 (m, 5H), 1.75 – 1.66 (m, 1H), 1.43 – 1.37 (m, 1H). **^{13}C NMR** (101 MHz, $CDCl_3$) δ 140.75, 138.93, 137.24 (t, J = 27.6 Hz), 130.75 (q, J = 32.8 Hz), 129.15 (t, J = 6.1 Hz), 128.79, 128.23, 128.05, 126.88 – 125.90 (m), 123.72 (t, J = 5.0 Hz), 123.70 (q, J = 272.4 Hz), 122.84 (t, J = 247.1 Hz), 122.79 – 122.50 (m), 62.09, 53.68, 50.39, 42.35 (t, J = 26.0 Hz), 38.44, 26.06, 21.40, 20.43 (t, J = 3.3 Hz). **^{19}F NMR** (377 MHz, $CDCl_3$) δ -62.64, -97.99 (dd, J = 245.1, 13.9 Hz), -100.23 (dd, J = 245.2, 15.1 Hz).

HRMS (ESI) for $C_{24}H_{24}F_5N$ [M+H]$^+$ m/z: calcd. 422.1902, found 422.1907.

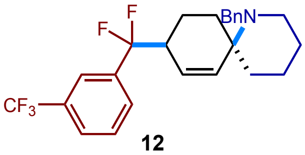

12

1-benzyl-9-(difluoro(3-(trifluoromethyl)phenyl)methyl)-1-azaspiro[5.5]undec-7-ene

Prepared following general procedure A the reaction mixture was purified by column chromatography using 1% EtOAc in hexane to provide the title compound in 42% yield (18.5 mg) as a yellow oil.

R$_f$ = 0.3 (10% EtOAc in hexane)

^{1}H NMR (400 MHz, $CDCl_3$) δ 7.75 (s, 1H), 7.71 (d, J = 7.9 Hz, 1H), 7.66 (d, J = 7.9 Hz, 1H), 7.55 (t, J = 7.8 Hz, 1H), 7.35 – 7.27 (m, 4H), 7.25 – 7.19 (m, 1H), 5.88 – 5.82 (m, 1H), 5.75 – 5.68 (m, 1H), 3.73 (d, J = 14.0 Hz, 1H), 2.92 (d, J = 14.0 Hz, 1H), 2.89 – 2.75 (m, 1H), 2.55 – 2.39 (m, 1H), 2.32 – 2.21 (m, 1H), 1.93 – 1.72 (m, 3H), 1.63 – 1.39 (m, 7H). **^{13}C NMR** (101 MHz, $CDCl_3$) δ 142.20, 141.25, 137.35 (t, J = 27.5 Hz), 130.85 (q, J = 32.8 Hz), 129.17 (t, J = 6.2 Hz), 128.85, 128.14, 127.98, 126.50, 126.38, 123.72 (q, J = 272.5 Hz), 123.04 – 122.73 (m), 122.89 (t, J = 247.1 Hz), 122.73 – 122.42 (m), 55.71, 55.55, 45.54, 42.53 (t, J = 25.9 Hz), 36.93, 26.12, 20.22, 20.14, 20.00 (t, J = 3.3 Hz). **^{19}F NMR** (377 MHz, $CDCl_3$) δ -62.66, -98.50 (dd, J = 245.1, 14.2 Hz), -100.54 (dd, J = 245.2, 15.3 Hz).

HRMS (ESI) for $C_{25}H_{26}F_5N$ $[M+H]^+$ m/z: calcd. 436.2058, found 436.2060.

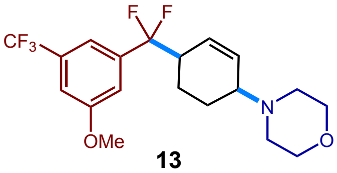

13

4-4-(difluoro(3-methoxy-5-(trifluoromethyl)phenyl)methyl)cyclohex-2-en-1-yl)morpholine

Prepared following general procedure A the reaction mixture was purified by column chromatography using 35% EtOAc in hexane to provide the title compound in 72% yield (28.5 mg) as a yellow oil.

R$_f$ =0.25 (35% EtOAc in hexane).

^{1}H NMR (600 MHz, $CDCl_3$) δ 7.26 (s, 1H), 7.16 (d, J = 11.7 Hz, 2H), 5.93 (dt, J = 10.5, 2.7 Hz, 1H), 5.82 (dt, J = 10.5, 2.8 Hz, 1H), 3.87 (s, 3H), 3.65 (t, J = 4.6 Hz, 4H), 3.05 (s, 1H), 2.80 (t, 1H), 2.54 – 2.46 (m, 2H), 2.42 – 2.35 (m, 2H), 1.79 (dp, J = 13.9, 4.4 Hz, 1H), 1.64 (dtd, J = 14.1, 10.1, 8.4, 5.3 Hz, 1H), 1.53 – 1.43 (m, 2H). **^{13}C NMR** (151 MHz, $CDCl_3$) δ 159.76, 138.58 (t, J = 27.6 Hz), 133.59, 132.04 (q, J = 32.9 Hz), 125.54, 123.55 (q, J = 272.7 Hz), 122.53 (t, J = 247.2 Hz), 115.35 (q, J = 6.3 Hz), 114.96 – 114.74 (m), 111.69, 67.34,

58.63, 55.76, 49.34, 42.67 (t, J = 25.9 Hz), 20.36 (t, J = 3.3 Hz), 19.80. ^{19}**F NMR** (565 MHz, $CDCl_3$) δ -62.75, -98.59 (dd, J = 245.0, 14.2 Hz), -100.40 (dd, J = 244.5, 14.8 Hz).

HRMS (ESI) for $C_{19}H_{22}F_5NO_2$ $[M+H]^+$ m/z: calcd. 392.1644, found 392.1647.

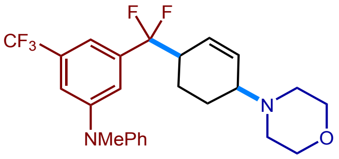

14

3-(difluoro(1R,4S)-4-morpholinocyclohex-2-en-1-yl)methyl)-N-methyl-N-phenyl-5-(trifluoromethyl)aniline

Prepared following general procedure A the reaction mixture was purified by column chromatography using 30% EtOAc in hexane to provide the title compound in 78% yield (36.5 mg) as a yellow oil.

$\mathbf{R_f}$=0.25 (30% EtOAc in hexane).

^{1}**H NMR** (600 MHz, $CDCl_3$) δ 7.39 (t, J = 7.9 Hz, 2H), 7.18 (t, J = 7.4 Hz, 1H), 7.15 (d, J = 7.2 Hz, 2H), 7.08 (d, J = 6.0 Hz, 2H), 7.04 (s, 1H), 5.92 (d, J = 9.9 Hz, 1H), 5.81 (dt, J = 10.6, 2.7 Hz, 1H), 3.67 (s, 4H), 3.36 (s, 3H), 3.08 (s, 1H), 2.82 – 2.71 (m, 1H), 2.56 – 2.49 (m, 2H), 2.47 – 2.38 (m, 2H), 1.78 (ddt, J = 12.8, 8.0, 4.1 Hz, 1H), 1.68 – 1.60 (m, 1H), 1.59 – 1.53 (m, 1H), 1.52 – 1.45 (m, 1H).^{13}**C NMR** (151 MHz, $CDCl_3$) δ 149.34, 147.48, 137.71 (t, J = 27.2 Hz), 133.08, 131.52 (q, J = 32.3 Hz), 129.92, 125.94, 124.93, 123.86 (q, J = 272.7 Hz), 124.54, 122.81 (t, J = 246.6 Hz), 116.09 (t, J = 5.5 Hz)), 113.34, 112.40, 67.27, 58.72, 49.29, 42.60 (t, J = 26.2 Hz), 40.41, 20.41, 19.79. ^{19}**F NMR** (565 MHz, $CDCl_3$) δ -62.82, -98.87 (dd, J = 243.2, 14.2 Hz), -100.56 (dd, J = 243.5, 14.8 Hz).

HRMS (ESI) for $C_{25}H_{27}F_5N_2O$ $[M+H]^+$ m/z: calcd. 467.2117, found 467.2122.

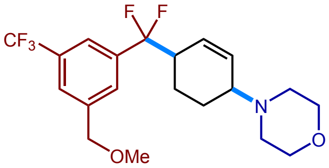

15

4-(4-(difluoro(3-(methoxymethyl)-5-(trifluoromethyl)phenyl)methyl)cyclohex-2-en-1-yl)morpholine

Prepared following general procedure A the reaction mixture was purified by column chromatography using 15% EtOAc in hexane to provide the title compound in 43% yield (17.5 mg) as a yellow oil.

$\mathbf{R_f}$=0.25 (30% EtOAc in hexane).

^{1}**H NMR** (400 MHz, $CDCl_3$) δ 7.67 (s, 1H), 7.60 (s, 2H), 5.95 (dt, J = 10.4, 2.6 Hz, 1H), 5.84 (dt, J = 10.4, 2.6 Hz, 1H), 4.53 (s, 2H), 3.66 (t, J = 4.6 Hz, 4H), 3.44 (s, 3H), 3.08 (s, 1H), 2.92

– 2.75 (m, 1H), 2.59 – 2.47 (m, 2H), 2.46 – 2.34 (m, 2H), 1.86 – 1.74 (m, 1H), 1.64 (tdd, *J* = 13.5, 6.4, 4.3 Hz, 1H), 1.58 – 1.43 (m, 2H). **^{13}C NMR** (151 MHz, $CDCl_3$) δ 140.03, 137.27 (t, *J* = 27.6 Hz), 133.15, 130.97 (q, *J* = 32.9 Hz), 127.71 (t, *J* = 6.2 Hz), 125.75, 125.39, 123.68 (d, *J* = 272.6 Hz), 122.63 (t, *J* = 247.0 Hz), 121.87, 73.45, 67.15, 58.68, 58.62, 49.27, 42.64 (t, *J* = 26.1 Hz), 20.30 (t, *J* = 3.4 Hz), 19.76. **^{19}F NMR** (565 MHz, $CDCl_3$) δ -62.64, -98.65 (d, *J* = 246.2 Hz), -100.29 (d, *J* = 245.8 Hz).
HRMS (ESI) for $C_{20}H_{24}F_5NO_2$ $[M+H]^+$ m/z: calcd. 406.1800, found 406.1802.

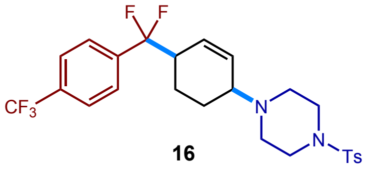

16

1-(4-(difluoro(4-(trifluoromethyl)phenyl)methyl)cyclohex-2-en-1-yl)-4-tosylpiperazine
Prepared following general procedure A the reaction mixture was purified by column chromatography using 20% EtOAc in hexane to provide the title compound in 47% yield (24.5 mg) as a yellow oil.
R_f =0.25 (35% EtOAc in hexane).
^{1}H NMR (600 MHz, $CDCl_3$) δ 7.67 (d, *J* = 8.1 Hz, 2H), 7.62 (d, *J* = 8.2 Hz, 2H), 7.55 (d, *J* = 8.1 Hz, 2H), 7.31 (d, *J* = 8.0 Hz, 2H), 5.83 – 5.74 (m, 2H), 3.06 (s, 1H), 2.94 (s, 4H), 2.81 – 2.72 (m, 1H), 2.63 – 2.56 (m, 2H), 2.51 – 2.45 (m, 2H), 2.42 (s, 3H), 1.76 – 1.67 (m, 1H), 1.63 – 1.54 (m, 2H), 1.49 – 1.40 (m, 1H). **^{13}C NMR** (101 MHz, $CDCl_3$) δ 143.64, 139.72 (t, *J* = 27.0 Hz), 133.11, 132.35, 131.98 (q, *J* = 33.2 Hz), 129.63, 127.87, 126.19 (t, *J* = 6.3 Hz), 125.97 – 125.67 (m), 125.39 – 125.22 (m), 123.68 (q, *J* = 272.4 Hz), 122.68 (t, *J* = 246.8 Hz), 58.23, 48.16, 46.42, 42.62 (t, *J* = 25.9 Hz), 21.52, 20.34 (t, *J* = 3.3 Hz), 20.18. **^{19}F NMR** (377 MHz, $CDCl_3$) δ -62.81, -100.07 (dd, *J* = 245.6, 15.1 Hz), -100.91 (dd, *J* = 245.6, 14.7 Hz).
HRMS (ESI) for $C_{25}H_{27}F_5N_2O_2S$ $[M+H]^+$ m/z: calcd. 515.1787, found 515.1784.

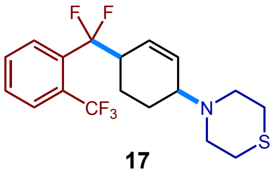

17

4-(4-(difluoro(2-(trifluoromethyl)phenyl)methyl)cyclohex-2-en-1-yl)thiomorpholine
Prepared following general procedure A the reaction mixture was purified by column chromatography using 15% EtOAc in hexane to provide the title compound in 67% yield (25.5 mg) as a white solid.
R_f =0.25 (30% EtOAc in hexane).
^{1}H NMR (600 MHz, $CDCl_3$) δ 7.81 (d, *J* = 7.9 Hz, 1H), 7.59 (ddt, *J* = 23.3, 15.1, 7.1 Hz, 3H),

5.87 (dt, J = 10.5, 2.5 Hz, 1H), 5.70 (dt, J = 10.4, 2.9 Hz, 1H), 3.16 (ddt, J = 8.5, 5.5, 2.7 Hz, 1H), 2.94 (ddd, J = 10.9, 7.1, 2.9 Hz, 2H), 2.90 – 2.84 (m, 1H), 2.80 (ddd, J = 11.2, 7.2, 2.8 Hz, 2H), 2.74 – 2.63 (m, 4H), 1.96 – 1.86 (m, 2H), 1.61 – 1.54 (m, 2H). **^{13}C NMR** (101 MHz, $CDCl_3$) δ 134.77 (t, J = 26.3 Hz), 133.97, 131.44, 129.99, 129.12 (t, J = 9.4 Hz), 127.72 (q, J = 6.6 Hz), 127.28, 125.83 – 125.36 (m), 123.56 (q, J = 273.5 Hz), 122.86 (t, J = 249.0 Hz), 60.59, 51.38, 42.19 – 41.33 (m), 28.63, 20.78 (t, J = 3.0 Hz), 19.57. **^{19}F NMR** (377 MHz, $CDCl_3$) δ -57.32 (t, J = 16.6 Hz), -96.48 (dq, J = 24.4, 16.5 Hz).

HRMS (ESI) for $C_{18}H_{20}F_5NS$ $[M+H]^+$ m/z: calcd. 378.1310, found 378.1310.

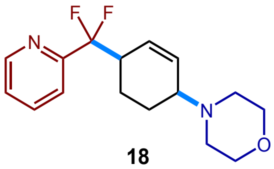

18

4-(4-(difluoro(pyridin-2-yl)methyl)cyclohex-2-en-1-yl)morpholine

Prepared following general procedure A the reaction mixture was purified by column chromatography using 30% EtOAc in hexane to provide the title compound in 49% yield (14.5 mg) as a yellow oil.

R_f=0.25 (35% EtOAc in hexane).

^{1}H NMR (600 MHz, CDCl3) δ 8.66 (d, J = 4.9 Hz, 1H), 7.79 (td, J = 7.8, 1.7 Hz, 1H), 7.59 (d, J = 7.9 Hz, 1H), 7.36 (dd, J = 7.7, 4.8 Hz, 1H), 5.91 (dt, J = 10.5, 2.6 Hz, 1H), 5.82 (dt, J = 10.5, 2.8 Hz, 1H), 3.74 – 3.65 (m, 4H), 3.28 – 3.17 (m, 1H), 3.13 (s, 1H), 2.61 (dt, J = 9.9, 4.3 Hz, 2H), 2.52 (dt, J = 11.0, 4.5 Hz, 2H), 1.83 – 1.74 (m, 2H), 1.67 – 1.53 (m, 2H). **^{13}C NMR** (151 MHz, CDCl3) δ 154.14 (t, J = 28.9 Hz), 149.48, 136.90, 132.22, 126.41, 124.63, 121.71 (t, J = 246.1 Hz), 120.60 (t, J = 4.7 Hz), 67.20, 58.80, 49.34, 40.38 (t, J = 24.2 Hz), 29.71, 20.36 – 19.93 (m). **^{19}F NMR** (565 MHz, CDCl3) δ -103.36 (dd, J = 248.8, 15.4 Hz), -105.39 (dd, J = 249.2, 17.2 Hz).

HRMS (ESI) for $C_{16}H_{20}F_2N_2O$ [M+H]+ m/z: calcd. 295.1617 , found 295.1613.

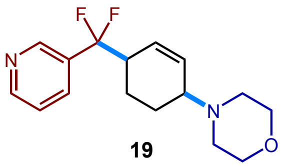

19

4-(4-(difluoro(pyridin-3-yl)methyl)cyclohex-2-en-1-yl)morpholine

Prepared following general procedure A the reaction mixture was purified by column chromatography using 300% EtOAc in hexane to provide the title compound in 70% yield (21 mg) as a yellow oil.

R_f=0.2 (EtOAc).

^{1}H NMR (600 MHz, $CDCl_3$) δ 8.68 (s, 1H), 8.66 (d, *J* = 4.9 Hz, 1H), 7.73 (dt, *J* = 8.0, 2.0 Hz, 1H), 7.34 (dd, *J* = 8.0, 4.8 Hz, 1H), 5.92 (dt, *J* = 10.4, 2.7 Hz, 1H), 5.80 (dt, *J* = 10.4, 2.8 Hz, 1H), 3.62 (t, *J* = 4.7 Hz, 4H), 3.06 – 3.00 (m, 1H), 2.85 – 2.75 (m, 1H), 2.47 (dt, *J* = 11.5, 4.7 Hz, 2H), 2.36 (dt, *J* = 11.3, 4.7 Hz, 2H), 1.82 – 1.74 (m, 1H), 1.67 – 1.61 (m, 1H), 1.58 – 1.51 (m, 1H), 1.50 – 1.43 (m, 1H). **^{13}C NMR** (151 MHz, $CDCl_3$) δ 150.95, 147.19 (t, *J* = 6.6 Hz), 133.75, 133.45 (t, *J* = 6.1 Hz), 131.82 (t, *J* = 27.4 Hz), 125.38, 122.94, 122.46 (t, *J* = 246.7 Hz), 67.30, 58.53, 49.34, 42.78 (t, *J* = 25.8 Hz), 20.29 (t, *J* = 3.4 Hz), 19.91. **^{19}F NMR** (565 MHz, $CDCl_3$) δ -99.06 (dd, *J* = 250.1, 14.3 Hz), -100.81 (dd, *J* = 250.0, 14.4 Hz).

HRMS (ESI) for $C_{16}H_{20}F_2N_2O$ $[M+H]^+$ m/z: calcd. 295.1617, found 295.1616.

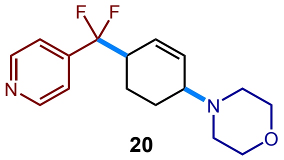

20

4-(4-(difluoro(pyridin-4-yl)methyl)cyclohex-2-en-1-yl)morpholine

Prepared following general procedure A the reaction mixture was purified by column chromatography using EtOAc to provide the title compound in 77% yield (23 mg) as a yellow oil.

R_f =0.2 (EtOAc).

^{1}H NMR (600 MHz, $CDCl_3$) δ 8.69 (d, *J* = 5.3 Hz, 2H), 7.34 (d, *J* = 5.3 Hz, 2H), 5.92 (dt, *J* = 10.4, 2.7 Hz, 1H), 5.77 (dt, *J* = 10.5, 2.8 Hz, 1H), 3.63 (t, *J* = 4.7 Hz, 4H), 3.03 (s, 1H), 2.82 – 2.72 (m, 1H), 2.49 (dt, *J* = 11.4, 4.7 Hz, 2H), 2.39 (dt, *J* = 11.3, 4.7 Hz, 2H), 1.80 – 1.73 (m, 1H), 1.67 – 1.58 (m, 2H), 1.53 – 1.44 (m, 1H). **^{13}C NMR** (151 MHz, $CDCl_3$) δ 150.05, 144.23 (t, *J* = 28.3 Hz), 133.77, 125.10, 122.04 (t, *J* = 246.6 Hz), 120.25 (t, *J* = 6.0 Hz), 67.36, 58.51, 49.42, 42.38 (t, *J* = 25.3 Hz), 20.27 (t, *J* = 3.3 Hz), 20.12. **^{19}F NMR** (565 MHz, $CDCl_3$) δ -101.81 (dd, *J* = 247.1, 15.2 Hz), -102.87 (dd, *J* = 246.8, 14.7 Hz).

HRMS (ESI) for $C_{16}H_{20}F_2N_2O$ $[M+H]^+$ m/z: calcd. 295.1617, found 295.1614.

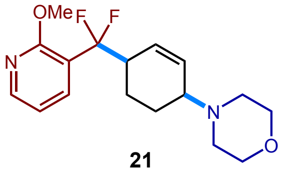

21

4-(4-(difluoro(2-methoxypyridin-3-yl)methyl)cyclohex-2-en-1-yl)morpholine

Prepared following general procedure A the reaction mixture was purified by column chromatography using 30% EtOAc in hexane to provide the title compound in 66% yield (21.5 mg) as a colorless oil.

R_f = 0.1 (50% EtOAc in hexane)

^{1}H NMR (600 MHz, $CDCl_3$) δ 8.22 (dd, J = 5.0, 1.8 Hz, 1H), 7.75 (dd, J = 7.4, 1.9 Hz, 1H), 6.94 (dd, J = 7.4, 5.0 Hz, 1H), 5.93 – 5.82 (m, 1H), 5.80 – 5.66 (m, 1H), 3.98 (s, 3H), 3.75 – 3.65 (m, 4H), 3.33 – 3.19 (m, 1H), 3.14 – 3.04 (m, 1H), 2.67 – 2.58 (m, 2H), 2.57 – 2.47 (m, 2H), 1.90 – 1.81 (m, 1H), 1.79 – 1.71 (m, 1H), 1.61 – 1.52 (m, 2H). **^{13}C NMR** (101 MHz, $CDCl_3$) δ 160.30 (t, J = 4.3 Hz), 148.38, 136.45 (t, J = 8.4 Hz), 132.23, 126.45 – 125.83 (m), 122.14 (t, J = 246.7 Hz), 118.60 (t, J = 27.3 Hz), 116.29, 67.36, 58.73, 53.68, 49.41, 39.60 (t, J = 24.7 Hz), 20.47 (t, J = 3.1 Hz), 20.37. **^{19}F NMR** (377 MHz, $CDCl_3$) δ -101.35 (dd, J = 248.2, 16.7 Hz), -102.54 (dd, J = 248.2, 17.9 Hz).

HRMS (ESI) for $C_{17}H_{22}F_2N_2O_2$ [M+H]+ m/z: calcd. 325.1722, found 325.1720.

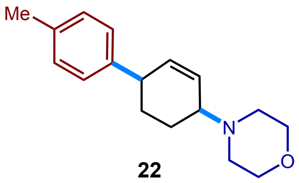

22

4-(4'-methyl-1,2,3,4-tetrahydro-[1,1'-biphenyl]-4-yl)morpholine

Prepared following general procedure C the reaction mixture was purified by column chromatography using 15% EtOAc in hexane to provide the title compound in 53% yield (13.5 mg) as a yellow oil.

R_f=0.5 (EtOAc).

^{1}H NMR (600 MHz, $CDCl_3$) δ 7.16 – 7.09 (m, 4H), 5.92 (s, 2H), 3.80 – 3.70 (m, 4H), 3.41 – 3.36 (m, 1H), 3.25 – 3.20 (m, 1H), 2.74 – 2.68 (m, 2H), 2.64 – 2.57 (m, 2H), 2.33 (s, 3H), 2.02 – 1.94 (m, 1H), 1.78 – 1.72 (m, 1H), 1.72 – 1.64 (m, 1H), 1.60 – 1.53 (m, 1H). **^{13}C NMR** (151 MHz, $CDCl_3$) δ 142.15, 135.71, 132.79, 130.04, 128.99, 127.93, 67.45, 59.95, 49.35, 40.11, 29.99, 21.01, 19.06.

HRMS (ESI) for $C_{17}H_{23}NO$ $[M+H]^+$ m/z: calcd. 258.1853, found 258.1850.

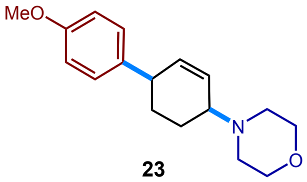

23

4-(4'-methoxy-1,2,3,4-tetrahydro-[1,1'-biphenyl]-4-yl)morpholine

Prepared following general procedure C the reaction mixture was purified by column chromatography using 15% EtOAc in hexane to provide the title compound in 54% yield (15 mg) as a yellow oil.

R_f=0.4 (EtOAc).

^{1}H NMR (600 MHz, $CDCl_3$) δ 7.15 – 7.11 (m, 2H), 6.88 – 6.84 (m, 2H), 5.91 (s, 2H), 3.79 (s, 3H), 3.78 – 3.71 (m, 4H), 3.40 – 3.35 (m, 1H), 3.24 – 3.19 (m, 1H), 2.73 – 2.67 (m, 2H), 2.64

– 2.58 (m, 2H), 2.01 – 1.92 (m, 1H), 1.76 – 1.70 (m, 1H), 1.70 – 1.63 (m, 1H), 1.60 – 1.52 (m, 1H). **^{13}C NMR** (151 MHz, $CDCl_3$) δ 158.02, 137.25, 132.89, 129.94, 128.95, 113.68, 67.43, 59.97, 55.28, 49.32, 39.68, 30.04, 18.96.

HRMS (ESI) for $C_{17}H_{23}NO_2$ $[M+H]^+$ m/z: calcd. 274.1802, found 274.1802.

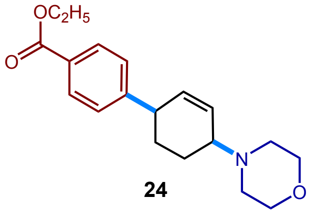

24

ethyl-4'-morpholino-1',2',3',4'-tetrahydro-[1,1'-biphenyl]-4-carboxylate

Prepared following general procedure C the reaction mixture was purified by column chromatography using 25% EtOAc in hexane to provide the title compound in 59% yield (19 mg) as a yellow oil.

R_f=0.2 (25% EtOAc in hexane).

^{1}H NMR (600 MHz, $CDCl_3$) δ 8.01 – 7.96 (m, 2H), 7.28 (d, J = 8.3 Hz, 2H), 5.97 (dt, J = 10.4, 2.3 Hz, 1H), 5.91 (dt, J = 10.2, 2.6 Hz, 1H), 4.36 (q, J = 7.1 Hz, 2H), 3.78 – 3.69 (m, 4H), 3.48 – 3.44 (m, 1H), 3.24 – 3.19 (m, 1H), 2.72 – 2.66 (m, 2H), 2.62 – 2.56 (m, 2H), 2.07 – 1.96 (m, 1H), 1.80 – 1.72 (m, 1H), 1.68 – 1.61 (m, 1H), 1.60 – 1.53 (m, 1H), 1.38 (t, J = 7.1 Hz, 3H). **^{13}C NMR** (151 MHz, $CDCl_3$) δ 166.61, 150.49, 131.71, 130.89, 129.60, 128.50, 128.00, 67.43, 60.85, 59.76, 49.39, 40.55, 29.72, 19.15, 14.36.

HRMS (ESI) for $C_{19}H_{25}NO_3$ $[M+H]^+$ m/z: calcd. 316.1908, found 316.1906.

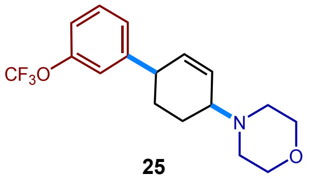

25

4-(3'-(trifluoromethoxy)-1,2,3,4-tetrahydro-[1,1'-biphenyl]-4-yl)morpholine

Prepared following general procedure C the reaction mixture was purified by column chromatography using 15% EtOAc in hexane to provide the title compound in 54% yield (18 mg) as a yellow oil.

R_f=0.4 (EtOAc).

^{1}H NMR (600 MHz, $CDCl_3$) δ 7.32 (t, J = 7.9 Hz, 1H), 7.15 (d, J = 7.6 Hz, 1H), 7.06 (d, J = 7.6 Hz, 2H), 5.97 (dt, J = 10.3, 2.2 Hz, 1H), 5.89 (dt, J = 10.3, 3.1 Hz, 1H), 3.78 – 3.69 (m, 4H), 3.45 – 3.41 (m, 1H), 3.25 – 3.19 (m, 1H), 2.71 – 2.65 (m, 2H), 2.63 – 2.55 (m, 2H), 2.05 – 1.97 (m, 1H), 1.79 – 1.73 (m, 1H), 1.67 – 1.60 (m, 1H), 1.60 – 1.53 (m, 1H). **^{13}C NMR** (151 MHz, $CDCl_3$) δ 149.28, 147.55, 131.49, 131.22, 129.53, 126.44, 120.53, 120.50 (q, J = 256.8

Hz), 118.60, 67.44, 59.83, 49.29, 40.16, 29.75, 18.81. **^{19}F NMR** (565 MHz, $CDCl_3$) δ -57.71.
HRMS (ESI) for $C_{17}H_{20}F_3NO_2$ $[M+H]^+$ m/z: calcd. 328.1519, found 328.1518.

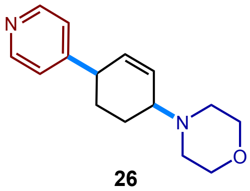

26

4-(4-(pyridin-4-yl)cyclohex-2-en-1-yl)morpholine

Prepared following general procedure C the reaction mixture was purified by column chromatography using EtOAc to provide the title compound in 59% yield (14.5 mg) as a yellow oil.

R$_f$=0.2 (EtOAc).

^{1}H NMR (600 MHz, $CDCl_3$) δ 8.47 (d, J = 2.3 Hz, 1H), 8.44 (dd, J = 4.8, 1.6 Hz, 1H), 7.50 (dt, J = 7.9, 2.0 Hz, 1H), 7.22 (dd, J = 7.8, 4.8 Hz, 1H), 5.97 (dt, J = 10.2, 2.3 Hz, 1H), 5.87 (dt, J = 10.3, 3.2 Hz, 1H), 3.76 – 3.66 (m, 4H), 3.44 – 3.40 (m, 1H), 3.23 – 3.17 (m, 1H), 2.70 – 2.63 (m, 2H), 2.61 – 2.55 (m, 2H), 2.05 – 1.97 (m, 1H), 1.78 – 1.70 (m, 1H), 1.67 – 1.61 (m, 1H), 1.61 – 1.55 (m, 1H). **^{13}C NMR** (151 MHz, $CDCl_3$) δ 149.68, 147.63, 140.32, 135.41, 131.32, 131.08, 123.25, 67.40, 59.65, 49.39, 38.21, 29.60, 18.95.

HRMS (ESI) for $C_{15}H_{20}N_2O$ $[M+H]^+$ m/z: calcd. 245.1649, found 245.1648.

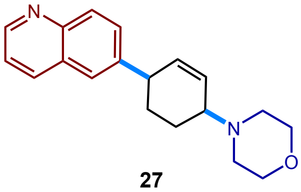

27

4-(4-(quinolin-6-yl)cyclohex-2-en-1-yl)morpholine

Prepared following general procedure C the reaction mixture was purified by column chromatography using 100% EtOAc in hexane to provide the title compound in 57% yield (17 mg) as a yellow oil.

R$_f$=0.2 (100% EtOAc in hexane).

^{1}H NMR (600 MHz, $CDCl_3$) δ 8.87 (dd, J = 4.2, 1.7 Hz, 1H), 8.10 (dd, J = 8.3, 1.0 Hz, 1H), 8.05 (d, J = 9.0 Hz, 1H), 7.62 – 7.58 (m, 2H), 7.38 (dd, J = 8.3, 4.2 Hz, 1H), 6.05 – 5.98 (m, 2H), 3.82 – 3.71 (m, 4H), 3.61 – 3.57 (m, 1H), 3.27 – 3.22 (m, 1H), 2.76 – 2.69 (m, 2H), 2.66 – 2.59 (m, 2H), 2.11 – 2.02 (m, 1H), 1.90 – 1.83 (m, 1H), 1.76 – 1.66 (m, 1H), 1.65 – 1.57 (m, 1H). **^{13}C NMR** (151 MHz, $CDCl_3$) δ 149.91, 147.27, 143.51, 135.83, 132.04, 130.81, 130.49, 129.44, 128.10, 125.98, 121.19, 67.42, 59.85, 49.47, 40.49, 29.58, 19.32.

HRMS (ESI) for $C_{19}H_{22}N_2O$ $[M+H]^+$ m/z: calcd. 295.1805, found 295.1804.

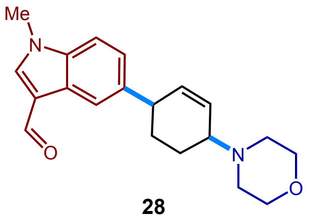

28

1-methyl-5-(4-morpholinocyclohex-2-en-1-yl)-1H-indole-3-carbaldehyde

Prepared following general procedure C the reaction mixture was purified by column chromatography using 100% EtOAc in hexane to provide the title compound in 49% yield (16 mg) as a yellow oil.

R$_f$=0.2 (100% EtOAc in hexane).

^{1}H NMR (600 MHz, $CDCl_3$) δ 9.95 (s, 1H), 8.17 (s, 1H), 7.65 (s, 1H), 7.31 (d, *J* = 8.4 Hz, 1H), 7.23 (dd, *J* = 8.4, 1.7 Hz, 1H), 6.02 – 5.98 (m, 1H), 5.98 – 5.94 (m, 1H), 3.85 (s, 3H), 3.81 – 3.73 (m, 4H), 3.61 – 3.54 (m, 1H), 3.30 – 3.23 (m, 1H), 2.80 – 2.74 (m, 2H), 2.68 – 2.61 (m, 2H), 2.08 – 1.99 (m, 1H), 1.82 – 1.76 (m, 1H), 1.76 – 1.69 (m, 1H), 1.61 – 1.52 (m, 1H). **^{13}C NMR** (151 MHz, $CDCl_3$) δ 184.38, 140.12, 139.51, 136.70, 133.02, 130.14, 125.33, 124.47, 121.34, 117.97, 109.65, 67.46, 59.90, 49.35, 40.54, 33.77, 30.32, 18.78.

HRMS (ESI) for $C_{20}H_{24}N_2O_2$ $[M+H]^+$ m/z: calcd. 325.1911, found 325.1909.

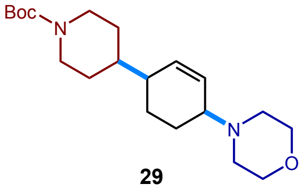

29

tert-butyl 4-(4-morpholinocyclohex-2-en-1-yl)piperidine-1-carboxylate

Prepared following general procedure C the reaction mixture was purified by column chromatography using 30% EtOAc in hexane to provide the title compound in 81% yield (28.5 mg) as a yellow oil.

R$_f$=0.25 (100% EtOAc in hexane).

^{1}H NMR (600 MHz, $CDCl_3$) δ 5.84 – 5.79 (m, 1H), 5.72 – 5.67 (m, 1H), 4.11 (d, *J* = 47.0 Hz, 2H), 3.74 – 3.63 (m, 4H), 3.05 – 3.00 (m, 1H), 2.62 – 2.56 (m, 3H), 2.54 – 2.48 (m, 2H), 2.34 – 2.07 (m, 1H), 1.88 – 1.81 (m, 1H), 1.74 – 1.68 (m, 1H), 1.68 – 1.61 (m, 2H), 1.60 – 1.51 (m, 3H), 1.43 (s, 9H), 1.41 – 1.34 (m, 1H), 1.22 – 1.09 (m, 2H). **^{13}C NMR** (151 MHz, $CDCl_3$) δ 154.82, 133.24, 128.85, 79.27, 67.42, 59.29, 49.69, 45.28 – 43.10 (m), 40.56, 39.38, 29.70, 28.48, 22.92, 21.20.

HRMS (ESI) for $C_{20}H_{34}N_2O_3$$[M+H]^+$ m/z: calcd. 351.2643, found 351.2641.

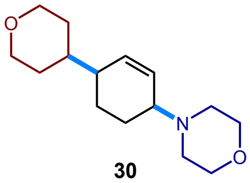

30

4-(4-(tetrahydro-2H-pyran-4-yl)cyclohex-2-en-1-yl)morpholine

Prepared following general procedure C the reaction mixture was purified by column chromatography using 30% EtOAc in hexane to provide the title compound in 69% yield (17.5 mg) as a yellow oil.

R_f=0.25 (100% EtOAc in hexane).

^{1}H NMR (600 MHz, $CDCl_3$) δ 5.88 – 5.82 (m, 1H), 5.71 (dt, J = 10.4, 2.5 Hz, 1H), 4.01 – 3.95 (m, 2H), 3.75 – 3.66 (m, 4H), 3.35 (tt, J = 11.7, 2.3 Hz, 2H), 3.07 – 3.01 (m, 1H), 2.64 – 2.57 (m, 2H), 2.56 – 2.49 (m, 2H), 1.87 – 1.78 (m, 1H), 1.75 – 1.68 (m, 1H), 1.66 – 1.52 (m, 5H), 1.51 – 1.43 (m, 1H), 1.43 – 1.31 (m, 2H). **^{13}C NMR** (151 MHz, $CDCl_3$) δ 133.04, 128.86, 68.30, 68.27, 67.43, 59.47, 49.66, 39.66, 39.41, 30.73, 30.71, 22.86, 21.03.

HRMS (ESI) for $C_{15}H_{25}NO_2$ $[M+H]^+$ m/z: calcd. 252.1959, found 252.1956.

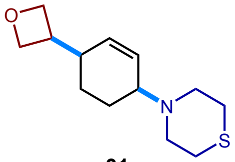

31

4-(4-(oxetan-3-yl)cyclohex-2-en-1-yl)thiomorpholine

Prepared following general procedure C the reaction mixture was purified by column chromatography using 25% EtOAc in hexane to provide the title compound in 73% yield (17.5 mg) as a yellow oil.

R_f=0.25 (25% EtOAc in hexane).

^{1}H NMR (400 MHz, $CDCl_3$) δ 5.76 – 5.70 (m, 1H), 5.70 – 5.65 (m, 1H), 4.79 – 4.72 (m, 2H), 4.52 (t, J = 6.2 Hz, 1H), 4.44 (t, J = 6.2 Hz, 1H), 3.19 – 3.11 (m, 1H), 2.95 – 2.83 (m, 3H), 2.80 – 2.71 (m, 2H), 2.71 – 2.59 (m, 4H), 2.47 – 2.37 (m, 1H), 1.72 – 1.49 (m, 3H), 1.37 – 1.26 (m, 1H). **^{13}C NMR** (101 MHz, $CDCl_3$) δ 131.08, 130.56, 76.60, 75.81, 61.17, 51.32, 39.21, 37.93, 28.51, 23.92, 19.14.

HRMS (ESI) for $C_{13}H_{21}NOS$ $[M+H]^+$ m/z: calcd. 240.1417, found 240.1415.

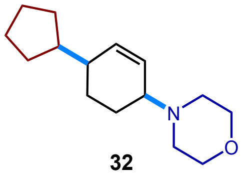

32

4-(4-cyclopentylcyclohex-2-en-1-yl)morpholine

Prepared following general procedure C the reaction mixture was purified by column chromatography using 20% EtOAc in hexane to provide the title compound in 52% yield (12.5 mg) as a yellow oil.

R$_f$ =0.25 (20% EtOAc in hexane).

^{1}H NMR (600 MHz, $CDCl_3$) δ 5.86 (dt, J = 10.4, 2.9 Hz, 1H), 5.63 (dt, J = 10.4, 2.4 Hz, 1H), 3.74 – 3.65 (m, 4H), 3.08 – 3.02 (m, 1H), 2.64 – 2.57 (m, 2H), 2.56 – 2.49 (m, 2H), 1.81 – 1.74 (m, 3H), 1.74 – 1.70 (m, 1H), 1.70 – 1.62 (m, 2H), 1.62 – 1.56 (m, 2H), 1.56 – 1.45 (m, 4H), 1.22 – 1.07 (m, 2H). **^{13}C NMR** (151 MHz, $CDCl_3$) δ 134.71, 127.91, 67.49, 59.69, 49.57, 44.96, 40.37, 31.30, 30.64, 25.63, 25.45, 25.10, 20.59.

HRMS (ESI) for $C_{15}H_{25}NO$ $[M+H]^+$ m/z: calcd.236.2009, found 236.2006.

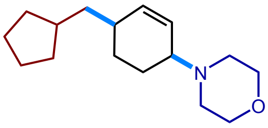

33

4-(4-(cyclopentylmethyl)cyclohex-2-en-1-yl)morpholine

Prepared following general procedure C the reaction mixture was purified by column chromatography using 10% EtOAc in hexane to provide the title compound in 74% yield (18.5 mg) as a yellow oil.

R$_f$ =0.15 (10% EtOAc in hexane).

^{1}H NMR (600 MHz, $CDCl_3$) δ 5.81 (dt, J = 10.4, 3.1 Hz, 1H), 5.60 (dt, J = 10.5, 2.4 Hz, 1H), 3.75 – 3.66 (m, 4H), 3.09 – 3.05 (m, 1H), 2.64 – 2.57 (m, 2H), 2.53 (dt, J = 11.4, 3.8 Hz, 2H), 2.04 (s, 1H), 1.91 – 1.80 (m, 1H), 1.80 – 1.71 (m, 2H), 1.70 – 1.63 (m, 2H), 1.63 – 1.54 (m, 3H), 1.54 – 1.45 (m, 3H), 1.39 – 1.26 (m, 2H), 1.13 – 1.00 (m, 2H). **^{13}C NMR** (151 MHz, $CDCl_3$) δ 135.53, 127.58, 67.48, 60.07, 49.44, 41.83, 37.75, 33.40, 33.00, 32.8, 26.27, 25.17, 25.10, 20.04.

HRMS (ESI) for $C_{16}H_{27}NO$ $[M+H]^+$ m/z: calcd. 250.2166, found 250.2163.

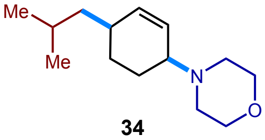

34

4-(4-isobutylcyclohex-2-en-1-yl)morpholine

Prepared following general procedure C the reaction mixture was purified by column chromatography using 5% EtOAc in hexane to provide the title compound in 58% yield (13 mg) as a yellow oil.

R$_f$ =0.25 (20% EtOAc in hexane).

^{1}H NMR (600 MHz, $CDCl_3$) δ 5.77 (dt, J = 9.8, 3.0 Hz, 1H), 5.61 (dt, J = 10.3, 2.4 Hz, 1H),

3.75 – 3.66 (m, 4H), 3.10 – 3.03 (m, 1H), 2.64 – 2.57 (m, 2H), 2.56 – 2.50 (m, 2H), 2.12 – 2.04 (m, 1H), 1.72 – 1.61 (m, 3H), 1.59 – 1.53 (m, 1H), 1.46 – 1.40 (m, 1H), 1.23 – 1.11 (m, 2H), 0.89 (d, J = 6.6 Hz, 3H), 0.86 (d, J = 6.6 Hz, 3H). **^{13}C NMR** (151 MHz, $CDCl_3$) δ 135.44, 127.65, 67.47, 60.02, 49.47, 44.59, 31.89, 26.17, 25.36, 22.97, 22.54, 20.04.

HRMS (ESI) for $C_{14}H_{25}NO$ $[M+H]^+$ m/z: calcd.224.2009, found 224.2007.

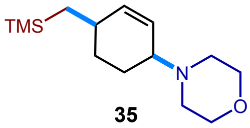

35

4-(4-((trimethylsilyl)methyl)cyclohex-2-en-1-yl)morpholine

Prepared following general procedure C the reaction mixture was purified by column chromatography using 15% EtOAc in hexane to provide the title compound in 36% yield (9.3 mg) as a yellow oil.

R_f=0.20 (15% EtOAc in hexane).

^{1}H NMR (600 MHz, $CDCl_3$) δ 5.78 (dt, J = 10.2, 2.6 Hz, 1H), 5.55 (dt, J = 10.2, 1.8 Hz, 1H), 3.78 – 3.64 (m, 4H), 3.06 (dp, J = 7.8, 2.5 Hz, 1H), 2.65 – 2.58 (m, 2H), 2.57 – 2.49 (m, 2H), 2.21 – 2.15 (m, 1H), 1.79 – 1.68 (m, 2H), 1.60 – 1.52 (m, 1H), 1.46 – 1.40 (m, 1H), 0.68 (dd, J = 14.6, 7.5 Hz, 1H), 0.59 (dd, J = 14.6, 7.5 Hz, 1H), 0.02 (s, 9H). **^{13}C NMR** (151 MHz, $CDCl_3$) δ 137.57, 126.60, 67.48, 59.71, 49.42, 30.92, 29.52, 23.68, 19.74, -0.67.

HRMS (ESI) for $C_{14}H_{27}NOSi$ $[M+H]^+$ m/z: calcd. 254.1935, found 254.1932.

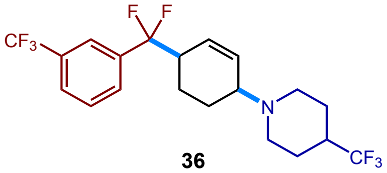

36

1-(4-(difluoro(3-(trifluoromethyl)phenyl)methyl)cyclohex-2-en-1-yl)-4-(trifluoromethyl)piperidine

Prepared following general procedure A the reaction mixture was purified by column chromatography using 25% EtOAc in hexane to provide the title compound in 72% yield (31 mg) as a colorless oil.

R_f= 0.35 (25% EtOAc in hexane)

^{1}H NMR (400 MHz, $CDCl_3$) δ 7.76 – 7.61 (m, 3H), 7.56 (t, J = 7.7 Hz, 1H), 5.96 – 5.86 (m, 1H), 5.85 – 5.76 (m, 1H), 3.18 – 3.07 (m, 1H), 2.91 – 2.67 (m, 3H), 2.35 – 2.19 (m, 1H), 2.05 – 1.96 (m, 1H), 1.95 – 1.83 (m, 1H), 1.83 – 1.73 (m, 3H), 1.72 – 1.62 (m, 1H), 1.61 – 1.40 (m, 4H). **^{13}C NMR** (101 MHz, $CDCl_3$) δ 137.12 (t, J = 27.7 Hz), 134.43, 130.80 (q, J = 32.8 Hz), 129.07 (t, J = 6.4 Hz), 128.87, 127.48 (q, J = 278.3 Hz), 126.75 – 126.42 (m), 125.39 – 124.86

(m), 123.72 (q, J = 270.3 Hz), 123.12 – 122.52 (m), 122.70 (t, J = 247.0 Hz), 58.74, 49.13, 46.84, 42.51 (t, J = 26.0 Hz), 40.51 (q, J = 27.1 Hz), 25.57 – 23.55 (m), 20.57 (t, J = 3.4 Hz), 19.27. **^{19}F NMR** (377 MHz, $CDCl_3$) δ -62.74, -73.77 (d, J = 8.4 Hz), -98.34 (dd, J = 245.5, 13.8 Hz), -100.55 (dd, J = 245.6, 14.7 Hz).

HRMS (ESI) for $C_{20}H_{21}F_8N$ $[M+H]^+$ m/z: calcd. 428.1619, found 428.1623.

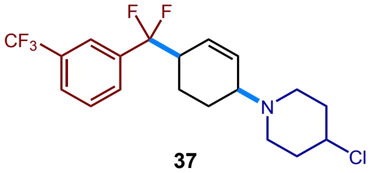

37

4-chloro-1-(4-(difluoro(3-(trifluoromethyl)phenyl)methyl)cyclohex-2-en-1-yl)piperidine

Prepared following general procedure A the reaction mixture was purified by column chromatography using 15% EtOAc in hexane to provide the title compound in 71% yield (28 mg) as a colorless oil.

R_f = 0.45 (25% EtOAc in hexane)

^{1}H NMR (400 MHz, $CDCl_3$) δ 7.73 – 7.68 (m, 2H), 7.67 – 7.62 (m, 1H), 7.60 – 7.52 (m, 1H), 5.97 – 5.87 (m, 1H), 5.86 – 5.72 (m, 1H), 4.11 – 3.87 (m, 1H), 3.22 – 3.01 (m, 1H), 2.95 – 2.76 (m, 1H), 2.76 – 2.55 (m, 2H), 2.45 – 2.33 (m, 1H), 2.30 – 2.13 (m, 1H), 2.11 – 1.99 (m, 2H), 1.92 – 1.75 (m, 3H), 1.70 – 1.58 (m, 1H), 1.55 – 1.42 (m, 2H). **^{13}C NMR** (101 MHz, $CDCl_3$) δ 137.13 (t, J = 27.6 Hz), 134.64 – 134.01 (m), 130.80 (q, J = 32.8 Hz), 129.07 (t, J = 6.3 Hz), 128.91, 126.59, 125.45 – 124.73 (m), 123.73 (q, J = 272.5 Hz), 122.99 – 122.53 (m), 122.70 (t, J = 247.1 Hz), 58.67, 57.61, 46.44, 42.51 (t, J = 26.1 Hz), 35.86, 20.58 (t, J = 3.4 Hz), 19.40. **^{19}F NMR** (377 MHz, $CDCl_3$) δ -62.71, -98.52 (dd, J = 245.7, 14.0 Hz), -100.49 (dd, J = 245.6, 14.6 Hz).

HRMS (ESI) for $C_{19}H_{21}ClF_5N$ $[M+H]^+$ m/z: calcd. 394.1355, found 394.1356.

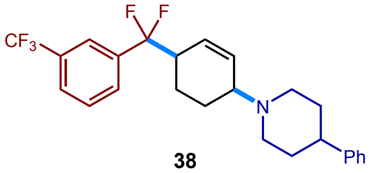

38

1-(4-(difluoro(3-(trifluoromethyl)phenyl)methyl)cyclohex-2-en-1-yl)-4-phenylpiperidine

Prepared following general procedure A the reaction mixture was purified by column chromatography using 15% EtOAc in hexane to provide the title compound in 62% yield (27 mg) as a colorless oil.

R_f = 0.3 (25% EtOAc in hexane)

^{1}H NMR (400 MHz, $CDCl_3$) δ 7.77 – 7.64 (m, 3H), 7.61 – 7.52 (m, 1H), 7.35 – 7.27 (m, 2H), 7.25 – 7.15 (m, 3H), 6.07 – 5.92 (m, 1H), 5.88 – 5.76 (m, 1H), 3.28 – 3.14 (m, 1H), 2.93 – 2.71

(m, 3H), 2.53 – 2.37 (m, 2H), 2.22 – 2.11 (m, 1H), 1.91 – 1.63 (m, 6H), 1.52 (q, J = 6.6 Hz, 2H). **^{13}C NMR** (101 MHz, $CDCl_3$) δ 146.34, 137.15 (t, J = 27.6 Hz), 134.75, 130.74 (q, J = 32.8 Hz), 129.06 (t, J = 6.2 Hz), 128.83, 128.36, 126.83, 126.52, 126.06, 124.78 (t, J = 5.1 Hz), 123.71 (q, J = 272.5 Hz), 123.05 – 122.54 (m), 122.72 (t, J = 246.9 Hz), 58.91, 50.79, 48.44, 42.85, 42.46 (t, J = 26.0 Hz), 33.81, 33.79, 20.73 (t, J = 3.4 Hz), 19.08. **^{19}F NMR** (377 MHz, $CDCl_3$) δ -62.68, -98.14 (dd, J = 245.4, 13.8 Hz), -100.55 (dd, J = 245.4, 14.8 Hz).
HRMS (ESI) for $C_{25}H_{26}F_5N$ $[M+H]^+$ m/z: calcd. 436.2058, found 436.2060.

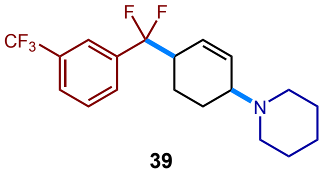

39

1-(4-(difluoro(3-(trifluoromethyl)phenyl)methyl)cyclohex-2-en-1-yl)piperidine

Prepared following general procedure A the reaction mixture was purified by column chromatography using 30% EtOAc in hexane to provide the title compound in 53% yield (19 mg) as a colorless oil.

R_f = 0.2 (100% EtOAc in hexane)

^{1}H NMR (600 MHz, $CDCl_3$) δ 7.70 (d, J = 8.6 Hz, 2H), 7.65 (d, J = 7.8 Hz, 1H), 7.56 (t, J = 7.7 Hz, 1H), 6.04 – 5.95 (m, 1H), 5.87 – 5.73 (m, 1H), 3.25 – 3.15 (m, 1H), 2.89 – 2.74 (m, 1H), 2.57 – 2.44 (m, 2H), 2.42 – 2.30 (m, 2H), 1.85 – 1.76 (m, 1H), 1.66 (ddd, J = 11.2, 6.9, 4.2 Hz, 1H), 1.63 – 1.53 (m, 4H), 1.53 – 1.46 (m, 2H), 1.45 – 1.37 (m, 2H). ^{13}C NMR (101 MHz, $CDCl_3$) δ 137.82 – 136.55 (m), 134.38 – 134.01 (m), 130.80 (q, J = 32.8 Hz), 129.07 (t, J = 6.4 Hz), 128.90, 126.58 (d, J = 4.2 Hz), 125.46 – 124.90 (m), 123.74 (q, J = 274.7 Hz), 122.79 (dd, J = 6.4, 3.7 Hz), 122.67, (t, J = 244.4 Hz), 59.41, 49.84, 42.39 (t, J = 26.0 Hz), 26.02, 24.36, 20.71 (t, J = 3.4 Hz), 18.89. ^{19}F NMR (377 MHz, $CDCl_3$) δ -62.74, -98.23 (dd, J = 245.4, 13.8 Hz), -100.54 (dd, J = 245.4, 14.9 Hz).
HRMS (ESI) for $C_{19}H_{22}F_5N$ $[M+H]^+$ m/z: calcd. 360.1745, found 360.1744

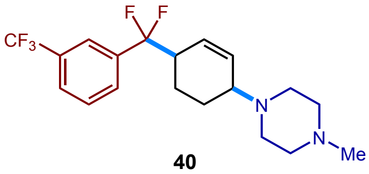

40

1-(4-(difluoro(3-(trifluoromethyl)phenyl)methyl)cyclohex-2-en-1-yl)-4-methylpiperazine

Prepared following general procedure A the reaction mixture was purified by column chromatography using 5% MeOH in DCM to provide the title compound in 53% yield (20 mg) as a yellow oil.

R_f = 0.6 (10% MeOH in DCM)

^{1}H NMR (400 MHz, $CDCl_3$) δ 7.72 – 7.62 (m, 3H), 7.55 (t, *J* = 7.7 Hz, 1H), 5.99 – 5.88 (m, 1H), 5.85 – 5.76 (m, 1H), 3.13 – 3.04 (m, 1H), 2.82 (tdd, *J* = 8.6, 5.9, 2.2 Hz, 1H), 2.73 – 2.58 (m, 1H), 2.57 – 2.46 (m, 3H), 2.45 – 2.33 (m, 4H), 2.28 (s, 3H), 1.87 – 1.72 (m, 1H), 1.70 – 1.57 (m, 1H), 1.51 – 1.37 (m, 2H). **^{13}C NMR** (151 MHz, $CDCl_3$) δ 137.02 (t, *J* = 27.8 Hz), 134.21, 130.73 (q, *J* = 32.9 Hz), 129.05 (t, *J* = 5.9 Hz), 128.83, 126.56 (q, *J* = 3.5 Hz), 125.15 (t, *J* = 5.2 Hz), 123.70 (q, *J* = 272.3 Hz), 123.01 – 122.67 (m), 122.66 (t, *J* = 246.8 Hz), 58.27, 55.32, 48.79 – 47.97 (m), 45.82, 42.60 (t, *J* = 26.1 Hz), 20.45 (t, *J* = 3.4 Hz), 19.40. **^{19}F NMR** (377 MHz, $CDCl_3$) δ -62.71, -98.11 (dd, *J* = 245.5, 13.7 Hz), -100.50 (dd, *J* = 245.6, 14.5 Hz).
HRMS (ESI) for $C_{19}H_{23}F_5N_2$ $[M+H]^+$ m/z: calcd. 375.1854, found 375.1857.

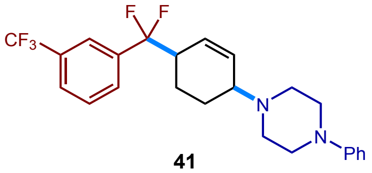

41

1-(4-(difluoro(3-(trifluoromethyl)phenyl)methyl)cyclohex-2-en-1-yl)-4-phenylpiperazine

Prepared following general procedure A the reaction mixture was purified by column chromatography using 10% EtOAc in hexane to provide the title compound in 64% yield (28 mg) as a colorless oil.

R$_f$ = 0.35 (25% EtOAc in hexane)

^{1}H NMR (400 MHz, $CDCl_3$) δ 7.75 – 7.64 (m, 3H), 7.57 (t, *J* = 8.0 Hz, 1H), 7.30 – 7.23 (m, 2H), 6.96 – 6.89 (m, 2H), 6.89 – 6.82 (m, 1H), 6.03 – 5.94 (m, 1H), 5.90 – 5.81 (m, 1H), 3.25 – 3.16 (m, 1H), 3.16 – 3.06 (m, 4H), 2.93 – 2.78 (m, 1H), 2.71 – 2.61 (m, 2H), 2.60 – 2.48 (m, 2H), 1.87 – 1.78 (m, 1H), 1.72 – 1.63 (m, 1H), 1.55 – 1.46 (m, 2H). **^{13}C NMR** (101 MHz, $CDCl_3$) δ 151.38, 137.12 (t, *J* = 27.6 Hz), 134.10, 130.81 (q, *J* = 32.6 Hz), 129.11, 128.93, 126.83 – 126.27 (m), 125.63 – 125.24 (m), 123.77 (q, *J* = 273.7 Hz), 123.21 – 122.78 (m), 122.74 (t, *J* = 247.4 Hz), 119.74, 116.15, 58.45, 49.60, 48.76, 42.69 (t, *J* = 26.0 Hz), 20.52 (t, *J* = 3.4 Hz), 19.57. **^{19}F NMR** (377 MHz, $CDCl_3$) δ -62.66, -98.28 (dd, *J* = 245.6, 13.8 Hz), -100.47 (dd, *J* = 245.6, 14.5 Hz).

HRMS (ESI) for $C_{24}H_{25}F_5N_2$ $[M+H]^+$ m/z: calcd. 437.2011, found 437.2014.

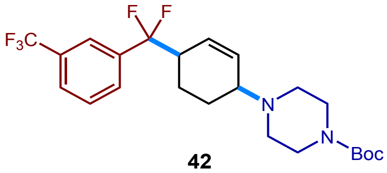

42

tert-butyl 4-(4-(difluoro(3-(trifluoromethyl)phenyl)methyl)cyclohex-2-en-1-yl)piperazine-1-carboxylate

Prepared following general procedure A the reaction mixture was purified by column

chromatography using 20% EtOAc in hexane to provide the title compound in 82% yield (38 mg) as a colorless oil.

R$_f$ = 0.6 (50% EtOAc in hexane)

^{1}H NMR (400 MHz, $CDCl_3$) δ 7.72 – 7.67 (m, 2H), 7.66 – 7.61 (m, 1H), 7.59 – 7.52 (m, 1H), 5.98 – 5.86 (m, 1H), 5.85 – 5.73 (m, 1H), 3.43 – 3.28 (m, 4H), 3.16 – 3.04 (m, 1H), 2.92 – 2.72 (m, 1H), 2.50 – 2.38 (m, 2H), 2.35 – 2.22 (m, 2H), 1.82 – 1.72 (m, 1H), 1.68 – 1.58 (m, 1H), 1.55 – 1.46 (m, 2H), 1.44 (s, 9H). **^{13}C NMR** (101 MHz, $CDCl_3$) δ 154.70, 137.09 (t, *J* = 27.6 Hz), 133.94, 130.80 (q, *J* = 32.6 Hz), 129.06 (t, *J* = 6.06 Hz), 128.91, 126.61, 125.41 (t, *J* = 5.0 Hz), 123.72 (q, *J* = 272.3 Hz), 122.86 – 122.63 (m), 122.67 (t, *J* = 247.4 Hz), 79.57, 58.55, 48.65, 42.60 (t, *J* = 26.0 Hz), 28.41, 20.45 (t, *J* = 3.4 Hz), 19.57. **^{19}F NMR** (377 MHz, $CDCl_3$) δ -62.72, -98.69 (dd, *J* = 245.7, 14.1 Hz), -100.55 (dd, *J* = 245.7, 14.6 Hz).

HRMS (ESI) for $C_{23}H_{29}F_5N_2O_2$ $[M+H]^+$ m/z: calcd. 461.2222, found 461.2225.

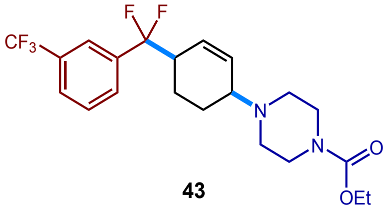

43

ethyl 4-(4-(difluoro(3-(trifluoromethyl)phenyl)methyl)cyclohex-2-en-1-yl) piperazine-1-carboxylate

Prepared following general procedure A the reaction mixture was purified by column chromatography using 25% EtOAc in hexane to provide the title compound in 56% yield (24 mg) as a colorless oil.

R$_f$ = 0.35 (50% EtOAc in hexane)

^{1}H NMR (400 MHz, $CDCl_3$) δ 7.73 – 7.66 (m, 2H), 7.66 – 7.61 (m, 1H), 7.56 (t, *J* = 7.7 Hz, 1H), 5.96 – 5.86 (m, 1H), 5.86 – 5.77 (m, 1H), 4.12 (q, *J* = 7.1 Hz, 2H), 3.50 – 3.30 (m, 4H), 3.18 – 3.04 (m, 1H), 2.93 – 2.71 (m, 1H), 2.55 – 2.39 (m, 2H), 2.32 (s, 2H), 1.84 – 1.69 (m, 1H), 1.69 – 1.55 (m, 1H), 1.53 – 1.40 (m, 2H), 1.25 (t, *J* = 7.1 Hz, 3H). **^{13}C NMR** (101 MHz, $CDCl_3$) δ 155.46, 137.07 (t, *J* = 27.5 Hz), 133.90, 123.72 (q, *J* = 272.4 Hz), 130.81 (q, *J* = 32.8 Hz), 129.05 (t, *J* = 6.0 Hz), 128.91, 126.78 – 126.25 (m), 125.72 – 125.29 (m), 123.08 – 122.48 (m), 122.67 (t, *J* = 247.0 Hz), 61.31, 58.58, 48.60, 44.79 – 43.25 (m), 42.61 (t, *J* = 26.0 Hz), 20.43 (t, *J* = 3.4 Hz), 19.59, 14.68. **^{19}F NMR** (377 MHz, $CDCl_3$) δ -62.73, -98.72 (dd, *J* = 245.9, 14.1 Hz), -100.55 (dd, *J* = 245.8, 14.6 Hz).

HRMS (ESI) for $C_{21}H_{25}F_5N_2O_2$ $[M+H]^+$ m/z: calcd. 433.1909, found 433.1910.

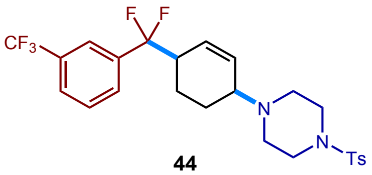

44

1-(4-(difluoro(3-(trifluoromethyl)phenyl)methyl)cyclohex-2-en-1-yl)-4-tosylpiperazine

Prepared following general procedure A the reaction mixture was purified by column chromatography using 10% EtOAc in hexane to provide the title compound in 83% yield (43 mg) as a colorless oil.

R$_f$ = 0.25 (25% EtOAc in hexane)

^{1}H NMR (400 MHz, $CDCl_3$) δ 7.70 – 7.64 (m, 2H), 7.62 (dd, J = 8.3, 2.2 Hz, 3H), 7.54 (t, J = 7.7 Hz, 1H), 7.35 – 7.28 (m, 2H), 5.80 (s, 2H), 3.14 – 3.00 (m, 1H), 2.99 – 2.87 (m, 4H), 2.86 – 2.73 (m, 1H), 2.62 – 2.50 (m, 2H), 2.47 – 2.36 (m, 5H), 1.81 – 1.67 (m, 1H), 1.60 (dq, J = 13.6, 6.6 Hz, 1H), 1.48 – 1.35 (m, 2H). **^{13}C NMR** (101 MHz, $CDCl_3$) δ 143.63, 136.98 (t, J = 27.4 Hz), 133.50, 132.40, 130.78 (q, J = 32.7 Hz), 129.61, 129.21 – 128.88 (m), 128.91, 127.87, 126.62, 125.93 – 125.47 (m), 123.71 (q, J = 272.7 Hz), 122.88 – 122.51 (m), 122.61 (t, J = 247.2 Hz), 58.23, 48.05, 46.43, 42.62 (t, J = 26.1 Hz), 21.49, 20.27 (t, J = 3.5 Hz), 19.79. **^{19}F NMR** (377 MHz, $CDCl_3$) δ -62.70, -98.63 (dd, J = 246.0, 14.1 Hz), -100.47 (dd, J = 246.0, 14.4 Hz).

HRMS (ESI) for $C_{25}H_{27}F_5N_2O_2S$ $[M+H]^+$ m/z: calcd. 515.1786, found 515.1789.

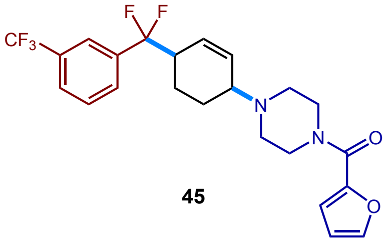

45

(4-(4-(difluoro(3-(trifluoromethyl)phenyl)methyl)cyclohex-2-en-1-yl)piperazin-1-yl)(furan-2-yl)methanone

Prepared following general procedure A the reaction mixture was purified by column chromatography using 50% EtOAc in hexane to provide the title compound in 92% yield (42 mg) as a yellow oil.

R$_f$ = 0.2 (50% EtOAc in hexane)

^{1}H NMR (400 MHz, $CDCl_3$) δ 7.76 – 7.67 (m, 2H), 7.66 – 7.62 (m, 1H), 7.56 (t, J = 7.7 Hz, 1H), 7.49 – 7.46 (m, 1H), 6.97 (d, J = 3.4 Hz, 1H), 6.53 – 6.43 (m, 1H), 6.04 – 5.89 (m, 1H), 5.89 – 5.77 (m, 1H), 3.98 – 3.53 (m, 4H), 3.29 – 3.05 (m, 1H), 2.94 – 2.73 (m, 1H), 2.66 – 2.28 (m, 4H), 1.88 – 1.71 (m, 1H), 1.71 – 1.58 (m, 1H), 1.56 – 1.39 (m, 2H). **^{13}C NMR** (101 MHz,

$CDCl_3$) δ 159.02, 147.88, 143.65, 137.04 (t, *J* = 27.6 Hz), 132.31 – 131.76 (m), 130.82 (q, *J* = 32.9 Hz), 129.21 – 128.94 (m), 128.98, 126.79 – 126.47 (m), 126.02 – 125.65 (m), 123.71 (q, *J* = 272.6 Hz), 122.96 – 122.54 (m), 122.36 (t, *J* = 247.3 Hz), 116.36, 111.26, 58.51, 48.96, 42.58 (t, *J* = 26.0 Hz), 20.37 (t, *J* = 3.5 Hz), 19.62. **^{19}F NMR** (377 MHz, $CDCl_3$) δ -62.69, -98.86 (dd, *J* = 246.0, 14.1 Hz), -100.43 (dd, *J* = 245.9, 14.6 Hz).

HRMS (ESI) for $C_{23}H_{23}F_5N_2O_2$ $[M+H]^+$ m/z: calcd. 455.1752, found 455.1756.

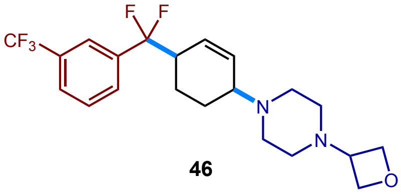

46

1-(4-(difluoro(3-(trifluoromethyl)phenyl)methyl)cyclohex-2-en-1-yl)-4-(oxetan-3-yl)piperazine

Prepared following general procedure A the reaction mixture was purified by column chromatography using 8% MeOH in DCM to provide the title compound in 62% yield (26 mg) as a colorless oil.

R$_f$ = 0.5 (10% MeOH in DCM)

^{1}H NMR (400 MHz, $CDCl_3$) δ 7.76 – 7.61 (m, 3H), 7.55 (t, *J* = 7.7 Hz, 1H), 6.03 – 5.88 (m, 1H), 5.86 – 5.75 (m, 1H), 4.72 – 4.52 (m, 4H), 3.57 – 3.38 (m, 1H), 3.17 – 3.06 (m, 1H), 2.94 – 2.75 (m, 1H), 2.61 – 2.52 (m, 2H), 2.52 – 2.09 (m, 6H), 1.82 – 1.75 (m, 1H), 1.69 – 1.60 (m, 1H), 1.51 – 1.39 (m, 2H). **^{13}C NMR** (151 MHz, $CDCl_3$) δ 137.01 (t, *J* = 27.9 Hz), 134.09, 130.72 (q, *J* = 32.9 Hz), 129.02 (t, *J* = 5.8 Hz), 128.86, 126.60, 125.29, 123.69 (q, *J* = 273.3 Hz), 122.88 – 122.67 (m), 122.64 (q, *J* = 246.1 Hz), 75.45, 59.14, 58.36, 49.85, 48.09, 42.55 (t, *J* = 26.2 Hz), 20.46 (d, *J* = 3.4 Hz), 19.36. **^{19}F NMR** (565 MHz, $CDCl_3$) δ -62.70, -98.21 (dd, *J* = 245.3, 13.4 Hz), -100.46 (dd, *J* = 245.7, 14.7 Hz).

HRMS (ESI) for $C_{21}H_{25}F_5N_2O$ $[M+H]^+$ m/z: calcd. 417.1960, found 417.1960.

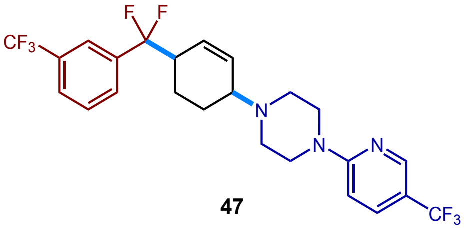

47

1-(4-(difluoro(3-(trifluoromethyl)phenyl)methyl)cyclohex-2-en-1-yl)-4-(5-(trifluoromethyl)pyridin-2-yl)piperazine

Prepared following general procedure A the reaction mixture was purified by column chromatography using 15% EtOAc in hexane to provide the title compound in 61% yield (31

mg) as a colorless oil.

R$_f$ = 0.2 (25% EtOAc in hexane)

^{1}H NMR (400 MHz, $CDCl_3$) δ 8.38 (d, *J* = 2.5 Hz, 1H), 7.71 (d, *J* = 8.3 Hz, 2H), 7.66 (d, *J* = 8.0 Hz, 1H), 7.63 – 7.53 (m, 2H), 6.60 (d, *J* = 9.0 Hz, 1H), 6.02 – 5.92 (m, 1H), 5.88 – 5.80 (m, 1H), 3.70 – 3.47 (m, 4H), 3.24 – 3.11 (m, 1H), 2.93 – 2.76 (m, 1H), 2.65 – 2.55 (m, 2H), 2.52 – 2.42 (m, 2H), 1.87 – 1.74 (m, 1H), 1.71 – 1.59 (m, 1H), 1.55 – 1.40 (m, 2H). **^{13}C NMR** (101 MHz, $CDCl_3$) δ 160.37, 145.76 (q, *J* = 4.4 Hz), 137.13 (t, *J* = 27.6 Hz), 134.46 (q, *J* = 3.1 Hz), 133.81, 130.84 (q, *J* = 32.7 Hz), 129.09 (t, *J* = 6.06 Hz), 128.97, 126.85 – 126.41 (m), 125.81 – 125.47 (m), 124.65 (q, *J* = 270.3 Hz), 123.77 (q, *J* = 273.7 Hz), 123.03 – 122.75 (m), 122.70 (t, *J* = 246.8 Hz), 115.04 (q, *J* = 33.0 Hz), 105.54, 58.54, 48.55, 45.10, 42.69 (t, *J* = 26.1 Hz), 20.46 (t, *J* = 3.4 Hz), 19.69. **^{19}F NMR** (377 MHz, $CDCl_3$) δ -61.10, -62.68, -98.70 (dd, *J* = 245.8, 14.1 Hz), -100.47 (dd, *J* = 245.8, 14.6 Hz).

HRMS (ESI) for $C_{24}H_{23}F_8N_3$ $[M+H]^+$ m/z: calcd. 506.1837, found 506.1841.

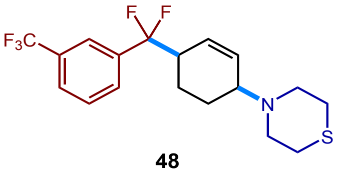

48

4-(4-(difluoro(3-(trifluoromethyl)phenyl)methyl)cyclohex-2-en-1-yl)thiomorpholine

Prepared following general procedure A the reaction mixture was purified by column chromatography using 15% EtOAc in hexane to provide the title compound in 85% yield (32 mg) as a yellow oil.

R$_f$ = 0.3 (25% EtOAc in hexane)

^{1}H NMR (400 MHz, $CDCl_3$) δ 7.74 – 7.62 (m, 3H), 7.56 (t, *J* = 7.7 Hz, 1H), 5.94 – 5.84 (m, 1H), 5.83 – 5.77 (m, 1H), 3.14 – 3.03 (m, 1H), 2.89 – 2.70 (m, 3H), 2.67 – 2.53 (m, 6H), 1.83 – 1.75 (m, 1H), 1.69 – 1.59 (m, 1H), 1.50 – 1.36 (m, 2H). **^{13}C NMR** (101 MHz, $CDCl_3$) δ 137.15 (t, *J* = 27.6 Hz), 134.94, 130.83 (q, *J* = 32.9 Hz), 129.21 – 128.97 (m), 128.94, 126.98 – 126.28 (m), 125.26 (t, *J* = 5.1 Hz), 123.75 (q, *J* = 273 Hz), 123.09 – 122.46 (m), 122.72 (t, *J* = 246.9 Hz), 60.42, 51.24, 42.38 (t, *J* = 26.1 Hz), 28.56, 20.69 (t, *J* = 3.5 Hz), 18.96. **^{19}F NMR** (377 MHz, $CDCl_3$) δ -62.73, -98.09 (dd, *J* = 245.6, 13.8 Hz), -100.39 (dd, *J* = 245.8, 14.8 Hz).

HRMS (ESI) for $C_{18}H_{20}F_5NS$ $[M+H]^+$ m/z: calcd. 378.1309, found 378.1313.

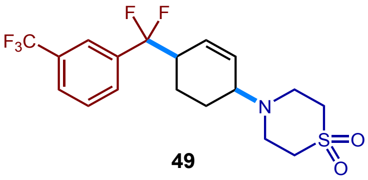

49

4-(4-(difluoro(3-(trifluoromethyl)phenyl)methyl)cyclohex-2-en-1-yl)thiomorpholine 1,1-

dioxide

Prepared following general procedure A the reaction mixture was purified by column chromatography using 40% EtOAc in hexane to provide the title compound in 40% yield (16.5 mg) as a yellow oil.

R$_f$ = 0.5 (50% EtOAc in hexane)

^{1}H NMR (600 MHz, $CDCl_3$) δ 7.72 (d, J = 7.8 Hz, 1H), 7.69 – 7.63 (m, 2H), 7.58 (t, J = 7.8 Hz, 1H), 5.92 – 5.81 (m, 2H), 3.30 – 3.20 (m, 1H), 3.05 – 2.93 (m, 6H), 2.88 – 2.78 (m, 3H), 1.81 – 1.73 (m, 1H), 1.70 – 1.62 (m, 1H), 1.56 – 1.40 (m, 2H). **^{13}C NMR** (151 MHz, $CDCl_3$) δ 136.97 (t, J = 27.4 Hz), 133.84, 130.91 (d, J = 33.0 Hz), 129.07, 128.95 (t, J = 6.1 Hz), 126.91 – 126.52 (m), 126.52 – 126.22 (m), 123.67 (q, J = 272.5 Hz), 122.81 – 122.56 (m), 122.53 (t, J = 247.1 Hz), 59.14, 52.45, 47.05, 42.32 (t, J = 26.1 Hz), 20.35 (t, J = 3.3 Hz), 19.77. **^{19}F NMR** (565 MHz, $CDCl_3$) δ -62.71, -98.85 (dd, J = 246.3, 14.4 Hz), -100.09 (dd, J = 246.3, 14.4 Hz).

HRMS (ESI) for $C_{18}H_{20}F_5NO_2S$ $[M+H]^+$ m/z: calcd. 410.1208, found 410.1208.

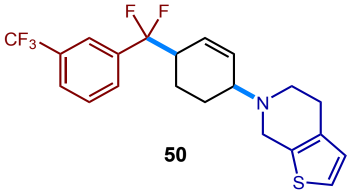

50

6-(4-(difluoro(3-(trifluoromethyl)phenyl)methyl)cyclohex-2-en-1-yl)-4,5,6,7-tetrahydrothieno[2,3-c]pyridine

Prepared following general procedure A the reaction mixture was purified by column chromatography using 10% EtOAc in hexane to provide the title compound in 70% yield (29 mg) as a colorless oil.

R$_f$ = 0.55 (25% EtOAc in hexane)

^{1}H NMR (400 MHz, $CDCl_3$) δ 7.78 – 7.68 (m, 3H), 7.60 (t, J = 7.7 Hz, 1H), 7.09 (d, J = 5.1 Hz, 1H), 6.72 (d, J = 5.1 Hz, 1H), 6.11 – 6.03 (m, 1H), 5.95 – 5.84 (m, 1H), 3.72 – 3.51 (m, 2H), 3.45 – 3.34 (m, 1H), 2.97 – 2.78 (m, 4H), 2.75 – 2.60 (m, 1H), 1.94 – 1.85 (m, 1H), 1.78 – 1.69 (m, 1H), 1.65 – 1.48 (m, 2H). **^{13}C NMR** (101 MHz, $CDCl_3$) δ 137.13 (t, J = 27.7 Hz), 134.12, 133.86, 133.34, 132.13, 130.83 (q, J = 32.8 Hz), 129.23 – 129.01 (m), 128.95, 126.63, 125.59 – 125.33 (m), 125.21, 123.75 (q, J = 272.4 Hz), 122.83, 122.73 (t, J = 247.4 Hz), 58.44, 48.68, 46.55, 42.48 (t, J = 26.0 Hz), 26.12, 20.61 (t, J = 3.4 Hz), 19.59. **^{19}F NMR** (377 MHz, $CDCl_3$) δ -62.63, -98.21 (dd, J = 245.6, 14.0 Hz), -100.19 (dd, J = 245.6, 14.6 Hz).

HRMS (ESI) for $C_{21}H_{20}F_5NS$ $[M+H]^+$ m/z: calcd. 414.1312, found 414.1309.

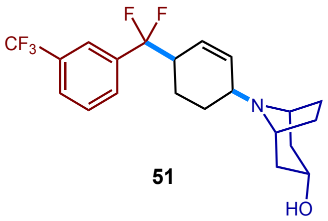

51

8-(4-(difluoro(3-(trifluoromethyl)phenyl)methyl)cyclohex-2-en-1-yl)-8-azabicyclo[3.2.1]octan-3-ol

Prepared following general procedure A the reaction mixture was purified by column chromatography using 10% MeOH in DCM to provide the title compound in 52% yield (21 mg) as a yellow oil.

R_f = 0.4 (10% MeOH in DCM)

^{1}H NMR (600 MHz, $CDCl_3$) δ 7.73 – 7.64 (m, 3H), 7.54 (t, *J* = 7.8 Hz, 1H), 6.03 – 5.89 (m, 1H), 5.79 (d, *J* = 10.5 Hz, 1H), 4.08 – 3.91 (m, 1H), 3.52 – 3.19 (m, 2H), 3.04 – 2.73 (m, 2H), 2.49 – 2.09 (m, 3H), 2.04 – 1.92 (m, 2H), 1.86 – 1.75 (m, 2H), 1.74 – 1.59 (m, 3H), 1.57 – 1.41 (m, 2H). **^{13}C NMR** (151 MHz, $CDCl_3$) δ 136.93 (t, *J* = 27.4 Hz), 130.61 (q, *J* = 32.7 Hz), 129.28 (t, *J* = 6.5 Hz), 128.76, 126.47, 126.24 – 125.66 (m), 123.78 (q, *J* = 272.4 Hz), 122.74 – 122.51 (m), 122.68 (t, *J* = 247.2 Hz), 64.60, 56.80, 55.92, 51.99, 43.66 (t, *J* = 25.9 Hz), 38.74, 38.56, 27.01, 26.48, 25.40, 18.71. **^{19}F NMR** (565 MHz, $CDCl_3$) δ -62.68, -99.30 (dd, *J* = 245.5, 14.5 Hz), -100.95 (dd, *J* = 245.6, 14.6 Hz).

HRMS (ESI) for $C_{21}H_{24}F_5NO$ $[M+H]^+$ m/z: calcd. 402.1851, found 402.1856.

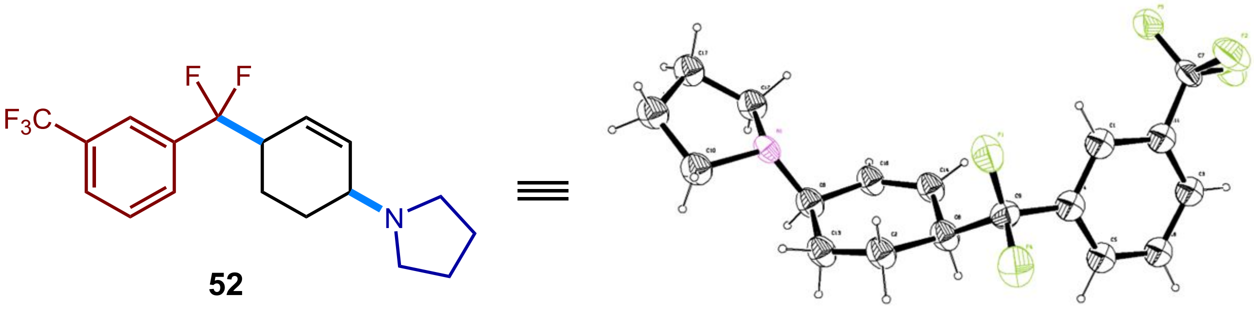

52

1-(4-(difluoro(3-(trifluoromethyl)phenyl)methyl)cyclohex-2-en-1-yl)pyrrolidine

Prepared following general procedure A the reaction mixture was purified by column chromatography using 10% MeOH in DCM to provide the title compound in 55% yield (19 mg) as a white solid.

R_f = 0.4 (10% MeOH in DCM)

^{1}H NMR (400 MHz, $CDCl_3$) δ 7.72 (d, *J* = 7.6 Hz, 1H), 7.65 (d, *J* = 10.2 Hz, 2H), 7.59 (t, *J* =

7.7 Hz, 1H), 6.12 – 6.01 (m, 2H), 3.86 – 3.71 (m, 1H), 3.07 (t, J = 8.5 Hz, 4H), 2.97 – 2.81 (m, 1H), 2.09 – 1.96 (m, 5H), 1.92 (ddd, J = 10.5, 7.0, 4.1 Hz, 1H), 1.83 – 1.64 (m, 2H). **^{13}C NMR** (101 MHz, $CDCl_3$) δ 136.55 (t, J = 27.3 Hz), 131.10 (q, J = 32.9 Hz), 130.59 – 130.18 (m), 129.37, 129.09 – 128.76 (m), 127.04, 126.20, 123.52 (q, J = 272.5 Hz), 122.42 (td, J = 6.6, 3.8 Hz), 122.20 (t, J = 247.6 Hz), 57.07, 49.50, 42.05 (t, J = 26.3 Hz), 24.06, 21.65, 19.51 (t, J = 3.1 Hz). **^{19}F NMR** (377 MHz, $CDCl_3$) δ -62.72, -98.72 – -100.29 (m).

HRMS (ESI) for $C_{18}H_{20}F_5N$ $[M+H]^+$ m/z: calcd.346.1589, found 346.1590.

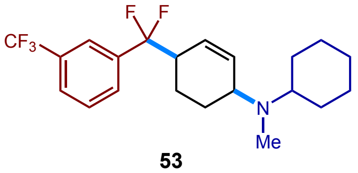

53

N-cyclohexyl-4-(difluoro(3-(trifluoromethyl)phenyl)methyl)-N-methylcyclohex-2-en-1-amine

Prepared following general procedure A the reaction mixture was purified by column chromatography using 18% EtOAc in hexane to provide the title compound in 72% yield (28 mg) as a colorless oil.

R_f = 0.1 (50% EtOAc in hexane)

^{1}H NMR (600 MHz, $CDCl_3$) δ 7.73 – 7.66 (m, 2H), 7.65 (d, J = 7.9 Hz, 1H), 7.55 (t, J = 7.8 Hz, 1H), 6.00 – 5.84 (m, 1H), 5.78 – 5.66 (m, 1H), 3.45 – 3.24 (m, 1H), 2.86 – 2.74 (m, 1H), 2.37 – 2.25 (m, 1H), 2.07 (s, 3H), 1.87 – 1.76 (m, 2H), 1.77 – 1.69 (m, 3H), 1.69 – 1.60 (m, 1H), 1.61 – 1.54 (m, 1H), 1.47 – 1.35 (m, 2H), 1.23 – 1.12 (m, 4H), 1.11 – 1.02 (m, 1H). **^{13}C NMR** (151 MHz, $CDCl_3$) δ 137.21 (t, J = 27.7 Hz), 136.47, 130.75 (q, J = 32.8 Hz), 129.13 (t, J = 6.1 Hz), 128.78, 126.58 – 126.29 (m), 124.10 (t, J = 5.0 Hz), 123.72 (d, J = 273.0 Hz), 122.78 – 122.66 (m), 122.82 (t, J = 246.1 Hz), 60.04, 54.57, 42.45 (t, J = 26.0 Hz), 33.43, 30.92, 30.85, 26.09, 25.89, 25.83, 21.48, 20.63 (t, J = 3.3 Hz). **^{19}F NMR** (565 MHz, $CDCl_3$) δ -62.72, -97.86 (dd, J = 245.1, 13.8 Hz), -100.19 (dd, J = 245.0, 14.8 Hz).

HRMS (ESI) for $C_{21}H_{26}F_5N$ $[M+H]^+$ m/z: calcd. 388.2058, found 388.2058.

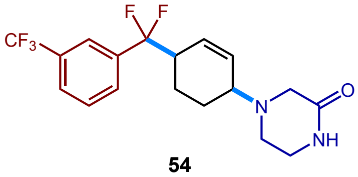

54

4-(4-(difluoro(3-(trifluoromethyl)phenyl)methyl)cyclohex-2-en-1-yl)piperazin-2-one

Prepared following general procedure A the reaction mixture was purified by column chromatography using 2% MeOH in DCM to provide the title compound in 80% yield (30 mg)

as a colorless oil.

R$_f$ = 0.5 (10% MeOH in DCM)

^{1}H NMR (400 MHz, $CDCl_3$) δ 7.72 – 7.64 (m, 2H), 7.64 – 7.59 (m, 1H), 7.57 – 7.49 (m, 1H), 7.20 (s, 1H), 5.96 – 5.73 (m, 2H), 3.36 – 3.06 (m, 5H), 2.90 – 2.73 (m, 1H), 2.67 – 2.57 (m, 1H), 2.53 – 2.40 (m, 1H), 1.85 – 1.72 (m, 1H), 1.69 – 1.57 (m, 1H), 1.57 – 1.39 (m, 2H). **^{13}C NMR** (151 MHz, $CDCl_3$) δ 170.37, 137.05 (t, *J* = 27.5 Hz), 132.74, 130.80 (q, *J* = 32.9 Hz), 129.18 – 129.03 (m), 128.99, 126.79 – 126.53 (m), 126.16 (t, *J* = 4.9 Hz), 123.70 (q, *J* = 272.4 Hz), 122.70 – 122.29 (m), 122.58 (d, *J* = 247.1 Hz), 57.09, 53.43, 44.69, 42.67 (t, *J* = 26.0 Hz), 41.66, 20.06 (t, *J* = 3.2 Hz), 19.74. **^{19}F NMR** (377 MHz, $CDCl_3$) δ -62.73, -99.22 (dd, *J* = 246.0, 14.5 Hz), -100.67 (dd, *J* = 245.7, 15.0 Hz).

HRMS (ESI) for $C_{18}H_{19}F_5N_2O$ $[M+H]^+$ m/z: calcd. 375.1490, found 375.1494.

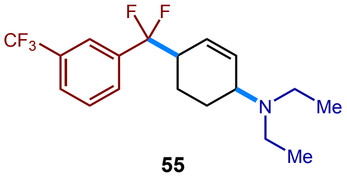

55

4-(difluoro(3-(trifluoromethyl)phenyl)methyl)-N,N-diethylcyclohex-2-en-1-amine

Prepared following general procedure A the reaction mixture was purified by column chromatography using 5% MeOH in DCM to provide the title compound in 72% yield (25 mg) as a yellow oil.

R$_f$ = 0.2 (50% EtOAc in hexane)

^{1}H NMR (400 MHz, $CDCl_3$) δ 7.72 (d, *J* = 7.6 Hz, 1H), 7.70 – 7.63 (m, 2H), 7.59 (t, *J* = 7.7 Hz, 1H), 6.24 (d, *J* = 10.5 Hz, 1H), 5.93 (dt, *J* = 10.6, 3.1 Hz, 1H), 3.96 – 3.78 (m, 1H), 3.01 – 2.80 (m, 3H), 2.73 – 2.58 (m, 2H), 1.99 – 1.92 (m, 1H), 1.84 – 1.68 (m, 2H), 1.61 – 1.51 (m, 1H), 1.33 (t, *J* = 7.3 Hz, 6H). **^{13}C NMR** (151 MHz, $CDCl_3$) δ 136.77 (t, *J* = 27.8 Hz), 131.07 (q, *J* = 33.1 Hz), 129.23, 128.93 (t, *J* = 6.0 Hz), 128.18 – 127.91 (m), 126.93, 123.61 (q, *J* = 272.6 Hz), 123.27 (d, *J* = 247.0 Hz), 122.66 – 122.43 (m), 56.13, 44.97, 41.65 (t, *J* = 26.3 Hz), 20.17, 18.60, 11.58. **^{19}F NMR** (565 MHz, $CDCl_3$) δ -62.73, -98.24 (d, *J* = 244.0 Hz), -99.75 (dd, *J* = 246.2, 15.5 Hz).

HRMS (ESI) for $C_{18}H_{22}F_5N$ $[M+H]^+$ m/z: calcd. 348.1745, found 348.1746

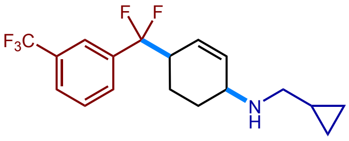

56

N-(cyclopropylmethyl)-4-(difluoro(3-(trifluoromethyl)phenyl)methyl)cyclohex-2-en-1-

amine

Prepared following general procedure A the reaction mixture was purified by column chromatography using 3% MeOH in DCM to provide the title compound in 36% yield (12.5 mg) as a yellow oil.

R_f = 0.5 (50% EtOAc in hexane)

^{1}H NMR (600 MHz, $CDCl_3$) δ 9.72 (s, 1H), 7.71 (d, J = 7.9 Hz, 1H), 7.70 – 7.63 (m, 2H), 7.58 (t, J = 7.8 Hz, 1H), 6.23 (dt, J = 10.4, 2.7 Hz, 1H), 6.05 (dt, J = 10.4, 2.6 Hz, 1H), 4.04 – 3.89 (m, 1H), 2.94 – 2.79 (m, 1H), 2.78 – 2.70 (m, 1H), 2.69 – 2.59 (m, 1H), 2.04 – 1.87 (m, 3H), 1.71 – 1.62 (m, 1H), 1.32 – 1.20 (m, 1H), 0.74 – 0.59 (m, 2H), 0.49 – 0.34 (m, 2H). **^{13}C NMR** (151 MHz, $CDCl_3$) δ 136.68 (t, J = 27.1 Hz), 131.11 (q, J = 32.5 Hz), 129.81, 129.25, 129.03 (t, J = 5.7 Hz), 127.03, 126.93, 123.61 (q, J = 272.4 Hz), 122.57 – 122.23 (m), 122.11 (t, J = 247.5 Hz), 50.91, 48.24, 42.43 (t, J = 26.3 Hz), 21.75, 19.26, 7.59, 4.84, 4.73. **^{19}F NMR** (565 MHz, $CDCl_3$) δ -62.69, -99.64 (dd, J = 247.3, 14.4 Hz), -100.99 (dd, J = 247.3, 16.3 Hz).

HRMS (ESI) for $C_{18}H_{20}F_5N$ $[M+H]^+$ m/z: calcd. 346.1589, found 346.1588.

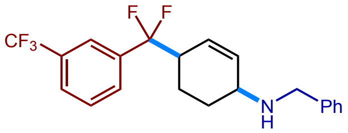

57

N-benzyl-4-(difluoro(3-(trifluoromethyl)phenyl)methyl)cyclohex-2-en-1-amine

Prepared following general procedure A the reaction mixture was purified by column chromatography using 15% EtOAc in hexane to provide the title compound in 60% yield (23 mg) as a colorless oil.

R_f = 0.5 (50% EtOAc in hexane)

^{1}H NMR (400 MHz, $CDCl_3$) δ 7.80 – 7.71 (m, 2H), 7.68 (d, J = 8.0 Hz, 1H), 7.59 (t, J = 7.8 Hz, 1H), 7.40 – 7.32 (m, 4H), 7.30 – 7.24 (m, 1H), 6.13 – 5.92 (m, 1H), 5.84 – 5.66 (m, 1H), 3.89 – 3.73 (m, 2H), 3.24 – 3.08 (m, 1H), 3.02 – 2.69 (m, 1H), 1.83 – 1.69 (m, 2H), 1.69 – 1.57 (m, 2H). **^{13}C NMR** (101 MHz, $CDCl_3$) δ 140.35, 137.14 (t, J = 27.6 Hz), 133.83, 130.80 (q, J = 33.3 Hz), 129.16 – 128.84 (m), 128.89, 128.37, 128.04, 126.93, 126.57, 124.18 (t, J = 5.0 Hz), 123.71 (q, J = 272.4 Hz), 122.58 (t, J = 246.7 Hz), 122.71 – 122.44 (m), 51.03, 50.40, 43.78 (t, J = 25.9 Hz), 26.06, 18.75 (t, J = 3.5 Hz). **^{19}F NMR** (377 MHz, $CDCl_3$) δ -67.42, -105.84 (dd, J = 245.0, 15.0 Hz), -105.98 (dd, J = 245.0, 15.0 Hz).

HRMS (ESI) for $C_{21}H_{20}F_5N$ $[M+H]^+$ m/z: calcd. 382.1589, found 382.1589.

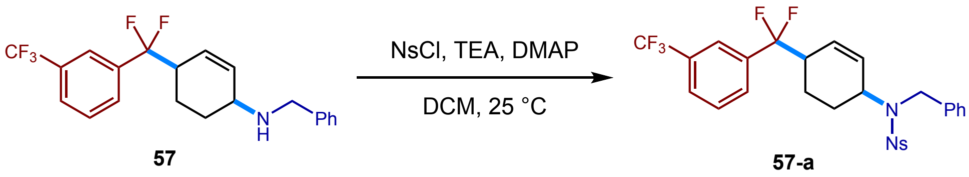

To a mixture of N-benzyl-4-(difluoro(3-(trifluoromethyl)phenyl)methyl) cyclo-hex-2-en-1-amine **57** (76.0 mg, 0.2 mmol), TEA (606 mg, 0.6 mmol), DMAP (12.0 mg, 0.1 mmol) in DCM (5 mL) at 25 °C was added NsCl (88.0 mg, 0.4 mmol) in DCM (1 mL). The reaction mixture was stirred at 25 °C for 24 h and TLC showed the reaction was complete. Then the mixture was poured into water (10 mL). The aqueous phase was extracted with DCM (3*10 mL) and the combined organic phases were washed with brine (3*10 mL), dried over Na_2SO_4, filtered and concentrated under reduced pressure to give a residue, which was purified by column chromatography (petroleum ether/ethyl acetate = 100:1) to afford the product **57-a** as white solid (102 mg, 90% yield).

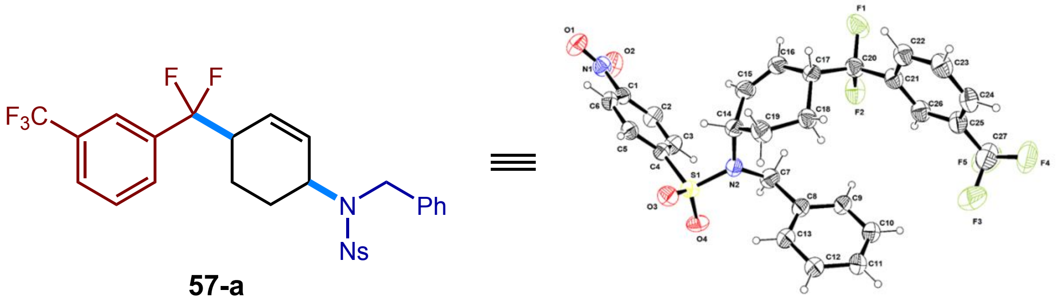

N-benzyl-N-(4-(difluoro(3-(trifluoromethyl)phenyl)methyl)cyclohex-2-en-1-yl)-4-nitrobenzenesulfonamide

^{1}H NMR (400 MHz, $CDCl_3$) δ 8.33 – 8.24 (m, 2H), 7.93 – 7.84 (m, 2H), 7.68 – 7.62 (m, 1H), 7.60 (s, 1H), 7.56 – 7.48 (m, 2H), 7.35 – 7.19 (m, 5H), 5.85 – 5.64 (m, 1H), 5.54 – 5.38 (m, 1H), 4.67 – 4.58 (m, 1H), 4.48 (d, J = 16.0 Hz, 1H), 4.17 (d, J = 16.0 Hz, 1H), 2.87 – 2.65 (m, 1H), 1.71 – 1.57 (m, 4H). **^{13}C NMR** (101 MHz, $CDCl_3$) δ 149.71, 146.78, 137.07, 136.70 (t, J = 27.4 Hz), 131.38, 130.85 (q, J = 32.8 Hz), 129.01, 128.72 (t, J = 6.0 Hz), 128.30, 128.04, 127.96 (d, J = 4.8 Hz), 127.78, 127.52, 126.87 – 126.55 (m), 124.22, 123.52 (q, J = 272.4 Hz), 122.48 – 122.11 (m), 122.26 (t, J = 247.6 Hz), 54.32, 48.13, 41.37 (t, J = 26.3 Hz), 25.28, 20.15 (t, J = 3.1 Hz). **^{19}F NMR** (377 MHz, $CDCl_3$) δ -62.69, -99.43 – -99.58 (m).

HRMS (ESI) for $C_{27}H_{23}F_5N_2O_4S$ $[M+Na]^+$ m/z: calcd. 589.1191, found 589.1186.

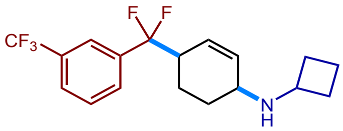

58

N-cyclobutyl-4-(difluoro(3-(trifluoromethyl)phenyl)methyl)cyclohex-2-en-1-amine

Prepared following general procedure A the reaction mixture was purified by column chromatography using 30% EtOAc in hexane to provide the title compound in 46% yield (16 mg) as a colorless oil.

R$_f$ = 0.5 (50% EtOAc in hexane)

^{1}H NMR (400 MHz, $CDCl_3$) δ 7.70 (d, J = 6.8 Hz, 2H), 7.64 (d, J = 7.8 Hz, 1H), 7.56 (t, J = 7.9 Hz, 1H), 6.02 – 5.86 (m, 1H), 5.77 – 5.65 (m, 1H), 3.38 – 3.20 (m, 1H), 3.14 – 3.03 (m, 1H), 2.89 – 2.71 (m, 1H), 2.28 – 2.11 (m, 2H), 1.72 – 1.62 (m, 4H), 1.61 – 1.51 (m, 4H). **^{13}C NMR** (151 MHz, $CDCl_3$) δ 137.21 (t, J = 27.5 Hz), 134.48, 130.85 (q, J = 32.7 Hz), 129.04 (t, J = 5.8 Hz), 128.92, 126.52 (d, J = 21.8 Hz), 123.80, 123.74 (q, J = 272.4 Hz), 122.60, 122.59 (t, J = 246.1 Hz), 52.67, 50.16, 43.64 (t, J = 25.9 Hz), 32.69, 32.53, 27.13, 18.84 (t, J = 3.5 Hz), 14.85. **^{19}F NMR** (565 MHz, $CDCl_3$) δ -62.70, -100.92 (dd, J = 245.6, 14.4 Hz), -101.49 (dd, J = 245.5, 15.0 Hz).

HRMS (ESI) for $C_{18}H_{20}F_5N$ $[M+H]^+$ m/z: calcd. 346.1589, found 346.1589.

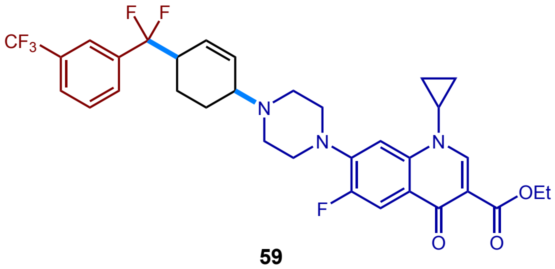

59

ethyl 1-cyclopropyl-7-(4-(4-(difluoro(3-(trifluoromethyl)phenyl)methyl)cyclohex-2-en-1-yl)piperazin-1-yl)-6-fluoro-4-oxo-1,4-dihydroquinoline-3-carboxylate

Prepared following general procedure A the reaction mixture was purified by column chromatography using 10% MeOH in DCM to provide the title compound in 66% yield (42 mg) as a yellow oil.

R$_f$ = 0.4 (50% EtOAc in hexane)

^{1}H NMR (400 MHz, $CDCl_3$) δ 8.49 (s, 1H), 8.02 – 7.94 (m, 1H), 7.73 – 7.63 (m, 3H), 7.57 (t, J = 8.0 Hz, 1H), 7.22 (d, J = 7.1 Hz, 1H), 5.96 (dt, J = 10.3, 2.5 Hz, 1H), 5.85 (ddd, J = 10.4, 3.3, 2.1 Hz, 1H), 4.36 (q, J = 7.1 Hz, 2H), 3.41 (dt, J = 7.0, 3.2 Hz, 1H), 3.30 – 3.12 (m, 5H), 2.91 – 2.80 (m, 1H), 2.76 – 2.65 (m, 2H), 2.64 – 2.52 (m, 2H), 1.87 – 1.76 (m, 1H), 1.72 – 1.62 (m, 1H), 1.61 – 1.46 (m, 2H), 1.39 (t, J = 7.1 Hz, 3H), 1.33 – 1.27 (m, 2H), 1.15 – 1.09 (m,

2H). **^{13}C NMR** (101 MHz, $CDCl_3$) δ 173.19 – 173.02 (m), 165.87, 153.39 (d, *J* = 248.5 Hz), 148.13, 144.61 (d, *J* = 10.6 Hz), 137.98, 137.08 (t, *J* = 27.6 Hz), 133.79, 130.75 (q, *J* = 32.7 Hz), 129.21 – 129.01 (m), 128.98, 126.94 – 126.11 (m), 125.99 – 125.31 (m), 123.75 (q, *J* = 272.5 Hz), 122.97 (d, *J* = 7.0 Hz), 122.88 – 122.62 (m), 122.69 (t, *J* = 247.0 Hz), 113.18 (d, *J* = 23.1 Hz), 110.34, 104.79 (d, *J* = 3.1 Hz), 60.87, 58.36, 50.38 (d, *J* = 4.4 Hz), 48.61, 42.68 (t, *J* = 26.1 Hz), 34.48, 20.41, 19.76, 14.46, 8.14. **^{19}F NMR** (377 MHz, $CDCl_3$) δ -62.66, -98.67 (dd, *J* = 245.8, 14.1 Hz), -100.46 (dd, *J* = 245.8, 14.4 Hz), -123.65 (dd, *J* = 13.3, 7.2 Hz).
HRMS (ESI) for $C_{33}H_{33}F_6N_3O_3$ $[M+Na]^+$ m/z: calcd. 656.2318, found 656.2318.

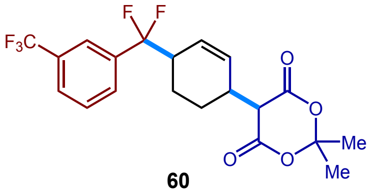

60

5-(4-(difluoro(3-(trifluoromethyl)phenyl)methyl)cyclohex-2-en-1-yl)-2,2-dimethyl-1,3-dioxane-4,6-dione

Prepared following general procedure A (**NOTE**: one additional equivalent base was used) the reaction mixture was purified by column chromatography using 15% EtOAc in hexane to provide the title compound in 71% yield (30 mg) as a yellow oil.

R_f = 0.8 (50% EtOAc in hexane)

^{1}H NMR (600 MHz, $CDCl_3$) δ 7.73 – 7.70 (m, 1H), 7.69 (d, *J* = 7.9 Hz, 1H), 7.65 (d, *J* = 7.9 Hz, 1H), 7.56 (t, *J* = 7.8 Hz, 1H), 5.85 (dt, *J* = 10.4, 2.3 Hz, 1H), 5.72 – 5.59 (m, 1H), 3.50 (d, *J* = 3.7 Hz, 1H), 3.20 – 3.05 (m, 1H), 2.91 – 2.75 (m, 1H), 2.09 – 1.98 (m, 1H), 1.74 (s, 3H), 1.73 (s, 3H), 1.72 – 1.68 (m, 1H), 1.62 – 1.57 (m, 2H). **^{13}C NMR** (151 MHz, $CDCl_3$) δ 164.43, 164.04, 137.04 (t, *J* = 27.6 Hz), 132.72, 130.75 (q, *J* = 32.8 Hz), 129.36 (d, *J* = 6.2 Hz), 128.93, 126.79 – 126.36 (m), 123.79 (q, *J* = 272.4 Hz), 123.64 (t, *J* = 5.1 Hz), 122.60 – 122.36 (m), 122.48 (t, *J* = 247.3 Hz), 104.94, 49.79, 41.51 (t, *J* = 26.4 Hz), 35.60, 28.22, 27.52, 22.03, 21.31 – 20.81 (m). **^{19}F NMR** (565 MHz, $CDCl_3$) δ -62.65, -97.44 (dd, *J* = 244.2, 13.2 Hz), -101.09 (dd, *J* = 244.3, 17.2 Hz).
HRMS (ESI) for $C_{20}H_{19}F_5O_4$ $[M+H]^+$ m/z: calcd. 419.1276, found 419.1280.

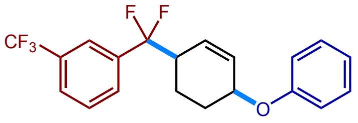

61

1-(difluoro(4-phenoxycyclohex-2-en-1-yl)methyl)-3-(trifluoromethyl)benzene

Prepared following general procedure A the reaction mixture was purified by column

chromatography using 10% EtOAc in hexane to provide the title compound in 34% yield (13 mg) as a yellow oil.

R_f=0.40 (10% EtOAc in hexane).

^{1}H NMR (600 MHz, $CDCl_3$) δ 7.75 (s, 1H), 7.72 (d, *J* = 7.9 Hz, 1H), 7.68 (d, *J* = 7.9 Hz, 1H), 7.58 (t, *J* = 7.8 Hz, 1H), 7.29 – 7.25 (m, 2H), 6.94 (tt, *J* = 7.3, 1.1 Hz, 1H), 6.90 – 6.87 (m, 2H), 6.13 (dt, *J* = 10.3, 3.3 Hz, 1H), 6.03 – 5.98 (m, 1H), 4.72 (q, *J* = 3.8 Hz, 1H), 2.93 – 2.82 (m, 1H), 2.08 – 2.01 (m, 1H), 1.88 – 1.79 (m, 1H), 1.77 – 1.68 (m, 1H), 1.67 – 1.59 (m, 1H). **^{13}C NMR** (151 MHz, CDCl3) δ 157.55, 136.98 (t, *J* = 27.3 Hz), 130.99 (q, *J* = 32.7 Hz), 129.84, 129.53, 129.09, 129.03, 127.62 (t, *J* = 5.0 Hz), 126.75, 123.73 (q, *J* = 272.4), 122.62 (td, *J* = 6.6, 3.5 Hz), 122.39 (t, *J* = 246.9 Hz), 121.00, 116.00, 68.60, 44.22 (t, *J* = 26.2 Hz), 26.27, 18.28 (t, *J* = 3.6 Hz). **^{19}F NMR** (565 MHz, $CDCl_3$) δ -62.70, -100.66 (dd, *J* = 247.1, 14.1 Hz), -101.98 (dd, *J* = 247.2, 15.6 Hz).

HRMS (ESI) for $C_{20}H_{17}F_5O$ $[M+H]^+$ m/z: calcd. 369.1273, found 369.1287.

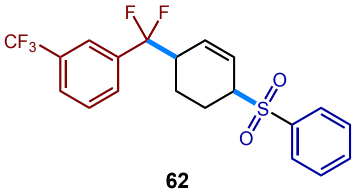

62

1-(difluoro(4-(phenylsulfonyl)cyclohex-2-en-1-yl)methyl)-3-(trifluoromethyl)benzene

Prepared following general procedure A the reaction mixture was purified by column chromatography using 10% EtOAc in hexane to provide the title compound in 50% yield (21 mg) as a colorless oil.

R_f= 0.5 (25% EtOAc in hexane)

^{1}H NMR (400 MHz, $CDCl_3$) δ 7.91 – 7.82 (m, 2H), 7.74 – 7.65 (m, 2H), 7.61 – 7.50 (m, 5H), 6.11 – 6.01 (m, 2H), 3.85 – 3.67 (m, 1H), 2.94 – 2.69 (m, 1H), 2.32 – 2.16 (m, 1H), 1.97 – 1.80 (m, 1H), 1.57 – 1.45 (m, 2H). **^{13}C NMR** (101 MHz, $CDCl_3$) δ 136.86, 136.45 (t, *J* = 27.3 Hz), 133.85, 130.89 (q, *J* = 32.8 Hz), 130.26 (t, *J* = 4.9 Hz), 129.22, 129.05, 128.99, 128.89 – 128.69 (m), 126.99 – 126.58 (m), 123.63 (q, *J* = 272.2 Hz), 123.23, 122.67 – 122.02 (m), 121.86 (t, *J* = 247.4 Hz), 60.46, 42.98 (t, *J* = 26.6 Hz), 20.60, 18.66 (t, *J* = 3.6 Hz). **^{19}F NMR** (377 MHz, $CDCl_3$) δ -62.70, -100.81 (dd, *J* = 246.9, 14.4 Hz), -101.82 (dd, *J* = 246.8, 15.1 Hz).

HRMS (ESI) for $C_{20}H_{17}F_5O_2S$ $[M+Na]^+$ m/z: calcd. 439.0762, found 439.0766.

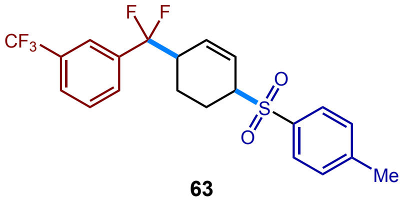

63

1-(difluoro(4-tosylcyclohex-2-en-1-yl)methyl)-3-(trifluoromethyl)benzene

Prepared following general procedure A the reaction mixture was purified by column chromatography using 8% EtOAc in hexane to provide the title compound in 65% yield (28 mg) as a yellow oil.

R_f = 0.5 (30% EtOAc in hexane)

^{1}H NMR (400 MHz, $CDCl_3$) δ 7.73 – 7.64 (m, 3H), 7.59 – 7.47 (m, 3H), 7.33 (d, J = 8.0 Hz, 2H), 6.12 – 5.95 (m, 2H), 3.75 – 3.60 (m, 1H), 2.88 – 2.70 (m, 1H), 2.46 (s, 3H), 2.26 – 2.11 (m, 1H), 1.92 – 1.77 (m, 1H), 1.60 – 1.39 (m, 2H). **^{13}C NMR** (151 MHz, $CDCl_3$) δ 144.90, 136.57 (t, J = 27.4 Hz), 134.03, 130.91 (q, J = 32.8 Hz), 130.12 (t, J = 4.9 Hz), 129.66, 129.25, 129.06, 128.85 (t, J = 6.0 Hz), 126.77, 123.68 (q, J = 272.5 Hz), 123.41, 122.52 – 122.14 (m), 121.92 (t, J = 247.3 Hz), 60.47, 43.02 (t, J = 26.5 Hz), 21.63, 20.63, 18.74 (t, J = 3.3 Hz). **^{19}F NMR** (565 MHz, $CDCl_3$) δ -62.70, -100.99 (dd, J = 246.6, 14.5 Hz), -101.88 (dd, J = 247.1, 15.4 Hz).

HRMS (ESI) for $C_{21}H_{19}F_5O_2S$ $[M+Na]^+$ m/z: calcd. 453.0918, found 453.0918.

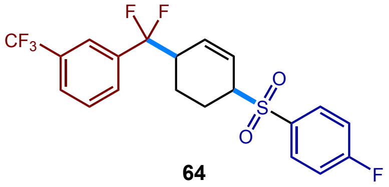

64

1-(difluoro(4-((4-fluorophenyl)sulfonyl)cyclohex-2-en-1-yl)methyl)-3-(trifluoromethyl)benzene

Prepared following general procedure A the reaction mixture was purified by column chromatography using 8% EtOAc in hexane to provide the title compound in 64% yield (28 mg) as a yellow oil.

R_f = 0.5 (30% EtOAc in hexane)

^{1}H NMR (600 MHz, $CDCl_3$) δ 7.86 – 7.78 (m, 2H), 7.73 – 7.66 (m, 1H), 7.58 – 7.51 (m, 2H), 7.52 – 7.46 (m, 1H), 7.23 – 7.16 (m, 2H), 6.08 – 6.02 (m, 1H), 6.03 – 5.97 (m, 1H), 3.74 – 3.65 (m, 1H), 2.89 – 2.75 (m, 1H), 2.25 – 2.11 (m, 1H), 1.94 – 1.83 (m, 1H), 1.58 – 1.40 (m, 2H). **^{13}C NMR** (151 MHz, $CDCl_3$) δ 165.93 (d, J = 256.9 Hz), 136.32 (t, J = 27.3 Hz), 132.90 (d, J = 3.1 Hz), 132.05 (d, J = 9.5 Hz), 130.93 (q, J = 32.8 Hz), 130.48 (t, J = 5.0 Hz), 129.06, 128.78 (t, J = 6.3 Hz), 127.01 – 126.67 (m), 123.61 (q, J = 272.5 Hz), 123.12, 122.46 – 122.15 (m), 121.87 (t, J = 247.3 Hz), 116.31 (d, J = 22.6 Hz), 60.63, 42.96 (t, J = 26.6 Hz), 20.60, 18.65 (t, J = 3.5 Hz). **^{19}F NMR** (565 MHz, $CDCl_3$) δ -62.74, -100.65 (dd, J = 247.3, 14.1 Hz), -101.58 (dd, J = 247.2, 14.7 Hz), -102.95 (td, J = 8.7, 4.4 Hz).

HRMS (ESI) for $C_{20}H_{16}F_6O_2S$ $[M+Na]^+$ m/z: calcd. 457.0667, found 457.0667.

VII. Synthesis of drug and its analog

7.1 Synthesis of bioactive molecules (66)

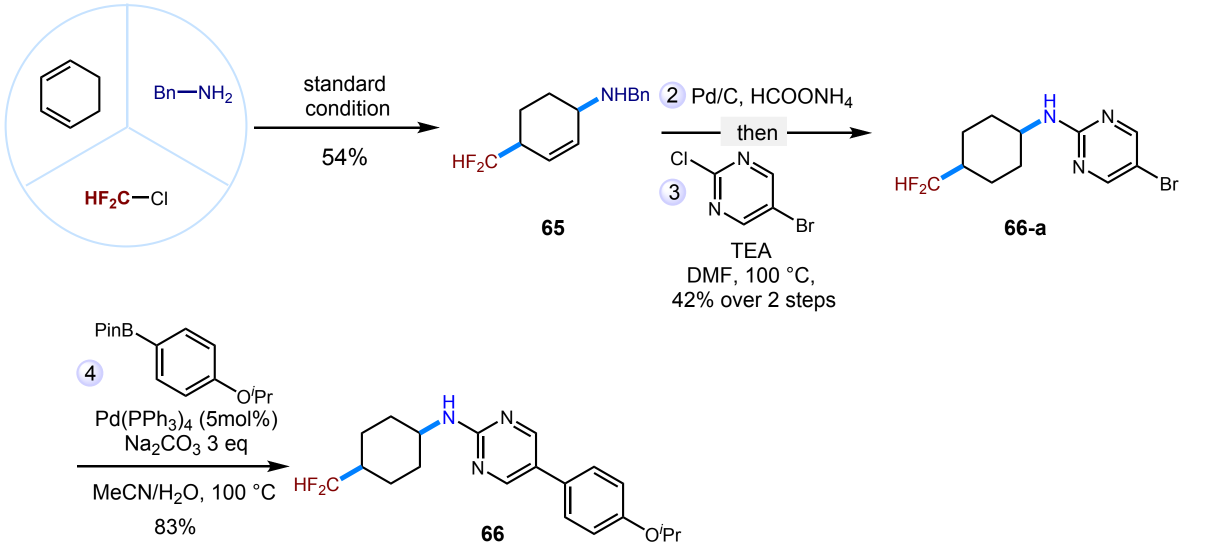

Step 1:

Prepared following general procedure D the reaction mixture was purified by column chromatography using 10% EtOAc in hexane to provide the compound of **65** in 54% yield (13 mg) as a yellow oil.

$\mathbf{R_f}$=0.30 (33% EtOAc in hexane).

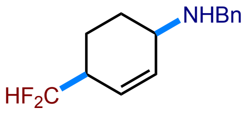

65

^{1}H NMR (600 MHz, $CDCl_3$) δ 7.39 – 7.29 (m, 4H), 7.28 – 7.21 (m, 1H), 6.06 – 5.95 (m, 1H), 5.79 – 5.45 (m, 2H), 3.91 – 3.76 (m, 2H), 3.23 – 3.11 (m, 1H), 2.57 – 2.42 (m, 1H), 1.82 – 1.67 (m, 4H), 1.61 – 1.45 (m, 1H). **^{13}C NMR** (151 MHz, $CDCl_3$) δ 140.43, 133.94, 128.48, 128.16, 127.03, 124.05 (dd, J = 7.2, 5.1 Hz), 118.08 (t, J = 242.9 Hz), 51.24, 51.04, 39.76 (dd, J = 20.4, 19.2 Hz), 26.14, 18.76 (dd, J = 5.4, 3.7 Hz). **^{19}F NMR** (565 MHz, $CDCl_3$) δ -119.84 (ddd, J = 278.0, 57.1, 13.3 Hz), -122.12 (ddd, J = 278.0, 57.0, 14.3 Hz).

HRMS (ESI) for $C_{14}H_{17}F_2N$ $[M+H]^+$ m/z: calcd. 238.1402, found 238.1401.

Step 2:

To a mixture of **65** (N-benzyl-4-(difluoromethyl)cyclohex-2-en-1-amine)(109 mg, 0.46 mmol) and ammonium formate (145 mg, 2.30 mmol) in MeOH (5 mL) was added Pd/C (48.3 mg, 0.046 mmol, 10 wt%) in a glovebox. The mixture was then sealed and stirred at 65 °C for 18 h. After the reaction completed, the mixture was filtered through celite and the filtrate was

concentrated in vacuum to give the crude product 4-(difluoromethyl)cyclohexan-1-amine, which was then dissolved in DMF (8 mL). To the above solution was added TEA (151 mg, 1.50 mmol) and 5-bromo-2-chloropyrimidine (117 mg, 0.46 mmol) and the mixture was then stirred at 100 °C for 8 h. After cooling to room temperature, the reaction mixture was diluted with EtOAc (30 mL), and washed with brine (5*10 mL). The organic layer was separated, dried over Na_2SO_4 and concentrated in vacuum. Then the resulting crude product, which was purified by silica gel chromatography (petroleum ether/ethyl acetate = 100/1) to afford 5-bromo-N-(4-(difluoromethyl) cyclohexyl) pyrimidin-2-amine (59.0 mg, 42% yield over 2 steps).

Step 3:

To a solution of 5-bromo-*N*-(4-(difluoromethyl)cyclohexyl)pyrimidin-2-amine (59.0 mg, 0.2 mmol), 4-(trifluoromethoxy) phenylboronic acid (105 mg, 0.4 mmol) and Na_2CO_3 (63.0 mg, 0.6 mmol) in CH_3CN (4 mL) and H_2O (1 mL) was added $Pd(PPh_3)_4$ (12 mg, 0.01 mmol). The mixture was stirred at 100 °C for 12 h. After cooling to room temperature, the reaction mixture was diluted with EtOAc (10 mL), and washed with brine (3*10 mL). The organic layer was separated, dried over Na_2SO_4 and concentrated in vacuum. Then the resulting crude product, which was purified by silica gel chromatography (petroleum ether/ethyl acetate = 10/1) to afford *N*-(4-(difluoromethyl)cyclohexyl)-5-(4-isopropoxyphenyl)pyrimidin-2-amine **66** (60.0 mg, 83%) as a white solid.

R$_f$ =0.10 (10% EtOAc in hexane).

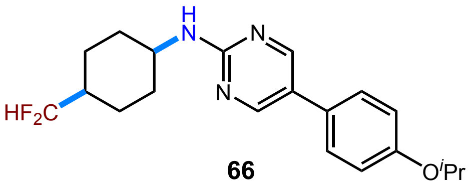

66

The spectral data of the substrates were consisted with that reported in the literature[5]

^{1}H NMR (600 MHz, $CDCl_3$) δ 8.46 (s, 2H), 7.43 – 7.33 (m, 2H), 7.03 – 6.91 (m, 2H), 5.62 (td, *J* = 56.8, 4.6 Hz, 1H), 5.39 (d, *J* = 7.5 Hz, 1H), 4.56 (p, *J* = 6.1 Hz, 1H), 4.19 (dt, *J* = 7.8, 3.9 Hz, 1H), 2.01 – 1.91 (m, 2H), 1.90 – 1.80 (m, 1H), 1.75 – 1.64 (m, 4H), 1.55 – 1.47 (m, 2H), 1.35 (d, *J* = 6.1 Hz, 6H). **^{13}C NMR** (101 MHz, $CDCl_3$) δ 160.57, 157.41, 155.84, 127.70, 126.97, 123.68, 118.56 (t, *J* = 241.7 Hz), 116.40, 69.94, 45.89, 40.21 (t, *J* = 19.6 Hz), 28.71, 21.97, 20.31 (t, *J* = 4.7 Hz). **^{19}F NMR** (377 MHz, $CDCl_3$) δ -122.84 (dd, *J* = 56.9, 13.8 Hz).

7.2 Synthesis of bioactive molecules (67)

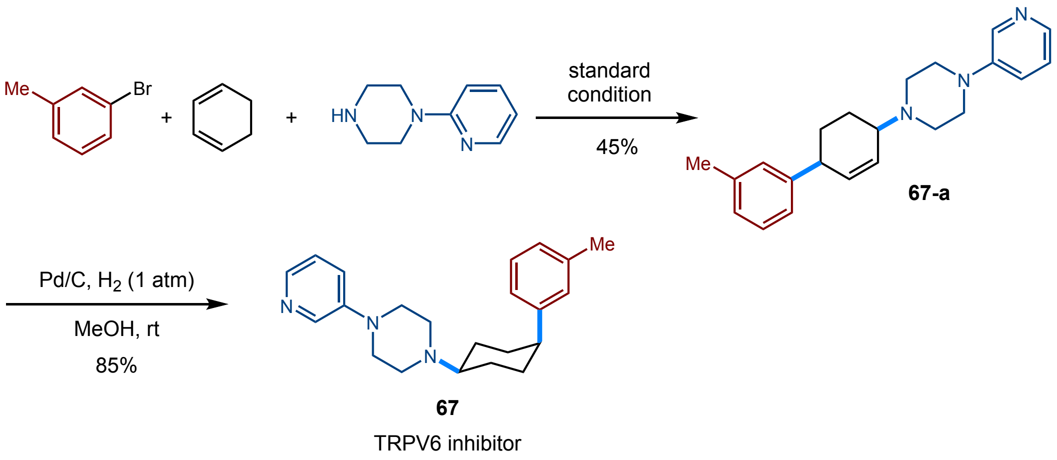

Step 1: Prepared following general procedure C the reaction mixture was purified by column chromatography using 40% EtOAc in hexane to provide the compound (15 mg) in 45% yield as a yellow oil.

R$_f$=0.5 (10% MeOH in DCM).

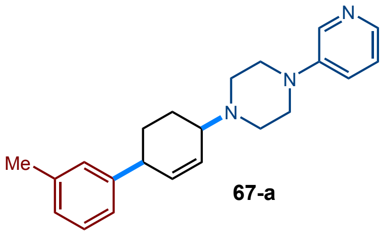

67-a

^{1}H NMR (400 MHz, $CDCl_3$) δ 8.40 – 8.27 (m, 1H), 8.09 (dd, J = 4.4, 1.7 Hz, 1H), 7.25 – 7.13 (m, 3H), 7.09 – 6.97 (m, 3H), 6.03 – 5.86 (m, 2H), 3.45 – 3.33 (m, 2H), 3.32 – 3.22 (m, 4H), 2.97 – 2.86 (m, 2H), 2.85 – 2.73 (m, 2H), 2.35 (s, 3H), 2.06 – 1.96 (m, 1H), 1.84 – 1.70 (m, 2H), 1.66 – 1.57 (m, 1H). ^{13}C NMR (151 MHz, $CDCl_3$) δ 147.00, 145.01, 140.62, 138.58, 137.91, 133.06, 129.83, 128.89, 128.20, 126.99, 125.02, 123.51, 122.41, 59.68, 48.83, 48.52, 40.40, 29.88, 21.55, 19.04.

HRMS (ESI) for $C_{22}H_{27}N_3$ $[M+H]^+$ m/z: calcd. 334.2278, found 334.2277.

Step 2: To a mixture of **67-a** (1-(3'-methyl-1,2,3,4-tetrahydro-[1,1'-biphenyl]-4-yl)-4-(pyridin-3-yl)piperazine) (15 mg, 0.45 mmol) in MeOH (2 mL) was added Pd/C (48.3 mg, 0.045 mmol, 10 wt%) in a glovebox. The mixture was then degassed and purged with H_2 three times and stirred under H_2 atmosphere at room temperature for 18 h. After the reaction completed, the mixture was filtered through celite and the filtrate was concentrated in vacuum to give the crude product, which was purified by column chromatography using 40% EtOAc in hexane to provide the compound (13 mg) in 85% yield as a white solid.

R$_f$=0.5 (10% MeOH in DCM).

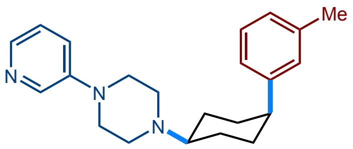

67

^{1}H NMR (600 MHz, $CDCl_3$) δ 8.33 (d, *J* = 2.8 Hz, 1H), 8.09 (dd, *J* = 4.5, 1.5 Hz, 1H), 7.22 – 7.13 (m, 3H), 7.10 – 7.05 (m, 2H), 7.00 (d, *J* = 7.4 Hz, 1H), 3.26 (t, *J* = 5.0 Hz, 4H), 2.74 – 2.62 (m, 5H), 2.39 – 2.31 (m, 4H), 2.04 – 1.93 (m, 4H), 1.66 – 1.56 (m, 4H).

^{13}C NMR (151 MHz, $CDCl_3$) δ 147.01, 146.97, 140.51, 138.39, 137.77, 128.20, 127.80, 126.55, 124.00, 123.47, 122.13, 58.81, 49.63, 48.73, 42.91, 29.73, 28.33, 21.58.

HRMS (ESI) for $C_{22}H_{29}N_3$ $[M+H]^+$ m/z: calcd. 336.2434, found 336.2431.

7.3 Synthesis of bioactive molecules analogs (68)

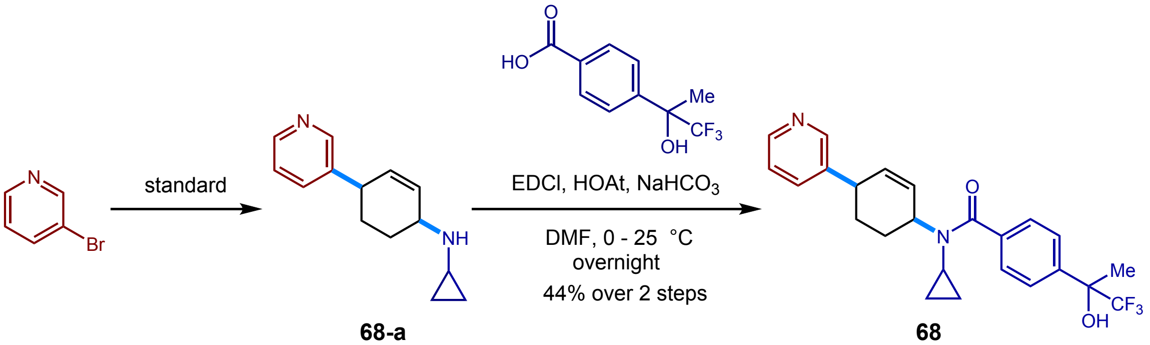

Step 1: Prepared following general procedure C the reaction mixture was purified by column chromatography using 5% MeOH in DCM to provide the crude compound (164 mg) as a yellow oil (7 reactions were conducted in parallel). The crude product was used directly in the next step without further purification.

Step 2: To a solution of crude N-cyclopropyl-4-(pyridin-3-yl)cyclohex-2-en-1-amine (164 mg, 0.7 mmol, 1.1 eq), $NaHCO_3$ (107 mg, 1.28 mmol, 2.0 eq) and 4-(1,1,1-trifluoro-2-hydroxypropan-2-yl)benzoic acid (150 mg, 0.64 mmol, 1.0 eq) in DMF (10 mL) was added EDCI (160 mg, 0.83 mmol, 1.3 eq) and HOAt (113 mg, 0.83 mmol, 1.3 eq) at 0 °C. After being stirred at 25 °C for 12 h, the reaction mixture was diluted with EtOAc (20 ml) and the organic phase was washed with brine (5×10 ml), dried over Na_2SO_4 and concentrated under reduced pressure to give a residue, which was purified by column chromatography (2% MeOH in DCM) to give the compound of **68** as a white solid (133 mg, 44% yield).

R_f=0.20 (100% EtOAc in hexane).

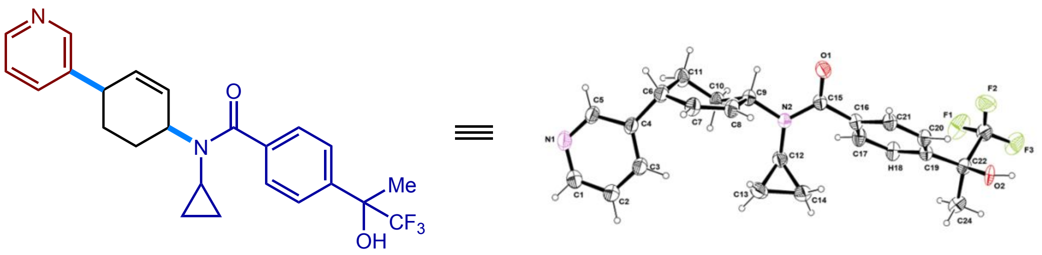

68

^{1}H NMR (600 MHz, DMSO-d_6) δ 8.66 (s, 1H), 8.54 (s, 1H), 7.86 (d, J = 7.9 Hz, 1H), 7.76 – 7.59 (m, 4H), 7.46 (dd, J = 7.9, 4.7 Hz, 1H), 6.83 (s, 1H), 6.07 (d, J = 10.2 Hz, 1H), 5.92 (d, J = 9.1 Hz, 1H), 5.09 – 4.60 (m, 1H), 3.69 – 3.61 (m, 1H), 3.02 – 2.81 (m, 1H), 2.22 (d, J = 14.4 Hz, 1H), 2.09 (q, J = 11.6 Hz, 1H), 1.95 – 1.83 (m, 2H), 1.81 (s, 3H), 0.84 – 0.29 (m, 4H). **^{13}C NMR** (151 MHz, DMSO-d_6) δ 171.83 – 171.01 (m), 149.44, 147.35, 140.73, 140.35, 137.88, 135.65, 132.02, 130.61, 128.79, 126.82, 126.03, 125.97 (q, J = 285.8 Hz), 123.40, 73.25 (q, J = 28.1 Hz), 54.50, 36.41, 29.76, 22.80, 22.72, 21.99, 10.02, 9.17. **^{19}F NMR** (565 MHz, DMSO-d_6) δ -79.73.

HRMS (ESI) for $C_{24}H_{25}F_3N_2O_2$ $[M+H]^+$ m/z: calcd. 431.1941, found 431.1940.

7.4 Synthesis of bioactive molecules analogs (69)

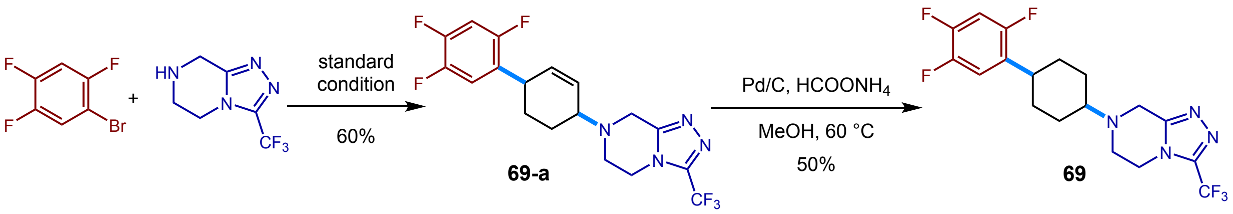

Step 1:

Prepared following general procedure C the reaction mixture was purified by column chromatography using 25% EtOAc in hexane to provide 7-(2',4',5'-trifluoro-1, 2,3,4-tetrahydro-[1,1'-biphenyl]-4-yl)-3-(trifluoromethyl)-5,6,7,8-tetrahydro-[1,2,4] triazolo [4,3-a]pyrazine in 60% yield (24 mg) as a yellow oil.

Rf = 0.7 (50% EtOAc in hexane)

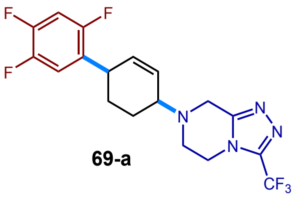

69-a

^{1}H NMR (400 MHz, $CDCl_3$) δ 7.06 – 6.82 (m, 2H), 6.06 – 5.93 (m, 1H), 5.93 – 5.80 (m, 1H), 4.26 – 4.11 (m, 2H), 4.05 (q, J = 15.3 Hz, 2H), 3.76 – 3.61 (m, 1H), 3.53 – 3.40 (m, 1H), 3.23

– 3.09 (m, 1H), 3.07 – 2.93 (m, 1H), 2.07 – 1.91 (m, 1H), 1.84 – 1.67 (m, 2H), 1.64 – 1.53 (m, 1H). **^{13}C NMR** (101 MHz, $CDCl_3$) δ 155.32 (ddd, *J* = 244.9, 9.1, 2.6 Hz), 152.54, 148.66 (ddd, *J* = 249.7, 14.4, 12.4 Hz), 146.40 (ddd, *J* = 244.4, 12.2, 3.5 Hz), 143.22 (q, *J* = 39.7 Hz), 132.02, 130.61, 127.95 – 127.39 (m), 118.41 (d, *J* = 270.2 Hz), 116.97 (dd, *J* = 19.5, 6.0 Hz), 105.54 (dd, *J* = 28.4, 20.8 Hz), 58.64, 45.57, 45.21, 44.14, 33.13, 27.28, 19.52. **^{19}F NMR** (377 MHz, $CDCl_3$) δ -63.20, -120.01 – -120.22 (m), -136.52 (dtd, *J* = 22.0, 9.6, 3.4 Hz), -142.42 – -142.72 (m).

HRMS (ESI) for $C_{18}H_{16}F_6N_4$ $[M+H]^+$ m/z: calcd. 403.1352, found 403.1352.

Step 2:

To the mixture of 7-(2',4',5'-trifluoro-1, 2,3,4-tetrahydro-[1,1'-biphenyl]-4-yl)-3-(trifluoromethyl) -5,6,7,8-tetrahydro-[1,2,4]triazolo[4,3-a]pyrazine (24 mg, 0.059 mmol) in MeOH (5 mL), Then was added ammonium formate (24.0 mg, 0.059 mmol) and Pd/C (42.0 mg, 0.0405 mmol, 10 wt%) in a glovebox. The mixture was then stirred at 65 °C for 18 h. After the reaction completed, the mixture was filtered through celite and the filtrate was concentrated in vacuum to give a residue, which was purified by column chromatography using 24% EtOAc in hexane to provide the title compound 3-(trifluoromethyl)-7-(4-(2,4,5-trifluorophenyl)cyclohexyl)-5,6,7,8-tetrahydro-[1,2,4] triazolo [4,3-a]pyrazine **69** (12 mg, 50% yield) as a colorless oil.

R_f = 0.7 (50% EtOAc in hexane)

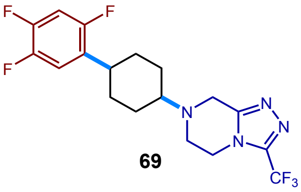

69

^{1}H NMR (400 MHz, $CDCl_3$) δ 7.10 – 6.94 (m, 1H), 6.91 – 6.73 (m, 1H), 4.15 (t, *J* = 5.5 Hz, 2H), 3.94 (s, 2H), 3.01 (t, *J* = 5.5 Hz, 2H), 2.99 – 2.83 (m, 1H), 2.66 – 2.54 (m, 1H), 2.22 – 2.04 (m, 2H), 1.84 – 1.65 (m, 4H), 1.64 – 1.55 (m, 2H). **^{13}C NMR** (101 MHz, $CDCl_3$) δ 155.16 (ddd, *J* = 243.5, 9.1, 2.6 Hz), 152.70, 149.55 – 146.42 (m), 146.73 (ddd, *J* = 243.4, 12.4, 3.6 Hz), 143.11 (q, *J* = 39.7 Hz), 129.99 – 129.49 (m), 118.42 (q, *J* = 270.2 Hz), 115.37 (dd, *J* = 19.4, 6.5 Hz), 105.12 (dd, *J* = 29.2, 20.5 Hz), 56.67, 46.65, 45.74, 43.62, 35.95, 28.47, 26.43. **^{19}F NMR** (377 MHz, $CDCl_3$) δ -63.09, -120.96 – -121.26 (m), -137.34 – -138.03 (m), -142.82 – -143.50 (m).

HRMS (ESI) for $C_{18}H_{18}F_6N_4$ $[M+H]^+$ m/z: calcd. 405.1508, found 405.1508.

7.5 Synthesis of functionalized 1-N-iminosugar (70)

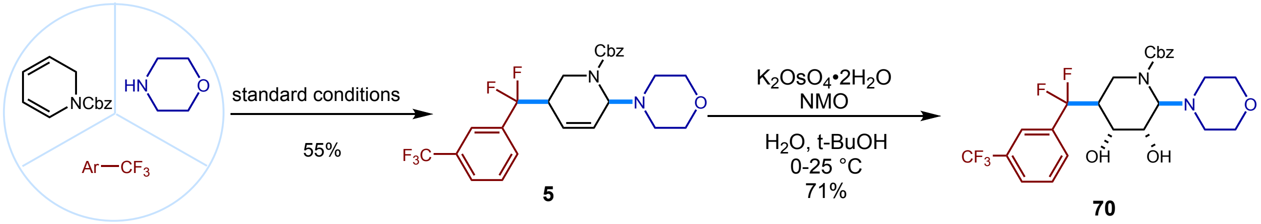

To the mixture of NMO (100 mg, 0.84 mmol) and benzyl-3-(difluoro(3-(trifluoromethyl)phenyl)methyl)-6-morpholino-3,6-dihydropyridine-1(2H)-carboxylate **5** (210 mg, 0.42 mmol) in t-BuOH (5 mL) and H_2O (5 mL) was added K_2OsO_4•$2H_2O$ (16.0 mg, 0.042 mmol) at 0 °C and the reaction mixture was stirred at 25 °C. for 12 h. TLC showed the reaction was complete and quenched with brine (10 mL). The aqueous layer was extracted with EtOAc (3*10 mL). The combined organic phases were washed with brine (3*10 mL), dried over Na_2SO_4. filtered and concentrated under reduced pressure to give the residue, which was purified by column chromatography (petroleum ether/ethyl acetate = 2/1) to give benzyl-5-(difluoro(3-(trifluoromethyl)phenyl)methyl)-3,4-dihydroxy-2-morpholinopiperidine-1-carboxylate **70** as white solid (160 mg, 71% yield).

R_f = 0.25 (50% EtOAc in hexane)

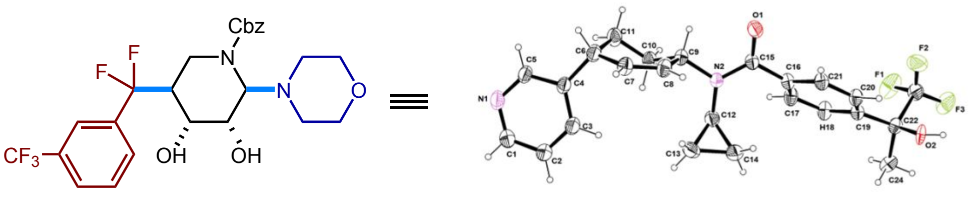

^{1}H NMR (400 MHz, CD_3CN) δ 7.95 – 7.70 (m, 3H), 7.69 – 7.60 (m, 1H), 7.46 – 7.19 (m, 5H), 5.10 (s, 2H), 4.42 (s, 1H), 4.13 – 3.96 (m, 2H), 3.96 – 3.83 (m, 1H), 3.68 – 3.57 (m, 1H), 3.57 – 3.39 (m, 4H), 3.27 – 3.08 (m, 1H), 3.07 – 2.91 (m, 1H), 2.88 – 2.70 (m, 1H), 2.46 – 2.23 (m, 4H). **^{13}C NMR** (151 MHz, CD_3CN) δ 157.73 – 156.59 (m), 139.01 – 138.20 (m), 137.88, 130.95 (q, J = 32.4 Hz), 130.40, 129.43, 128.89, 128.61, 128.40, 127.82 – 127.49 (m), 125.06 (q, J = 271.6 Hz), 123.87 (t, J = 246.2 Hz), 123.22, 77.42 – 76.48 (m), 68.09, 67.90, 67.29, 66.65, 50.32, 46.50 – 44.76 (m), 38.71 – 37.43 (m). **^{19}F NMR** (565 MHz, CD_3CN) δ -63.03, -92.50 – -96.91 (m), -98.97 – -103.27 (m).

HRMS (ESI) for $C_{25}H_{27}F_5N_2O_5$ $[M+H]^+$ m/z: calcd. 531.1913, found 531.1915.

VIII. Mechanistic Experiments

8.1 Stern-Volmer quenching experiments

Stern-Volmer fluorescence quenching experiments were record by a Hitachi RF-6000 fluorescence spectrometer. **$Pd(PPh_3)_4$** and K_3PO_4 was purchased from Bide. DMSO was degassed with N_2 for 30 mins. All solution prepared at glovebox and covered by silver paper. **$Pd(PPh_3)_4$** solution was excited at 420 nm and all samples was measured at 500 nm to 800 nm. Preparing solution of **$Pd(PPh_3)_4$** (34.7 mg in 30 mL DMSO, 1 mM), solution of 1,3-Bis(trifluoromethyl)-benzene **S1** (62.5 μL in 10 mL DMSO, 40 mM) and solution of 1,3-Cyclohexadiene **S2** (38 μL in 10 mL DMSO, 40 mM), solution of morpholine **S3** (35 μL in 10 mL DMSO, 40 mM).

8.1.1 Luminescence quenching of $Pd(PPh_3)_4$ by S1

1 mL **$Pd(PPh_3)_4$** solution in cuvettes were added **S1** solution (0 mL, 125 μL, 250 μL, 375 μL, 500 μL) and DMSO until to 2 mL to prepare five samples **$Pd(PPh_3)_4$**, **$Pd(PPh_3)_4$+S1** (2.5 mM), **$Pd(PPh_3)_4$+S1** (5 mM), **$Pd(PPh_3)_4$+S1** (7.5 mM), **$Pd(PPh_3)_4$+S1** (10 mM), The results were depicted as follows:

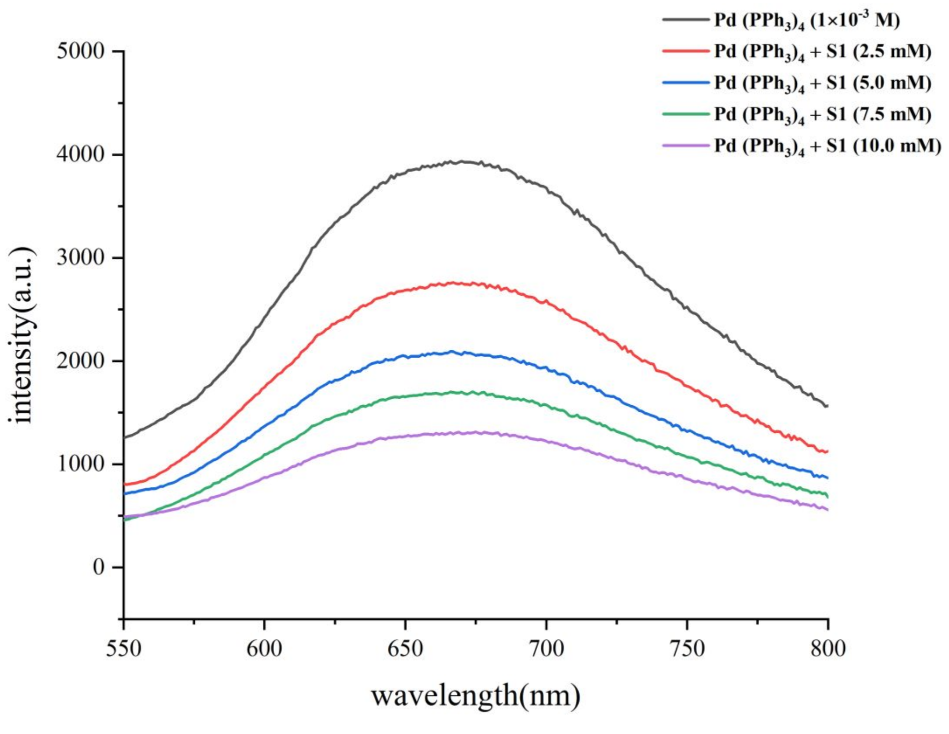

Figure S2. Emission quenching of **$Pd(PPh_3)_4$** by **S1** after irradiation at 420 nm. [Pd]=1×10^{-3} M in DMSO

8.1.2 Luminescence quenching of $Pd(PPh_3)_4$ by S2

1 mL **Pd(PPh$_3$)$_4$** solution in cuvettes were added **S2** solution (0 mL, 0.125 mL, 0.25 mL, 0.375 mL, 0.5mL) and DMSO until to 2 mL to prepare five samples **Pd(PPh$_3$)$_4$**, **Pd(PPh$_3$)$_4$+S2** (2.5 mM), **Pd(PPh$_3$)$_4$+S2** (5 mM), **Pd(PPh$_3$)$_4$+S2** (7.5 mM), **Pd(PPh$_3$)$_4$+S2** (10 mM), The results were depicted as follows:

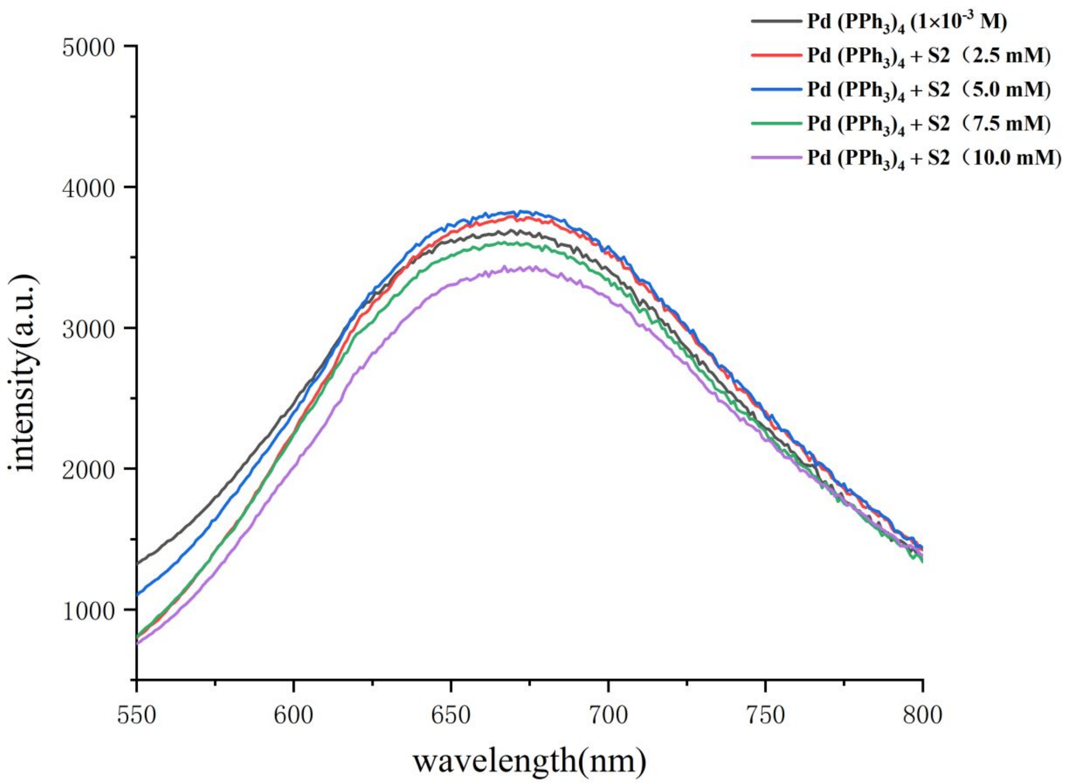

Figure S3. Emission quenching of **Pd(PPh$_3$)$_4$** by **S2** after irradiation at 420 nm. [Pd]=1×10^{-3} M in DMSO

8.1.3 Luminescence quenching of Pd(PPh$_3$)$_4$ by S3

1 mL **Pd(PPh$_3$)$_4$** solution in cuvettes were added **S3**solution (0 mL, 0.125 mL, 0.25 mL, 0.375 mL, 0.5mL) and DMSO until to 2 mL to prepare five samples **Pd(PPh$_3$)$_4$**, **Pd(PPh$_3$)$_4$+S3** (2.5 mM), **Pd(PPh$_3$)$_4$+S3** (5 mM), **Pd(PPh$_3$)$_4$+S3** (7.5 mM), **Pd(PPh$_3$)$_4$+S3** (10 mM), The results were depicted as follows:

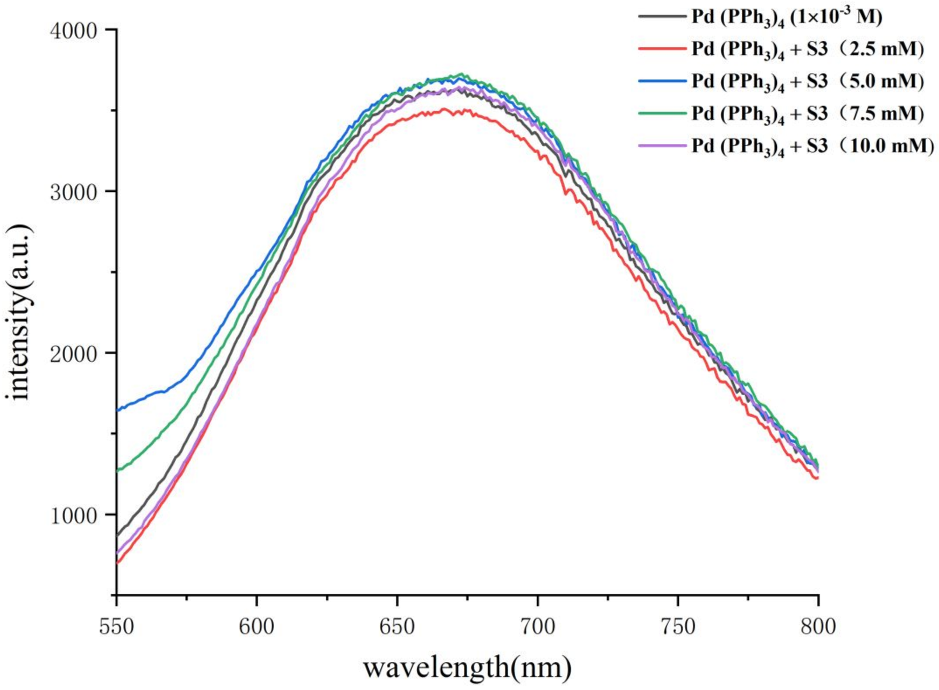

Figure S4. Emission quenching of **$Pd(PPh_3)_4$** by **S3** after irradiation at 420 nm. [Pd]=1×10^{-3} M in DMSO

8.1.4 Luminescence quenching of $Pd(PPh_3)_4$ by S1 and S2

1 mL **$Pd(PPh_3)_4$** solution in cuvettes were added **S1** solution (0 mL, 0.125 mL, 0.25 mL, 0.375 mL, 0.5mL), **S2** solution (0 mL, 0.125 mL, 0.25 mL, 0.375 mL, 0.5mL) and DMSO until to 2 mL to prepare five samples **$Pd(PPh_3)_4$**, **$Pd(PPh_3)_4$** + **S1** (2.5 mM)+ **S2**(2.5 mM), **$Pd(PPh_3)_4$** + **S1** (5 mM) + **S2** (5 mM), **$Pd(PPh_3)_4$** + **S1** (7.5 mM) + **S2** (7.5 mM), **$Pd(PPh_3)_4$**+**S1**(10 mM)+ **S2** (10 mM), The results were depicted as follows:

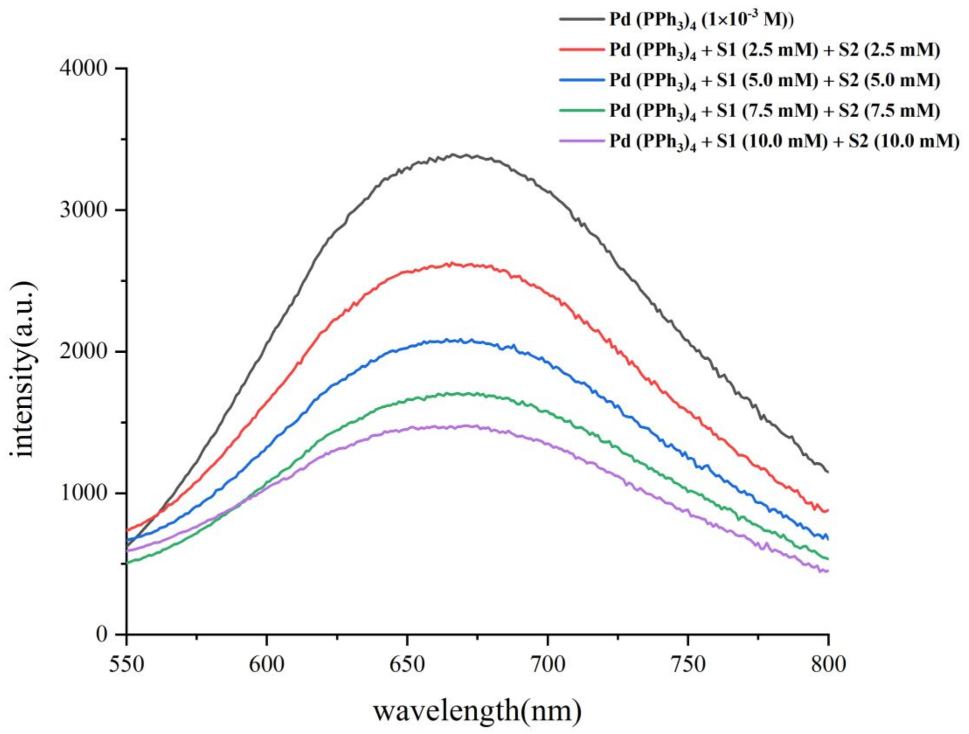

Figure S5. Emission quenching of **$Pd(PPh_3)_4$** by **S1 and S2** after irradiation at 420 nm. [Pd]=1×10^{-3} M in DMSO

8.1.5 Luminescence quenching of $Pd(PPh_3)_4$ by S1 and S3

1 mL **$Pd(PPh_3)_4$** solution in cuvettes were added **S1** solution (0 mL, 0.125 mL, 0.25 mL, 0.375 mL, 0.5mL), **S3** solution (0 mL, 0.125 mL, 0.25 mL, 0.375 mL, 0.5mL) and DMSO until to 2 mL to prepare five samples **$Pd(PPh_3)_4$**, **$Pd(PPh_3)_4$** + **S1** (2.5 mM) + **S3** (2.5 mM), **$Pd(PPh_3)_4$** + **S1** (5 mM)+ **S3** (5 mM), **$Pd(PPh_3)_4$** + **S1** (7.5 mM)+ **S3** (7.5 mM), **$Pd(PPh_3)_4$** + **S1** (10 mM)+ **S3** (10 mM), The results were depicted as follows:

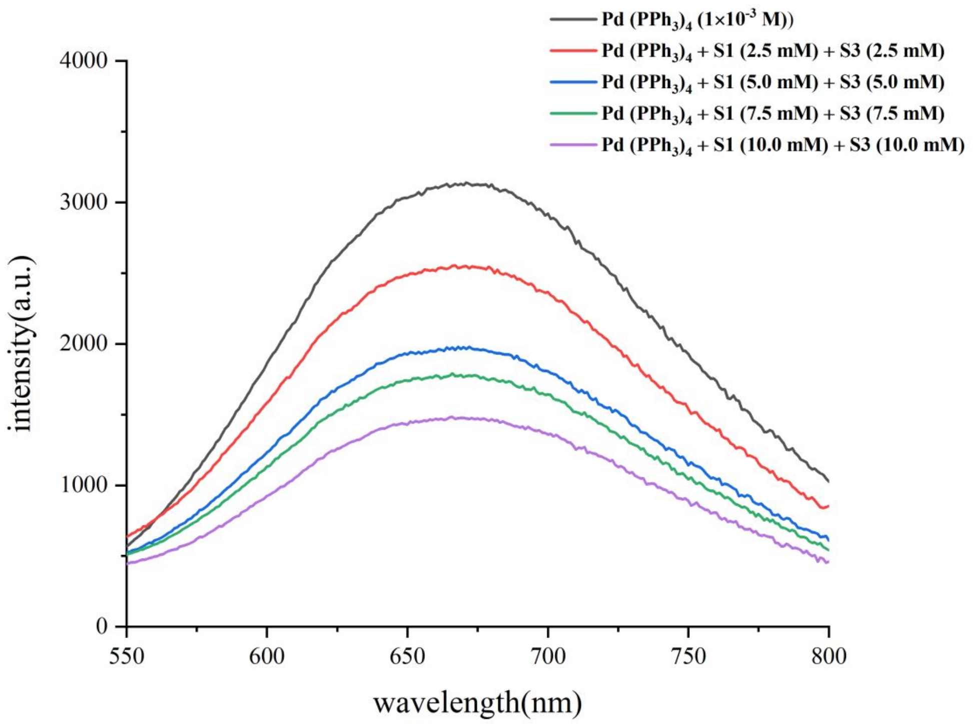

Figure S6. Emission quenching of **$Pd(PPh_3)_4$** by **S1 and S3** after irradiation at 420 nm. [Pd]=1×10^{-3} M in DMSO

8.1.6 Luminescence quenching of $Pd(PPh_3)_4$ by S2 and **S3**

1 mL **$Pd(PPh_3)_4$** solution in cuvettes were added **S2** solution (0 mL, 0.125 mL, 0.25 mL, 0.375 mL, 0.5mL), **S3** solution (0 mL, 0.125 mL, 0.25 mL, 0.375 mL, 0.5mL) and DMSO until to 2 mL to prepare five samples **$Pd(PPh_3)_4$**, **$Pd(PPh_3)_4$** + **S2** (2.5 mM)+ **S3** (2.5 mM), **$Pd(PPh_3)_4$** + **S2** (5 mM)+ **S3** (5 mM), **$Pd(PPh_3)_4$** +**S2** (7.5 mM)+ **S3** (7.5 mM), **$Pd(PPh_3)_4$** + **S2** (10 mM) + **S3** (10 mM), The results were depicted as follows:

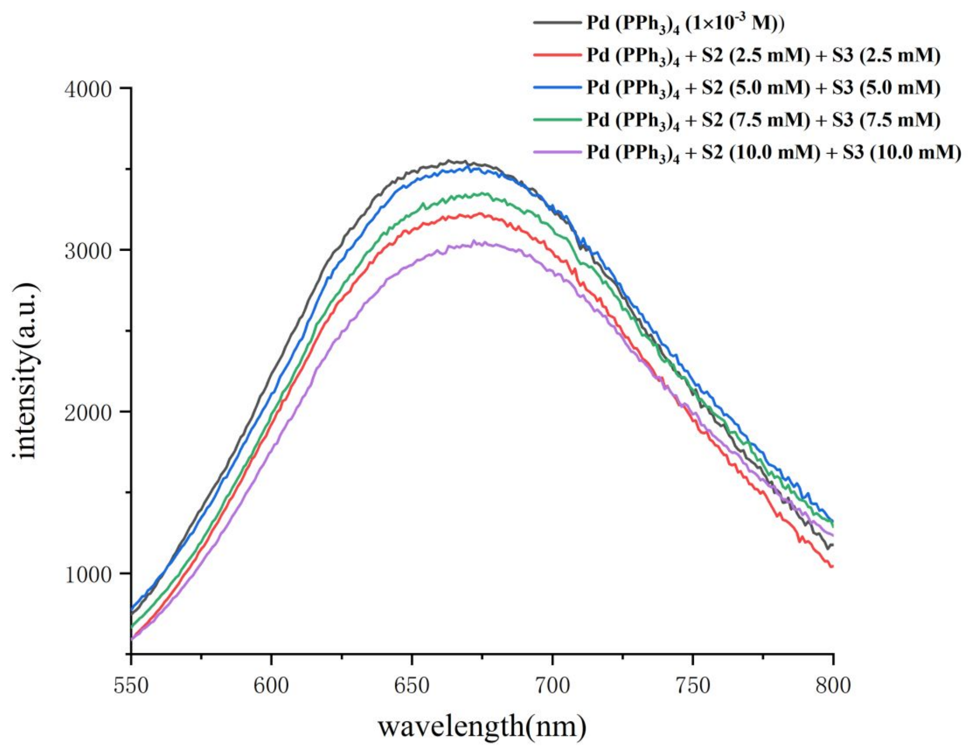

Figure S7. Emission quenching of **Pd(PPh$_3$)$_4$** by **S2 and S3** after irradiation at 420 nm. [Pd]=1×10^{-3} M in DMSO

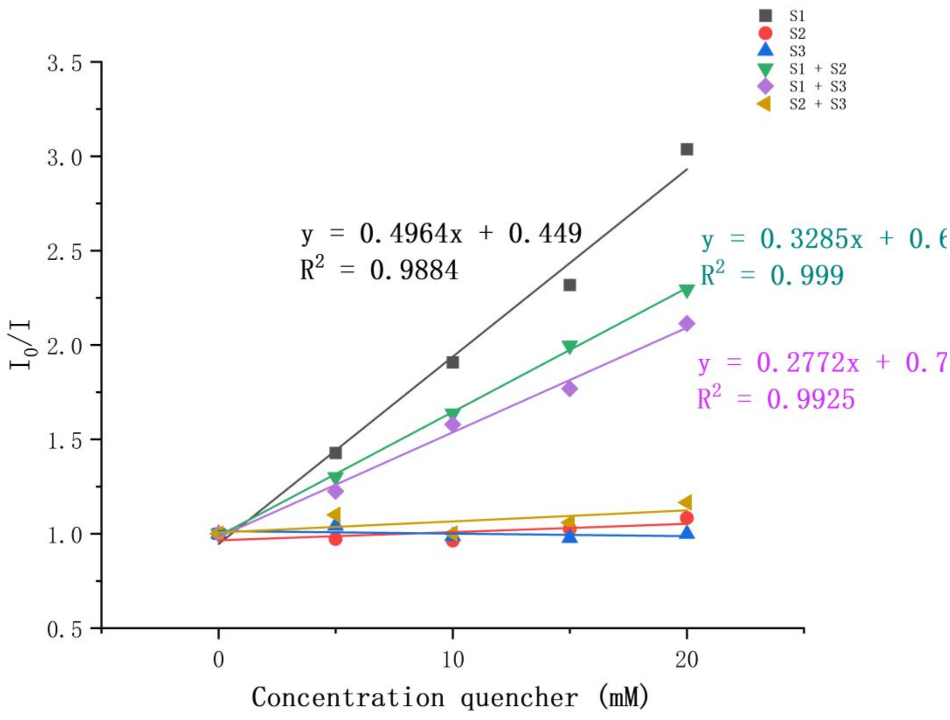

Figure S8. Stern-Volmer plot for the emission quenching of **Pd(PPh$_3$)$_4$** .

8.2 Table S23. Radical inhibition experiments.

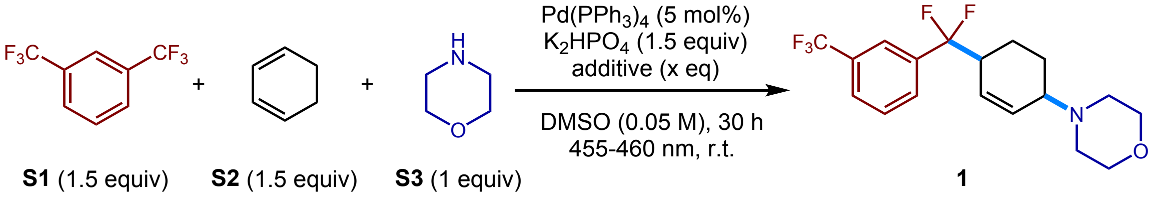

entry[a]	additive	Equivalent (x eq)	**1**/yield/%[b]
1	TEMPO	0.5	0
2	TEMPO	1.0	0
3	TEMPO	1.5	0
4	TEMPO	2.0	0

[a]0.15 mmol **S1**, 0.15 mmol **S2**, 0.1 mmol **S3**,5 mol% $Pd(PPh_3)_4$ and 0.15 mmol K_2HPO_4 in dry DMSO (2 mL) irradiated at 10 w 455-460 nm lamp panel at room temperature for 30 h. [b]Yield determined by ^{1}H-NMR using 2,2,2-Trifluoro-N,N-dimethylacetamide as an external standard.

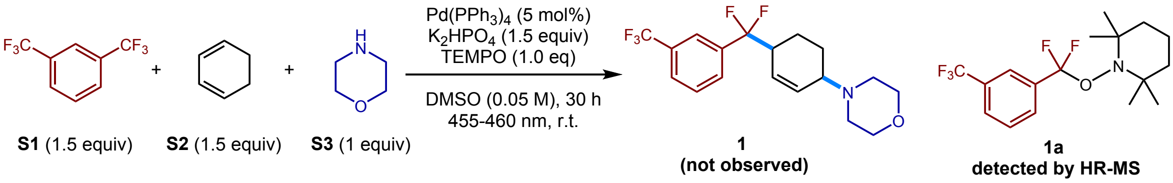

Following general procedure A, addition of 1.0 equiv TEMPO (15.6 mg, 0.1 mmol) to the reaction mixture completely shut-down the process and TEMPO trapping adduct **1a** was detected by HR-MS.

HRMS (ESI) for $C_{17}H_{22}F_5NO$ $[M+H]^+$ m/z: calcd. 352.1694, found 352.1706.

IX. Computational Studies

9.1 Methodology

To understand the mechanism that leads to the formation of the *cis*- and *trans*-1,4 di-substituted cyclohexenes, the Gibbs free energy profiles (at 298.15 K) of the reaction are calculated. Characterizing the energy profiles of reactions like the current one has always been challenging to computational chemists due to the large size of the system. There is almost an unlimited number of combinations of the density theory functionals and the basis sets (including effective core potentials) and in theory, there is no *definitive* hierarchy of these combinations on any given reaction systems. For example, the commonly referred Jacob's ladder speaks to the expected accuracy of a set of chemical reactions, instead of a specific one. In an ideal case, the optimal combination should be selected by comparing with results calculated from high level *ab initio* theories [6-9], but these calculations are too expensive for the current system. As an alternative, the choice of the density functional theory and basis sets is often selected from

previous literatures of similar reaction systems and then validated by the experiments. In this case, B3LYP/6-31G(d) with SDDAll as the effective core potential for Pd has been reported to provide accurate results for similar reactions involving Pd complexes [10-18]. These studies include carbon-carbon/carbon-heteroatom bond formation such as the Tsuji-Trost and Suzuki Miyaura reactions [12-13], the origin of ligand effects on stereo inversion of Pd-catalysed reactions [14], Pd-catalysed 1,1-alkynylbromination of alkenes [15], alkylation of aliphatic carboxamides *via* Pd-migration [16], Pd-catalysed selective C-C bond cleavage and alkenylation between cyclopropanol and 1,3 diene [17], and rearrangement of carboxy-substituted spiro[4.4]nonatriene through Pd-mediated vinyl-shift [18], to name a few recent examples. Therefore, B3LYP-D3/6-31G(d) [19-22] with SDDAll [23] on Pd is selected and the effect of solvent is accounted for with the polarizable continuum model (PCM) [24] employing *N*,*N*-dimethylformamide as the solvent. The stationary points are characterized as intermediates or transition states by its frequencies. A transition state is characterized by $3N$-7 positive and one imaginary frequency, whereas an intermediate on the PES is characterized by having $3N$-6 positive frequencies (where N is the number of atoms in the system). The connections between transition states and intermediates are confirmed with intrinsic reaction coordinate (IRC) calculations. To get a better account for the dispersion interaction, the double hybrid density functional, B2PLYP [25] single point energy was calculated on the B3LYP-D3/6-31G(d) geometries in presence of the PCM. All the calculations were performed using Gaussian software package. [26]

9.2 Results

9.2.1 The mechanism of the reaction

The *cis*- and *trans*-1,4 di-substituted cyclohexenes could technically be formed *via* two reaction mechanisms: (i) stepwise alkene migration followed by a nucleophilic substitution (S_N2') at the π-allylpalladium complex and (ii) cross-coupling of the substituted cyclohexyl radical and nucleophile in presence of the Pd catalyst. In the case where the nucleophile attacks allylic Pd(II) intermediate, studies have shown that the S_N2' is preferred thus it is the focus of the computational study. [27] The free energy corrections are calculated at the B3LYP-D3/6-31G(d)/SDDAll(Pd) with the PCM level of theory. The reaction starts with the photoexcitation of the catalyst $Pd(PPh_3)_4$ with a concerted Pd-PPh3 bond breaking via an intersystem crossing that leads to the formation of $Pd(PPh_3)_3^T$ in its triplet state. $Pd(PPh_3)_3^T + PPh_3$ is ~13 kcal/mol higher in energy as compared to the ground state catalyst, $Pd(PPh_3)_4$. The introduction of this triplet species then leads to an electron transfer from the photoexcited Pd catalyst to **S19**

(trifluoromethylated arene, Figure S9), forming a carbon radical. The addition of the carbon radical to **S2** (1,3-diene, Figure S9) leads to the formation of an allylic carbon radical **acr** (Figure S9), The free energy profile of pathway (i) can be found in Figure S10. The **acr** coordinates with the catalyst $Pd(PPh_3)_2$ through π-π interactions to form an intermediate **int0** after losing a fluoride and a PPh_3 ligand. The conformer of **int0** that eventually leads to the *cis*-conformer of the product (***c*-int0**, 1.1 kcal/mol) is ~ 3.2 kcal/mol lower in energy as compared to its *trans*-counterpart (***t*-int0**, 4.3 kcal/mol). The nucleophile **S3** (Figure S9) then forms a van der Waals complex with **int0** to yield intermediates ***c*-int1** (7.3 kcal/mol) and ***t*-int1** (12.5 kcal/mol). The intermediate **int1** serves as a pre-reaction complex for the nucleophilic substitution reaction, followed by **S3** further attacking the allylic ring (e.g., formation of a C-N bond and weakening the π-π interactions between the catalyst $Pd(PPh_3)_2$ and **acr**), i.e., transition states **ts1**. **ts1** is the rate-limiting step of the overall reaction in this pathway, where the energy barrier associated with the *trans*-pathway (***t*-ts1**, 20.4 kcal/mol) is higher than its *cis*-pathway counterpart (***c*-ts1**, 18.0 kcal/mol). **ts1** leads to the formation of stable post-reaction complex **int2**, which are (-47.8 and -42.5 kcal/mol lower compared to the reactants for the *cis*- and *trans*-pathway, respectively). Post-reaction complex **int2** can further decompose to the ***c*19-** (-81.5 kcal/mol) or ***t*19-product** (-80.2 kcal/mol) and the catalyst. The overall higher energies of the *trans*-version of the pathways ($\Delta\Delta G^{\ddagger}$ = 3.2, 5.2, 2.4, 5.3, and 1.3 kcal/mol for **int0**, **int1**, **ts1**, **int2**, and **product** respectively) is a result of the stronger steric hindrance of PPh_3 in the catalyst to the **acr**.

It is important to note that the structures shown in Figure S9 and S10 are not the only optimal structure of a specific stereoisomer. As reported, molecules of similar size possess many optimal structures [28]. The protocols of searching for these optimal structures in our study is reported elsewhere [29]. In this manuscript, the representative structures of the intermediates and transition states are selected from numerous candidates with the criteria of (1) possessing the lowest Gibbs free energy and (2) IRC connectivity. Here we use the products to demonstrate this point. As Figure S12 shows, for both the *cis*- and *trans*-product, the rotation of the C-CF_2 (θ) and C-N (φ) single bonds leads to many optimal (local minimal) structures that are connected by shallow barriers. It is also interesting to note that Gibbs free energy of the *cis*- and *trans*-product are similar due to the relative flexible rotation of the C-CF_2 (θ) and C-N (φ) bonds – the ***t*19-** and ***c*19-products** can look very similar (Figure S13).

9.2.2 The configurational isomer selectivity

According to the Eyring-Polanyi equation [30-31], the rate constant for a reaction can be written as,

$$k = \frac{k_B T}{hc^o} \exp\left(\frac{-\Delta G^{\ddagger}}{RT}\right)$$

where k_B is the Boltzman constant, T is the reaction temperature, h is the Planck's constant, c^0 is the standard state concentration, R is the molar gas constant, and $\Delta G^{\ddagger}$ is the free energy barrier for the reaction. According to the Curtin-Hammett principle, the barrier difference, not the difference of absolute barrier, should be used to compare the experimental selectivity, [32] thus the branching ratio between the *cis*- and *trans*-product via mechanism 1 should be:

$$\frac{k_{cis}}{k_{trans}} = \exp\left(\frac{-\Delta G^{\ddagger}_{c-\boldsymbol{ts1}} + \Delta G^{\ddagger}_{t-\boldsymbol{ts1}}}{RT}\right) = 56.8$$

Therefore, the computation predicts the overwhelming majority of the product should be in the *cis* configuration, agreeing well with the experimensts.

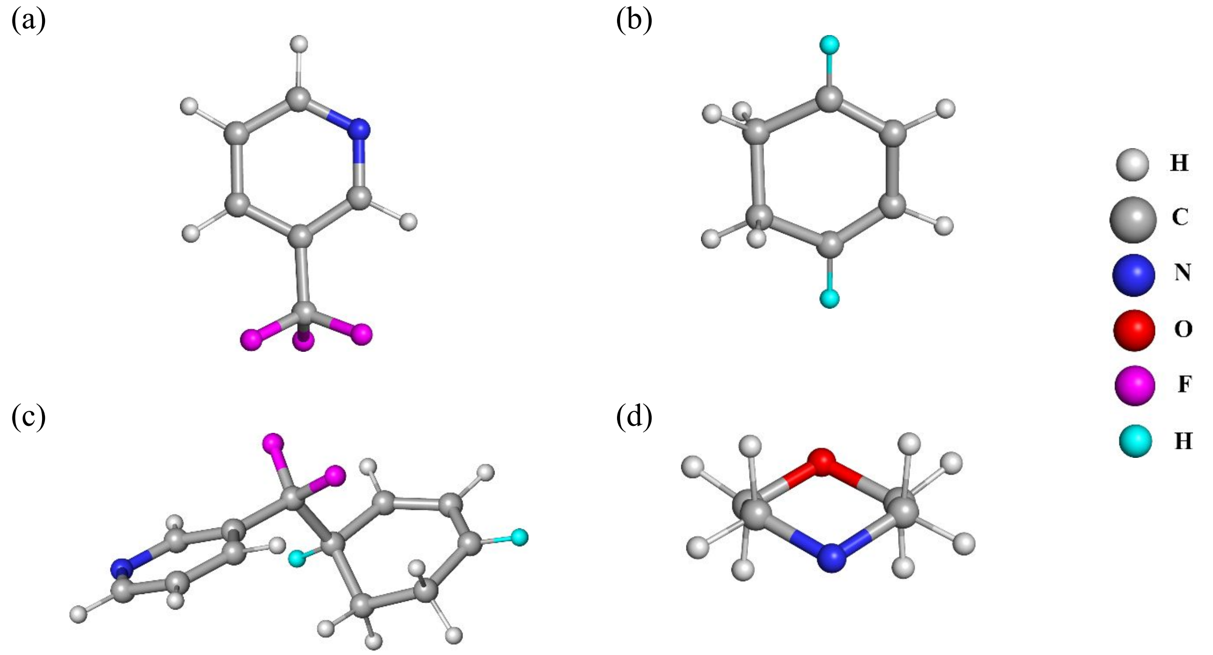

Figure S9. Optimized structures of (a) trifluoromethylated arene, **S19**; (b) cyclohex-1,3-diene, **S2**; (c) allylic carbon radical, **acr**; and (d) nucleophile, **S3**; at B3LYP-D3/6-31G* level of theory.

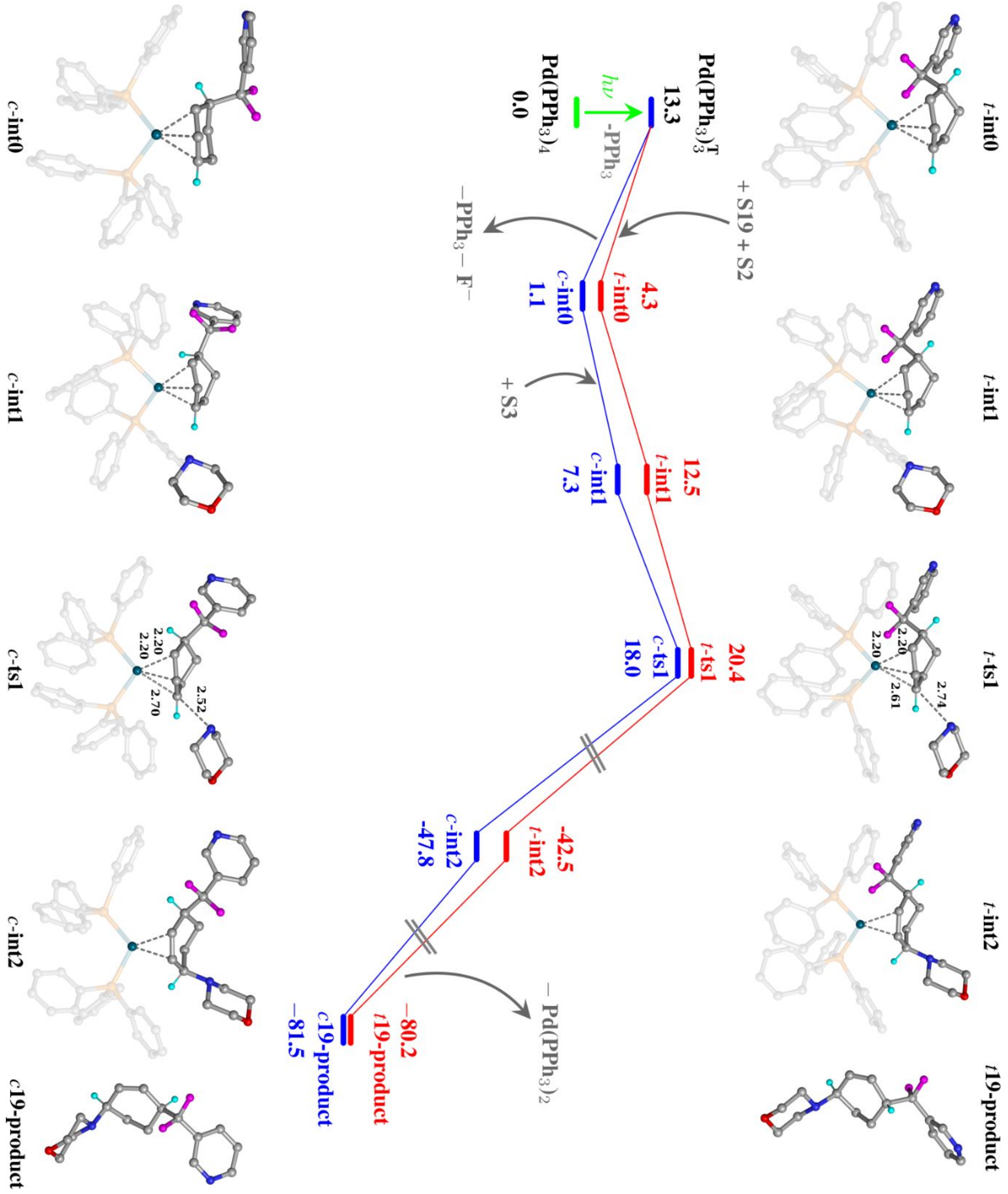

Figure S10. Gibbs free energy profile for the formation of *cis* and *trans*-products via mechanism (i), i.e. stepwise alkene migration followed by a nucleophilic substitution at the π-allylpalladium complex. Only the important H-atoms (cyan color) are shown in the figures for clarity.

Figure S11. Representative figure showing the atom numbers used to tabulate and compare different Pd-C interactions

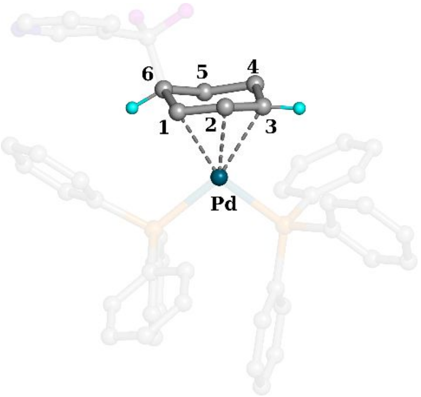

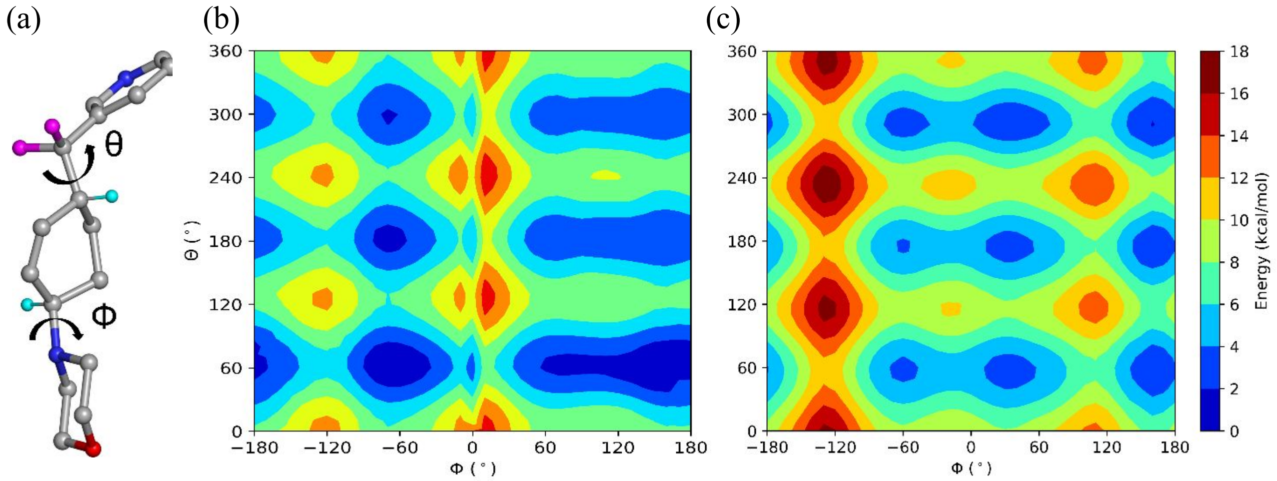

Figure S12. (a) Important internal coordinates θ and φ used to generate different conformational isomers for the *cis*- and *trans*-product. 2D potential energy surface at B3LYP-D3/6-31G* level of theory obtained by performing a relaxed scan of the two internal coordinates θ and φ for (b) ***c*19-product** and (c) ***t*19-product**.

(a) (b)

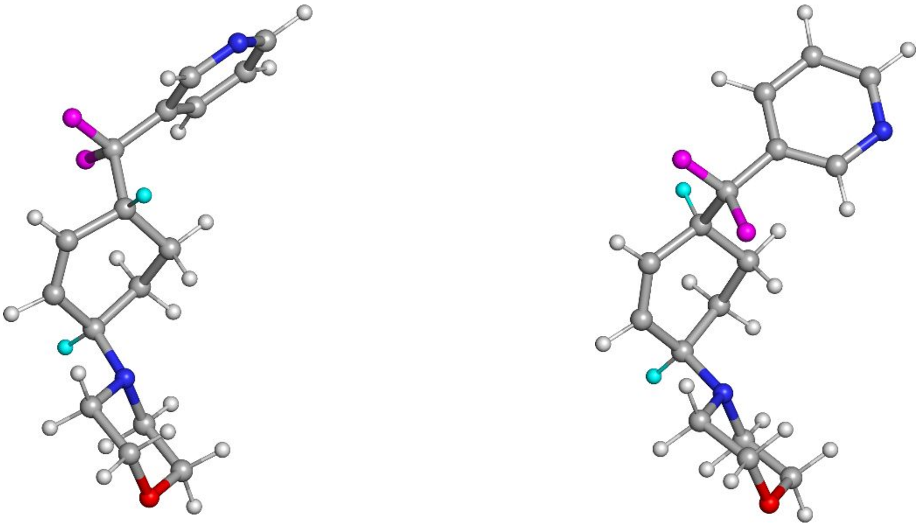

Figure S13. Representative figure showing one of the isomers of (a) ***t*19-** and (b) ***c*19-product**.

Table S24. Pd-C distances (in Å) for the intermediates *c*-/*t*-**int0** and *c*-/*t*-**int0′.** The label of the atoms can be found in Figure S11.

	**Pd-C1**	**Pd-C2**	**Pd-C3**	**Pd-C4**	**Pd-C5**	**Pd-C6**
***c*-int0**	2.21	2.18	2.28	3.03	3.62	3.06
***t*-int0**	2.23	2.16	2.28	3.13	3.69	3.12
***c*-int0′**	2.28	2.18	2.24	3.06	3.68	3.16
***t*-int0′**	2.25	2.19	2.24	2.99	3.57	3.02

Table S25. Geometrical parameters (θ and φ) for different ***t*19-** and ***c*19-product** isomers chosen to perform full geometry optimization and the relative free energies obtained after optimization at B3LYP-D3/6-31G(d) level of theory.

	φ (°)	θ (°)	ΔG (kcal/mol)
***c*19-product**	-60	60	-11.08
	-60	180	-10.89
	-60	300	-10.41
	-180	300	-9.80

	-180	60	-11.43
	-180	180	-10.47
	150	300	-9.86
	150	180	-10.47
	150	60	-11.47
	20	300	-9.63
	20	60	-9.25
	20	180	-9.16
	-60	60	-8.94
***t*19-product**	-60	300	-9.01
	-60	180	-8.61
	160	180	-9.80
	160	60	-9.99
	160	300	-10.05

Optimized coordinates for different stationary points:

1. **S19**

14

C	-1.997632	-2.765440	0.203877
F	-1.439358	-2.058193	1.212938
C	-2.754406	-3.957765	0.713803
C	-3.889132	-4.418248	0.041176
C	-2.307009	-4.652099	1.839266
H	-4.258754	-3.886478	-0.831851
C	-3.015766	-5.780502	2.241193
H	-1.433401	-4.312134	2.385831
C	-4.136391	-6.163221	1.500116
H	-2.711442	-6.352788	3.111026
H	-4.708943	-7.041804	1.790008
F	-2.790312	-1.922617	-0.495268
N	-4.577133	-5.502228	0.419121
F	-0.986619	-3.129778	-0.622397

2. **S2**

14

C	0.111620	-1.425756	-0.068737
C	1.259209	-0.726371	-0.107854
C	1.259208	0.726373	0.107854
C	0.111618	1.425756	0.068737
H	0.111951	-2.506463	-0.193452
H	2.210535	-1.225439	-0.279550
H	2.210533	1.225441	0.279550
H	0.111948	2.506463	0.193452
C	-1.195834	0.730896	-0.241749
H	-1.352536	0.757544	-1.333848
H	-2.040805	1.272065	0.198735
C	-1.195833	-0.730897	0.241749
H	-2.040803	-1.272067	-0.198735
H	-1.352535	-0.757546	1.333848

3. **S3**

14

C	1.149189	0.781892	0.180558
C	-1.149379	0.781600	0.180612
C	1.184027	-0.713623	-0.189665
H	1.246065	0.789105	1.312666
H	2.085041	1.239695	-0.196699
C	-1.183853	-0.713834	-0.189721
H	-1.246158	0.788731	1.312764

H	-2.085297	1.239321	-0.196547
H	2.025141	-1.256443	0.269467
H	1.255315	-0.813165	-1.289659
H	-2.024885	-1.256941	0.269221
H	-1.254970	-0.813196	-1.289743
N	-0.000170	1.471520	-0.322542
O	0.000129	-1.379245	0.271952

4. **acr**

27

C	-0.450604	-1.970339	-1.783670
C	0.826299	-1.486838	-1.577230
C	-0.971038	-3.215946	-1.154155
H	-1.145548	-1.380162	-2.375054
H	1.139405	-0.553855	-2.031835
C	0.162519	-4.159095	-0.717717
H	-1.615566	-3.715900	-1.886628
C	1.314961	-3.419637	-0.029965
H	-0.225723	-4.949472	-0.071470
H	0.551020	-4.645112	-1.617829
H	2.224582	-4.033656	0.015528
H	1.105019	-3.183254	1.029015
C	-1.936563	-2.806837	0.003578
F	-1.201732	-2.131477	0.950415
C	-2.671850	-3.957266	0.635017
C	-3.749506	-4.540296	-0.042256
C	-2.300938	-4.474451	1.877428
H	-4.066784	-4.146756	-1.005818
C	-3.021547	-5.552913	2.385298
H	-1.478894	-4.038778	2.434601
C	-4.076271	-6.067046	1.629328
H	-2.773070	-5.986559	3.348017
H	-4.653215	-6.912229	1.998217
F	-2.840396	-1.908219	-0.514112
N	-4.446142	-5.575838	0.435781
C	1.646597	-2.131249	-0.654417
H	2.562349	-1.635696	-0.337526

5. ***t*-int0**

96

Pd	-0.040739	-0.319222	-0.938153
C	-1.799487	-0.077136	-2.280595
C	-0.743048	-0.758830	-2.936628
C	-2.998018	-0.881837	-1.762136
H	-1.999183	0.955174	-2.551359

H	-0.093500	-0.232891	-3.632586
C	-2.859628	-2.376022	-2.129740
H	-3.873242	-0.483070	-2.291437
C	-1.456367	-2.948415	-1.869728
H	-3.616635	-2.971900	-1.611427
H	-3.070663	-2.459419	-3.201258
H	-1.362938	-3.912761	-2.384751
H	-1.309944	-3.163173	-0.808077
C	-3.334895	-0.673102	-0.277802
F	-2.393776	-1.315738	0.517474
C	-4.708725	-1.140108	0.137325
C	-5.822979	-0.340192	-0.140608
C	-4.910285	-2.366811	0.772584
H	-5.691159	0.626427	-0.622611
C	-6.213504	-2.740137	1.096346
H	-4.068650	-3.008268	1.011236
C	-7.257390	-1.872471	0.770618
H	-6.417588	-3.683314	1.592184
H	-8.285342	-2.138309	1.008301
F	-3.218746	0.668171	0.021157
N	-7.077907	-0.688157	0.164761
P	1.902626	-1.259744	0.076288
P	-0.053676	1.843983	0.017542
C	-0.380779	-2.020202	-2.415031
H	0.542479	-2.474328	-2.763289
C	1.585025	2.611757	-0.345669
C	2.299823	3.396458	0.568228
C	2.127760	2.398532	-1.626510
C	3.513226	3.979885	0.197613
H	1.934323	3.538753	1.577672
C	3.336175	2.988506	-1.995802
H	1.608029	1.764832	-2.339699
C	4.029765	3.787779	-1.084432
H	4.060221	4.575908	0.922165
H	3.736982	2.814034	-2.990272
H	4.975015	4.242712	-1.366021
C	-1.253473	3.046383	-0.710768
C	-1.039431	3.548346	-2.005867
C	-2.432337	3.399044	-0.035892
C	-1.980392	4.385397	-2.607337
H	-0.135804	3.298011	-2.551209
C	-3.370358	4.238882	-0.639339
H	-2.629165	3.015091	0.958082
C	-3.149704	4.733648	-1.926170
H	-1.795468	4.766731	-3.607585
H	-4.275068	4.503662	-0.099287
H	-3.881271	5.385966	-2.394313
C	-0.345624	1.942309	1.821091
C	-0.388462	3.169136	2.507684
C	-0.557109	0.754049	2.536153

C	-0.594825	3.197050	3.886910
H	-0.275700	4.103009	1.965458
C	-0.768565	0.784081	3.915840
H	-0.560745	-0.197543	2.016971
C	-0.778160	2.004555	4.594129
H	-0.618243	4.149824	4.407527
H	-0.924291	-0.145741	4.455192
H	-0.938914	2.029585	5.668018
C	3.096701	-0.163424	0.955770
C	4.266719	0.316552	0.351977
C	2.804712	0.217257	2.276749
C	5.129563	1.155397	1.059945
H	4.516719	0.038485	-0.665437
C	3.666962	1.058450	2.978167
H	1.912228	-0.152516	2.768531
C	4.834696	1.528087	2.372084
H	6.031201	1.521440	0.577892
H	3.425212	1.341949	3.998653
H	5.507785	2.183474	2.917388
C	2.887429	-2.064175	-1.254025
C	3.317576	-3.396606	-1.196878
C	3.151575	-1.308916	-2.411660
C	4.010001	-3.958337	-2.273960
H	3.110525	-4.004943	-0.323439
C	3.855427	-1.868125	-3.477344
H	2.800616	-0.283221	-2.481460
C	4.284624	-3.197514	-3.411557
H	4.333495	-4.993870	-2.219183
H	4.058513	-1.269739	-4.360828
H	4.823561	-3.637871	-4.245408
C	1.578448	-2.548756	1.344239
C	0.268719	-2.963793	1.625131
C	2.644130	-3.107945	2.073383
C	0.028186	-3.930604	2.605457
H	-0.571642	-2.528096	1.100192
C	2.401812	-4.077474	3.045100
H	3.661929	-2.779063	1.885899
C	1.092462	-4.491371	3.312509
H	-0.992130	-4.237991	2.815999
H	3.233325	-4.504469	3.598118
H	0.905108	-5.242160	4.074745

6. ***t*-int1**

110

Pd	0.075637	0.257737	-0.511995
C	1.151216	1.610485	-2.016060
C	-0.250734	1.697853	-2.106339

C	1.920231	2.719226	-1.296326
H	1.701659	1.070130	-2.778156
H	-0.790372	1.183284	-2.899098
C	0.971340	3.819036	-0.771101
C	-0.303146	3.268044	-0.112302
H	1.502110	4.489690	-0.088145
H	0.676064	4.421114	-1.637672
H	-1.027279	4.081592	0.017753
H	-0.097276	2.897113	0.897609
P	-1.497479	-0.243971	1.206875
P	1.367317	-1.753446	-0.777979
C	-0.948211	2.197695	-0.978729
H	-2.042372	2.083373	-0.987324
C	-5.216786	1.264028	-1.171376
C	-4.228731	3.316333	-1.620722
C	-6.326875	1.555623	-2.194096
H	-5.634158	1.589015	-0.175185
H	-5.084457	0.175183	-1.100752
C	-5.311991	3.642089	-2.660638
H	-4.592151	3.785559	-0.662472
H	-3.305268	3.858458	-1.894082
H	-7.301109	1.143172	-1.893763
H	-6.044675	1.119161	-3.170540
H	-5.564109	4.712080	-2.694888
H	-4.961889	3.328500	-3.661843
N	-3.972078	1.905340	-1.501198
O	-6.534165	2.965591	-2.345133
C	0.251435	-3.228358	-0.808191
C	-0.122637	-3.817275	0.411939
C	-0.321502	-3.711387	-1.994884
C	-1.059637	-4.849318	0.444365
H	0.323875	-3.480869	1.339781
C	-1.260438	-4.745234	-1.958132
H	-0.040894	-3.291993	-2.954196
C	-1.638298	-5.312412	-0.739525
H	-1.338571	-5.283610	1.400110
H	-1.694115	-5.105773	-2.886711
H	-2.373904	-6.111202	-0.713266
C	2.176389	-1.769243	-2.436359
C	3.508714	-2.162529	-2.625383
C	1.432508	-1.335630	-3.549311
C	4.081129	-2.128017	-3.900198
H	4.106497	-2.493610	-1.783228
C	2.001073	-1.316700	-4.822824
H	0.406497	-1.001097	-3.419317
C	3.330630	-1.710558	-5.000840
H	5.116368	-2.431120	-4.029462
H	1.409157	-0.984043	-5.670663
H	3.778472	-1.686577	-5.990125
C	2.699887	-2.270224	0.380649

C	3.354553	-1.311737	1.166271
C	3.099413	-3.615132	0.472016
C	4.381783	-1.688774	2.033632
H	3.058569	-0.274714	1.110532
C	4.122766	-3.990406	1.342656
H	2.608015	-4.371949	-0.130961
C	4.765109	-3.028197	2.127482
H	4.874692	-0.934579	2.640685
H	4.417672	-5.033886	1.406933
H	5.559101	-3.322566	2.807925
C	-0.818098	-1.349225	2.514754
C	-1.563574	-2.376643	3.111799
C	0.527202	-1.184146	2.878503
C	-0.964547	-3.233213	4.037410
H	-2.601267	-2.530818	2.837724
C	1.124699	-2.038610	3.805929
H	1.116819	-0.399118	2.416746
C	0.380840	-3.071883	4.381080
H	-1.547935	-4.035166	4.481038
H	2.174468	-1.911777	4.052172
H	0.847533	-3.750417	5.089451
C	-3.039843	-1.070230	0.668917
C	-4.211203	-1.027871	1.442665
C	-3.019075	-1.836395	-0.505007
C	-5.335594	-1.756767	1.053805
H	-4.244563	-0.426863	2.346035
C	-4.145931	-2.563109	-0.892192
H	-2.121295	-1.873399	-1.111836
C	-5.303702	-2.529162	-0.111150
H	-6.236835	-1.718989	1.659214
H	-4.111733	-3.159714	-1.798959
H	-6.180468	-3.096251	-0.411320
C	-2.043238	1.250450	2.139864
C	-3.010528	2.100495	1.572120
C	-1.445698	1.597666	3.361557
C	-3.366981	3.276748	2.236058
H	-3.436943	1.882103	0.585269
C	-1.805577	2.781160	4.011600
H	-0.704138	0.949574	3.816997
C	-2.768376	3.623110	3.451340
H	-4.114224	3.928103	1.791474
H	-1.335869	3.037659	4.957376
H	-3.050032	4.542392	3.958041
C	2.899608	2.212967	-0.234274
C	3.934982	3.209327	0.225880
C	3.789259	3.928395	1.413806
C	5.063241	3.450929	-0.566240
H	2.931205	3.762347	2.056270
H	5.206534	2.896260	-1.491092
C	5.857604	5.025232	0.891931

H	6.639400	5.743791	1.129359
F	3.552212	1.104423	-0.744414
F	2.193162	1.768261	0.879533
H	2.569048	3.168458	-2.060704
C	4.772621	4.855830	1.753664
H	4.701573	5.435618	2.667946
N	6.013074	4.338494	-0.250843

7. ***t*-ts1**

110

Pd	0.016557	-0.142296	-0.459715
C	-0.511535	-1.844126	-1.748673
C	0.891212	-1.621035	-1.829039
C	-1.026130	-3.076034	-0.986884
H	-1.133800	-1.516499	-2.576825
H	1.299776	-1.015412	-2.635865
C	0.144878	-3.974866	-0.529541
H	-1.633784	-3.651712	-1.697501
C	1.304343	-3.187672	0.101465
H	-0.208436	-4.752790	0.154515
H	0.526626	-4.486261	-1.420311
H	2.155668	-3.853559	0.271267
H	1.016585	-2.811781	1.088143
C	-2.000348	-2.775393	0.163452
F	-1.328231	-2.174116	1.221038
C	-2.750753	-3.972531	0.697843
C	-3.863811	-4.456474	0.001083
C	-2.353589	-4.636489	1.859866
H	-4.203085	-3.947325	-0.898675
C	-3.080027	-5.753698	2.268537
H	-1.500599	-4.283573	2.429720
C	-4.171071	-6.159485	1.498094
H	-2.808218	-6.299757	3.165891
H	-4.754843	-7.030075	1.790227
F	-2.928656	-1.848153	-0.265052
N	-4.568646	-5.527974	0.382356
P	1.422426	1.226199	0.891066
P	-1.896923	1.203266	-0.781588
C	1.747806	-2.072462	-0.810367
H	2.777579	-1.774760	-0.831875
C	4.776689	-1.798522	-1.836128
C	4.518962	-3.893266	-0.867668
C	6.297842	-2.004307	-1.924046
H	4.610025	-1.244836	-0.870278
H	4.468396	-1.107506	-2.636009
C	6.039646	-4.130773	-0.938564
H	4.332123	-3.468251	0.155639

H	4.012963	-4.872622	-0.896867
H	6.847476	-1.065955	-1.778637
H	6.556627	-2.419500	-2.914105
H	6.407104	-4.720019	-0.087893
H	6.281239	-4.668338	-1.872671
N	4.047444	-3.044573	-1.930266
O	6.748435	-2.891011	-0.897061
C	-1.327129	2.842071	-1.420822
C	-1.856406	4.076896	-1.023600
C	-0.290114	2.828243	-2.372080
C	-1.383115	5.263363	-1.588267
H	-2.620969	4.127515	-0.257920
C	0.174959	4.013104	-2.941733
H	0.164012	1.883691	-2.659849
C	-0.376489	5.236595	-2.554691
H	-1.798030	6.212102	-1.259924
H	0.975190	3.978661	-3.675960
H	-0.011240	6.162887	-2.989176
C	-3.093486	0.660658	-2.085101
C	-2.699733	0.686791	-3.434584
C	-4.344360	0.110232	-1.763841
C	-3.537460	0.187916	-4.433053
H	-1.738214	1.102627	-3.716962
C	-5.181859	-0.387657	-2.764285
H	-4.667676	0.057005	-0.730870
C	-4.783460	-0.351563	-4.102132
H	-3.214252	0.222782	-5.469883
H	-6.147589	-0.805501	-2.492886
H	-5.435755	-0.740171	-4.879086
C	-2.944264	1.525795	0.689783
C	-4.117667	2.299322	0.642070
C	-2.557226	0.955703	1.912320
C	-4.861747	2.527252	1.800340
H	-4.459595	2.712288	-0.302190
C	-3.304336	1.180753	3.070359
H	-1.670961	0.331018	1.956634
C	-4.452735	1.973715	3.017700
H	-5.762650	3.132114	1.750973
H	-2.987357	0.736266	4.009637
H	-5.033723	2.152199	3.918104
C	0.953586	2.991281	1.182945
C	1.521204	4.055775	0.469996
C	-0.051012	3.267319	2.126449
C	1.098531	5.366377	0.702230
H	2.294075	3.871326	-0.267930
C	-0.474261	4.576178	2.353051
H	-0.499152	2.461507	2.697121
C	0.102380	5.632151	1.642791
H	1.546843	6.179083	0.137955
H	-1.252966	4.767822	3.086410

H	-0.226090	6.652935	1.817435
C	3.119121	1.345885	0.181139
C	4.297962	1.202589	0.926247
C	3.215079	1.597330	-1.199056
C	5.544187	1.335796	0.307288
H	4.254266	0.984212	1.987556
C	4.459185	1.749071	-1.810298
H	2.310798	1.677868	-1.795058
C	5.628912	1.621758	-1.056443
H	6.449341	1.216561	0.896243
H	4.514012	1.952165	-2.876093
H	6.599476	1.729154	-1.532284
C	1.621343	0.626686	2.620028
C	1.051696	-0.600419	2.987994
C	2.287472	1.388229	3.597066
C	1.164587	-1.072817	4.298470
H	0.498851	-1.176766	2.256213
C	2.405699	0.913375	4.902949
H	2.703251	2.357681	3.338784
C	1.846788	-0.320013	5.255556
H	0.713627	-2.023787	4.568142
H	2.926910	1.508561	5.647408
H	1.935197	-0.684926	6.274965

8. ***t*-int2**

110

Pd	-0.000595	0.085681	-0.413777
C	0.924131	-1.744371	-1.376032
C	1.888573	-0.739880	-1.200351
C	0.993107	-3.079339	-0.625566
H	0.380793	-1.785700	-2.317400
H	2.009089	-0.031163	-2.018746
C	2.371080	-3.282375	0.042284
H	0.854722	-3.876101	-1.368645
C	2.866543	-2.002104	0.721658
H	2.337079	-4.111566	0.756610
H	3.082346	-3.570207	-0.739609
H	3.760553	-2.199055	1.318907
H	2.110798	-1.641971	1.421267
C	-0.158601	-3.321644	0.365557
F	-0.086899	-2.439868	1.434380
C	-0.249151	-4.726355	0.917938
C	-0.798843	-5.744698	0.130924
C	0.223318	-5.054367	2.189947
H	-1.190604	-5.512800	-0.857338
C	0.132629	-6.379217	2.613692
H	0.647393	-4.288533	2.830606

C	-0.425893	-7.320714	1.747547
H	0.486432	-6.677882	3.595098
H	-0.506608	-8.363255	2.048963
F	-1.354179	-3.047686	-0.269392
N	-0.890772	-7.020550	0.524668
P	0.704335	2.014681	0.724135
P	-2.348300	0.092445	-0.775213
C	3.115619	-0.889609	-0.304829
H	3.287355	0.050503	0.246397
C	4.726478	0.098630	-1.837856
C	5.468188	-1.710446	-0.488415
C	5.872565	-0.161885	-2.806728
H	5.050834	0.849542	-1.094191
H	3.892358	0.538182	-2.388797
C	6.607578	-1.935931	-1.481855
H	5.823628	-1.052143	0.329208
H	5.217439	-2.680794	-0.052704
H	6.216175	0.778066	-3.249894
H	5.533462	-0.831124	-3.615189
H	7.495102	-2.309400	-0.961623
H	6.292676	-2.687186	-2.225752
N	4.302912	-1.145815	-1.178885
O	6.994004	-0.733956	-2.138559
C	-2.930221	1.754961	-1.362715
C	-4.208042	2.276087	-1.114330
C	-2.023014	2.526200	-2.107545
C	-4.567307	3.536128	-1.597251
H	-4.925485	1.712397	-0.527830
C	-2.386575	3.777902	-2.606044
H	-1.019008	2.146005	-2.279956
C	-3.660160	4.289280	-2.346756
H	-5.557777	3.930073	-1.384959
H	-1.669199	4.359214	-3.179215
H	-3.941523	5.270395	-2.719502
C	-3.047618	-0.995524	-2.107328
C	-2.920805	-0.598683	-3.450565
C	-3.583214	-2.265990	-1.837370
C	-3.321860	-1.441592	-4.488635
H	-2.513050	0.378767	-3.692076
C	-3.988580	-3.106917	-2.876105
H	-3.680290	-2.607694	-0.813662
C	-3.859565	-2.700244	-4.206227
H	-3.218196	-1.110425	-5.518687
H	-4.406026	-4.082781	-2.641660
H	-4.174865	-3.355643	-5.013432
C	-3.407274	-0.259631	0.696178
C	-4.778398	-0.562981	0.630759
C	-2.793190	-0.193823	1.958026
C	-5.514977	-0.780113	1.796827
H	-5.271984	-0.642205	-0.333143

C	-3.530339	-0.405773	3.125148
H	-1.729822	0.017204	2.021736
C	-4.893847	-0.698370	3.046891
H	-6.573913	-1.013906	1.728489
H	-3.035810	-0.348286	4.091166
H	-5.469234	-0.868086	3.952788
C	-0.334096	3.547799	0.805020
C	0.144746	4.833440	0.514467
C	-1.674388	3.398906	1.196987
C	-0.700706	5.943617	0.610408
H	1.177886	4.975745	0.214566
C	-2.514796	4.505534	1.299269
H	-2.066979	2.409776	1.411456
C	-2.030457	5.783771	1.003798
H	-0.314933	6.933273	0.379721
H	-3.551986	4.367449	1.590992
H	-2.687347	6.646531	1.073872
C	2.298654	2.680781	0.070727
C	3.436435	2.943440	0.846290
C	2.357400	2.915463	-1.315166
C	4.601274	3.439110	0.251426
H	3.420649	2.764610	1.916380
C	3.512468	3.426224	-1.905051
H	1.490698	2.692900	-1.933354
C	4.641550	3.688062	-1.121620
H	5.476284	3.632652	0.866149
H	3.536917	3.607202	-2.976249
H	5.546652	4.075677	-1.580759
C	1.001222	1.740584	2.529573
C	0.918173	0.435657	3.038366
C	1.260199	2.798976	3.417414
C	1.105960	0.188575	4.401210
H	0.687555	-0.384935	2.367080
C	1.454084	2.552181	4.777127
H	1.303923	3.818590	3.044588
C	1.378500	1.245620	5.271890
H	1.032880	-0.827607	4.779490
H	1.657933	3.379249	5.451805
H	1.523257	1.056306	6.331975

9. ***t*19-product**

41

C	-0.177186	1.900298	0.294637
C	-1.353195	1.799142	-0.331369
C	0.769461	0.741143	0.503913
H	0.149145	2.863722	0.678412
H	-1.977838	2.685083	-0.424521

C	0.126084	-0.596035	0.087012
C	-0.711190	-0.455409	-1.188656
H	0.888695	-1.372954	-0.026603
H	-0.539910	-0.916082	0.895406
H	-0.092815	-0.079375	-2.009825
H	-1.088016	-1.436408	-1.494193
C	-1.877457	0.523812	-0.957548
D	-2.310666	0.804427	-1.936464
C	-3.793796	0.797229	0.615597
C	-3.772631	-1.015946	-0.934827
C	-4.759325	-0.001074	1.488122
H	-3.179528	1.431084	1.261284
H	-4.376626	1.458020	-0.055538
C	-4.738326	-1.778887	-0.033474
H	-3.147549	-1.733766	-1.474493
H	-4.354415	-0.447839	-1.687086
H	-4.184756	-0.571431	2.236686
H	-5.447956	0.670254	2.010034
H	-4.166760	-2.427575	0.650650
H	-5.410536	-2.402657	-0.630402
N	-2.930249	-0.123197	-0.131028
O	-5.563061	-0.887072	0.712523
C	2.111633	1.024858	-0.194868
D	1.030686	0.692237	1.570230
F	2.577234	2.246705	0.266515
F	1.907712	1.184302	-1.551522
C	3.192782	-0.005866	0.024868
C	3.897831	-0.027769	1.232913
C	3.495631	-0.970617	-0.937143
H	3.692426	0.722350	1.993624
C	4.480251	-1.913310	-0.648027
H	2.973754	-0.977204	-1.888040
C	5.119929	-1.852163	0.590933
H	4.748834	-2.680219	-1.367199
H	5.890260	-2.576981	0.846552
N	4.844758	-0.927683	1.524346

10. ***c*-int0**

96

Pd	0.046289	-0.641264	-0.461385
C	-1.891995	-0.828693	-1.511864
C	-0.985434	-1.818649	-1.976276
C	-2.959118	-1.195014	-0.481356
H	-2.123013	0.007545	-2.164130
H	-0.472192	-1.699278	-2.927367
C	-2.910004	-2.700308	-0.155090
C	-1.478034	-3.198309	0.087377

H	-3.535656	-2.919598	0.714631
H	-3.328300	-3.245529	-1.008845
H	-1.470694	-4.293652	0.139449
H	-1.128899	-2.852414	1.067392
P	2.300000	-1.130916	0.135752
P	-0.053796	1.664824	-0.033333
C	-0.542166	-2.763526	-1.031987
H	0.281063	-3.421597	-1.295617
C	1.129657	2.521441	-1.150025
C	1.961717	3.573286	-0.746249
C	1.189451	2.075597	-2.483875
C	2.816765	4.184483	-1.665435
H	1.971481	3.905123	0.284617
C	2.038485	2.695385	-3.400269
H	0.574216	1.240685	-2.808875
C	2.852014	3.755654	-2.992888
H	3.465625	4.990218	-1.335108
H	2.069257	2.343382	-4.427443
H	3.520387	4.234036	-3.702984
C	-1.679171	2.474564	-0.349364
C	-2.004850	3.008669	-1.604710
C	-2.637381	2.509392	0.678158
C	-3.260713	3.578934	-1.822764
H	-1.278607	2.998034	-2.411000
C	-3.885789	3.092045	0.458040
H	-2.404437	2.102187	1.657564
C	-4.201931	3.626814	-0.792874
H	-3.496676	3.995244	-2.798086
H	-4.609941	3.130764	1.267184
H	-5.174958	4.078548	-0.962731
C	0.294349	2.164893	1.695695
C	0.225373	3.511321	2.096485
C	0.568451	1.182476	2.657251
C	0.480810	3.864729	3.421025
H	-0.041358	4.281847	1.379555
C	0.815080	1.536282	3.985632
H	0.592442	0.136756	2.373645
C	0.782840	2.878792	4.366513
H	0.435576	4.908678	3.716921
H	1.031901	0.761188	4.714998
H	0.978536	3.157508	5.397875
C	3.495780	0.273275	0.147604
C	4.201112	0.647845	-1.005596
C	3.669011	1.021641	1.324385
C	5.068782	1.740339	-0.977128
H	4.086239	0.089692	-1.927764
C	4.536470	2.113588	1.347461
H	3.141362	0.748897	2.230787
C	5.241264	2.474667	0.197427
H	5.605166	2.019036	-1.879180

H	4.660413	2.677968	2.267373
H	5.915814	3.325864	0.214645
C	2.947015	-2.283129	-1.148029
C	3.672952	-3.441183	-0.836577
C	2.654459	-2.001063	-2.495562
C	4.104532	-4.295957	-1.854879
H	3.899480	-3.687026	0.195137
C	3.098585	-2.849633	-3.509358
H	2.075182	-1.118532	-2.754975
C	3.823000	-4.002081	-3.190332
H	4.661911	-5.192654	-1.599401
H	2.870528	-2.614872	-4.545065
H	4.160385	-4.668956	-3.978447
C	2.593669	-1.945737	1.751309
C	1.502055	-2.434578	2.482644
C	3.889466	-2.086238	2.279176
C	1.696073	-3.057333	3.717546
H	0.497403	-2.318955	2.092149
C	4.082236	-2.712137	3.510343
H	4.744344	-1.699099	1.733420
C	2.986477	-3.197561	4.231652
H	0.840187	-3.425706	4.275400
H	5.087109	-2.816331	3.908916
H	3.139756	-3.678522	5.193354
C	-4.341992	-0.802878	-1.026595
H	-2.829600	-0.606851	0.436338
F	-4.561258	-1.477356	-2.211640
F	-4.316459	0.538513	-1.351001
C	-5.489077	-1.053660	-0.080045
C	-5.687589	-0.194334	1.006345
C	-6.344217	-2.146136	-0.230410
H	-5.042953	0.671608	1.135093
C	-7.355436	-2.331942	0.710770
H	-6.221215	-2.827693	-1.064946
C	-7.465368	-1.420459	1.762463
H	-8.045661	-3.165510	0.632767
H	-8.242214	-1.542666	2.514555
N	-6.652607	-0.363385	1.917830

11. ***c*-int1**

110

Pd	-0.023097	0.288644	-0.386926
C	1.437550	1.517951	-1.548045
C	0.130499	2.016906	-1.713476
C	2.327188	2.093173	-0.444599
H	1.928663	1.027289	-2.380265
H	-0.428762	1.811188	-2.623280

C	1.732632	3.406980	0.093528
C	0.266975	3.210180	0.516332
H	2.325369	3.774530	0.936490
H	1.783045	4.158868	-0.702910
H	-0.202474	4.187696	0.685318
H	0.207917	2.685966	1.478534
P	-1.693318	-0.152414	1.246522
P	0.979923	-1.831951	-0.849225
C	-0.530897	2.482139	-0.553975
H	-1.614408	2.611601	-0.626858
C	-4.878598	1.907148	-1.258691
C	-3.708460	3.898178	-1.473567
C	-5.849362	2.336106	-2.370311
H	-5.376785	2.212550	-0.294283
H	-4.834653	0.809178	-1.239896
C	-4.650757	4.363456	-2.595235
H	-4.139599	4.336446	-0.528462
H	-2.728318	4.387961	-1.616766
H	-6.875699	1.980661	-2.197963
H	-5.494442	1.933927	-3.337890
H	-4.823314	5.449563	-2.584210
H	-4.217641	4.084247	-3.573801
N	-3.561291	2.466871	-1.417479
O	-5.942998	3.763608	-2.453024
C	-0.212019	-3.236598	-0.911381
C	-0.598810	-3.868030	0.282544
C	-0.840089	-3.607250	-2.111269
C	-1.601979	-4.837040	0.276394
H	-0.115361	-3.613083	1.218011
C	-1.845680	-4.576096	-2.111662
H	-0.552996	-3.143533	-3.048811
C	-2.234184	-5.189086	-0.918286
H	-1.890403	-5.308603	1.211331
H	-2.323457	-4.850412	-3.047938
H	-3.020713	-5.938249	-0.920217
C	1.745994	-1.824853	-2.525663
C	2.986400	-2.420463	-2.793590
C	1.059051	-1.178406	-3.569192
C	3.527400	-2.371437	-4.081207
H	3.537692	-2.918254	-2.002947
C	1.595063	-1.143624	-4.856584
H	0.106081	-0.693016	-3.373486
C	2.834159	-1.737598	-5.114303
H	4.492672	-2.831123	-4.273812
H	1.050491	-0.643923	-5.652521
H	3.258135	-1.701271	-6.113668
C	2.313932	-2.410422	0.272987
C	3.035258	-1.461338	1.011634
C	2.648072	-3.768115	0.402227
C	4.061462	-1.853991	1.871269

H	2.778359	-0.412179	0.931408
C	3.673707	-4.162378	1.263603
H	2.103064	-4.519592	-0.159969
C	4.380214	-3.208400	2.000916
H	4.600707	-1.106671	2.446493
H	3.918436	-5.216208	1.359187
H	5.173013	-3.519015	2.675082
C	-1.138394	-1.414919	2.465659
C	-1.986387	-2.405488	2.981121
C	0.212809	-1.408057	2.845608
C	-1.481598	-3.384246	3.839918
H	-3.032148	-2.433595	2.694446
C	0.717631	-2.386254	3.702553
H	0.878647	-0.648488	2.449412
C	-0.129368	-3.383288	4.194663
H	-2.144067	-4.156130	4.221371
H	1.772582	-2.380651	3.960378
H	0.262570	-4.157079	4.848533
C	-3.288611	-0.786241	0.612435
C	-4.483647	-0.642531	1.335256
C	-3.301729	-1.480904	-0.605451
C	-5.667570	-1.200107	0.850439
H	-4.490623	-0.093264	2.271531
C	-4.485592	-2.042790	-1.084648
H	-2.387472	-1.590676	-1.176860
C	-5.670214	-1.904796	-0.356949
H	-6.587853	-1.082960	1.415725
H	-4.477391	-2.587186	-2.024395
H	-6.593325	-2.338955	-0.730758
C	-2.130488	1.303267	2.287682
C	-2.908177	2.331141	1.722182
C	-1.645895	1.438187	3.597664
C	-3.188731	3.473749	2.474538
H	-3.239769	2.266093	0.677093
C	-1.929765	2.589356	4.337993
H	-1.052007	0.650675	4.049877
C	-2.702171	3.609230	3.778883
H	-3.788183	4.264503	2.031684
H	-1.549577	2.682358	5.351721
H	-2.923756	4.503472	4.355447
C	3.758911	2.244237	-0.966543
F	3.763751	3.125360	-2.025502
F	4.149558	1.015725	-1.480262
C	4.769619	2.674136	0.065721
C	5.319490	1.724740	0.933677
C	5.138720	4.011251	0.219825
H	5.067109	0.673663	0.817336
C	6.031127	4.339285	1.238834
H	4.738838	4.770444	-0.443847
C	6.512257	3.318154	2.061078

H	6.348615	5.364920	1.395316
H	7.203896	3.545867	2.869581
N	6.173355	2.027212	1.918452
H	2.410730	1.395851	0.397691

12. ***c*-ts1**

110

Pd	0.084413	0.063328	-0.493906
C	-1.753349	-0.799966	-1.329883
C	-0.736796	-1.797751	-1.307516
C	-2.935295	-0.884478	-0.361754
H	-1.975035	-0.327704	-2.283476
H	-0.159079	-2.027658	-2.199129
C	-2.845519	-2.125280	0.550197
C	-1.415768	-2.379340	1.045320
H	-3.528889	-2.018750	1.398272
H	-3.166081	-3.004052	-0.021951
H	-1.376732	-3.284450	1.657989
H	-1.111014	-1.552473	1.704724
C	-0.465513	-2.509503	-0.114676
H	0.358352	-3.181804	-0.079283
C	1.201730	-4.941315	-0.785432
C	-0.498693	-5.580473	0.686487
C	1.813543	-6.347388	-0.703653
H	1.754059	-4.319561	-0.021165
H	1.449947	-4.501805	-1.760915
C	0.098922	-6.997596	0.771899
H	-0.051215	-5.009682	1.537419
H	-1.581571	-5.639841	0.874057
H	2.907325	-6.311045	-0.771755
H	1.426739	-6.965703	-1.531735
H	-0.043569	-7.439262	1.765853
H	-0.387920	-7.645128	0.022554
N	-0.241809	-4.973646	-0.599008
O	1.507683	-6.965294	0.546364
C	-4.247341	-0.925045	-1.157270
H	-2.996282	0.022596	0.252006
F	-4.201170	-1.975481	-2.053532
F	-4.332775	0.222218	-1.931396
C	-5.498825	-1.039252	-0.320266
C	-5.898150	0.036157	0.481392
C	-6.257422	-2.210139	-0.285625
H	-5.331481	0.963113	0.470083
C	-7.378204	-2.253581	0.542322
H	-5.975336	-3.062804	-0.893098
C	-7.690410	-1.126377	1.303846
H	-7.997706	-3.142797	0.598301

H	-8.556967	-1.131935	1.962229
N	-6.970852	0.006748	1.281896
P	-0.391942	2.309330	-0.005750
P	2.426832	-0.223030	-0.033428
C	0.174416	3.533188	-1.260156
C	-0.350858	4.835325	-1.329104
C	1.185141	3.158326	-2.157638
C	0.140253	5.745005	-2.266608
H	-1.144841	5.137899	-0.652782
C	1.679216	4.071309	-3.091439
H	1.594017	2.155167	-2.120108
C	1.158803	5.365934	-3.147015
H	-0.272132	6.749277	-2.309417
H	2.471801	3.767755	-3.769051
H	1.540696	6.076692	-3.874645
C	-2.150607	2.802535	0.235881
C	-2.657981	3.244947	1.466407
C	-3.012487	2.729979	-0.871168
C	-3.998235	3.626363	1.580664
H	-2.007137	3.308689	2.332652
C	-4.343998	3.125614	-0.759193
H	-2.640032	2.363293	-1.823218
C	-4.840474	3.579593	0.467718
H	-4.378437	3.970723	2.538434
H	-4.998345	3.059704	-1.623215
H	-5.880187	3.881553	0.556600
C	0.420414	2.834911	1.564861
C	0.574440	1.865095	2.569005
C	0.951165	4.116802	1.771376
C	1.264291	2.159631	3.745567
H	0.186451	0.863097	2.413384
C	1.642743	4.411537	2.948369
H	0.853691	4.879064	1.005640
C	1.808054	3.433308	3.933487
H	1.398878	1.385905	4.496042
H	2.063147	5.403353	3.089822
H	2.361896	3.661481	4.839785
C	3.519172	1.209728	-0.436114
C	3.559778	2.304079	0.445675
C	4.199483	1.303330	-1.660180
C	4.253189	3.464847	0.105247
H	3.055147	2.250607	1.403324
C	4.890007	2.469610	-1.999686
H	4.190714	0.471461	-2.356791
C	4.915337	3.555390	-1.121976
H	4.266558	4.299648	0.800435
H	5.409457	2.525529	-2.952457
H	5.448548	4.463016	-1.390407
C	3.129079	-1.595808	-1.046437
C	4.050698	-2.525388	-0.544355

C	2.705194	-1.711456	-2.381918
C	4.541606	-3.545762	-1.363432
H	4.386437	-2.458829	0.485447
C	3.209587	-2.720080	-3.203582
H	1.977950	-1.007324	-2.778586
C	4.127349	-3.643241	-2.693492
H	5.249627	-4.264026	-0.959396
H	2.876994	-2.791737	-4.235338
H	4.510789	-4.437851	-3.327237
C	2.891068	-0.655757	1.694970
C	4.137183	-0.334783	2.256353
C	1.952168	-1.344847	2.477661
C	4.425322	-0.677384	3.579121
H	4.880342	0.192509	1.666968
C	2.242142	-1.692837	3.797384
H	0.989998	-1.604256	2.052855
C	3.478194	-1.352638	4.353763
H	5.390585	-0.415824	4.003572
H	1.500768	-2.220890	4.390583
H	3.702836	-1.612984	5.384258

13. ***c*-int2**

110

Pd	-0.298786	-0.011803	-0.271546
C	1.815579	0.302337	-0.975077
C	1.203253	1.539408	-0.739702
C	2.952068	-0.250903	-0.118839
H	1.775127	-0.099835	-1.985301
H	0.748194	2.050896	-1.582554
C	3.387337	0.737188	0.983113
C	2.186521	1.503735	1.544141
H	3.915123	0.201485	1.778861
H	4.088654	1.463489	0.556744
H	2.470649	2.076479	2.430844
H	1.418216	0.791623	1.871805
P	-2.211302	1.339521	0.041128
P	-0.767176	-2.302971	-0.059681
C	1.557938	2.405466	0.465297
H	0.633957	2.849017	0.880840
C	1.720299	4.480216	-0.794742
C	3.053748	4.253569	1.162629
C	2.636773	5.598400	-1.274355
H	0.874090	4.924086	-0.237119
H	1.298776	3.978288	-1.667459
C	3.943158	5.382509	0.644368
H	2.278736	4.677717	1.832119
H	3.682591	3.586851	1.758088

H	2.065293	6.335834	-1.846571
H	3.424941	5.180951	-1.923198
H	4.344714	5.962302	1.481188
H	4.785822	4.948032	0.080454
N	2.456622	3.518304	0.040902
O	3.225780	6.294485	-0.179394
C	-2.020806	-2.872542	-1.297386
C	-2.954066	-3.892170	-1.060381
C	-2.046560	-2.207618	-2.535258
C	-3.892994	-4.234208	-2.036264
H	-2.967526	-4.412177	-0.108534
C	-2.975043	-2.559419	-3.515857
H	-1.346379	-1.396101	-2.719467
C	-3.905382	-3.571305	-3.265969
H	-4.617012	-5.018729	-1.832906
H	-2.980391	-2.034060	-4.467079
H	-4.639067	-3.837297	-4.021901
C	0.617797	-3.505696	-0.302783
C	0.825809	-4.173172	-1.519274
C	1.543818	-3.697336	0.738779
C	1.922829	-5.023302	-1.685174
H	0.124761	-4.040953	-2.338018
C	2.626615	-4.562080	0.578649
H	1.403995	-3.187651	1.688739
C	2.821522	-5.228268	-0.636013
H	2.065181	-5.536188	-2.632640
H	3.324225	-4.708939	1.398813
H	3.667679	-5.897919	-0.761745
C	-1.438948	-2.864549	1.571193
C	-1.465537	-4.213797	1.967308
C	-1.939823	-1.895761	2.453494
C	-2.002127	-4.579843	3.202583
H	-1.055787	-4.979384	1.315107
C	-2.476411	-2.260357	3.690904
H	-1.900474	-0.849420	2.175757
C	-2.512209	-3.604521	4.066371
H	-2.018470	-5.626760	3.493050
H	-2.854808	-1.490125	4.357807
H	-2.925938	-3.892942	5.028747
C	-3.810076	0.731406	-0.662980
C	-4.604532	1.470304	-1.551976
C	-4.227537	-0.560013	-0.297847
C	-5.781401	0.922922	-2.073342
H	-4.310027	2.473378	-1.842445
C	-5.405054	-1.101106	-0.809712
H	-3.624524	-1.151097	0.382999
C	-6.183784	-0.362493	-1.706110
H	-6.383182	1.506541	-2.765026
H	-5.699173	-2.107519	-0.527548
H	-7.095525	-0.787800	-2.116590

C	-2.032826	3.013469	-0.717547
C	-2.210354	4.209613	-0.008218
C	-1.638648	3.079299	-2.066553
C	-1.995453	5.442589	-0.632206
H	-2.516408	4.184133	1.032731
C	-1.439824	4.309251	-2.692541
H	-1.484942	2.159393	-2.626304
C	-1.612103	5.496954	-1.973388
H	-2.131357	6.360589	-0.066755
H	-1.139846	4.341070	-3.736418
H	-1.445191	6.455895	-2.455921
C	-2.673406	1.716334	1.788898
C	-1.656840	1.721965	2.757950
C	-3.992763	1.981602	2.187738
C	-1.950962	1.984204	4.097173
H	-0.635407	1.502342	2.461836
C	-4.287981	2.238649	3.528335
H	-4.791283	1.981759	1.451809
C	-3.269204	2.239211	4.485608
H	-1.154057	1.979098	4.835789
H	-5.314252	2.437236	3.825103
H	-3.502091	2.434201	5.528730
C	4.136076	-0.605803	-1.027850
H	2.658489	-1.202792	0.341590
F	4.495400	0.505905	-1.766725
F	3.705244	-1.552769	-1.947432
C	5.360451	-1.140931	-0.326887
C	5.427388	-2.492375	0.024748
C	6.424834	-0.309508	0.027851
H	4.622235	-3.162336	-0.259682
C	7.496151	-0.861704	0.727455
H	6.414378	0.740476	-0.243751
C	7.458380	-2.221278	1.044074
H	8.345259	-0.253325	1.021710
H	8.277723	-2.678442	1.595652
N	6.449019	-3.035740	0.699586

14. ***c*19-product**

41

C	0.171518	-2.227434	-0.130116
C	1.461054	-2.100183	-0.453515
C	-0.893598	-1.227085	-0.514542
H	-0.164091	-3.097736	0.428597
H	2.164085	-2.869709	-0.141701
C	-0.286411	0.060963	-1.094645
C	0.869967	-0.270695	-2.041534
H	-1.058444	0.643852	-1.606798

H	0.107152	0.674084	-0.276062
H	1.225937	0.641563	-2.530638
H	0.527036	-0.945443	-2.836928
C	2.015943	-0.952325	-1.269675
D	2.730334	-1.387162	-1.993589
C	3.378643	-0.469784	0.769179
C	3.697114	0.818830	-1.216269
C	3.955947	0.690601	1.576139
H	4.192821	-1.180490	0.527235
H	2.643200	-0.995918	1.384316
C	4.270497	1.952156	-0.371028
H	4.527371	0.174698	-1.566837
H	3.212225	1.243234	-2.101022
H	4.498330	0.317611	2.450205
H	3.132354	1.338519	1.919189
H	5.044248	2.492610	-0.924672
H	3.464611	2.657007	-0.107920
N	2.723478	0.051446	-0.433536
O	4.888795	1.451927	0.812160
C	-1.803678	-0.932746	0.684196
D	-1.551168	-1.689708	-1.266510
F	-1.037194	-0.433251	1.718279
F	-2.326189	-2.133342	1.139439
C	-2.948326	0.010462	0.406139
C	-2.869313	1.365354	0.739767
C	-4.101184	-0.450025	-0.234274
H	-1.986718	1.747860	1.245126
C	-5.116677	0.460140	-0.514750
H	-4.200526	-1.498675	-0.496900
C	-4.936914	1.794587	-0.143525
H	-6.030772	0.145779	-1.007776
H	-5.711855	2.529841	-0.351282
N	-3.838567	2.251875	0.475389

X. The Single Crystal Data

10.1 The Single Crystal Data of 52

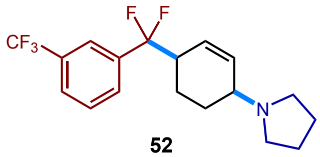

52

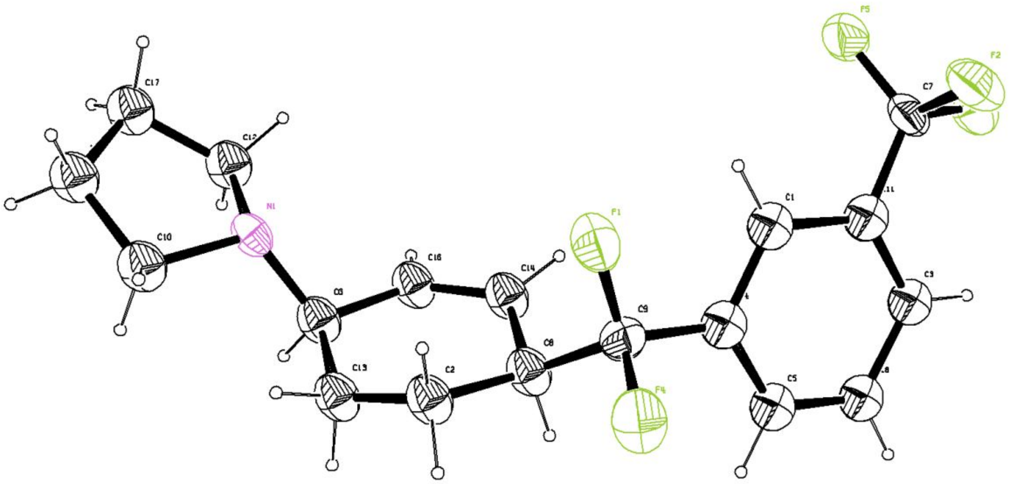

Table S26. Crystal data and structure refinement for 2023040604_0m.

Identification code	2023040604_0m
Empirical formula	$C_{18}H_{20}F_5N$
Formula weight	345.358
Temperature/K	295.00
Crystal system	monoclinic
Space group	$P2_1/c$
a/Å	10.1884(8)
b/Å	6.9129(5)
c/Å	25.659(2)
α/°	90
β/°	97.347(4)
γ/°	90
Volume/Å^3	1792.4(2)
Z	4
ρ_{calc}g/cm^3	1.280
μ/mm^{-1}	0.958
F(000)	723.0

Crystal size/mm^3	0.14 × 0.12 × 0.1
Radiation	Cu Kα (λ = 1.54184)
2Θ range for data collection/°	6.94 to 136.86
Index ranges	-11 ≤ h ≤ 12, -8 ≤ k ≤ 6, -30 ≤ l ≤ 30
Reflections collected	14039
Independent reflections	3288 [R_{int} = 0.1676, R_{sigma} = 0.1370]
Data/restraints/parameters	3288/105/217
Goodness-of-fit on F^2	2.364
Final R indexes [I>=2σ (I)]	R_1 = 0.4572, wR_2 = 0.7762
Final R indexes [all data]	R_1 = 0.5787, wR_2 = 0.8204
Largest diff. peak/hole / e Å^{-3}	6.70/-1.39

Table S27. Fractional Atomic Coordinates (×10^4) and Equivalent Isotropic Displacement Parameters (Å^2×10^3) for 2023040604_0m. U_{eq} is defined as 1/3 of the trace of the orthogonalised U_{IJ} tensor.

Atom	*x*	*y*	*z*	U(eq)
F1	5980(50)	2670(60)	3701(18)	350(20)
F4	4540(20)	3660(50)	3348(10)	264(15)
N1	7800(20)	7260(40)	5151(10)	113(8)
F5	9700(20)	-290(30)	2945(11)	197(12)
F3	9590(20)	-230(30)	2148(11)	175(11)
F2	8380(30)	-1940(30)	2501(10)	195(11)
C1	7320(40)	1900(50)	2961(15)	131(6)
C4	6480(40)	3310(60)	3015(17)	131(7)
C6	7380(30)	8230(50)	4661(13)	112(5)
C8	6170(30)	5850(50)	3730(13)	112(5)
C10	7180(40)	8120(50)	5636(19)	164(9)
C12	9320(30)	8100(50)	5443(16)	163(9)
C14	7670(30)	6270(40)	3853(13)	112(5)
C16	8250(40)	7400(40)	4252(12)	112(5)
C18	6830(30)	3730(50)	2065(15)	130(6)
C9	5980(40)	4320(80)	3407(18)	161(17)
C5	6120(40)	4540(50)	2531(13)	131(6)
C11	7670(40)	1560(50)	2439(16)	130(6)
C3	7250(40)	2510(50)	2046(16)	130(6)
C13	6080(30)	7710(40)	4468(12)	112(5)
C7	8780(50)	-100(60)	2550(20)	146(15)
C15	8080(30)	8140(50)	6036(18)	164(9)
C2	5440(30)	5990(40)	4136(11)	113(5)
C17	9240(40)	7510(50)	6013(18)	163(9)

Table S28. Anisotropic Displacement Parameters (Å^2×10^3) for 2023040604_0m. The Anisotropic displacement factor exponent takes the form: -2π^2[h^{2}a*^{2}U$_{11}$+2hka*b*U$_{12}$+…].

Atom	U_{11}	U_{22}	U_{33}	U_{12}	U_{13}	U_{23}
F1	470(60)	310(40)	300(40)	-270(40)	190(40)	-200(30)
F4	114(17)	490(40)	180(20)	-60(20)	-17(16)	40(20)
N1	51(13)	210(30)	86(14)	-7(15)	33(12)	-25(13)
F5	180(20)	160(20)	230(30)	96(17)	-72(19)	-66(17)
F3	180(20)	125(17)	240(30)	40(14)	110(20)	-12(15)
F2	280(30)	115(15)	190(20)	-12(18)	60(20)	13(16)
C1	162(16)	94(12)	135(14)	-27(11)	12(11)	4(11)
C4	162(16)	94(12)	135(14)	-27(11)	12(11)	5(11)
C6	135(12)	96(9)	115(11)	7(9)	55(9)	-17(7)
C8	135(12)	94(9)	117(11)	6(9)	54(9)	-16(7)
C10	89(15)	117(13)	280(30)	-4(12)	0(16)	-11(15)
C12	90(15)	114(13)	280(30)	-2(12)	1(16)	-12(15)
C14	135(12)	94(9)	117(11)	6(9)	54(9)	-16(7)
C16	135(12)	96(9)	115(11)	6(9)	54(9)	-16(7)
C18	162(16)	93(12)	134(14)	-28(11)	12(11)	5(11)
C9	140(20)	160(40)	170(40)	-80(30)	10(30)	40(40)
C5	163(16)	92(12)	135(14)	-27(11)	12(11)	5(11)
C11	162(16)	94(12)	134(14)	-27(11)	12(11)	4(11)
C3	161(16)	94(12)	133(14)	-27(11)	12(11)	4(11)
C13	136(12)	96(9)	115(11)	7(9)	55(9)	-17(7)
C7	150(40)	101(19)	190(40)	40(20)	0(30)	0(20)
C15	91(15)	117(13)	280(30)	-4(12)	1(16)	-11(15)
C2	137(12)	95(9)	117(11)	7(9)	55(9)	-16(7)
C17	90(15)	115(13)	280(30)	-3(12)	1(16)	-11(15)

Table S29. Bond Lengths for 2023040604_0m.

Atom	Atom	Length/Å	Atom	Atom	Length/Å
F1	C9	1.37(5)	C6	C13	1.40(4)
F4	C9	1.53(4)	C8	C14	1.55(4)
N1	C6	1.44(3)	C8	C9	1.34(5)
N1	C10	1.58(4)	C8	C2	1.36(3)
N1	C12	1.74(4)	C10	C15	1.29(4)
F5	C7	1.29(4)	C12	C17	1.53(5)
F3	C7	1.42(5)	C14	C16	1.36(4)
F2	C7	1.34(4)	C18	C5	1.58(5)
C1	C4	1.31(5)	C18	C3	0.94(4)
C1	C11	1.45(4)	C11	C3	1.24(4)
C4	C9	1.37(5)	C11	C7	1.61(5)
C4	C5	1.51(4)	C13	C2	1.56(4)
C6	C16	1.57(4)	C15	C17	1.27(4)

Table S30. Bond Angles for 2023040604_0m.

Atom	Atom	Atom	Angle/°	Atom	Atom	Atom	Angle/°
C10	N1	C6	114(3)	C4	C9	F4	102(4)
C12	N1	C6	112(3)	C8	C9	F1	109(4)
C12	N1	C10	88(2)	C8	C9	F4	111(4)
C11	C1	C4	117(4)	C8	C9	C4	145(4)
C9	C4	C1	139(5)	C18	C5	C4	109(3)
C5	C4	C1	115(4)	C3	C11	C1	124(4)
C5	C4	C9	104(4)	C7	C11	C1	101(4)
C16	C6	N1	106(3)	C7	C11	C3	134(4)
C13	C6	N1	111(3)	C11	C3	C18	123(6)
C13	C6	C16	105(3)	C2	C13	C6	134(3)
C9	C8	C14	109(3)	F3	C7	F5	97(4)
C2	C8	C14	117(3)	F2	C7	F5	99(3)
C2	C8	C9	119(4)	F2	C7	F3	93(3)
C15	C10	N1	108(3)	C11	C7	F5	130(4)
C17	C12	N1	100(3)	C11	C7	F3	112(4)
C16	C14	C8	126(3)	C11	C7	F2	118(4)
C14	C16	C6	119(3)	C17	C15	C10	122(5)
C3	C18	C5	128(5)	C13	C2	C8	104(3)
F4	C9	F1	74(3)	C15	C17	C12	97(4)
C4	C9	F1	90(4)				

Table S31. Torsion Angles for 2023040604_0m.

A	B	C	D	Angle/°	A	B	C	D	Angle/°
F1	C9	C4	C1	-37(5)	F3	C7	C11	C1	-160(3)
F1	C9	C4	C5	158(3)	F3	C7	C11	C3	13(5)
F1	C9	C8	C14	91(4)	F2	C7	C11	C1	93(4)
F1	C9	C8	C2	-47(5)	F2	C7	C11	C3	-93(5)
F4	C9	C4	C1	-112(3)	C1	C4	C9	C8	88(6)
F4	C9	C4	C5	84(3)	C1	C4	C5	C18	2(3)
F4	C9	C8	C14	171(3)	C1	C11	C3	C18	16(4)
F4	C9	C8	C2	33(4)	C4	C9	C8	C14	-29(8)
N1	C6	C16	C14	112(3)	C4	C9	C8	C2	-168(7)
N1	C6	C13	C2	-84(3)	C4	C5	C18	C3	14(4)
N1	C10	C15	C17	-1(4)	C6	C16	C14	C8	5(3)
N1	C12	C17	C15	-47(3)	C6	C13	C2	C8	-48(4)
F5	C7	C11	C1	-39(6)	C10	C15	C17	C12	33(4)
F5	C7	C11	C3	134(5)	C18	C3	C11	C7	-157(5)

Table S32. Hydrogen Atom Coordinates (Å×10[4]) and Isotropic Displacement Parameters (Å[2]×10[3]) for 2023040604_0m.

Atom	*x*	*y*	*z*	U(eq)
H1	7660(40)	1140(50)	3246(15)	157(8)

H6	7480(30)	9630(50)	4696(13)	135(6)
H8	5840(30)	6930(50)	3503(13)	134(6)
H10a	6860(40)	9420(50)	5558(19)	197(11)
H10b	6440(40)	7330(50)	5711(19)	197(11)
H12a	10050(30)	7460(50)	5302(16)	196(11)
H12b	9410(30)	9490(50)	5407(16)	196(11)
H14	8220(30)	5710(40)	3633(13)	134(6)
H16	9160(40)	7660(40)	4283(12)	135(6)
H18	6840(30)	4540(50)	1777(15)	156(8)
H5	5560(40)	5610(50)	2511(13)	157(8)
H3	7350(40)	2020(50)	1716(16)	156(8)
H13a	5740(30)	8840(40)	4271(12)	135(6)
H13b	5630(30)	7700(40)	4780(12)	135(6)
H15a	7730(30)	7420(50)	6313(18)	197(11)
H15b	8170(30)	9470(50)	6154(18)	197(11)
H2a	5490(30)	4810(40)	4341(11)	136(6)
H2b	4520(30)	6260(40)	4009(11)	136(6)
H17a	9900(40)	8160(50)	6259(18)	196(11)
H17b	9310(40)	6120(50)	6064(18)	196(11)

Table S33. Crystal data and structure refinement for 2023040604_0m.

Identification code	2023040604_0m
Empirical formula	$C_{18}H_{20}F_5N$
Formula weight	345.358
Temperature/K	295.00
Crystal system	monoclinic
Space group	$P2_1/c$
a/Å	10.1884(8)
b/Å	6.9129(5)
c/Å	25.659(2)
α/°	90
β/°	97.347(4)
γ/°	90
Volume/Å^3	1792.4(2)
Z	4
ρ_{calc}g/cm^3	1.280
μ/mm^{-1}	0.958
F(000)	723.0
Crystal size/mm^3	0.14 × 0.12 × 0.1
Radiation	Cu Kα (λ = 1.54184)
2Θ range for data collection/°	6.94 to 136.86
Index ranges	-11 ≤ h ≤ 12, -8 ≤ k ≤ 6, -30 ≤ l ≤ 30
Reflections collected	14039

Independent reflections	3288 [R_{int} = 0.1676, R_{sigma} = 0.1370]
Data/restraints/parameters	3288/105/217
Goodness-of-fit on F^2	2.364
Final R indexes [I>=2σ (I)]	R_1 = 0.4572, wR_2 = 0.7762
Final R indexes [all data]	R_1 = 0.5787, wR_2 = 0.8204
Largest diff. peak/hole / e Å^{-3}	6.70/-1.39

10.2 The Single Crystal Data of 57-a

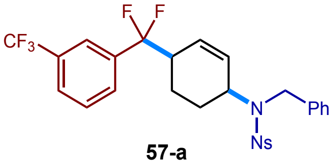

57-a

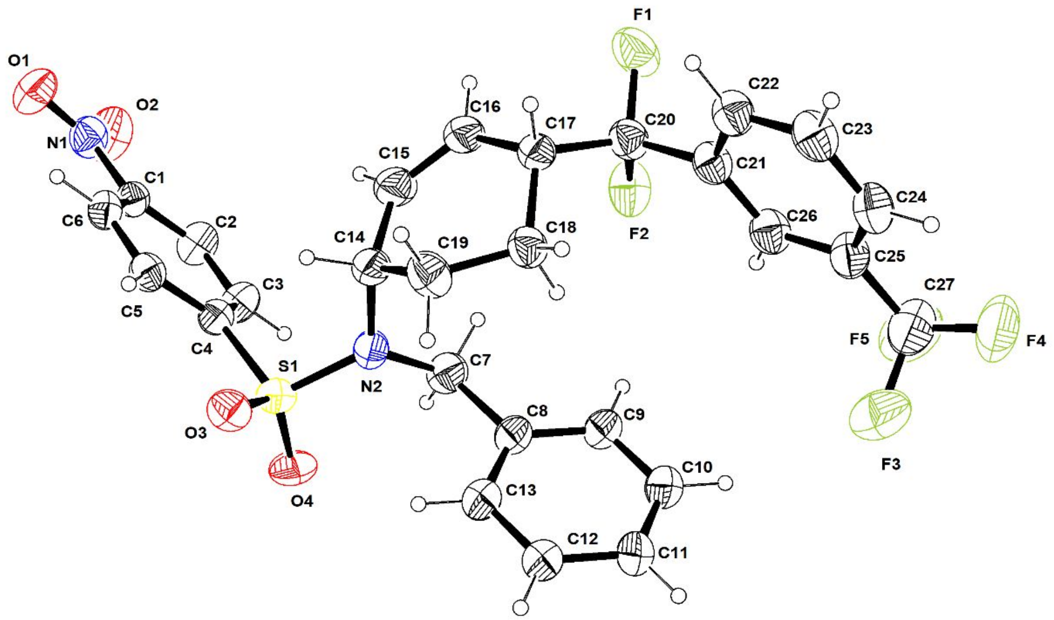

Table S34. Crystal data and structure refinement for 2023031601_0m.

2023031601_0m

Identification code	2023031601_0m
Empirical formula	$C_{27}H_{23}F_5N_2O_4S$

Formula weight	566.53
Temperature/K	150.00
Crystal system	monoclinic
Space group	$P2_1$
a/Å	11.2825(4)
b/Å	9.5421(3)
c/Å	12.7830(4)
α/°	90
β/°	106.155(2)
γ/°	90
Volume/Å^3	1321.86(8)
Z	2
ρ_{calc}g/cm^3	1.423
μ/mm^{-1}	1.735
F(000)	584.0
Crystal size/mm^3	0.18 × 0.17 × 0.15
Radiation	CuKα (λ = 1.54178)
2Θ range for data collection/°	7.2 to 136.49
Index ranges	$-13 \leq h \leq 13$, $-10 \leq k \leq 11$, $-15 \leq l \leq 15$
Reflections collected	8829
Independent reflections	4327 [R_{int} = 0.0368, R_{sigma} = 0.0499]
Data/restraints/parameters	4327/272/412
Goodness-of-fit on F^2	1.052
Final R indexes [I>=2σ (I)]	R_1 = 0.0434, wR_2 = 0.0940
Final R indexes [all data]	R_1 = 0.0588, wR_2 = 0.1056
Largest diff. peak/hole / e Å^{-3}	0.14/-0.18
Flack parameter	0.34(3)

Table S35. Fractional Atomic Coordinates (×10^4) and Equivalent Isotropic Displacement Parameters (Å^2×10^3) for 2023031601_0m. U_{eq} is defined as 1/3 of the trace of the orthogonalised U_{IJ} tensor.

Atom	*x*	*y*	*z*	U(eq)
S1	8873.4(10)	5468.7(13)	9023.7(9)	66.2(3)
O3	10084(3)	5975(4)	9096(3)	77.0(9)
F2	5639(3)	3503(4)	4484(3)	99.4(10)
O4	8706(3)	4414(4)	9763(3)	84.2(10)
F1	5341(3)	4975(4)	3129(3)	114.2(13)
N1	5775(4)	10423(6)	9329(3)	81.9(11)
N2	8283(3)	4880(4)	7802(3)	69.6(10)
C1	6544(4)	9187(5)	9264(3)	61.7(11)
O1	6151(4)	11577(5)	9196(4)	107.5(14)
O2	4785(4)	10208(5)	9495(4)	124.2(16)
C6	7705(4)	9420(5)	9173(4)	67.5(12)

C8	8174(13)	2316(13)	7374(15)	72(3)
C13	9427(13)	2161(13)	7881(14)	81(4)
C12	10048(13)	976(14)	7681(13)	83(4)
C11	9414(19)	-53(10)	6975(12)	84(4)
C10	8160(20)	101(10)	6469(9)	83(4)
C9	7540(14)	1286(14)	6669(13)	76(3)
C20	6239(4)	4188(6)	3829(4)	74.2(13)
C21	6702(4)	3120(6)	3176(4)	70.3(12)
C5	8434(4)	8268(5)	9122(3)	65.8(11)
C4	7971(4)	6950(5)	9143(3)	59.5(10)
C14	8328(5)	5821(5)	6885(3)	73.9(13)
C17	7182(4)	5173(5)	4528(4)	68.0(12)
C18	8302(4)	4374(6)	5215(4)	72.0(12)
C16	6604(5)	6084(6)	5209(4)	80.9(14)
C22	7248(4)	3570(6)	2383(4)	77.5(14)
C24	7616(5)	1188(7)	1966(4)	82.8(15)
C15	7076(5)	6309(6)	6259(4)	86.0(16)
C25	7066(5)	728(6)	2744(4)	80.8(14)
F5	5978(9)	-1135(11)	3219(13)	159(5)
C7	7495(4)	3635(6)	7620(5)	81.5(14)
C26	6615(5)	1703(6)	3344(4)	78.8(14)
C2	6071(5)	7878(6)	9305(4)	79.2(14)
C3	6795(4)	6738(6)	9243(4)	74.6(13)
C19	9046(4)	5212(7)	6144(4)	87.9(16)
F4	7062(15)	-1602(10)	2138(8)	169(5)
C23	7706(5)	2594(7)	1787(4)	85.7(16)
C27	6939(8)	-787(9)	2915(7)	119(2)
F3	7904(9)	-1237(8)	3785(8)	149(4)

Table S36. Anisotropic Displacement Parameters (Å^2×10^3) for 2023031601_0m. The Anisotropic displacement factor exponent takes the form: $-2\pi^2[h^2a^{*2}U_{11}+2hka^*b^*U_{12}+\ldots]$.

Atom	U_{11}	U_{22}	U_{33}	U_{23}	U_{13}	U_{12}
S1	65.8(6)	69.6(7)	64.0(6)	7.3(6)	19.5(4)	7.9(6)
O3	57.2(16)	89(3)	85(2)	0.8(18)	19.5(15)	6.8(16)
F2	100(2)	114(3)	103(2)	-27.5(19)	58.4(18)	-26.9(19)
O4	99(2)	77(2)	82(2)	29.7(19)	34.5(19)	14(2)
F1	93.9(19)	146(4)	86(2)	-18.7(19)	-1.2(16)	37(2)
N1	85(3)	83(3)	80(3)	-13(3)	27(2)	7(3)
N2	83(2)	59(2)	66(2)	-5.3(18)	19.3(18)	-4(2)
C1	70(3)	61(3)	56(2)	-5(2)	21(2)	2(2)
O1	120(3)	66(3)	139(4)	-5(3)	41(3)	8(3)
O2	109(3)	115(4)	171(4)	-30(3)	77(3)	11(3)
C6	70(3)	63(3)	69(3)	-7(2)	18(2)	-8(2)
C8	81(6)	57(6)	76(6)	2(5)	20(5)	-7(5)

C13	88(6)	68(6)	85(6)	-8(5)	17(5)	9(5)
C12	87(6)	73(6)	83(7)	-5(5)	12(5)	8(5)
C11	93(8)	69(6)	82(6)	-11(5)	12(6)	-9(6)
C10	87(8)	69(6)	86(6)	-8(5)	13(5)	-8(5)
C9	86(6)	59(6)	82(6)	0(5)	22(5)	-4(5)
C20	74(3)	82(4)	67(3)	-5(3)	19(2)	5(3)
C21	69(3)	81(4)	63(3)	-4(2)	22(2)	-9(3)
C5	59(2)	72(3)	66(3)	-3(2)	15.3(19)	-6(2)
C4	64(3)	60(3)	58(2)	0(2)	22.0(19)	0(2)
C14	96(3)	65(3)	60(3)	2(2)	20(2)	7(3)
C17	83(3)	63(3)	59(2)	2(2)	20(2)	6(2)
C18	76(3)	76(3)	65(3)	-4(2)	21(2)	2(3)
C16	95(4)	72(3)	72(3)	2(3)	17(3)	22(3)
C22	80(3)	81(4)	73(3)	-4(3)	24(3)	-13(3)
C24	84(3)	94(4)	74(3)	-19(3)	28(3)	-9(3)
C15	103(4)	80(4)	76(3)	1(3)	26(3)	26(3)
C25	93(3)	78(4)	73(3)	-10(3)	28(3)	-11(3)
F5	156(7)	104(5)	229(13)	19(8)	74(8)	-26(6)
C7	71(3)	75(3)	96(3)	1(3)	19(2)	-3(3)
C26	84(4)	88(4)	70(3)	-5(3)	31(3)	-7(3)
C2	71(3)	79(4)	101(4)	-3(3)	46(3)	-3(3)
C3	77(3)	65(3)	93(3)	3(3)	43(3)	-6(3)
C19	82(3)	108(5)	74(3)	-7(3)	21(2)	3(3)
F4	273(13)	98(5)	144(7)	-43(5)	72(7)	-11(8)
C23	84(3)	111(5)	70(3)	-6(3)	34(3)	-13(3)
C27	150(6)	93(5)	128(5)	-13(5)	61(5)	-7(5)
F3	180(8)	97(5)	167(7)	29(5)	46(6)	11(5)

Table S37. Bond Lengths for 2023031601_0m.

Atom	Atom	Length/Å	Atom	Atom	Length/Å
C31	H31	0.9500	C11	C10	1.3900
C30	H30	0.9500	C10	C9	1.3900
C0	H0	0.9500	C20	C21	1.501(7)
C00X	H00X	0.9500	C20	C17	1.512(7)
C29	C0	1.3900	C21	C22	1.392(6)
C28	C29	1.3900	C21	C26	1.377(8)
C0	C30	1.3900	C5	C4	1.365(6)
C28	C00X	1.3900	C4	C3	1.382(6)
C30	C31	1.3900	C14	C15	1.492(7)
C29	H29	0.9500	C14	C19	1.522(6)
C31	C00X	1.3900	C17	C18	1.527(6)
S1	O3	1.428(3)	C17	C16	1.502(6)
S1	O4	1.428(3)	C18	C19	1.483(7)
S1	N2	1.618(4)	C16	C15	1.316(7)
S1	C4	1.774(5)	C22	C23	1.391(7)

F2	C20	1.379(6)	C24	C25	1.383(7)
F1	C20	1.373(6)	C24	C23	1.370(8)
N1	C1	1.479(7)	C25	C26	1.390(7)
N1	O1	1.209(6)	C25	C27	1.474(10)
N1	O2	1.211(5)	F5	C27	1.294(10)
N2	C14	1.489(6)	C7	C28	1.448(11)
N2	C7	1.463(6)	C7	H15D	0.9900
C1	C6	1.365(6)	C7	H15C	0.9900
C1	C2	1.366(7)	C2	C3	1.376(7)
C6	C5	1.386(7)	F4	C27	1.298(10)
C8	C13	1.3900	C27	F00Y	1.286(15)
C8	C9	1.3900	C27	F0	1.283(15)
C8	C7	1.550(9)	C27	F00Q	1.414(14)
C13	C12	1.3900	C27	F3	1.391(10)
C12	C11	1.3900			

Table S38. Bond Angles for 2023031601_0m.

Atom	Atom	Atom	Angle/°	Atom	Atom	Atom	Angle/°
O3	S1	O4	119.7(2)	F00Y	C27	C25	113.7(12)
C29	C28	C00X	120.0	F00Q	C27	C25	105.2(9)
C30	C31	C00X	120.0	C12	C11	C10	120.0
O3	S1	N2	108.0(2)	C9	C10	C11	120.0
C30	C0	H0	120.0	C10	C9	C8	120.0
O3	S1	C4	106.7(2)	F2	C20	C21	108.8(4)
C31	C00X	H00X	120.0	F2	C20	C17	108.7(4)
O4	S1	N2	107.4(2)	F1	C20	F2	104.9(4)
C0	C29	H29	120.0	F1	C20	C21	108.4(4)
O4	S1	C4	108.6(2)	F1	C20	C17	108.4(4)
C31	C30	H30	120.0	N2	C7	H15C	107.9
N2	S1	C4	105.6(2)	N2	C7	H15D	107.9
C28	C00X	H00X	120.0	C21	C20	C17	117.0(4)
O1	N1	C1	118.9(4)	C22	C21	C20	119.2(5)
C31	C30	C0	120.0	C26	C21	C20	121.9(4)
O1	N1	O2	123.8(5)	C26	C21	C22	118.9(5)
C28	C29	H29	120.0	C4	C5	C6	119.6(4)
O2	N1	C1	117.3(6)	C5	C4	S1	120.0(3)
C29	C0	C30	120.0	C5	C4	C3	121.3(4)
C14	N2	S1	117.1(3)	C3	C4	S1	118.6(4)
C0	C30	H30	120.0	N2	C14	C15	112.1(4)
C7	N2	S1	120.3(3)	N2	C14	C19	113.6(4)
C30	C31	H31	120.0	C15	C14	C19	111.9(4)
C7	N2	C14	121.1(4)	C20	C17	C18	111.3(4)
C00X	C31	H31	120.0	C16	C17	C20	110.8(4)
C6	C1	N1	117.8(5)	C16	C17	C18	112.3(4)
C31	C00X	C28	120.0	C19	C18	C17	112.6(4)

C6	C1	C2	123.1(5)	C15	C16	C17	124.6(5)
C29	C0	H0	120.0	C23	C22	C21	119.9(5)
C2	C1	N1	119.0(5)	C23	C24	C25	120.0(5)
C1	C6	C5	118.1(5)	C16	C15	C14	123.4(5)
C13	C8	C9	120.0	C24	C25	C26	119.4(5)
C13	C8	C7	119.2(8)	C24	C25	C27	119.9(5)
C0	C29	C28	120.0	C26	C25	C27	120.6(5)
C00X	C28	C7	125.0(11)	N2	C7	C8	112.2(7)
C29	C28	C7	114.8(11)	C21	C26	C25	121.2(5)
C28	C7	H15D	107.9	C1	C2	C3	118.5(4)
H15C	C7	H15D	107.2	C2	C3	C4	119.3(5)
C28	C7	H15C	107.9	C18	C19	C14	115.0(4)
C9	C8	C7	120.8(8)	C24	C23	C22	120.6(5)
C8	C13	C12	120.0	F5	C27	C25	114.8(8)
C28	C7	N2	117.5(9)	F5	C27	F4	110.6(10)
F00Y	C27	F00Q	105.1(14)	F5	C27	F3	102.4(9)
F0	C27	F00Q	99.5(15)	F4	C27	C25	116.0(7)
C11	C12	C13	120.0	F4	C27	F3	101.6(9)
F0	C27	F00Y	115.1(18)	F3	C27	C25	109.6(7)
F0	C27	C25	116.0(12)				

Table S39. Torsion Angles for 2023031601_0m.

A	B	C	D	Angle/°	A	B	C	D	Angle/°
C28	C29	C0	C30	0.0	C12	C11	C10	C9	0.0
C30	C31	C00X	C28	0.0	C11	C10	C9	C8	0.0
C0	C30	C31	C00X	0.0	C9	C8	C13	C12	0.0
C00X	C28	C29	C0	0.0	C9	C8	C7	N2	-146.5(8)
C29	C0	C30	C31	0.0	C20	C21	C22	C23	178.6(5)
C29	C28	C00X	C31	0.0	C20	C21	C26	C25	-179.0(5)
S1	N2	C14	C15	-112.2(4)	C20	C17	C18	C19	-161.9(4)
S1	N2	C14	C19	119.6(4)	C20	C17	C16	C15	132.6(6)
S1	N2	C7	C8	-104.0(9)	C21	C20	C17	C18	-49.0(6)
S1	C4	C3	C2	-178.2(4)	C21	C20	C17	C16	-174.7(4)
O3	S1	N2	C14	-50.8(4)	C21	C22	C23	C24	0.5(8)
O3	S1	N2	C7	143.2(3)	C5	C4	C3	C2	0.9(7)
O3	S1	C4	C5	9.2(4)	C4	S1	N2	C14	63.0(4)
O3	S1	C4	C3	-171.7(3)	C4	S1	N2	C7	-103.0(4)
S1	N2	C7	C28	-104.0(12)	C14	N2	C7	C8	90.5(9)
F2	C20	C21	C22	174.5(4)	C17	C20	C21	C22	-62.0(6)
F2	C20	C21	C26	-6.2(6)	C17	C20	C21	C26	117.3(5)
C14	N2	C7	C28	90.5(12)	C17	C18	C19	C14	53.9(6)
F2	C20	C17	C18	74.6(5)	C17	C16	C15	C14	7.5(9)
F2	C20	C17	C16	-51.1(5)	C18	C17	C16	C15	7.5(8)
O4	S1	N2	C14	178.8(3)	C16	C17	C18	C19	-37.1(6)
O4	S1	N2	C7	12.8(4)	C22	C21	C26	C25	0.4(8)

O4	S1	C4	C5	139.6(4)	C24	C25	C26	C21	0.2(8)
O4	S1	C4	C3	-41.3(4)	C24	C25	C27	F5	-148.5(10)
F1	C20	C21	C22	60.9(6)	C24	C25	C27	F4	-17.3(14)
F1	C20	C21	C26	-119.7(5)	C24	C25	C27	F3	96.9(8)
F1	C20	C17	C18	-172.0(4)	C15	C14	C19	C18	-38.3(7)
F1	C20	C17	C16	62.4(5)	C25	C24	C23	C22	0.1(8)
N1	C1	C6	C5	179.4(4)	C7	C28	C29	C0	175(2)
N1	C1	C2	C3	179.9(4)	C7	C28	C00X	C31	-174(2)
N2	S1	C4	C5	-105.5(4)	C7	N2	C14	C15	53.7(6)
N2	S1	C4	C3	73.6(4)	C7	N2	C14	C19	-74.4(6)
N2	C14	C15	C16	-121.5(6)	C7	C8	C13	C12	179.1(16)
N2	C14	C19	C18	90.0(5)	C7	C8	C9	C10	-179.1(16)
N2	C7	C28	C00X	26.2(18)	C26	C21	C22	C23	-0.7(7)
N2	C7	C28	C29	-148.6(8)	C26	C25	C27	F5	30.3(13)
C1	C6	C5	C4	1.1(6)	C26	C25	C27	F4	161.4(11)
C1	C2	C3	C4	0.1(7)	C26	C25	C27	F3	-84.3(9)
O1	N1	C1	C6	7.0(6)	C2	C1	C6	C5	0.0(7)
O1	N1	C1	C2	-173.5(5)	C19	C14	C15	C16	7.5(8)
O2	N1	C1	C6	-174.3(5)	C23	C24	C25	C26	-0.4(8)
O2	N1	C1	C2	5.1(6)	C24	C25	C27	F00Q	-84.0(14)
C6	C1	C2	C3	-0.6(8)	C24	C25	C27	F0	167.2(17)
C6	C5	C4	S1	177.5(3)	C24	C25	C27	F00Y	30.4(18)
C6	C5	C4	C3	-1.6(7)	C26	C25	C27	F00Y	-150.8(16)
C8	C13	C12	C11	0.0	C26	C25	C27	F00Q	94.7(14)
C13	C8	C9	C10	0.0	C26	C25	C27	F0	-14(2)
C13	C8	C7	N2	34.3(12)	C23	C24	C25	C27	178.3(6)
C13	C12	C11	C10	0.0	C27	C25	C26	C21	-178.5(6)

Table S40. Hydrogen Atom Coordinates (Å×10^4) and Isotropic Displacement Parameters (Å^2×10^3) for 2023031601_0m.

Atom	*x*	*y*	*z*	U(eq)
H6	8004.67	10346.11	9146.8	81
H13	9860.48	2865.24	8363.15	98
H12	10904.36	870.62	8027.17	100
H11	9837.68	-863.23	6838.91	101
H10	7727.11	-602.47	5986.62	100
H9	6683.2	1392.14	6322.59	91
H5	9251.27	8394.51	9071.58	79
H14	8789.58	6676.59	7224.81	89
H17	7476.89	5801.89	4026.78	82
H18A	8017.36	3508.23	5498.22	86
H18B	8828.91	4094.68	4747.89	86
H16	5844.48	6529.09	4857.61	97
H22	7307.85	4543.76	2250.03	93
H24	7932.55	529.19	1555.28	99

H15	6593.74	6808.83	6636.8	103
H7A	6757.42	3810.46	7001.56	98
H7B	7211.61	3459.25	8275.34	98
H26	6239.1	1386.55	3880.74	95
H2	5260.59	7756.41	9374.84	95
H3	6490.28	5813.77	9267.41	89
H19A	9437.84	5992.98	5853.02	106
H19B	9715.05	4612.52	6587.12	106
H23	8084.72	2905.94	1251.69	103

Table S41. Atomic Occupancy for 2023031601_0m.

Atom	*Occupancy*	Atom	*Occupancy*	Atom	*Occupancy*
C8	0.55(4)	C13	0.55(4)	H13	0.55(4)
C12	0.55(4)	H12	0.55(4)	C11	0.55(4)
H11	0.55(4)	C10	0.55(4)	H10	0.55(4)
C9	0.55(4)	H9	0.55(4)	F5	0.675(12)
H7A	0.55(4)	H7B	0.55(4)	F4	0.675(12)
F3	0.675(12)				

Table S42. Crystal data and structure refinement for 2023031601_0m.

2023031601_0m

Identification code	2023031601_0m
Empirical formula	$C_{27}H_{23}F_5N_2O_4S$
Formula weight	566.53
Temperature/K	150.00
Crystal system	monoclinic
Space group	$P2_1$
a/Å	11.2825(4)
b/Å	9.5421(3)
c/Å	12.7830(4)
α/°	90
β/°	106.155(2)
γ/°	90
Volume/Å^3	1321.86(8)
Z	2
ρ_{calc}g/cm^3	1.423
μ/mm^{-1}	1.735
F(000)	584.0
Crystal size/mm^3	0.18 × 0.17 × 0.15
Radiation	CuKα (λ = 1.54178)

2Θ range for data collection/°	7.2 to 136.49
Index ranges	$-13 \leq h \leq 13$, $-10 \leq k \leq 11$, $-15 \leq l \leq 15$
Reflections collected	8829
Independent reflections	4327 [R_{int} = 0.0368, R_{sigma} = 0.0499]
Data/restraints/parameters	4327/272/412
Goodness-of-fit on F^2	1.052
Final R indexes [I>=2σ (I)]	R_1 = 0.0434, wR_2 = 0.0940
Final R indexes [all data]	R_1 = 0.0588, wR_2 = 0.1056
Largest diff. peak/hole / e Å^{-3}	0.14/-0.18
Flack parameter	0.34(3)

10.3 The Single Crystal Data of 68

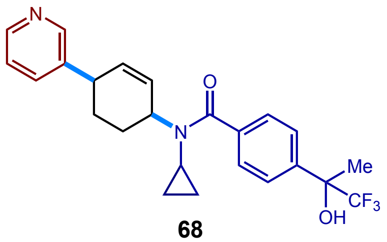

68

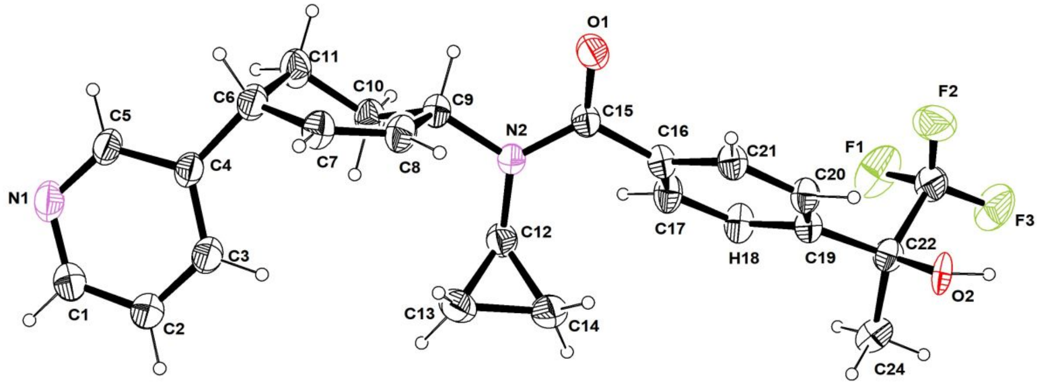

Table S43. Crystal data and structure refinement for 20230724btc01_0m_a.

20230724btc01_0m_a

Identification code	20230724btc01_0m_a
Empirical formula	$C_{24}H_{25}F_3N_2O_2$
Formula weight	430.46
Temperature/K	247.00
Crystal system	monoclinic
Space group	$P2_1/c$
a/Å	17.200(3)
b/Å	6.5046(12)
c/Å	20.052(3)
α/°	90
β/°	100.833(6)
γ/°	90
Volume/Å^3	2203.5(6)
Z	4
ρ_{calc}g/cm^3	1.298
μ/mm^{-1}	0.100
F(000)	904.0
Crystal size/mm^3	0.53 × 0.42 × 0.12
Radiation	MoKα (λ = 0.71073)
2Θ range for data collection/°	4.378 to 55.042
Index ranges	-22 ≤ h ≤ 22, -8 ≤ k ≤ 8, -25 ≤ l ≤ 23
Reflections collected	28665
Independent reflections	5036 [R_{int} = 0.1163, R_{sigma} = 0.0718]
Data/restraints/parameters	5036/699/338
Goodness-of-fit on F^2	1.059
Final R indexes [I>=2σ (I)]	R_1 = 0.0678, wR_2 = 0.2002
Final R indexes [all data]	R_1 = 0.1438, wR_2 = 0.2279
Largest diff. peak/hole / e Å^{-3}	0.32/-0.23

Table S44. Fractional Atomic Coordinates (×10^4) and Equivalent Isotropic Displacement Parameters (Å^2×10^3) for 20230724btc01_0m_a. U_{eq} is defined as 1/3 of the trace of the orthogonalised U_{IJ} tensor.

Atom	*x*	*y*	*z*	U(eq)
O1	5227.0(11)	-1485(3)	4183.1(10)	83.3(6)
O2	8685(4)	2806(13)	3584(4)	63.9(17)
N2	4554.9(11)	1475(3)	3893.3(9)	60.1(5)
N1	213.2(13)	3301(5)	3414.1(13)	87.7(8)
C19	7532.4(14)	2967(4)	4055.5(11)	61.3(6)
C21	6547.3(14)	354(4)	3735.4(12)	65.4(6)
C15	5234.5(14)	363(4)	4052.7(11)	62.0(6)
C16	6011.9(14)	1395(4)	4051.5(11)	61.3(6)
C20	7289.1(14)	1154(4)	3731.0(12)	65.7(7)

C9	3819.0(13)	431(4)	3979.3(12)	65.7(6)
C4	1484.6(14)	1768(5)	3413.5(11)	66.6(7)
C22	8378.8(15)	3697(4)	4082.0(12)	68.3(7)
C10	3289.0(15)	1763(5)	4336.3(12)	72.4(7)
C8	3355.7(15)	-370(4)	3317.6(13)	70.8(7)
C6	2055.0(15)	56(5)	3668.4(12)	75.3(7)
C12	4520.3(15)	3584(4)	3674.6(13)	71.0(7)
C18	7004.7(15)	3981(5)	4384.1(14)	76.4(7)
C7	2581.5(16)	-539(5)	3181.1(13)	74.7(7)
C17	6253.3(16)	3217(5)	4370.7(13)	75.2(7)
C3	1651.3(16)	3367(5)	3004.4(13)	80.2(8)
C5	747.4(15)	1814(5)	3598.8(13)	75.6(7)
C14	4895.8(18)	4229(5)	3099.6(15)	85.2(8)
C2	1106.4(18)	4886(5)	2805.2(15)	88.6(9)
C11	2552.2(16)	572(5)	4375.0(13)	84.0(8)
C24	8363(7)	6007(11)	3904(4)	80(3)
C23	8926(7)	2810(20)	4740(4)	85(3)
C1	397.6(17)	4798(6)	3017.2(15)	88.3(9)
C13	4011.3(18)	4118(5)	3005.4(15)	91.1(9)
F1	8633(3)	3930(10)	5270.5(19)	148(2)
F2	8847(3)	1029(9)	4832(2)	132.9(18)
F3	9632(2)	3463(8)	4782.2(19)	136.1(19)
F4	8284(3)	7095(6)	4492(2)	113.3(16)
F6	9151(2)	6576(6)	3903(2)	101.6(14)
C26	8504(8)	6047(14)	4076(6)	78(2)
F5	7908(3)	6601(7)	3436(2)	115.1(16)
C25	8621(8)	2640(20)	3486(5)	60(3)
O3	8830(8)	3310(20)	4753(5)	151(5)

Table S45. Anisotropic Displacement Parameters (Å2×103) for 20230724btc01_0m_a. The Anisotropic displacement factor exponent takes the form: -2π2[h2a*2U11+2hka*b*U12+…].

Atom	U_{11}	U_{22}	U_{33}	U_{23}	U_{13}	U_{12}
O1	61.8(12)	65.4(14)	119.2(15)	7.9(10)	7.7(9)	3.1(9)
O2	32(2)	81(3)	84(3)	-40(2)	26(2)	-7.9(19)
N2	50.2(12)	61.4(14)	69.5(11)	3.1(9)	13.6(8)	1.8(9)
N1	54.0(13)	115(2)	95.0(15)	0.7(15)	17.4(11)	1.8(14)
C19	52.2(12)	66.0(15)	65.1(11)	-7.5(11)	9.7(9)	0.8(11)
C21	58.1(14)	64.6(16)	72.9(12)	-14.3(11)	10.3(10)	2.0(12)
C15	51.9(13)	62.7(16)	70.1(12)	-3.8(11)	8.2(9)	2.1(12)
C16	50.8(12)	65.6(15)	65.9(11)	-7.1(11)	6.8(9)	4.0(11)
C20	57.3(14)	68.9(16)	72.8(13)	-12.1(11)	17.4(10)	3.0(12)
C9	51.2(13)	75.3(16)	72.3(12)	3.2(11)	16.0(10)	2.6(12)
C4	50.0(12)	88.2(18)	61.5(11)	-0.6(11)	10.2(9)	-9.3(12)
C22	60.0(14)	72.1(17)	73.4(13)	-4.4(12)	13.9(11)	-6.9(12)
C10	61.3(14)	94.3(18)	61.7(11)	-7.7(12)	11.8(10)	4.2(13)

C8	61.9(14)	73.7(16)	80.9(13)	-10.7(12)	23.5(11)	-0.9(12)
C6	56.1(14)	95.6(19)	75.2(13)	5.1(13)	14.5(11)	-7.0(13)
C12	63.1(14)	65.8(17)	84.2(14)	-2.3(12)	14.4(11)	1.9(12)
C18	62.4(15)	76.1(18)	91.8(15)	-29.2(13)	17.0(12)	-6.4(13)
C7	63.2(15)	86.2(18)	74.8(13)	-7.8(12)	13.1(11)	-5.7(13)
C17	60.4(15)	77.9(18)	90.2(15)	-25.3(13)	21.9(12)	1.0(13)
C3	58.5(14)	102(2)	80.8(14)	8.0(14)	15.7(12)	-6.4(14)
C5	52.9(14)	101(2)	75.7(13)	5.7(13)	18.8(11)	-8.5(14)
C14	84.2(19)	75.4(18)	95.5(17)	13.5(14)	15.7(14)	-3.9(15)
C2	68.9(17)	108(2)	88.8(16)	18.7(16)	14.0(13)	-2.9(16)
C11	64.4(15)	122(2)	67.5(13)	2.0(14)	17.8(11)	8.8(15)
C24	88(6)	90(5)	65(4)	-12(3)	20(4)	-28(4)
C23	57(4)	114(5)	76(4)	10(4)	-9(3)	-14(4)
C1	65.2(17)	104(2)	93.5(17)	7.7(17)	9.3(14)	5.1(16)
C13	86.9(19)	76.1(19)	104.0(19)	21.5(15)	2.2(15)	6.7(15)
F1	132(4)	239(6)	76(2)	-42(3)	24(2)	-61(3)
F2	96(3)	139(4)	149(3)	58(3)	-13(2)	-11(3)
F3	67(2)	220(5)	112(3)	12(3)	-5.3(18)	-50(3)
F4	143(4)	86(3)	129(3)	-43(2)	73(3)	-27(2)
F6	104(3)	77(3)	140(3)	-11(2)	63(2)	-20(2)
C26	72(4)	76(4)	89(4)	-13(3)	24(4)	-5(3)
F5	138(4)	97(3)	118(3)	28(2)	45(3)	26(2)
C25	58(3)	61(3)	61(3)	-3.4(18)	11.4(17)	0.3(19)
O3	117(7)	174(9)	159(8)	24(6)	15(6)	-36(6)

Table S46. Bond Lengths for 20230724btc01_0m_a.

Atom	Atom	Length/Å	Atom	Atom	Length/Å
O1	C15	1.231(3)	C22	C23	1.580(8)
O2	C22	1.345(6)	C22	C26	1.544(10)
N2	C15	1.360(3)	C22	C25	1.502(8)
N2	C9	1.475(3)	C22	O3	1.444(8)
N2	C12	1.438(3)	C10	C11	1.500(4)
N1	C5	1.337(4)	C8	C7	1.313(3)
N1	C1	1.333(4)	C6	C7	1.502(4)
C19	C20	1.374(3)	C6	C11	1.548(4)
C19	C22	1.523(3)	C12	C14	1.484(4)
C19	C18	1.385(3)	C12	C13	1.499(4)
C21	C16	1.388(3)	C18	C17	1.380(4)
C21	C20	1.379(3)	C3	C2	1.369(4)
C15	C16	1.496(3)	C14	C13	1.499(4)
C16	C17	1.374(4)	C2	C1	1.366(4)
C9	C10	1.529(4)	C23	F1	1.455(14)
C9	C8	1.507(3)	C23	F2	1.184(14)
C4	C6	1.508(4)	C23	F3	1.275(14)
C4	C3	1.388(4)	F4	C26	1.193(11)

C4	C5	1.387(4)	F6	C26	1.274(13)
C22	C24	1.543(7)	C26	F5	1.527(11)

Table S47. Bond Angles for 20230724btc01_0m_a.

Atom	Atom	Atom	Angle/°	Atom	Atom	Atom	Angle/°
C15	N2	C9	116.5(2)	O3	C22	C25	118.8(9)
C15	N2	C12	124.3(2)	C11	C10	C9	108.4(2)
C12	N2	C9	119.17(19)	C7	C8	C9	124.1(2)
C1	N1	C5	117.7(3)	C4	C6	C11	111.2(2)
C20	C19	C22	119.3(2)	C7	C6	C4	113.8(2)
C20	C19	C18	117.8(2)	C7	C6	C11	110.8(2)
C18	C19	C22	122.8(2)	N2	C12	C14	120.5(2)
C20	C21	C16	120.8(2)	N2	C12	C13	118.6(2)
O1	C15	N2	121.7(2)	C14	C12	C13	60.33(19)
O1	C15	C16	118.9(2)	C17	C18	C19	121.0(2)
N2	C15	C16	119.3(2)	C8	C7	C6	123.7(2)
C21	C16	C15	116.8(2)	C16	C17	C18	121.3(2)
C17	C16	C21	117.7(2)	C2	C3	C4	120.5(3)
C17	C16	C15	125.3(2)	N1	C5	C4	123.8(3)
C19	C20	C21	121.4(2)	C12	C14	C13	60.32(19)
N2	C9	C10	113.2(2)	C1	C2	C3	118.8(3)
N2	C9	C8	112.33(19)	C10	C11	C6	113.0(2)
C8	C9	C10	110.2(2)	F1	C23	C22	101.1(8)
C3	C4	C6	124.1(2)	F2	C23	C22	114.8(7)
C5	C4	C6	119.6(2)	F2	C23	F1	108.1(9)
C5	C4	C3	116.3(3)	F2	C23	F3	116.9(10)
O2	C22	C19	110.2(3)	F3	C23	C22	110.1(9)
O2	C22	C24	103.9(6)	F3	C23	F1	103.9(8)
O2	C22	C23	102.0(6)	N1	C1	C2	122.9(3)
C19	C22	C24	108.7(5)	C12	C13	C14	59.36(18)
C19	C22	C23	109.3(5)	F4	C26	C22	119.8(9)
C19	C22	C26	116.2(5)	F4	C26	F6	116.4(8)
C24	C22	C23	122.1(6)	F4	C26	F5	101.7(8)
C25	C22	C19	103.7(6)	F6	C26	C22	113.5(8)
C25	C22	C26	112.7(7)	F6	C26	F5	101.0(8)
O3	C22	C19	108.8(6)	F5	C26	C22	99.7(6)
O3	C22	C26	97.3(8)				

Table S48. Torsion Angles for 20230724btc01_0m_a.

A	B	C	D	Angle/°	A	B	C	D	Angle/°
O1	C15	C16	C21	43.9(3)	C4	C6	C7	C8	117.8(3)
O1	C15	C16	C17	-132.1(3)	C4	C6	C11	C10	-87.4(3)
O2	C22	C23	F1	175.1(7)	C4	C3	C2	C1	0.1(4)
O2	C22	C23	F2	-68.8(11)	C22	C19	C20	C21	175.3(2)

O2	C22	C23	F3	65.6(10)	C22	C19	C18	C17	-177.2(2)
N2	C15	C16	C21	-134.5(2)	C10	C9	C8	C7	-22.3(4)
N2	C15	C16	C17	49.6(3)	C8	C9	C10	C11	51.5(3)
N2	C9	C10	C11	178.25(19)	C6	C4	C3	C2	-178.7(3)
N2	C9	C8	C7	-149.6(3)	C6	C4	C5	N1	178.0(3)
N2	C12	C14	C13	107.6(3)	C12	N2	C15	O1	-173.9(2)
N2	C12	C13	C14	-110.8(3)	C12	N2	C15	C16	4.4(3)
C19	C22	C23	F1	-68.3(8)	C12	N2	C9	C10	-43.3(3)
C19	C22	C23	F2	47.8(11)	C12	N2	C9	C8	82.3(3)
C19	C22	C23	F3	-177.8(7)	C18	C19	C20	C21	-0.5(4)
C19	C22	C26	F4	55.3(11)	C18	C19	C22	O2	-163.2(5)
C19	C22	C26	F6	-160.8(6)	C18	C19	C22	C24	-49.9(5)
C19	C22	C26	F5	-54.3(8)	C18	C19	C22	C23	85.5(6)
C19	C18	C17	C16	2.2(4)	C18	C19	C22	C26	-36.9(6)
C21	C16	C17	C18	-0.7(4)	C18	C19	C22	C25	-161.0(6)
C15	N2	C9	C10	134.5(2)	C18	C19	C22	O3	71.7(8)
C15	N2	C9	C8	-99.9(2)	C7	C6	C11	C10	40.1(4)
C15	N2	C12	C14	52.5(3)	C3	C4	C6	C7	-33.3(4)
C15	N2	C12	C13	123.1(3)	C3	C4	C6	C11	92.6(3)
C15	C16	C17	C18	175.2(3)	C3	C4	C5	N1	-1.0(4)
C16	C21	C20	C19	2.0(4)	C3	C2	C1	N1	0.4(5)
C20	C19	C22	O2	21.2(5)	C5	N1	C1	C2	-1.1(4)
C20	C19	C22	C24	134.5(4)	C5	C4	C6	C7	147.9(2)
C20	C19	C22	C23	-90.0(6)	C5	C4	C6	C11	-86.2(3)
C20	C19	C22	C26	147.6(5)	C5	C4	C3	C2	0.2(4)
C20	C19	C22	C25	23.4(6)	C11	C6	C7	C8	-8.4(4)
C20	C19	C22	O3	-103.9(8)	C24	C22	C23	F1	60.1(8)
C20	C19	C18	C17	-1.6(4)	C24	C22	C23	F2	176.2(10)
C20	C21	C16	C15	-177.6(2)	C24	C22	C23	F3	-49.4(12)
C20	C21	C16	C17	-1.4(4)	C1	N1	C5	C4	1.4(4)
C9	N2	C15	O1	8.4(3)	C25	C22	C26	F4	174.7(10)
C9	N2	C15	C16	-173.28(19)	C25	C22	C26	F6	-41.4(11)
C9	N2	C12	C14	-129.8(2)	C25	C22	C26	F5	65.1(9)
C9	N2	C12	C13	-59.3(3)	O3	C22	C26	F4	-59.9(10)
C9	C10	C11	C6	-62.8(3)	O3	C22	C26	F6	84.0(11)
C9	C8	C7	C6	0.2(5)	O3	C22	C26	F5	-169.5(10)

Table S49. Hydrogen Atom Coordinates (Å×10^4) and Isotropic Displacement Parameters (Å^2×10^3) for 20230724btc01_0m_a.

Atom	*x*	*y*	*z*	U(eq)
H2	9175.46	2871.66	3677.45	96
H21	6402.45	-911.73	3521.6	79
H20	7635.26	443.2	3501.27	79
H9	3976.47	-777.19	4273.81	79
H10A	3567.66	2117.38	4794.16	87

H10B	3152.32	3039.99	4082	87
H8	3638.63	-774.83	2981.48	85
H6	1732.4	-1167.59	3727.43	90
H12	4531.4	4617.91	4038.55	85
H18	7160.69	5206.56	4619.81	92
H7	2343.04	-1061.04	2754.48	90
H17	5899.78	3959.05	4584.18	90
H3	2142.05	3408.05	2863.03	96
H5	615.57	738.17	3870.04	91
H14A	5165.94	3174.28	2878.72	102
H14B	5139.9	5595.11	3122.33	102
H2A	1218.01	5969.86	2527.69	106
H11A	2226.4	1373.05	4632.36	101
H11B	2699.78	-711.07	4622.69	101
H24A	8147.95	6777.03	4241.85	121
H24B	8897.63	6475.49	3898.45	121
H24C	8035.26	6221.28	3460.48	121
H1	23.36	5839.01	2877.17	106
H13A	3716.53	5414.83	2972.92	109
H13B	3742.58	2992.44	2729.15	109
H25A	8266.2	3046.52	3072.2	90
H25B	9158.26	3038.94	3459.28	90
H25C	8596.03	1165.92	3543.28	90
H3A	8539.62	2782.51	4992.25	227

Table S50. Atomic Occupancy for 20230724btc01_0m_a.

Atom	*Occupancy*	Atom	*Occupancy*	Atom	*Occupancy*
O2	0.516(3)	H2	0.516(3)	C24	0.516(3)
H24A	0.516(3)	H24B	0.516(3)	H24C	0.516(3)
C23	0.516(3)	F1	0.516(3)	F2	0.516(3)
F3	0.516(3)	F4	0.484(3)	F6	0.484(3)
C26	0.484(3)	F5	0.484(3)	C25	0.484(3)
H25A	0.484(3)	H25B	0.484(3)	H25C	0.484(3)
O3	0.484(3)	H3A	0.484(3)		

20230724btc01_0m_a

Table S51. Crystal data and structure refinement for 20230724btc01_0m_a.

Identification code	20230724btc01_0m_a
Empirical formula	$C_{24}H_{25}F_3N_2O_2$
Formula weight	430.46
Temperature/K	247.00
Crystal system	monoclinic
Space group	$P2_1/c$
a/Å	17.200(3)

b/Å	6.5046(12)
c/Å	20.052(3)
α/°	90
β/°	100.833(6)
γ/°	90
Volume/Å^3	2203.5(6)
Z	4
ρ_{calc}g/cm^3	1.298
μ/mm^{-1}	0.100
F(000)	904.0
Crystal size/mm^3	0.53 × 0.42 × 0.12
Radiation	MoKα (λ = 0.71073)
2Θ range for data collection/°	4.378 to 55.042
Index ranges	$-22 \leq h \leq 22$, $-8 \leq k \leq 8$, $-25 \leq l \leq 23$
Reflections collected	28665
Independent reflections	5036 [R_{int} = 0.1163, R_{sigma} = 0.0718]
Data/restraints/parameters	5036/699/338
Goodness-of-fit on F^2	1.059
Final R indexes [I>=2σ (I)]	R_1 = 0.0678, wR_2 = 0.2002
Final R indexes [all data]	R_1 = 0.1438, wR_2 = 0.2279
Largest diff. peak/hole / e Å^{-3}	0.32/-0.23

10.4 The Single Crystal Date of 1-N-iminosugar 70

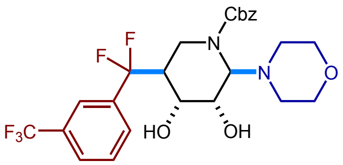

70

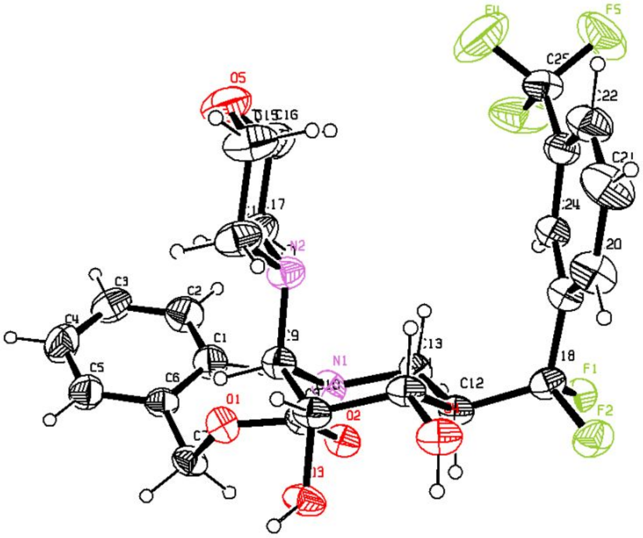

Table S52. Crystal data and structure refinement for 20230906_2_0m.

Identification code	20230906_2_0m
Empirical formula	$C_{25}H_{27}F_5N_2O_5$
Formula weight	530.48
Temperature/K	151.00
Crystal system	triclinic
Space group	P-1
a/Å	6.7280(3)
b/Å	13.2990(6)
c/Å	14.4233(7)
α/°	96.203(3)
β/°	94.290(3)
γ/°	103.239(2)
Volume/Å^3	1242.26(10)
Z	2
ρ_{calc}g/cm^3	1.418
μ/mm^{-1}	1.066
F(000)	552.0
Crystal size/mm^3	0.41 × 0.32 × 0.12
Radiation	CuKα (λ = 1.54178)
2Θ range for data collection/°	6.196 to 137.012
Index ranges	$-8 \leq h \leq 7$, $-16 \leq k \leq 16$, $-17 \leq l \leq 17$
Reflections collected	31636
Independent reflections	4539 [R_{int} = 0.0786, R_{sigma} = 0.0441]
Data/restraints/parameters	4539/39/365
Goodness-of-fit on F^2	1.080
Final R indexes [I>=2σ (I)]	R_1 = 0.0637, wR_2 = 0.1732
Final R indexes [all data]	R_1 = 0.0855, wR_2 = 0.1939
Largest diff. peak/hole / e Å^{-3}	0.23/-0.22

Table S53. Fractional Atomic Coordinates (×104) and Equivalent Isotropic Displacement Parameters (Å2×103) for 20230906_2_0m. Ueq is defined as 1/3 of the trace of the orthogonalised UIJ tensor.

**Atom**	***x***	***y***	***z***	**U(eq)**
F1	4336(2)	7446.7(12)	5412.6(11)	61.1(4)
F2	1577(3)	7931.9(15)	5745.4(12)	74.1(5)
O2	3372(3)	4115.9(15)	3890.2(14)	60.1(5)
O1	814(3)	3431.2(15)	2730.6(15)	63.1(5)
O4	-2044(3)	7087.8(17)	4484.4(15)	66.4(6)
O3	-2376(3)	4902.3(17)	3982.0(15)	67.8(6)
N1	1373(3)	5112.8(17)	3301.0(16)	53.2(5)
N2	373(3)	5956.4(19)	2041.7(16)	61.7(6)
C8	1943(4)	4214(2)	3351.0(19)	51.8(6)

C12	1259(3)	6582(2)	4493.1(18)	49.3(6)
C10	-1784(4)	5646(2)	3357.1(19)	54.9(6)
C18	2555(4)	7621(2)	5001.4(18)	53.8(6)
C19	3088(4)	8502(2)	4421(2)	56.0(6)
C9	-378(4)	5236(2)	2703.1(19)	55.1(6)
C11	-685(4)	6693(2)	3917.4(19)	53.1(6)
C24	4664(4)	8536(2)	3847.2(19)	57.1(6)
O5	1013(5)	6585(3)	233.1(19)	111.0(10)
C13	2577(4)	6062(2)	3872.6(19)	52.9(6)
C6	2525(4)	2171(2)	1982(2)	59.7(7)
C23	5037(5)	9293(2)	3252(2)	65.3(7)
F3	7562(14)	8524(5)	2623(6)	134(3)
C7	1251(5)	2409(2)	2749(2)	64.0(7)
C17	1779(5)	5572(3)	1442(2)	77.4(9)
C1	4608(5)	2620(3)	2036(2)	77.4(9)
C5	1657(6)	1453(3)	1220(2)	79.8(9)
C20	2007(5)	9269(3)	4431(3)	81.2(10)
C14	-1269(6)	6172(3)	1431(3)	82.6(10)
F5	8026(12)	10138(4)	2662(6)	147(3)
C25	6618(6)	9282(3)	2586(3)	85.6(10)
C22	3958(6)	10056(3)	3268(3)	87.9(11)
C3	4878(7)	1645(3)	590(3)	92.9(11)
C2	5759(6)	2365(3)	1344(3)	89.3(11)
C21	2467(6)	10051(3)	3870(4)	101.0(13)
C4	2821(7)	1189(4)	529(3)	99.3(12)
C16	2609(7)	6368(4)	824(3)	101.1(13)
F4	5749(11)	9154(10)	1704(3)	174(4)
C15	-393(8)	6945(4)	792(3)	108.3(14)
F8	8450(16)	9520(20)	3053(10)	147(8)
F6	6360(30)	8423(11)	2055(16)	128(7)
F7	6700(30)	9970(17)	2014(15)	132(7)

Table S54. Anisotropic Displacement Parameters (Å2×103) for 20230906_2_0m. The Anisotropic displacement factor exponent takes the form: 2π2[h2a*2U11+2hka*b*U12+…].

Atom	U_{11}	U_{22}	U_{33}	U_{23}	U_{13}	U_{12}
F1	50.4(8)	72.8(10)	62.3(9)	13.9(7)	3.2(7)	17.8(7)
F2	71.0(11)	90.6(12)	66.4(10)	4.3(9)	28.6(8)	26.4(9)
O2	42.4(9)	67.7(12)	75.7(12)	17.9(9)	6.5(9)	20.4(8)
O1	54.4(11)	57.4(11)	78.4(13)	5.0(9)	5.0(9)	17.6(8)
O4	46.7(10)	85.8(14)	80.5(13)	23.2(11)	23.8(9)	32.7(10)
O3	39.5(9)	81.2(14)	86.7(14)	38.7(11)	9.0(9)	8.9(9)
N1	37.1(10)	57.5(13)	68.2(13)	11.3(10)	4.9(9)	16.3(9)

N2	54.3(13)	76.2(16)	60.2(13)	18.4(11)	12.9(10)	20.3(11)
C8	35.6(12)	57.6(15)	65.5(16)	13.2(12)	15.4(11)	12.3(10)
C12	37.9(12)	58.3(15)	57.2(14)	15.2(11)	11.9(10)	17.0(10)
C10	35.3(12)	69.6(17)	65.6(16)	25.3(13)	8.1(11)	16.0(11)
C18	44.0(13)	70.1(17)	55.6(14)	11.3(12)	17.2(11)	24.9(12)
C19	46.6(13)	57.7(15)	67.6(16)	12.2(12)	8.3(12)	17.8(11)
C9	41.8(13)	63.7(16)	62.4(16)	14.1(12)	3.3(11)	15.7(11)
C11	38.3(12)	66.0(16)	64.2(15)	21.7(12)	16.4(11)	21.7(11)
C24	48.3(14)	57.4(15)	66.9(16)	12.8(12)	7.3(12)	13.1(11)
O5	119(2)	145(3)	76.0(16)	40.7(17)	26.4(16)	28.9(19)
C13	35.7(12)	56.4(15)	68.0(16)	6.6(12)	9.5(11)	13.5(10)
C6	63.7(16)	59.1(16)	62.6(16)	15.4(13)	15.5(13)	21.2(13)
C23	64.1(17)	63.0(17)	66.8(17)	18.4(14)	5.1(14)	7.0(13)
F3	157(5)	123(4)	161(6)	63(4)	110(5)	62(4)
C7	67.8(17)	50.8(15)	77.7(19)	11.7(13)	23.7(14)	16.3(13)
C17	70.6(19)	104(3)	63.3(18)	12.2(17)	19.4(15)	27.5(18)
C1	63.8(18)	86(2)	80(2)	2.5(17)	20.0(16)	13.5(16)
C5	73(2)	91(2)	73(2)	5.4(18)	12.8(16)	14.8(17)
C20	68.8(19)	73(2)	115(3)	22.2(19)	23.2(19)	33.7(16)
C14	78(2)	109(3)	75(2)	35.7(19)	15.0(17)	40(2)
F5	131(5)	99(3)	190(7)	8(3)	79(5)	-32(3)
C25	91(3)	85(3)	76(2)	28(2)	17.6(19)	1(2)
C22	86(2)	76(2)	107(3)	39(2)	5(2)	17.7(18)
C3	99(3)	117(3)	74(2)	13(2)	35(2)	40(2)
C2	67(2)	111(3)	94(3)	12(2)	28.4(19)	22.5(19)
C21	88(3)	72(2)	159(4)	41(2)	18(3)	37.5(19)
C4	103(3)	116(3)	71(2)	-11(2)	16(2)	16(2)
C16	91(3)	137(4)	78(2)	28(2)	30(2)	21(2)
F4	190(6)	254(10)	66(2)	22(4)	19(3)	29(6)
C15	123(3)	132(4)	91(3)	56(3)	29(2)	49(3)
F8	64(6)	230(20)	133(9)	30(11)	11(6)	12(9)
F6	135(12)	97(9)	137(12)	-17(8)	59(10)	-3(7)
F7	136(11)	141(12)	131(11)	83(10)	41(9)	17(9)

Table S55. Bond Lengths for 20230906_2_0m.

Atom	Atom	Length/Å	Atom	Atom	Length/Å
F1	C18	1.376(3)	O5	C16	1.422(5)
F2	C18	1.374(3)	O5	C15	1.419(5)
O2	C8	1.230(3)	C6	C7	1.500(4)
O1	C8	1.340(3)	C6	C1	1.384(4)
O1	C7	1.457(3)	C6	C5	1.372(5)
O4	C11	1.421(3)	C23	C25	1.487(5)
O3	C10	1.419(3)	C23	C22	1.376(5)
N1	C8	1.344(3)	F3	C25	1.312(6)
N1	C9	1.458(3)	C17	C16	1.495(5)

N1	C13	1.462(3)	C1	C2	1.369(5)
N2	C9	1.460(4)	C5	C4	1.377(5)
N2	C17	1.467(4)	C20	C21	1.380(5)
N2	C14	1.461(4)	C14	C15	1.504(5)
C12	C18	1.530(4)	F5	C25	1.292(5)
C12	C11	1.540(3)	C25	F4	1.335(6)
C12	C13	1.528(3)	C25	F8	1.313(11)
C10	C9	1.530(4)	C25	F6	1.273(11)
C10	C11	1.528(4)	C25	F7	1.291(10)
C18	C19	1.505(4)	C22	C21	1.374(6)
C19	C24	1.389(4)	C3	C2	1.367(6)
C19	C20	1.382(4)	C3	C4	1.370(6)
C24	C23	1.385(4)			

Table S56. Bond Angles for 20230906_2_0m.

Atom	Atom	Atom	Angle/˚	Atom	Atom	Atom	Angle/˚
C8	O1	C7	117.2(2)	N1	C13	C12	112.03(19)
C8	N1	C9	125.2(2)	C1	C6	C7	121.8(3)
C8	N1	C13	119.2(2)	C5	C6	C7	120.1(3)
C9	N1	C13	115.6(2)	C5	C6	C1	118.0(3)
C9	N2	C17	112.0(2)	C24	C23	C25	119.8(3)
C9	N2	C14	113.2(2)	C22	C23	C24	120.7(3)
C14	N2	C17	107.7(2)	C22	C23	C25	119.6(3)
O2	C8	O1	123.1(2)	O1	C7	C6	111.5(2)
O2	C8	N1	124.2(2)	N2	C17	C16	109.8(3)
O1	C8	N1	112.7(2)	C2	C1	C6	120.9(3)
C18	C12	C11	113.1(2)	C6	C5	C4	121.0(3)
C13	C12	C18	109.56(19)	C21	C20	C19	120.5(3)
C13	C12	C11	110.8(2)	N2	C14	C15	110.4(3)
O3	C10	C9	107.8(2)	F3	C25	C23	115.0(3)
O3	C10	C11	109.5(2)	F3	C25	F4	105.0(6)
C11	C10	C9	111.2(2)	F5	C25	C23	114.2(4)
F1	C18	C12	108.1(2)	F5	C25	F3	106.8(5)
F1	C18	C19	109.1(2)	F5	C25	F4	104.9(5)
F2	C18	F1	104.2(2)	F4	C25	C23	110.0(4)
F2	C18	C12	108.9(2)	F8	C25	C23	109.6(7)
F2	C18	C19	109.1(2)	F6	C25	C23	114.1(6)
C19	C18	C12	116.8(2)	F6	C25	F8	109.8(12)
C24	C19	C18	119.7(2)	F6	C25	F7	104.3(12)
C20	C19	C18	121.2(3)	F7	C25	C23	113.8(8)
C20	C19	C24	119.1(3)	F7	C25	F8	104.7(11)
N1	C9	N2	108.6(2)	C21	C22	C23	119.4(3)
N1	C9	C10	106.4(2)	C2	C3	C4	119.2(3)
N2	C9	C10	112.6(2)	C3	C2	C1	120.5(4)
O4	C11	C12	112.6(2)	C22	C21	C20	120.4(3)

O4	C11	C10	110.5(2)	C3	C4	C5	120.3(4)
C10	C11	C12	110.0(2)	O5	C16	C17	111.5(3)
C23	C24	C19	119.7(3)	O5	C15	C14	111.8(4)
C15	O5	C16	109.4(3)				

Table S57. Torsion Angles for 20230906_2_0m.

A	B	C	D	Angle/°	A	B	C	D	Angle/°
F1	C18	C19	C24	44.1(3)	C24	C23	C25	F6	54.7(17)
F1	C18	C19	C20	-137.4(3)	C24	C23	C25	F7	174.3(14)
F2	C18	C19	C24	157.3(2)	C24	C23	C22	C21	-1.3(6)
F2	C18	C19	C20	-24.2(4)	C13	N1	C8	O2	4.1(4)
O3	C10	C9	N1	-59.6(3)	C13	N1	C8	O1	-174.4(2)
O3	C10	C9	N2	-178.5(2)	C13	N1	C9	N2	61.4(3)
O3	C10	C11	O4	-64.0(3)	C13	N1	C9	C10	-60.0(3)
O3	C10	C11	C12	60.8(2)	C13	C12	C18	F1	-50.0(3)
N2	C17	C16	O5	-60.4(4)	C13	C12	C18	F2	-162.5(2)
N2	C14	C15	O5	58.3(5)	C13	C12	C18	C19	73.4(3)
C8	O1	C7	C6	-101.1(3)	C13	C12	C11	O4	174.7(2)
C8	N1	C9	N2	-117.9(3)	C13	C12	C11	C10	51.1(3)
C8	N1	C9	C10	120.7(3)	C6	C1	C2	C3	-1.3(6)
C8	N1	C13	C12	-124.5(2)	C6	C5	C4	C3	-0.3(7)
C12	C18	C19	C24	-78.7(3)	C23	C22	C21	C20	-2.1(7)
C12	C18	C19	C20	99.8(3)	C7	O1	C8	O2	3.8(3)
C18	C12	C11	O4	-61.9(3)	C7	O1	C8	N1	-177.7(2)
C18	C12	C11	C10	174.53(19)	C7	C6	C1	C2	177.8(3)
C18	C12	C13	N1	-174.6(2)	C7	C6	C5	C4	-177.0(3)
C18	C19	C24	C23	174.3(3)	C17	N2	C9	N1	60.4(3)
C18	C19	C20	C21	-177.7(3)	C17	N2	C9	C10	177.9(2)
C19	C24	C23	C25	-175.4(3)	C17	N2	C14	C15	-57.5(4)
C19	C24	C23	C22	4.5(5)	C1	C6	C7	O1	76.0(4)
C19	C20	C21	C22	2.4(7)	C1	C6	C5	C4	-0.2(5)
C9	N1	C8	O2	-176.7(2)	C5	C6	C7	O1	-107.3(3)
C9	N1	C8	O1	4.8(3)	C5	C6	C1	C2	1.0(5)
C9	N1	C13	C12	56.1(3)	C20	C19	C24	C23	-4.2(4)
C9	N2	C17	C16	-176.2(3)	C14	N2	C9	N1	-177.6(3)
C9	N2	C14	C15	178.1(3)	C14	N2	C9	C10	-60.0(3)
C9	C10	C11	O4	177.0(2)	C14	N2	C17	C16	58.6(4)
C9	C10	C11	C12	-58.2(3)	C25	C23	C22	C21	178.6(4)
C11	C12	C18	F1	-174.11(19)	C22	C23	C25	F3	-180.0(7)
C11	C12	C18	F2	73.3(3)	C22	C23	C25	F5	56.0(8)
C11	C12	C18	C19	-50.7(3)	C22	C23	C25	F4	-61.7(7)
C11	C12	C13	N1	-49.1(3)	C22	C23	C25	F8	111.2(16)
C11	C10	C9	N1	60.4(3)	C22	C23	C25	F6	-125.2(16)
C11	C10	C9	N2	-58.5(3)	C22	C23	C25	F7	-5.6(15)
C24	C19	C20	C21	0.8(5)	C2	C3	C4	C5	0.0(7)

C24	C23	C25	F3	-0.1(8)	C4	C3	C2	C1	0.8(7)
C24	C23	C25	F5	-124.1(7)	C16	O5	C15	C14	-57.2(5)
C24	C23	C25	F4	118.2(7)	C15	O5	C16	C17	58.5(5)
C24	C23	C25	F8	-68.9(16)					

Table S58. Hydrogen Atom Coordinates (Å×104) and Isotropic Displacement Parameters (Å2×103) for 20230906_2_0m.

Atom	*x*	*y*	*z*	U(eq)
H4	-2410.38	6683.46	4881.9	100
H3	-3661.83	4698.49	3929.15	102
H12	810.79	6119.75	4978.85	59
H10	-3032.78	5727.01	2979.07	66
H9	-1121.66	4546.1	2352.79	66
H11	-246.15	7195.59	3461.52	64
H24	5482.51	8042.01	3863.04	68
H13A	3232.97	6556.88	3456.38	63
H13B	3680.4	5888.96	4273.08	63
H7A	-57.45	1870.53	2676.91	77
H7B	1990.12	2386.57	3363.24	77
H17A	1041.36	4914.32	1052.27	93
H17B	2924.11	5426.91	1836.57	93
H1	5248.45	3110.92	2562.09	93
H5	231.1	1133.75	1167.85	96
H20	937.43	9258.13	4827.29	97
H14A	-2222.25	6453.47	1816.96	99
H14B	-2054.02	5516.84	1051.41	99
H22	4240.67	10581.87	2867.09	106
H3A	5682.79	1463.14	114.09	111
H2	7180.23	2692.21	1386.86	107
H21	1748.93	10589.78	3900.36	121
H4A	2194.64	687.53	7.88	119
H16A	3370.46	7018.55	1217.75	121
H16B	3582.64	6111.28	433.07	121
H15A	-1525.68	7074.53	378.73	130
H15B	314.38	7612.85	1173.51	130

Table S59. Atomic Occupancy for 20230906_2_0m.

Atom	*Occupancy*	Atom	*Occupancy*	Atom	*Occupancy*
F3	0.736(14)	F5	0.736(14)	F4	0.736(14)
F8	0.264(14)	F6	0.264(14)	F7	0.264(14)

Table S60. Crystal data and structure refinement for 20230906_2_0m.

Identification code	20230906_2_0m
Empirical formula	$C_{25}H_{27}F_5N_2O_5$
Formula weight	530.48
Temperature/K	151.00
Crystal system	triclinic
Space group	P-1
a/Å	6.7280(3)
b/Å	13.2990(6)
c/Å	14.4233(7)
α/°	96.203(3)
β/°	94.290(3)
γ/°	103.239(2)
Volume/Å^3	1242.26(10)
Z	2
ρ_{calc}g/cm^3	1.418
μ/mm^{-1}	1.066
F(000)	552.0
Crystal size/mm^3	0.41 × 0.32 × 0.12
Radiation	CuKα (λ = 1.54178)
2Θ range for data collection/°	6.196 to 137.012
Index ranges	$-8 \leq h \leq 7$, $-16 \leq k \leq 16$, $-17 \leq l \leq 17$
Reflections collected	31636
Independent reflections	4539 [R_{int} = 0.0786, R_{sigma} = 0.0441]
Data/restraints/parameters	4539/39/365
Goodness-of-fit on F^2	1.080
Final R indexes [I>=2σ (I)]	R_1 = 0.0637, wR_2 = 0.1732
Final R indexes [all data]	R_1 = 0.0855, wR_2 = 0.1939
Largest diff. peak/hole / e Å^{-3}	0.23/-0.22

XI. Spectral Data

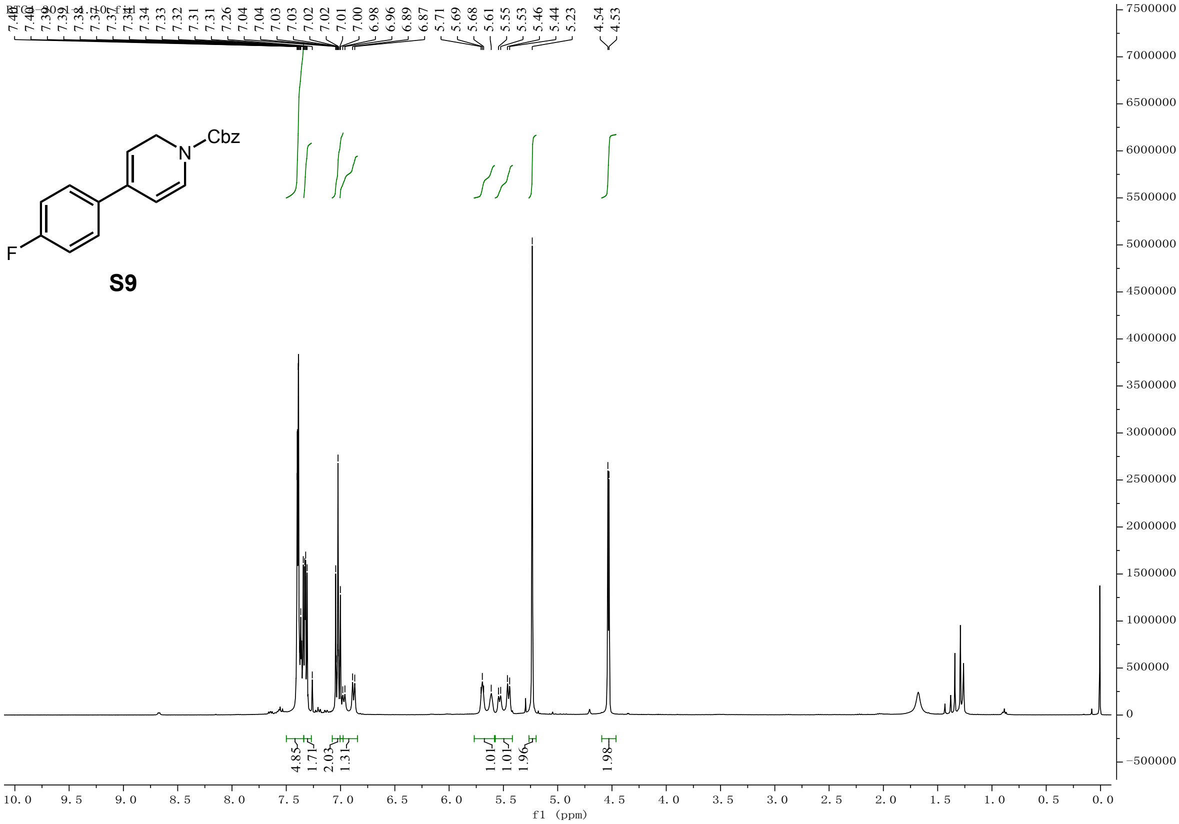

5.71
5.69
5.68
5.61
5.55
5.53
5.46
5.44
5.23
4.54
4.53
Cbz
N
F
S9
4.85
1.71
2.03
1.31
1.01
1.01
1.96
1.98
f1 (ppm)

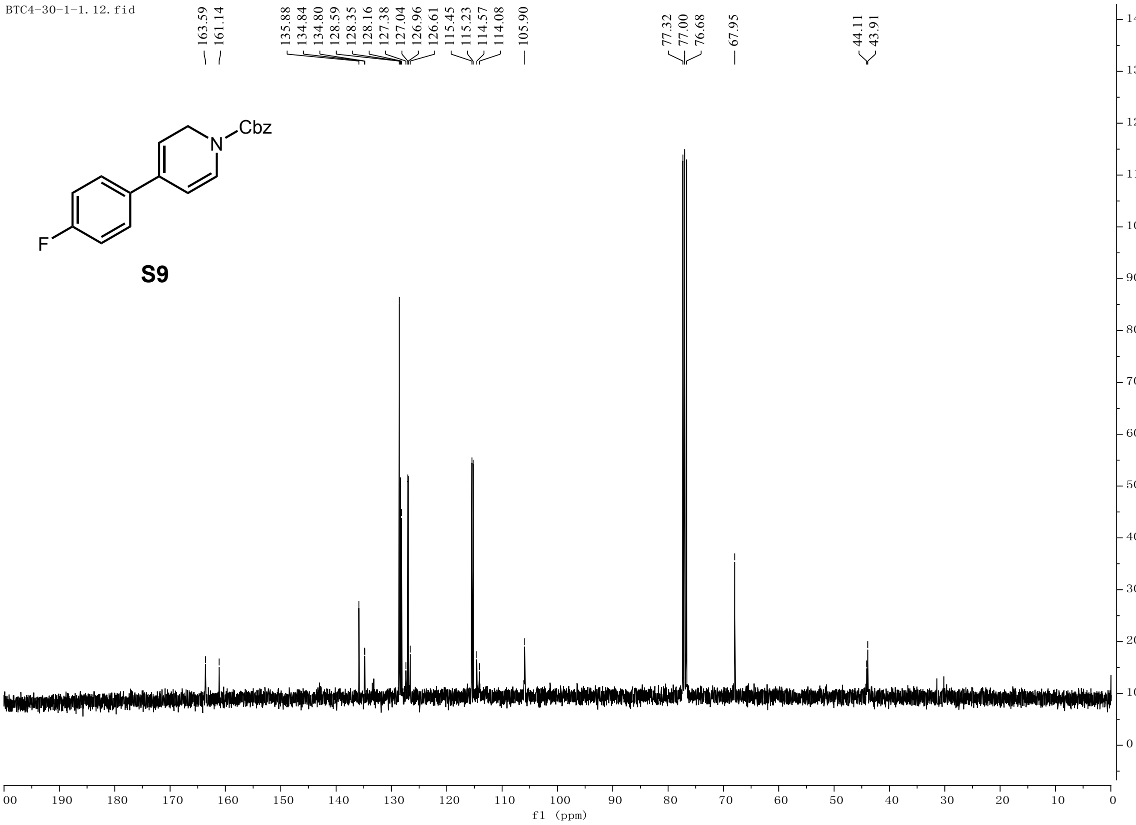

BTC4-30-1-1.12.fid
163.59
161.14
135.88
134.84
134.80
128.59
128.35
128.16
127.38
127.04
126.96
126.61
115.45
115.23
114.57
114.08
105.90
77.32
77.00
76.68
67.95
44.11
43.91
Cbz
N
F
S9
f1 (ppm)

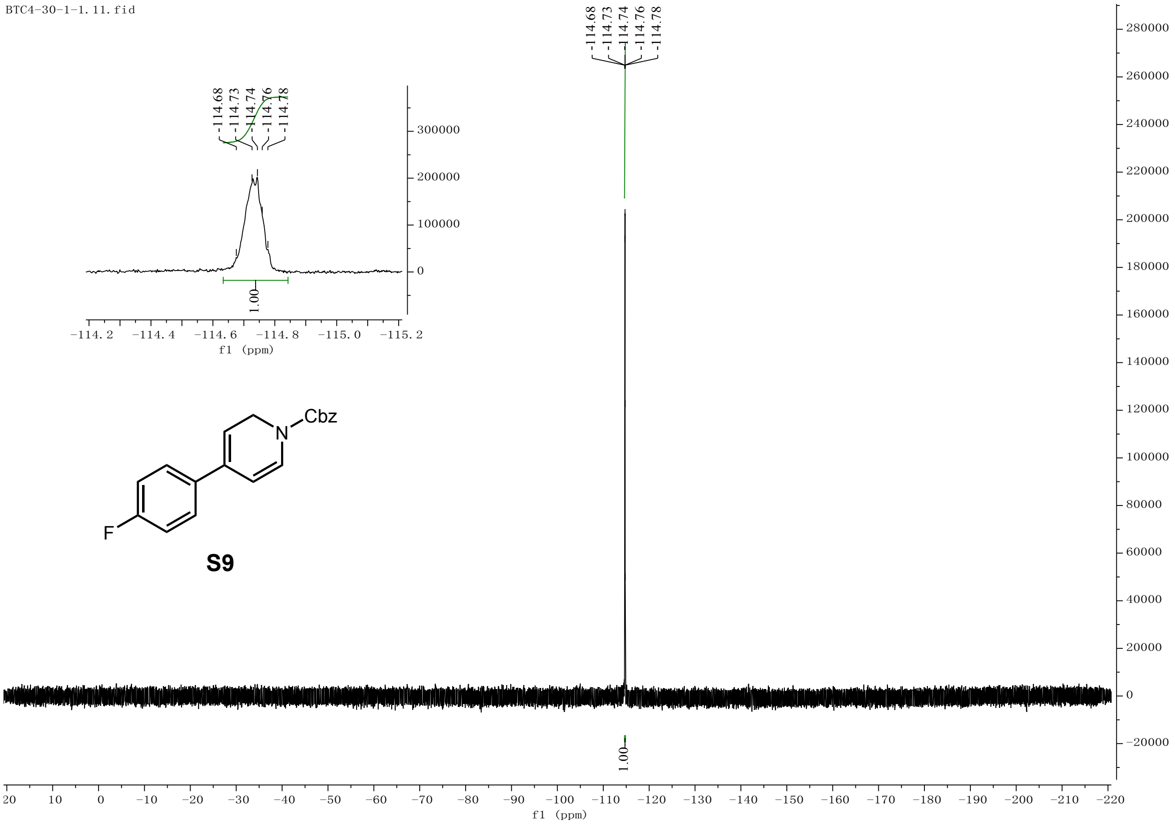
BTC4-30-1-1.11.fid
-114.68
-114.73
-114.74
-114.76
-114.78
1.00
f1 (ppm)
N
Cbz
F

S9

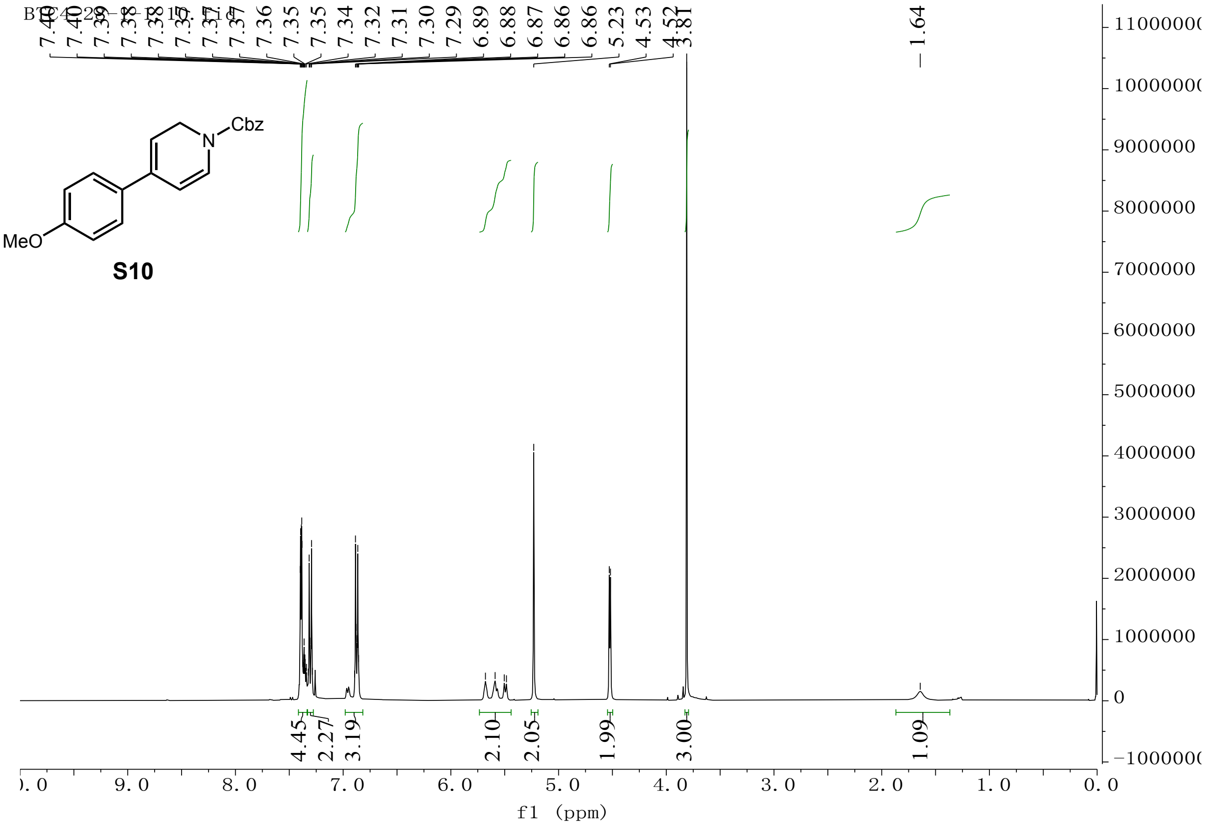
7.40
7.39
7.38
7.38
7.37
7.37
7.37
7.36
7.35
7.35
7.34
7.32
7.31
7.30
7.29
6.89
6.88
6.87
6.86
6.86
5.23
4.53
4.52
3.81
1.64
4.45
2.27
3.19
2.10
2.05
1.99
3.00
1.09
f1 (ppm)
N
Cbz
MeO

S10

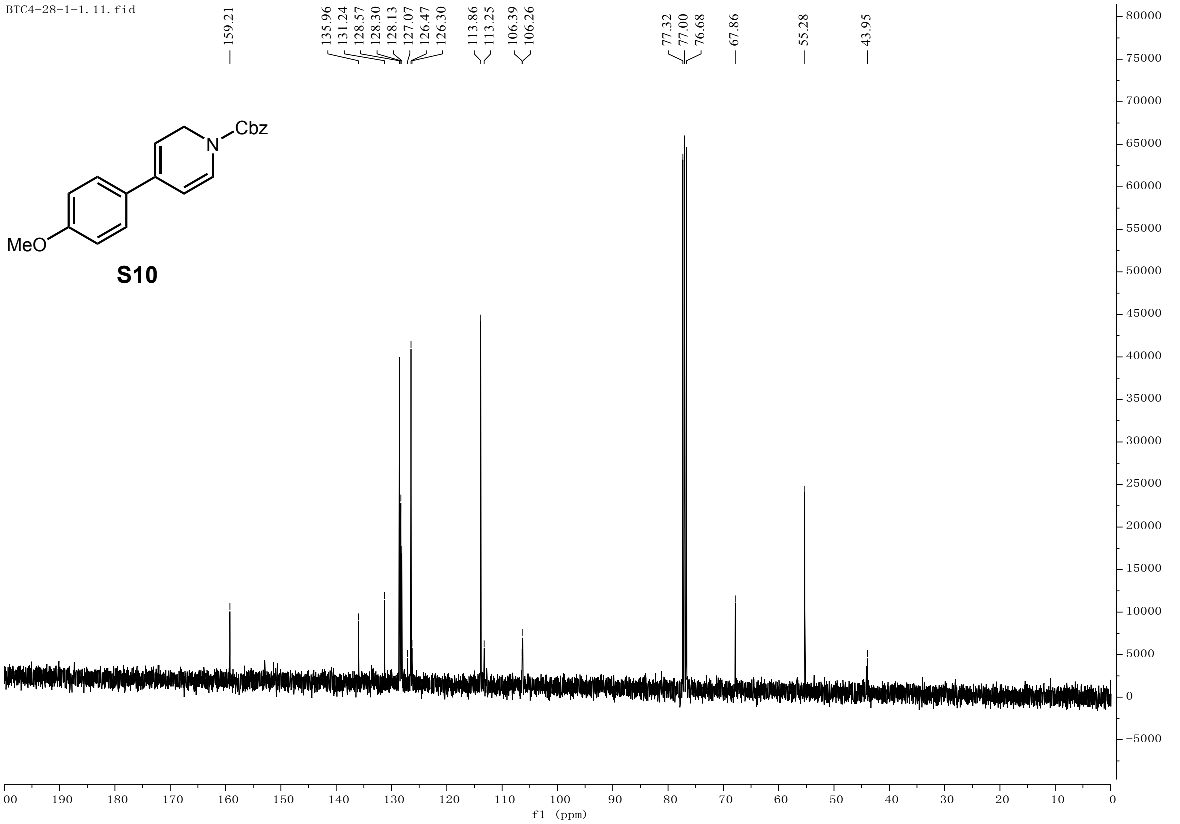

BTC4-28-1-1.11.fid
159.21
135.96
131.24
128.57
128.30
128.13
127.07
126.47
126.30
113.86
113.25
106.39
106.26
77.32
77.00
76.68
67.86
55.28
43.95
Cbz
N
MeO
S10
f1 (ppm)

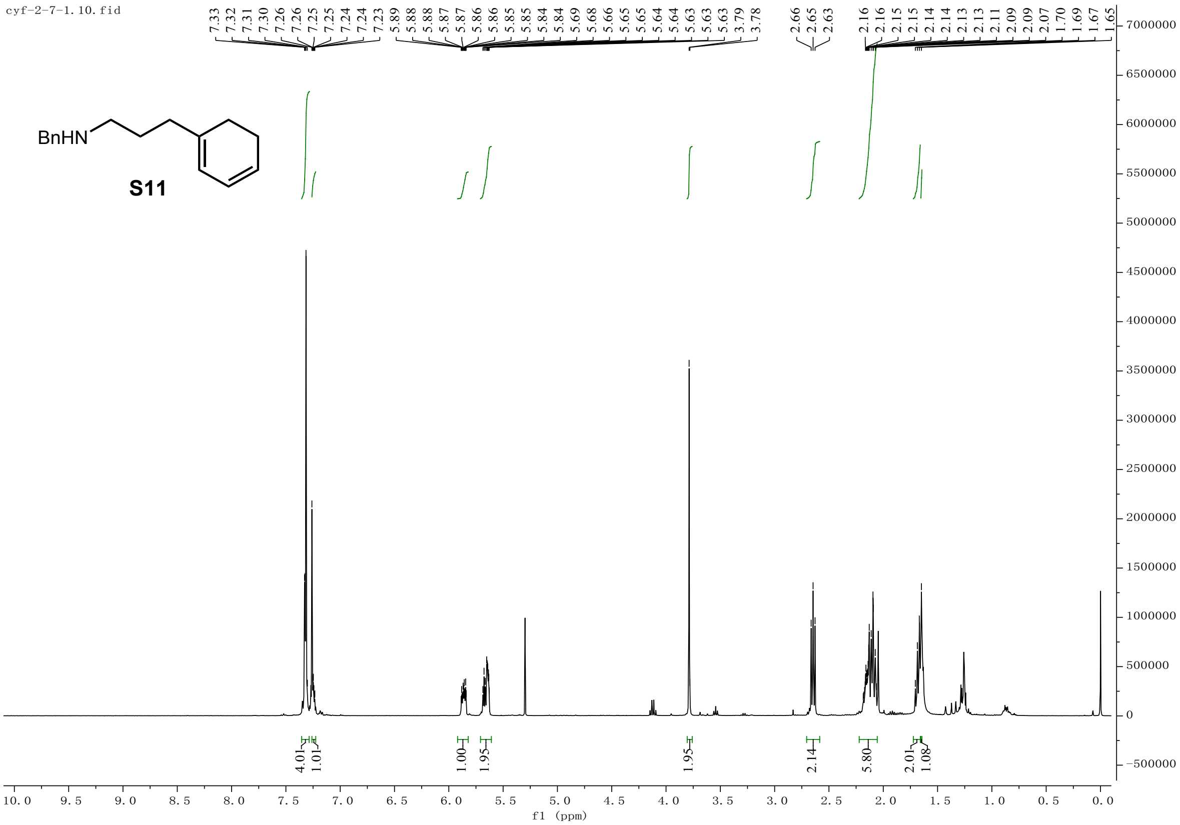

cyf-2-7-1.10.fid
BnHN
S11
4.01
1.01
1.00
1.95
1.95
2.14
5.80
2.01
1.08
f1 (ppm)

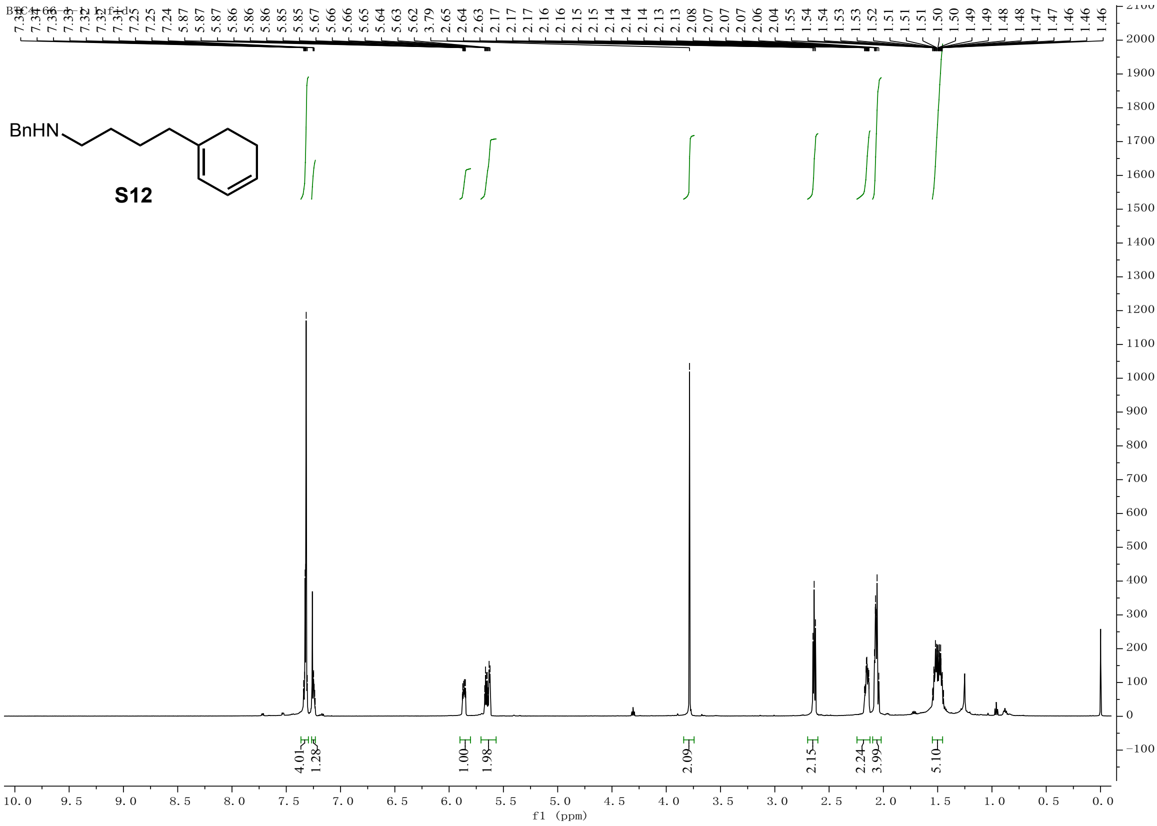
BnHN
S12
4.01
1.28
1.00
1.98
2.09
2.15
2.24
3.99
5.10
f1 (ppm)

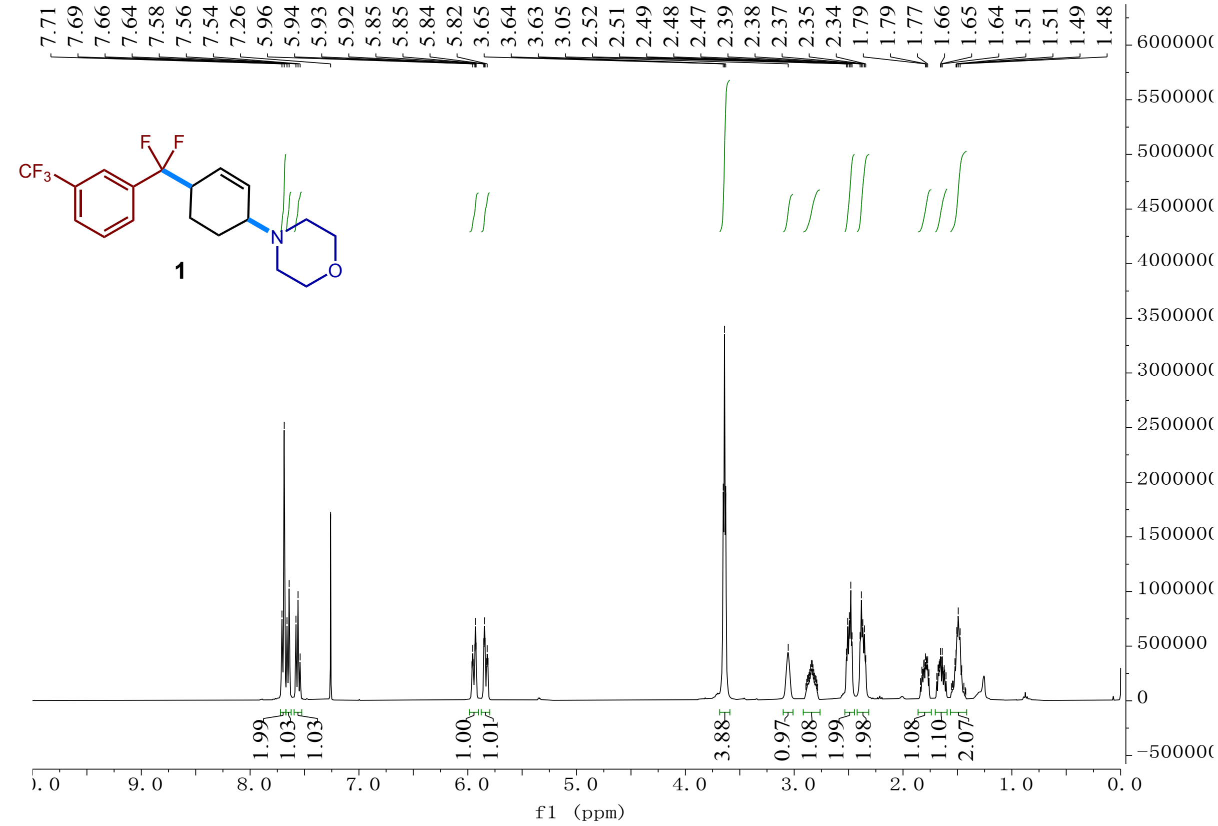
CF3
F
F
N
O
1
1.99
1.03
1.03
1.00
1.01
3.88
0.97
1.08
1.99
1.98
1.08
1.10
2.07
f1 (ppm)

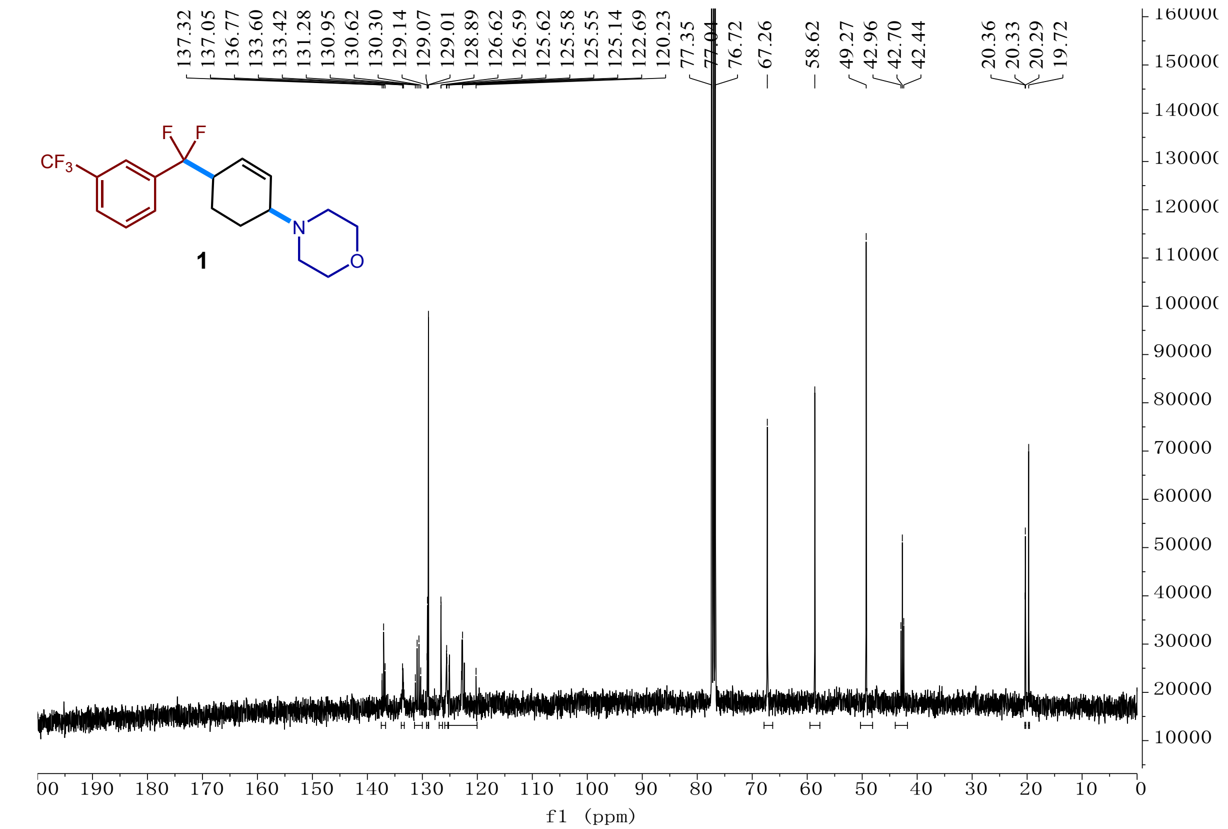
137.32
137.05
136.77
133.60
133.42
131.28
130.95
130.62
130.30
129.14
129.07
129.01
128.89
126.62
126.59
125.62
125.58
125.55
125.14
122.69
120.23
77.35
77.04
76.72
67.26
58.62
49.27
42.96
42.70
42.44
20.36
20.33
20.29
19.72
CF3
F
F
N
O
1
f1 (ppm)

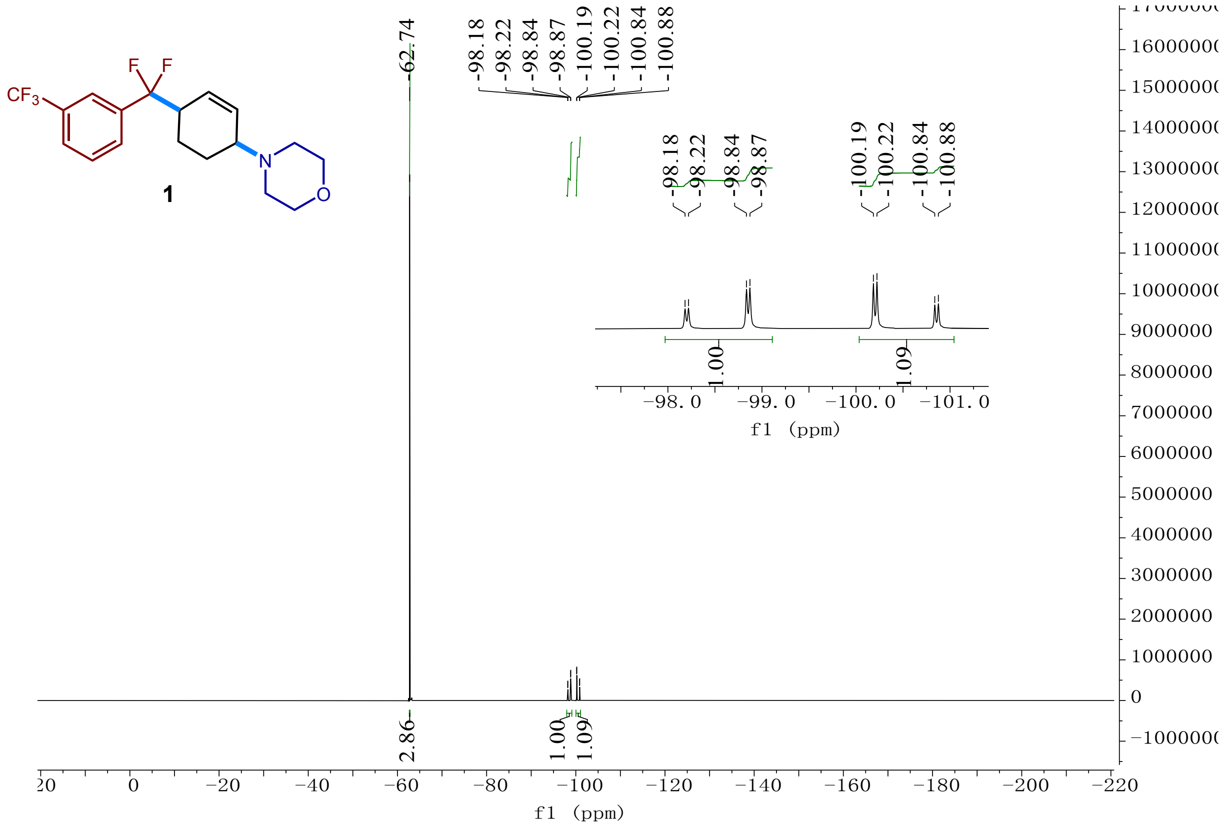
-62.74
-98.18
-98.22
-98.84
-98.87
-100.19
-100.22
-100.84
-100.88
CF3
F
F
N
O
1
1.00
1.09
2.86
f1 (ppm)

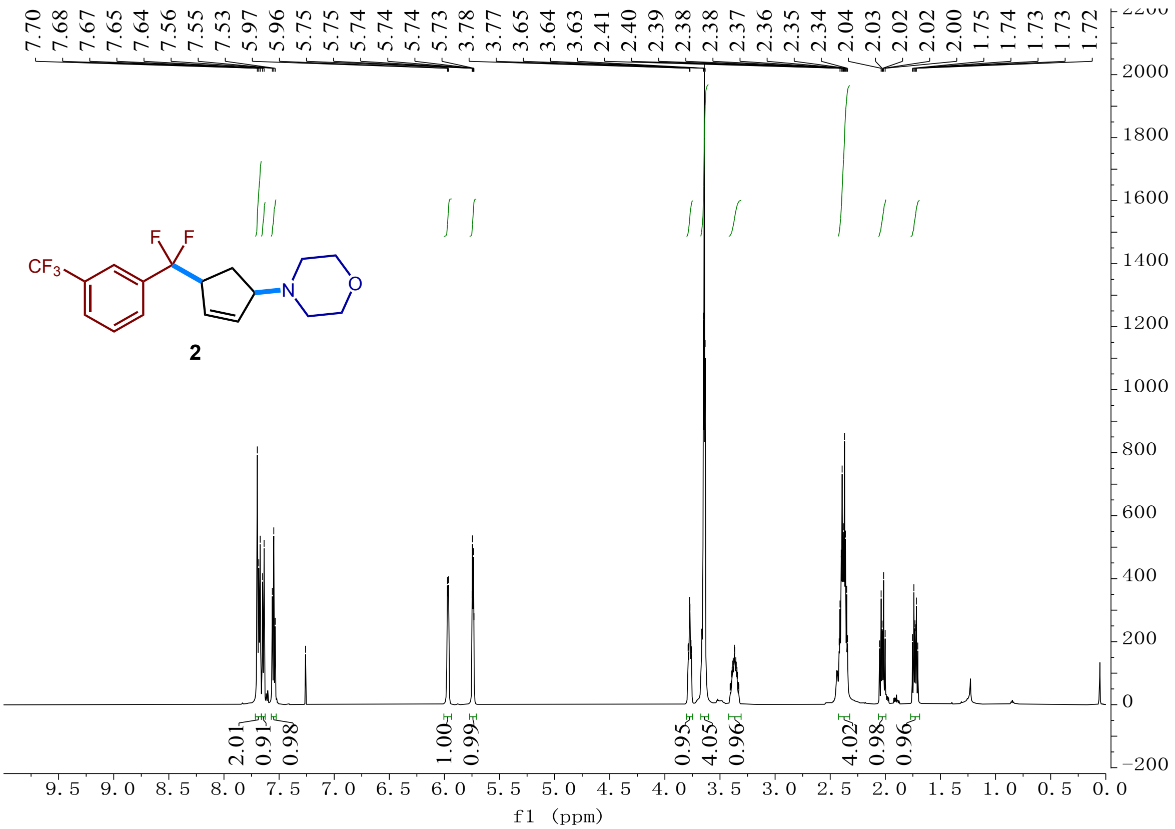
7.70 7.68 7.67 7.65 7.64 7.56 7.55 7.53 5.97 5.96 5.75 5.75 5.74 5.74 5.74 5.73 3.78 3.77 3.65 3.64 3.63 2.41 2.40 2.39 2.38 2.38 2.37 2.36 2.35 2.34 2.04 2.03 2.02 2.02 2.00 1.75 1.74 1.73 1.73 1.72
2
2.01 0.91 0.98 1.00 0.99 0.95 4.05 0.96 4.02 0.98 0.96
f1 (ppm)

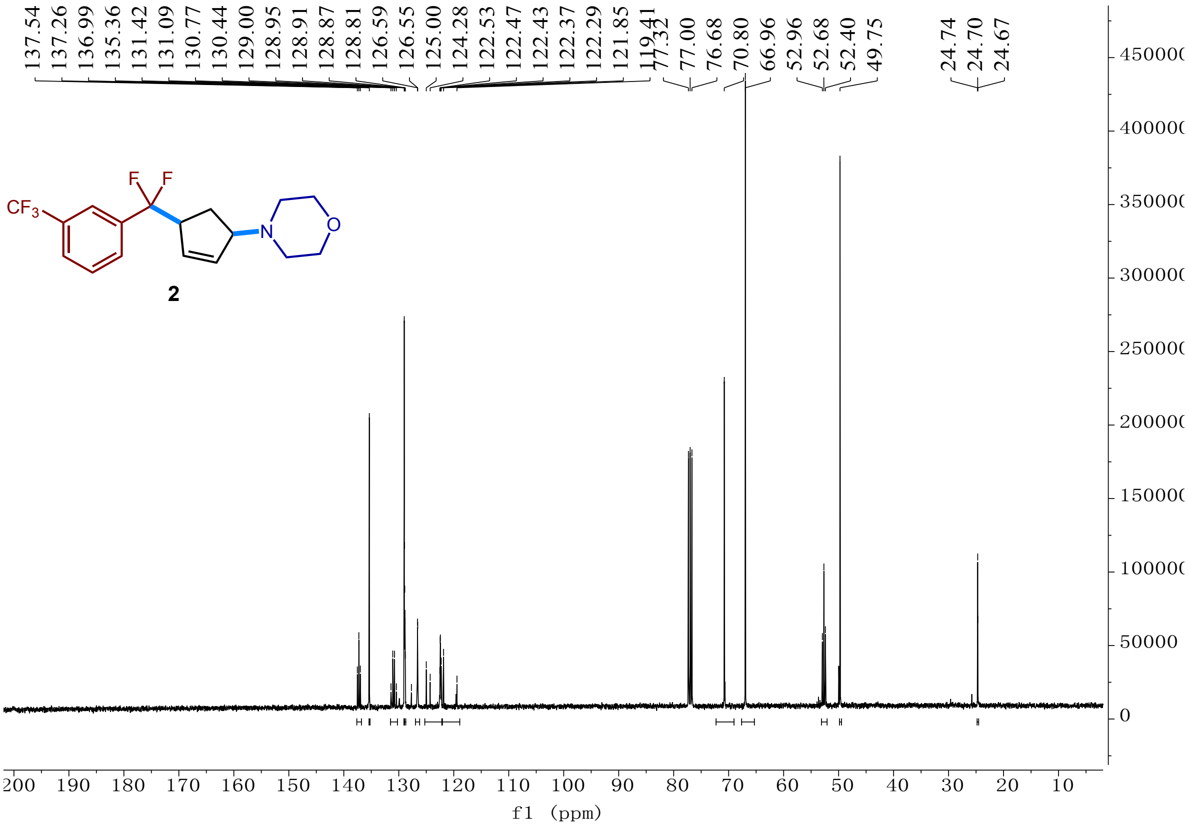
137.54 137.26 136.99 135.36 131.42 131.09 130.77 130.44 129.00 128.95 128.91 128.87 128.81 126.59 126.55 125.00 124.28 122.53 122.47 122.43 122.37 122.29 121.85 119.41 77.32 77.00 76.68 70.80 66.96 52.96 52.68 52.40 49.75 24.74 24.70 24.67
2
f1 (ppm)

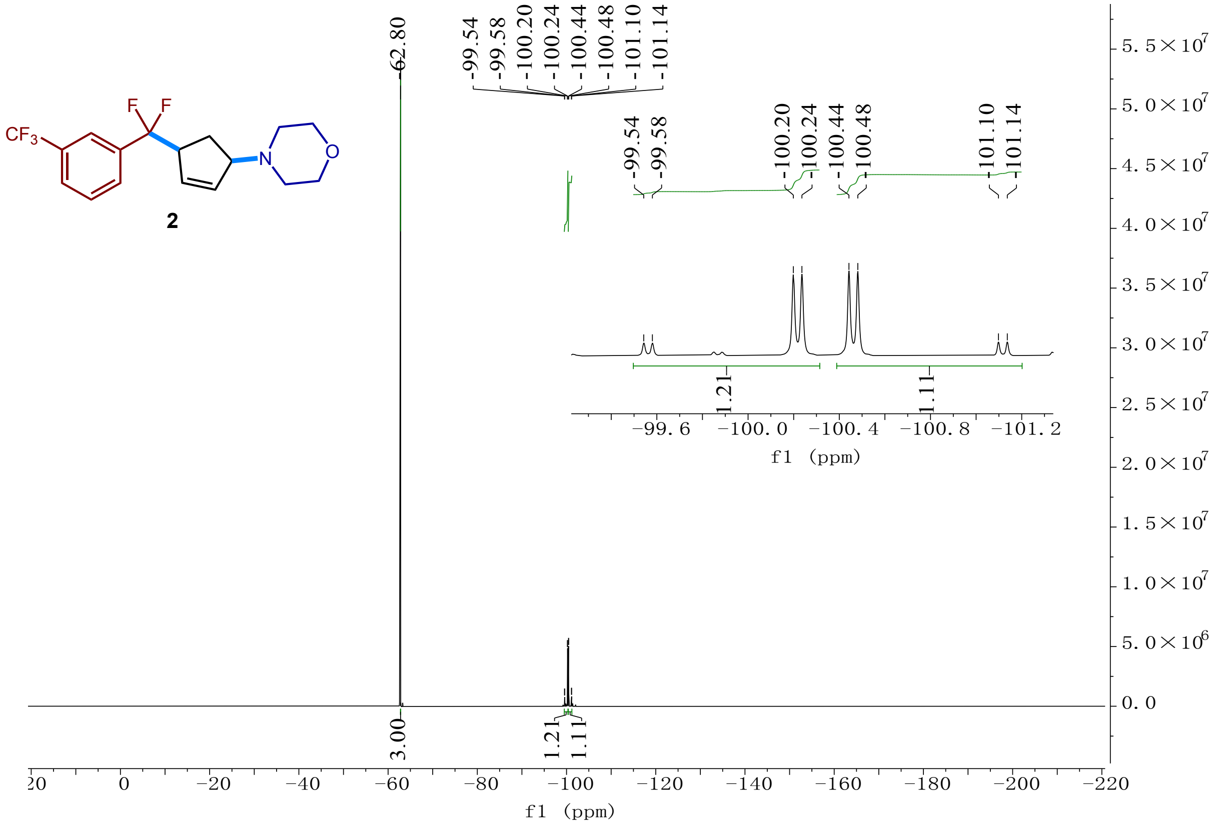
CF3
F F
N
O
2
-62.80
-99.54
-99.58
-100.20
-100.24
-100.44
-100.48
-101.10
-101.14
1.21
1.11
3.00
f1 (ppm)

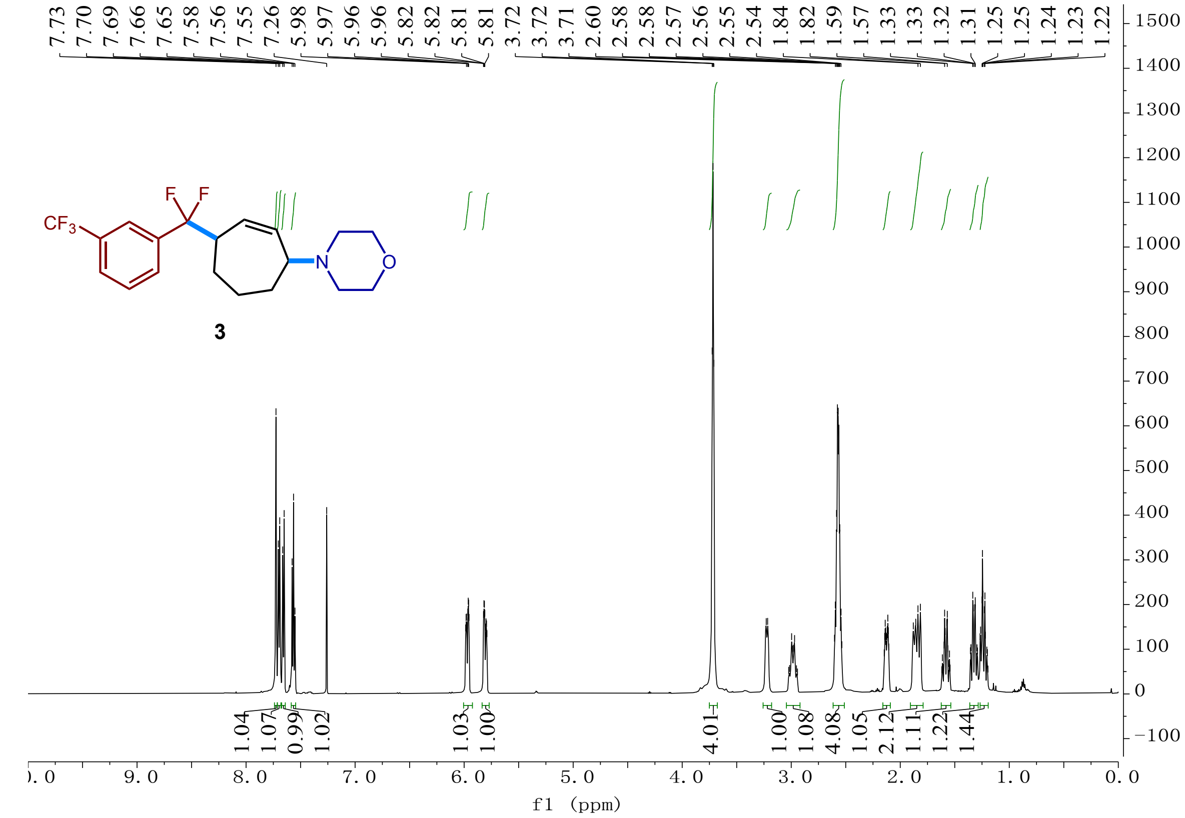
CF3
F F
N
O
3
7.73 7.70 7.69 7.66 7.65 7.58 7.56 7.55 7.26 5.98 5.97 5.96 5.96 5.82 5.82 5.81 5.81 3.72 3.72 3.71 2.60 2.58 2.58 2.57 2.56 2.55 2.54 1.84 1.82 1.59 1.57 1.33 1.33 1.32 1.31 1.25 1.25 1.24 1.23 1.22
1.04 1.07 0.99 1.02 1.03 1.00 4.01 1.00 1.08 4.08 1.05 2.12 1.11 1.22 1.44
f1 (ppm)

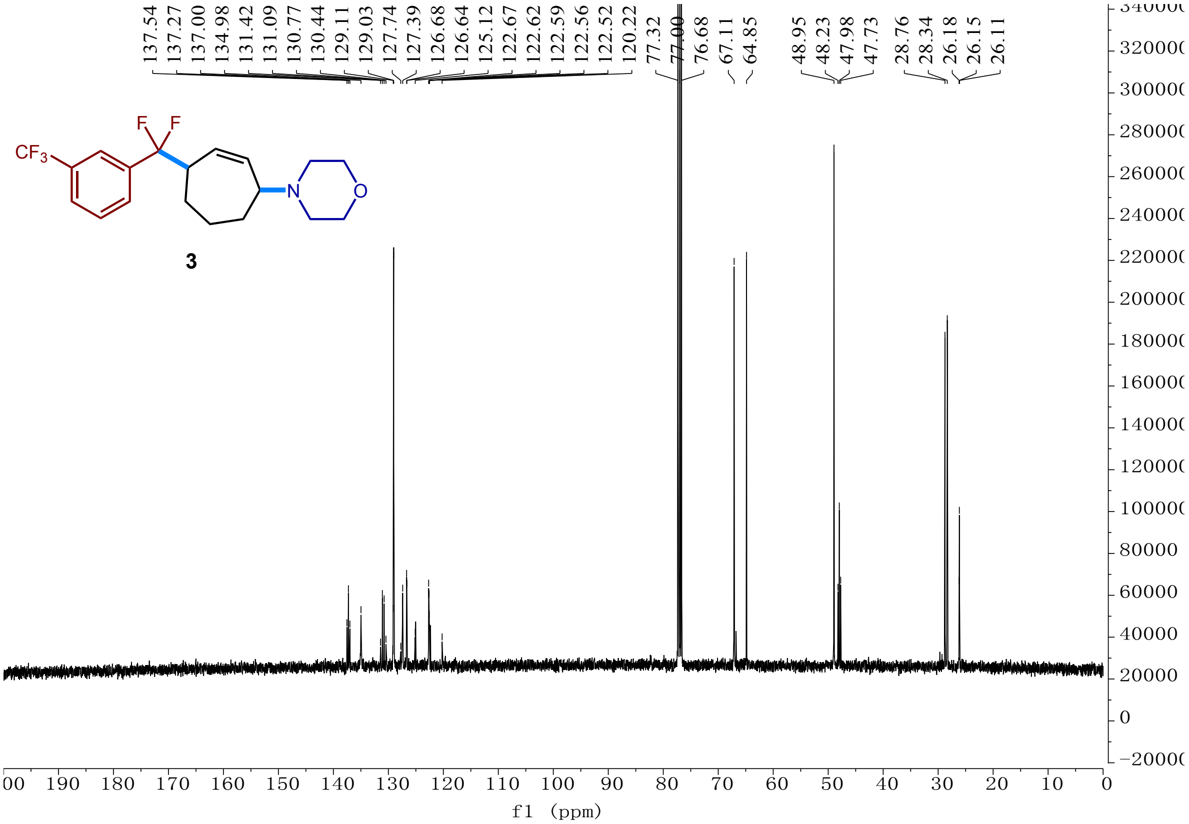

137.54
137.27
137.00
134.98
131.42
131.09
130.77
130.44
129.11
129.03
127.74
127.39
126.68
126.64
125.12
122.67
122.62
122.59
122.56
122.52
120.22
77.32
77.00
76.68
67.11
64.85
48.95
48.23
47.98
47.73
28.76
28.34
26.18
26.15
26.11
CF3
F
F
N
O
3
f1 (ppm)

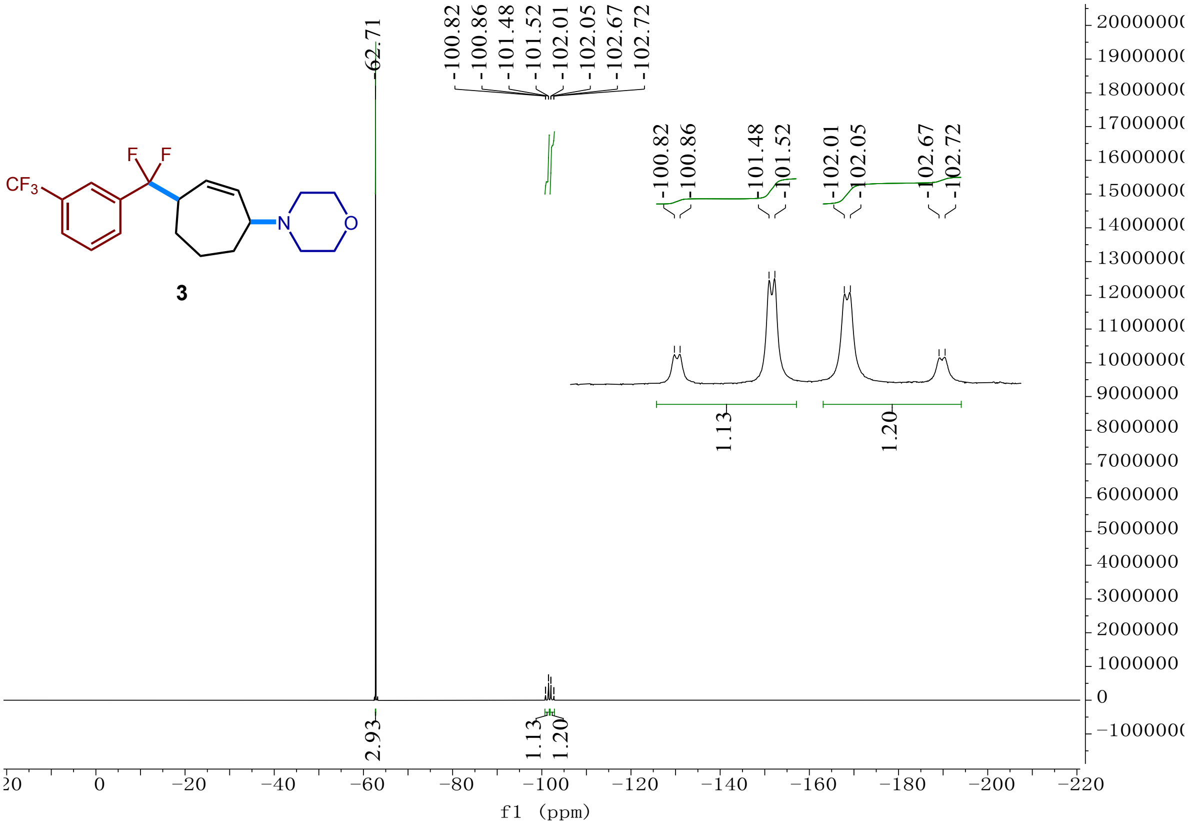

-62.71
-100.82
-100.86
-101.48
-101.52
-102.01
-102.05
-102.67
-102.72
CF3
F
F
N
O
3
1.13
1.20
2.93
f1 (ppm)

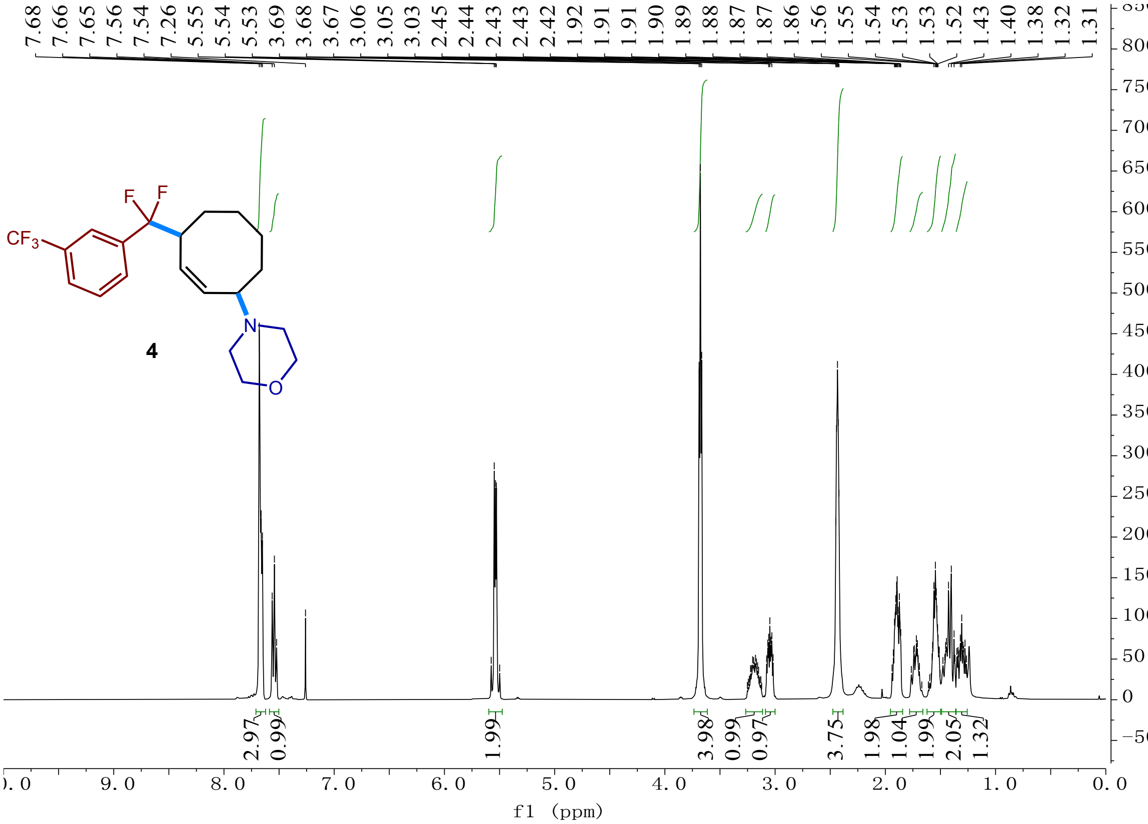
7.68 7.66 7.65 7.56 7.54 7.26 5.55 5.54 5.53 3.69 3.68 3.67 3.06 3.05 3.03 2.45 2.44 2.43 2.43 2.42 1.92 1.91 1.91 1.90 1.89 1.88 1.87 1.87 1.86 1.56 1.55 1.54 1.53 1.53 1.52 1.43 1.40 1.38 1.32 1.31
CF3
F
F
N
O
4
2.97 0.99 1.99 3.98 0.99 0.97 3.75 1.98 1.04 1.99 2.05 1.32
f1 (ppm)

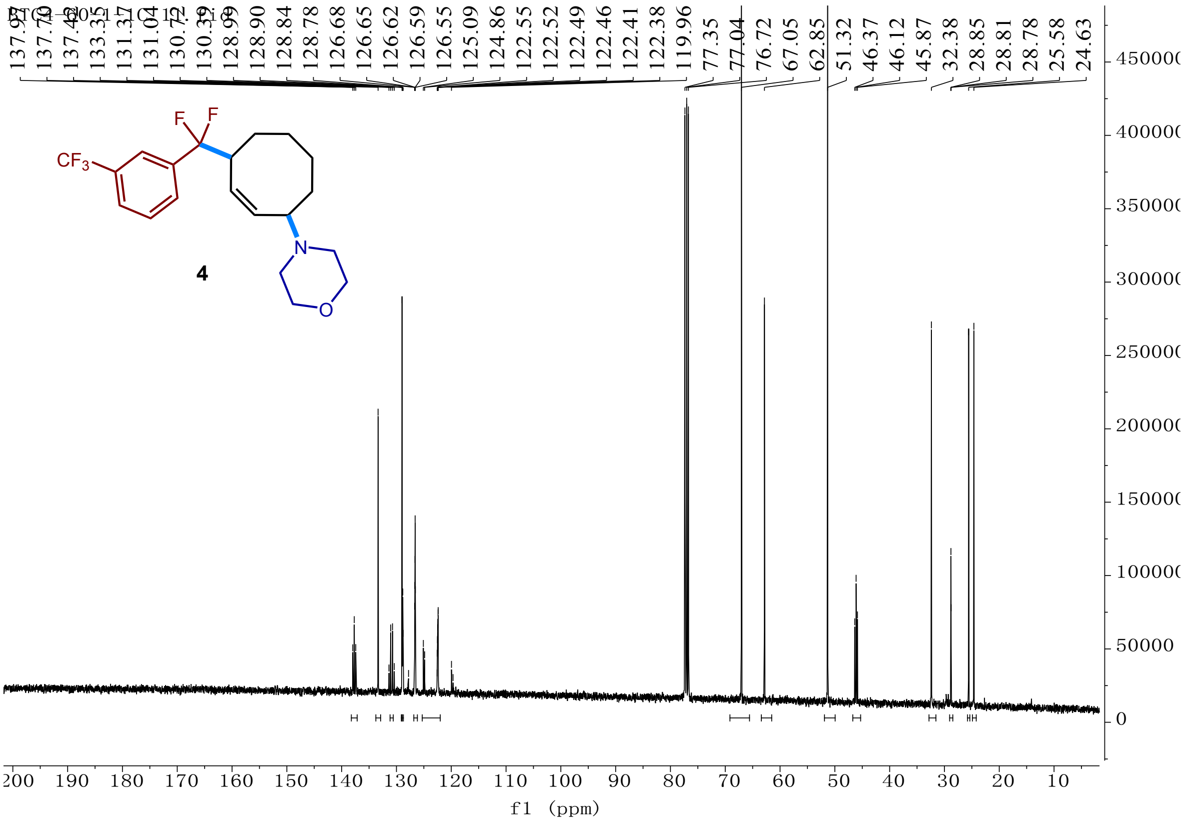
137.74 137.42 133.35 131.31 131.04 130.72 130.39 128.99 128.90 128.84 128.78 126.68 126.65 126.62 126.59 126.55 125.09 124.86 122.55 122.52 122.49 122.46 122.41 122.38 119.96 77.35 77.04 76.72 67.05 62.85 51.32 46.37 46.12 45.87 32.38 28.85 28.81 28.78 25.58 24.63
CF3
F
F
N
O
4
f1 (ppm)

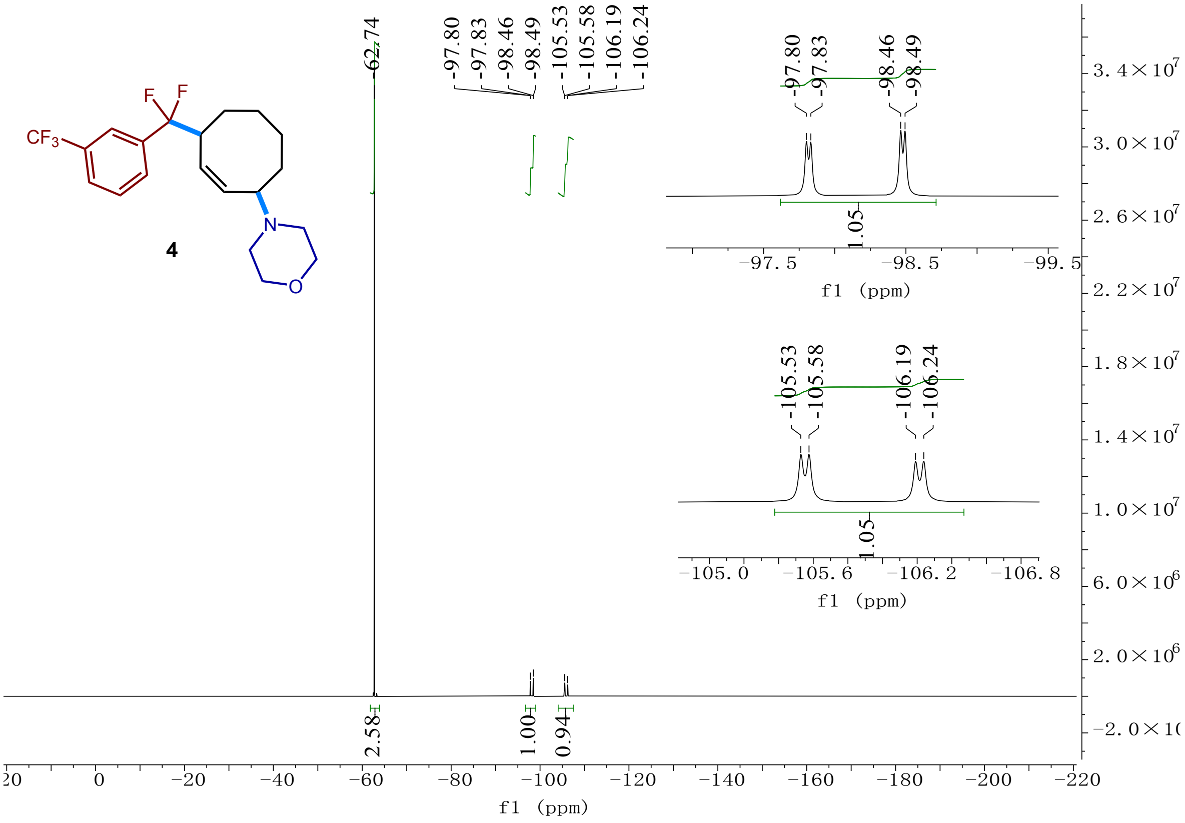
-62.74
-97.80
-97.83
-98.46
-98.49
-105.53
-105.58
-106.19
-106.24
CF3
F
F
N
O
4
2.58
1.00
0.94
1.05
1.05
f1 (ppm)

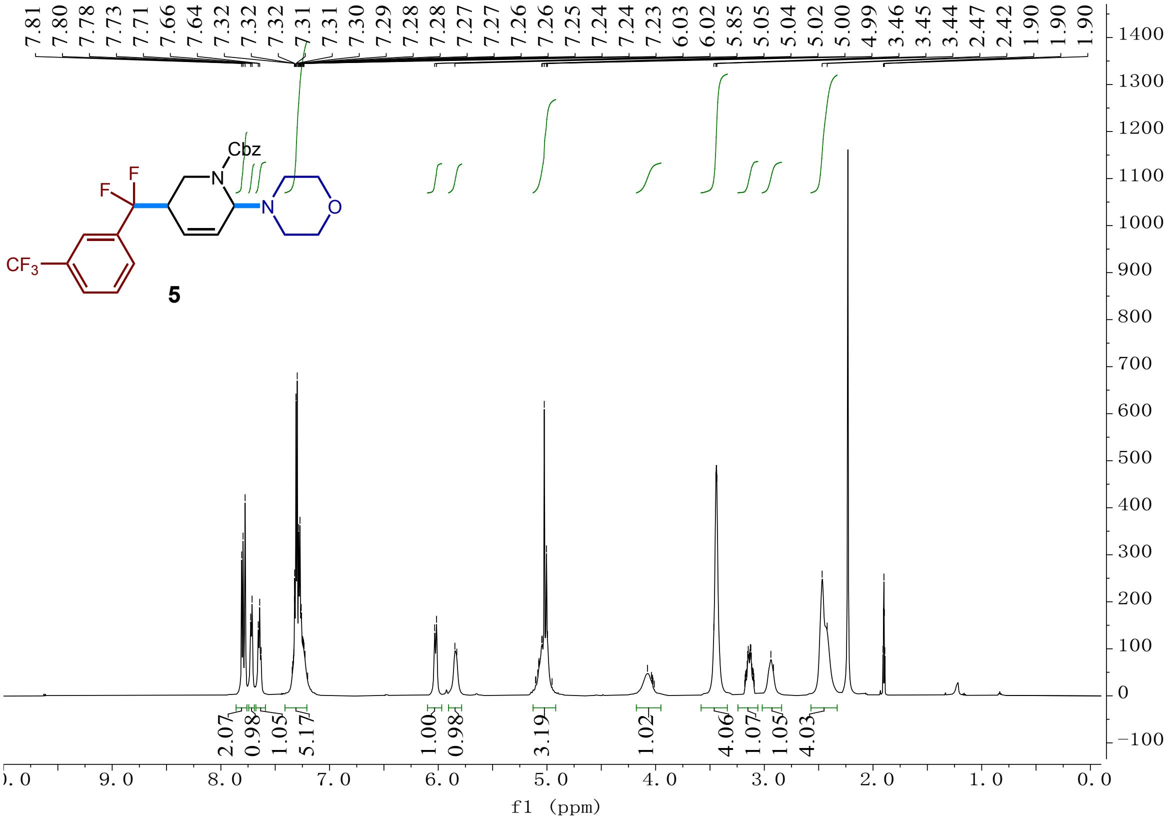
7.81 7.80 7.78 7.73 7.71 7.66 7.64 7.32 7.32 7.32 7.31 7.31 7.30 7.29 7.28 7.28 7.27 7.27 7.26 7.26 7.25 7.24 7.24 7.23 6.03 6.02 5.85 5.05 5.04 5.02 5.00 4.99 3.46 3.45 3.44 2.47 2.42 1.90 1.90 1.90
Cbz
N
F
F
N
O
CF3
5
2.07
0.98
1.05
5.17
1.00
0.98
3.19
1.02
4.06
1.07
1.05
4.03
f1 (ppm)

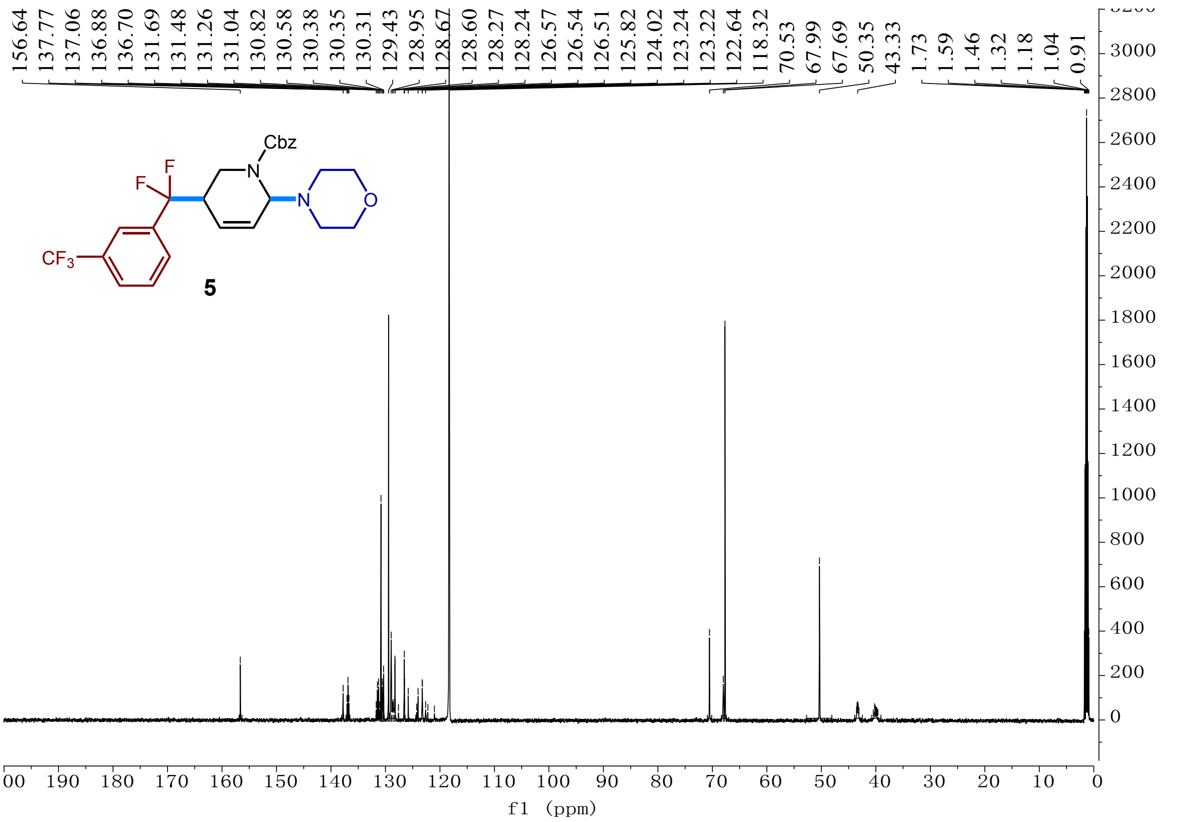
156.64
137.77
137.06
136.88
136.70
131.69
131.48
131.26
131.04
130.82
130.58
130.38
130.35
130.31
129.43
128.95
128.67
128.60
128.27
128.24
126.57
126.54
126.51
125.82
124.02
123.24
123.22
122.64
118.32
70.53
67.99
67.69
50.35
43.33
1.73
1.59
1.46
1.32
1.18
1.04
0.91
Cbz
N
F
F
N
O
CF_3
5
f1 (ppm)

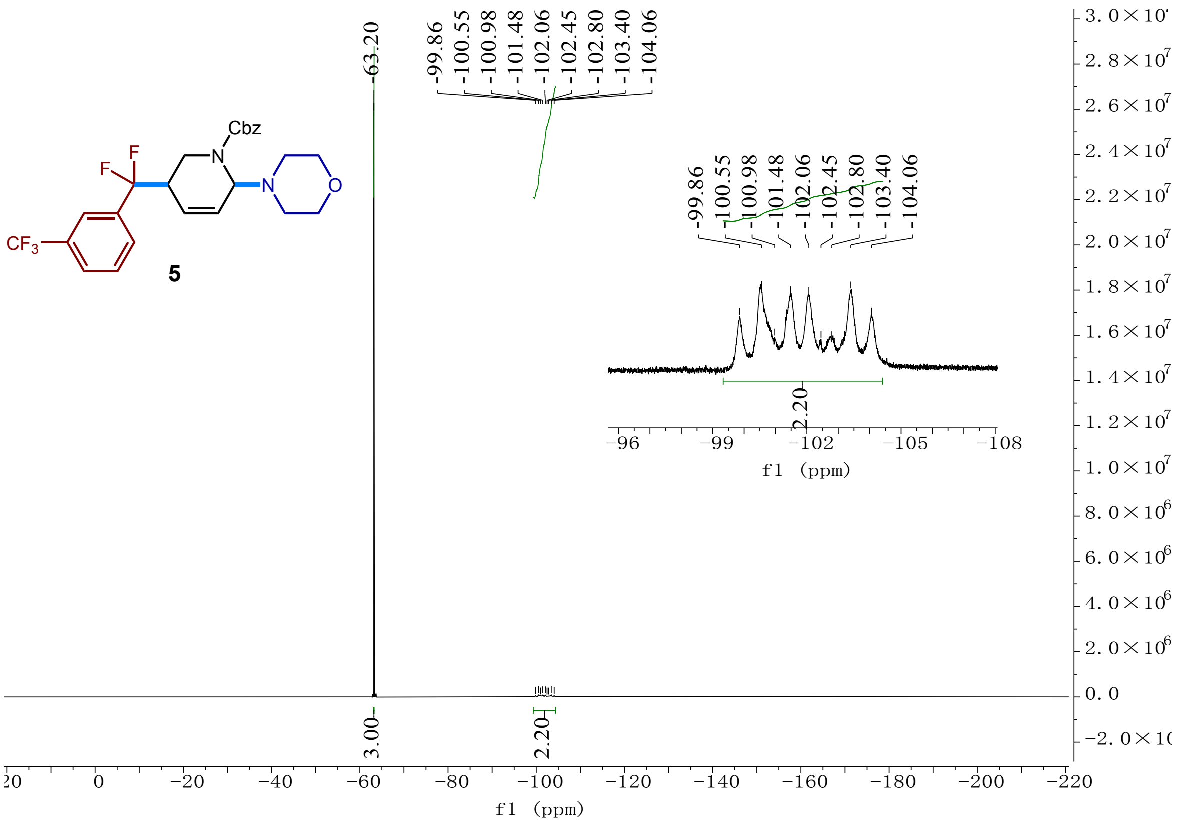
-63.20
-99.86
-100.55
-100.98
-101.48
-102.06
-102.45
-102.80
-103.40
-104.06
Cbz
N
F
F
N
O
CF_3
5
2.20
3.00
f1 (ppm)

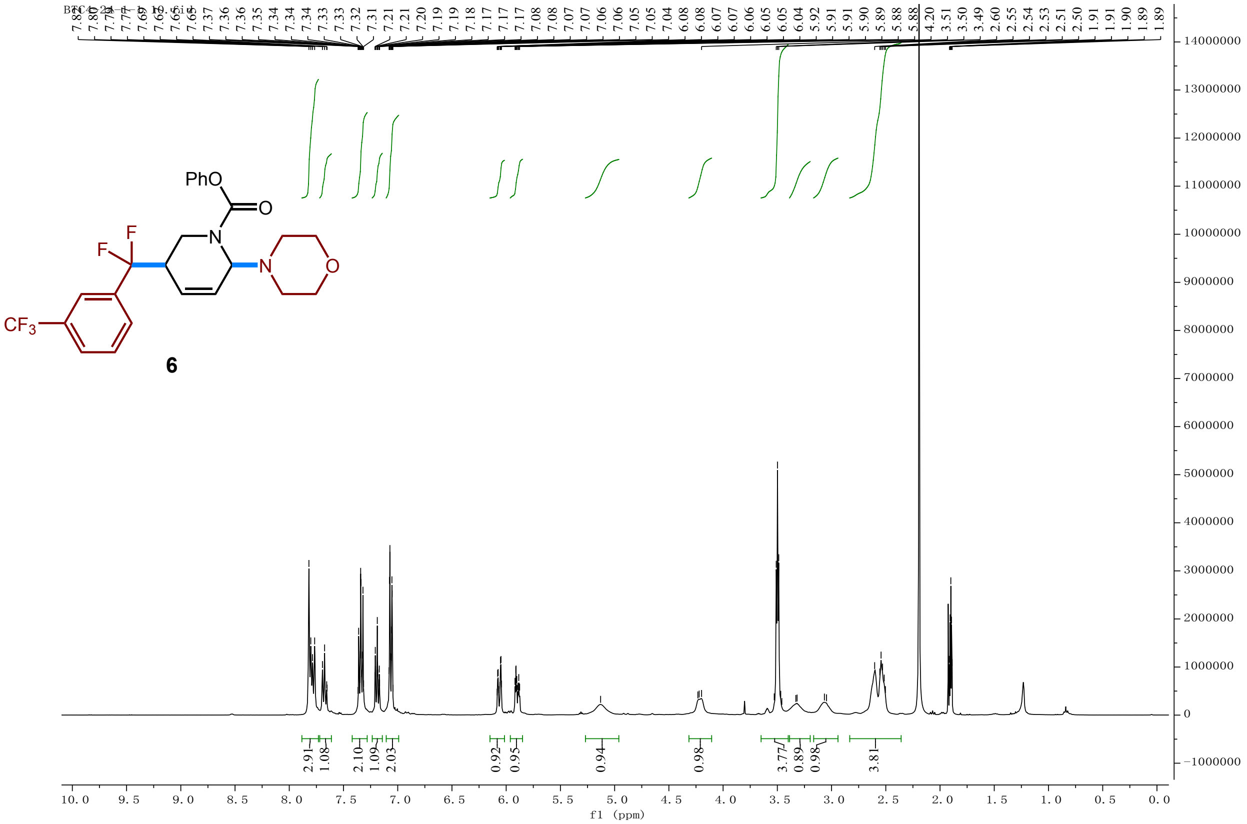
PhO
O
F
F
N
N
O
CF3
6
f1 (ppm)

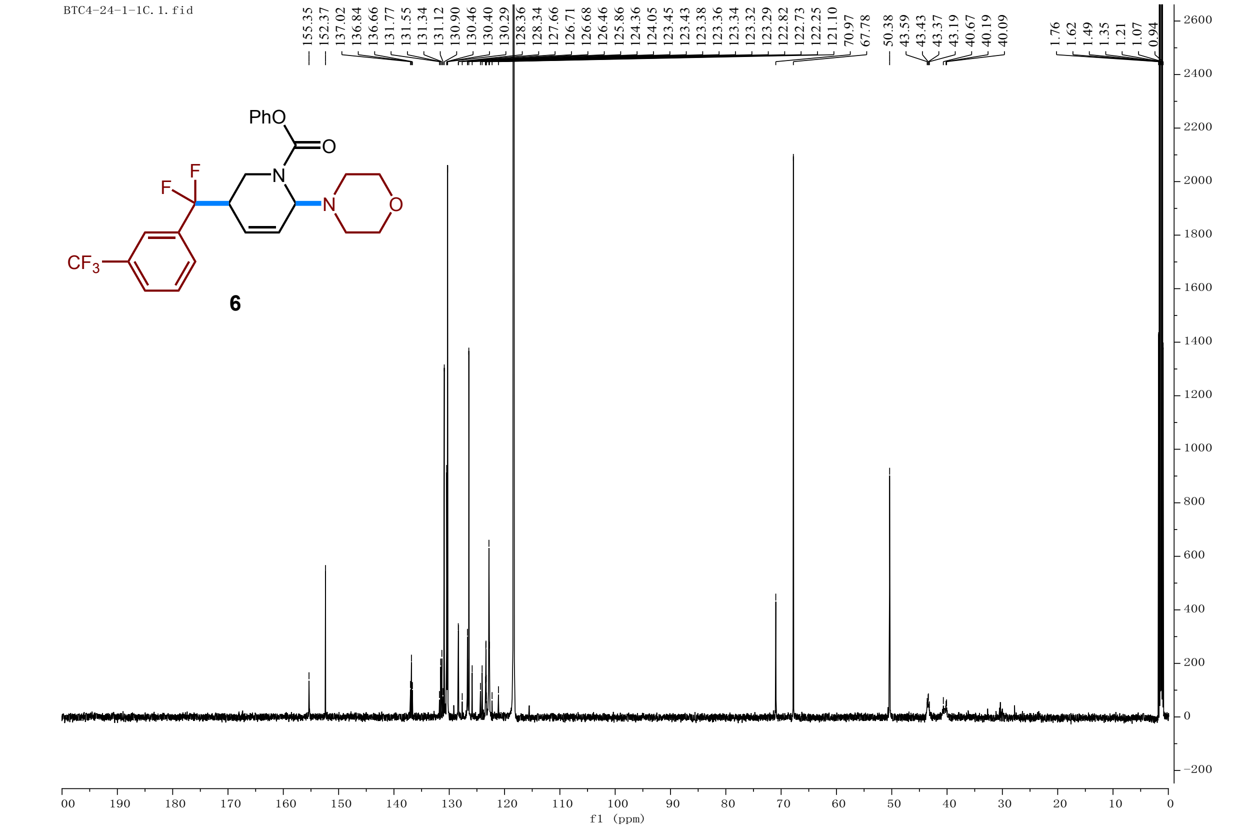
BTC4-24-1-1C.1.fid
PhO
O
F
F
N
N
O
CF3
6
f1 (ppm)

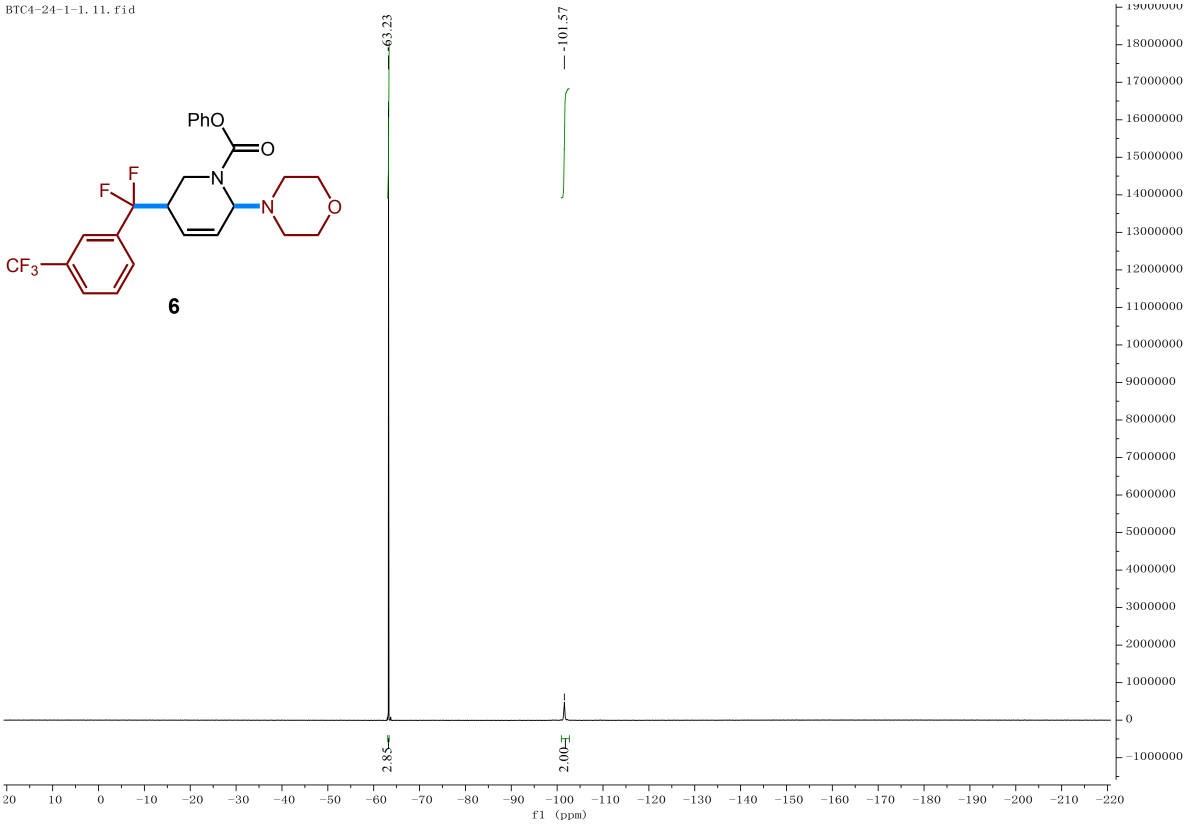
BTC4-24-1-1.11.fid
-63.23
-101.57
PhO
O
F
F
N
N
O
CF3
6
2.85
2.00
f1 (ppm)

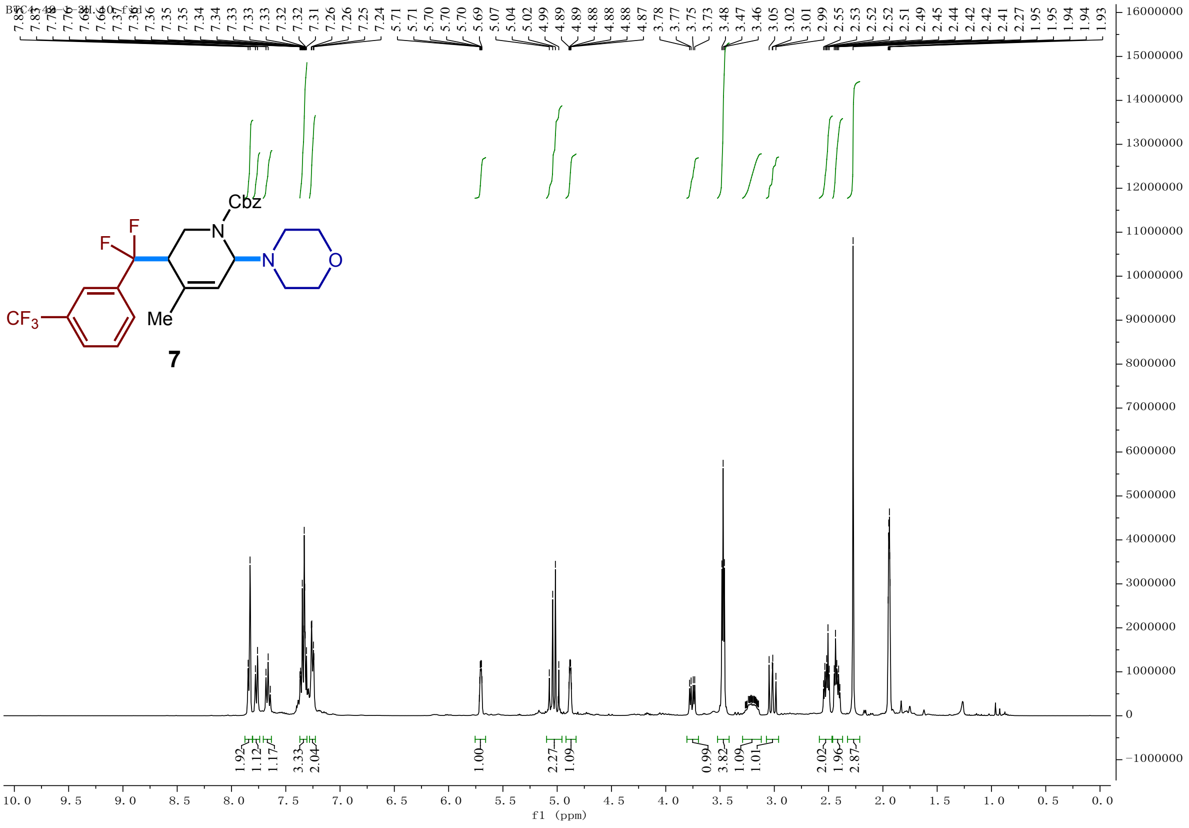
7.83
7.78
7.76
7.66
7.64
7.37
7.36
7.35
7.35
7.34
7.34
7.33
7.33
7.33
7.32
7.32
7.31
7.26
7.25
7.24
5.71
5.71
5.70
5.70
5.69
5.07
5.04
5.02
4.99
4.89
4.89
4.88
4.88
4.87
3.78
3.77
3.75
3.73
3.48
3.47
3.46
3.05
3.02
3.01
2.99
2.55
2.53
2.52
2.52
2.51
2.49
2.45
2.44
2.42
2.42
2.41
2.27
1.95
1.94
1.94
1.93
Cbz
F
F
N
N
O
CF3
Me
7
1.92
1.12
1.17
3.33
2.04
1.00
2.27
1.09
0.99
3.82
1.09
1.01
2.02
1.96
2.87
f1 (ppm)

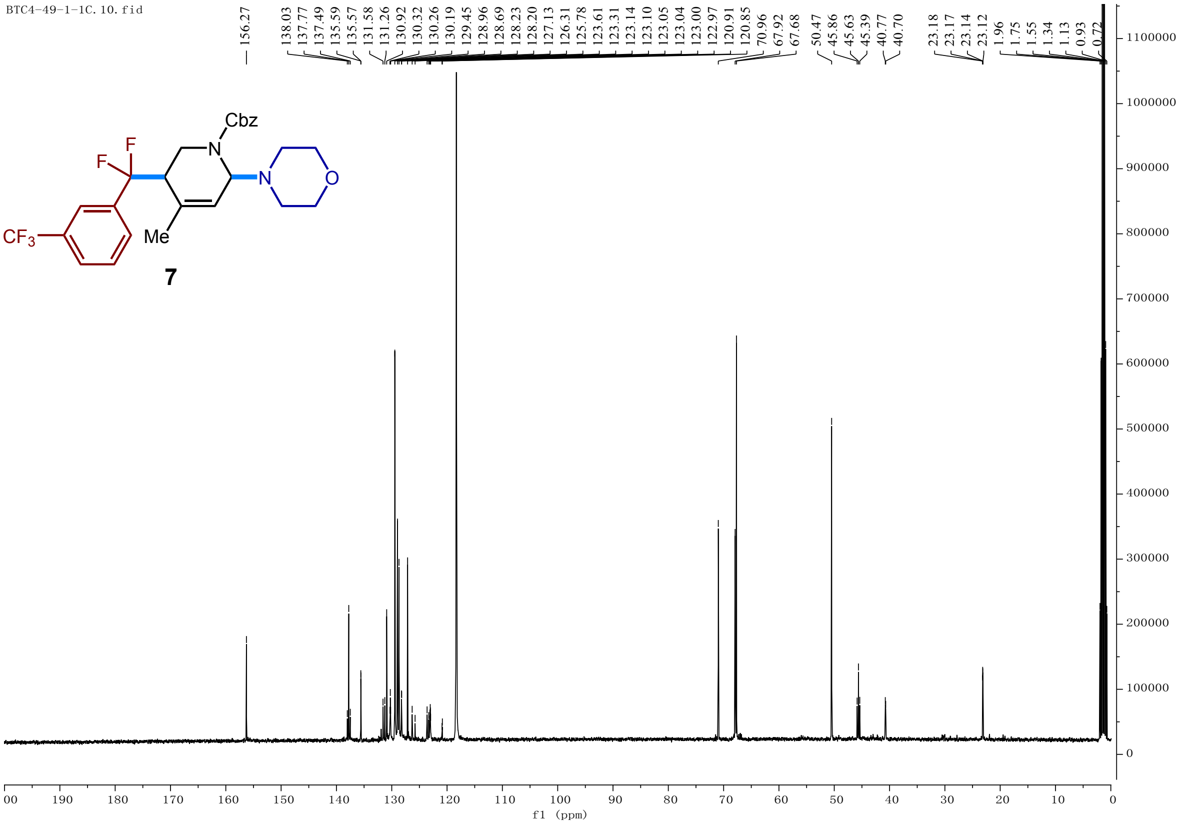
BTC4-49-1-1C.10.fid
156.27
138.03
137.77
137.49
135.59
135.57
131.58
131.26
130.92
130.32
130.26
130.19
129.45
128.96
128.69
128.23
128.20
127.13
126.31
125.78
123.61
123.31
123.14
123.10
123.05
123.04
123.00
122.97
120.91
120.85
70.96
67.92
67.68
50.47
45.86
45.63
45.39
40.77
40.70
23.18
23.17
23.14
23.12
1.96
1.75
1.55
1.34
1.13
0.93
0.72
Cbz
N
F
F
N
O
CF3
Me
7
f1 (ppm)

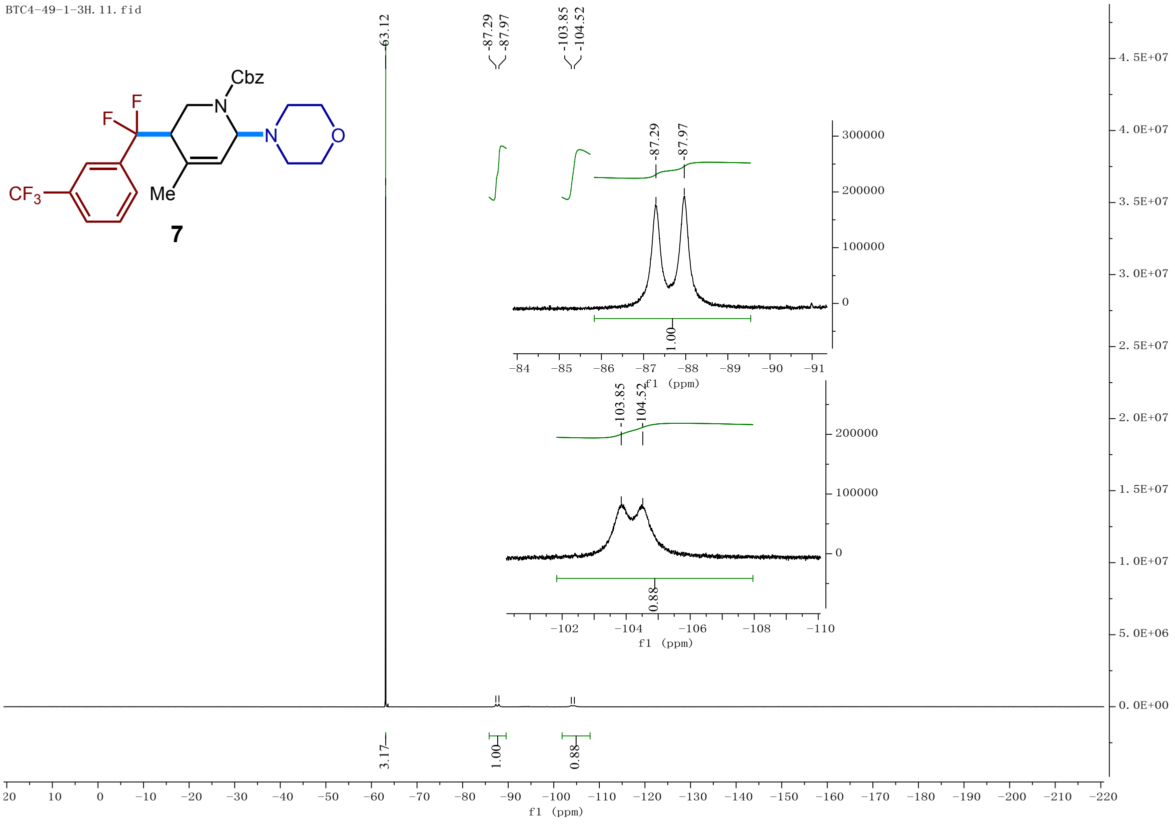
BTC4-49-1-3H.11.fid
-63.12
-87.29
-87.97
-103.85
-104.52
Cbz
N
F
F
N
O
CF3
Me
7
1.00
0.88
3.17
f1 (ppm)

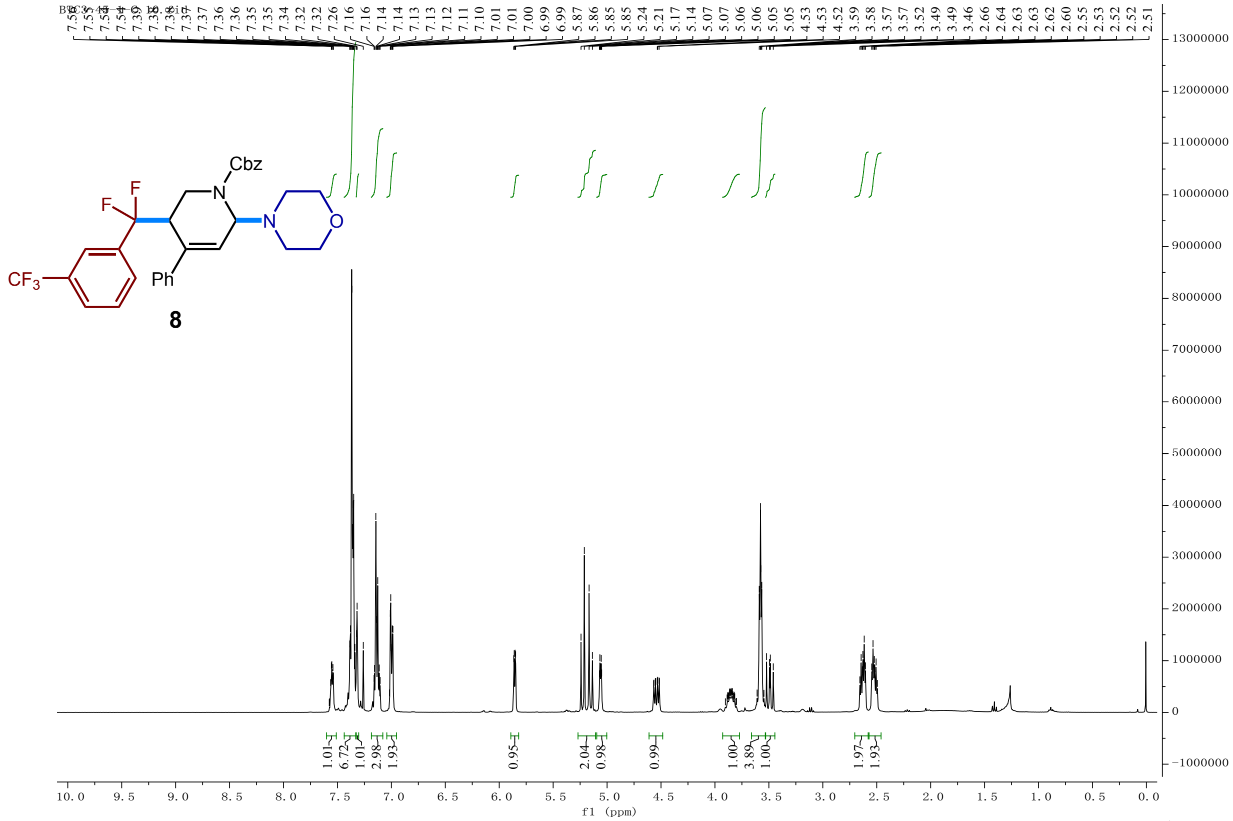
Cbz
F
F
N
N
O
CF3
Ph
8
f1 (ppm)

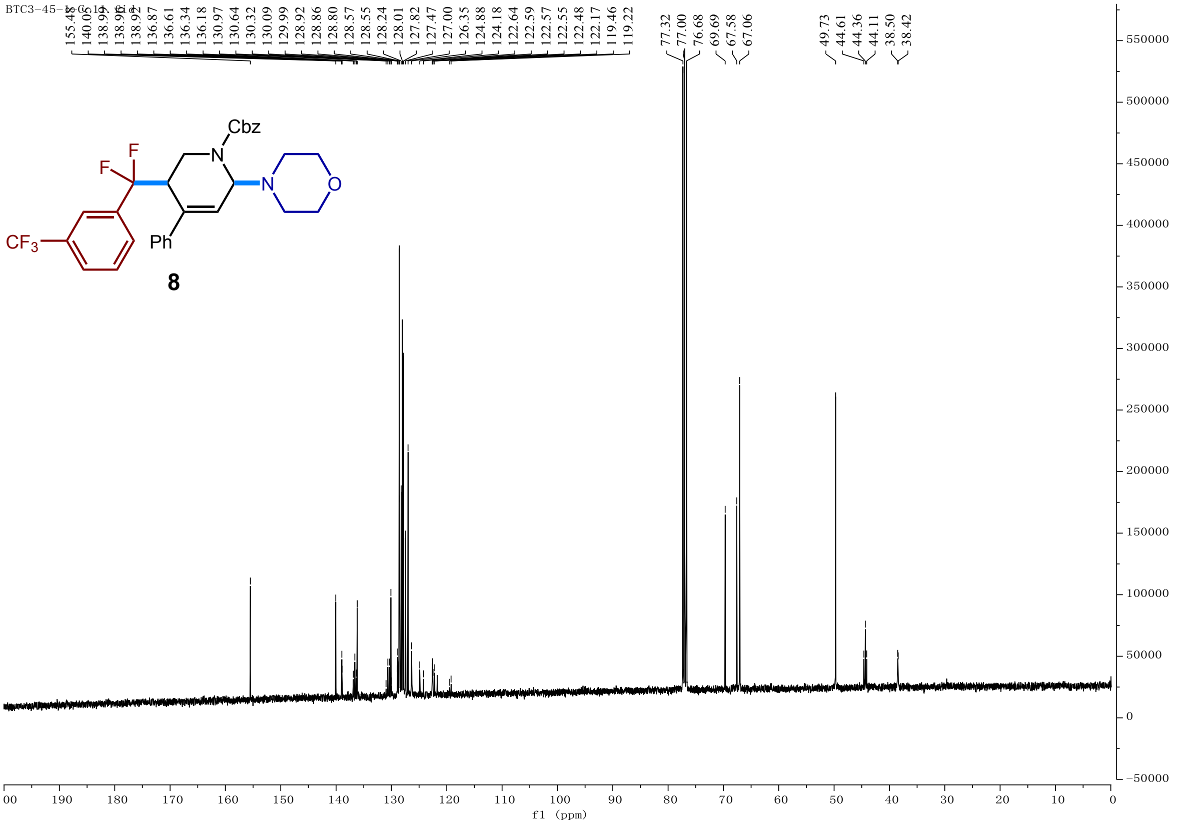
Cbz
F
F
N
N
O
CF3
Ph
8
f1 (ppm)

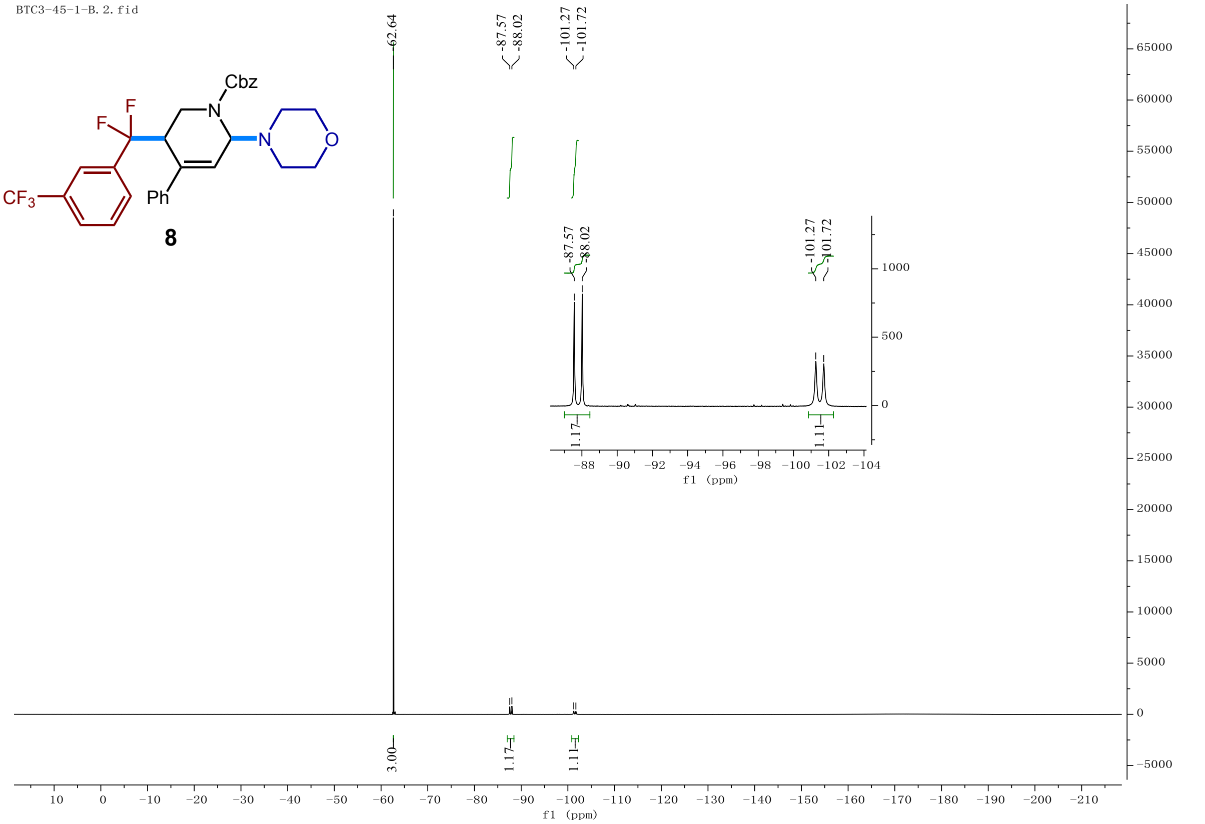
BTC3-45-1-B.2.fid
-62.64
-87.57
-88.02
-101.27
-101.72
Cbz
F
F
N
N
O
CF3
Ph
8
3.00
1.17
1.11
f1 (ppm)

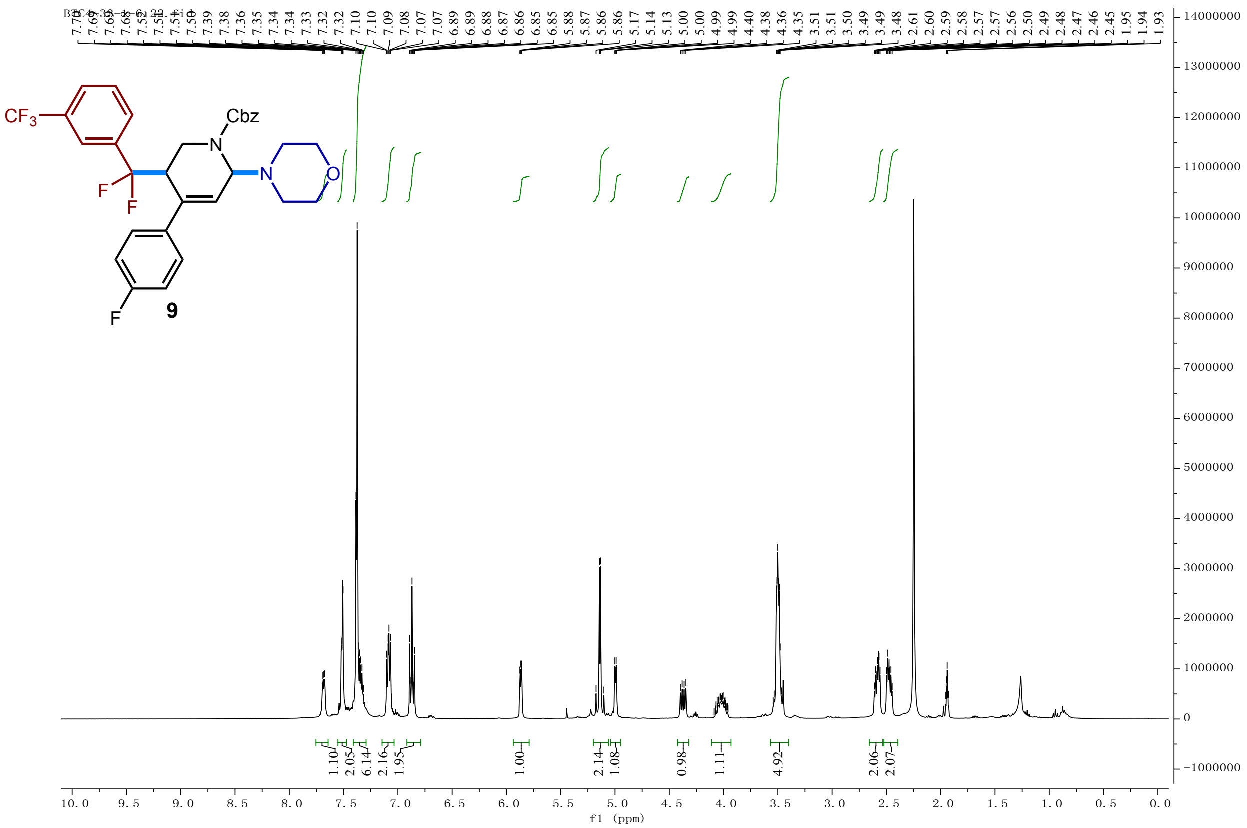
7.39
7.38
7.36
7.35
7.34
7.34
7.33
7.32
7.32
7.10
7.10
7.09
7.08
7.07
7.07
6.89
6.89
6.88
6.87
6.86
6.85
6.85
5.88
5.87
5.86
5.17
5.14
5.13
5.00
4.99
4.99
4.40
4.38
4.36
4.35
3.51
3.51
3.50
3.49
3.49
3.48
2.61
2.60
2.59
2.58
2.57
2.57
2.56
2.50
2.49
2.48
2.47
2.46
2.45
1.95
1.94
1.93
CF3
Cbz
N
N
O
F
F
F
9
1.10
2.05
6.14
2.16
1.95
1.00
2.14
1.08
0.98
1.11
4.92
2.06
2.07
f1 (ppm)

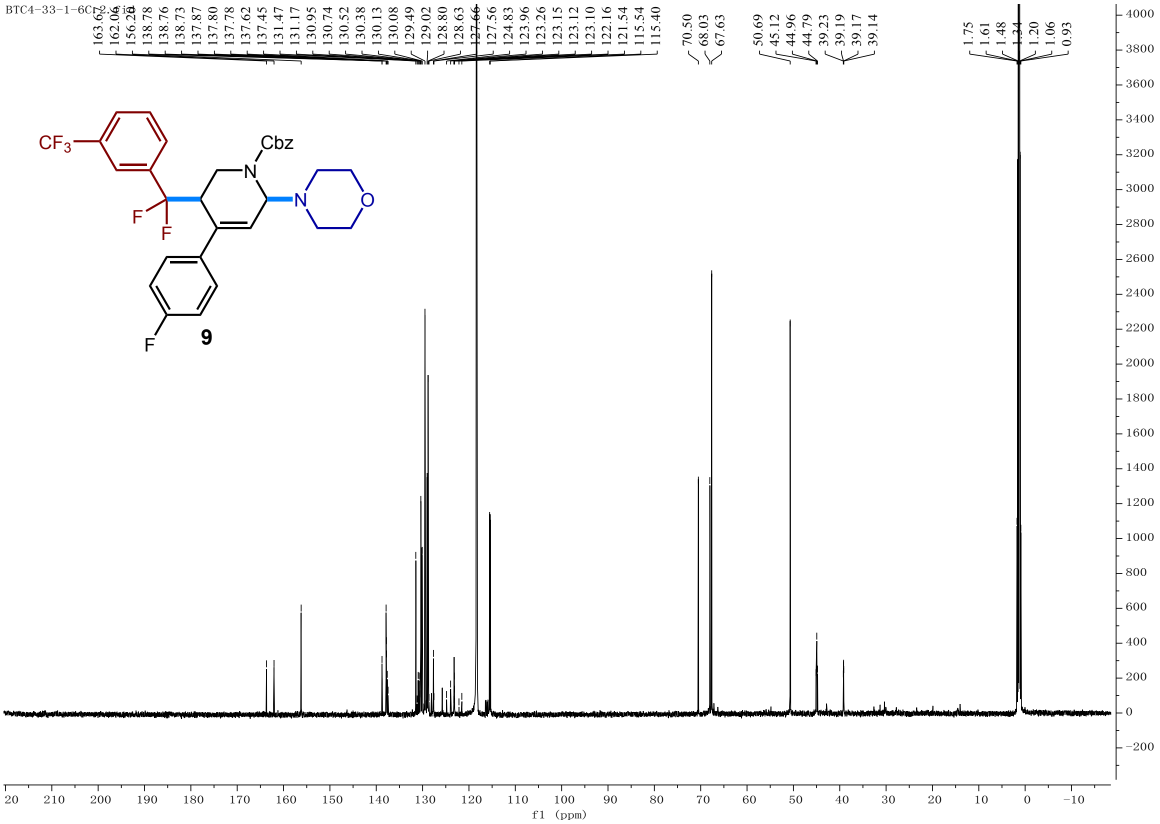
BTC4-33-1-6C.2.fid
f1 (ppm)
CF3
Cbz
N
O
F
F
F
9

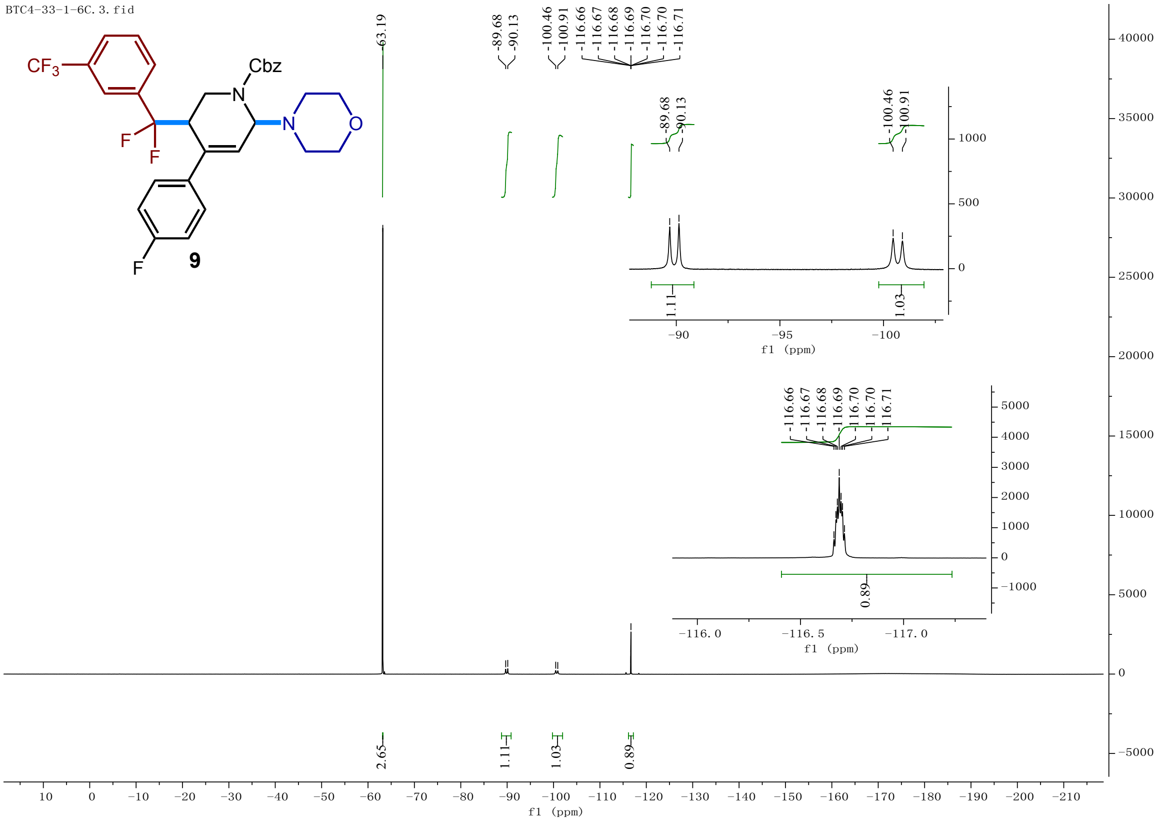
BTC4-33-1-6C.3.fid
f1 (ppm)
CF3
Cbz
N
O
F
F
F
9

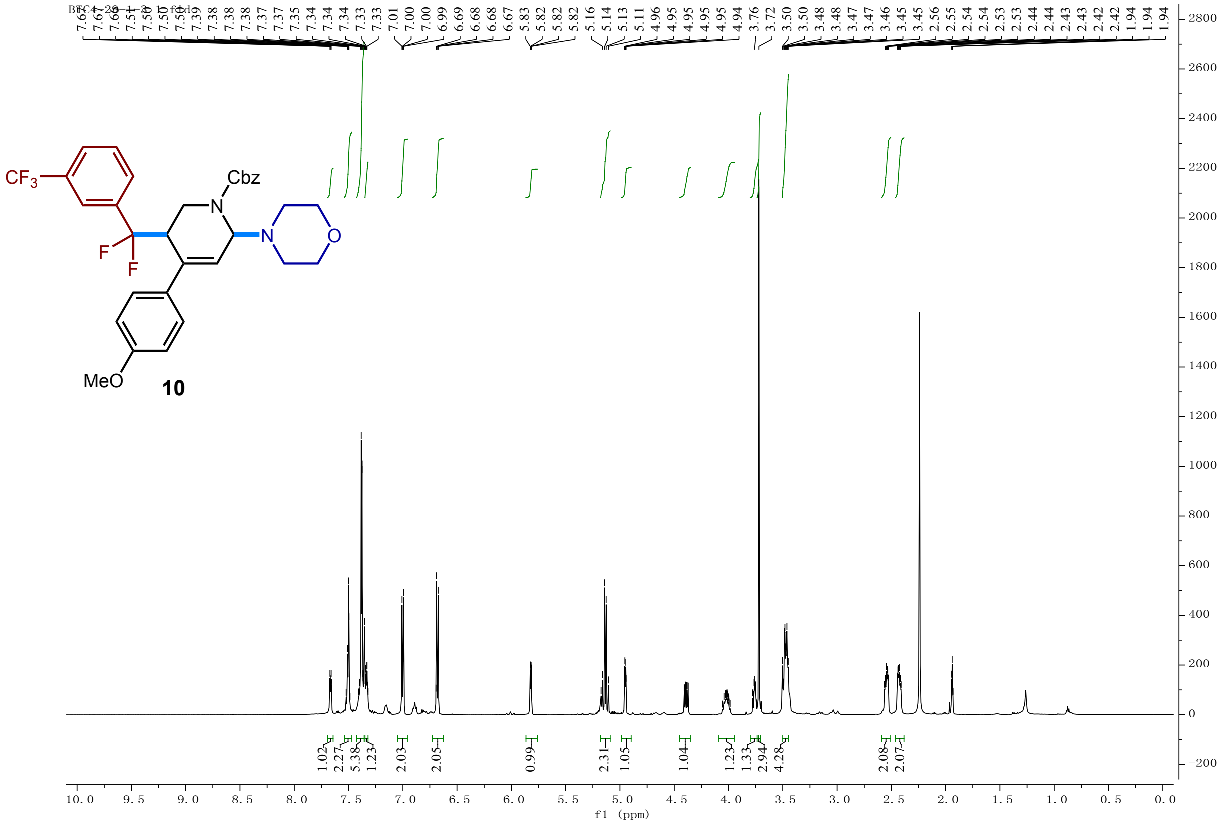
CF3
Cbz
N
N
O
F
F
MeO
10
f1 (ppm)

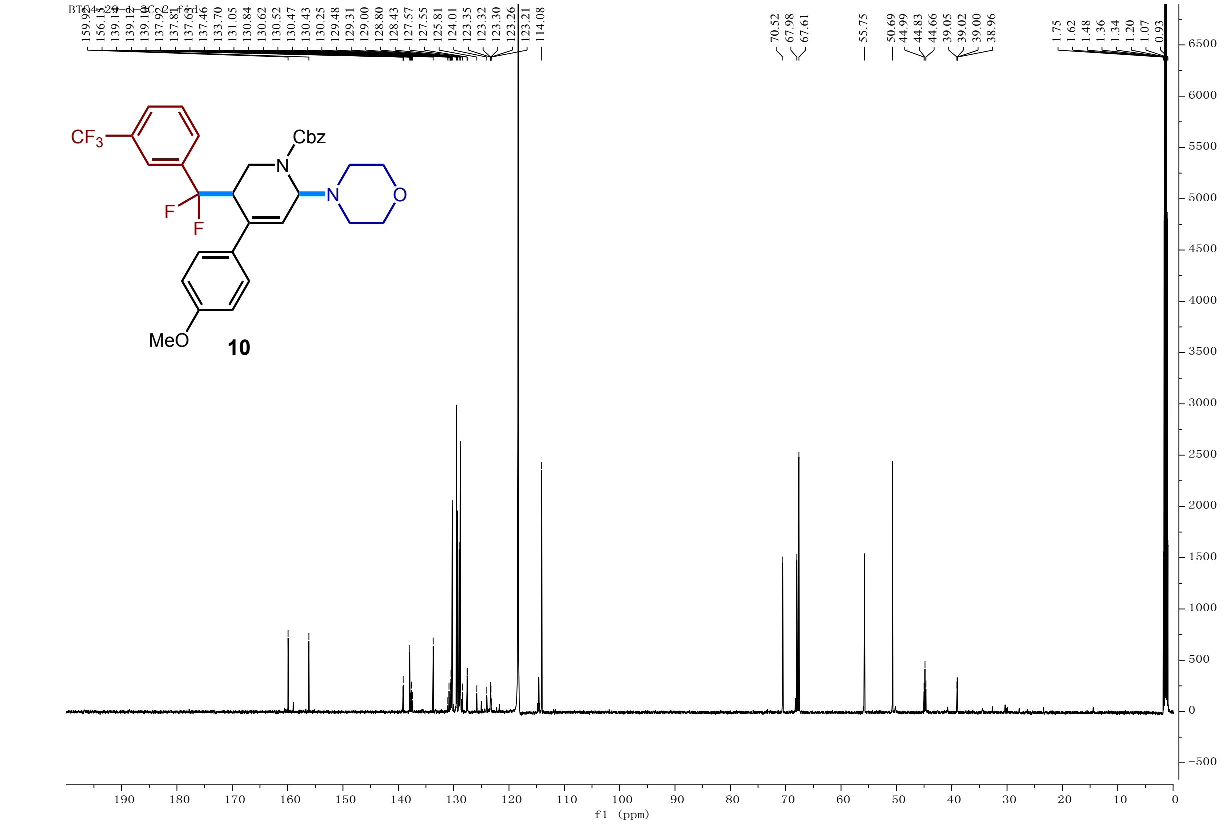
CF3
Cbz
N
N
O
F
F
MeO
10
f1 (ppm)

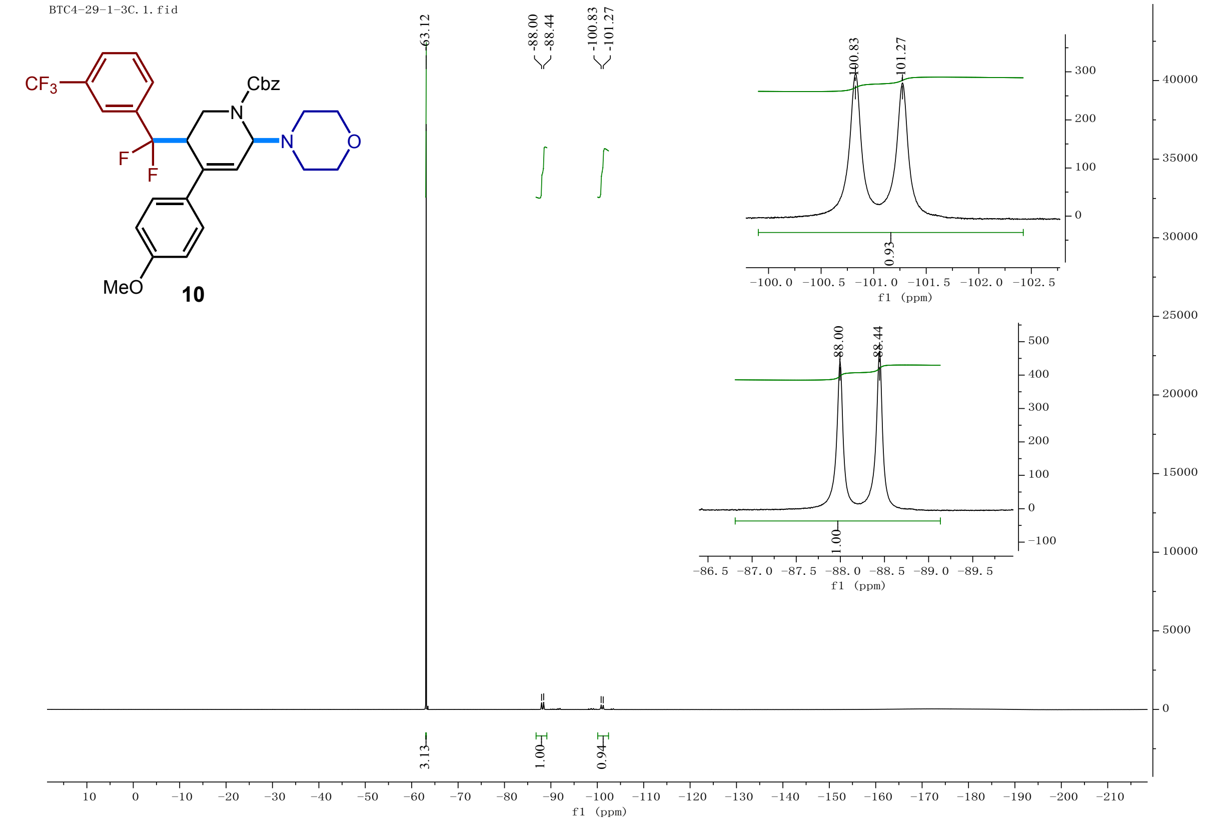
BTC4-29-1-3C.1.fid
10
Cbz
MeO
-63.12
-88.00
-88.44
-100.83
-101.27
f1 (ppm)

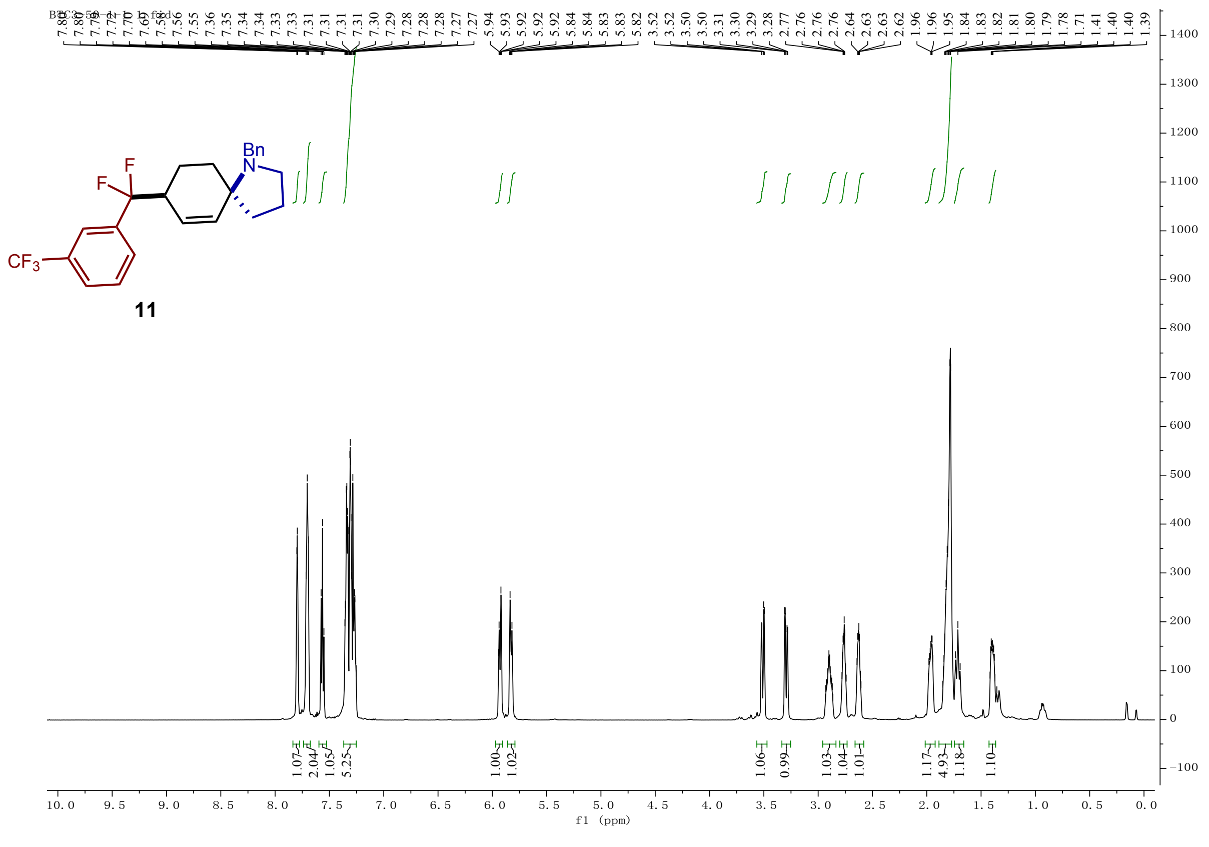
Bn
11
f1 (ppm)

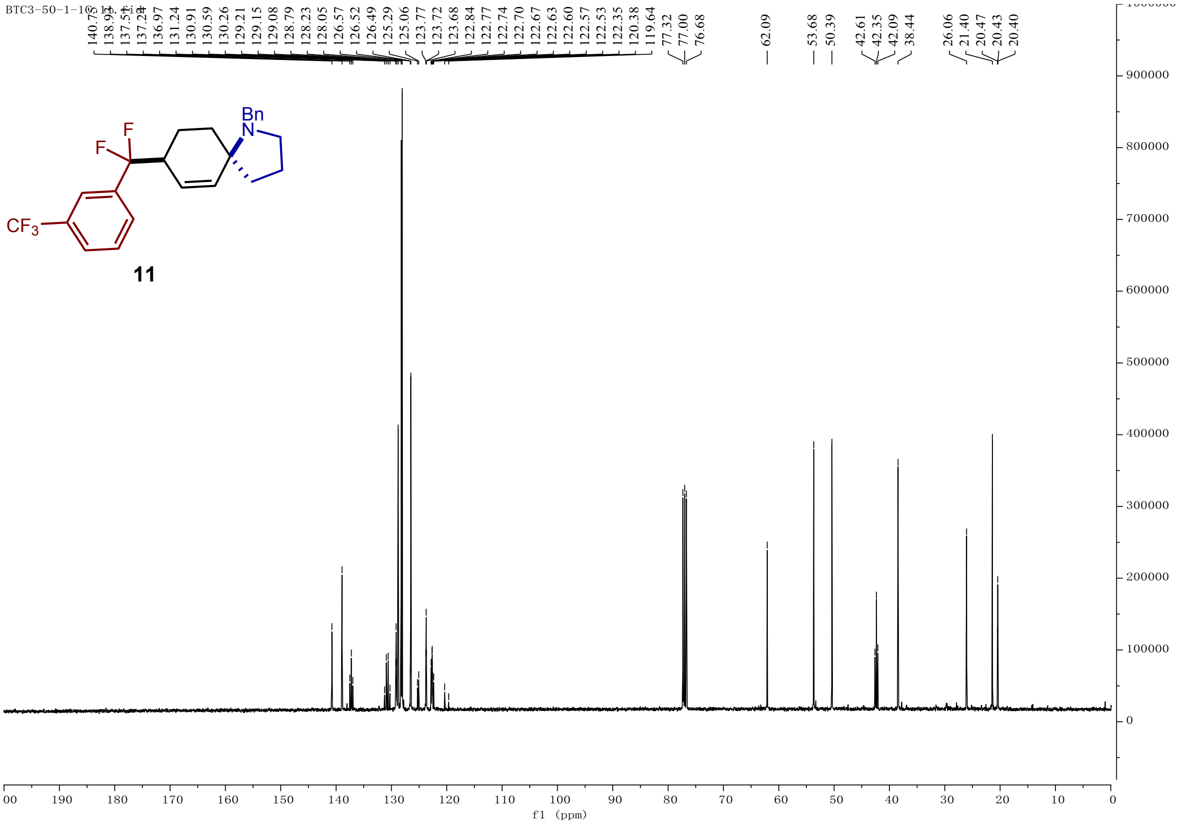
BTC3-50-1-1C
140.78 138.93 137.51 137.24 136.97 131.24 130.91 130.59 130.26 129.21 129.15 129.08 128.79 128.23 128.05 126.57 126.52 126.49 125.29 125.06 123.77 123.72 123.68 122.84 122.77 122.74 122.70 122.67 122.63 122.60 122.57 122.53 122.35 120.38 119.64 77.32 77.00 76.68 62.09 53.68 50.39 42.61 42.35 42.09 38.44 26.06 21.40 20.47 20.43 20.40
F
F
Bn
N
CF3
11
f1 (ppm)

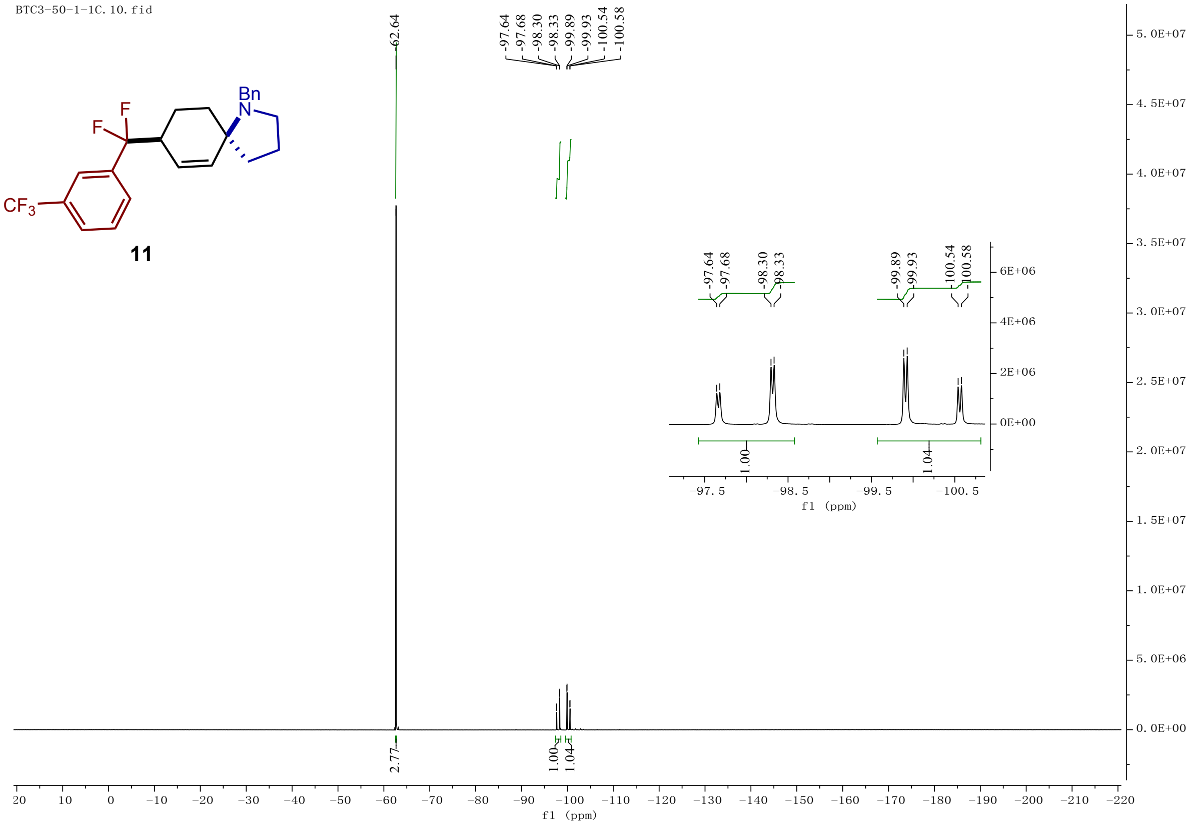
BTC3-50-1-1C.10.fid
-62.64
-97.64 -97.68 -98.30 -98.33 -99.89 -99.93 -100.54 -100.58
F
F
Bn
N
CF3
11
2.77
1.00
1.04
f1 (ppm)

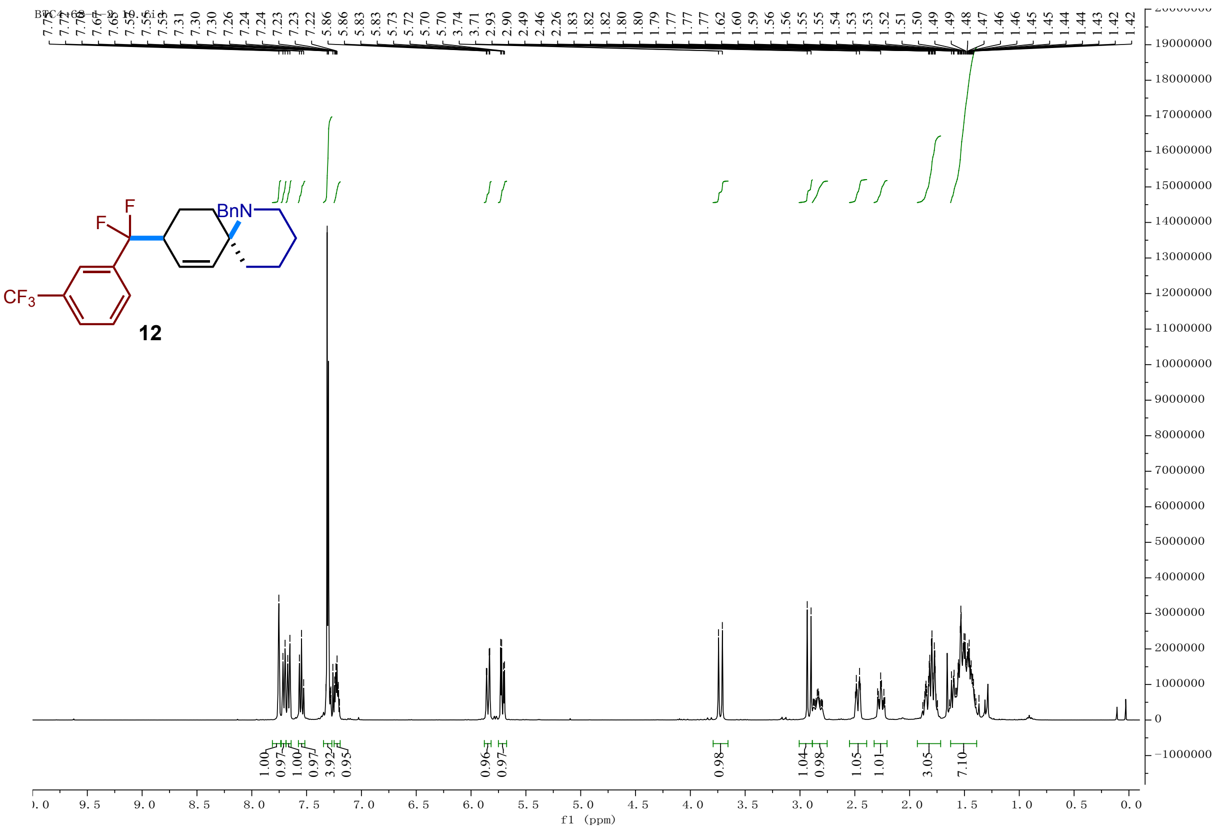
BTC4-68-1-2H.10.fid
f1 (ppm)
BnN
F
CF3
12

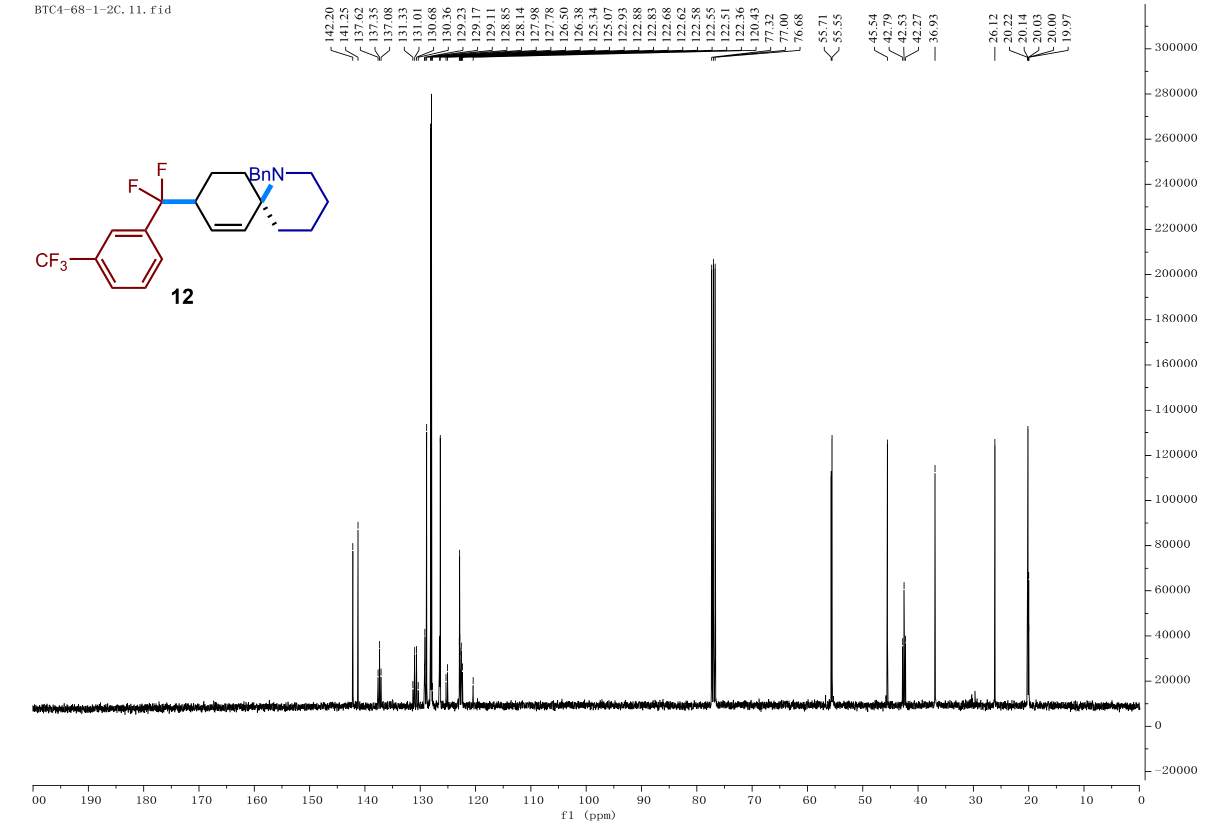
BTC4-68-1-2C.11.fid
f1 (ppm)
BnN
F
CF3
12

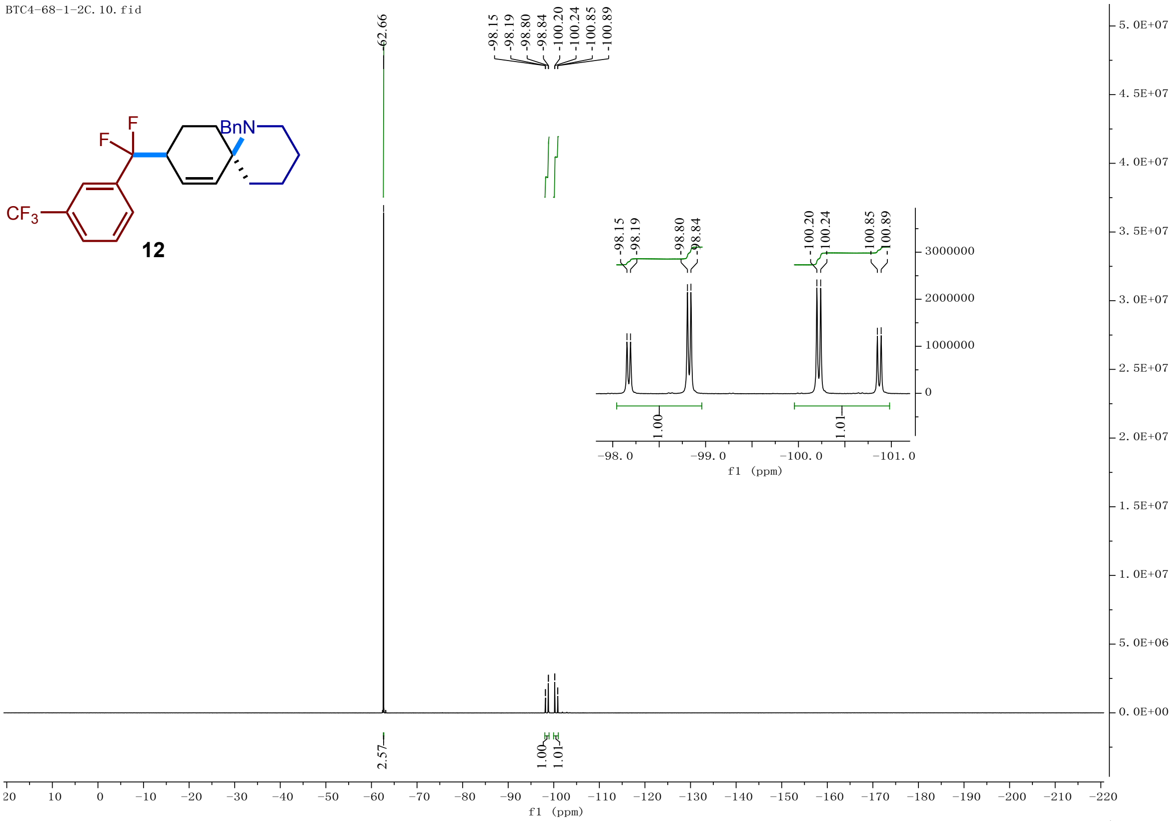
BTC4-68-1-2C.10.fid
-62.66
-98.15
-98.19
-98.80
-98.84
-100.20
-100.24
-100.85
-100.89
F
F
BnN
CF_3
12
2.57
1.00
1.01
f1 (ppm)

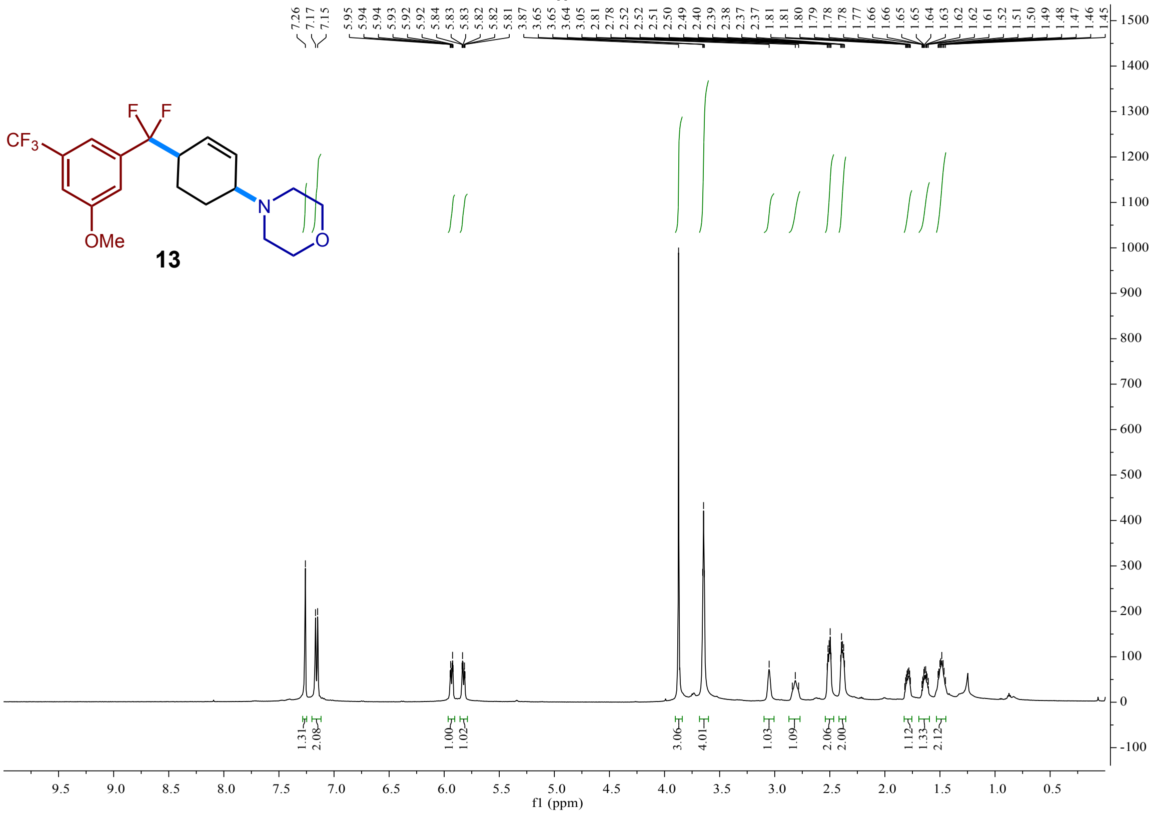
F
F
CF_3
N
O
OMe
13
3.06
4.01
f1 (ppm)

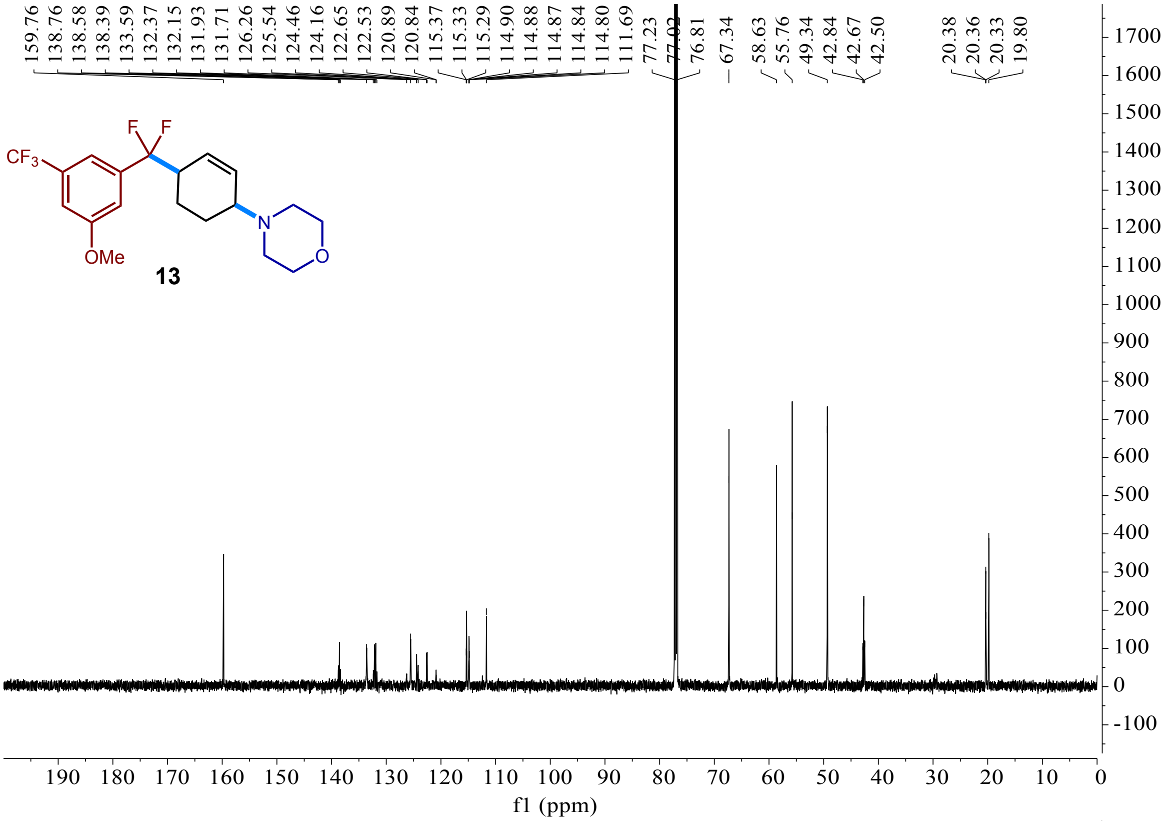

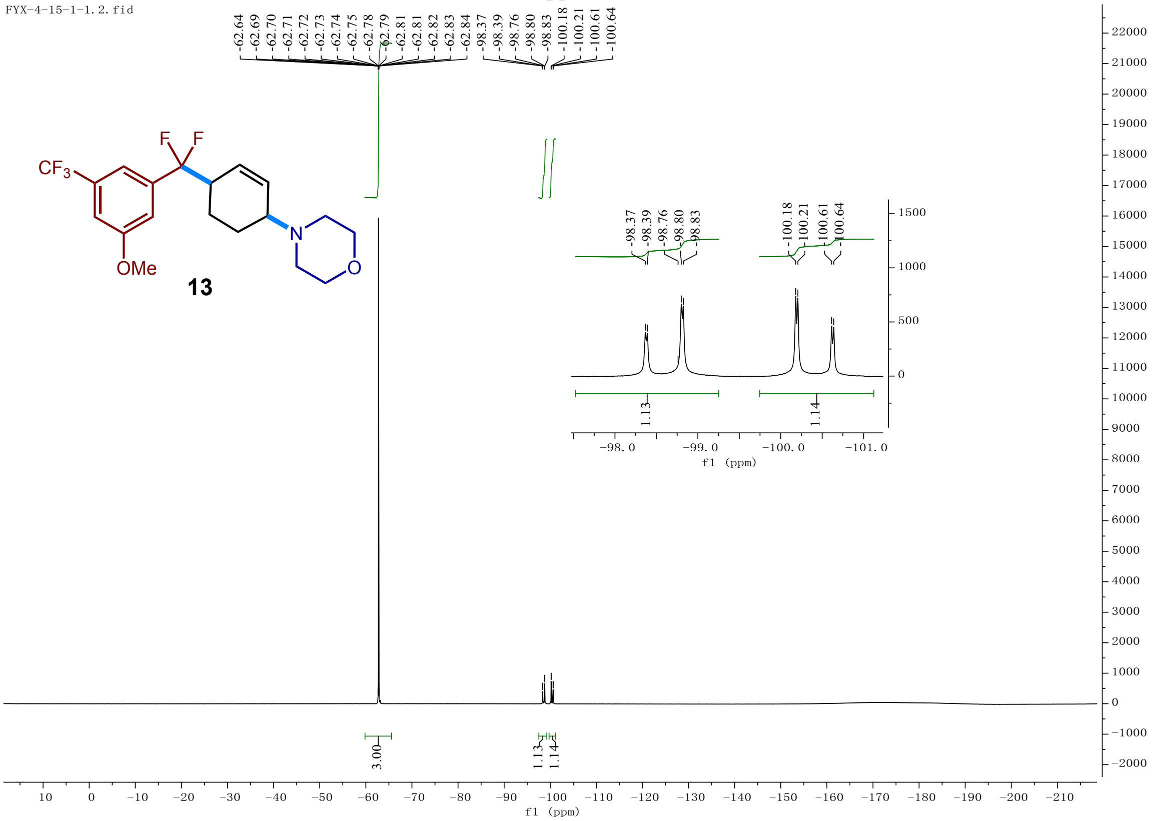

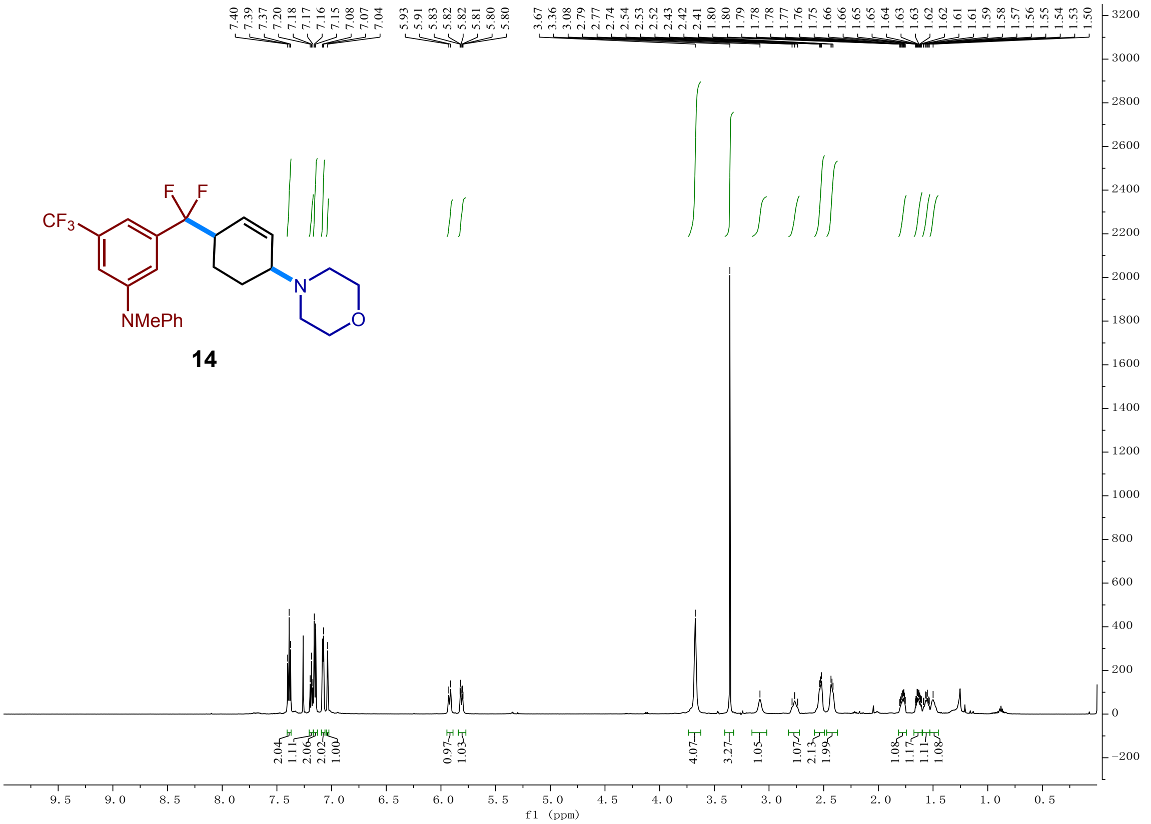
7.40 7.39 7.37 7.20 7.18 7.17 7.16 7.15 7.08 7.07 7.04
5.93 5.91 5.83 5.82 5.82 5.81 5.80 5.80
3.67 3.36 3.08 2.79 2.77 2.74 2.54 2.53 2.52 2.43 2.42 2.41 1.80 1.80 1.79 1.78 1.77 1.76 1.75 1.66 1.66 1.65 1.65 1.64 1.63 1.63 1.62 1.62 1.61 1.61 1.59 1.58 1.57 1.56 1.55 1.54 1.53 1.50
CF3
F
F
N
O
NMePh
14
2.04 1.11 2.06 2.02 1.00
0.97 1.03
4.07 3.27 1.05 1.07 2.13 1.99
1.08 1.17 1.11 1.08
f1 (ppm)

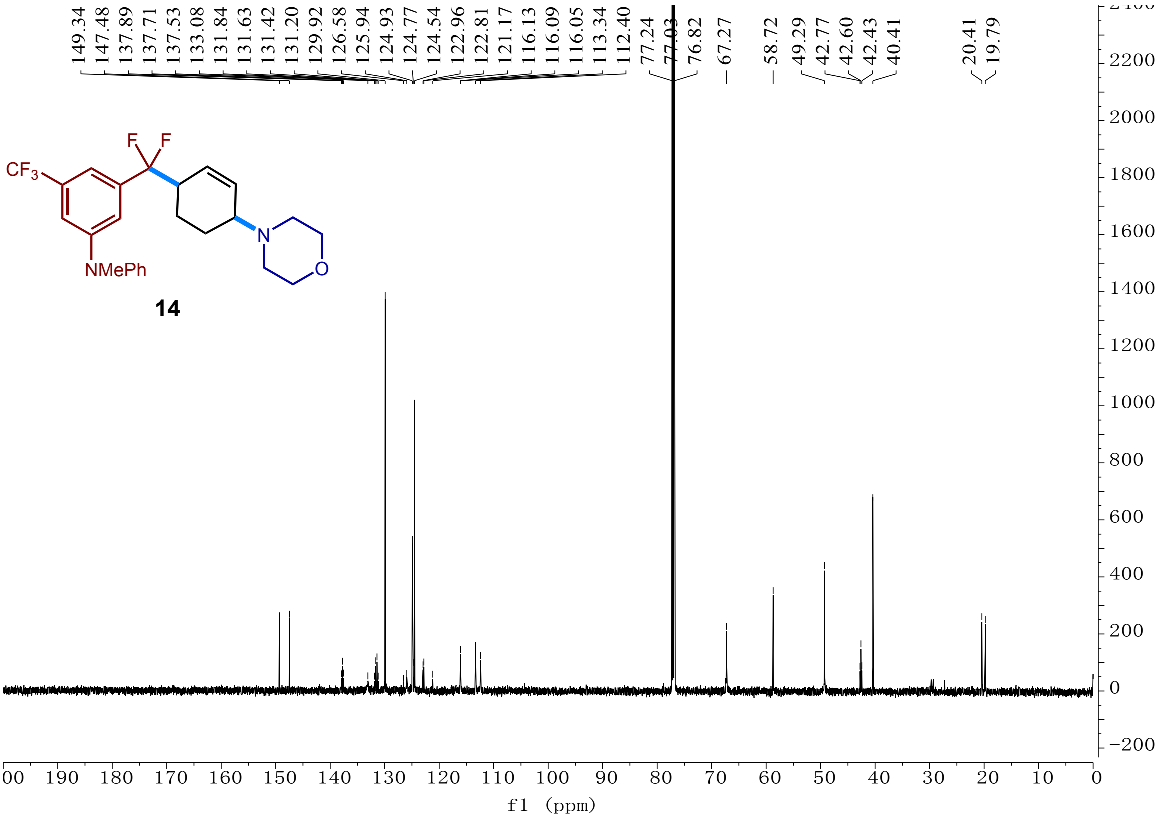
149.34 147.48 137.89 137.71 137.53 133.08 131.84 131.63 131.42 131.20 129.92 126.58 125.94 124.93 124.77 124.54 122.96 122.81 121.17 116.13 116.09 116.05 113.34 112.40 77.24 77.03 76.82 67.27 58.72 49.29 42.77 42.60 42.43 40.41 20.41 19.79
CF3
F
F
N
O
NMePh
14
f1 (ppm)

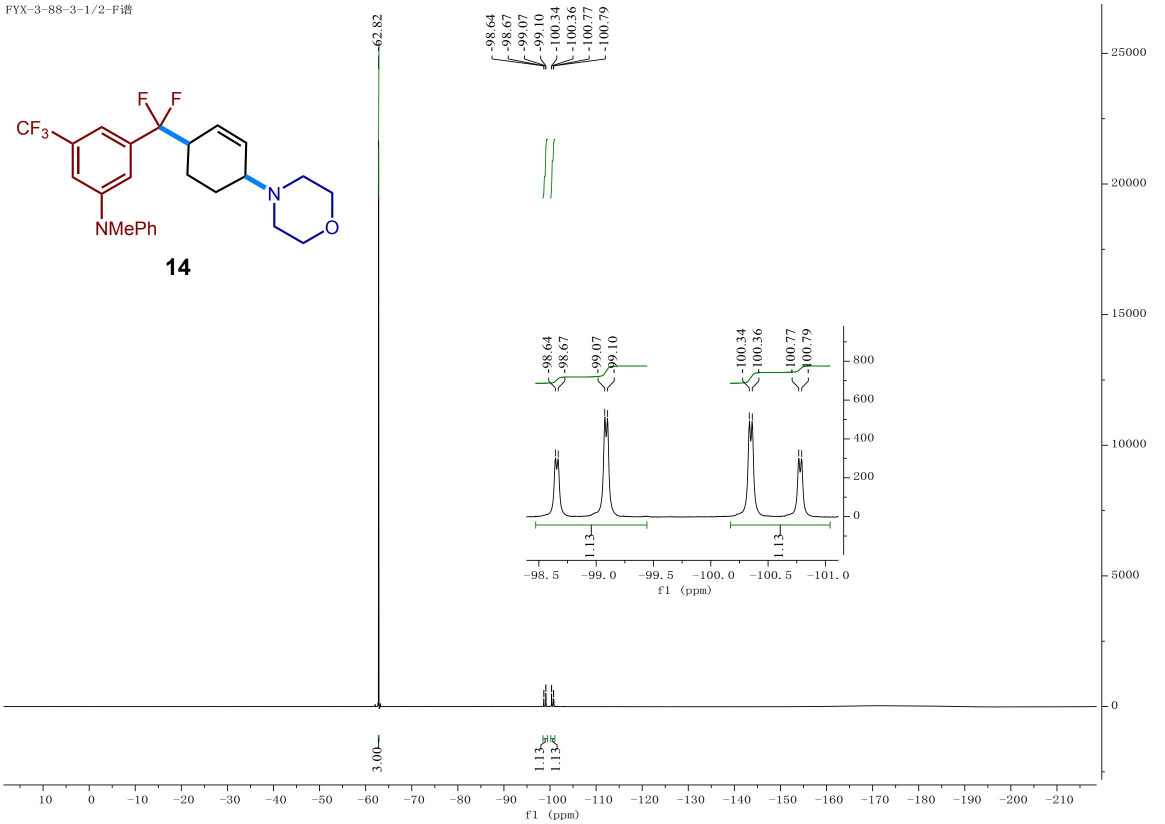
FYX-3-88-3-1/2-F谱
CF3
F F
N
O
NMePh
14
-62.82
-98.64
-98.67
-99.07
-99.10
-100.34
-100.36
-100.77
-100.79
3.00
1.13
1.13
f1 (ppm)

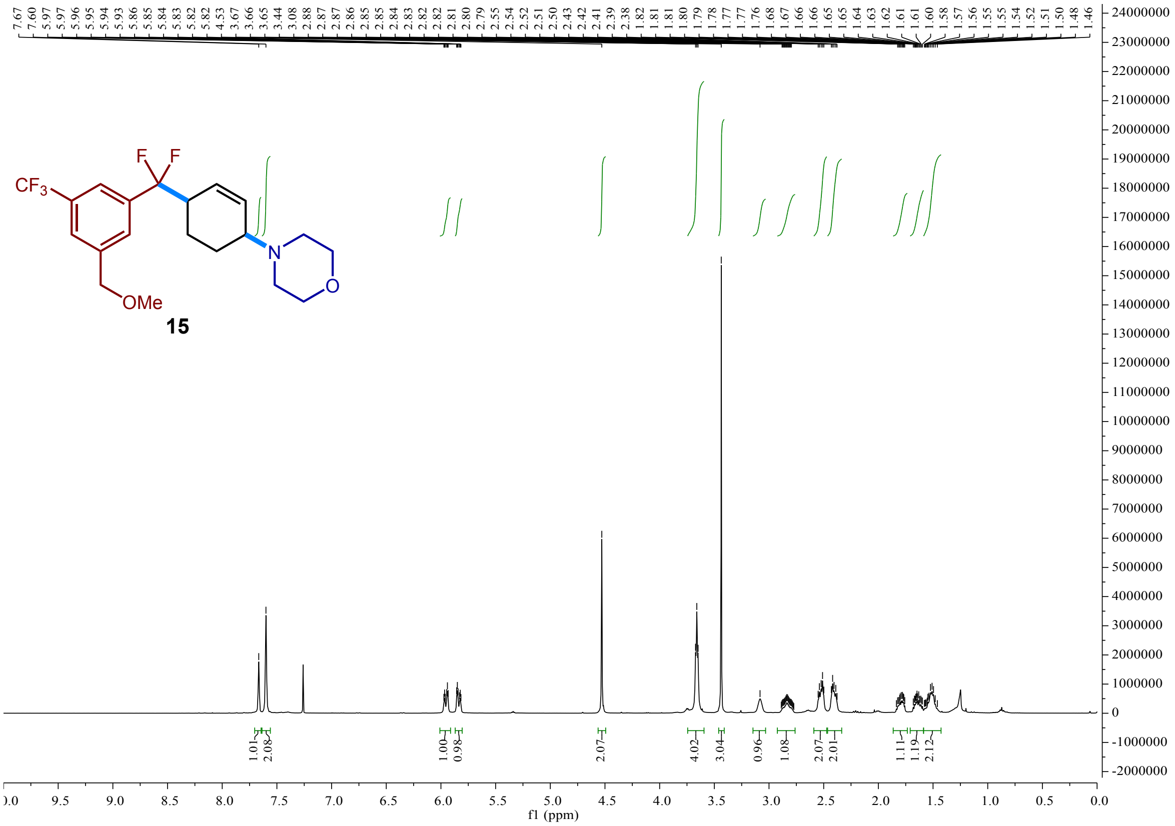
7.67
7.60
5.97
5.96
5.95
5.94
5.93
5.86
5.85
5.84
5.83
5.82
5.82
4.53
3.67
3.66
3.65
3.44
3.08
2.88
2.87
2.87
2.86
2.85
2.85
2.84
2.83
2.82
2.82
2.81
2.80
2.79
2.55
2.54
2.52
2.51
2.50
2.43
2.42
2.41
2.39
2.38
1.82
1.81
1.81
1.80
1.79
1.78
1.77
1.76
1.68
1.67
1.66
1.66
1.65
1.65
1.64
1.63
1.62
1.61
1.61
1.60
1.58
1.57
1.56
1.55
1.54
1.52
1.51
1.50
1.48
1.46
CF3
F F
N
O
OMe
15
1.01
2.08
1.00
0.98
2.07
4.02
3.04
0.96
1.08
2.07
2.01
1.11
1.19
2.12
f1 (ppm)

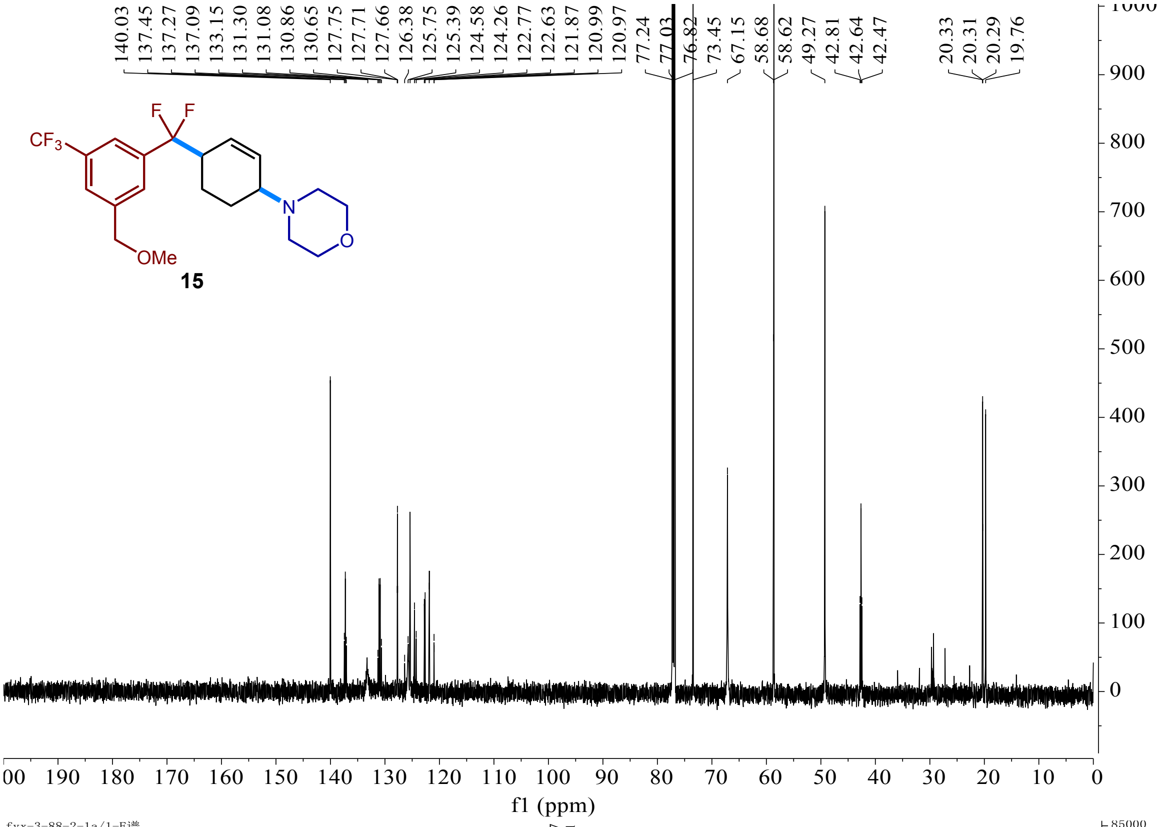
140.03
137.45
137.27
137.09
133.15
131.30
131.08
130.86
130.65
127.75
127.71
127.66
126.38
125.75
125.39
124.58
124.26
122.77
122.63
121.87
120.99
120.97
77.24
77.03
76.82
73.45
67.15
58.68
58.62
49.27
42.81
42.64
42.47
20.33
20.31
20.29
19.76
CF3
F
F
N
O
OMe
15
f1 (ppm)

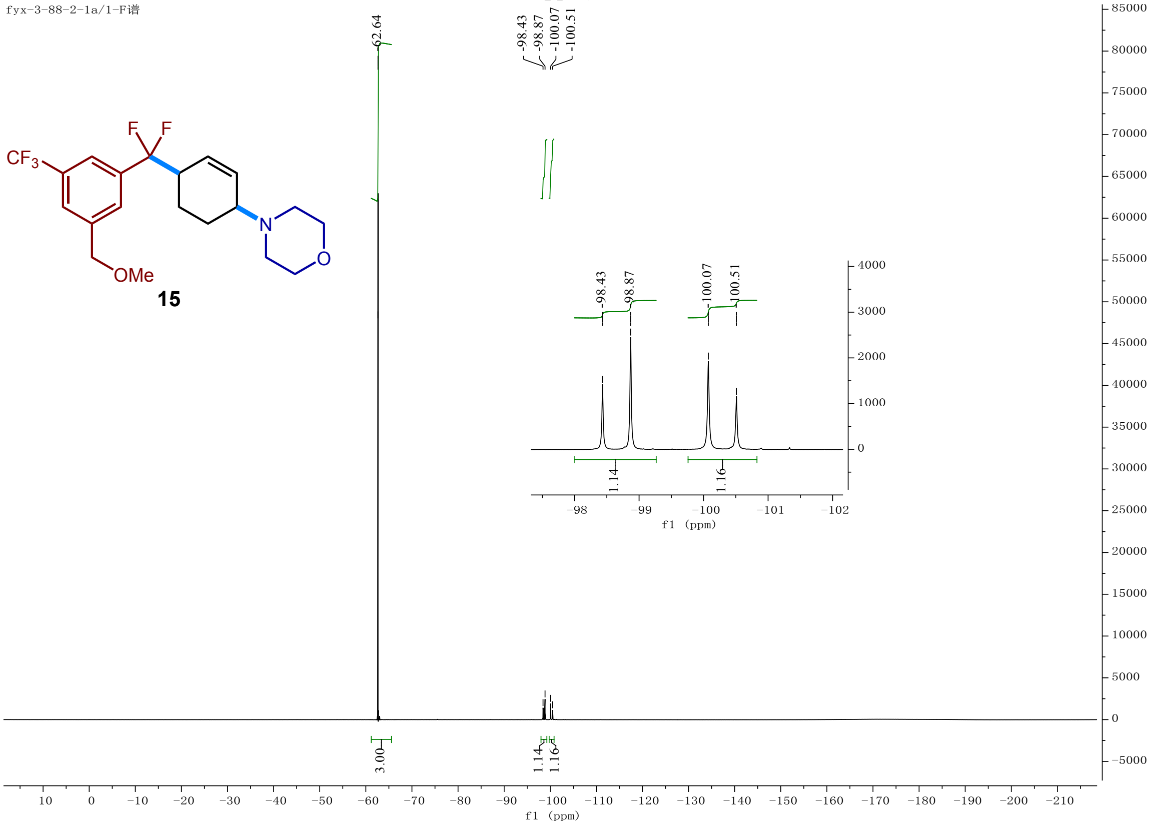
fyx-3-88-2-1a/1-F谱
-62.64
-98.43
-98.87
-100.07
-100.51
CF3
F
F
N
O
OMe
15
1.14
1.16
3.00
f1 (ppm)

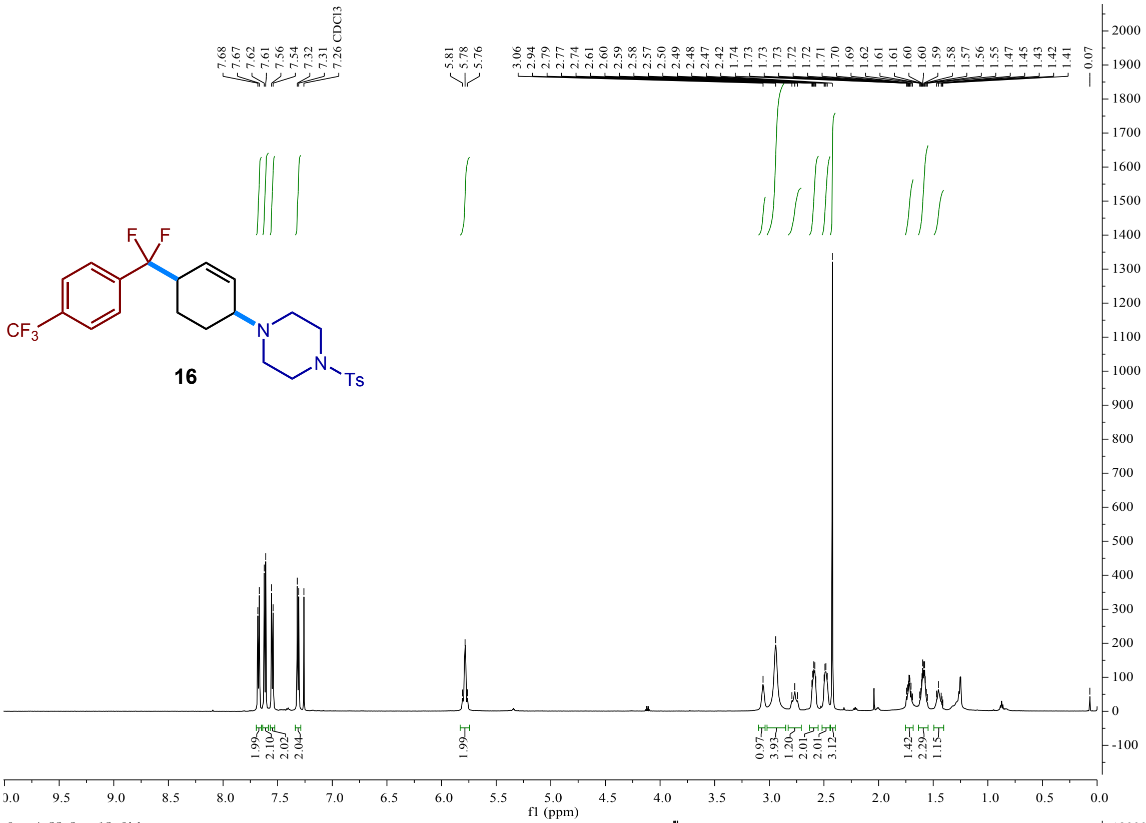
16
CF3
Ts
f1 (ppm)

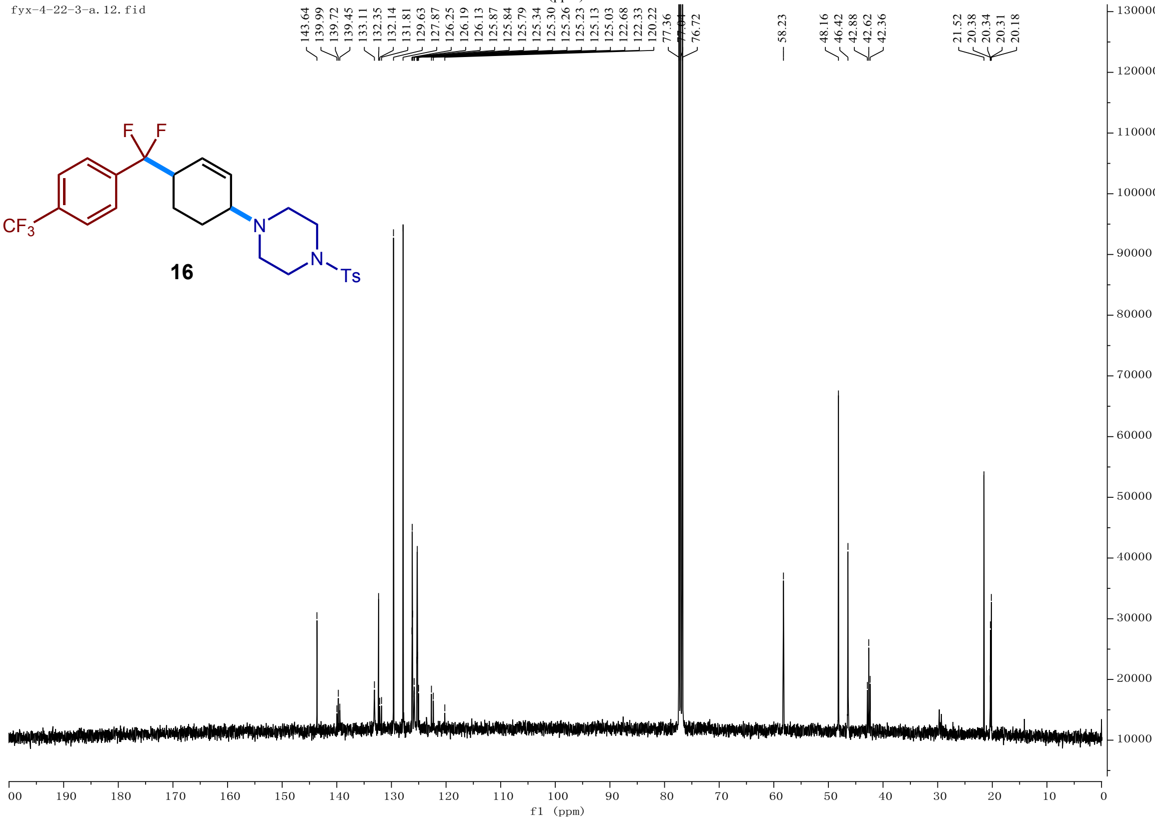
fyx-4-22-3-a.12.fid
16
CF3
Ts
f1 (ppm)

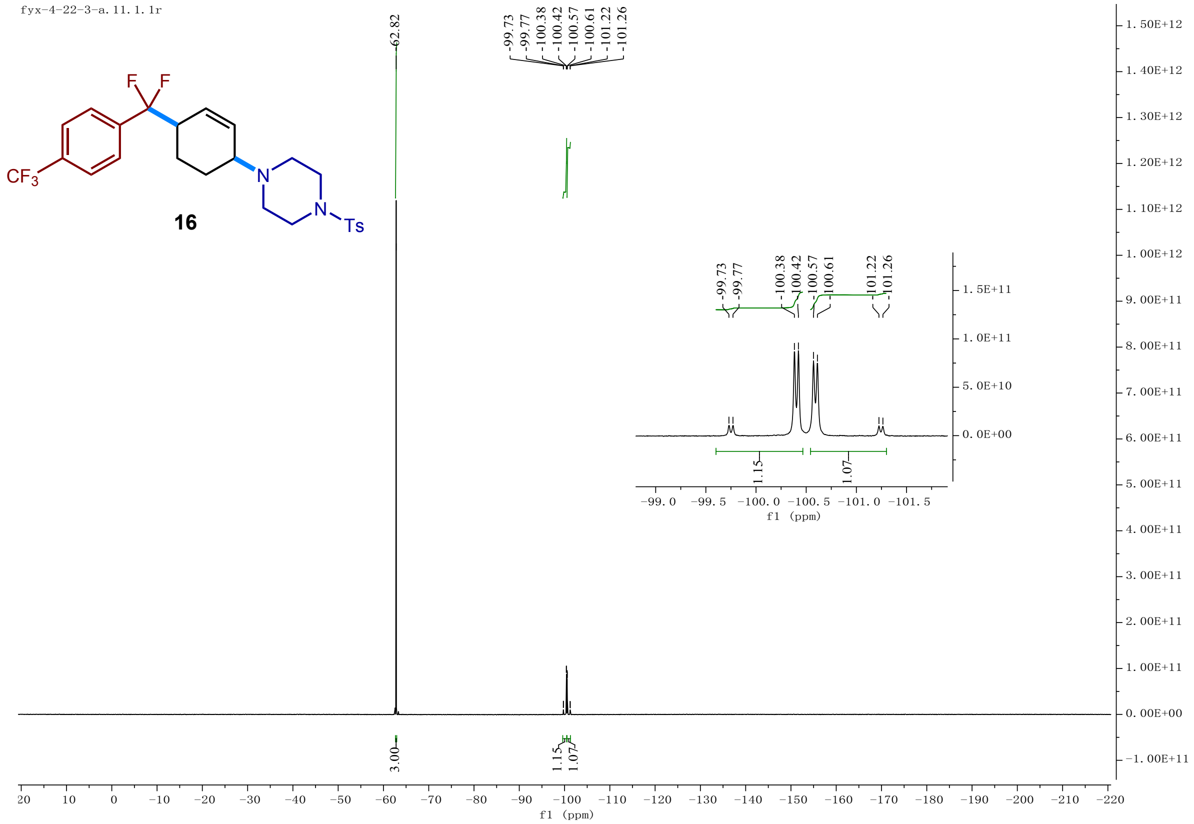
fyx-4-22-3-a.11.1.1r
F
F
CF3
N
N
Ts
16
-62.82
-99.73
-99.77
-100.38
-100.42
-100.57
-100.61
-101.22
-101.26
3.00
1.15
1.07
f1 (ppm)

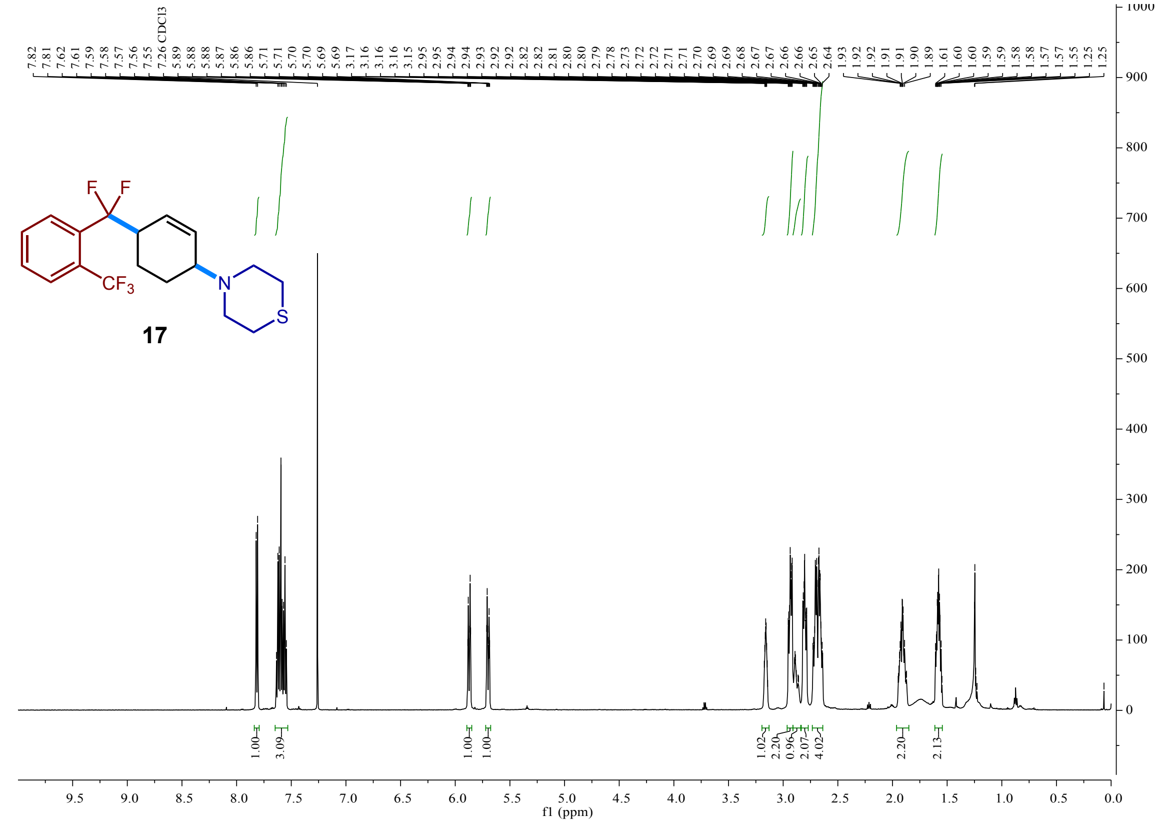
F
F
CF3
N
S
17
7.26 CDCl3
1.00
3.09
1.00
1.00
1.02
2.20
0.96
2.07
4.02
2.20
2.13
f1 (ppm)

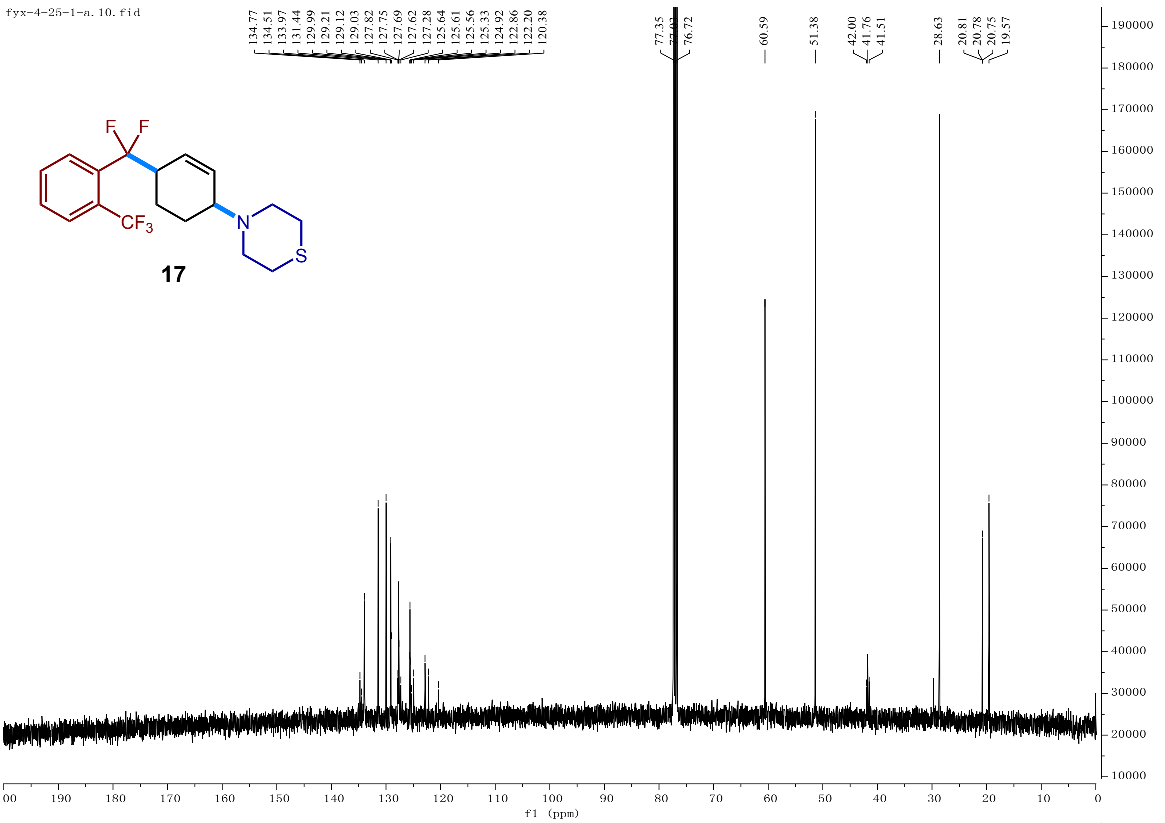
fyx-4-25-1-a.10.fid
134.77
134.51
133.97
131.44
129.99
129.21
129.12
129.03
127.82
127.75
127.69
127.62
127.28
125.64
125.61
125.56
125.33
124.92
122.86
122.20
120.38
77.35
76.72
60.59
51.38
42.00
41.76
41.51
28.63
20.81
20.78
20.75
19.57
17
f1 (ppm)

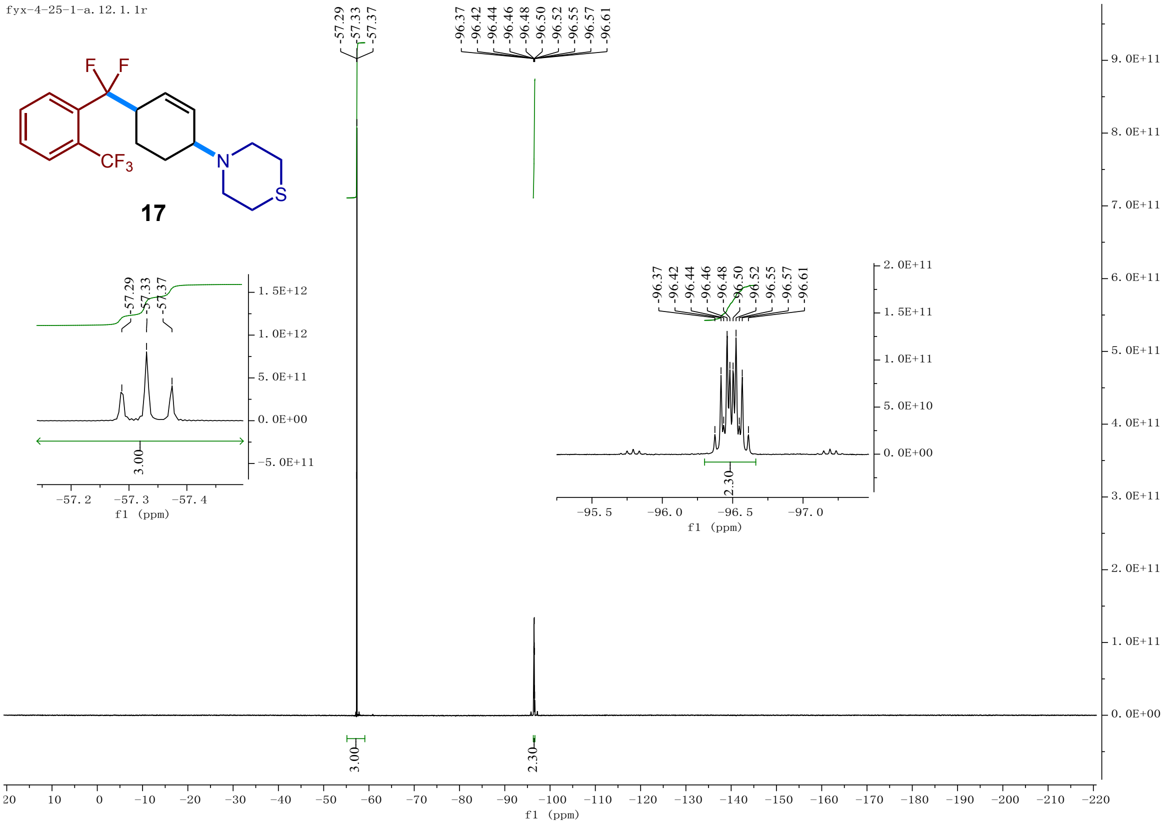
fyx-4-25-1-a.12.1.1r
-57.29
-57.33
-57.37
-96.37
-96.42
-96.44
-96.46
-96.48
-96.50
-96.52
-96.55
-96.57
-96.61
17
3.00
2.30
f1 (ppm)

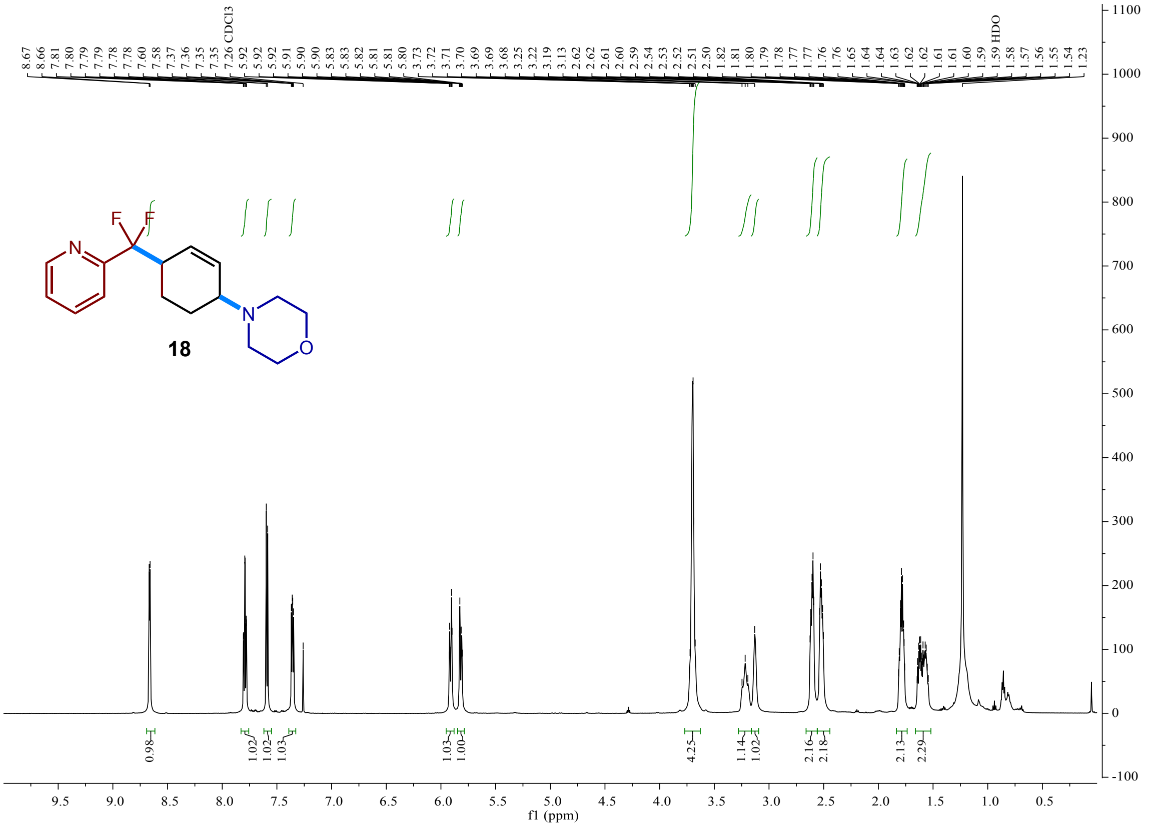

18

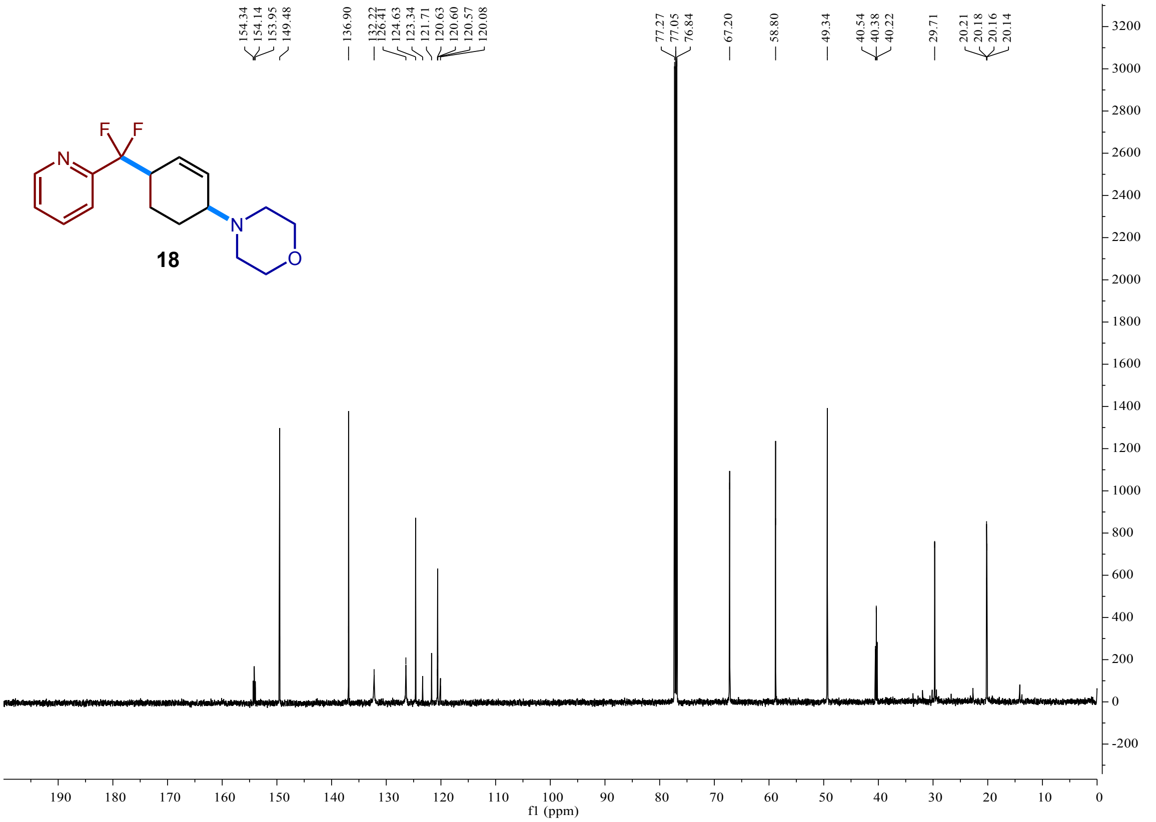

18

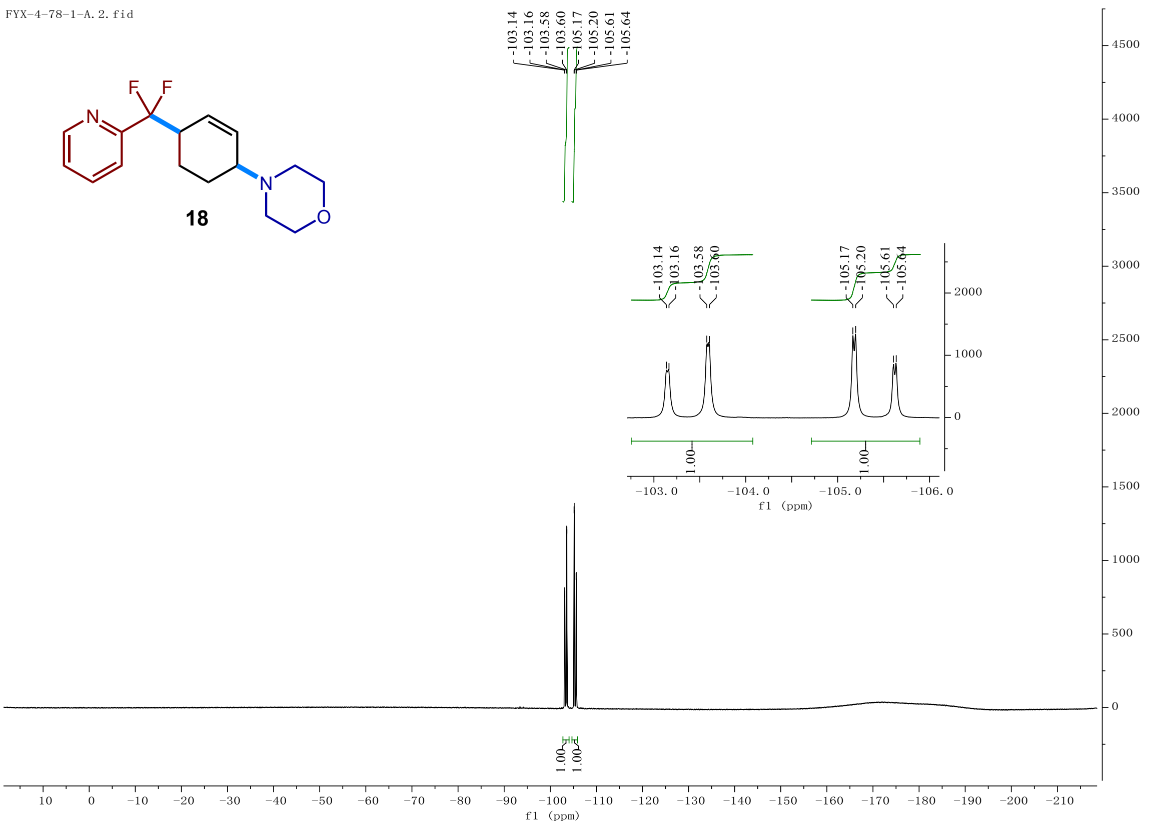
FYX-4-78-1-A.2.fid
18
-103.14
-103.16
-103.58
-103.60
-105.17
-105.20
-105.61
-105.64
1.00
1.00
f1 (ppm)

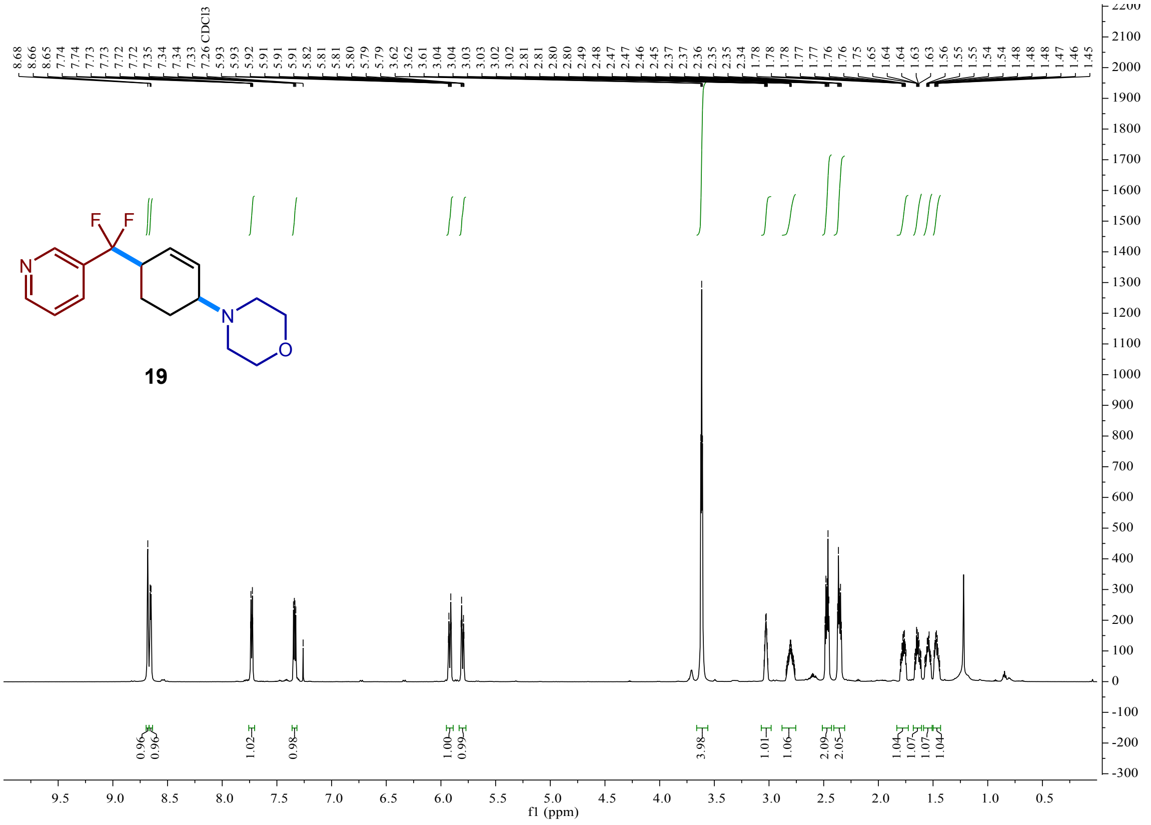
19
8.68 8.66 8.65 7.74 7.74 7.73 7.73 7.72 7.72 7.35 7.34 7.34 7.33 7.26 CDCl3 5.93 5.93 5.92 5.91 5.91 5.91 5.82 5.81 5.81 5.80 5.79 5.79 3.62 3.62 3.61 3.04 3.04 3.03 3.03 3.02 3.02 2.81 2.81 2.80 2.80 2.49 2.48 2.47 2.47 2.46 2.45 2.37 2.37 2.36 2.35 2.35 2.34 1.78 1.78 1.78 1.77 1.77 1.76 1.76 1.75 1.65 1.64 1.64 1.63 1.63 1.56 1.55 1.55 1.54 1.54 1.48 1.48 1.48 1.47 1.46 1.45
0.96 0.96 1.02 0.98 1.00 0.99 3.98 1.01 1.06 2.09 2.05 1.04 1.07 1.07 1.04
f1 (ppm)

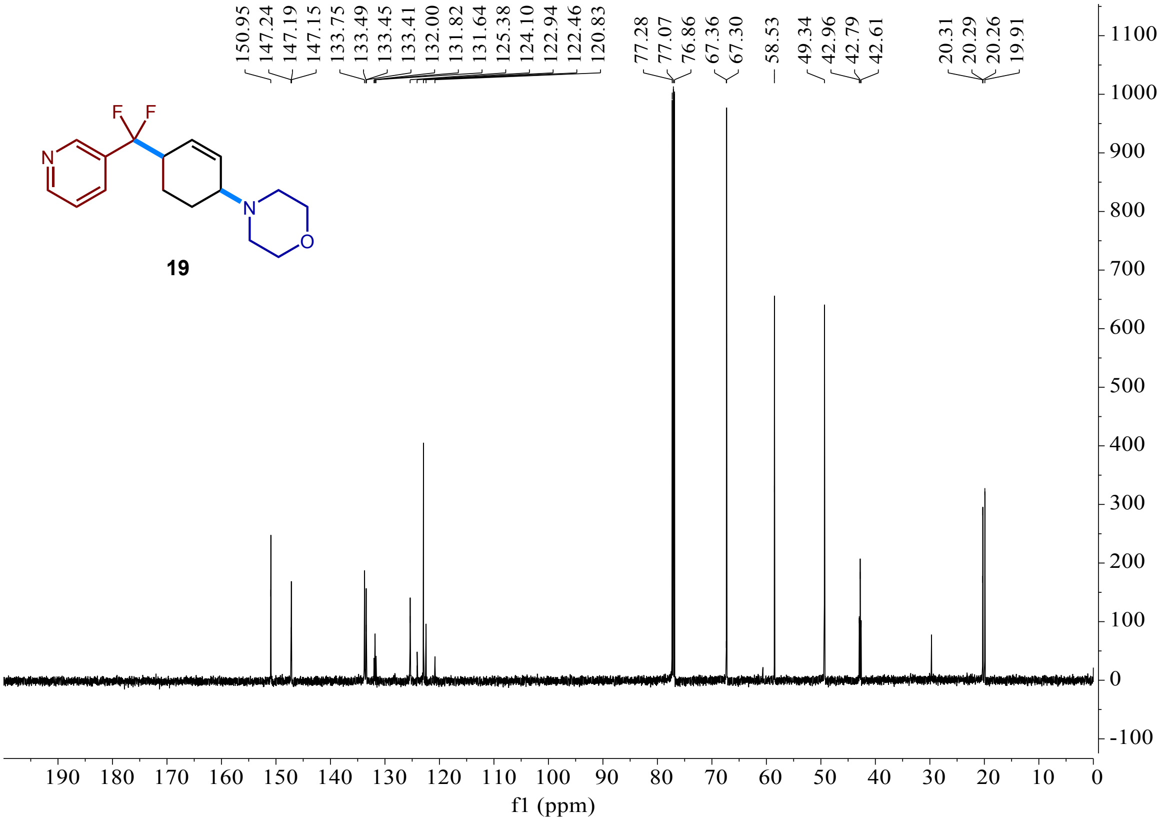
150.95
147.24
147.19
147.15
133.75
133.49
133.45
133.41
132.00
131.82
131.64
125.38
124.10
122.94
122.46
120.83
77.28
77.07
76.86
67.36
67.30
58.53
49.34
42.96
42.79
42.61
20.31
20.29
20.26
19.91
F F
N
N
O
19
f1 (ppm)

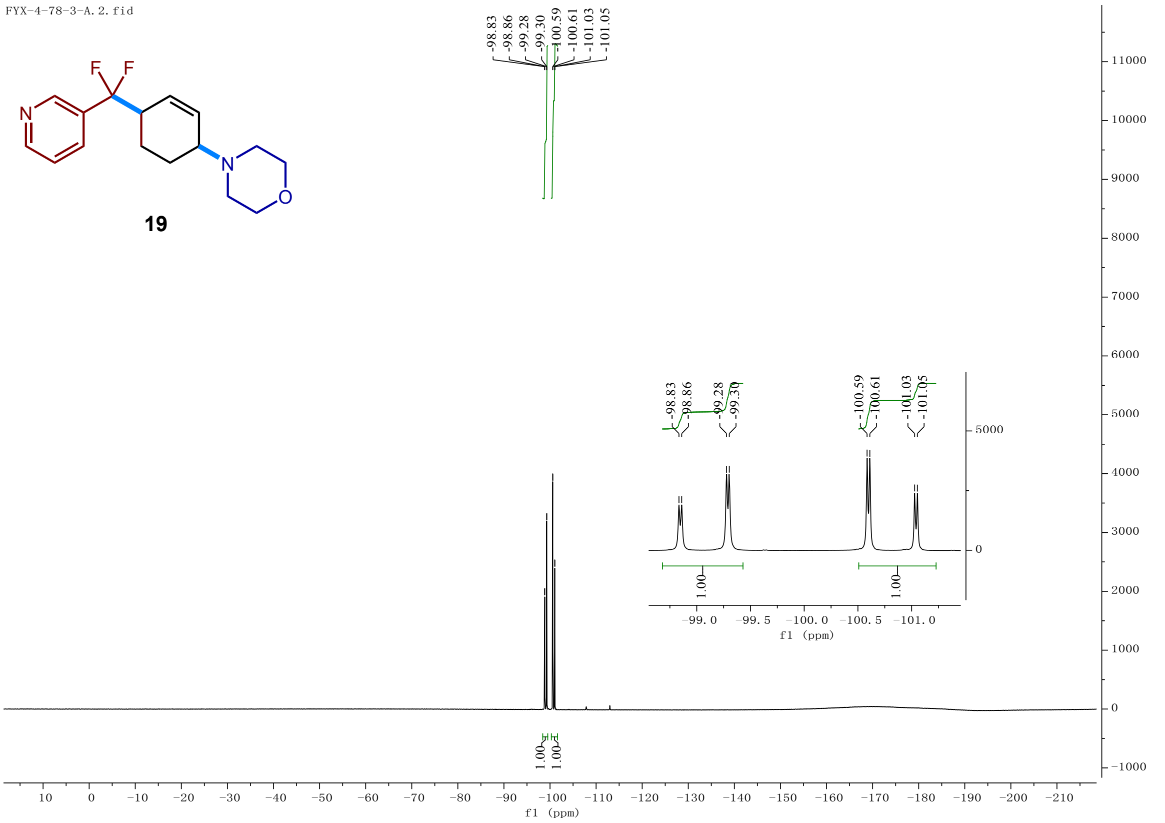
FYX-4-78-3-A.2.fid
-98.83
-98.86
-99.28
-99.30
-100.59
-100.61
-101.03
-101.05
F F
N
N
O
19
1.00
1.00
f1 (ppm)

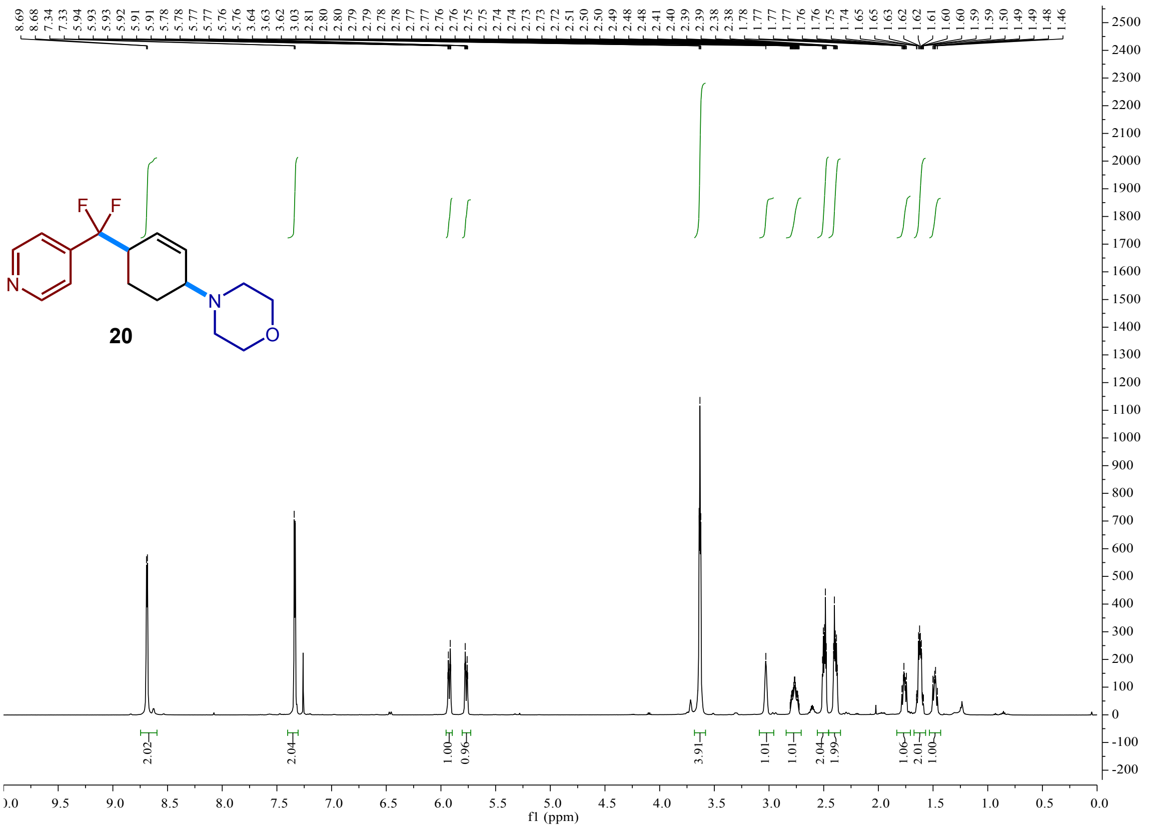
8.69 8.68 7.34 7.33 5.94 5.93 5.93 5.92 5.91 5.91 5.78 5.78 5.77 5.77 5.76 5.76 3.64 3.63 3.62 3.03 2.81 2.80 2.80 2.79 2.79 2.78 2.78 2.77 2.77 2.76 2.76 2.75 2.75 2.74 2.74 2.73 2.73 2.72 2.51 2.50 2.50 2.49 2.49 2.48 2.48 2.41 2.40 2.39 2.39 2.38 2.38 1.78 1.77 1.77 1.77 1.76 1.76 1.75 1.74 1.65 1.65 1.63 1.62 1.62 1.61 1.60 1.60 1.59 1.59 1.50 1.49 1.49 1.48 1.46
F
F
N
N
O
20
2.02
2.04
1.00
0.96
3.91
1.01
1.01
2.04
1.99
1.06
2.01
1.00
f1 (ppm)

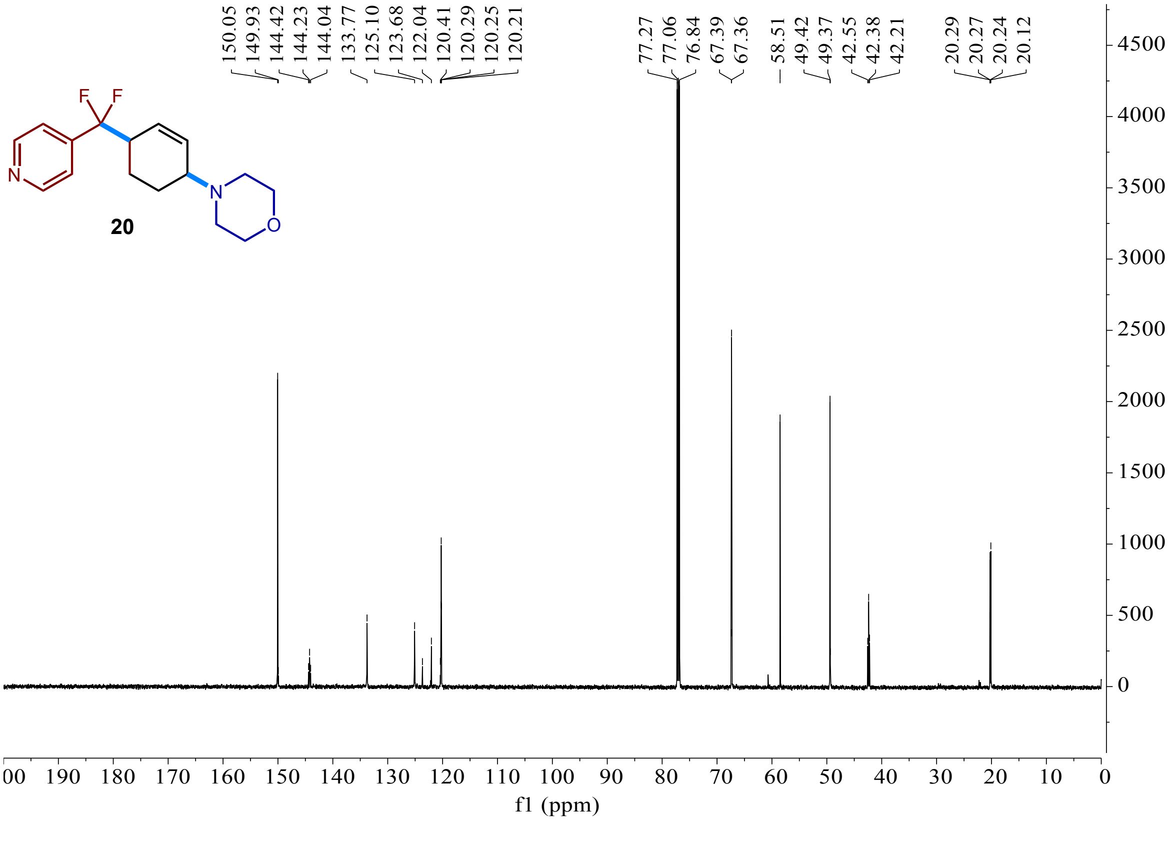
150.05 149.93 144.42 144.23 144.04 133.77 125.10 123.68 122.04 120.41 120.29 120.25 120.21
77.27 77.06 76.84 67.39 67.36 58.51 49.42 49.37 42.55 42.38 42.21
20.29 20.27 20.24 20.12
F
F
N
N
O
20
f1 (ppm)

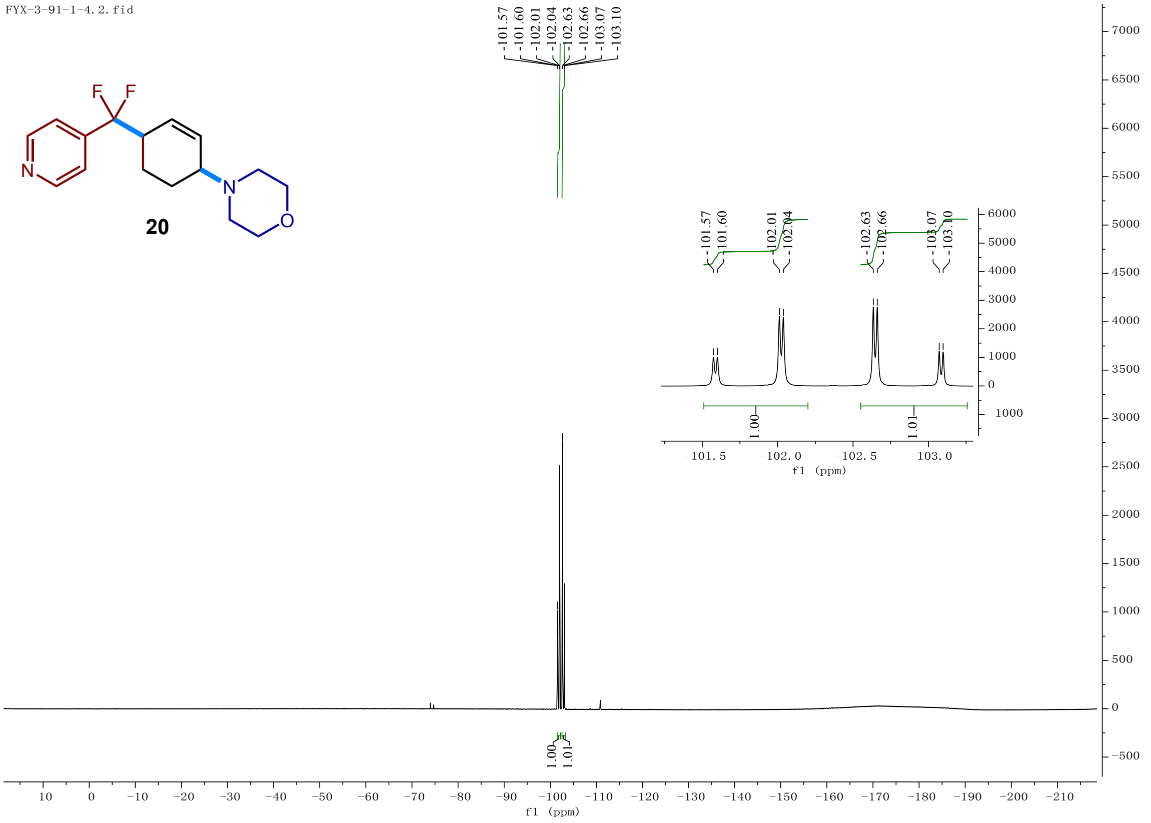
FYX-3-91-1-4.2.fid
-101.57
-101.60
-102.01
-102.04
-102.63
-102.66
-103.07
-103.10
F F
N
O
20
1.00
1.01
f1 (ppm)

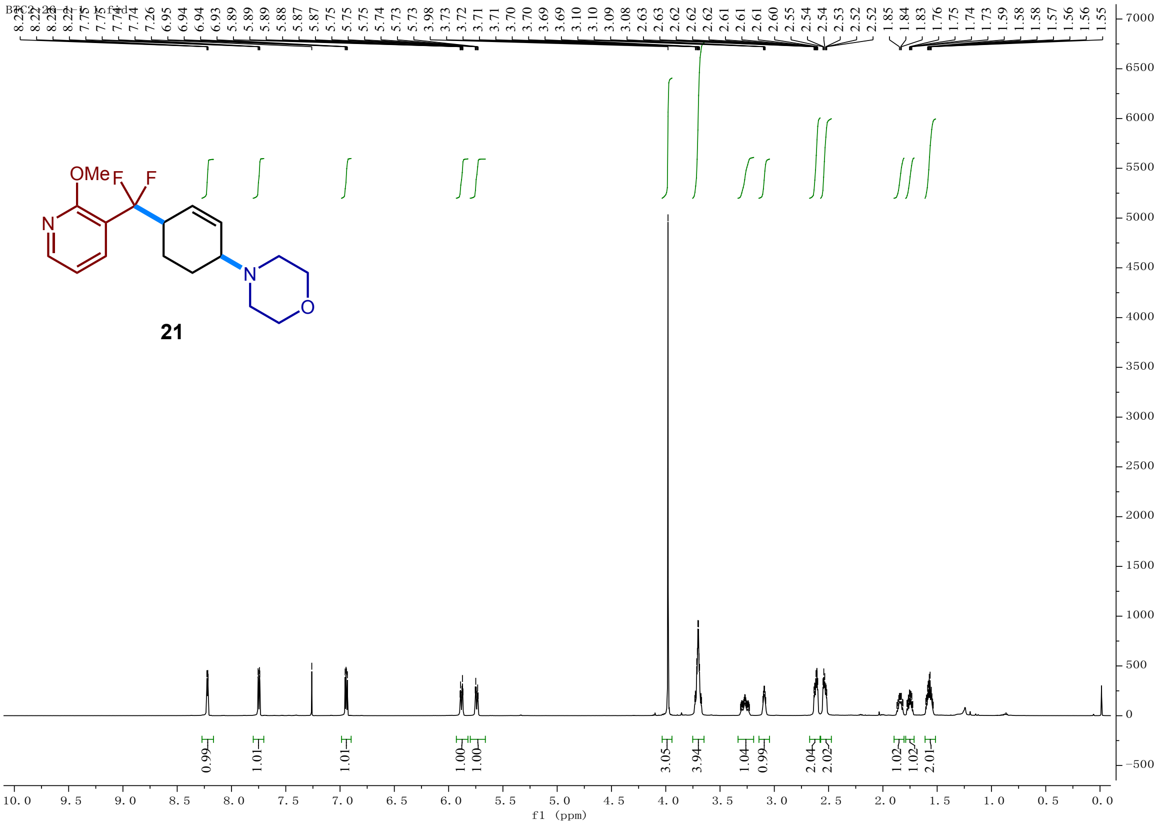
fid
8.22 8.22 8.22 7.75 7.75 7.74 7.74 7.26 6.95 6.94 6.94 6.93 5.89 5.89 5.88 5.87 5.87 5.75 5.75 5.74 5.73 5.73 3.98 3.73 3.72 3.71 3.71 3.70 3.70 3.69 3.69 3.10 3.10 3.09 3.08 2.63 2.63 2.62 2.62 2.62 2.61 2.61 2.61 2.60 2.55 2.54 2.54 2.53 2.52 2.52 1.85 1.84 1.83 1.76 1.75 1.74 1.73 1.59 1.58 1.58 1.57 1.56 1.56 1.55
OMe F F
N
O
21
0.99 1.01 1.01 1.00 1.00 3.05 3.94 1.04 0.99 2.04 2.02 1.02 1.02 2.01
f1 (ppm)

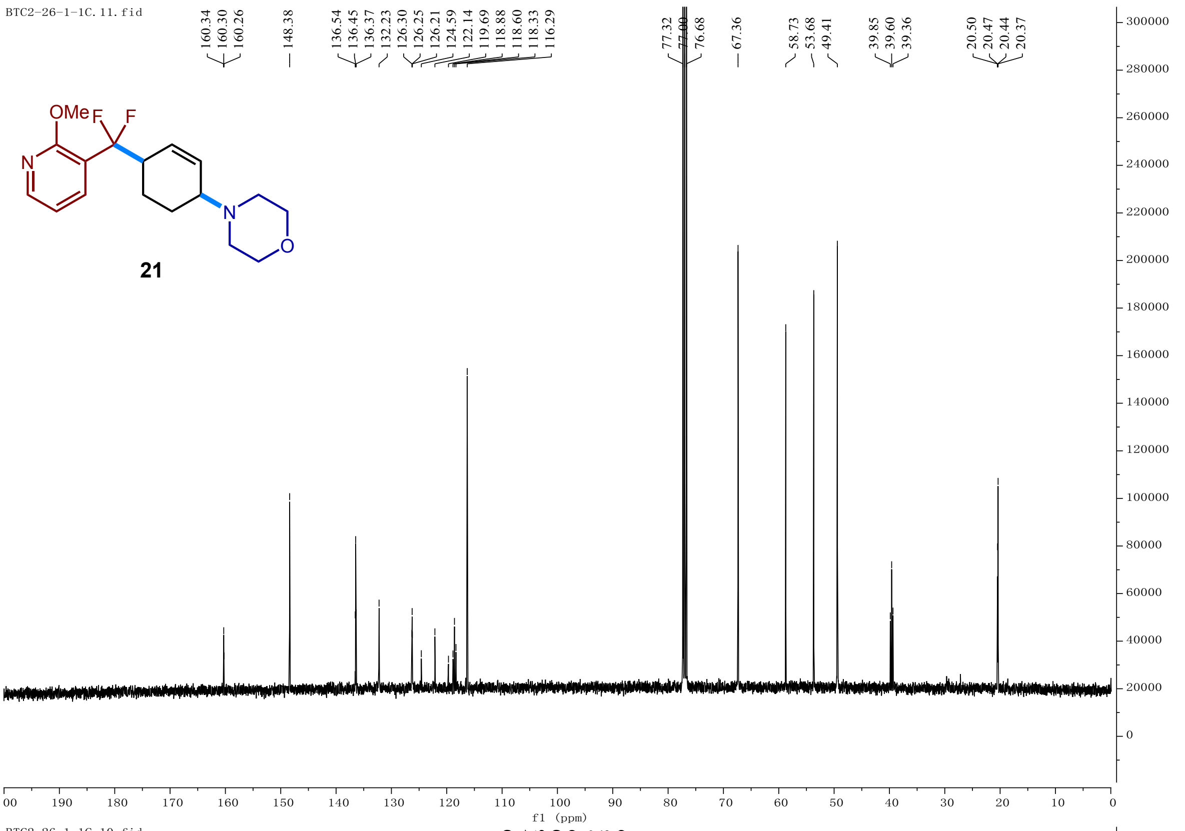
BTC2-26-1-1C.11.fid
160.34
160.30
160.26
148.38
136.54
136.45
136.37
132.23
126.30
126.25
126.21
124.59
122.14
119.69
118.88
118.60
118.33
116.29
77.32
77.00
76.68
67.36
58.73
53.68
49.41
39.85
39.60
39.36
20.50
20.47
20.44
20.37
OMe
F
F
N
O
21
f1 (ppm)

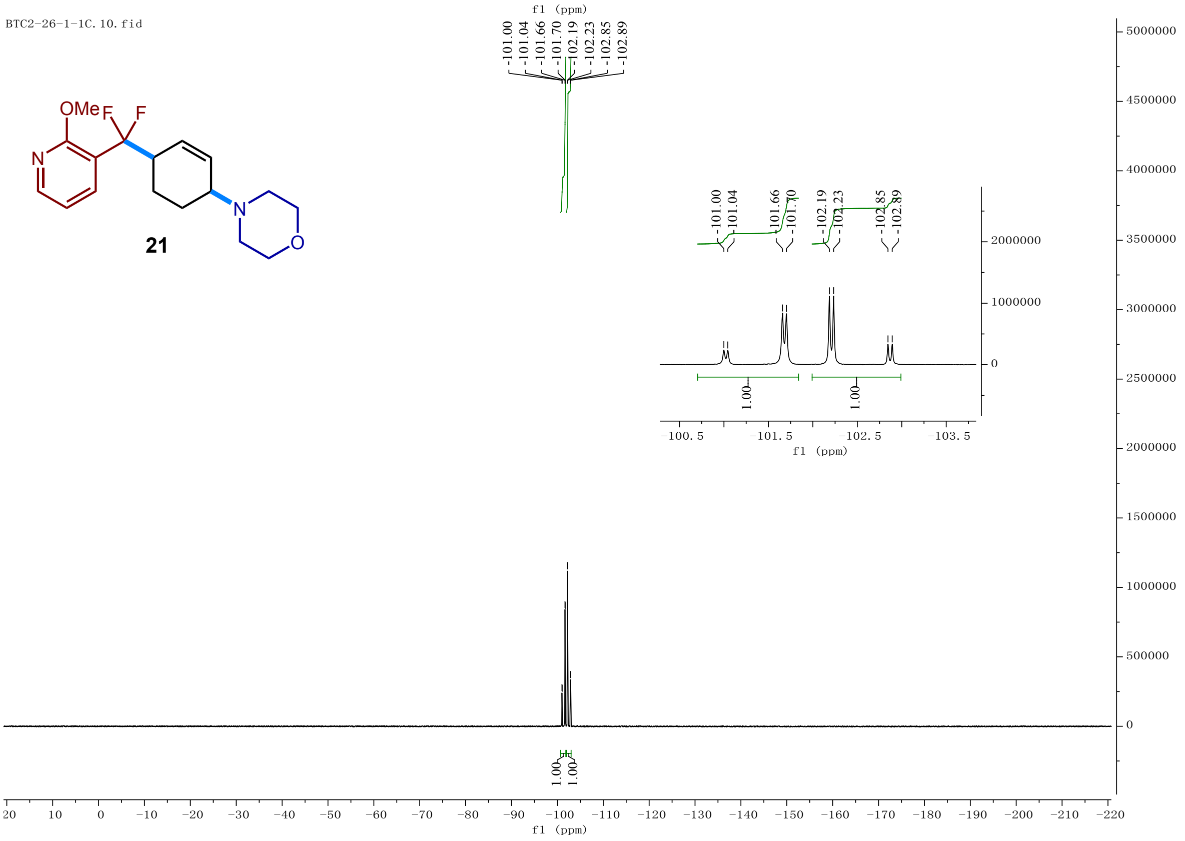
BTC2-26-1-1C.10.fid
-101.00
-101.04
-101.66
-101.70
-102.19
-102.23
-102.85
-102.89
OMe
F
F
N
O
21
1.00
1.00
f1 (ppm)

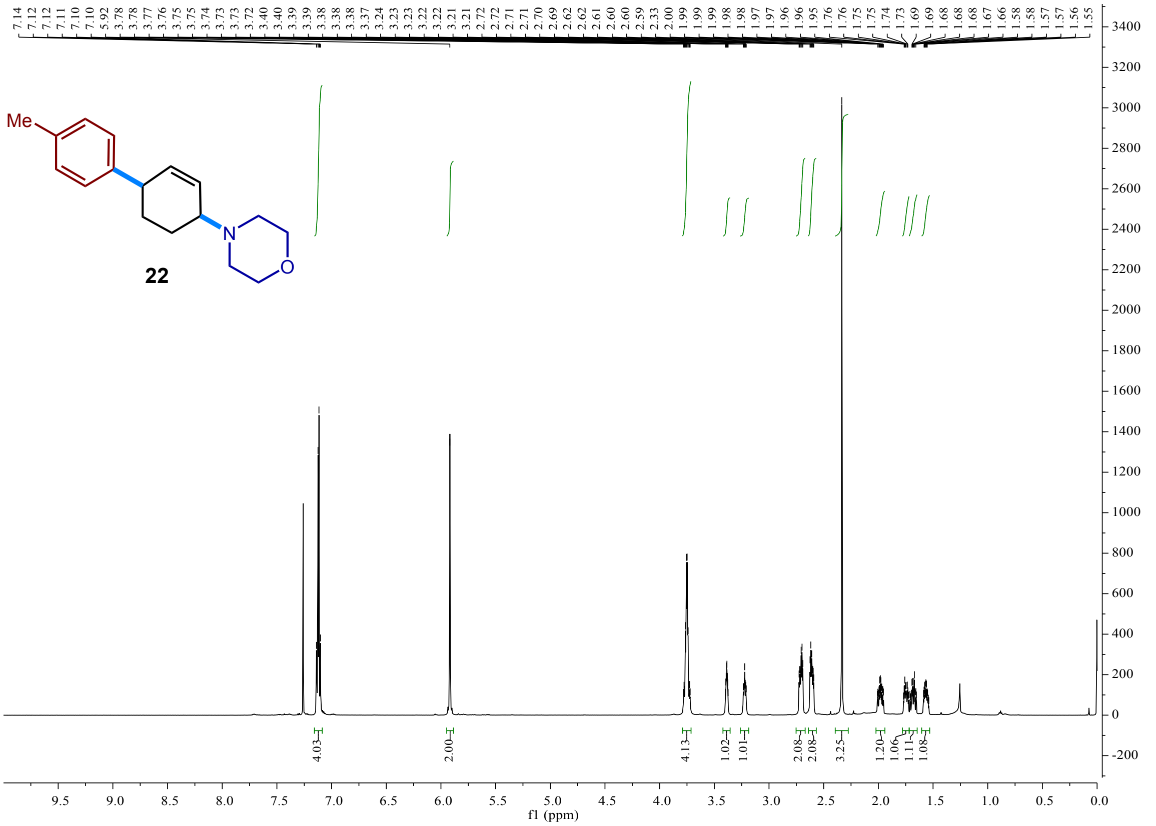
7.14 7.12 7.12 7.11 7.10 7.10 5.92 3.78 3.78 3.77 3.76 3.75 3.75 3.74 3.73 3.73 3.72 3.40 3.39 3.39 3.38 3.38 3.38 3.37 3.24 3.23 3.23 3.22 3.22 3.21 3.21 2.72 2.72 2.71 2.71 2.70 2.69 2.62 2.61 2.60 2.60 2.59 2.33 2.00 1.99 1.99 1.99 1.98 1.98 1.97 1.97 1.96 1.96 1.95 1.76 1.76 1.75 1.75 1.74 1.73 1.69 1.69 1.69 1.68 1.68 1.67 1.66 1.58 1.58 1.58 1.57 1.57 1.56 1.55
Me
N
O
22
4.03
2.00
4.13
1.02
1.01
2.08
2.08
3.25
1.20
1.06
1.11
1.08
f1 (ppm)

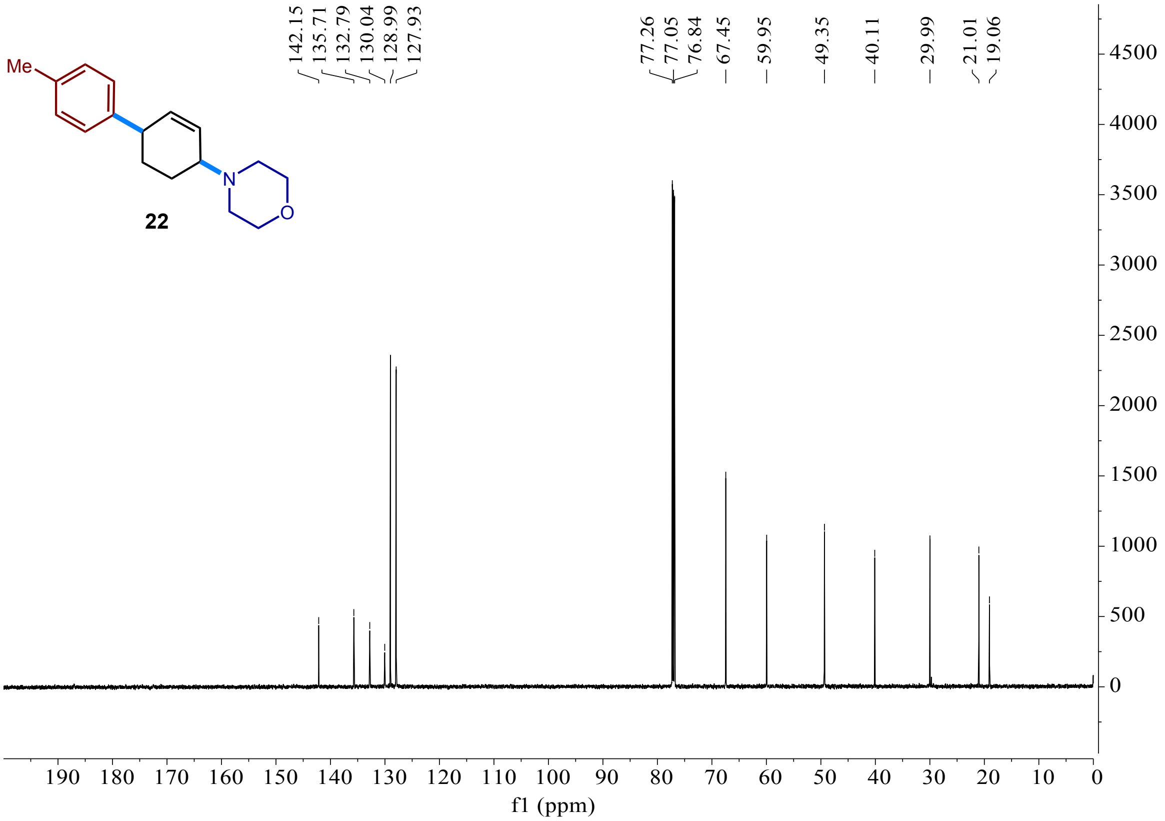
142.15
135.71
132.79
130.04
128.99
127.93
77.26
77.05
76.84
67.45
59.95
49.35
40.11
29.99
21.01
19.06
Me
N
O
22
f1 (ppm)

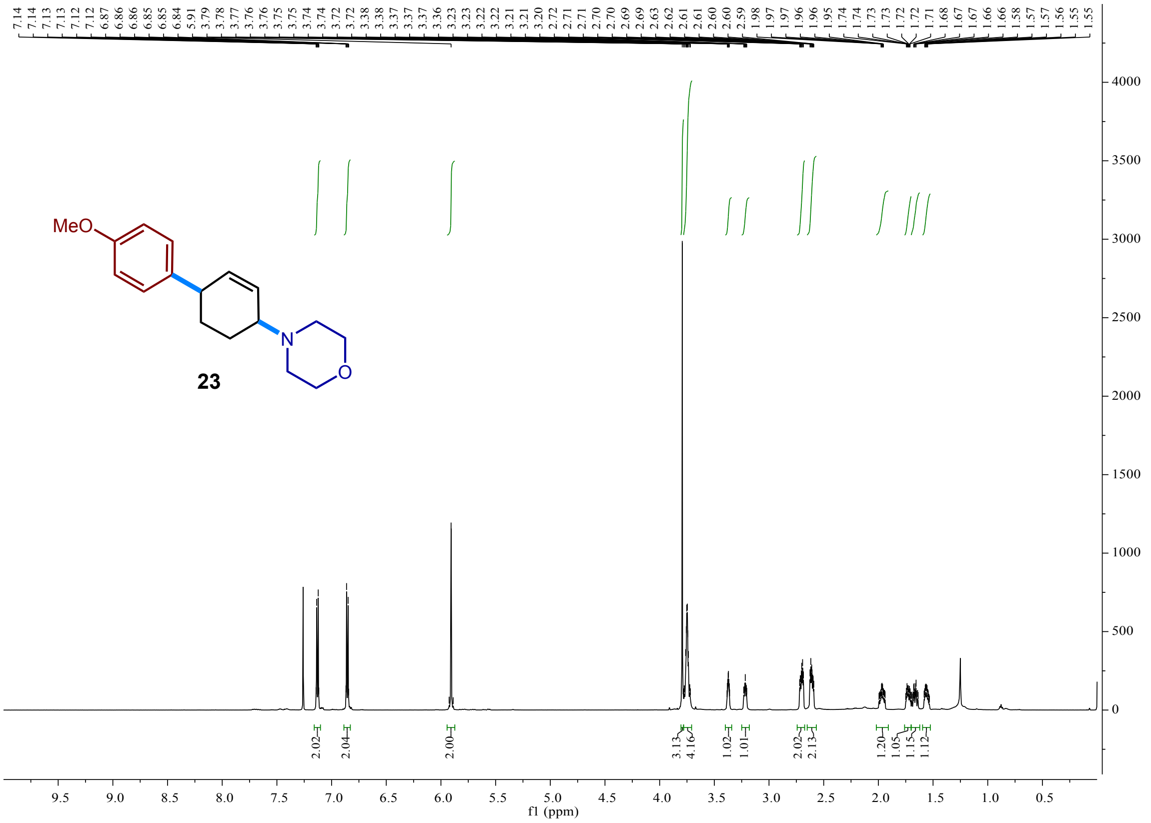

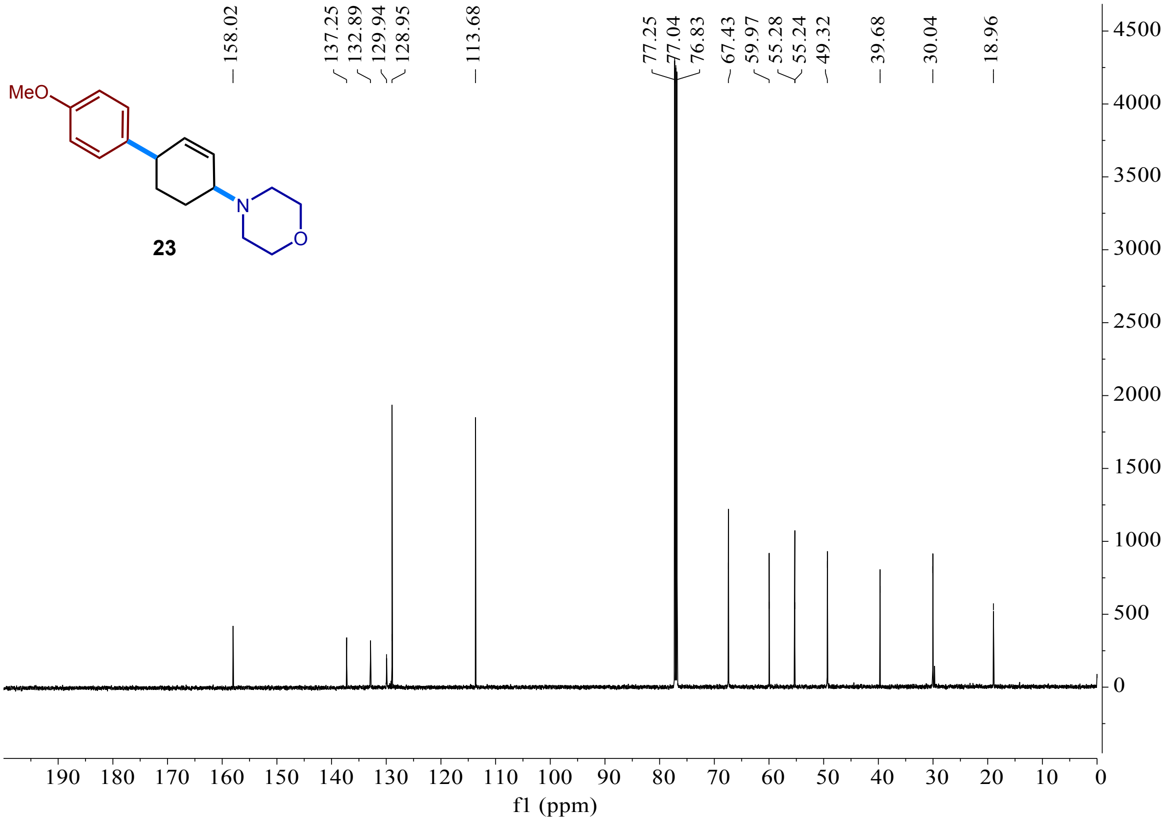

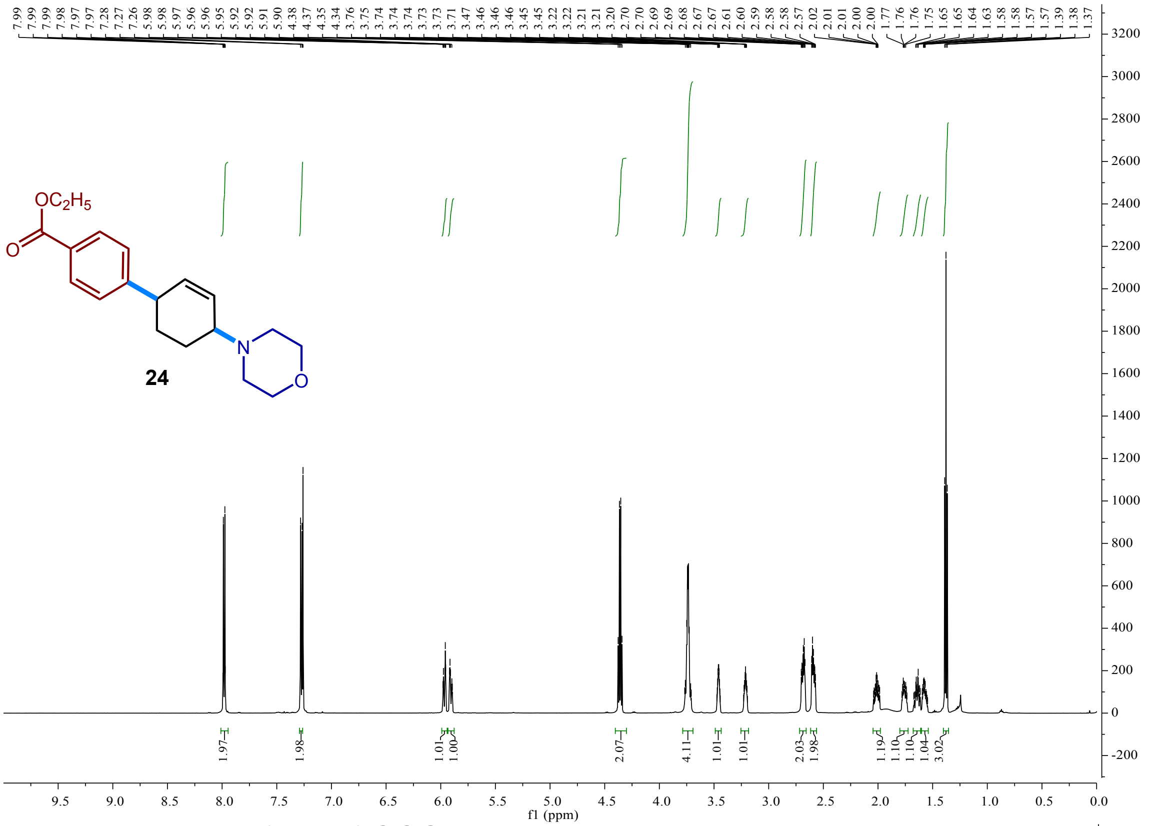

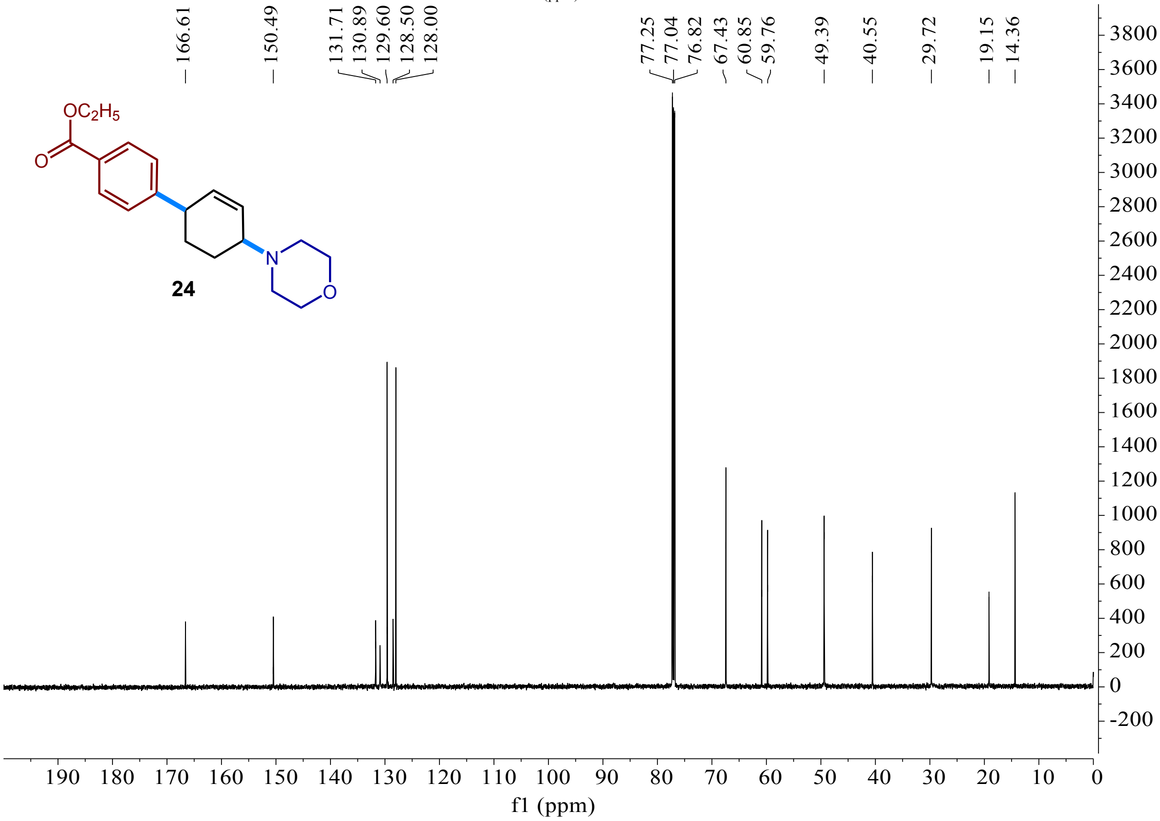

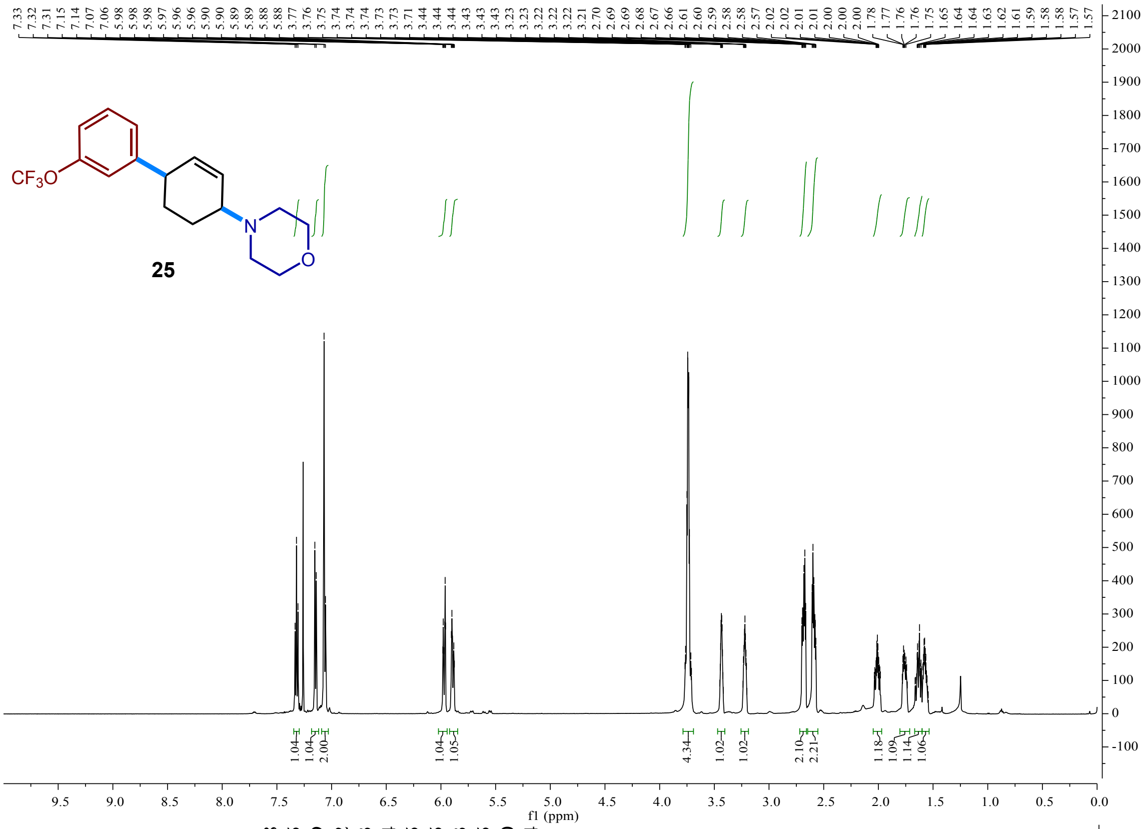
CF3O
25
f1 (ppm)

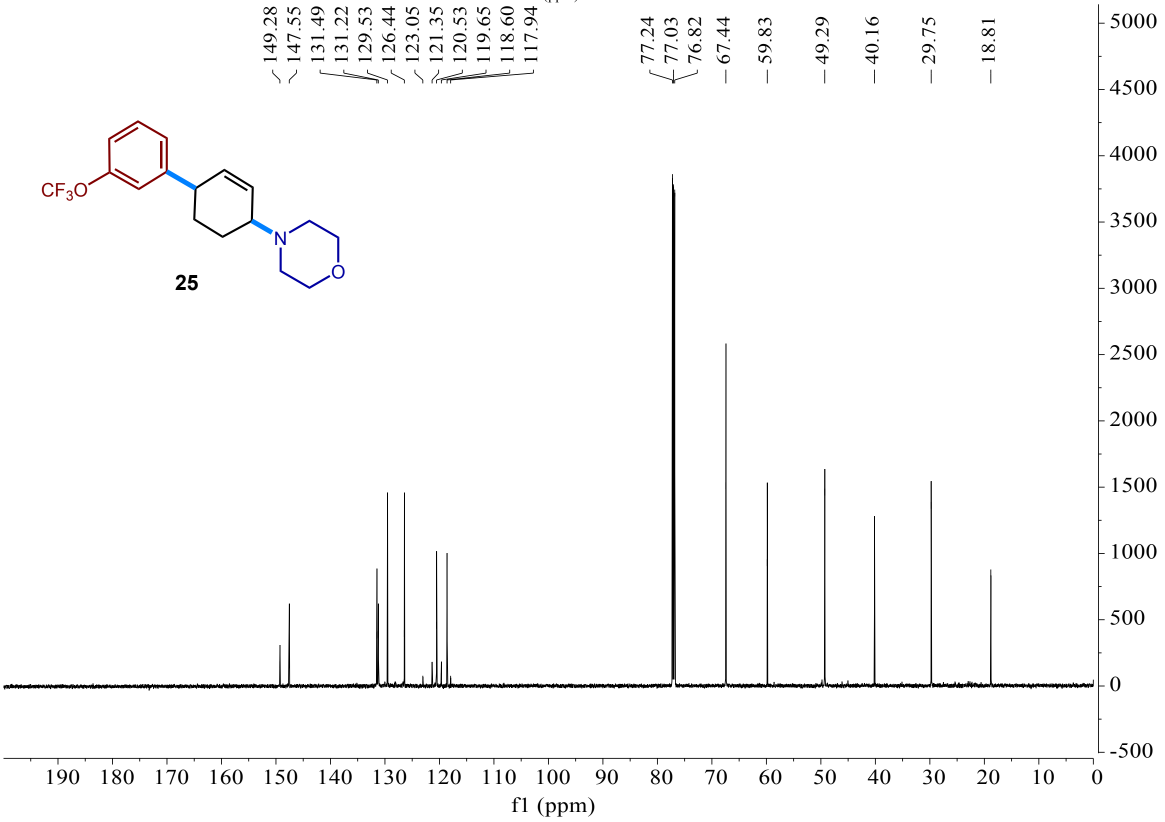
CF3O
25
f1 (ppm)

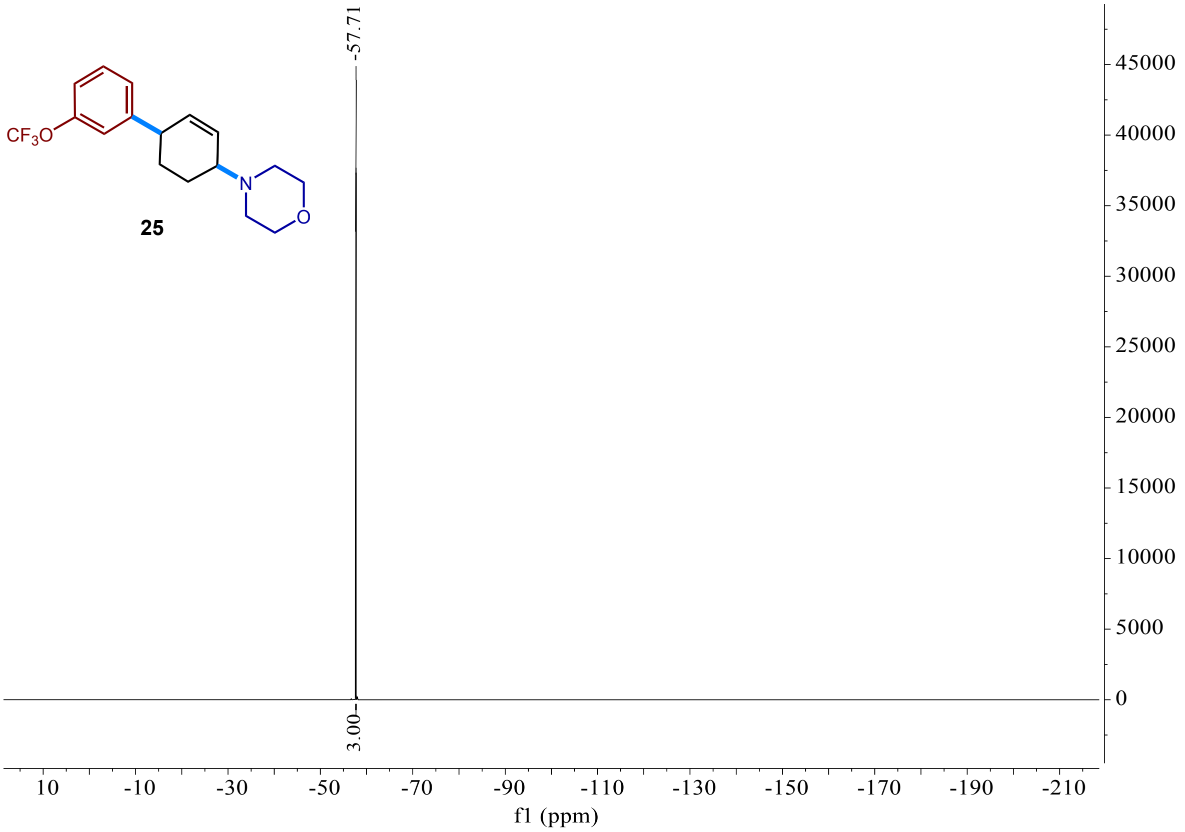
-57.71
CF3O
N
O
25
3.00
f1 (ppm)

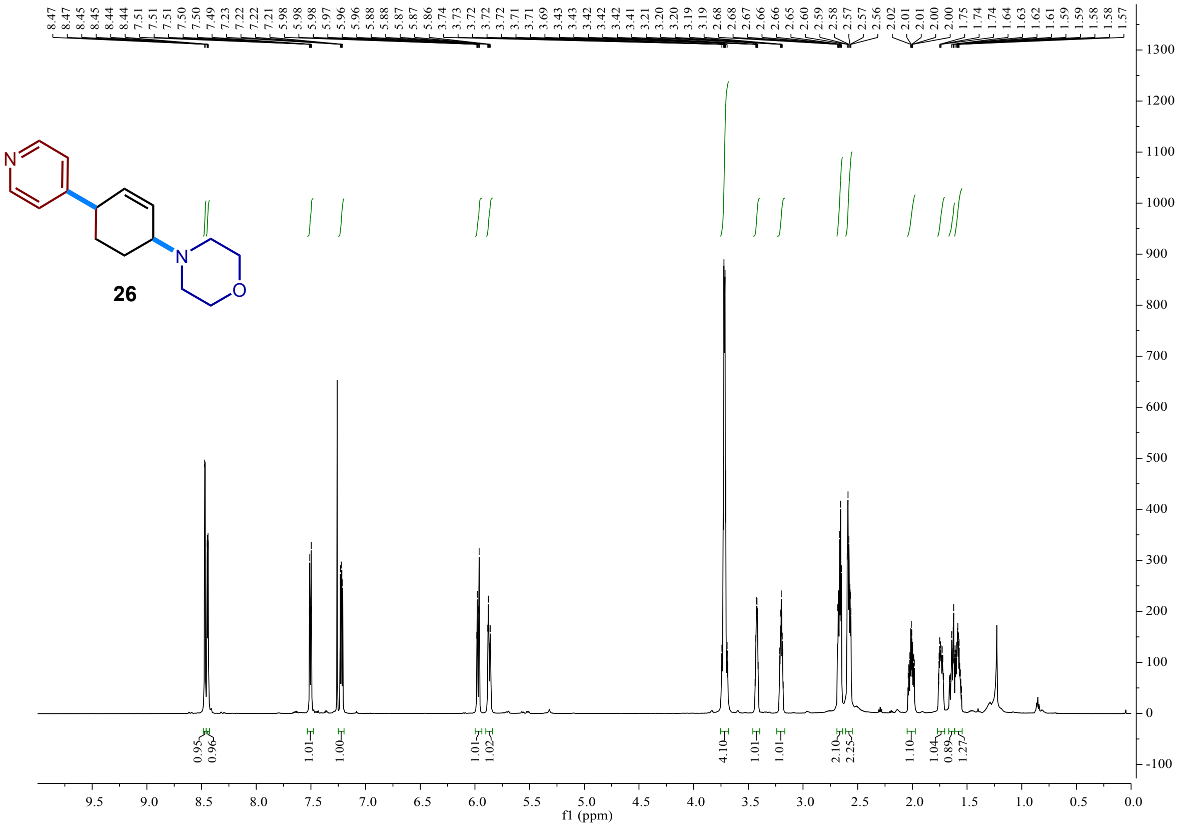
8.47 8.47 8.45 8.45 8.44 8.44 7.51 7.51 7.50 7.50 7.49 7.23 7.22 7.22 7.21 7.21 5.98 5.98 5.98 5.97 5.96 5.96 5.88 5.87 5.87 5.86 5.86 3.74 3.73 3.72 3.72 3.71 3.71 3.69 3.43 3.43 3.42 3.42 3.41 3.41 3.21 3.20 3.20 3.19 3.19 2.68 2.68 2.67 2.66 2.66 2.65 2.60 2.59 2.58 2.57 2.57 2.56 2.02 2.01 2.01 2.00 2.00 1.75 1.74 1.64 1.63 1.62 1.61 1.59 1.59 1.58 1.58 1.57
N
N
O
26
0.95 0.96 1.01 1.00 1.01 1.02 4.10 1.01 1.01 2.10 2.25 1.10 1.04 0.89 1.27
f1 (ppm)

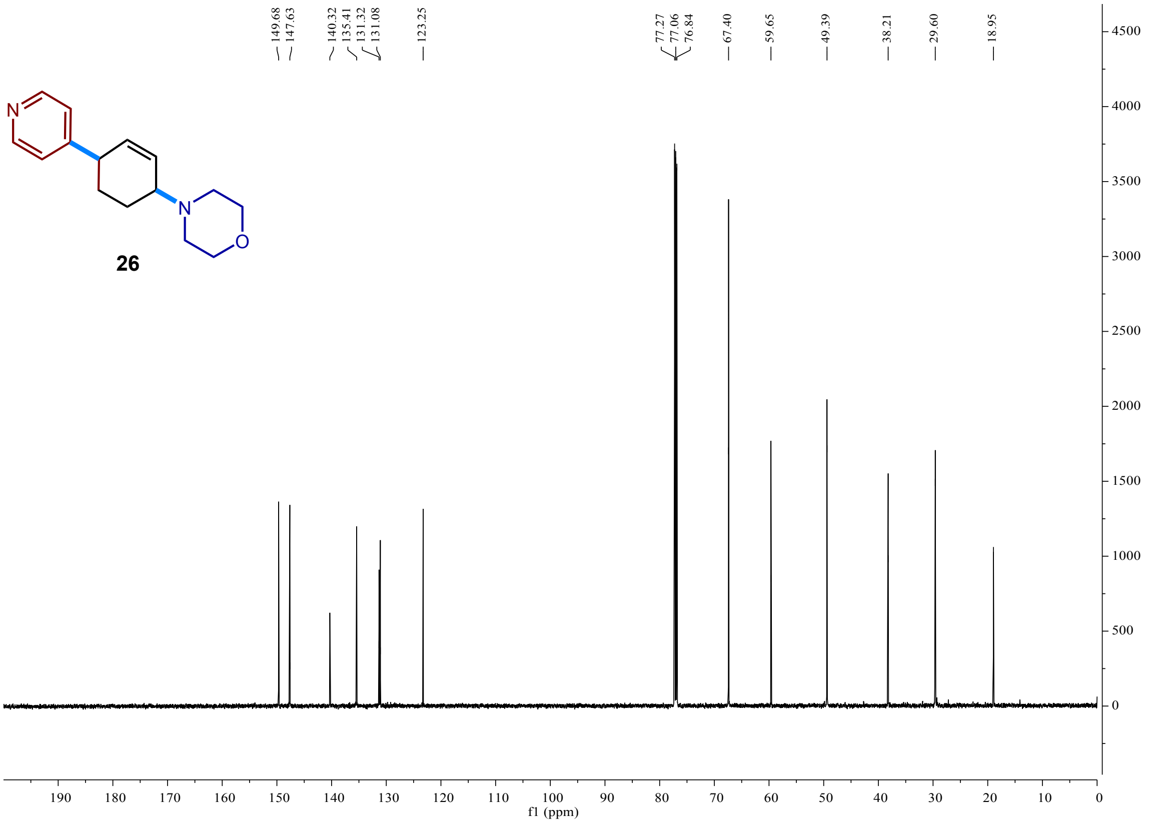
149.68
147.63
140.32
135.41
131.32
131.08
123.25
77.27
77.06
76.84
67.40
59.65
49.39
38.21
29.60
18.95
N
O
26
f1 (ppm)

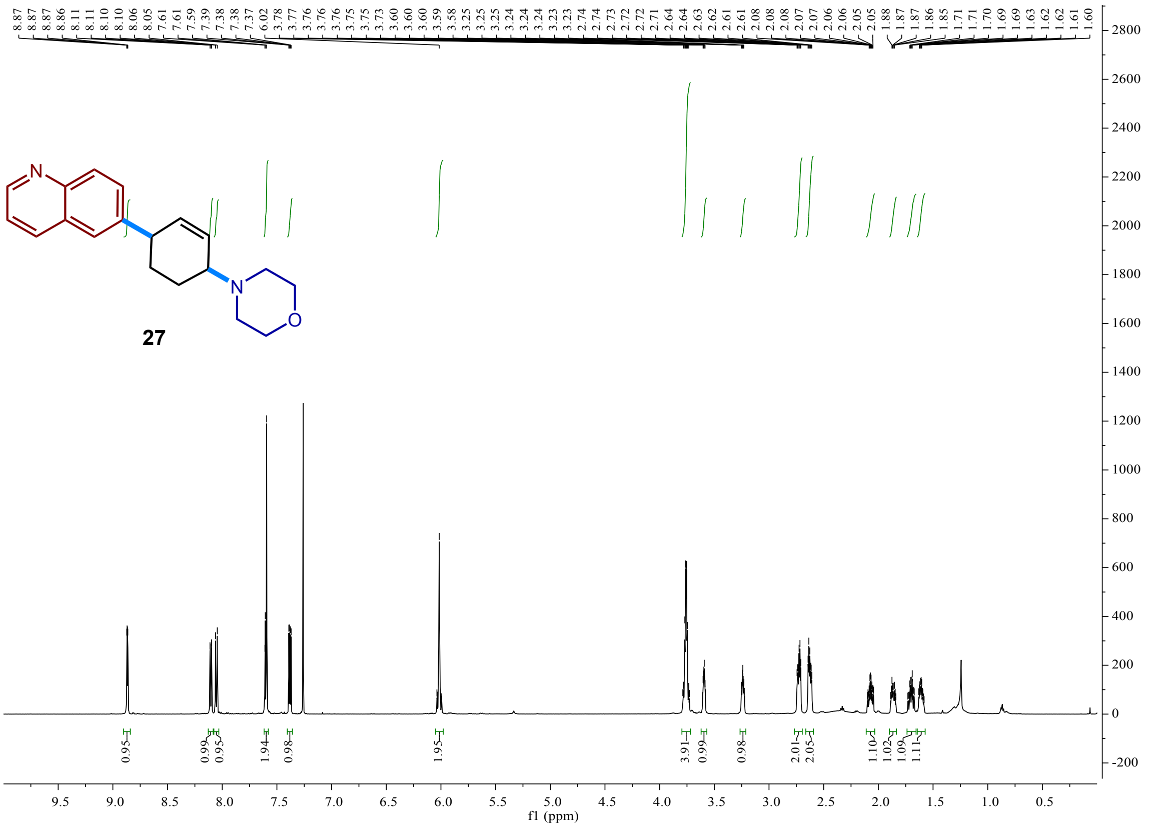
8.87 8.87 8.87 8.86 8.11 8.11 8.10 8.10 8.06 8.05 7.61 7.61 7.59 7.39 7.38 7.38 7.37 6.02 3.78 3.77 3.76 3.76 3.76 3.75 3.75 3.73 3.60 3.60 3.60 3.59 3.58 3.25 3.25 3.25 3.24 3.24 3.24 3.23 3.23 2.74 2.74 2.73 2.72 2.72 2.71 2.64 2.64 2.63 2.62 2.61 2.61 2.08 2.08 2.08 2.07 2.07 2.06 2.06 2.05 2.05 1.88 1.87 1.87 1.86 1.85 1.71 1.71 1.70 1.69 1.69 1.63 1.62 1.62 1.61 1.60
N
O
27
0.95
0.99
0.95
1.94
0.98
1.95
3.91
0.99
0.98
2.01
2.05
1.10
1.02
1.09
1.11
f1 (ppm)

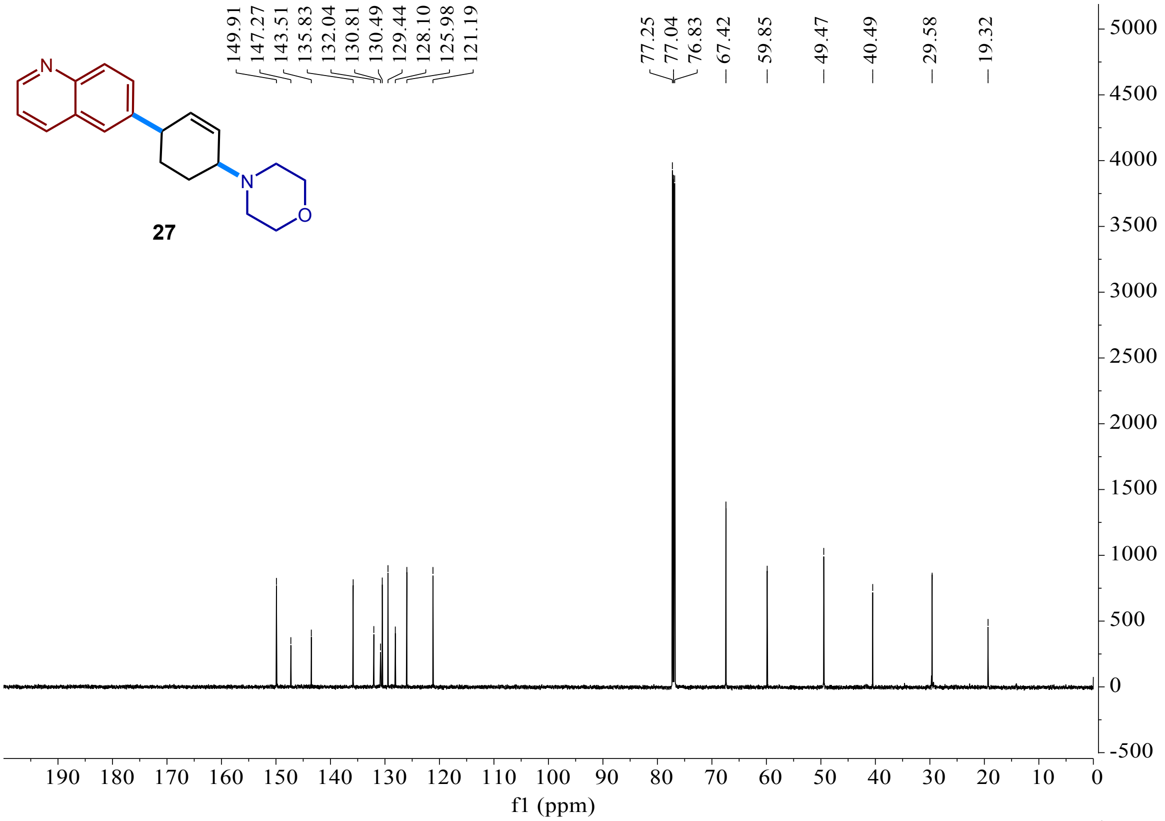
149.91
147.27
143.51
135.83
132.04
130.81
130.49
129.44
128.10
125.98
121.19
77.25
77.04
76.83
67.42
59.85
49.47
40.49
29.58
19.32
N
O
27
f1 (ppm)

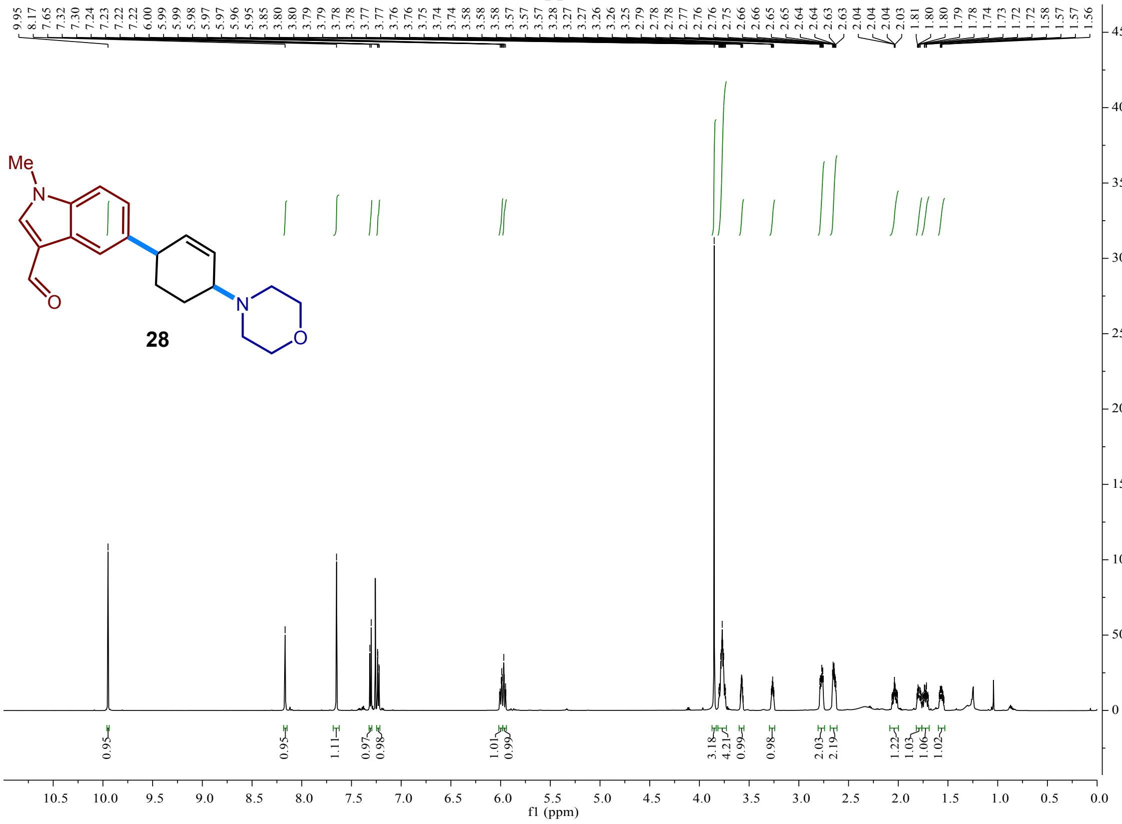
9.95 8.17 7.65 7.32 7.30 7.24 7.23 7.22 7.22 6.00 5.99 5.99 5.98 5.97 5.97 5.96 5.95 3.85 3.80 3.79 3.79 3.78 3.78 3.77 3.76 3.76 3.75 3.74 3.74 3.58 3.58 3.58 3.57 3.57 3.28 3.27 3.27 3.26 3.26 3.25 2.79 2.78 2.78 2.77 2.76 2.76 2.75 2.66 2.65 2.65 2.64 2.64 2.63 2.63 2.04 2.04 2.04 2.03 1.81 1.80 1.80 1.79 1.78 1.74 1.73 1.72 1.72 1.58 1.57 1.56
Me
N
O
N
O
28
0.95 0.95 1.11 0.97 0.98 1.01 0.99 3.18 4.21 0.99 0.98 2.03 2.19 1.22 1.03 1.06 1.02
f1 (ppm)

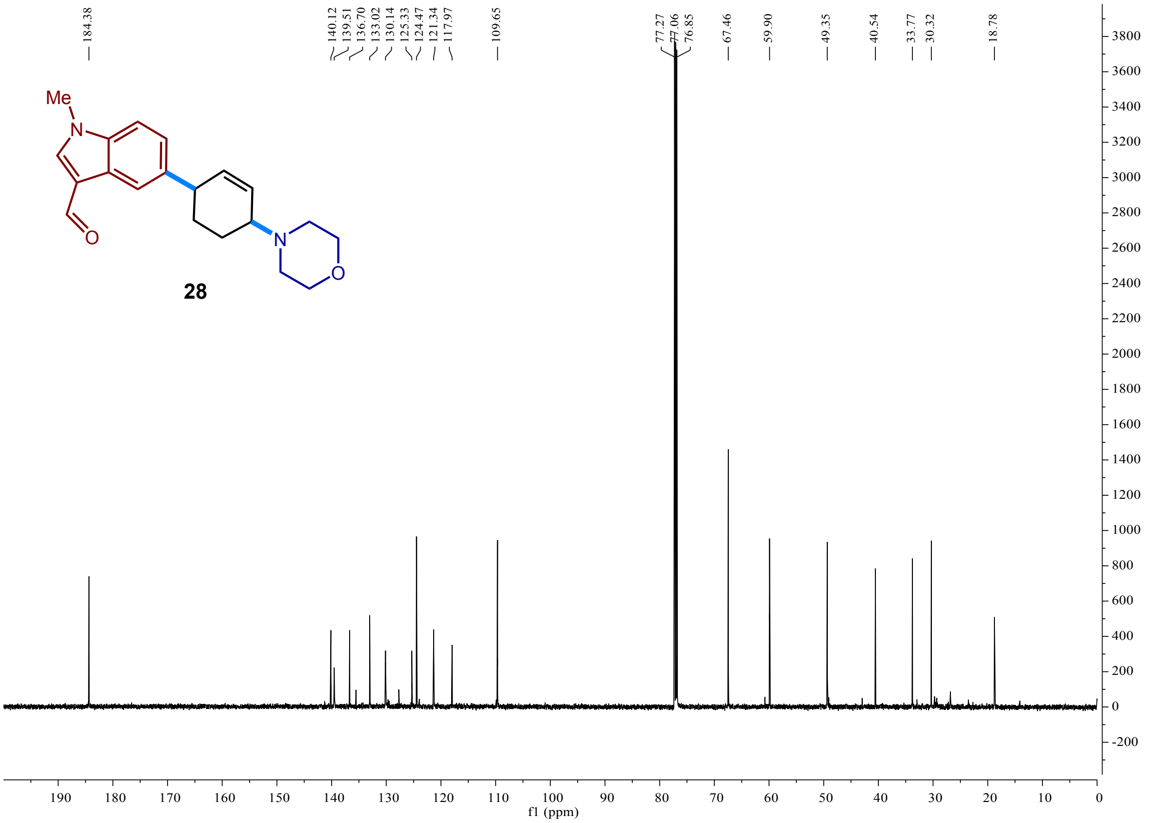
184.38
140.12
139.51
136.70
133.02
130.14
125.33
124.47
121.34
117.97
109.65
77.27
77.06
76.85
67.46
59.90
49.35
40.54
33.77
30.32
18.78
Me
N
O
N
O
28
f1 (ppm)

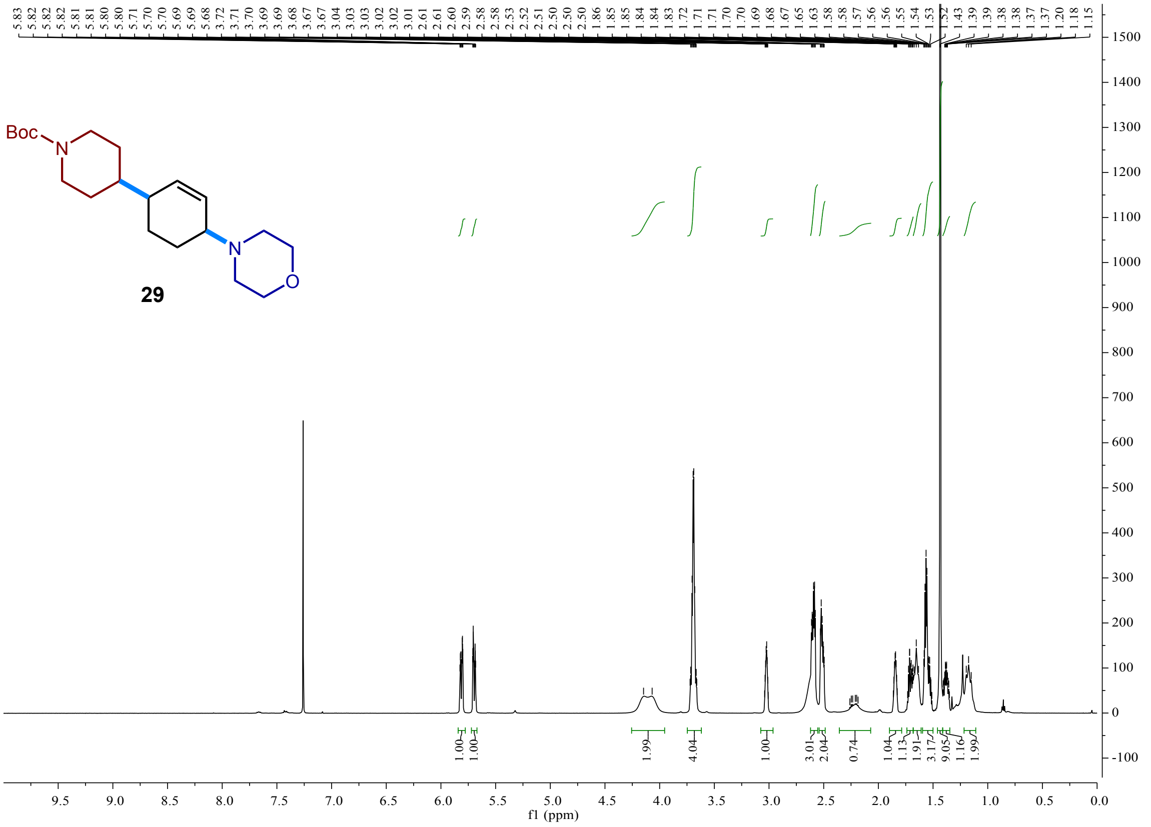
5.83 5.82 5.82 5.81 5.81 5.80 5.80 5.71 5.70 5.70 5.69 5.69 5.68 3.72 3.71 3.70 3.69 3.69 3.68 3.67 3.67 3.04 3.03 3.03 3.02 3.02 3.01 2.61 2.61 2.60 2.59 2.58 2.58 2.53 2.52 2.51 2.50 2.50 2.50 1.86 1.85 1.85 1.84 1.84 1.83 1.72 1.71 1.71 1.70 1.70 1.69 1.68 1.67 1.65 1.63 1.58 1.58 1.57 1.56 1.56 1.55 1.54 1.53 1.52 1.43 1.39 1.39 1.38 1.38 1.37 1.37 1.20 1.18 1.15
Boc
N
N
O
29
1.00
1.00
1.99
4.04
1.00
3.01
2.04
0.74
1.04
1.13
1.91
3.17
9.05
1.16
1.99
f1 (ppm)

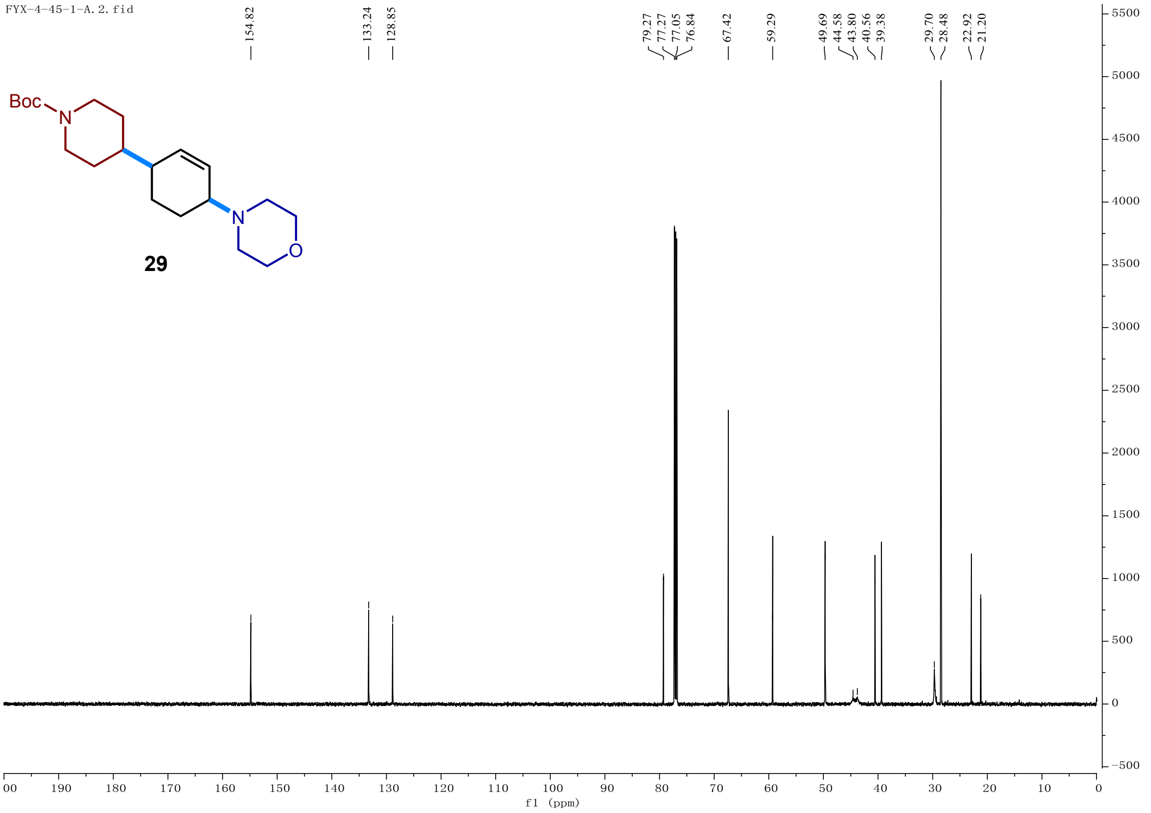
FYX-4-45-1-A.2.fid
154.82
133.24
128.85
79.27
77.27
77.05
76.84
67.42
59.29
49.69
44.58
43.80
40.56
39.38
29.70
28.48
22.92
21.20
Boc
N
N
O
29
f1 (ppm)

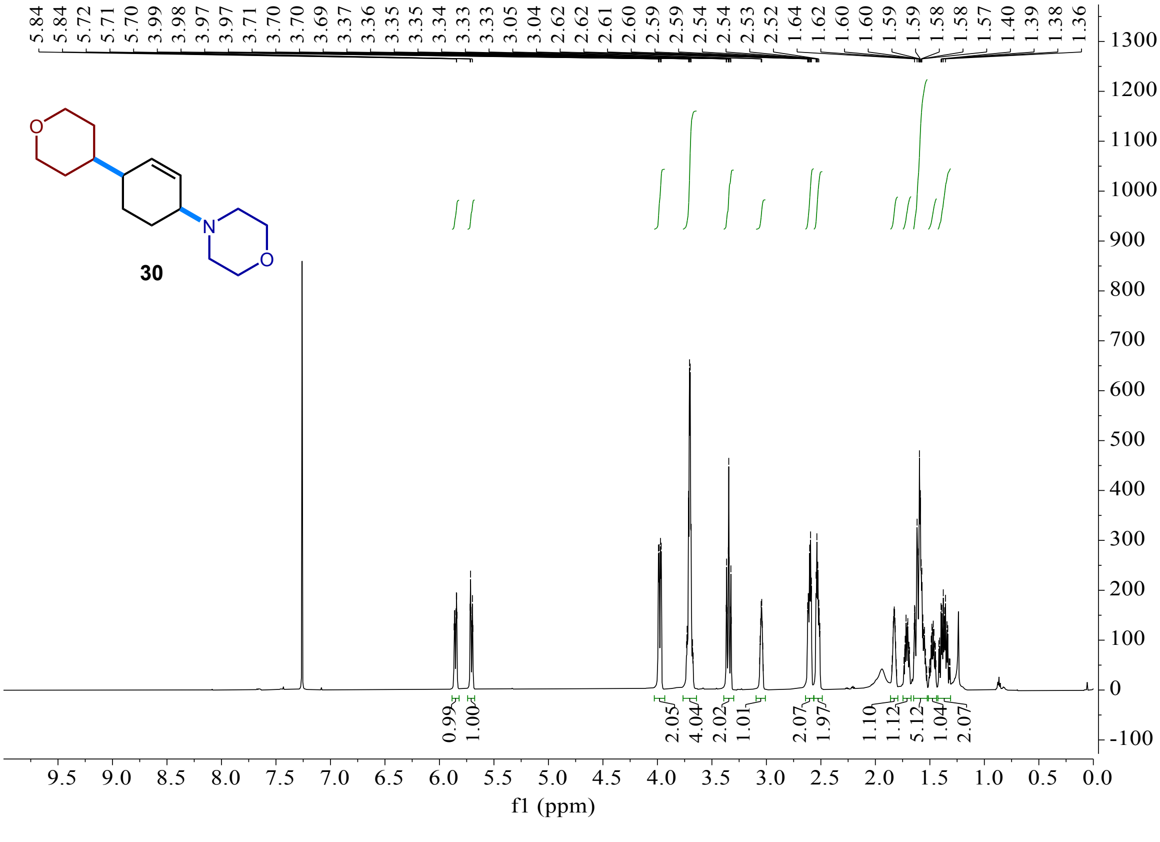
5.84 5.84 5.72 5.71 5.70 3.99 3.98 3.97 3.97 3.71 3.70 3.70 3.69 3.37 3.36 3.35 3.35 3.34 3.33 3.33 3.05 3.04 2.62 2.62 2.61 2.60 2.59 2.59 2.54 2.54 2.53 2.52 1.64 1.62 1.60 1.60 1.59 1.59 1.58 1.58 1.57 1.40 1.39 1.38 1.36
O
N
O
30
0.99
1.00
2.05
4.04
2.02
1.01
2.07
1.97
1.10
1.12
5.12
1.04
2.07
f1 (ppm)

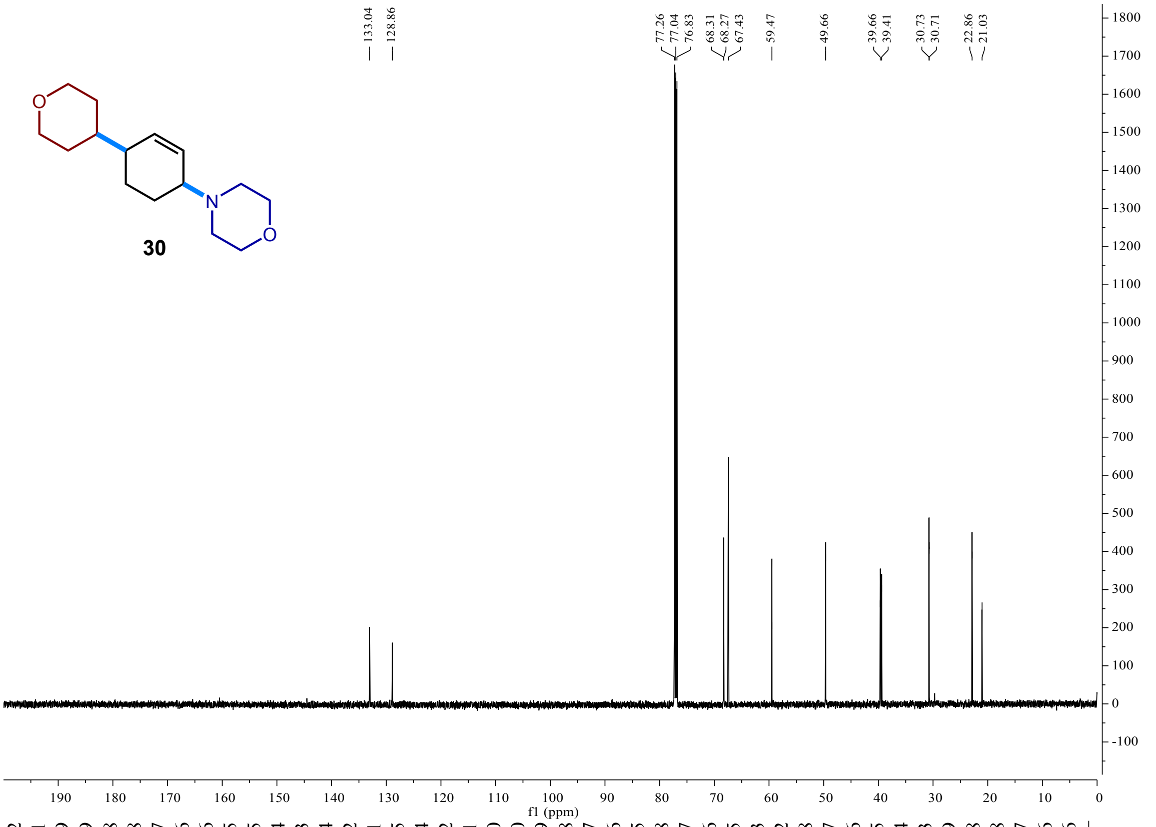
30
O
N
133.04
128.86
77.26
77.04
76.83
68.31
68.27
67.43
59.47
49.66
39.66
39.41
30.73
30.71
22.86
21.03
f1 (ppm)

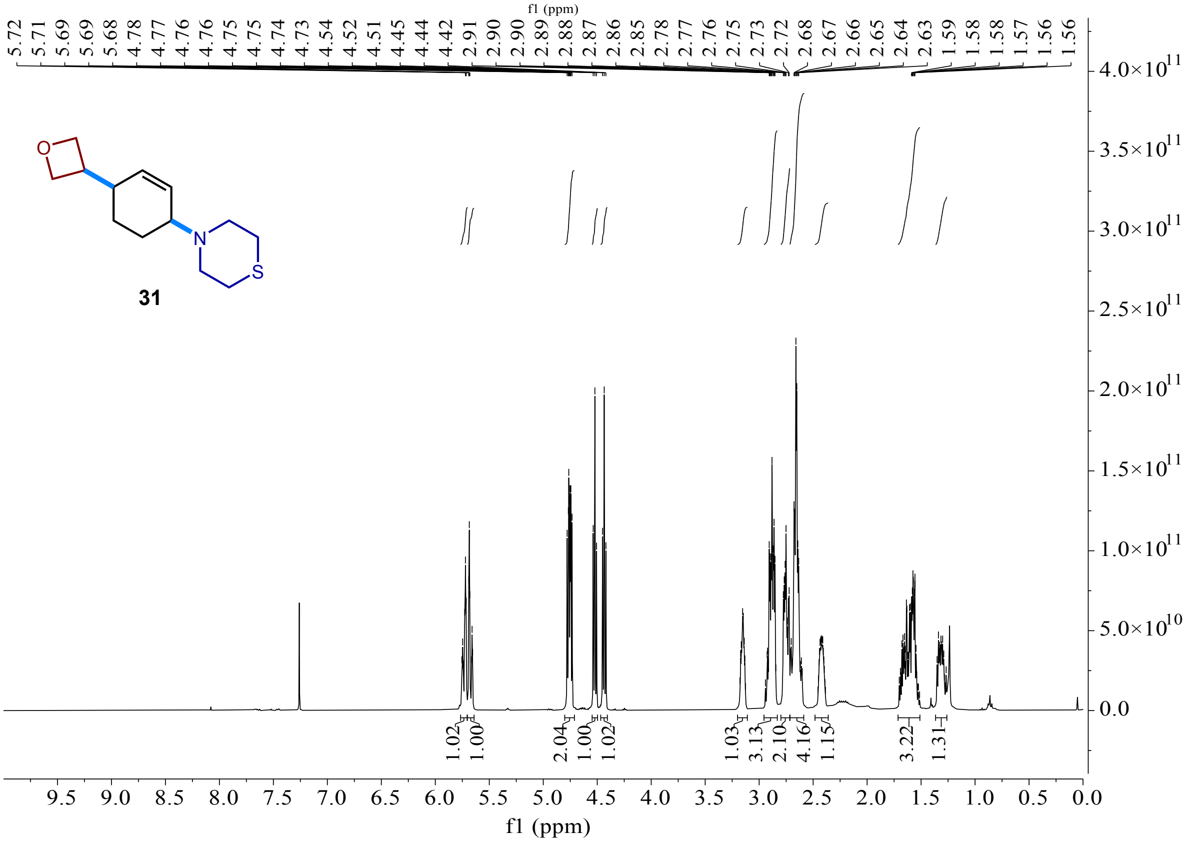
31
O
N
S
5.72 5.71 5.69 5.69 5.68 4.78 4.77 4.76 4.76 4.75 4.75 4.74 4.73 4.54 4.52 4.51 4.45 4.44 4.42 2.91 2.90 2.90 2.89 2.88 2.87 2.86 2.85 2.78 2.77 2.76 2.75 2.73 2.72 2.68 2.67 2.66 2.65 2.64 2.63 1.59 1.58 1.58 1.57 1.56 1.56
1.02 1.00 2.04 1.00 1.02 1.03 3.13 2.10 4.16 1.15 3.22 1.31
f1 (ppm)

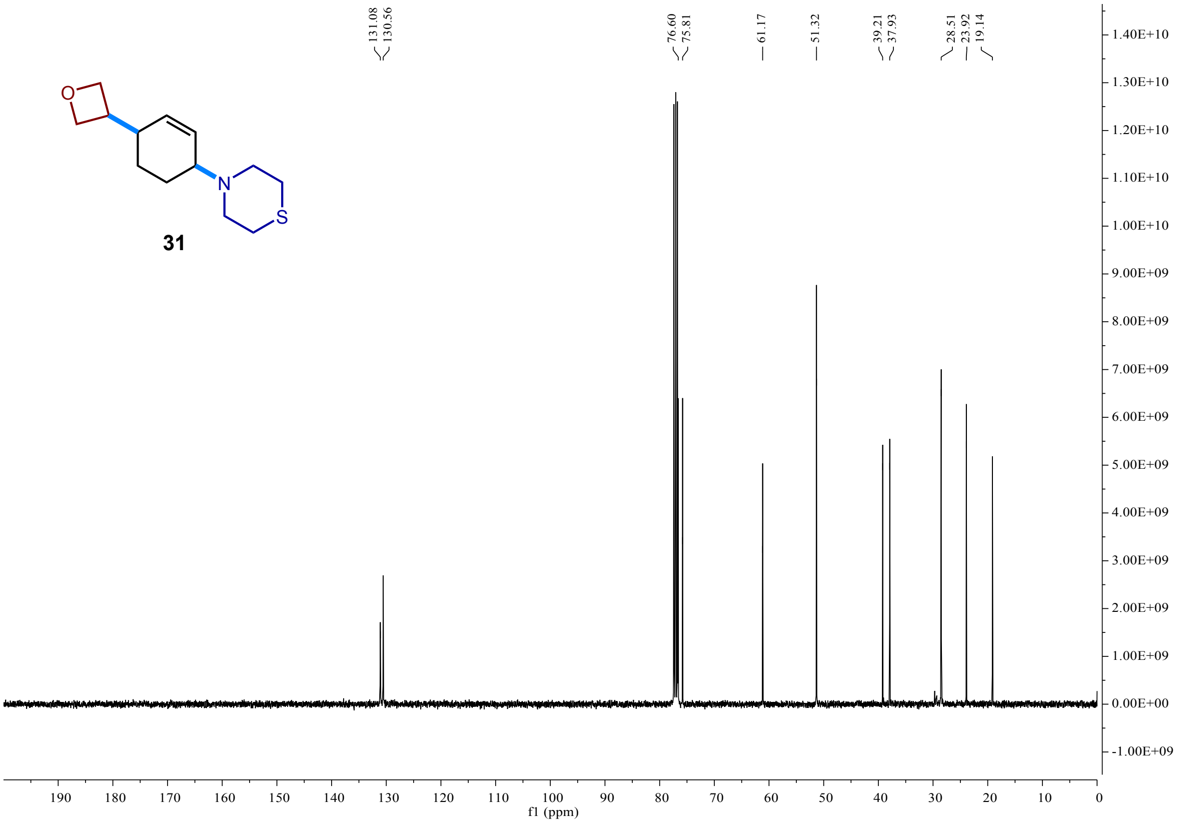
131.08
130.56
76.60
75.81
61.17
51.32
39.21
37.93
28.51
23.92
19.14
31
f1 (ppm)

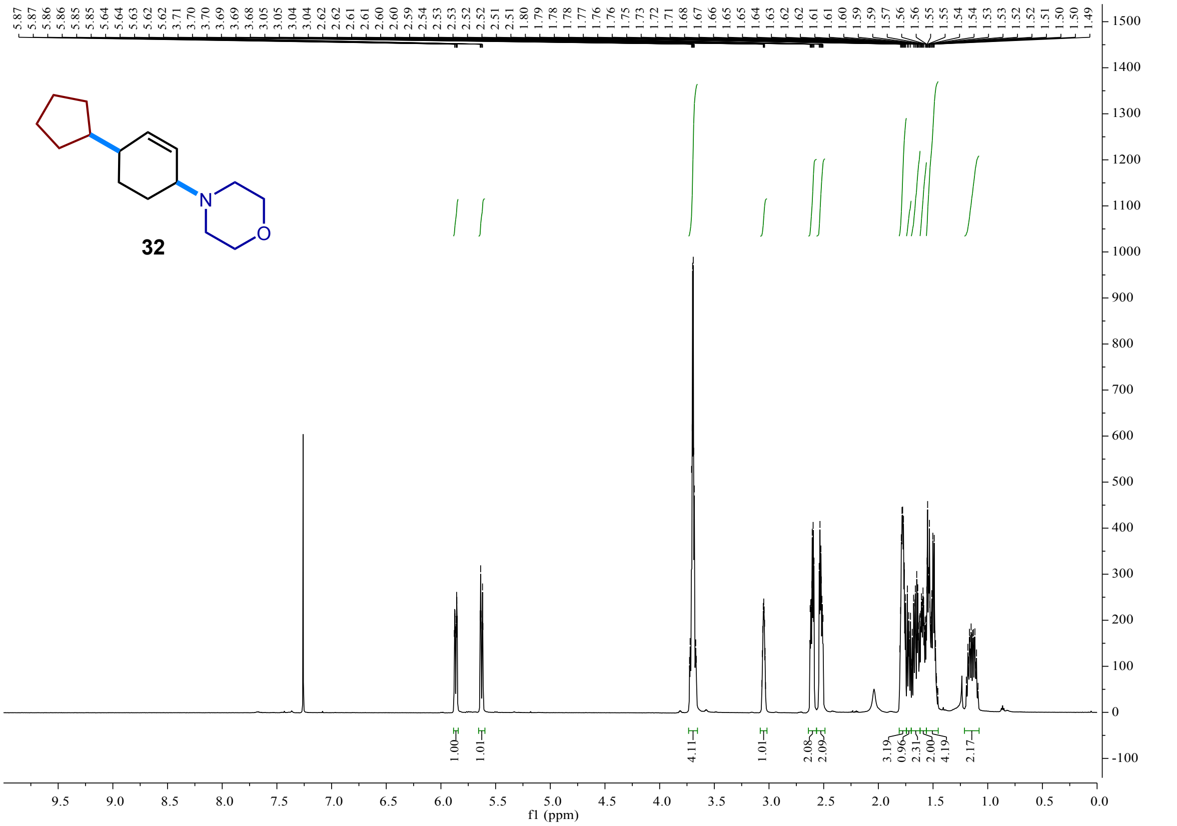
32
f1 (ppm)

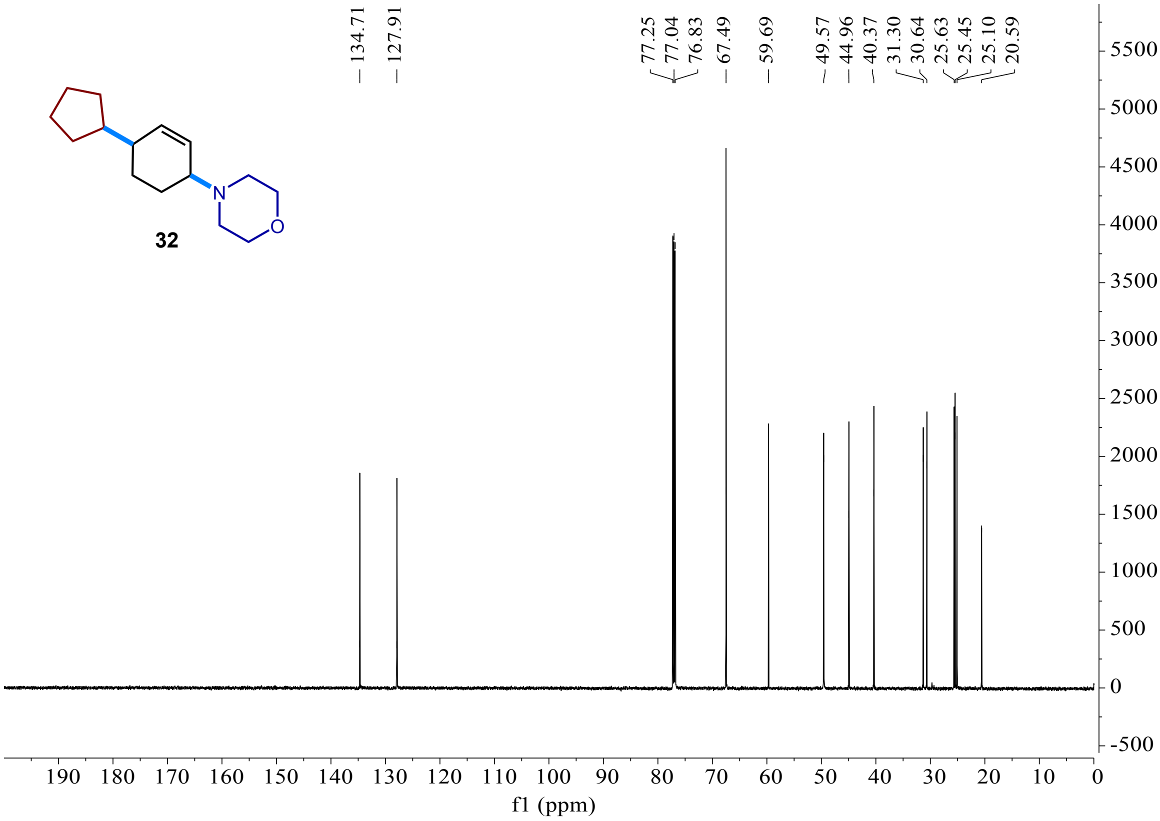
32
134.71
127.91
77.25
77.04
76.83
67.49
59.69
49.57
44.96
40.37
31.30
30.64
25.63
25.45
25.10
20.59
f1 (ppm)

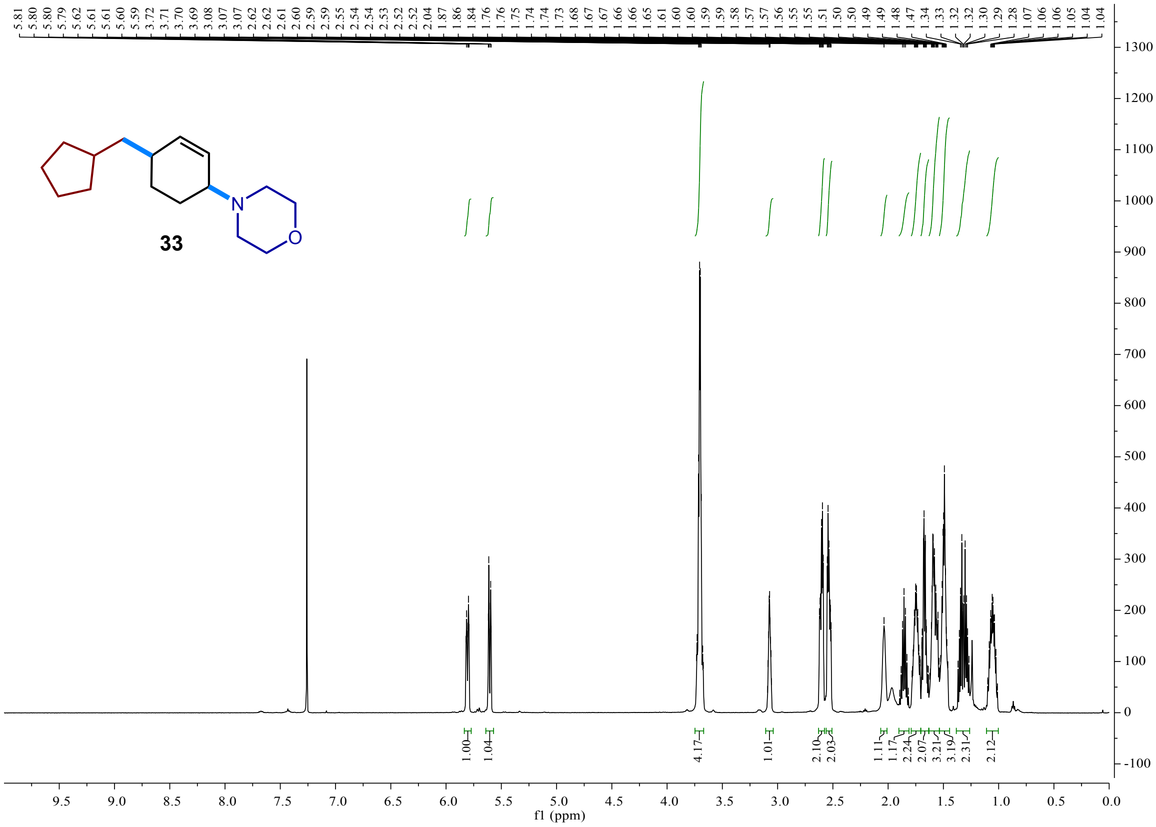
33
1.00
1.04
4.17
1.01
2.10
2.03
1.11
1.17
2.24
2.07
3.21
3.19
2.31
2.12
f1 (ppm)

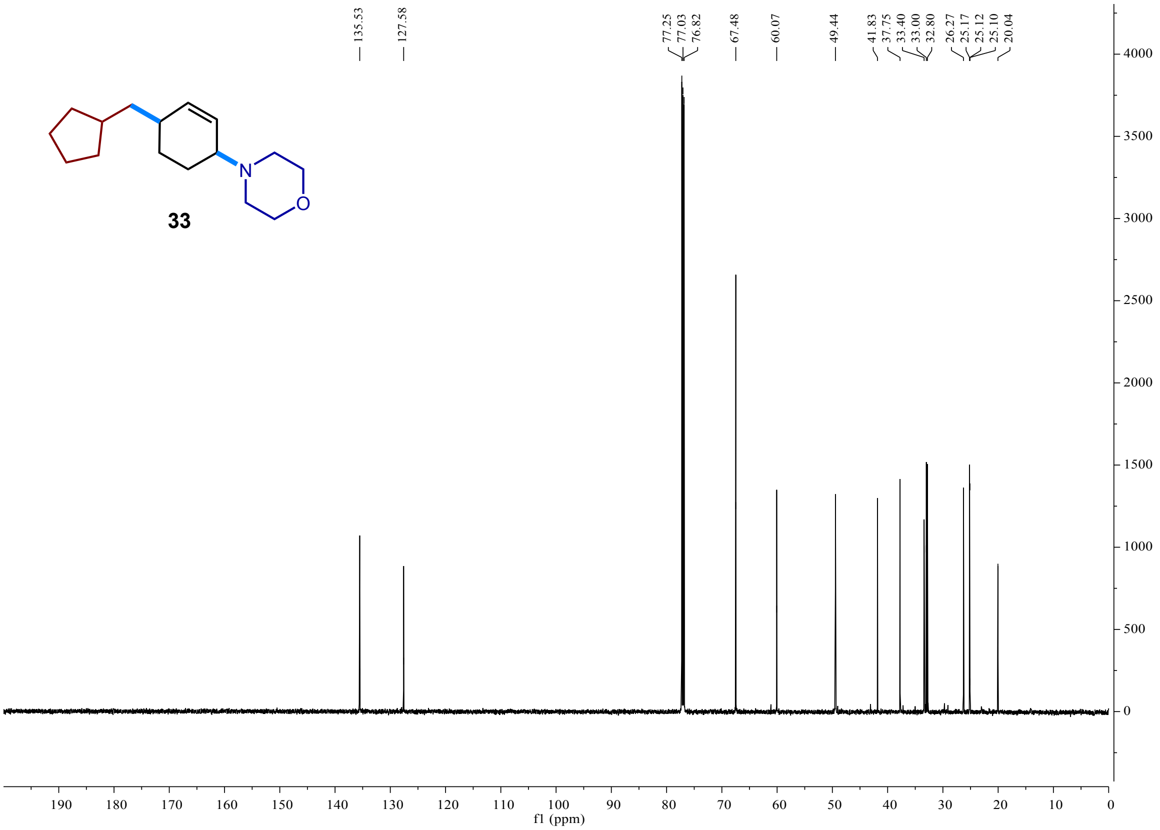

135.53
127.58
77.25
77.03
76.82
67.48
60.07
49.44
41.83
37.75
33.40
33.00
32.80
26.27
25.17
25.12
25.10
20.04
N
O
33
f1 (ppm)

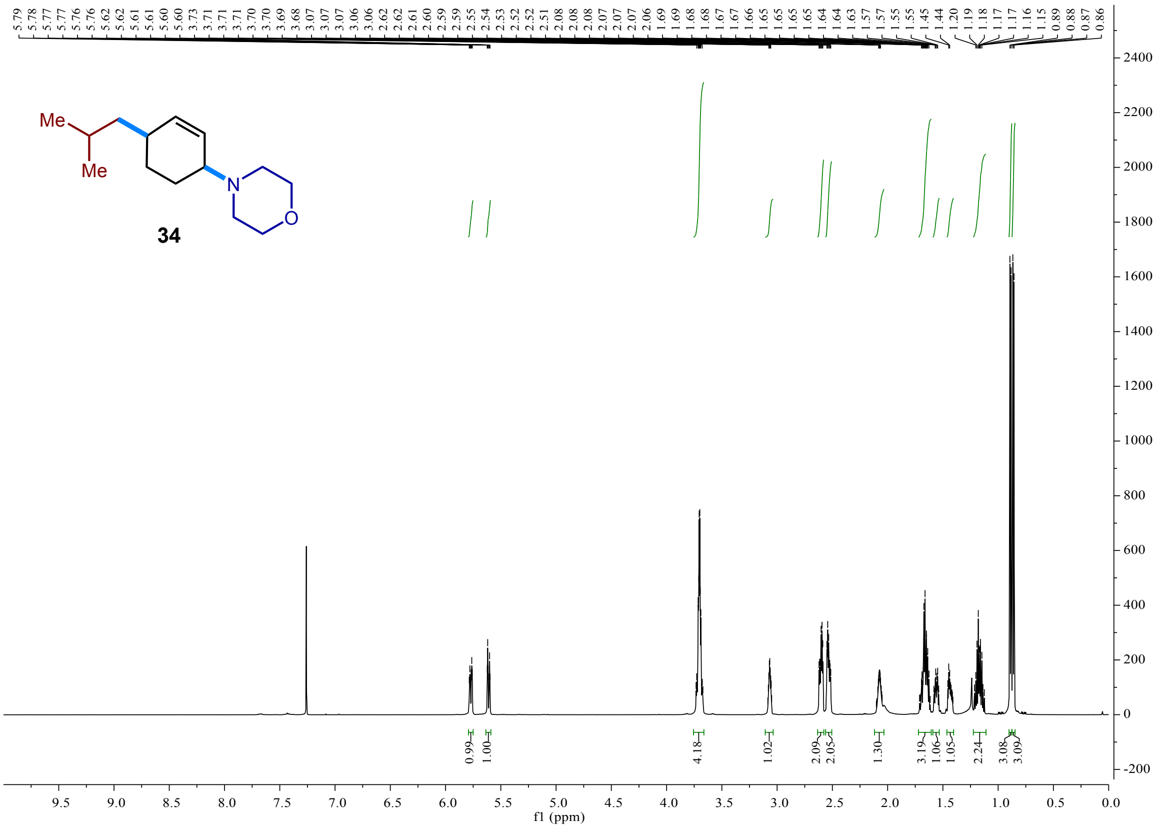

5.79 5.78 5.77 5.77 5.76 5.76 5.62 5.62 5.61 5.61 5.60 5.60 3.73 3.71 3.71 3.70 3.70 3.69 3.68 3.07 3.07 3.07 3.06 3.06 2.62 2.62 2.61 2.60 2.59 2.59 2.55 2.54 2.53 2.52 2.52 2.51 2.08 2.08 2.07 2.07 2.07 2.06 1.69 1.69 1.68 1.68 1.67 1.67 1.66 1.65 1.65 1.65 1.65 1.64 1.64 1.63 1.57 1.57 1.55 1.55 1.45 1.44 1.20 1.19 1.18 1.17 1.17 1.16 1.15 0.89 0.88 0.87 0.86
Me
Me
N
O
34
0.99
1.00
4.18
1.02
2.09
2.05
1.30
3.19
1.06
1.05
2.24
3.08
3.09
f1 (ppm)

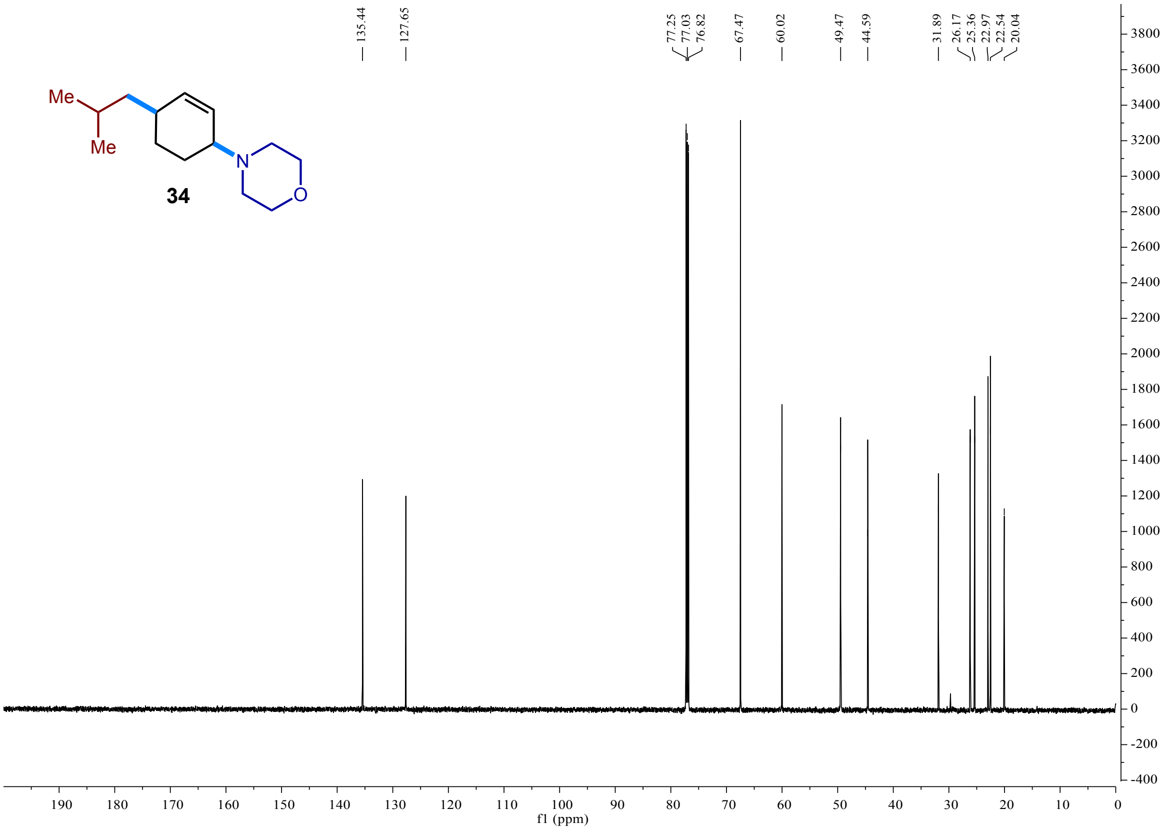

34

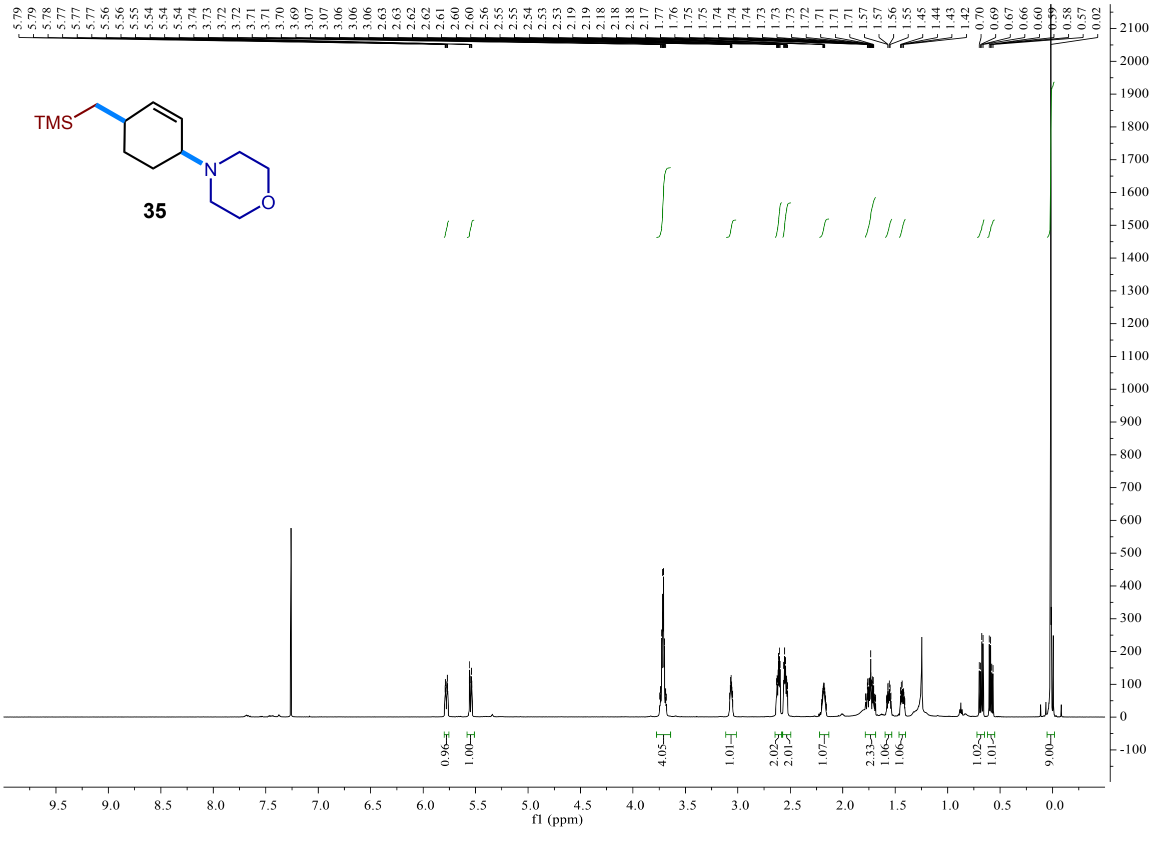

35

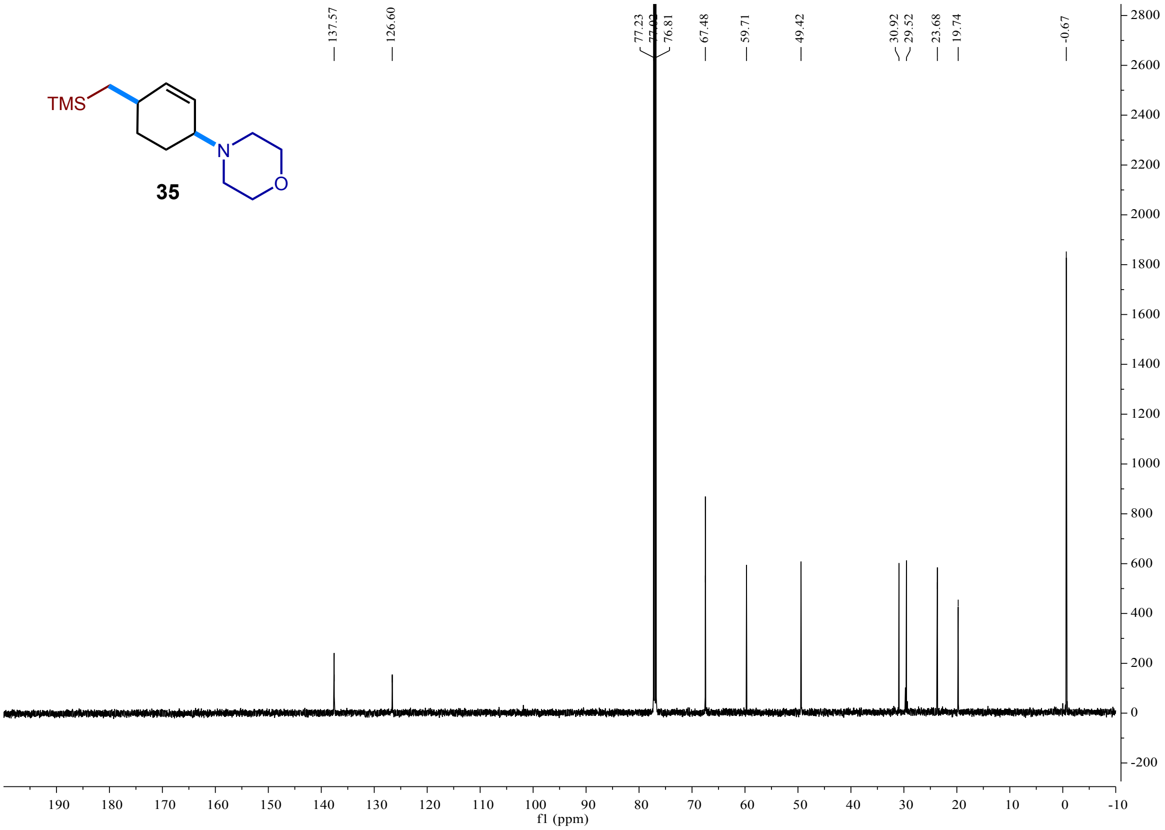

TMS
N
O
35
137.57
126.60
77.23
77.02
76.81
67.48
59.71
49.42
30.92
29.52
23.68
19.74
-0.67
f1 (ppm)

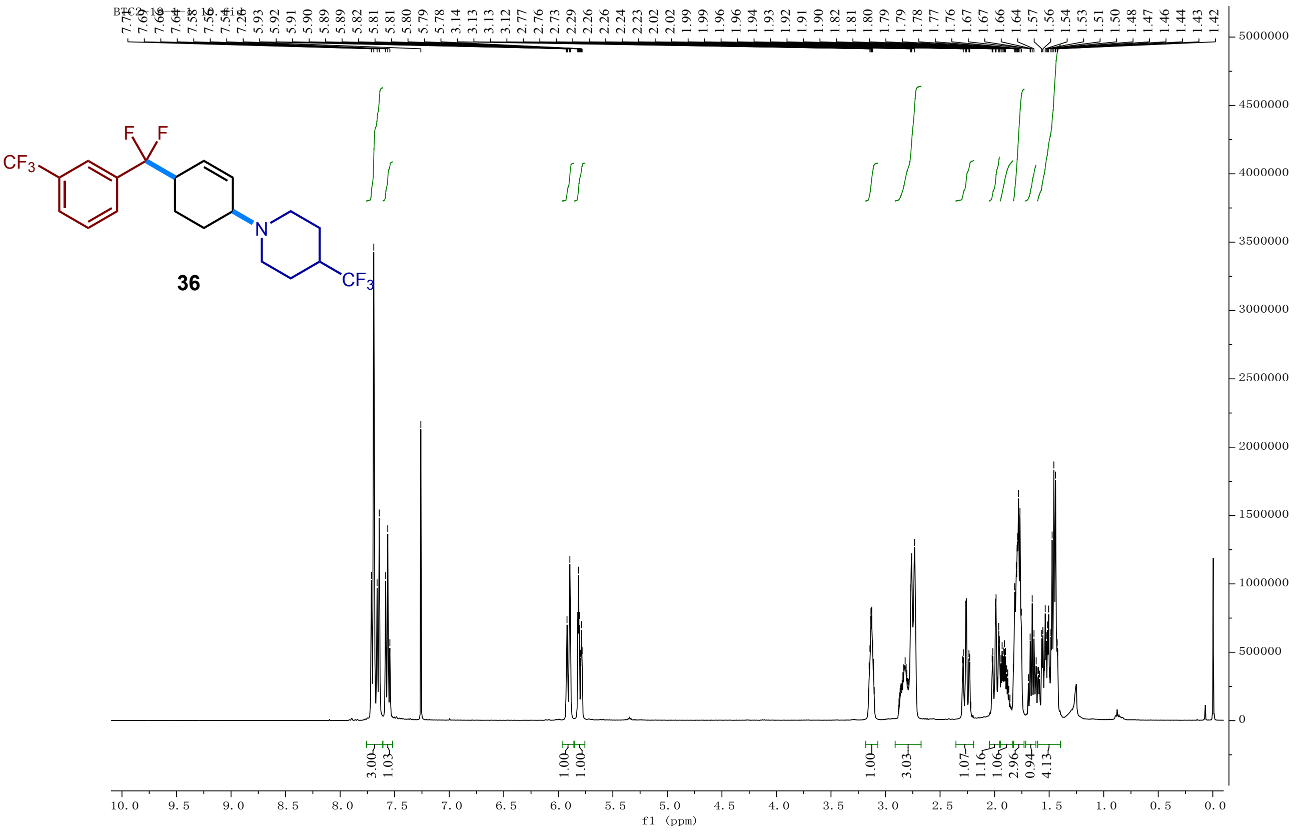

F F
CF_3
N
CF_3
36
7.72
7.69
7.66
7.64
7.58
7.56
7.54
7.26
5.93
5.92
5.91
5.90
5.89
5.89
5.82
5.81
5.81
5.80
5.79
5.78
3.14
3.13
3.13
3.12
2.77
2.76
2.73
2.29
2.26
2.26
2.24
2.23
2.02
1.99
1.99
1.96
1.96
1.94
1.93
1.92
1.91
1.90
1.82
1.81
1.80
1.79
1.78
1.77
1.76
1.67
1.67
1.66
1.64
1.57
1.56
1.54
1.53
1.51
1.50
1.48
1.47
1.46
1.44
1.43
1.42
3.00
1.03
1.00
1.00
1.00
3.03
1.07
1.16
1.06
2.96
0.94
4.13
f1 (ppm)

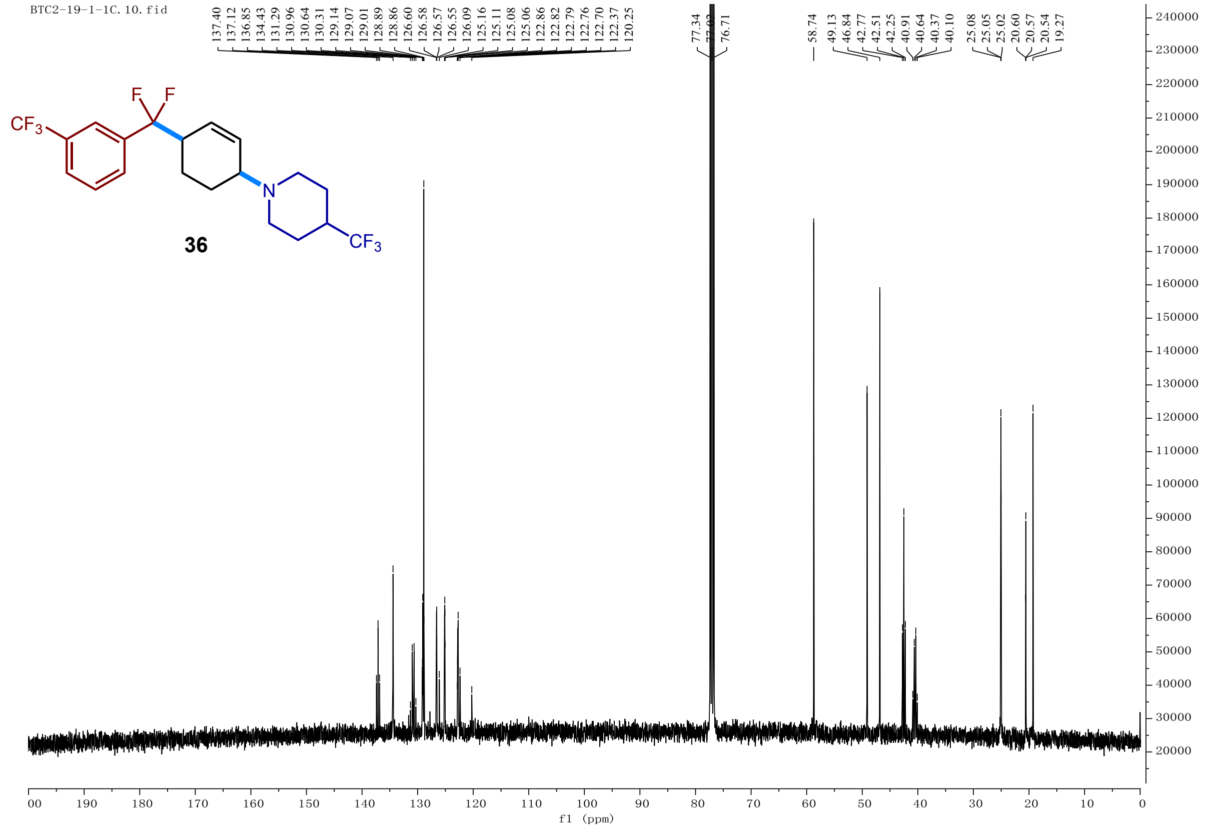
BTC2-19-1-1C.10.fid
CF3
F F
N
CF3
36
f1 (ppm)

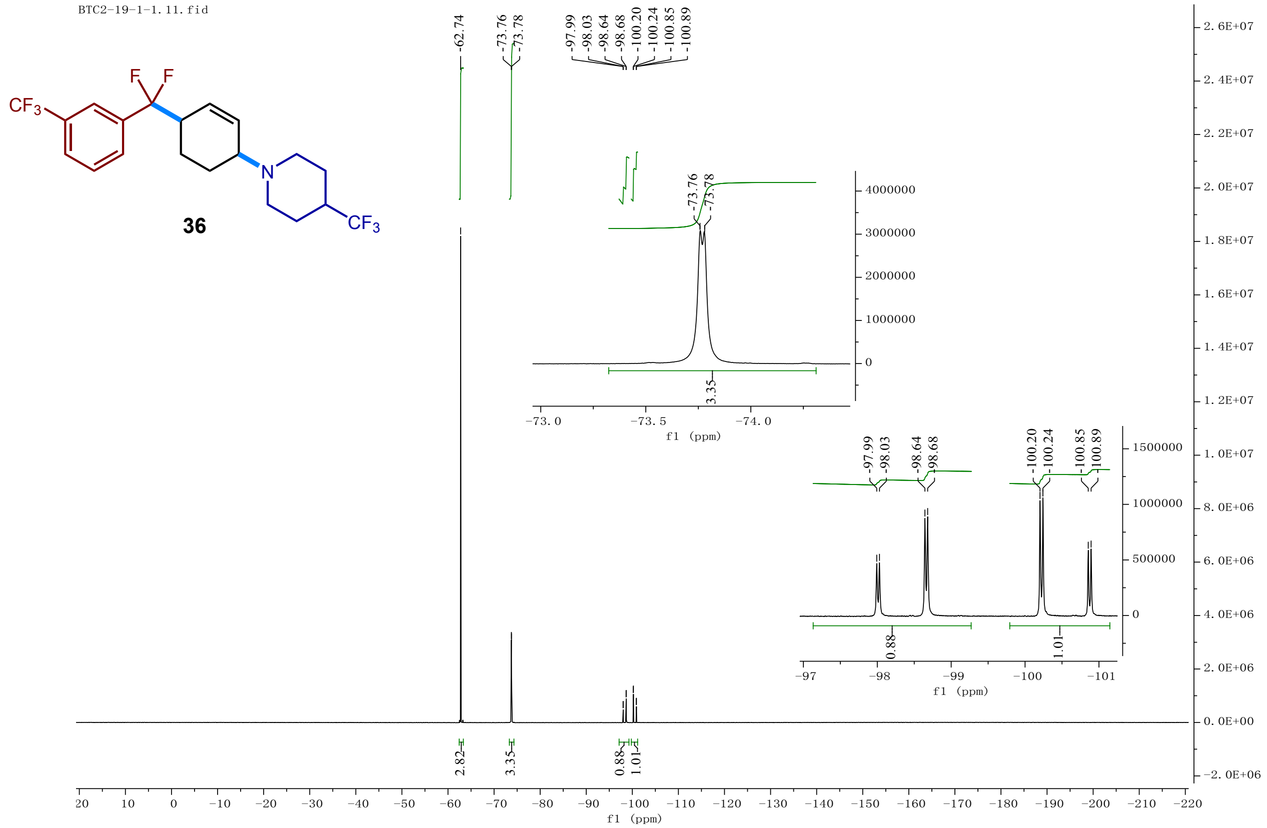
BTC2-19-1-1.11.fid
CF3
F F
N
CF3
36
f1 (ppm)

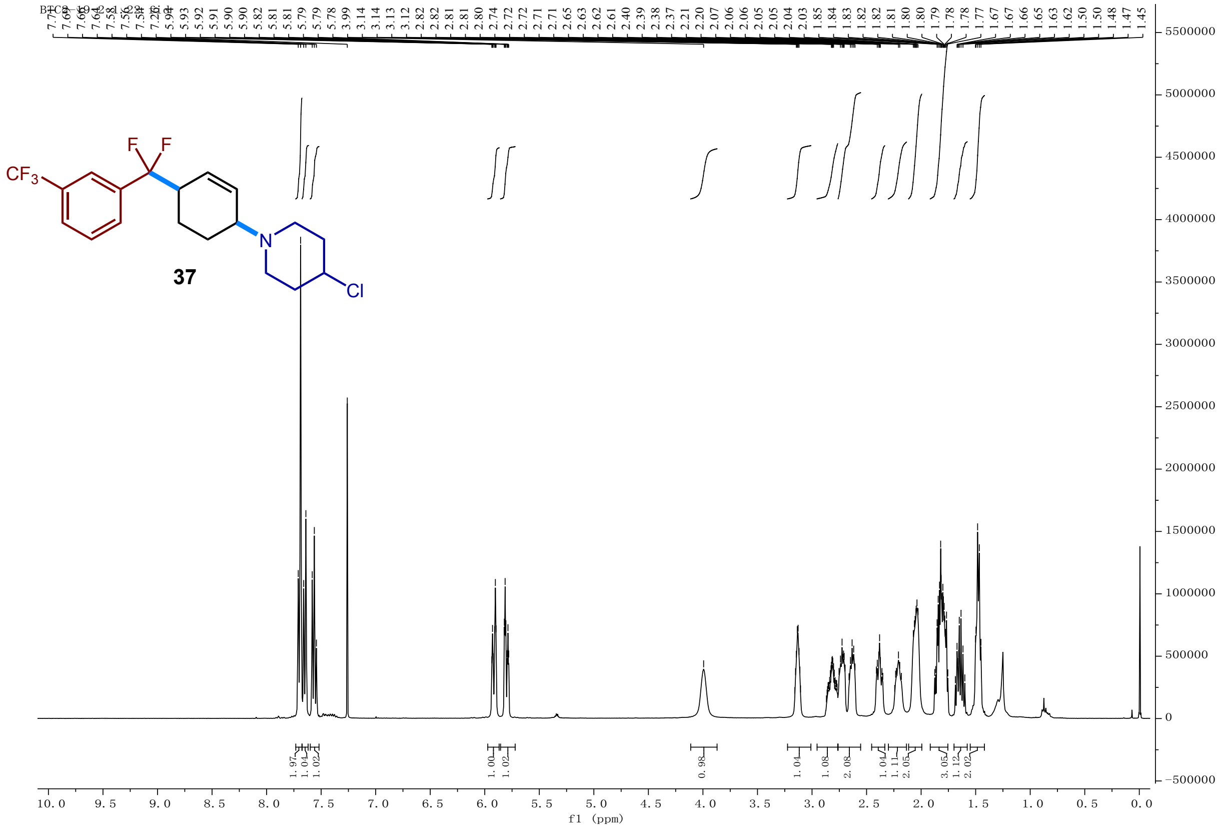

37
f1 (ppm)

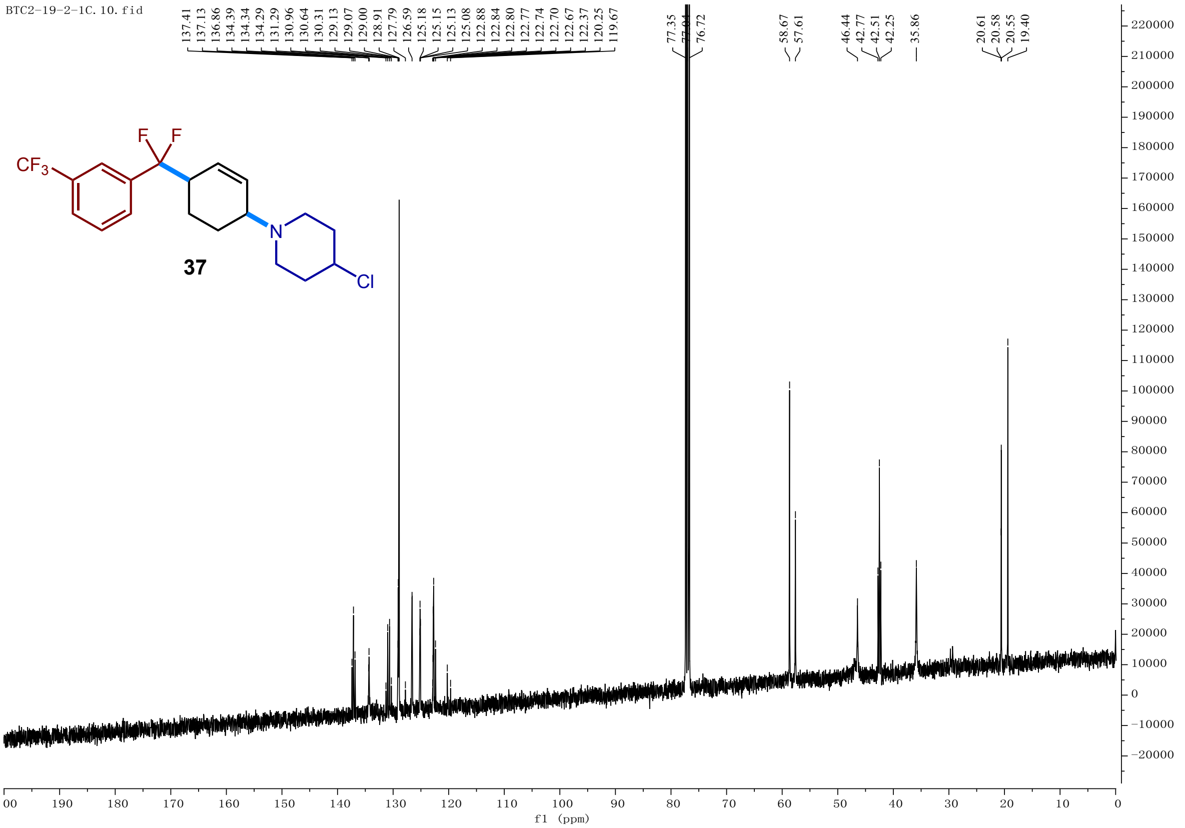

BTC2-19-2-1C.10.fid
37
f1 (ppm)

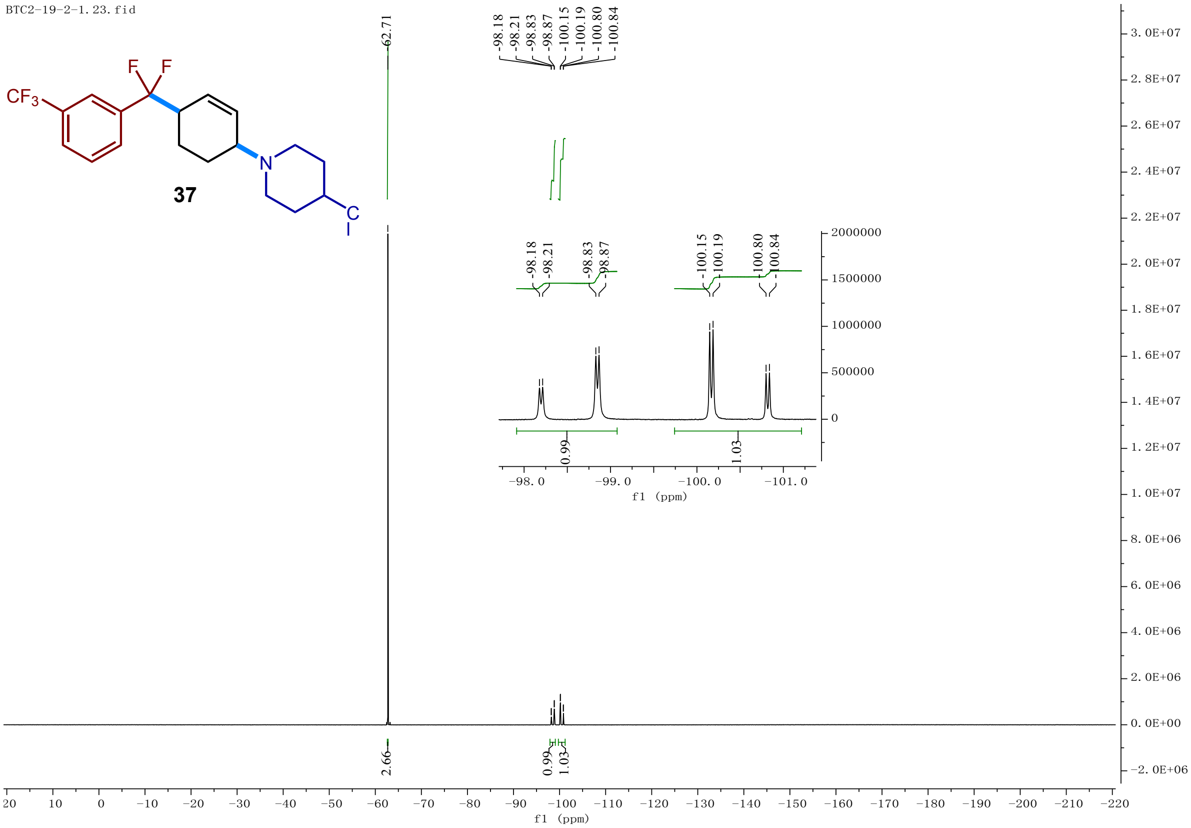
BTC2-19-2-1.23.fid
CF3
F
F
N
37
Cl
-62.71
-98.18
-98.21
-98.83
-98.87
-100.15
-100.19
-100.80
-100.84
0.99
1.03
2.66
f1 (ppm)

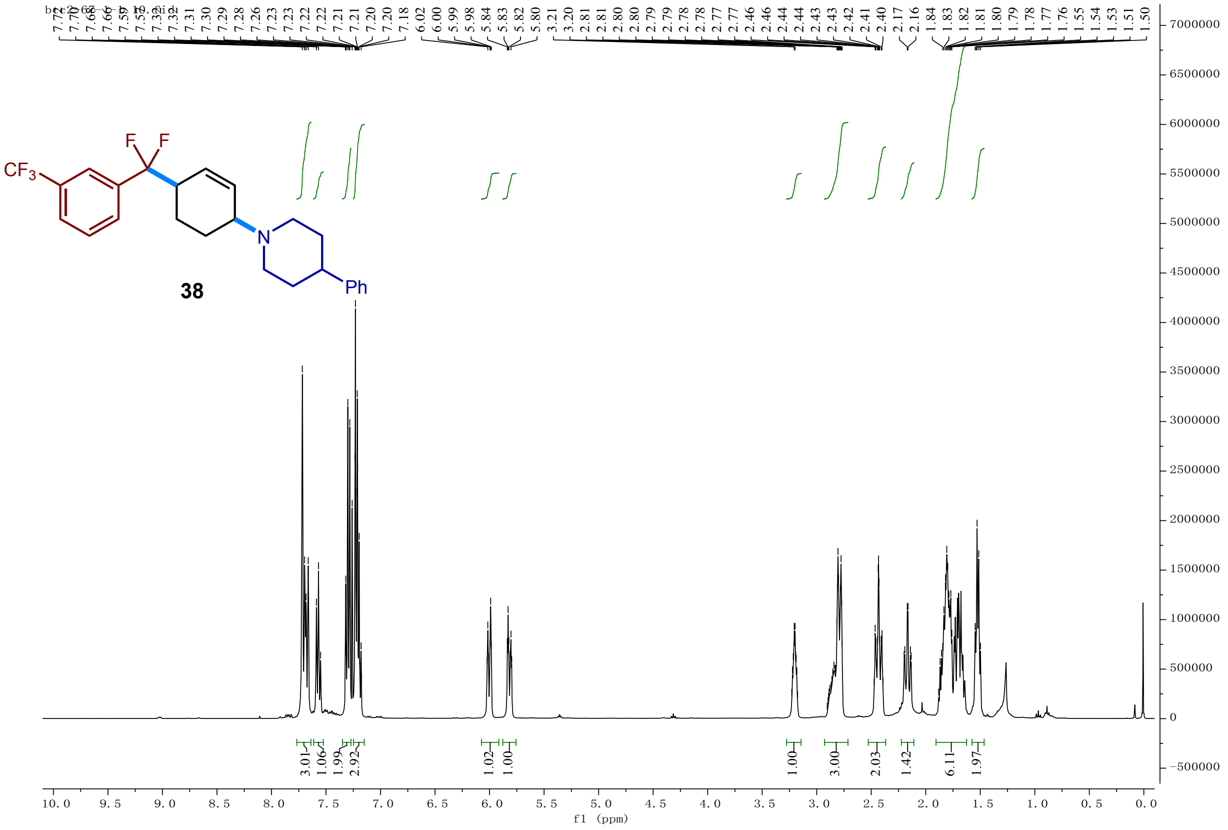
CF3
F
F
N
Ph
38
7.72
7.70
7.68
7.66
7.59
7.58
7.32
7.31
7.30
7.29
7.28
7.26
7.23
7.23
7.22
7.22
7.21
7.21
7.20
7.20
7.18
6.02
6.00
5.99
5.98
5.84
5.83
5.82
5.80
3.21
3.20
2.81
2.81
2.80
2.80
2.79
2.79
2.78
2.78
2.77
2.77
2.46
2.44
2.44
2.43
2.43
2.42
2.41
2.40
2.17
2.16
1.84
1.83
1.82
1.81
1.80
1.79
1.78
1.77
1.76
1.55
1.54
1.53
1.51
1.50
3.01
1.06
1.99
2.92
1.02
1.00
1.00
3.00
2.03
1.42
6.11
1.97
f1 (ppm)

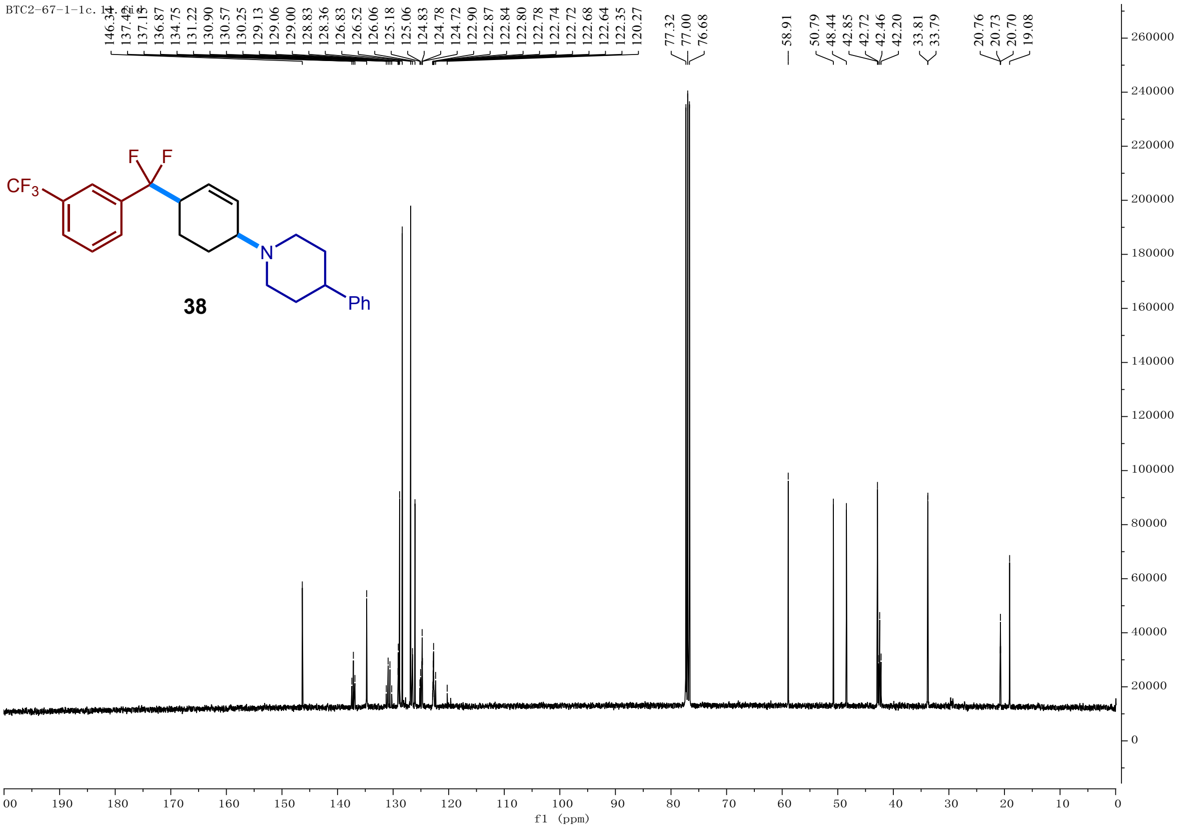
BTC2-67-1-1c.11.fid
146.34
137.42
137.15
136.87
134.75
131.22
130.90
130.57
130.25
129.13
129.06
129.00
128.83
128.36
126.83
126.52
126.06
125.18
125.06
124.83
124.78
124.72
122.90
122.87
122.84
122.80
122.78
122.74
122.72
122.68
122.64
122.35
120.27
77.32
77.00
76.68
58.91
50.79
48.44
42.85
42.72
42.46
42.20
33.81
33.79
20.76
20.73
20.70
19.08
CF3
F
F
N
Ph
38
f1 (ppm)

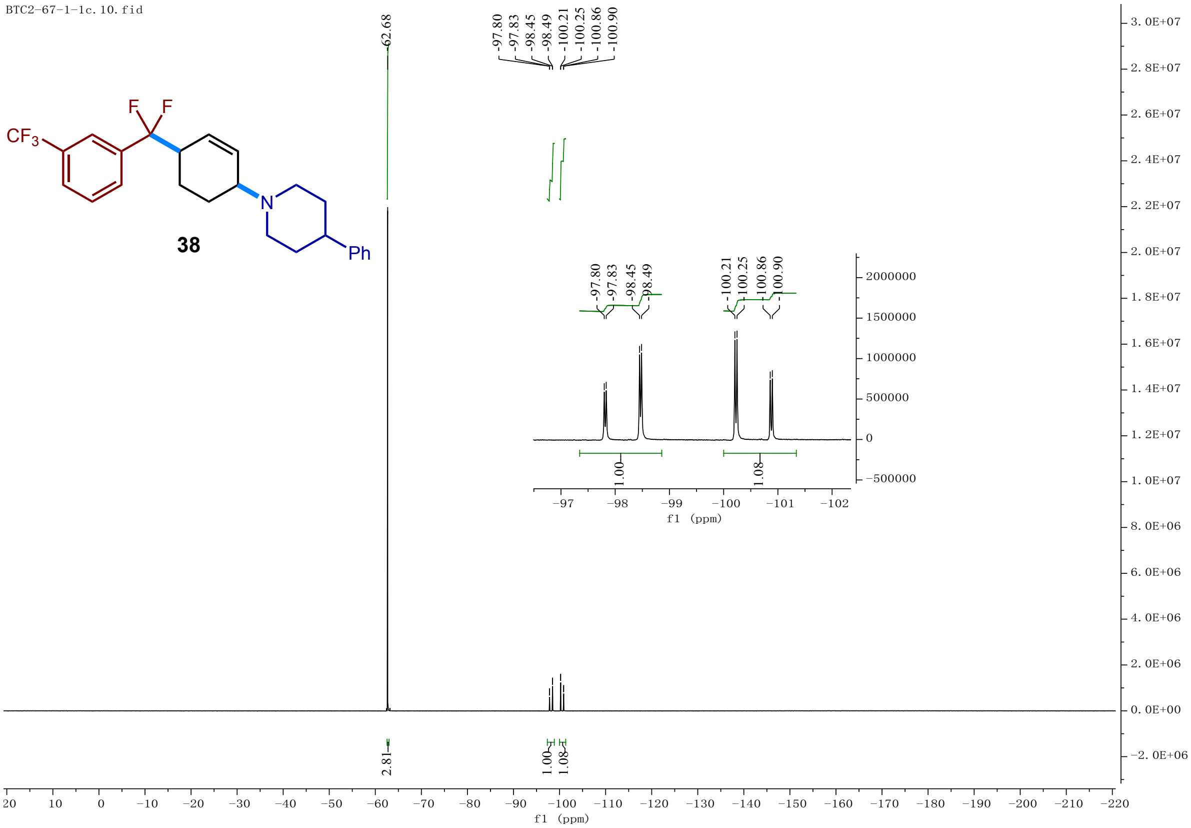
BTC2-67-1-1c.10.fid
-62.68
-97.80
-97.83
-98.45
-98.49
-100.21
-100.25
-100.86
-100.90
CF3
F
F
N
Ph
38
1.00
1.08
2.81
f1 (ppm)

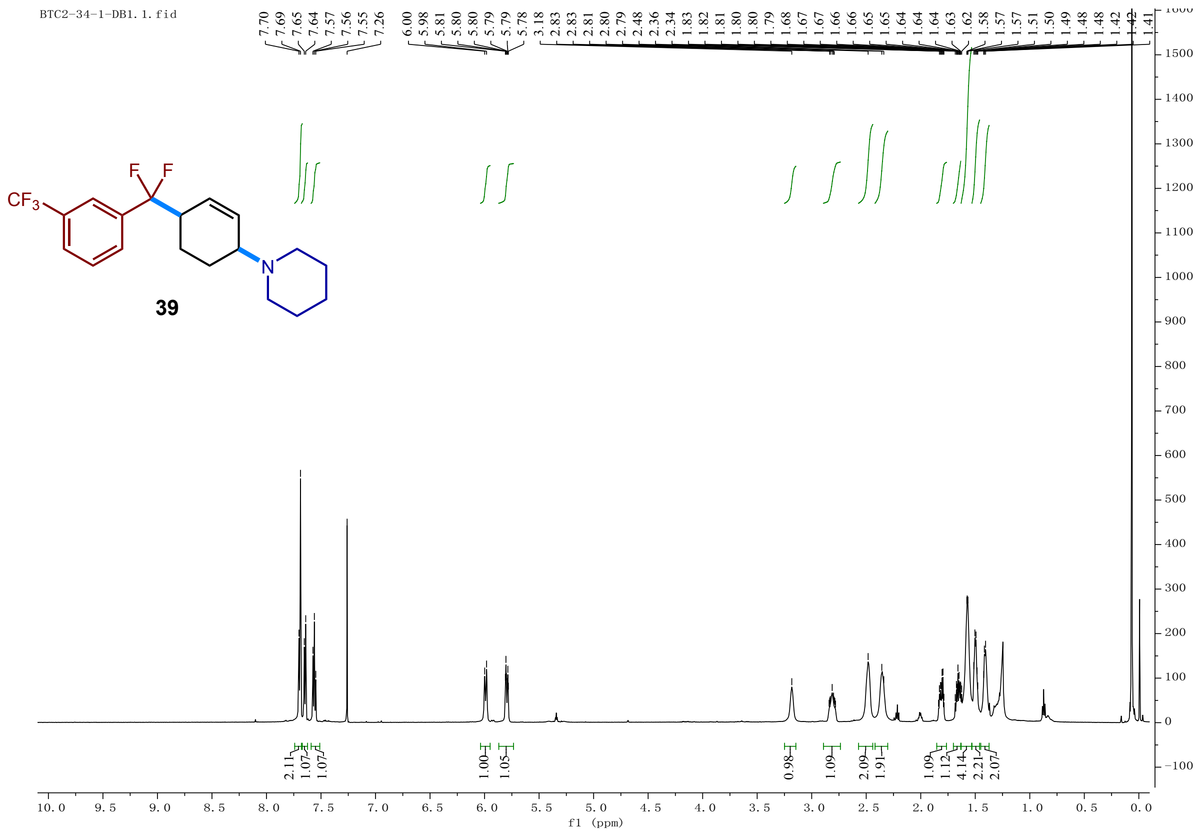
BTC2-34-1-DB1.1.fid
CF3
F F
N
39
f1 (ppm)

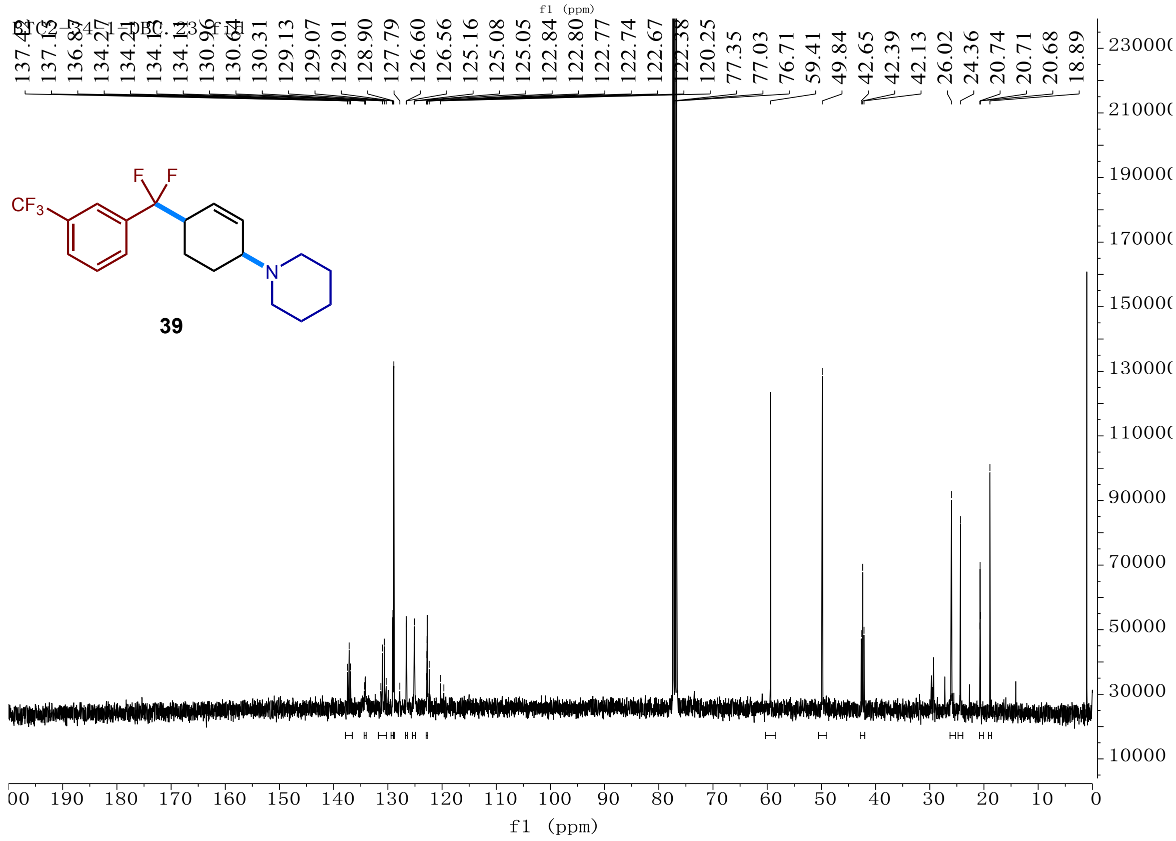
CF3
F F
N
39
f1 (ppm)

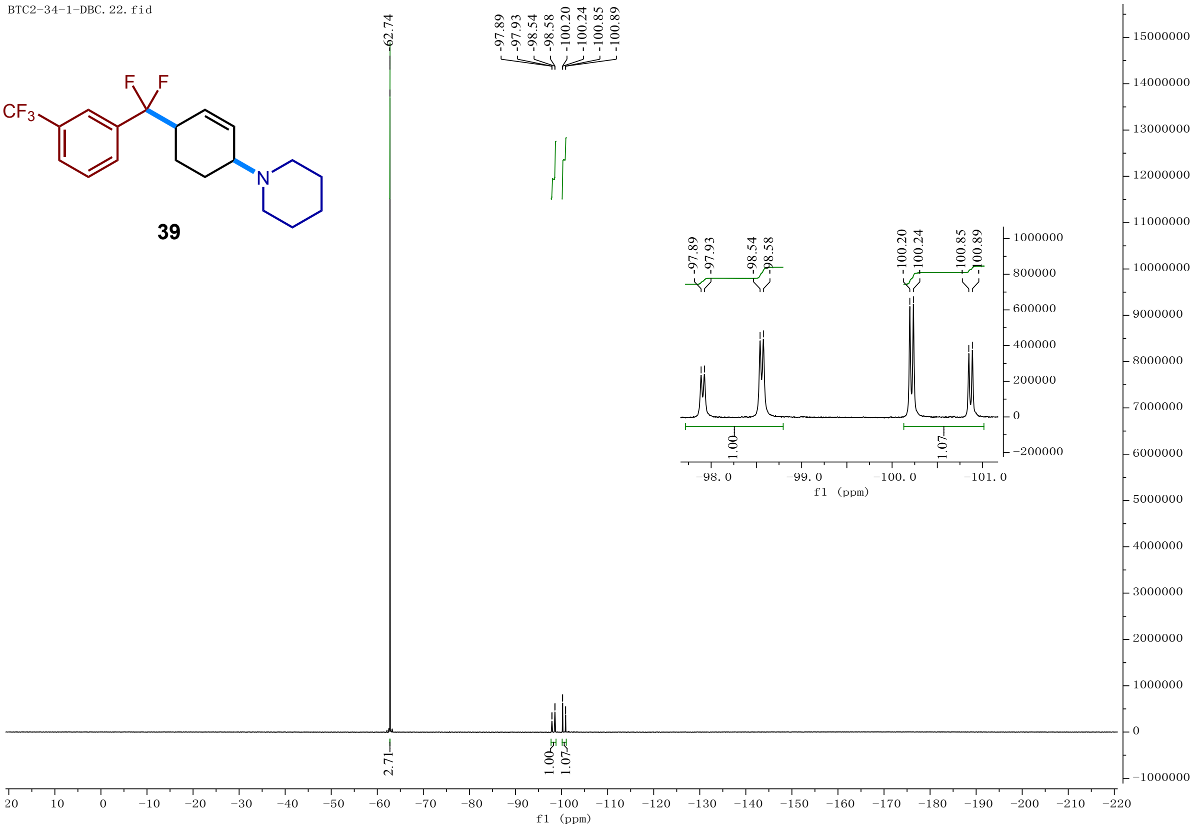
BTC2-34-1-DBC.22.fid
-62.74
-97.89
-97.93
-98.54
-98.58
-100.20
-100.24
-100.85
-100.89
CF3
F F
N
39
2.71
1.00
1.07
f1 (ppm)

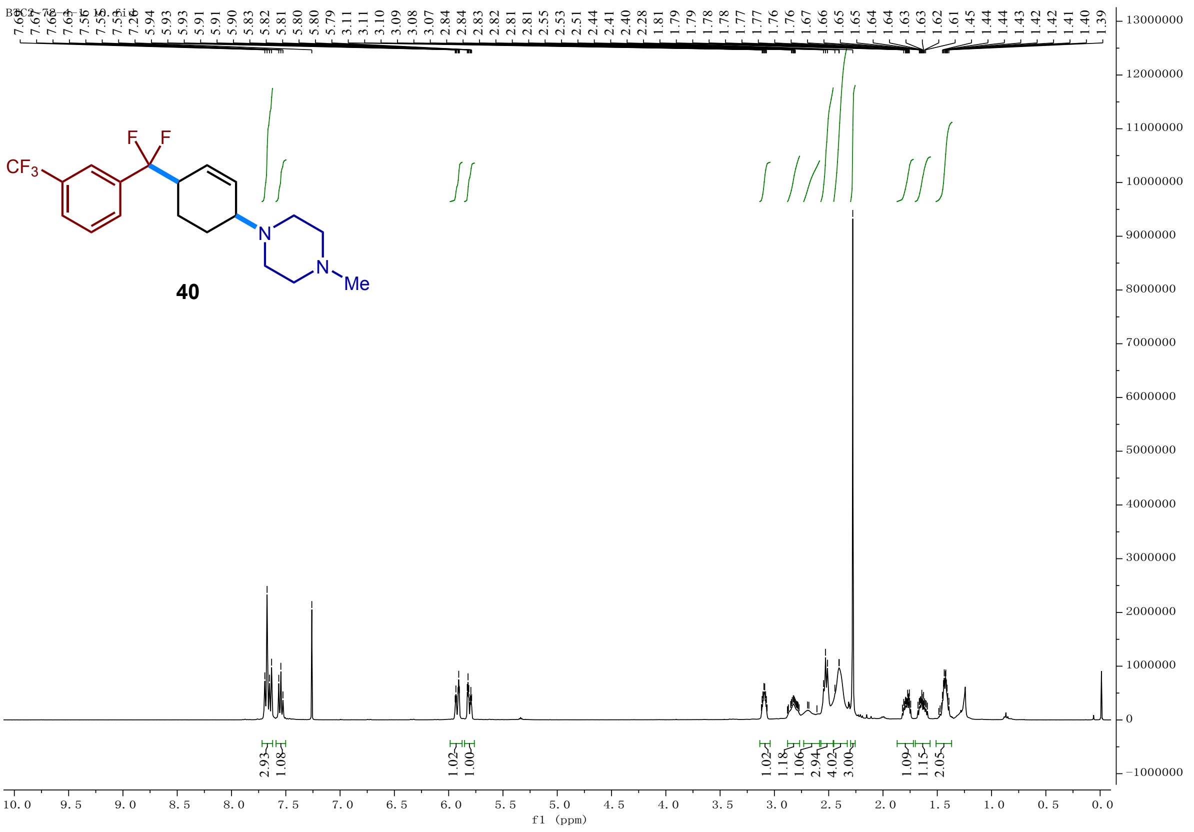
CF3
F F
N
N
Me
40
2.93
1.08
1.02
1.00
1.02
1.18
1.06
2.94
4.02
3.00
1.09
1.15
2.05
f1 (ppm)

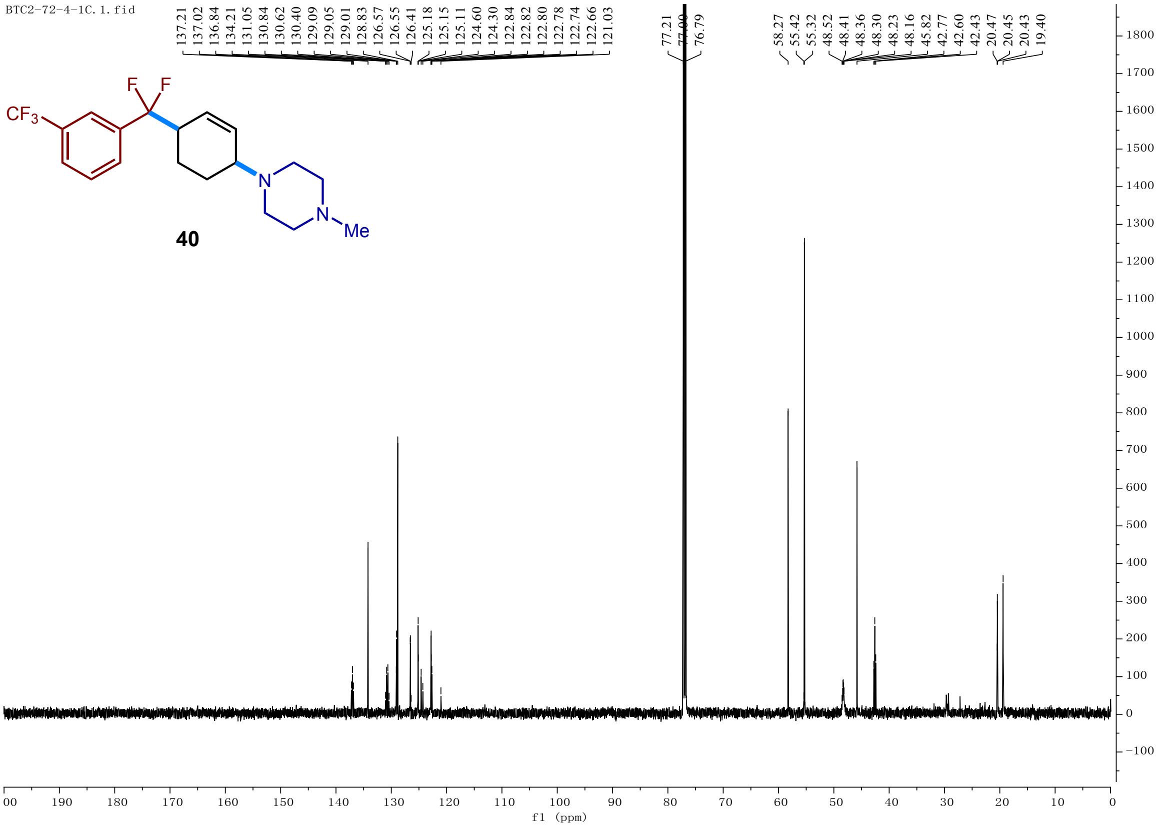

BTC2-72-4-1C.1.fid
137.21
137.02
136.84
134.21
131.05
130.84
130.62
130.40
129.09
129.05
129.01
128.83
126.57
126.55
126.41
125.18
125.15
125.11
124.60
124.30
122.84
122.82
122.80
122.78
122.74
122.66
121.03
77.21
77.00
76.79
58.27
55.42
55.32
48.52
48.41
48.36
48.30
48.23
48.16
45.82
42.77
42.60
42.43
20.47
20.45
20.43
19.40
CF_3
F
F
N
N
Me
40
f1 (ppm)

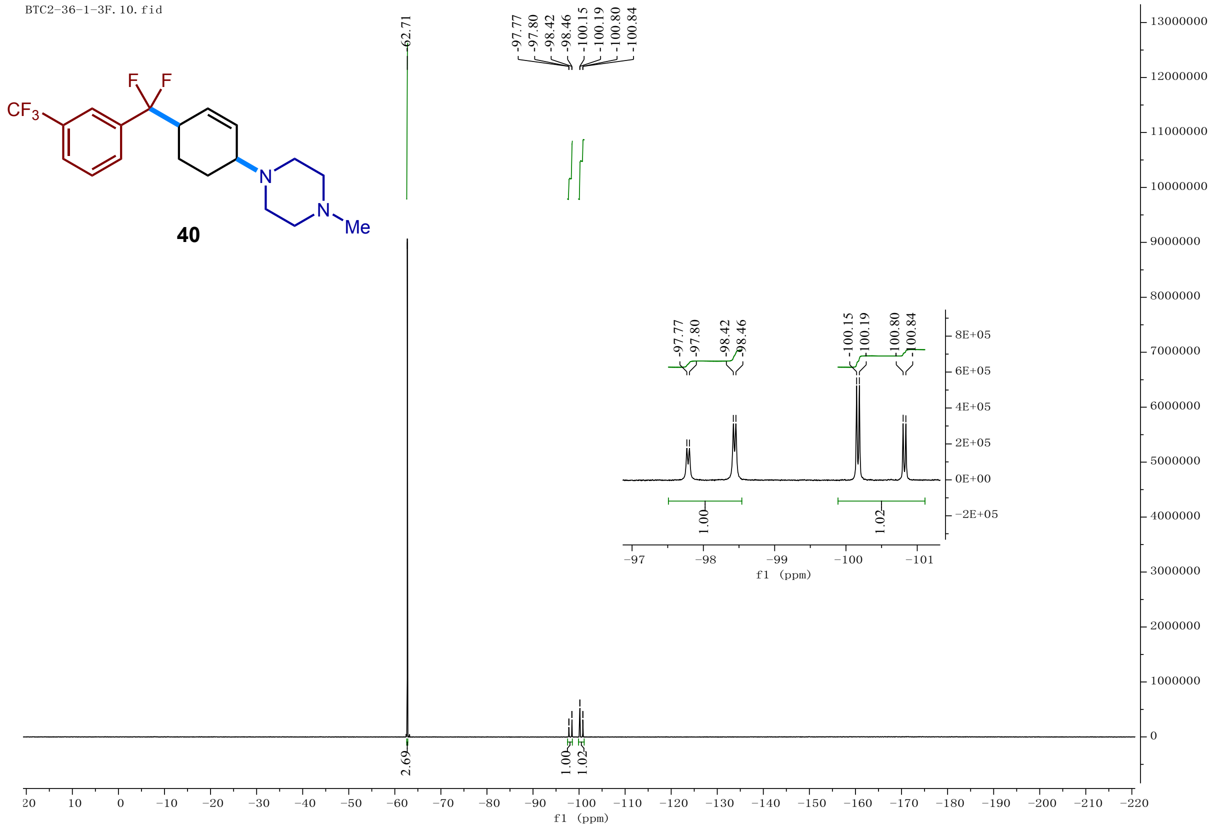

BTC2-36-1-3F.10.fid
-62.71
-97.77
-97.80
-98.42
-98.46
-100.15
-100.19
-100.80
-100.84
CF_3
F
F
N
N
Me
40
2.69
1.00
1.02
f1 (ppm)

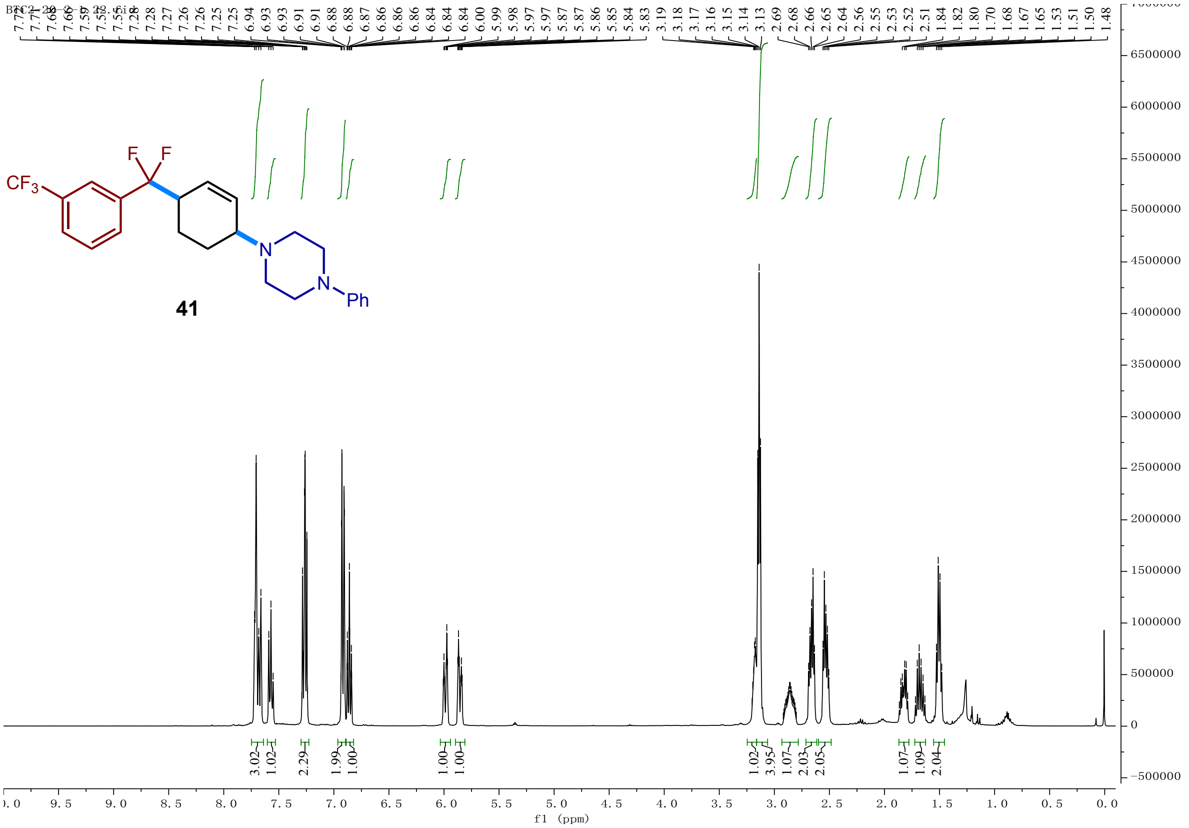

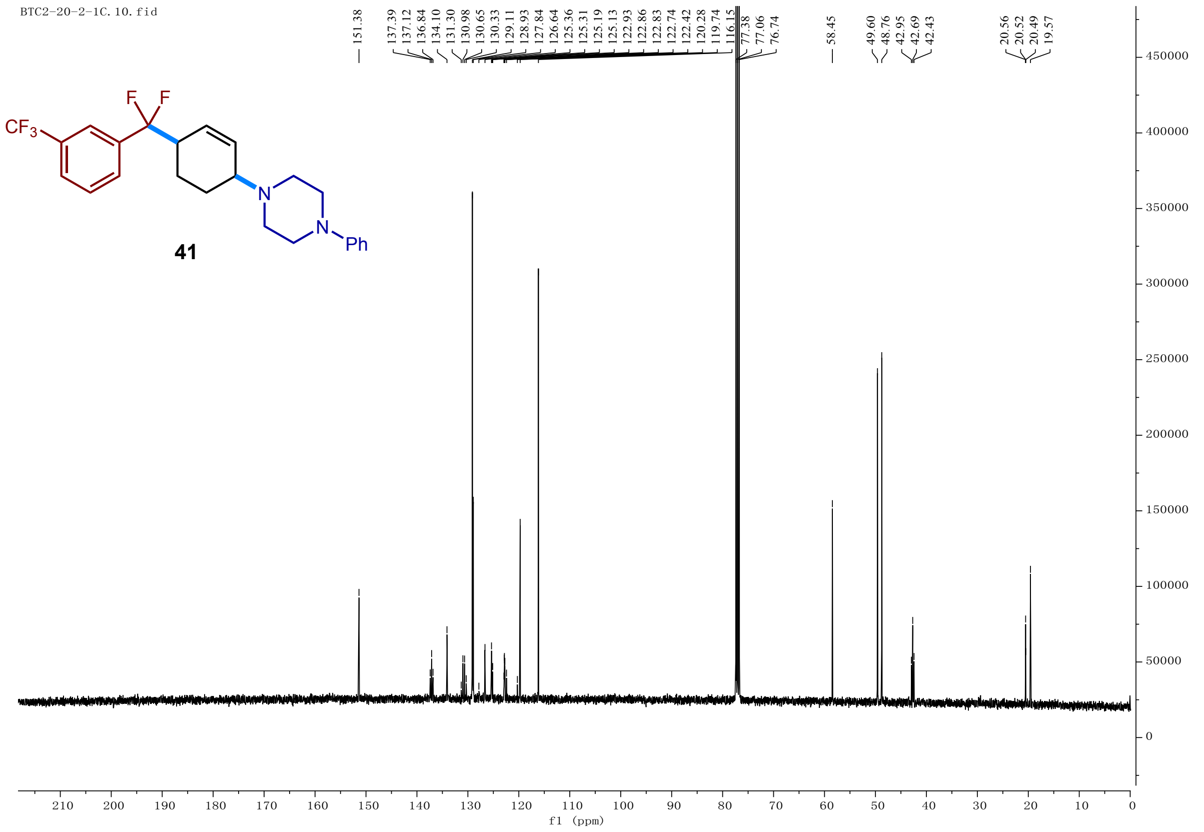

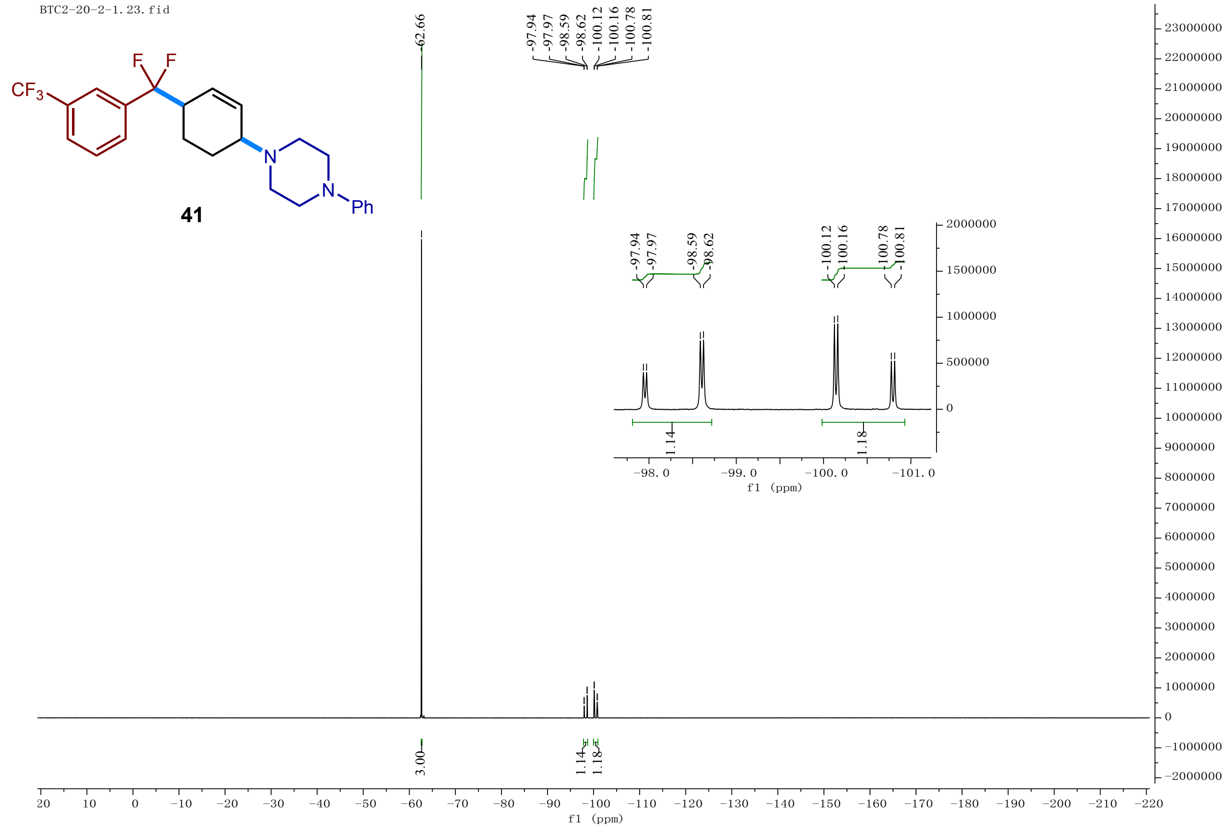
BTC2-20-2-1.23.fid
CF3
F F
N
N
Ph
41
-62.66
-97.94 -97.97 -98.59 -98.62 -100.12 -100.16 -100.78 -100.81
3.00
1.14
1.18
f1 (ppm)

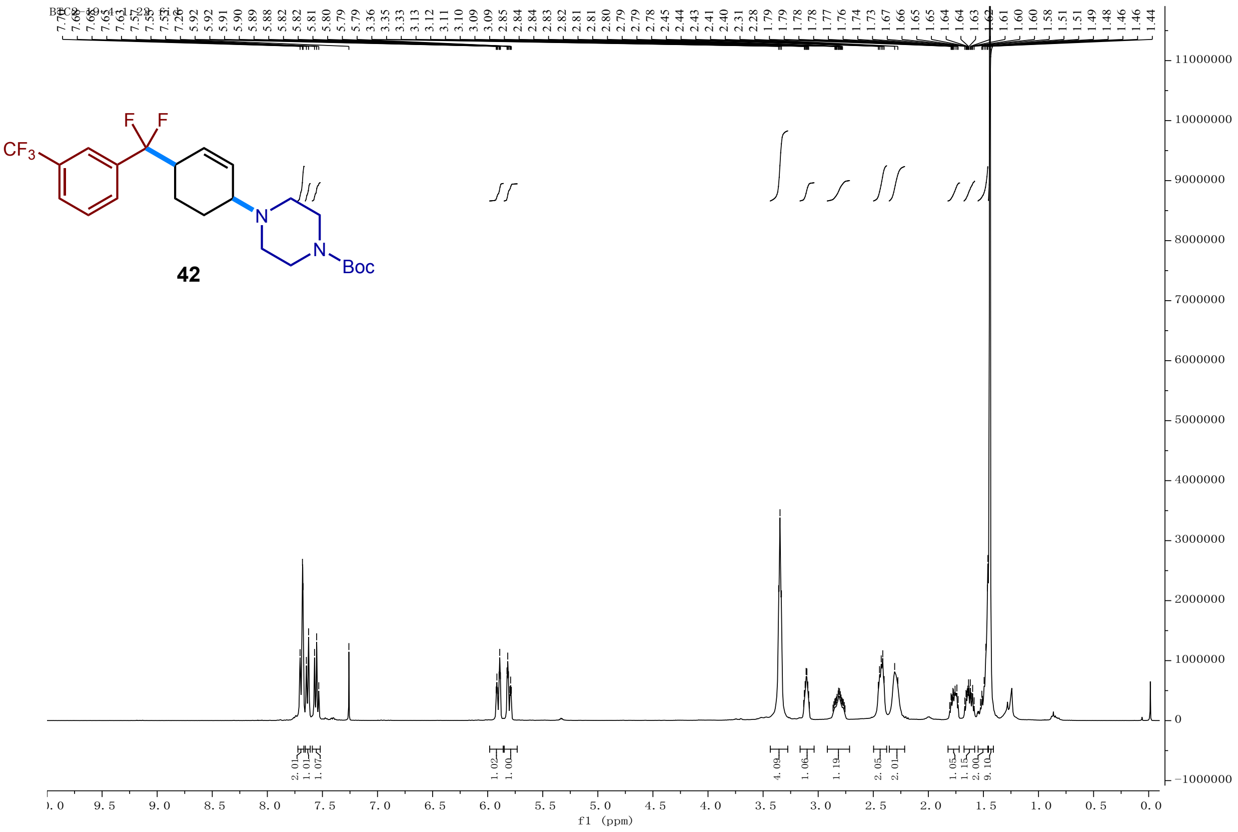
CF3
F F
N
N
Boc
42
2.01 1.01 1.07
1.02 1.00
4.09 1.06 1.19 2.05 2.01 1.05 1.15 2.00 9.10
f1 (ppm)

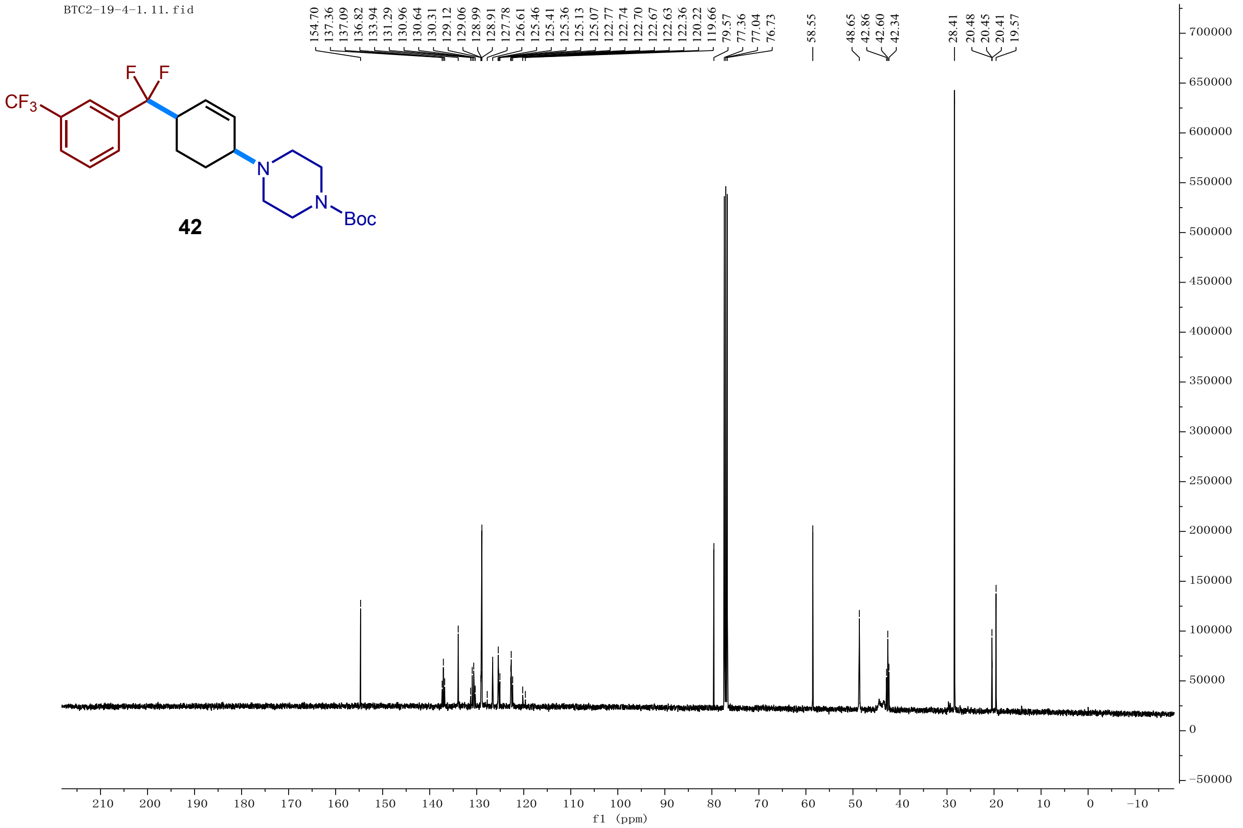
BTC2-19-4-1.11.fid
42
Boc
f1 (ppm)

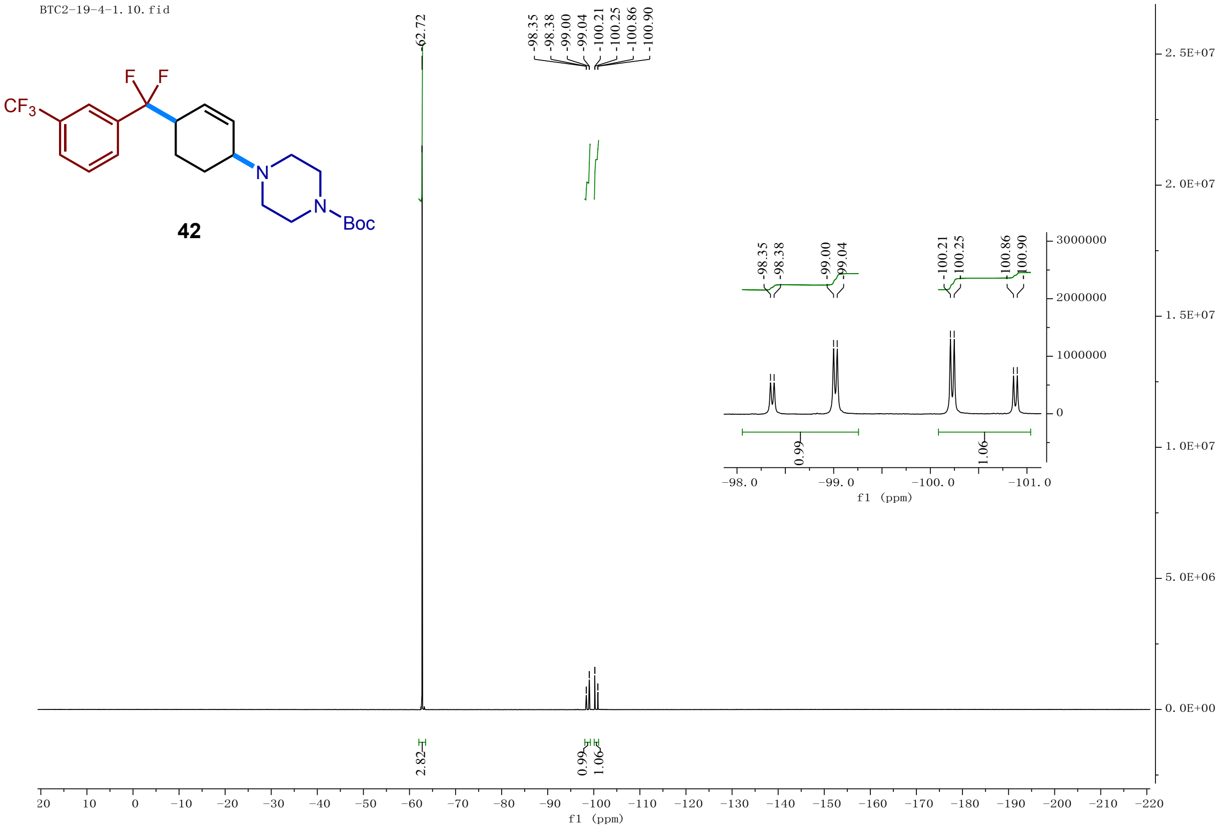
BTC2-19-4-1.10.fid
42
Boc
f1 (ppm)

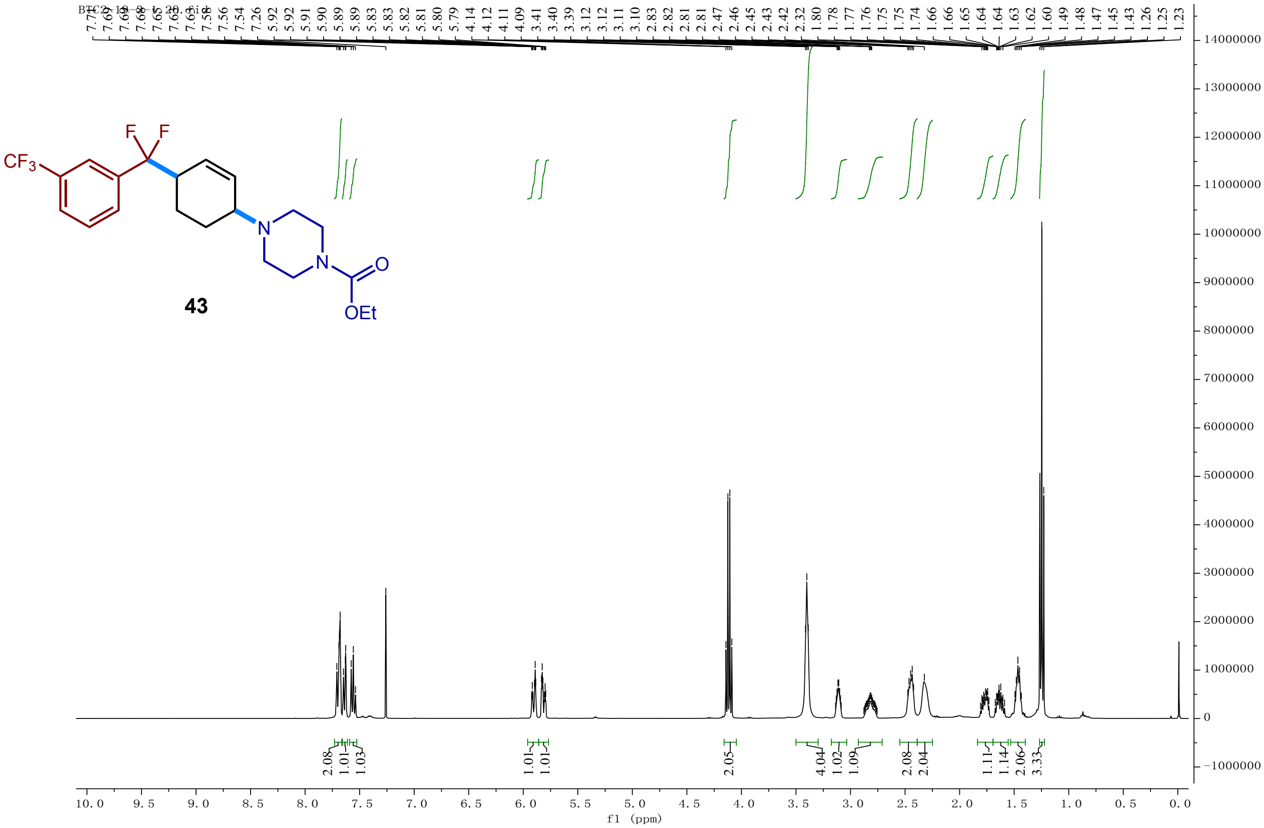
7.71 7.69 7.69 7.68 7.65 7.63 7.63 7.58 7.56 7.54 7.26 5.92 5.92 5.91 5.90 5.89 5.89 5.83 5.83 5.82 5.81 5.80 5.79 4.14 4.12 4.11 4.09 3.41 3.40 3.39 3.12 3.12 3.11 3.10 2.83 2.82 2.81 2.81 2.47 2.46 2.45 2.43 2.42 2.32 1.80 1.78 1.77 1.76 1.75 1.74 1.66 1.66 1.65 1.64 1.64 1.63 1.62 1.60 1.49 1.48 1.47 1.45 1.43 1.26 1.25 1.23
CF3
F F
N
N
O
OEt
43
2.08 1.01 1.03 1.01 1.01 2.05 4.04 1.02 1.09 2.08 2.04 1.11 1.14 2.06 3.33
f1 (ppm)

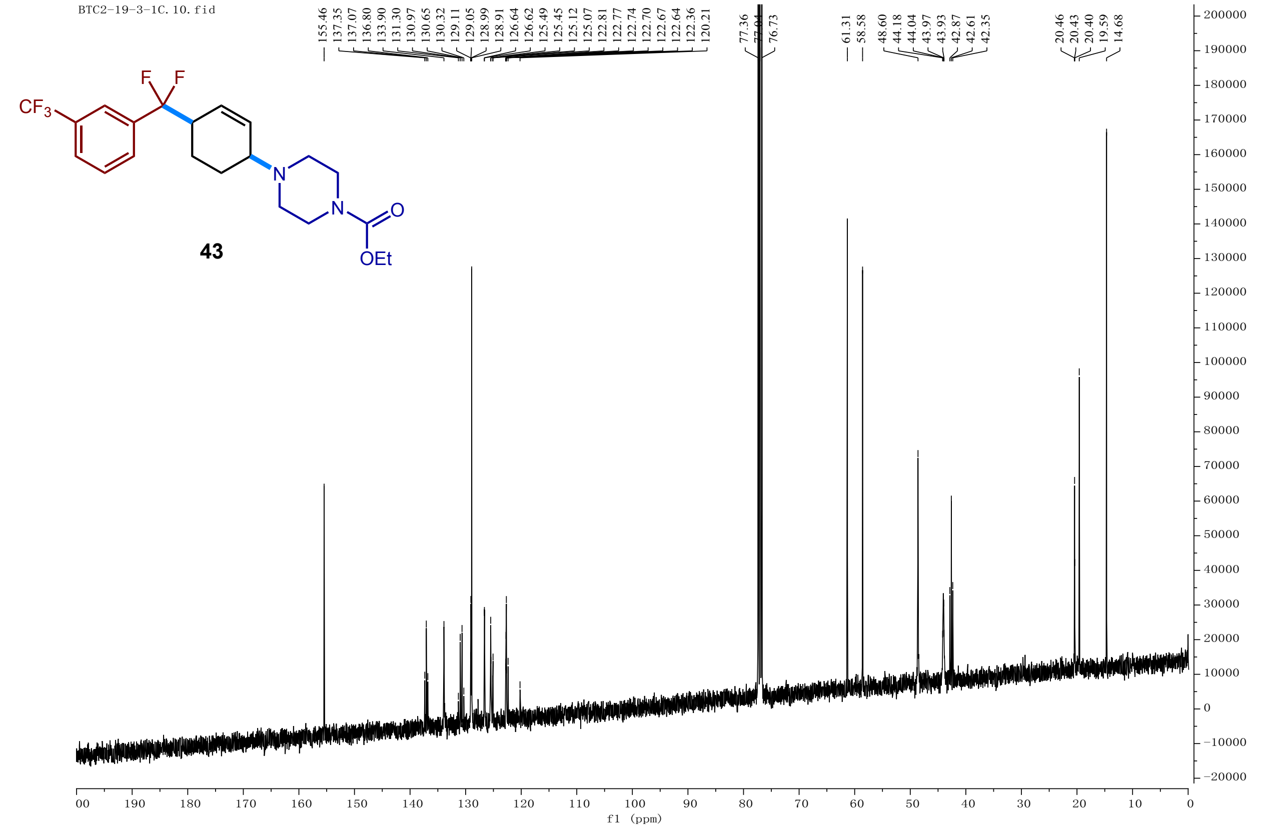
BTC2-19-3-1C.10.fid
155.46 137.35 137.07 136.80 133.90 131.30 130.97 130.65 130.32 129.11 129.05 128.99 128.91 126.64 126.62 125.49 125.45 125.12 125.07 122.81 122.77 122.74 122.70 122.67 122.64 122.36 120.21 77.36 76.73 61.31 58.58 48.60 44.18 44.04 43.97 43.93 42.87 42.61 42.35 20.46 20.43 20.40 19.59 14.68
CF3
F F
N
N
O
OEt
43
f1 (ppm)

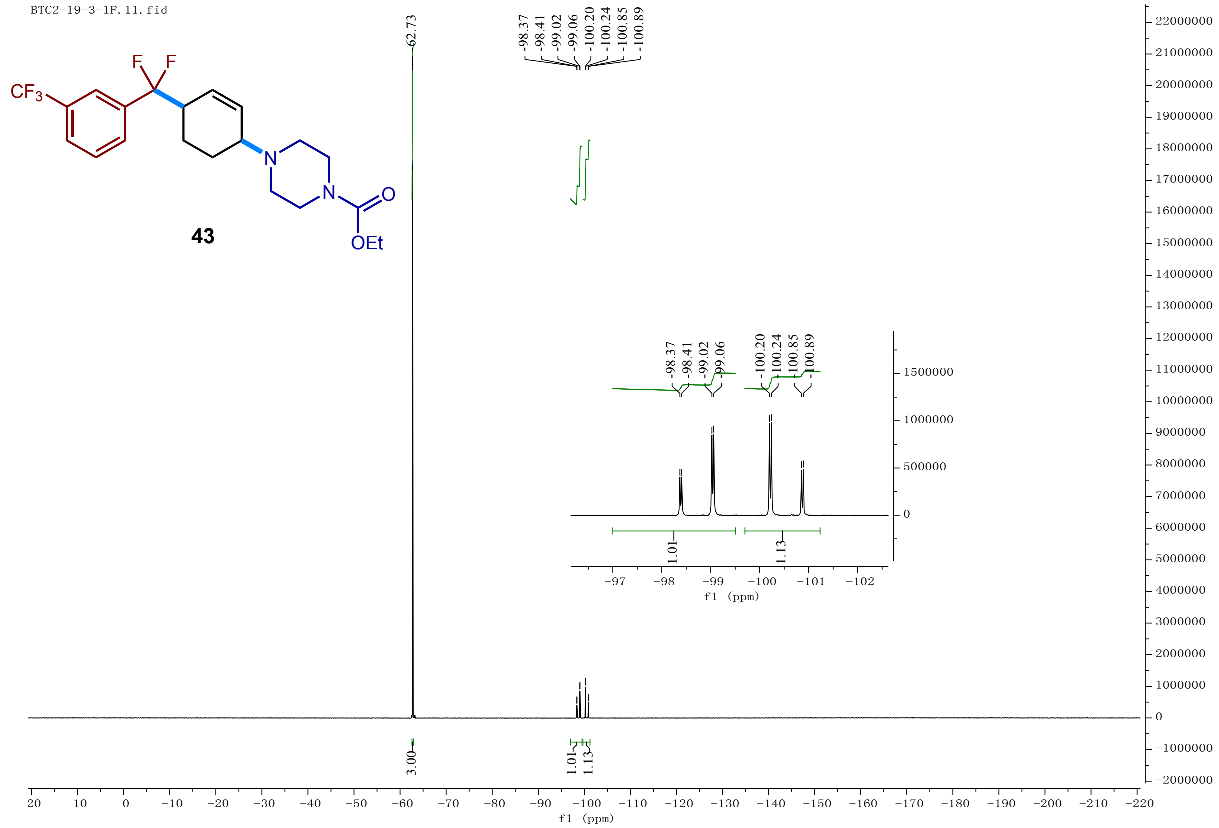
BTC2-19-3-1F. 11. fid
CF3
F F
N
N
O
OEt
43
-62.73
-98.37 -98.41 -99.02 -99.06 -100.20 -100.24 -100.85 -100.89
1.01 1.13
3.00
f1 (ppm)

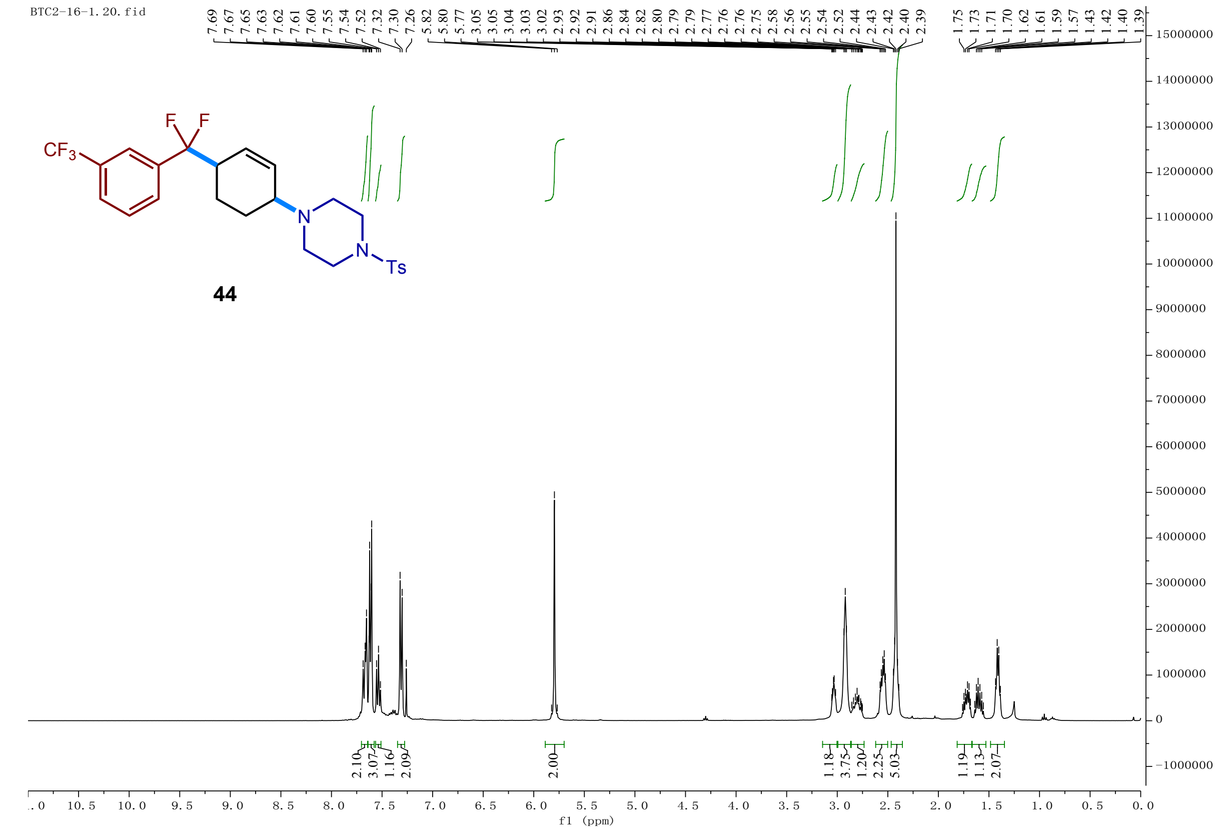
BTC2-16-1. 20. fid
CF3
F F
N
N
Ts
44
7.69 7.67 7.65 7.63 7.62 7.61 7.60 7.55 7.54 7.52 7.32 7.30 7.26 5.82 5.80 5.77 3.05 3.05 3.04 3.03 3.02 2.93 2.92 2.91 2.86 2.84 2.82 2.80 2.79 2.79 2.77 2.76 2.76 2.75 2.58 2.56 2.55 2.54 2.52 2.44 2.43 2.42 2.40 2.39 1.75 1.73 1.71 1.70 1.62 1.61 1.59 1.57 1.43 1.42 1.40 1.39
2.10 3.07 1.16 2.09 2.00 1.18 3.75 1.20 2.25 5.03 1.19 1.13 2.07
f1 (ppm)

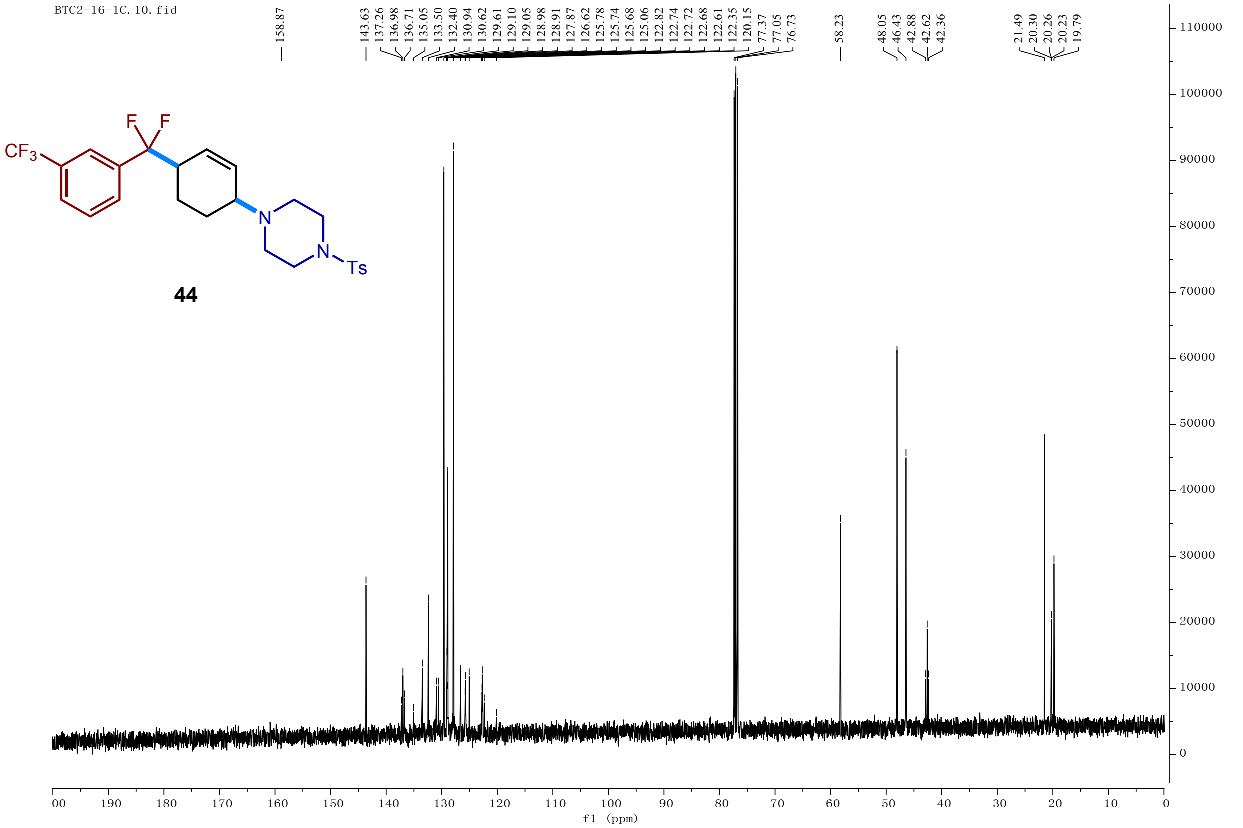
BTC2-16-1C.10.fid
158.87
143.63
137.26
136.98
136.71
135.05
133.50
132.40
130.94
130.62
129.61
129.10
129.05
128.98
128.91
127.87
126.62
125.78
125.74
125.68
125.06
122.82
122.74
122.72
122.68
122.61
122.35
120.15
77.37
77.05
76.73
58.23
48.05
46.43
42.88
42.62
42.36
21.49
20.30
20.26
20.23
19.79
CF3
F
N
Ts
44
f1 (ppm)

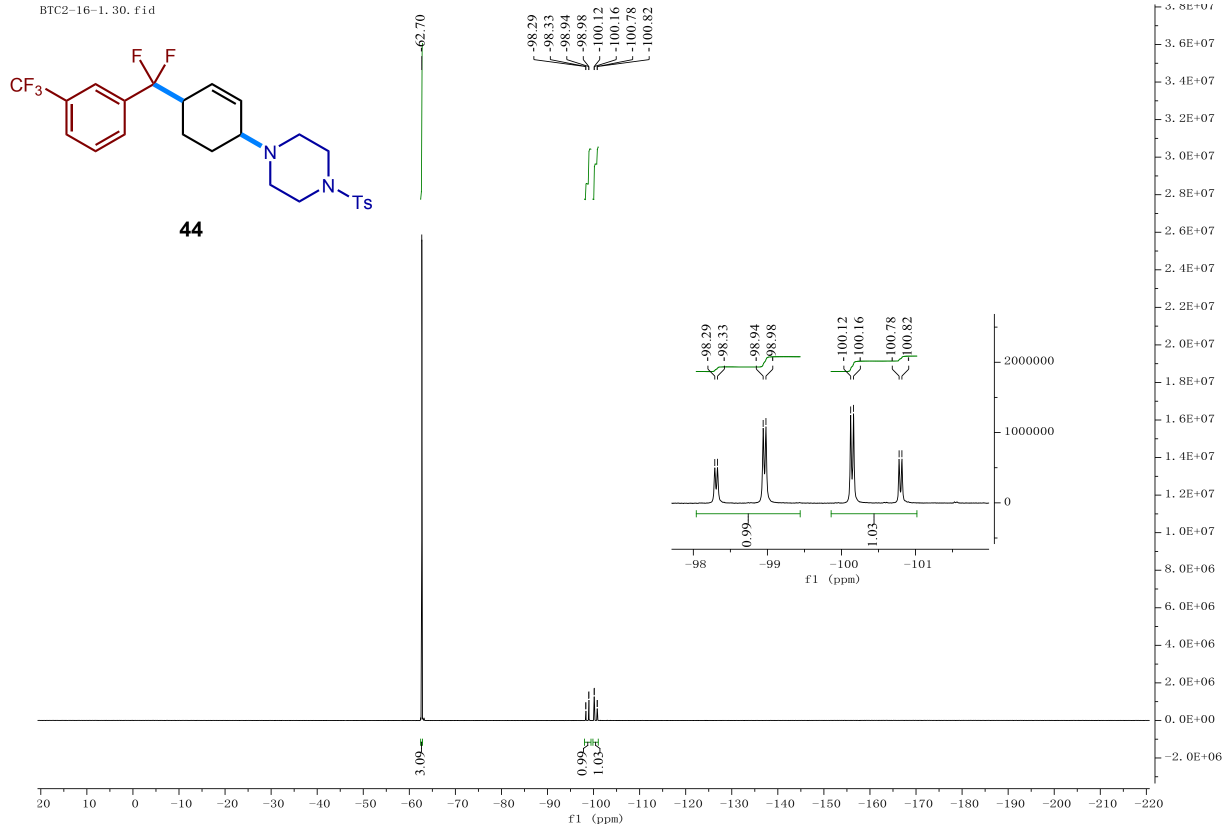
BTC2-16-1.30.fid
-62.70
-98.29
-98.33
-98.94
-98.98
-100.12
-100.16
-100.78
-100.82
CF3
F
N
Ts
44
3.09
0.99
1.03
f1 (ppm)

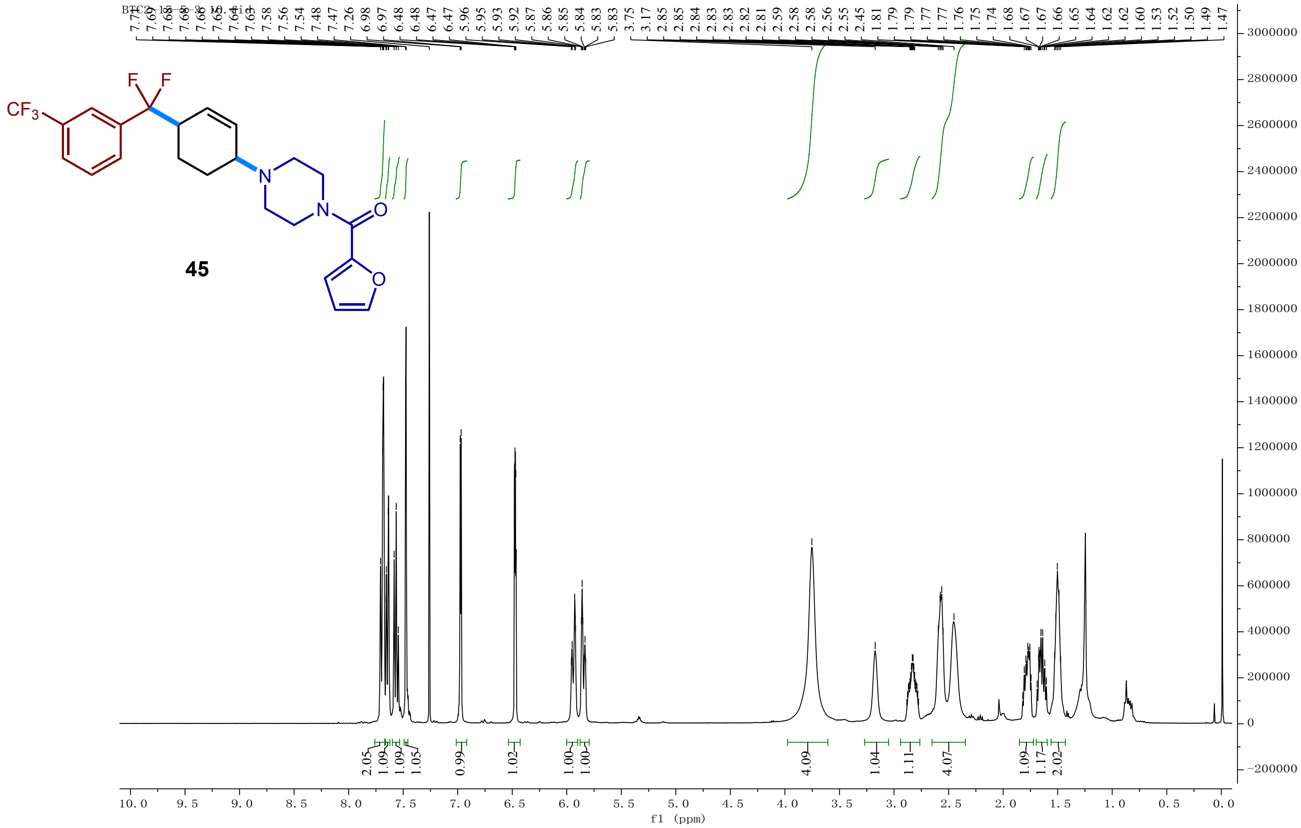
CF3
F F
N
N
O
O
45
f1 (ppm)

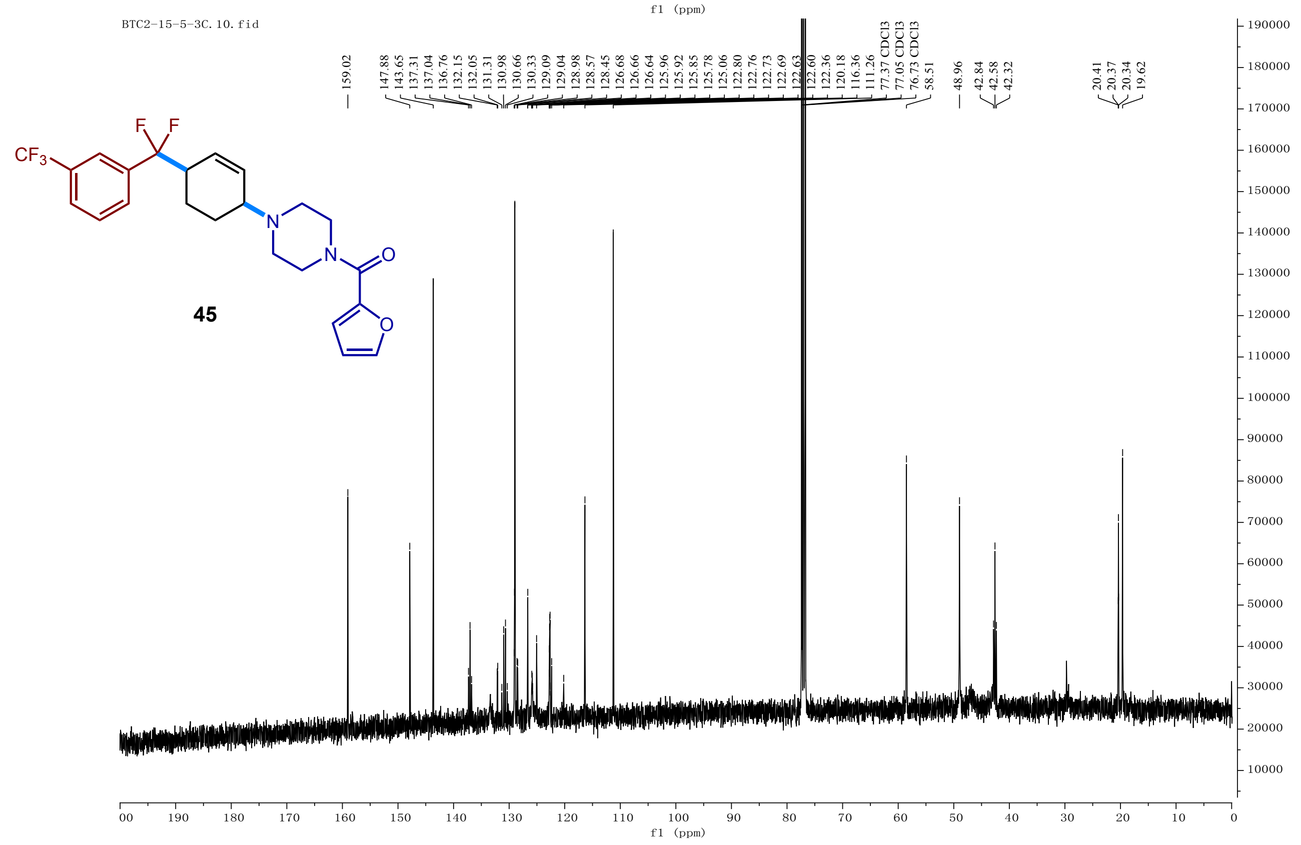
BTC2-15-5-3C.10.fid
159.02
147.88
143.65
137.31
137.04
136.76
132.15
132.05
131.31
130.98
130.66
130.33
129.09
129.04
128.98
128.57
128.45
126.68
126.66
126.64
125.96
125.92
125.85
125.78
125.06
122.80
122.76
122.73
122.69
122.63
122.60
122.36
120.18
116.36
111.26
77.37 CDCl3
77.05 CDCl3
76.73 CDCl3
58.51
48.96
42.84
42.58
42.32
20.41
20.37
20.34
19.62
CF3
F F
N
N
O
O
45
f1 (ppm)

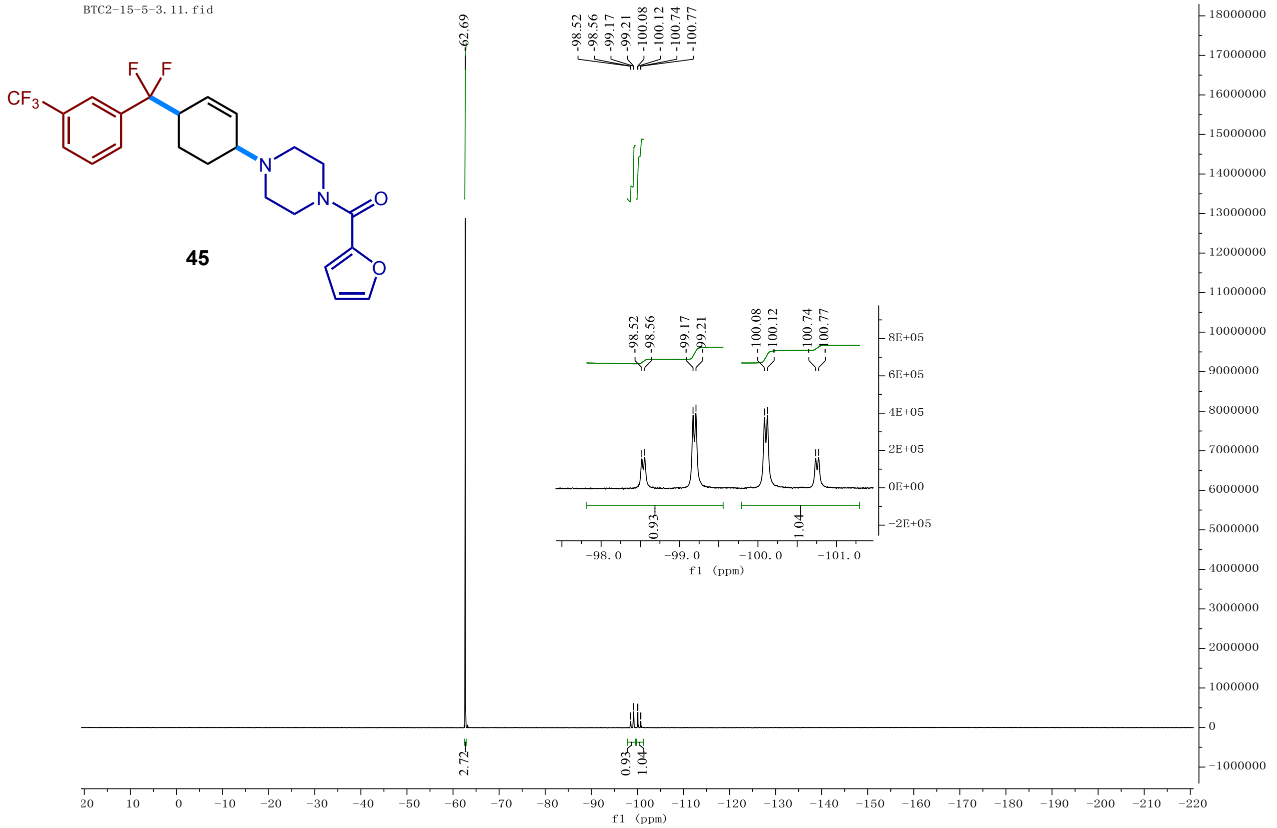
BTC2-15-5-3.11.fid
CF3
F F
N
N
O
O
45
-62.69
-98.52 -98.56 -99.17 -99.21 -100.08 -100.12 -100.74 -100.77
0.93
1.04
2.72
f1 (ppm)

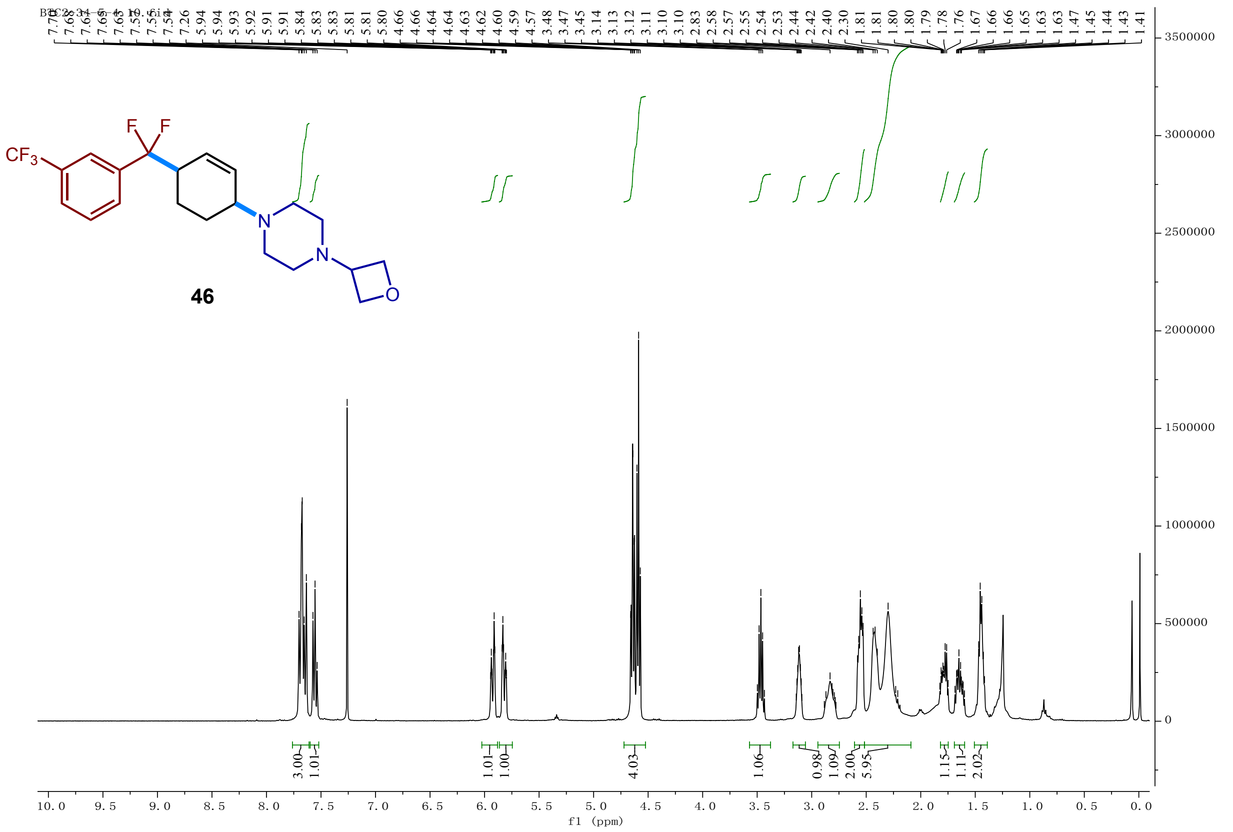
7.70 7.68 7.65 7.65 7.63 7.55 7.54 7.26 5.94 5.94 5.93 5.92 5.91 5.91 5.84 5.83 5.83 5.81 5.81 5.80 4.66 4.66 4.64 4.64 4.63 4.62 4.60 4.59 4.57 3.48 3.47 3.45 3.14 3.13 3.12 3.11 3.10 3.10 2.83 2.58 2.57 2.55 2.54 2.53 2.44 2.42 2.40 2.30 1.81 1.81 1.80 1.80 1.79 1.78 1.76 1.67 1.66 1.66 1.65 1.63 1.63 1.47 1.45 1.44 1.43 1.41
CF3
F F
N
N
O
46
3.00 1.01 1.01 1.00 4.03 1.06 0.98 1.09 2.00 5.95 1.15 1.11 2.02
f1 (ppm)

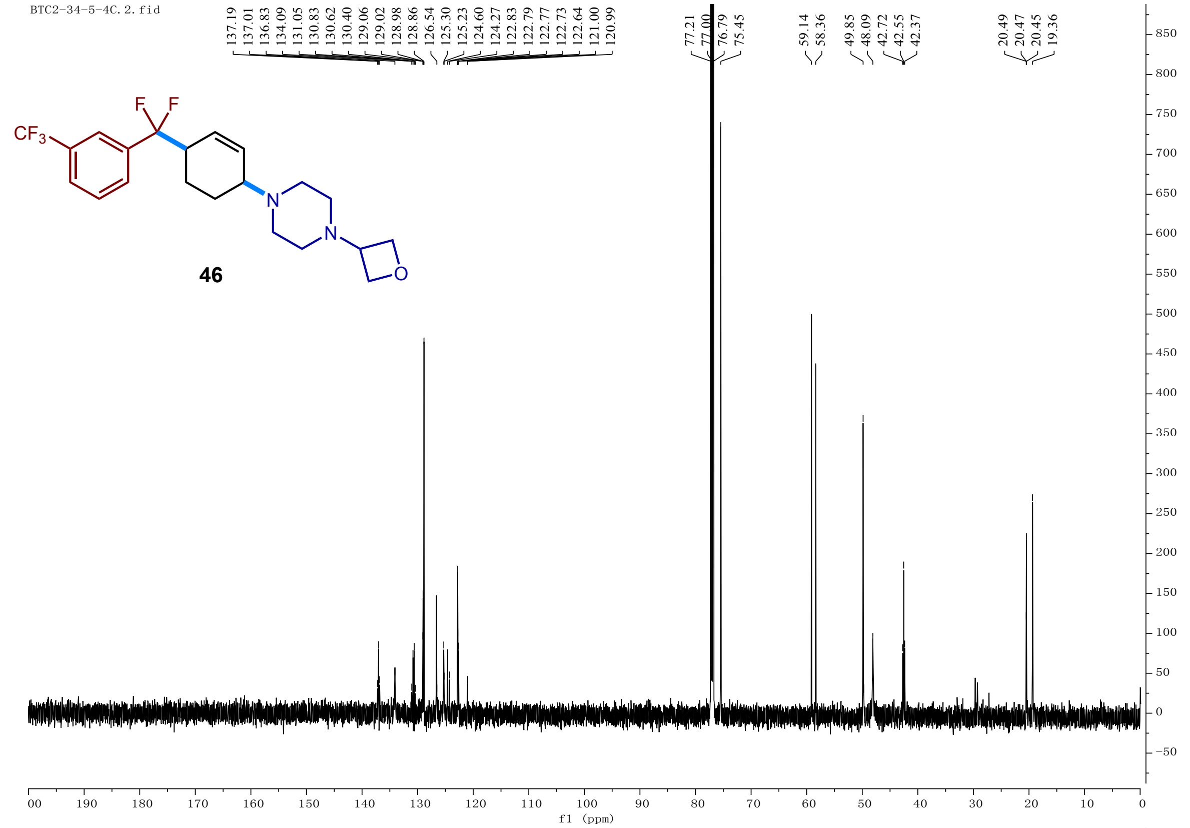
BTC2-34-5-4C.2.fid
137.19 137.01 136.83 134.09 131.05 130.83 130.62 130.40 129.06 129.02 128.98 128.86 126.54 125.30 125.23 124.60 124.27 122.83 122.79 122.77 122.73 122.64 121.00 120.99
77.21 77.00 76.79 75.45
59.14 58.36
49.85 48.09 42.72 42.55 42.37
20.49 20.47 20.45 19.36
CF3
F F
N
N
O
46
f1 (ppm)

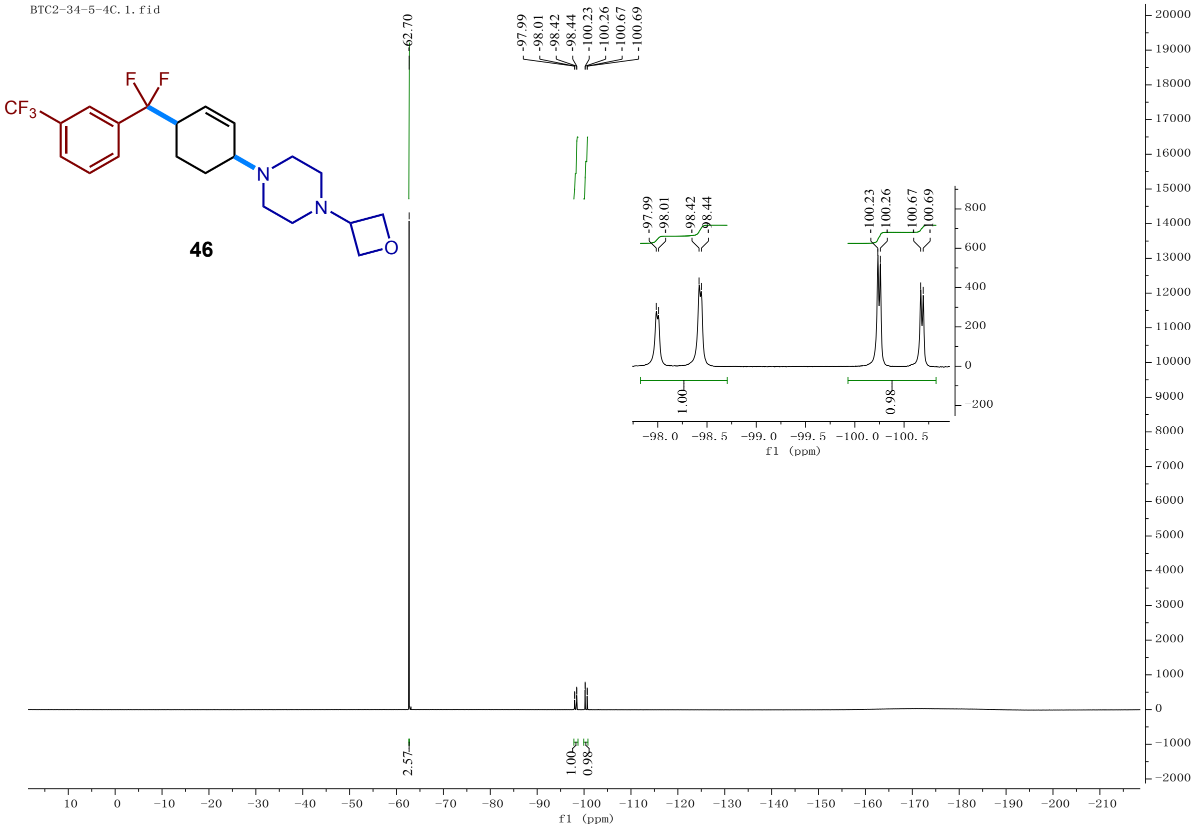
BTC2-34-5-4C.1.fid
-62.70
-97.99 -98.01 -98.42 -98.44 -100.23 -100.26 -100.67 -100.69
CF3
F F
N
N
O
46
1.00
0.98
2.57
f1 (ppm)

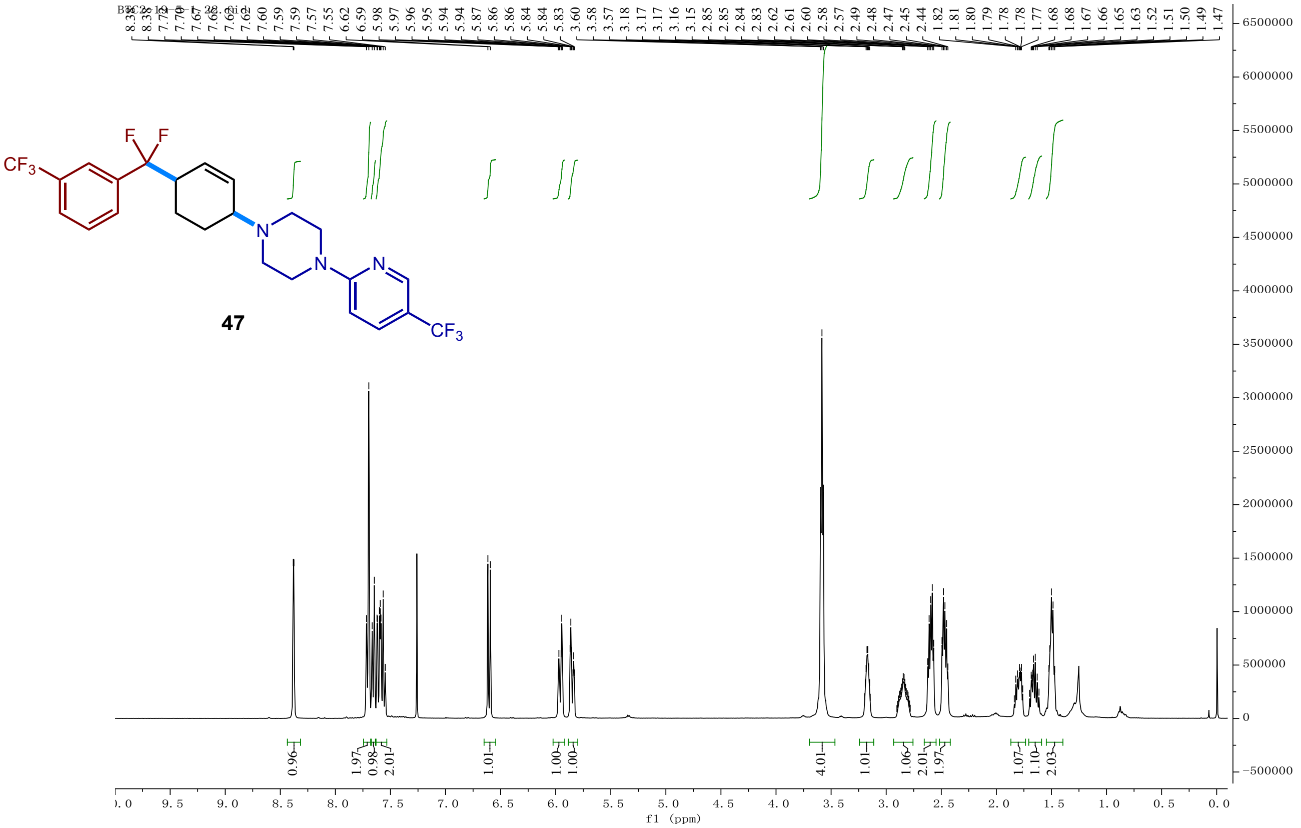

[illegible]
8.38 7.72 7.70 7.67 7.66 7.62 7.62 7.60 7.59 7.59 7.57 7.55 6.62 6.59 5.98 5.97 5.96 5.95 5.94 5.94 5.87 5.86 5.86 5.84 5.84 5.83 3.60 3.58 3.57 3.18 3.17 3.17 3.16 3.15 2.85 2.85 2.84 2.83 2.62 2.61 2.60 2.58 2.57 2.49 2.48 2.47 2.45 2.44 1.82 1.81 1.80 1.79 1.78 1.78 1.77 1.68 1.68 1.67 1.66 1.65 1.63 1.52 1.51 1.50 1.49 1.47
CF3
F F
N
N
N
CF3
47
0.96 1.97 0.98 2.01 1.01 1.00 1.00 4.01 1.01 1.06 2.01 1.97 1.07 1.10 2.03
f1 (ppm)

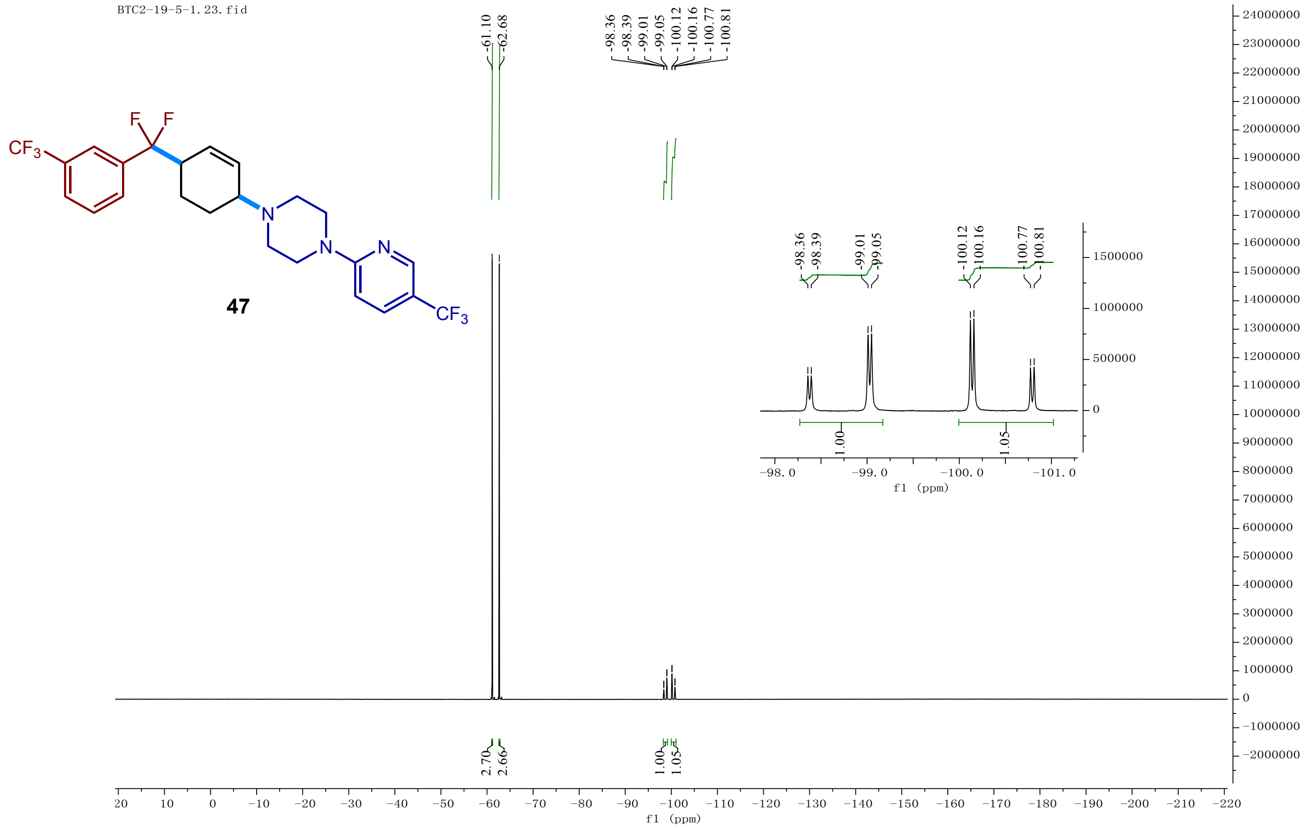

BTC2-19-5-1.23.fid
-61.10 -62.68
-98.36 -98.39 -99.01 -99.05 -100.12 -100.16 -100.77 -100.81
CF3
F F
N
N
N
CF3
47
1.00 1.05
2.70 2.66 1.00 1.05
f1 (ppm)

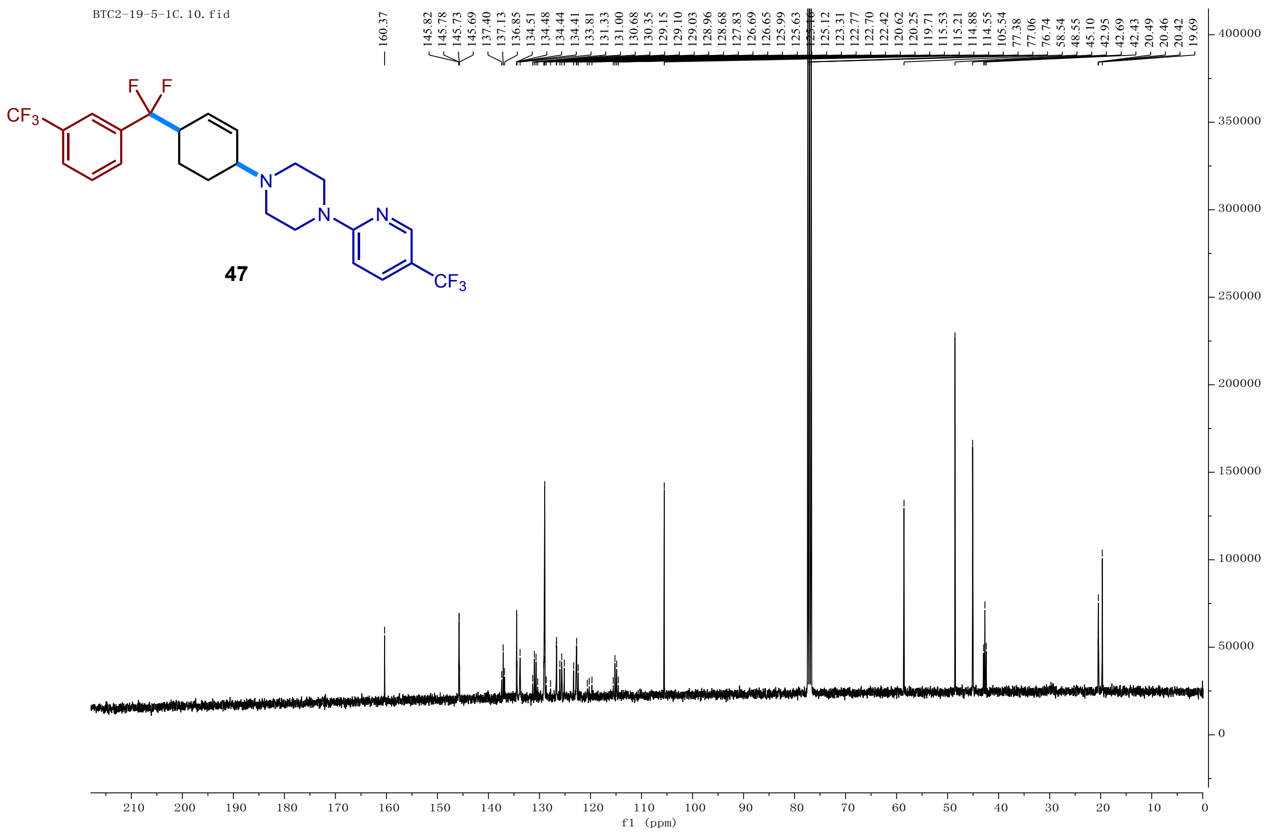

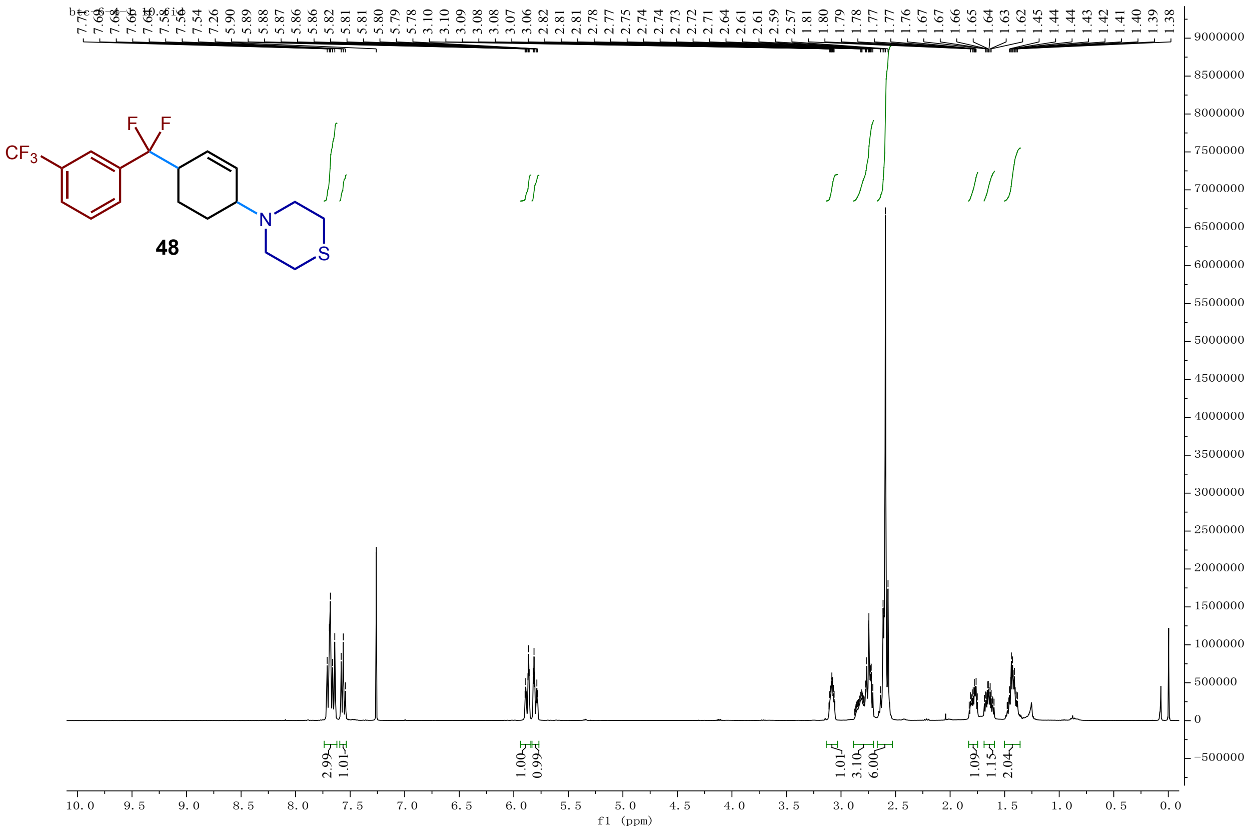

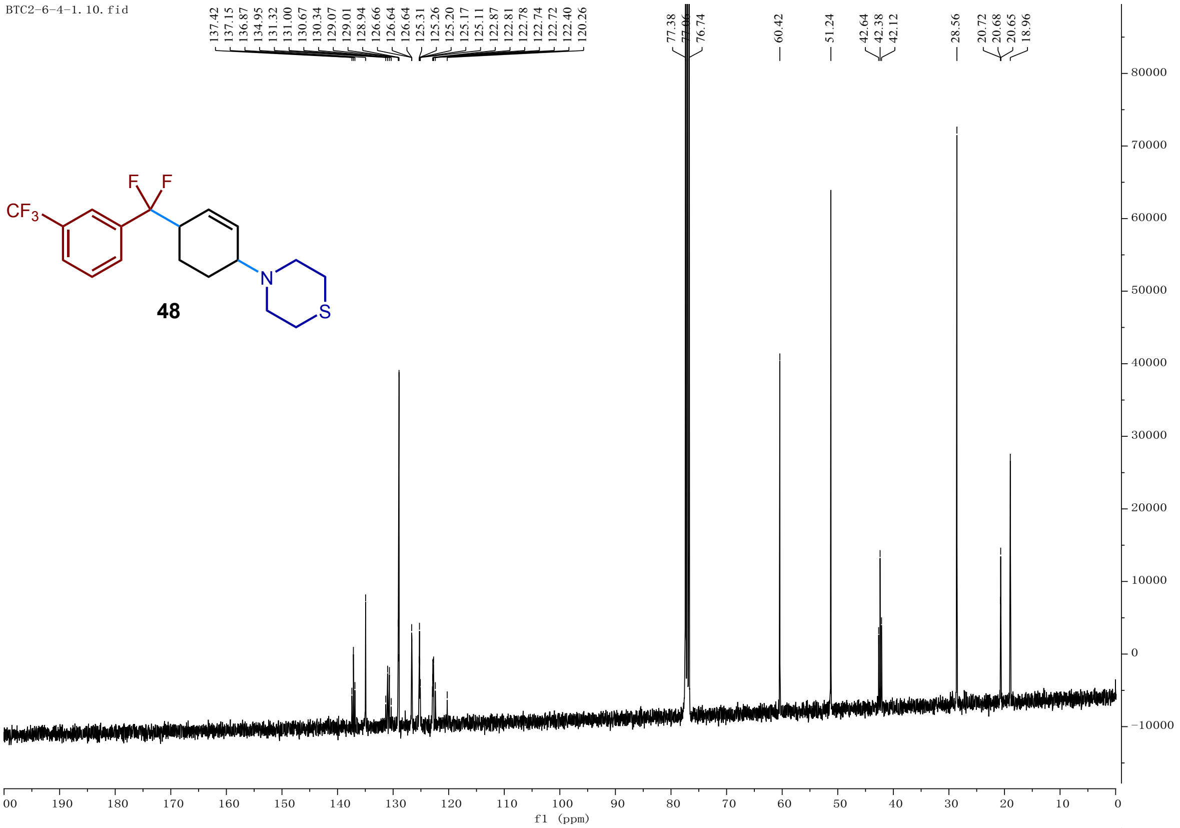
BTC2-6-4-1.10.fid
137.42
137.15
136.87
134.95
131.32
131.00
130.67
130.34
129.07
129.01
128.94
126.66
126.64
126.64
125.31
125.26
125.20
125.17
125.11
122.87
122.81
122.78
122.74
122.72
122.40
120.26
77.38
76.74
60.42
51.24
42.64
42.38
42.12
28.56
20.72
20.68
20.65
18.96
CF3
F
F
N
S
48
f1 (ppm)

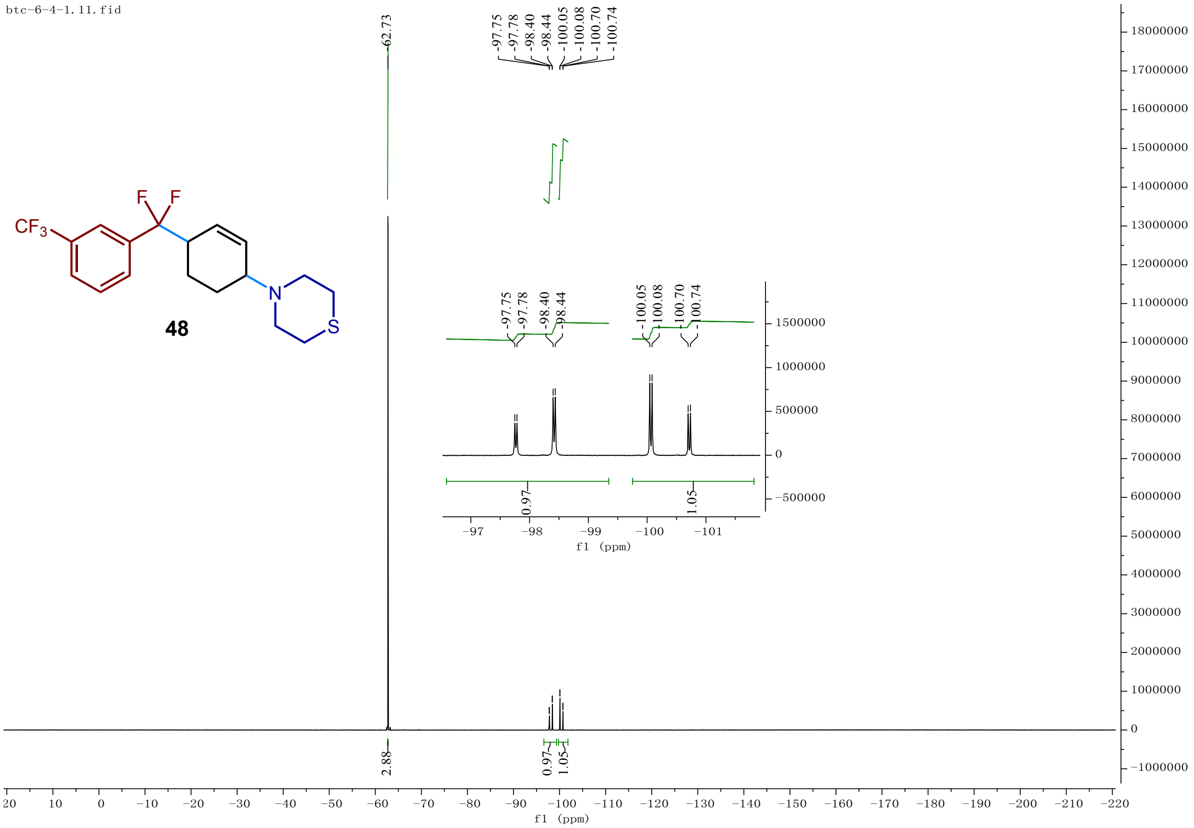
btc-6-4-1.11.fid
-62.73
-97.75
-97.78
-98.40
-98.44
-100.05
-100.08
-100.70
-100.74
CF3
F
F
N
S
48
0.97
1.05
2.88
f1 (ppm)

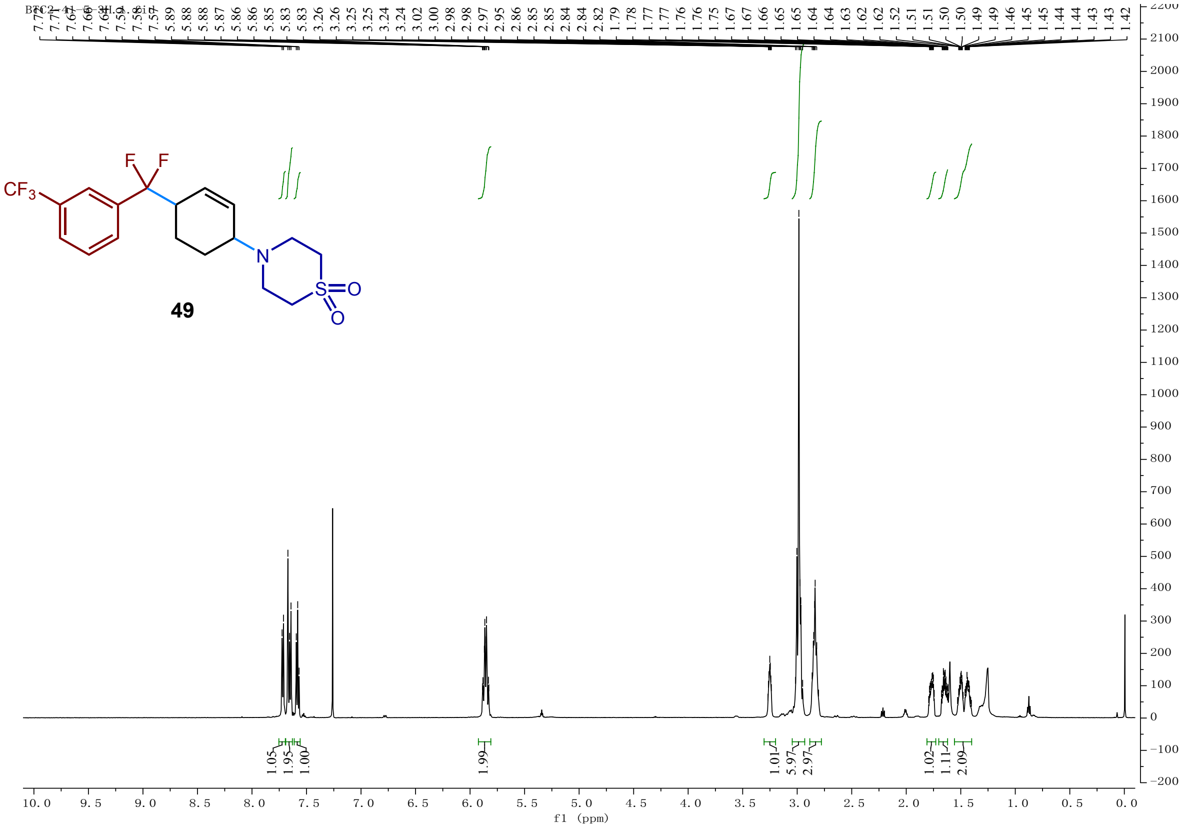
7.72 7.71 7.67 7.66 7.64 7.59 7.58 7.57 5.89 5.88 5.88 5.87 5.86 5.86 5.85 5.83 5.83 3.26 3.26 3.25 3.25 3.24 3.24 3.02 3.00 2.98 2.98 2.97 2.95 2.86 2.85 2.85 2.84 2.84 2.82 1.79 1.78 1.77 1.77 1.76 1.76 1.75 1.67 1.67 1.66 1.65 1.65 1.64 1.64 1.63 1.62 1.62 1.52 1.51 1.51 1.50 1.50 1.49 1.49 1.46 1.45 1.45 1.44 1.44 1.43 1.43 1.42
CF3
F F
N
S=O
O
49
1.05 1.95 1.00
1.99
1.01 5.97 2.97
1.02 1.11 2.09
f1 (ppm)

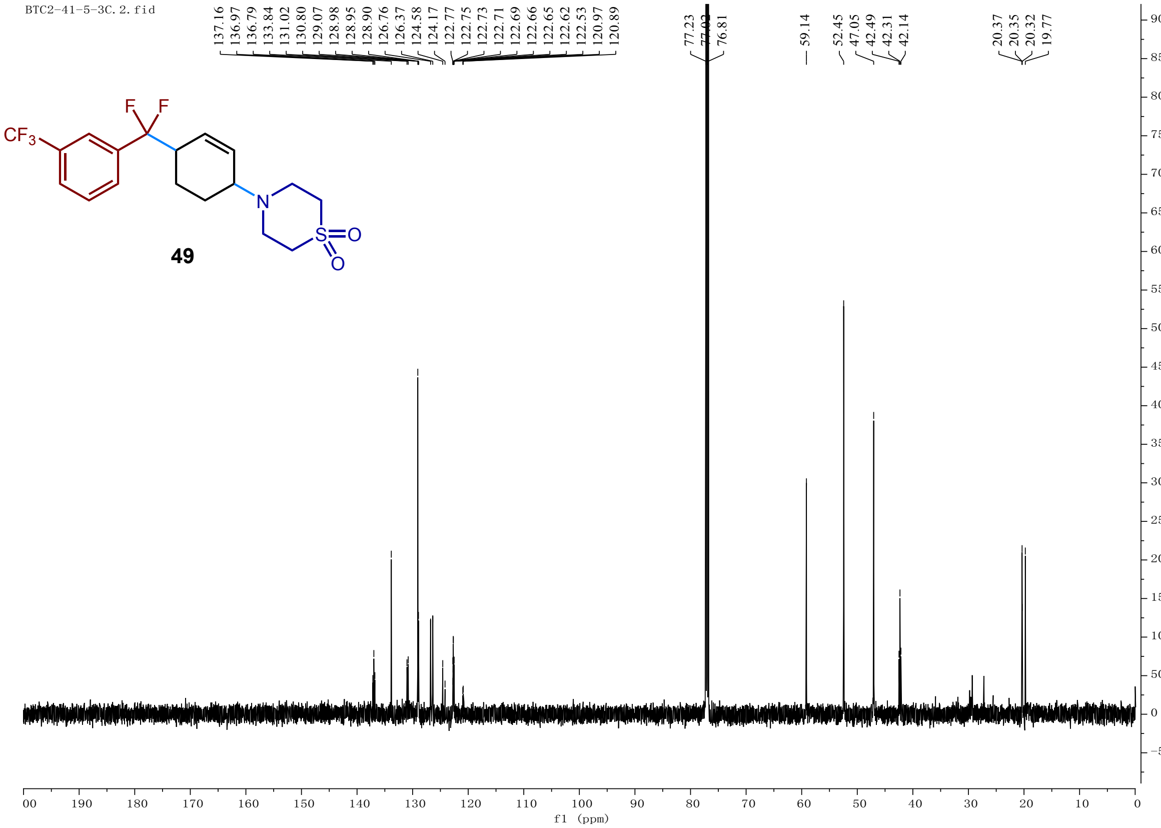
BTC2-41-5-3C.2.fid
137.16 136.97 136.79 133.84 131.02 130.80 129.07 128.98 128.95 128.90 126.76 126.37 124.58 124.17 122.77 122.75 122.73 122.71 122.69 122.66 122.65 122.62 122.53 120.97 120.89
77.23 77.02 76.81
59.14
52.45 47.05 42.49 42.31 42.14
20.37 20.35 20.32 19.77
CF3
F F
N
S=O
O
49
f1 (ppm)

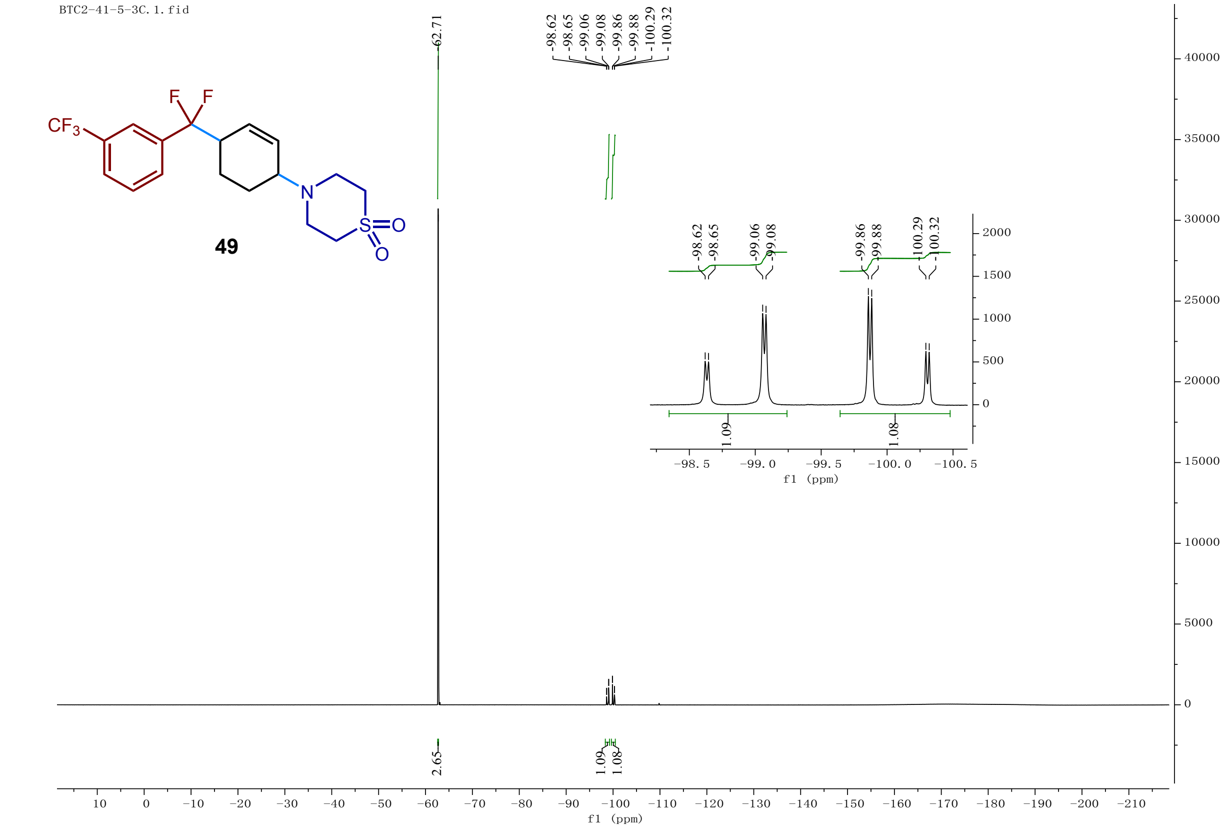
BTC2-41-5-3C.1.fid
49
f1 (ppm)

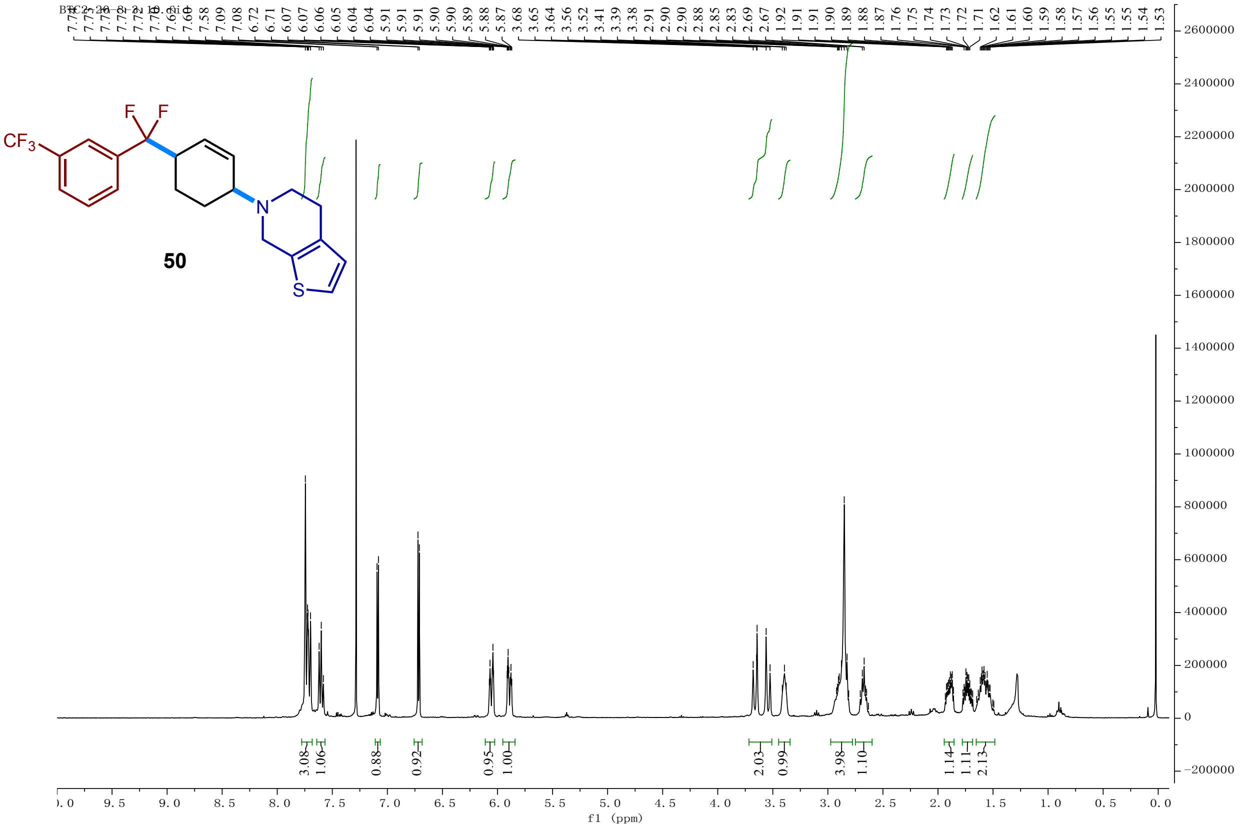
50
f1 (ppm)

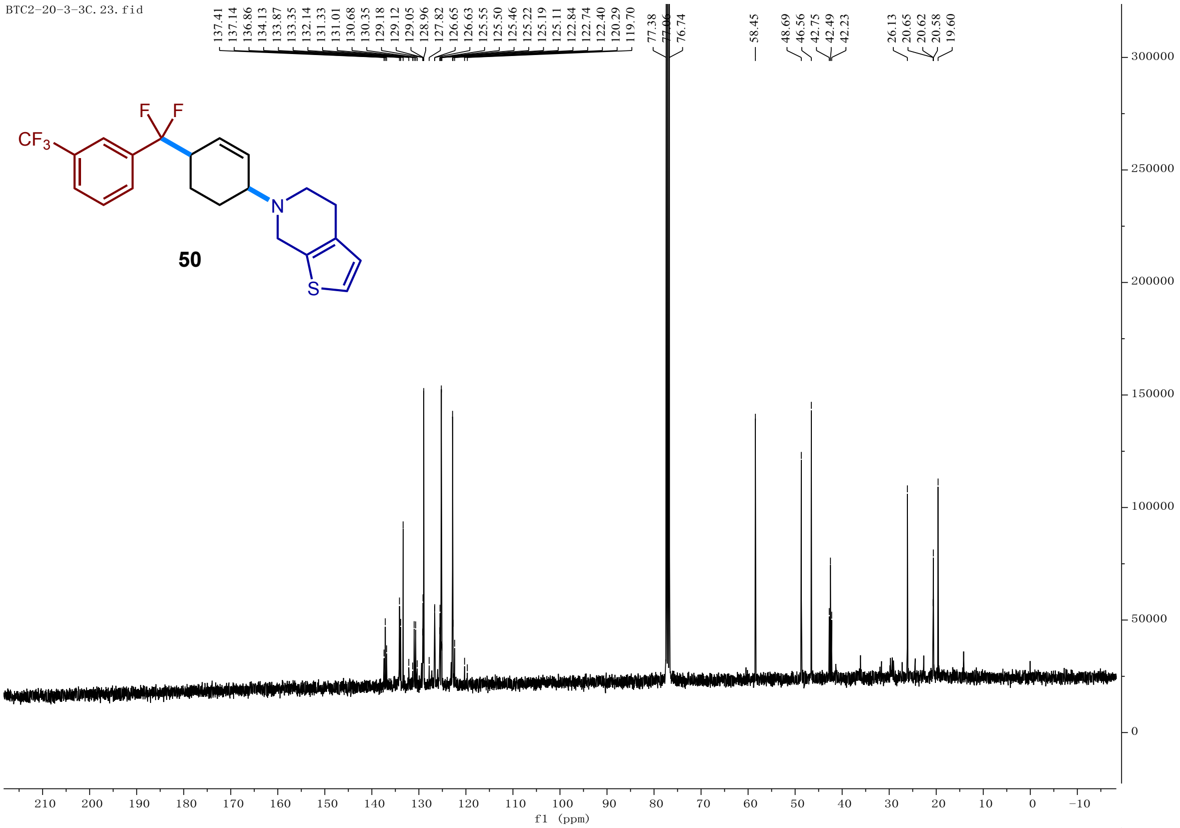
BTC2-20-3-3C.23.fid
137.41 137.14 136.86 134.13 133.87 133.35 132.14 131.33 131.01 130.68 130.35 129.18 129.12 129.05 128.96 127.82 126.65 126.63 125.55 125.50 125.46 125.22 125.19 125.11 122.84 122.74 122.40 120.29 119.70 77.38 77.06 76.74 58.45 48.69 46.56 42.75 42.49 42.23 26.13 20.65 20.62 20.58 19.60
CF3
F F
N
S
50
f1 (ppm)

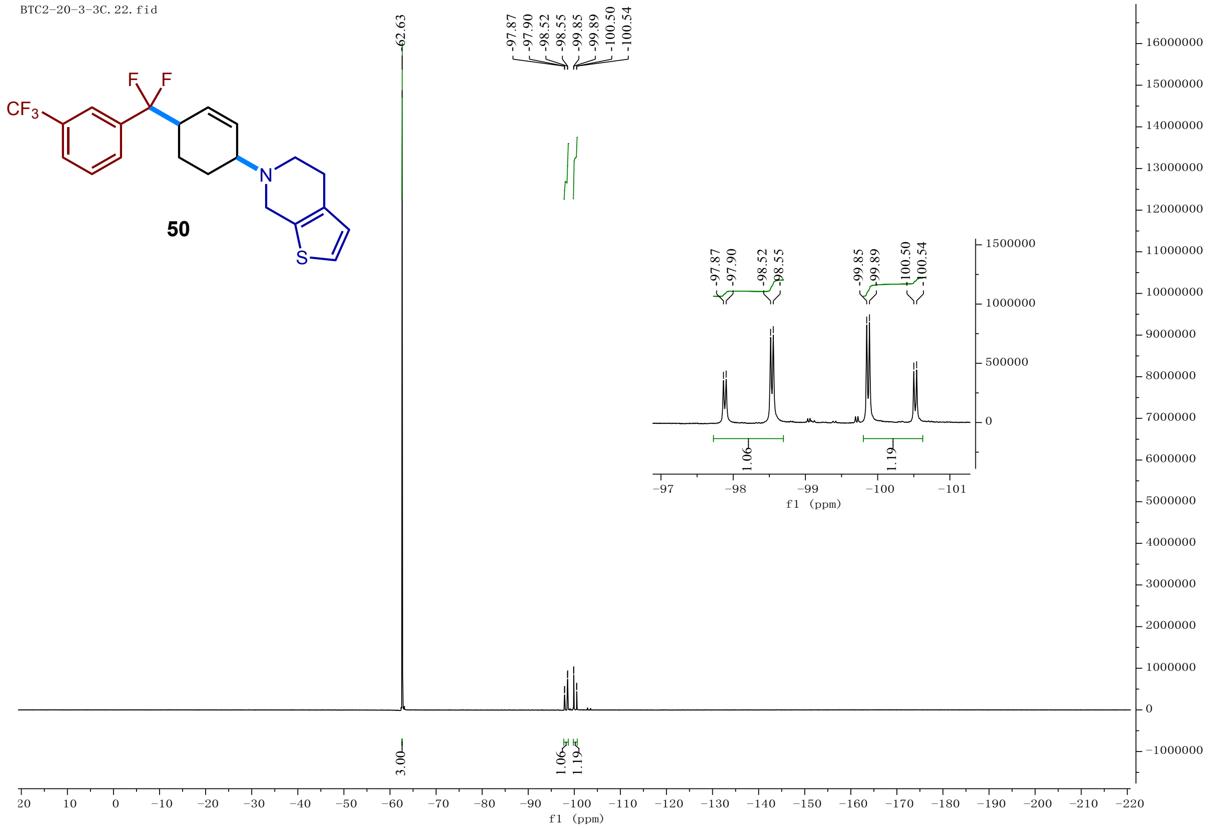
BTC2-20-3-3C.22.fid
-62.63
-97.87 -97.90 -98.52 -98.55 -99.85 -99.89 -100.50 -100.54
CF3
F F
N
S
50
1.06
1.19
3.00
f1 (ppm)

BTC2-42-3-3.1.fid

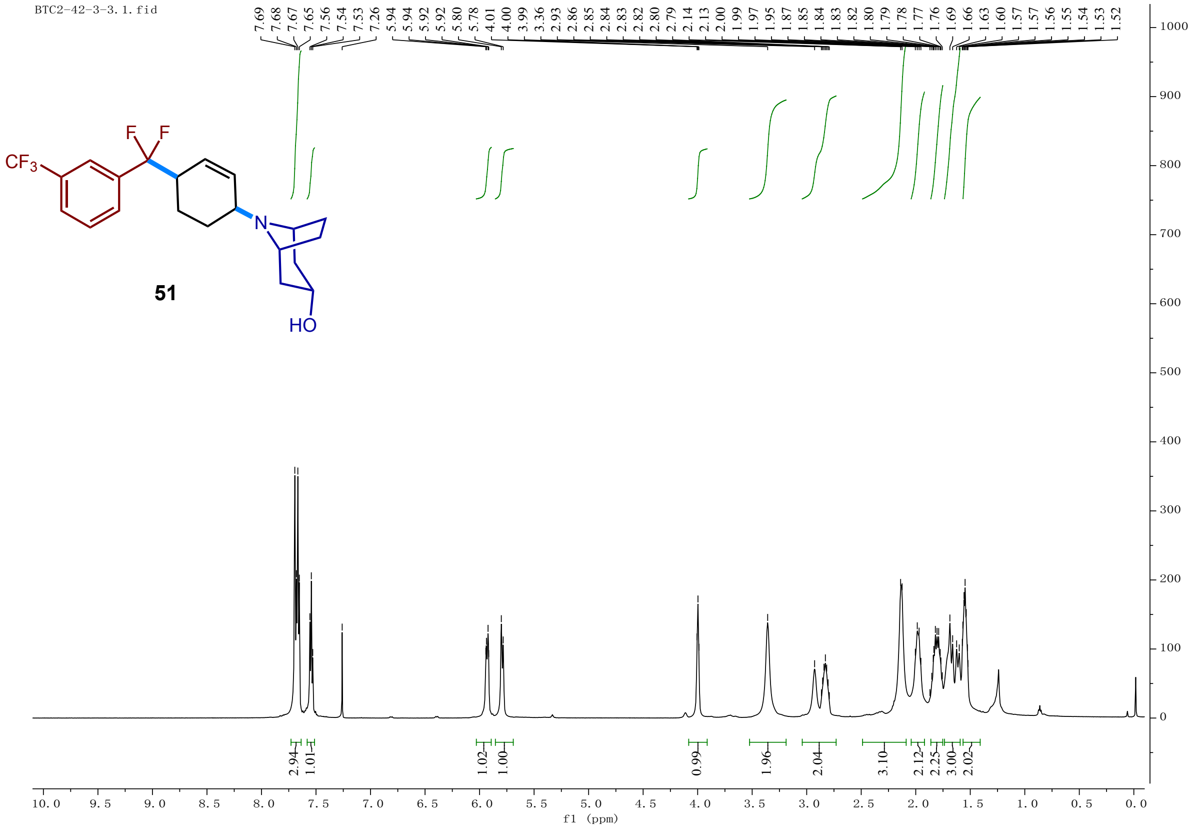

51

BTC2-42-3-3C.2.fid

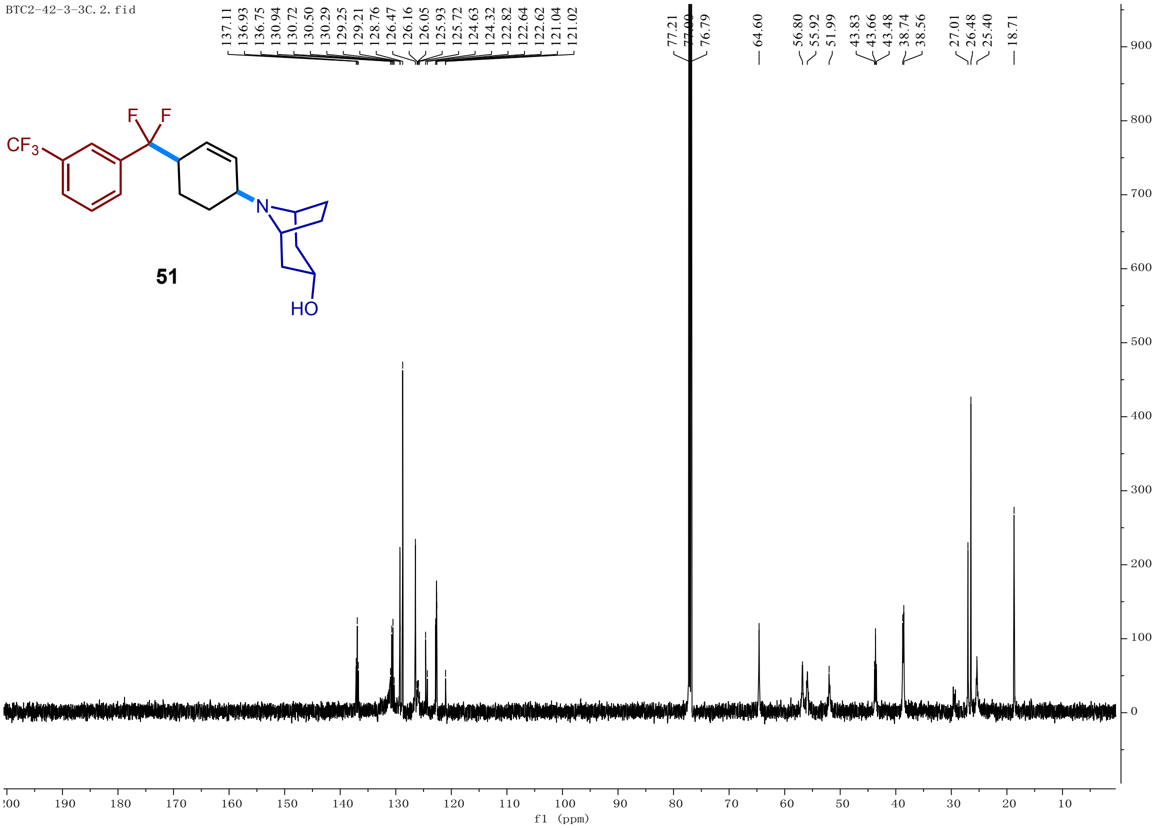

51

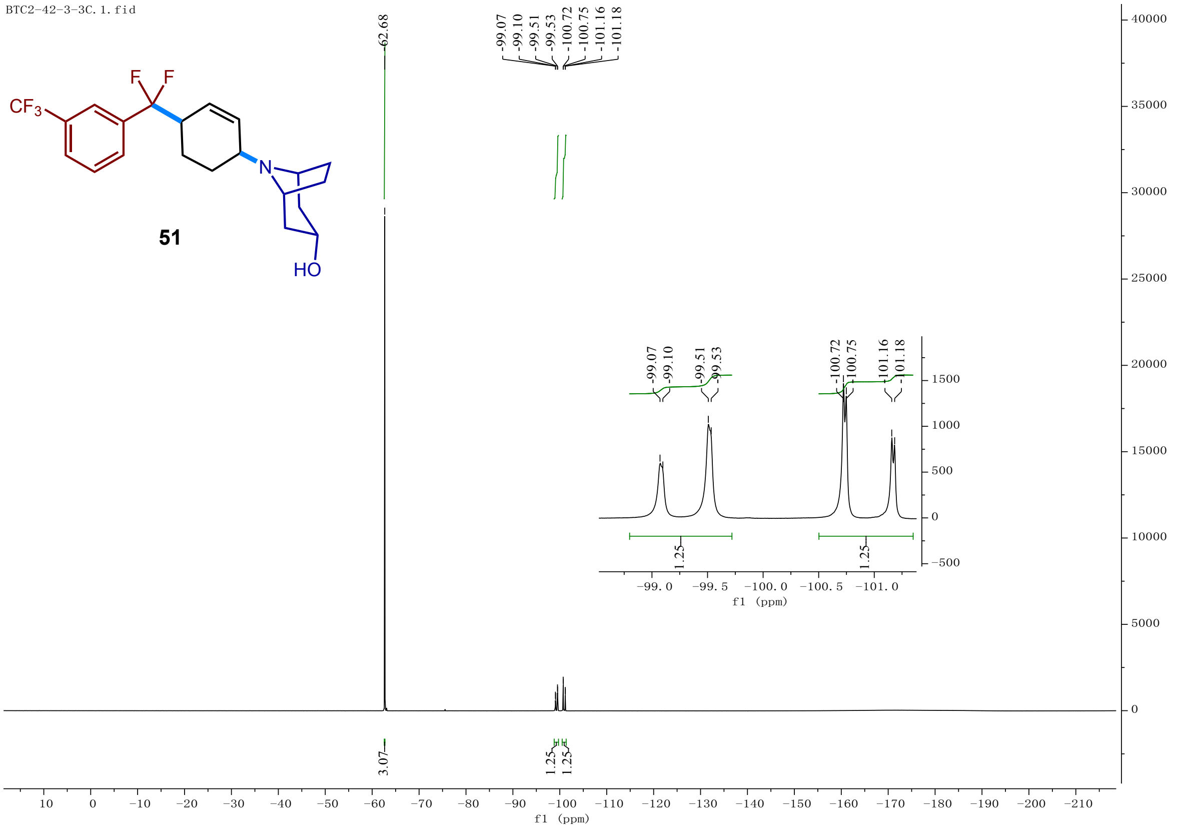
BTC2-42-3-3C.1.fid
CF3
F F
N
HO
51
-62.68
-99.07
-99.10
-99.51
-99.53
-100.72
-100.75
-101.16
-101.18
3.07
1.25
1.25
f1 (ppm)

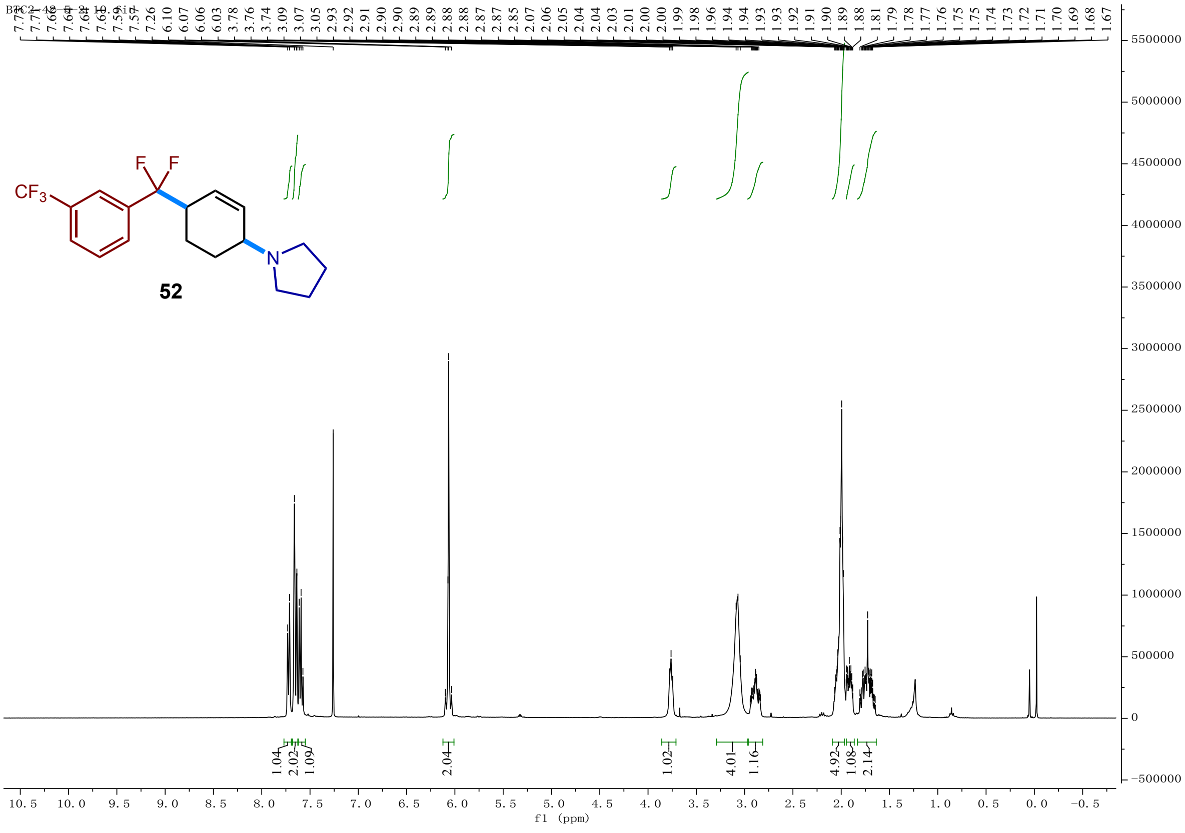
CF3
F F
N
52
7.73
7.71
7.66
7.64
7.64
7.61
7.59
7.57
7.26
6.10
6.07
6.06
6.03
3.78
3.76
3.74
3.09
3.07
3.05
2.93
2.92
2.91
2.90
2.90
2.89
2.89
2.88
2.88
2.87
2.87
2.85
2.07
2.06
2.05
2.04
2.04
2.03
2.01
2.00
2.00
1.99
1.98
1.96
1.94
1.93
1.93
1.92
1.91
1.90
1.89
1.88
1.81
1.79
1.78
1.77
1.76
1.75
1.75
1.74
1.73
1.72
1.71
1.70
1.69
1.68
1.67
1.04
2.02
1.09
2.04
1.02
4.01
1.16
4.92
1.08
2.14
f1 (ppm)

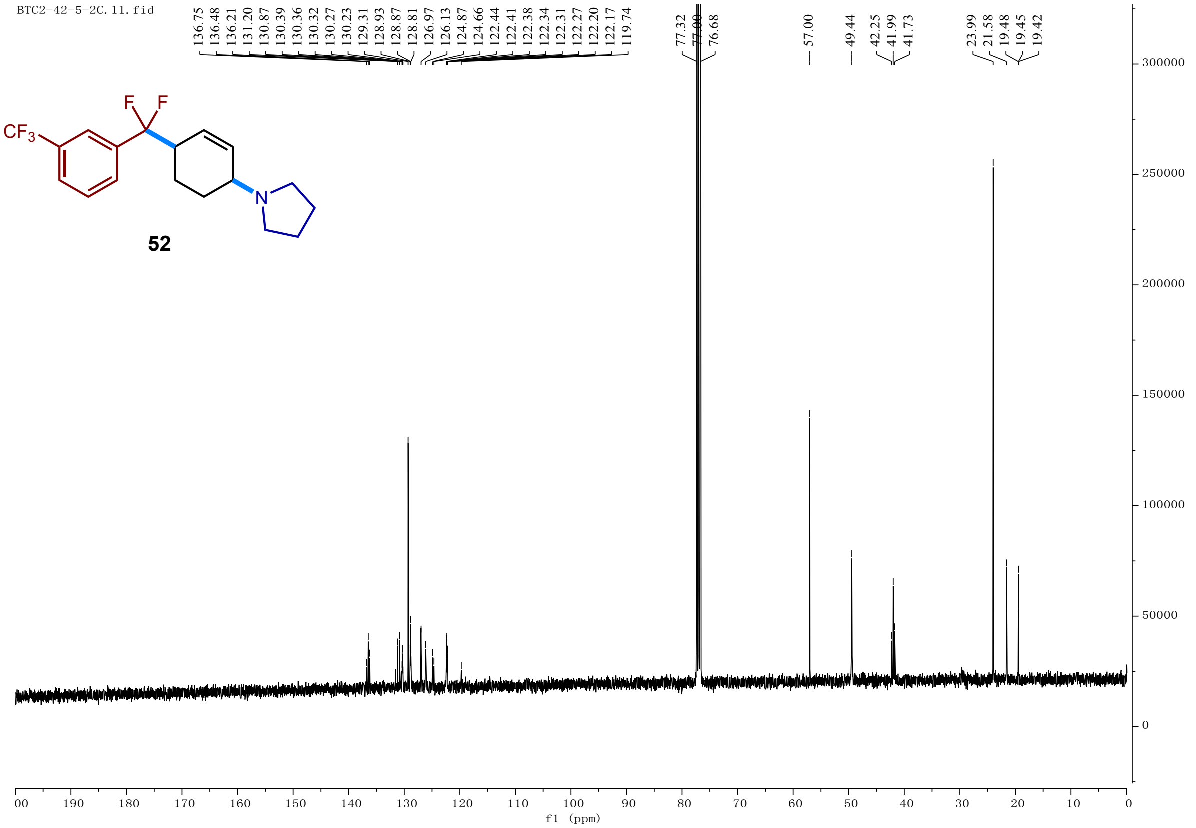
BTC2-42-5-2C.11.fid
136.75
136.48
136.21
131.20
130.87
130.39
130.36
130.32
130.27
130.23
129.31
128.93
128.87
128.81
126.97
126.13
124.87
124.66
122.44
122.41
122.38
122.34
122.31
122.27
122.20
122.17
119.74
77.32
77.00
76.68
57.00
49.44
42.25
41.99
41.73
23.99
21.58
19.48
19.45
19.42
CF3
F F
N
52
f1 (ppm)

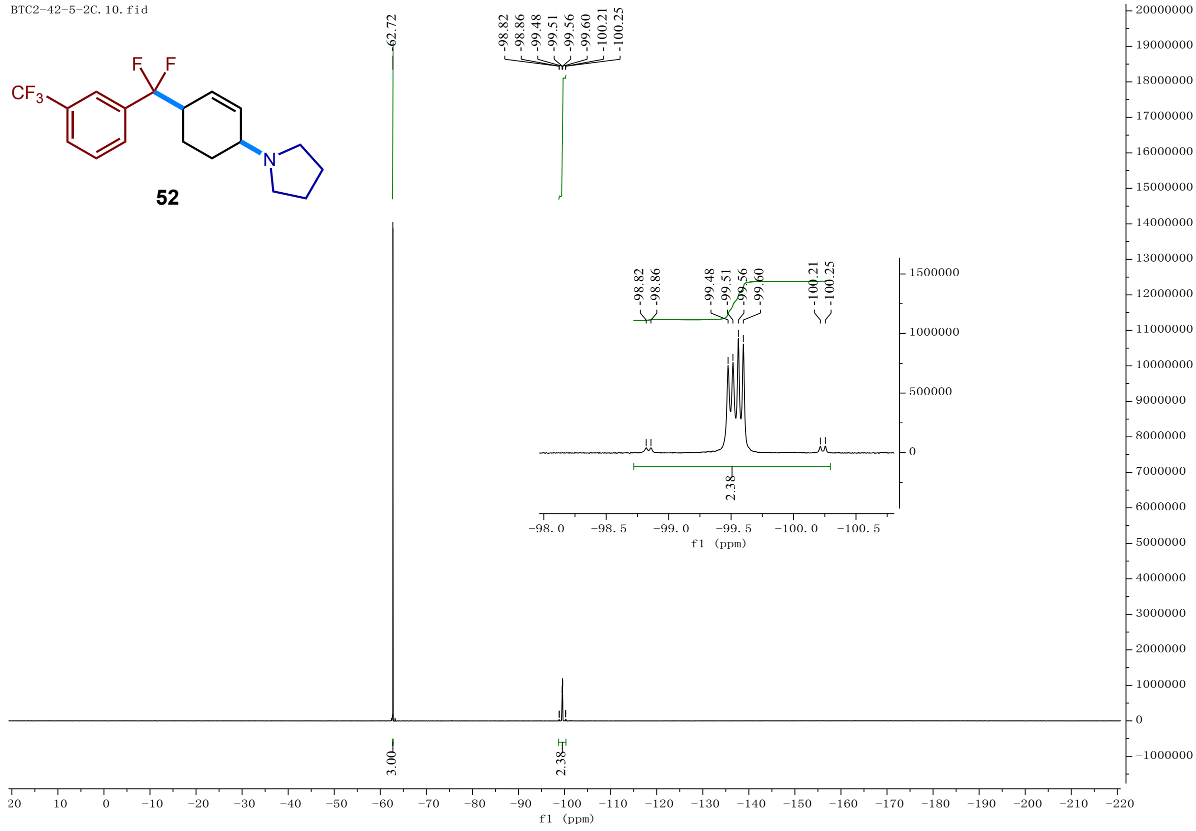
BTC2-42-5-2C.10.fid
-62.72
-98.82
-98.86
-99.48
-99.51
-99.56
-99.60
-100.21
-100.25
CF3
F F
N
52
2.38
3.00
f1 (ppm)

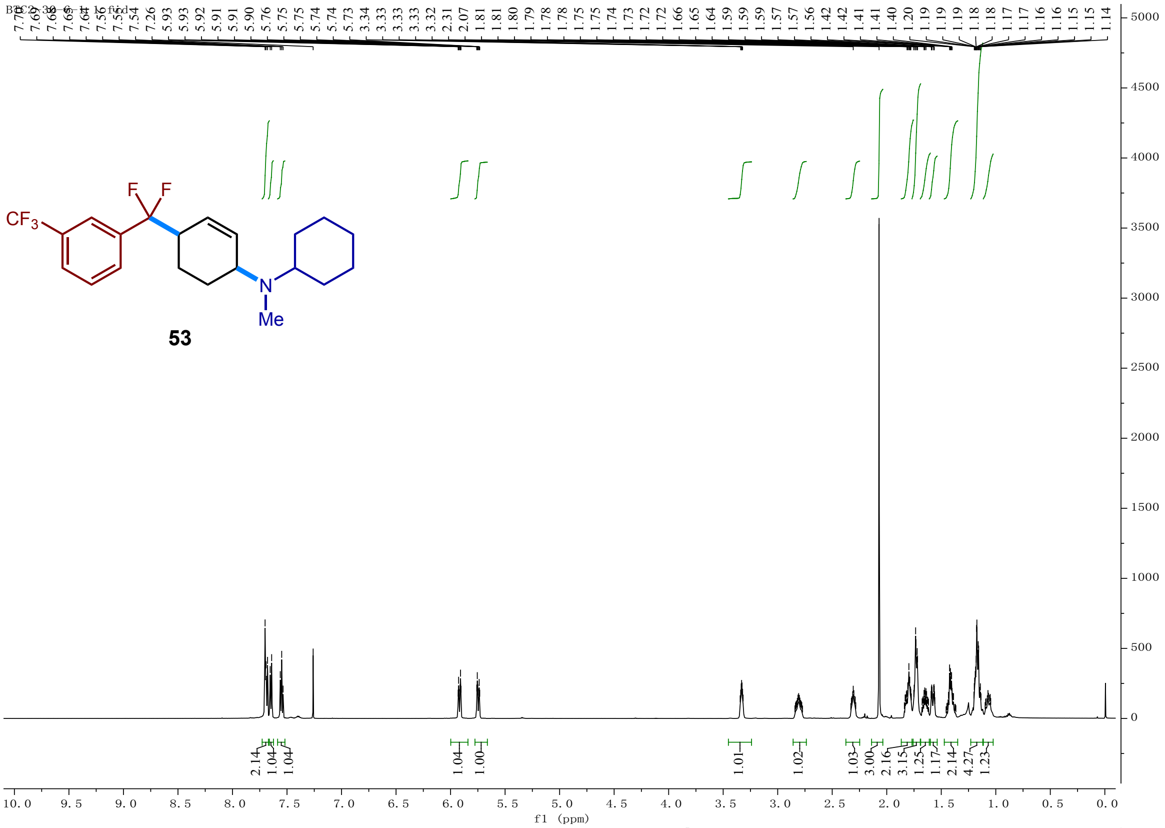
BTC2-30-6-1[illegible]
7.70 7.69 7.68 7.65 7.64 7.56 7.55 7.54 7.26 5.93 5.93 5.92 5.91 5.91 5.90 5.76 5.75 5.75 5.74 5.74 5.73 3.34 3.33 3.33 3.32 2.31 2.07 1.81 1.81 1.80 1.79 1.78 1.78 1.75 1.75 1.74 1.73 1.72 1.72 1.66 1.65 1.64 1.59 1.59 1.59 1.57 1.57 1.56 1.42 1.42 1.41 1.41 1.40 1.20 1.19 1.19 1.19 1.18 1.18 1.17 1.17 1.16 1.16 1.15 1.15 1.14
CF3
F F
N
Me
53
2.14 1.04 1.04 1.04 1.00 1.01 1.02 1.03 3.00 2.16 3.15 1.25 1.17 2.14 4.27 1.23
f1 (ppm)

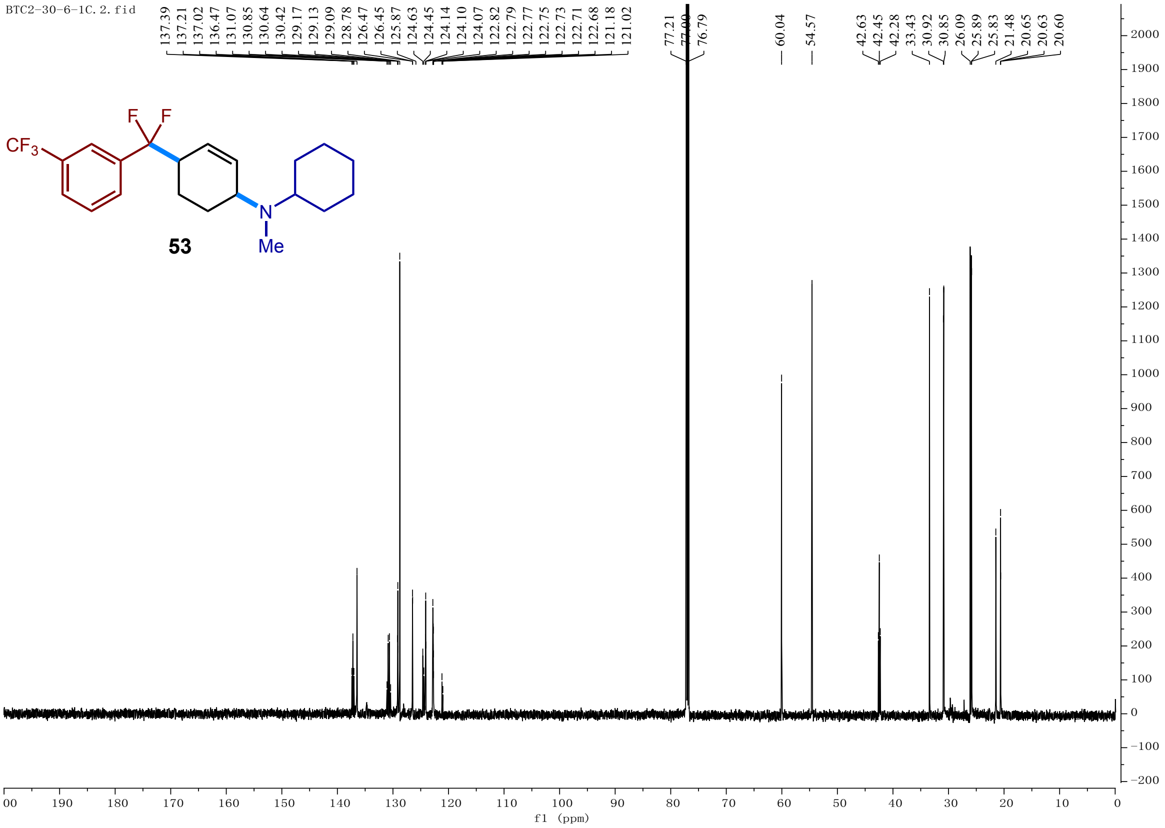
BTC2-30-6-1C.2.fid
137.39 137.21 137.02 136.47 131.07 130.85 130.64 130.42 129.17 129.13 129.09 128.78 126.47 126.45 125.87 124.63 124.45 124.14 124.10 124.07 122.82 122.79 122.77 122.75 122.73 122.71 122.68 121.18 121.02
77.21 77.00 76.79
60.04
54.57
42.63 42.45 42.28 33.43 30.92 30.85 26.09 25.89 25.83 21.48 20.65 20.63 20.60
CF3
F F
N
Me
53
f1 (ppm)

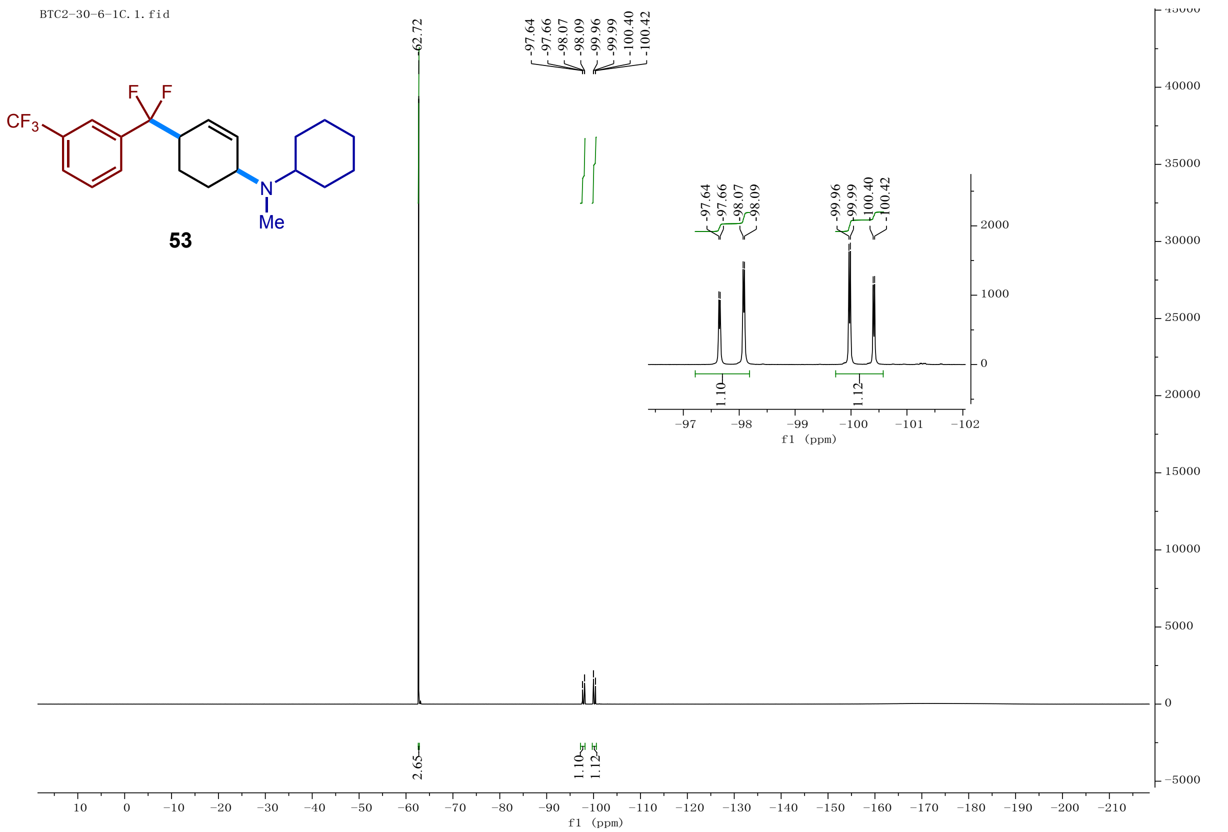
BTC2-30-6-1C.1.fid
CF3
F F
N
Me
53
-62.72
-97.64
-97.66
-98.07
-98.09
-99.96
-99.99
-100.40
-100.42
1.10
1.12
2.65
f1 (ppm)

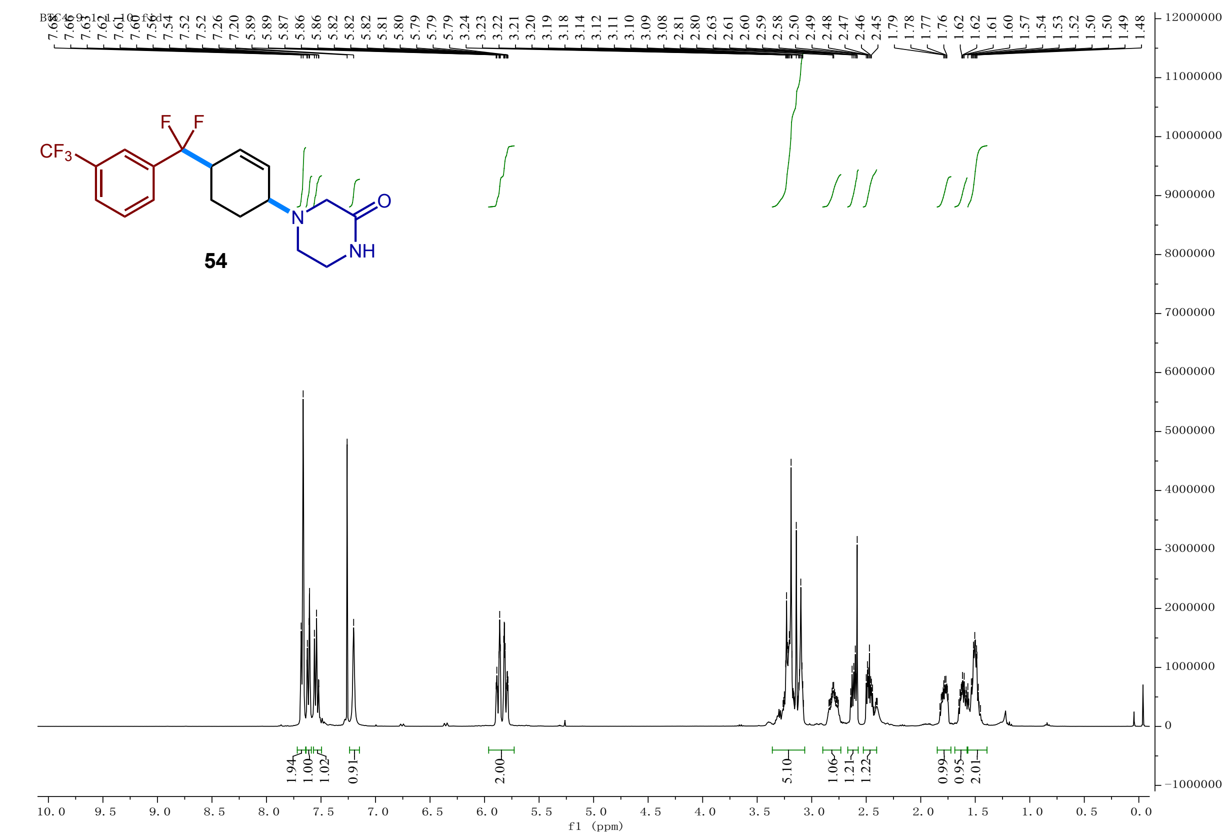
CF3
F F
N
O
NH
54
1.94
1.00
1.02
0.91
2.00
5.10
1.06
1.21
1.22
0.99
0.95
2.01
f1 (ppm)

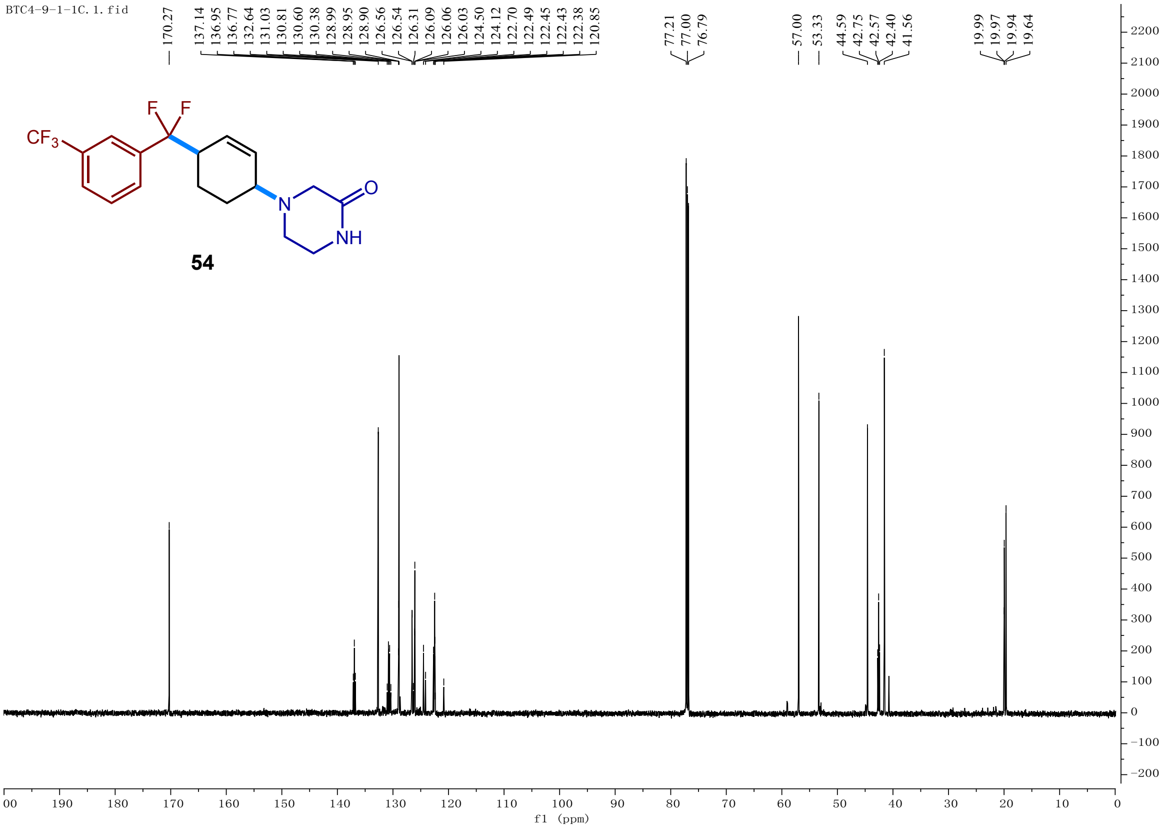
BTC4-9-1-1C.1.fid
170.27
137.14
136.95
136.77
132.64
131.03
130.81
130.60
130.38
128.99
128.95
128.90
126.56
126.54
126.31
126.09
126.06
126.03
124.50
124.12
122.70
122.49
122.45
122.43
122.38
120.85
77.21
77.00
76.79
57.00
53.33
44.59
42.75
42.57
42.40
41.56
19.99
19.97
19.94
19.64
CF3
F
F
N
O
NH
54
f1 (ppm)

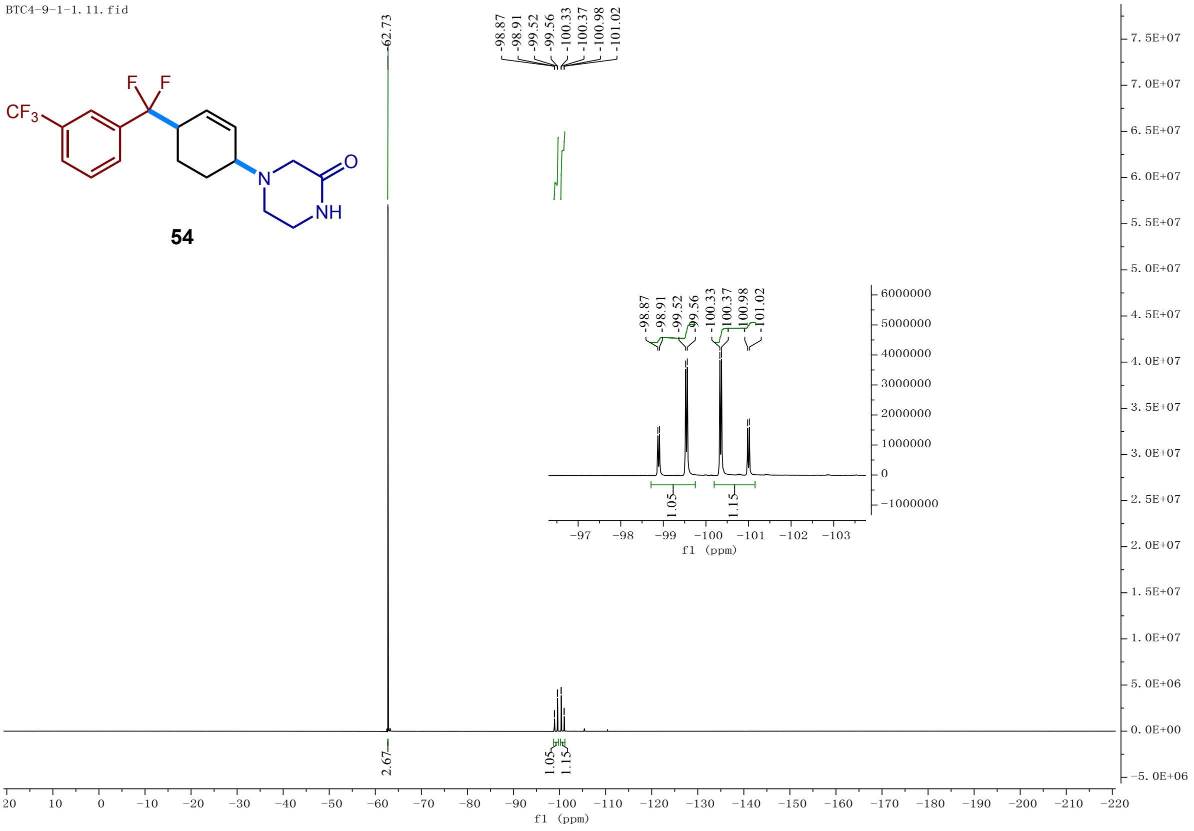
BTC4-9-1-1.11.fid
-62.73
-98.87
-98.91
-99.52
-99.56
-100.33
-100.37
-100.98
-101.02
CF3
F
F
N
O
NH
54
1.05
1.15
2.67
f1 (ppm)

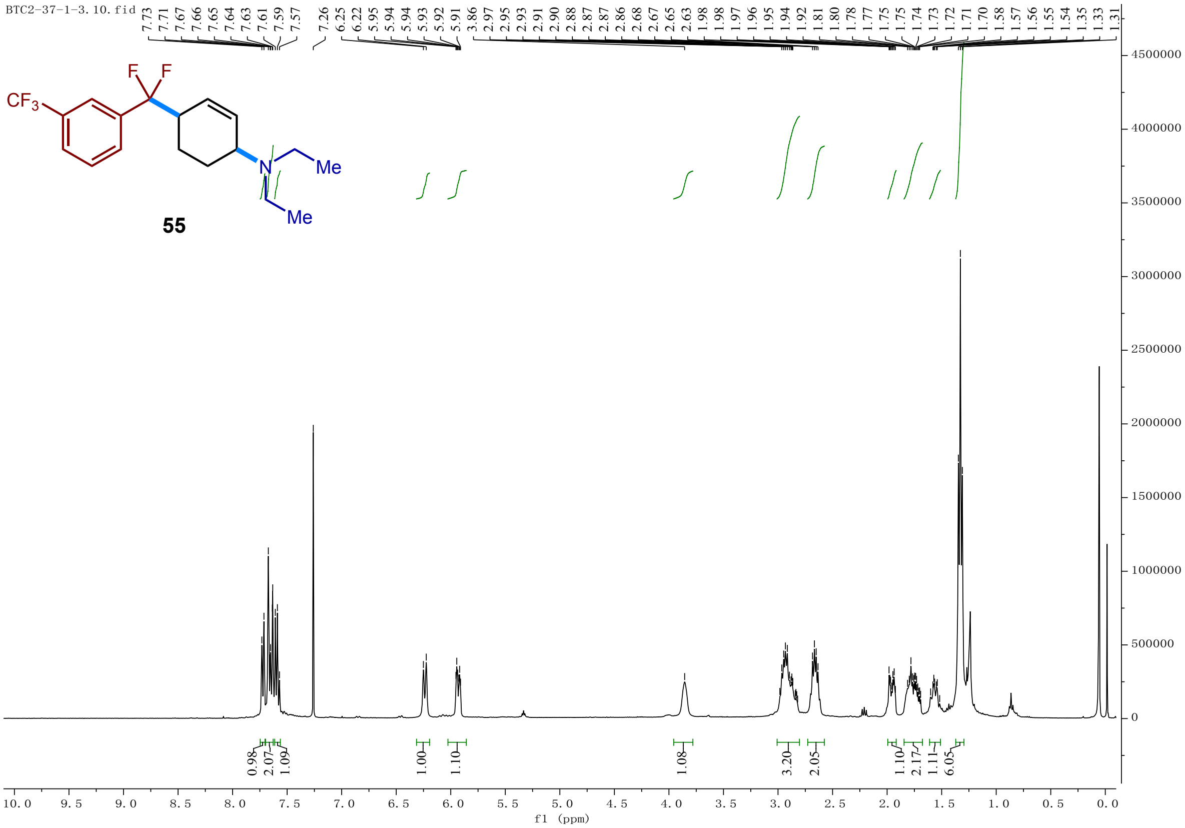

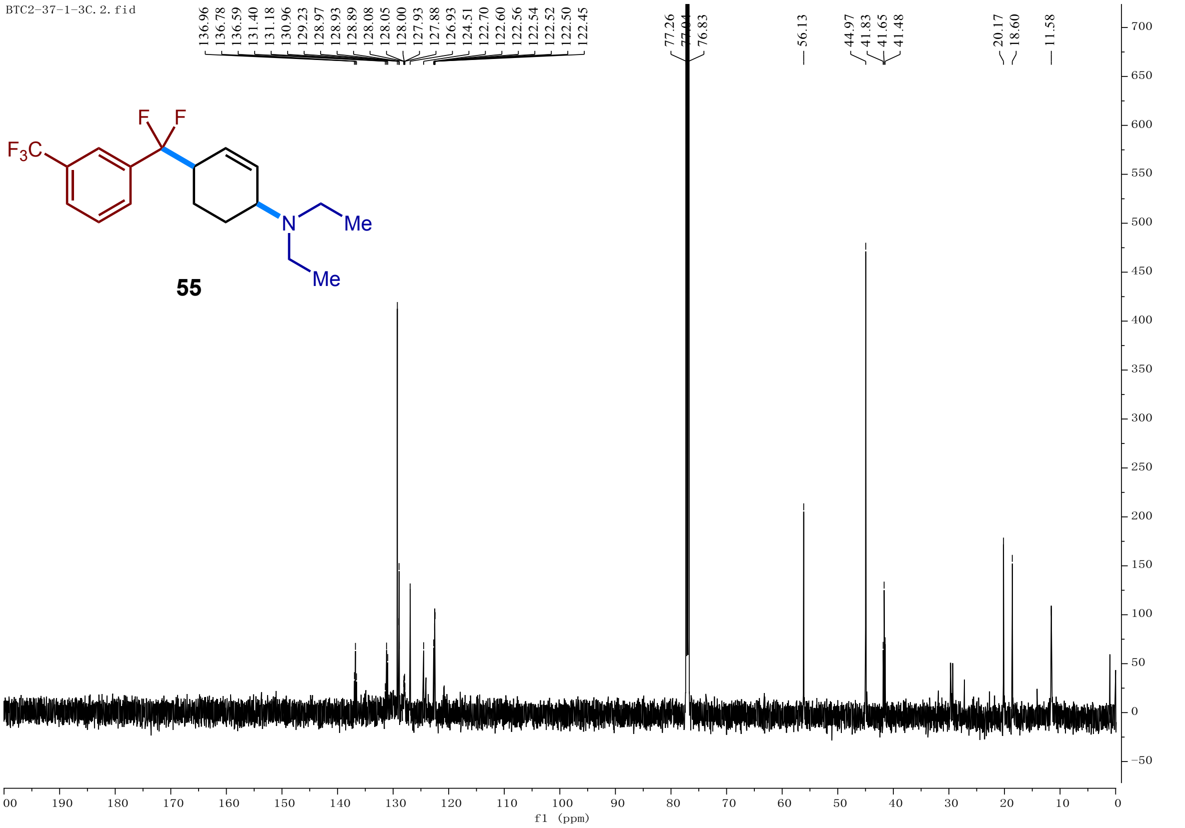

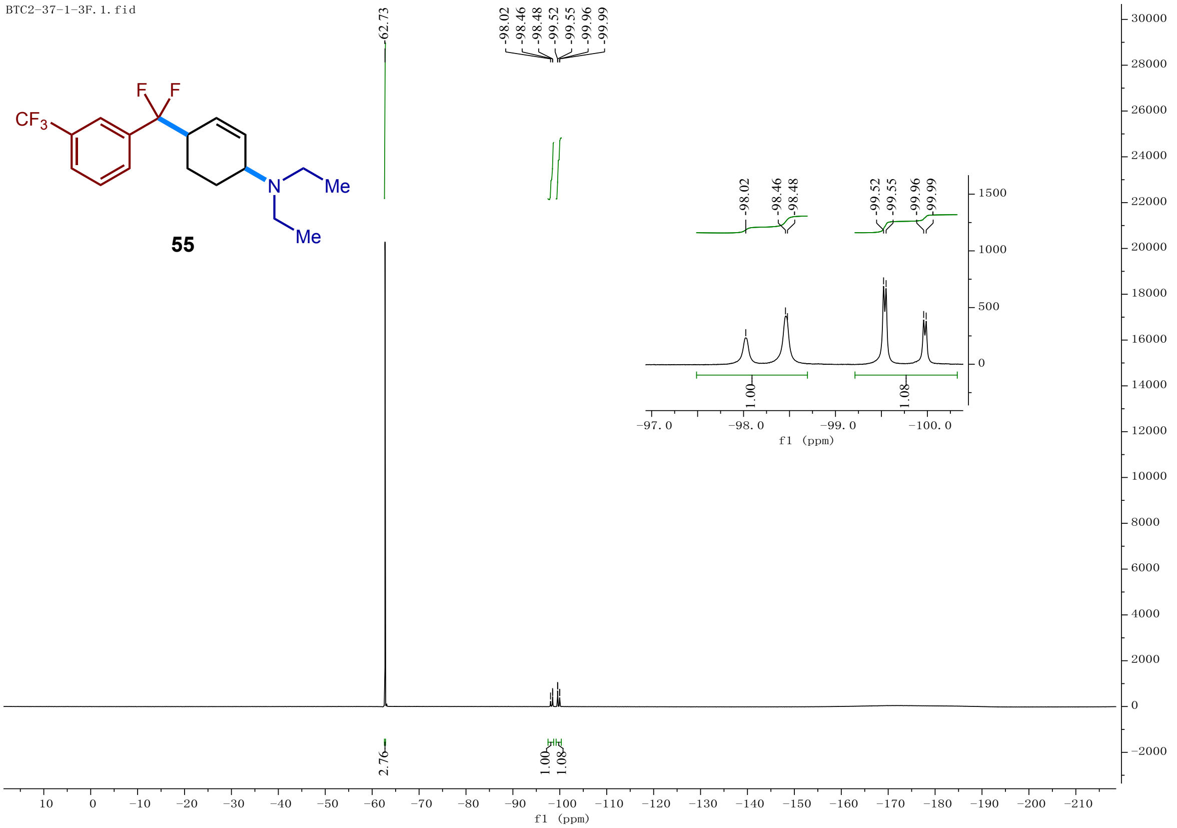
BTC2-37-1-3F.1.fid
-62.73
-98.02
-98.46
-98.48
-99.52
-99.55
-99.96
-99.99
CF3
F
F
N
Me
Me
55
2.76
1.00
1.08
f1 (ppm)

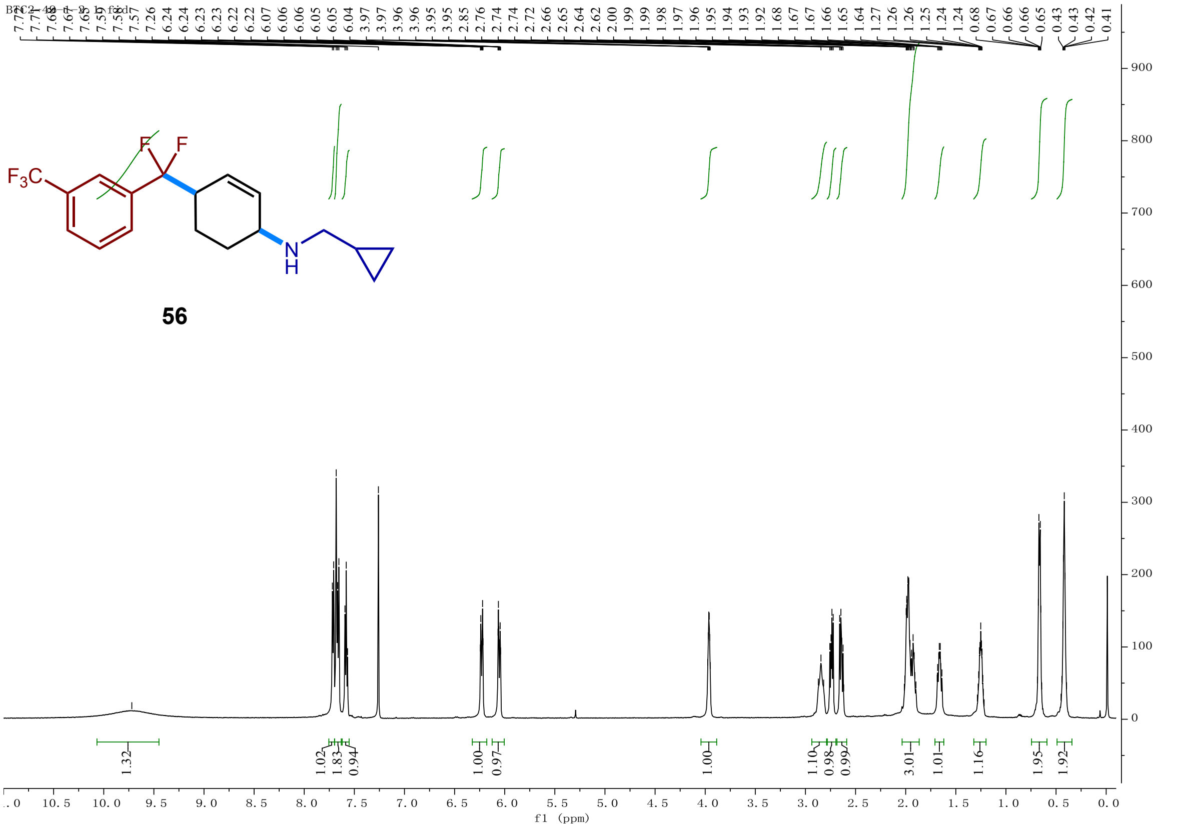
F3C
F
F
N
H
56
1.32
1.02
1.83
0.94
1.00
0.97
1.00
1.10
0.98
0.99
3.01
1.01
1.16
1.95
1.92
f1 (ppm)

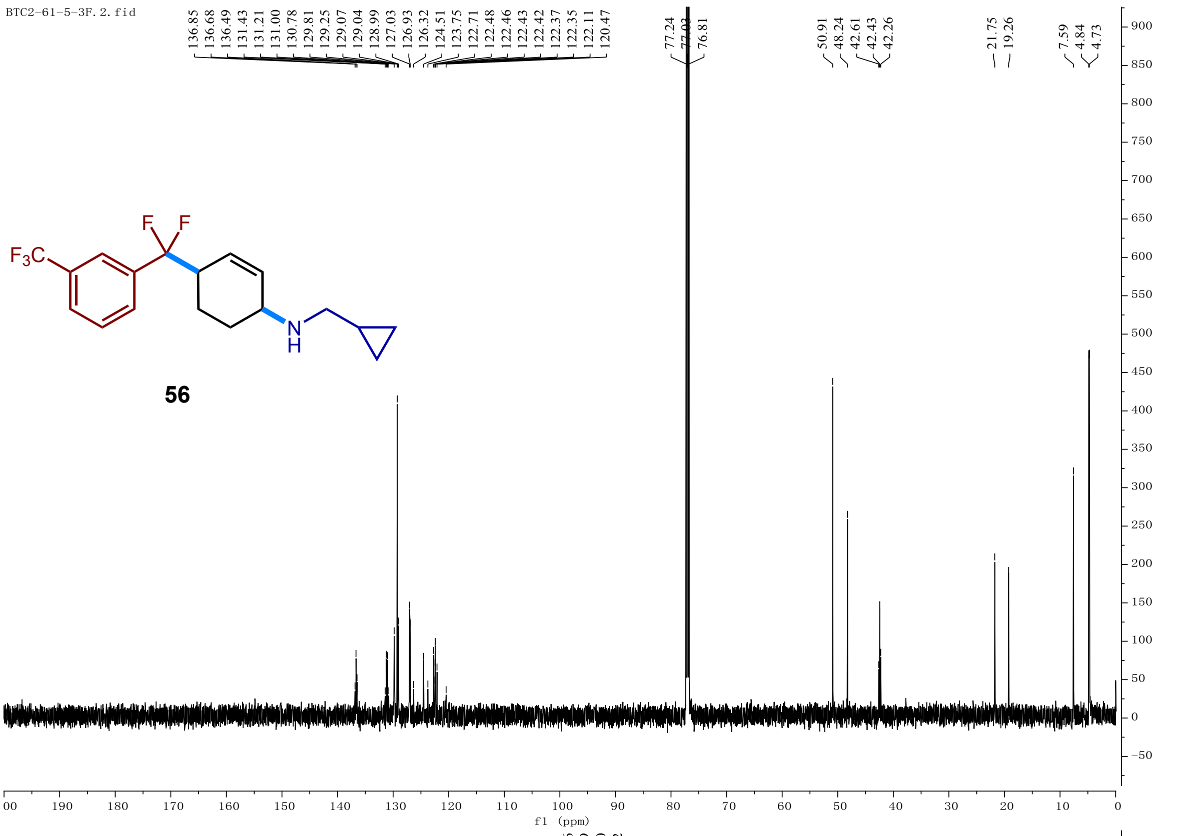

56

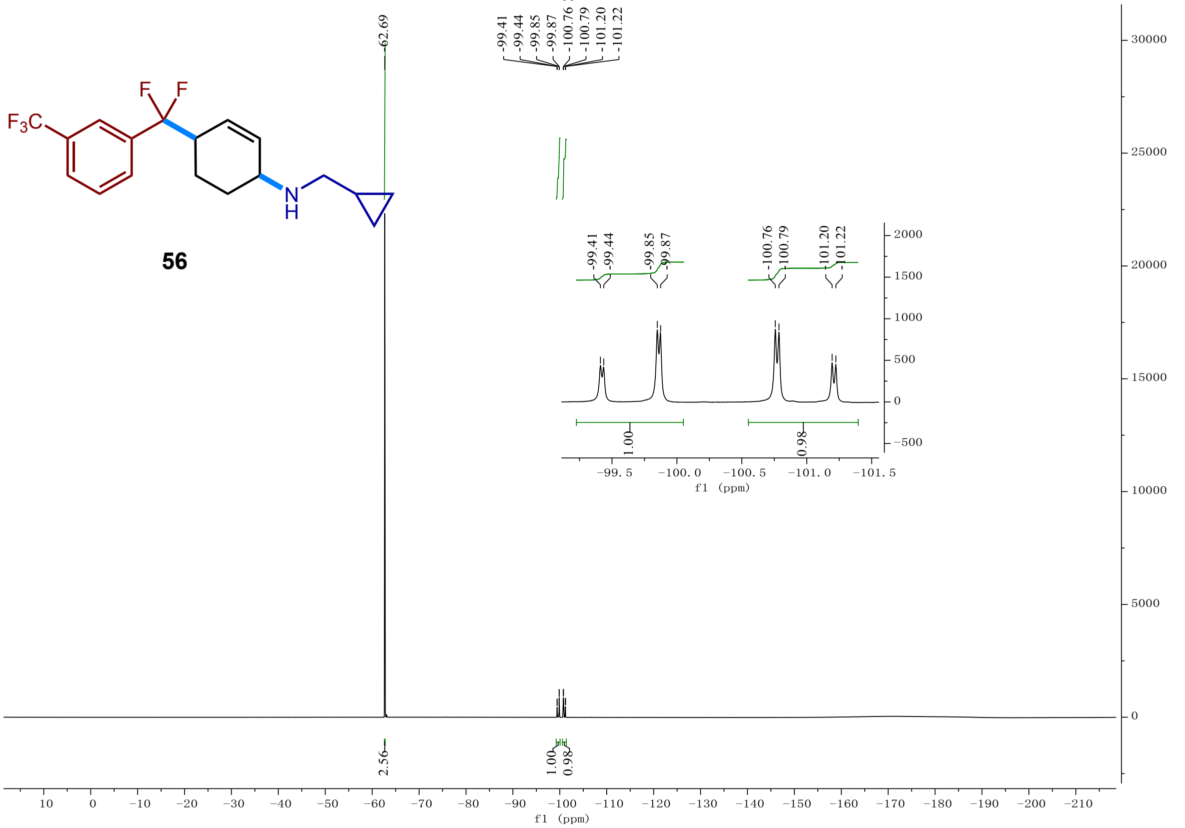

56

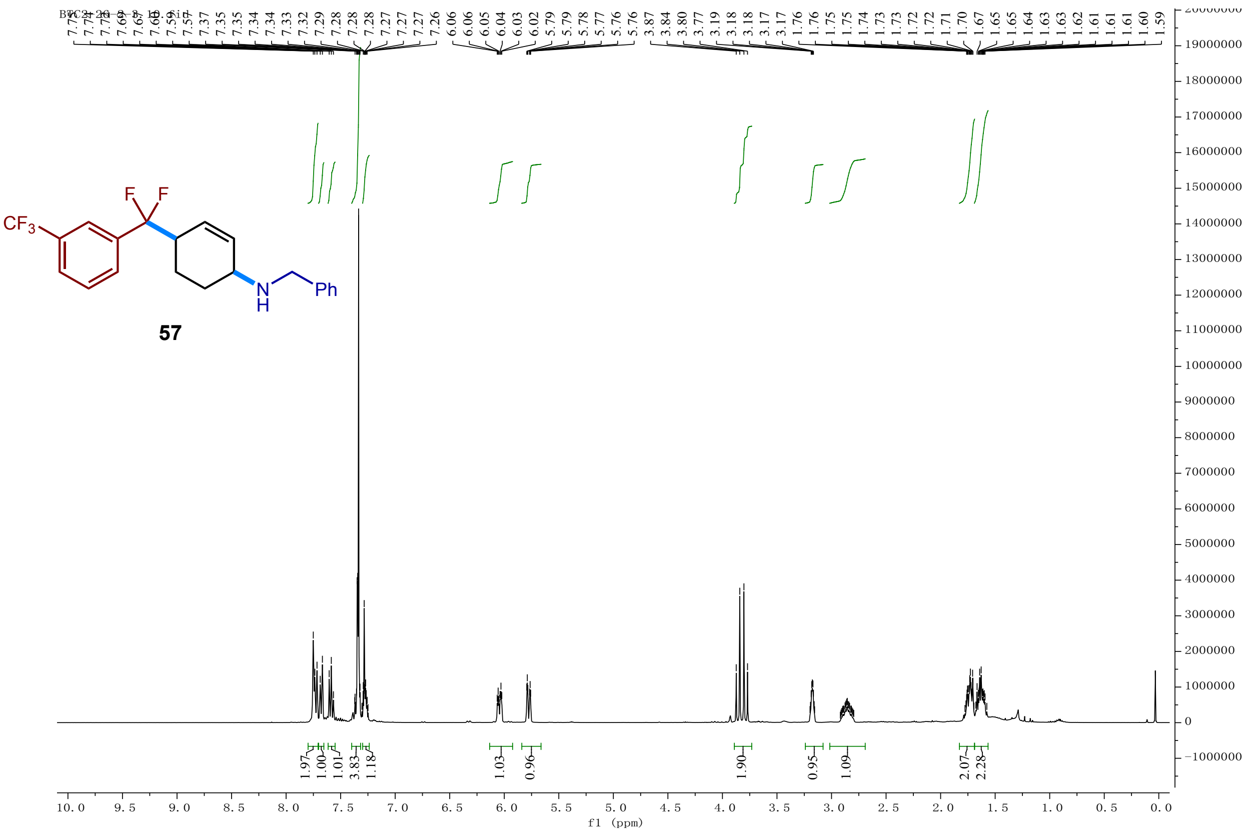
BTC2-26-2-3 [illegible]
7.74
7.69
7.67
7.60
7.59
7.57
7.37
7.35
7.35
7.34
7.34
7.33
7.32
7.29
7.28
7.28
7.28
7.27
7.27
7.27
7.26
6.06
6.06
6.05
6.04
6.03
6.02
5.79
5.79
5.78
5.77
5.76
3.87
3.84
3.80
3.77
3.19
3.18
3.17
3.17
1.76
1.76
1.75
1.75
1.74
1.73
1.73
1.72
1.72
1.71
1.70
1.67
1.65
1.64
1.63
1.63
1.62
1.61
1.61
1.60
1.59
CF3
F F
N
H
Ph
57
1.97
1.00
1.01
3.83
1.18
1.03
0.96
1.90
0.95
1.09
2.07
2.28
f1 (ppm)

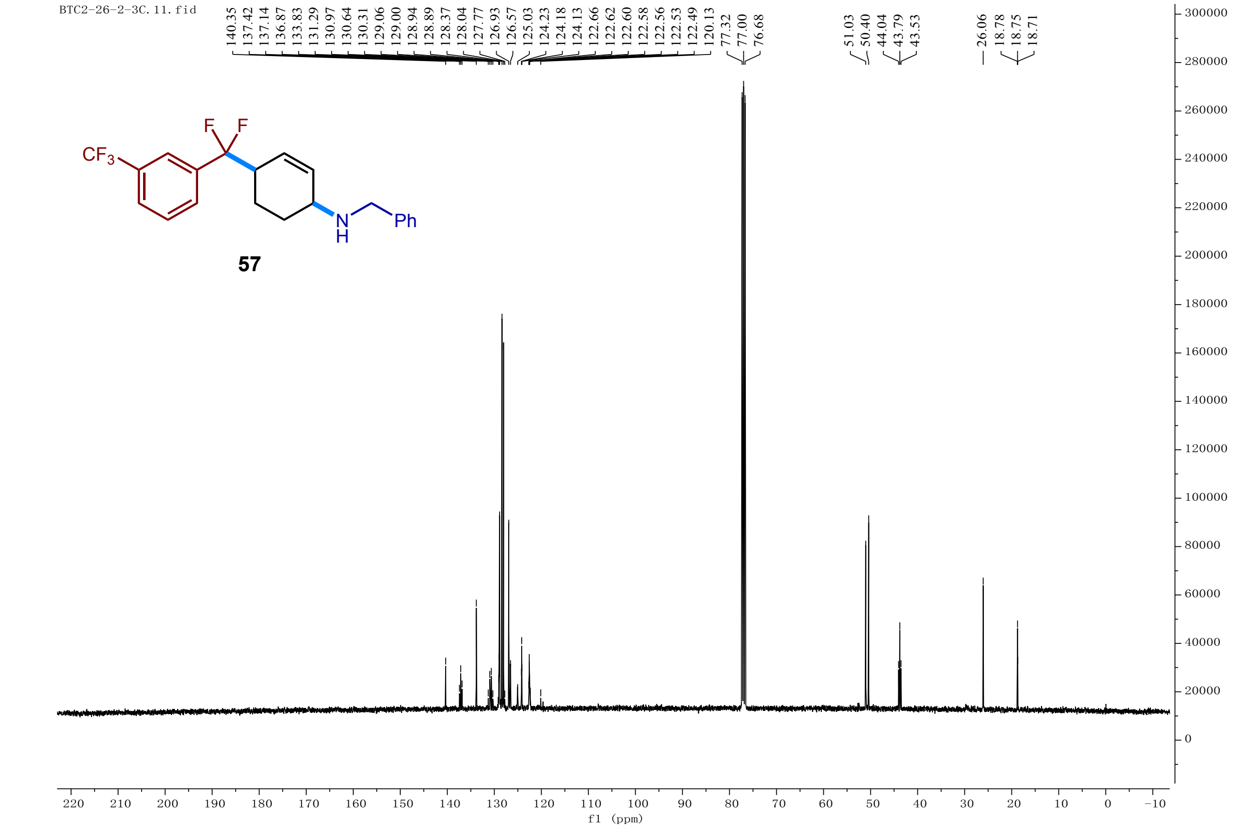
BTC2-26-2-3C.11.fid
140.35
137.42
137.14
136.87
133.83
131.29
130.97
130.64
130.31
129.06
129.00
128.94
128.89
128.37
128.04
127.77
126.93
126.57
125.03
124.23
124.18
124.13
122.66
122.62
122.60
122.58
122.56
122.53
122.49
120.13
77.32
77.00
76.68
51.03
50.40
44.04
43.79
43.53
26.06
18.78
18.75
18.71
CF3
F F
N
H
Ph
57
f1 (ppm)

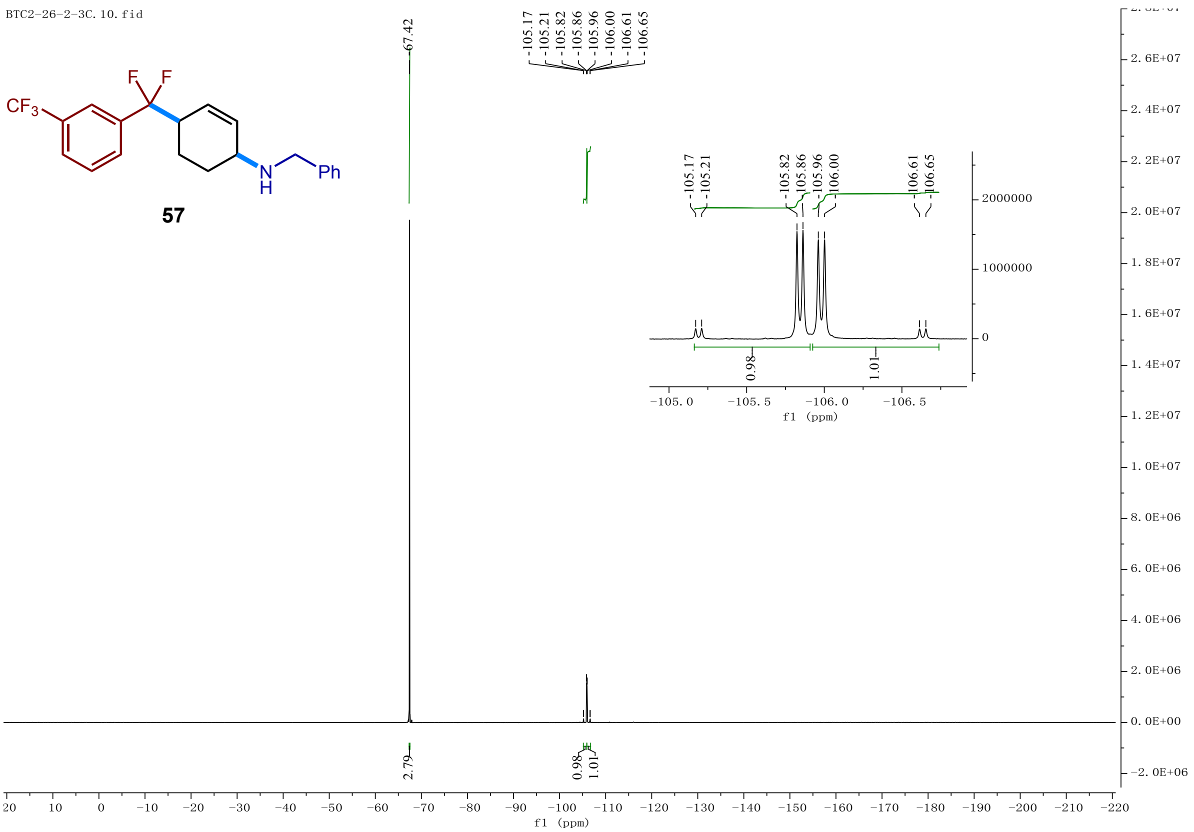
BTC2-26-2-3C.10.fid
CF3
F
F
N
H
Ph
57
-67.42
f1 (ppm)

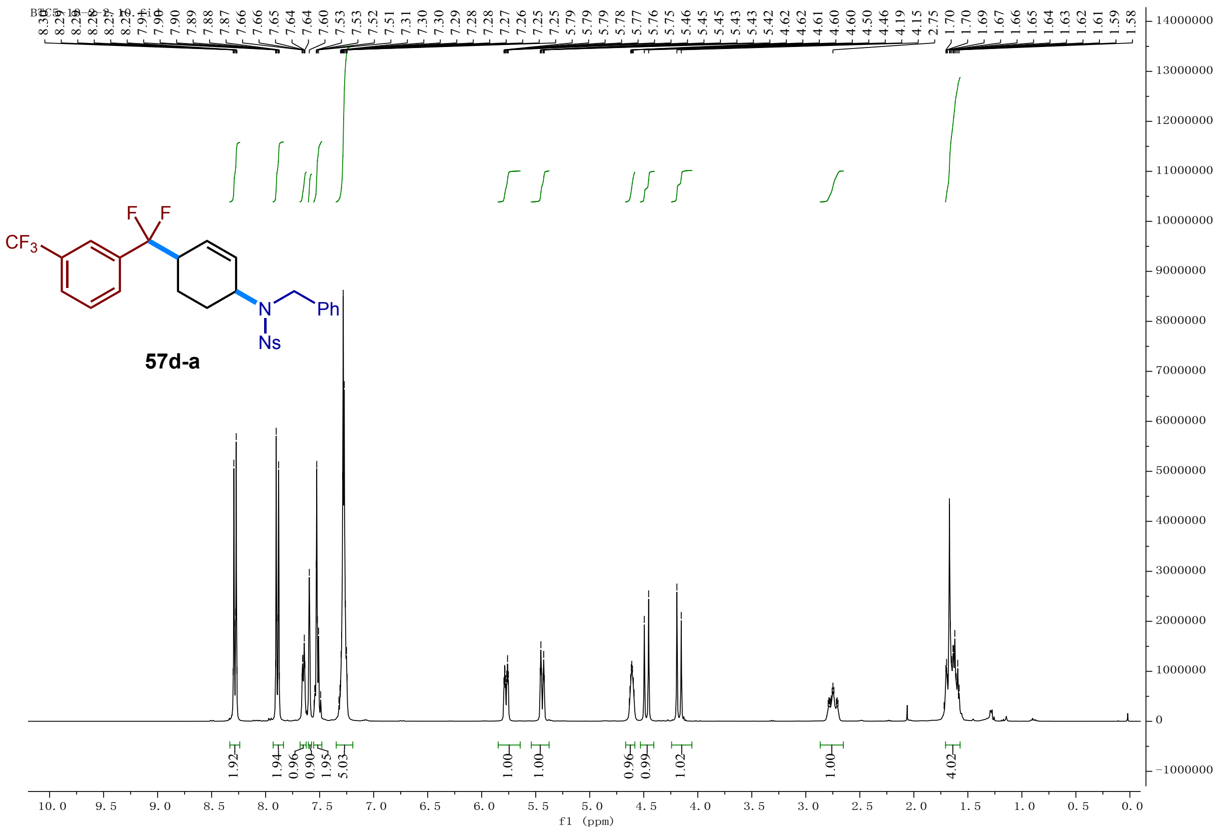
CF3
F
F
N
Ns
Ph
57d-a
f1 (ppm)

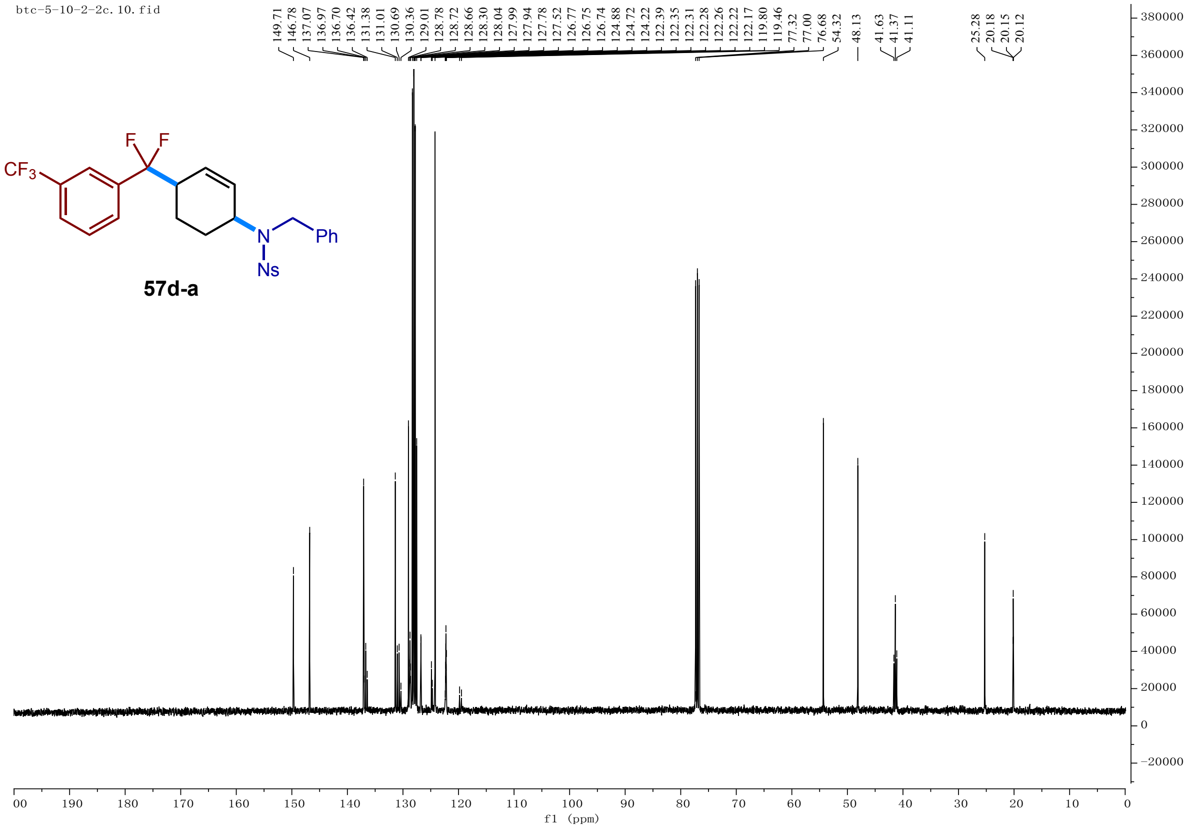
btc-5-10-2-2c.10.fid
149.71
146.78
137.07
136.97
136.70
136.42
131.38
131.01
130.69
130.36
129.01
128.78
128.72
128.66
128.30
128.04
127.99
127.94
127.78
127.52
126.77
126.75
126.74
124.88
124.72
124.22
122.39
122.35
122.31
122.28
122.26
122.22
122.17
119.80
119.46
77.32
77.00
76.68
54.32
48.13
41.63
41.37
41.11
25.28
20.18
20.15
20.12
CF3
F F
N
Ph
Ns
57d-a
f1 (ppm)

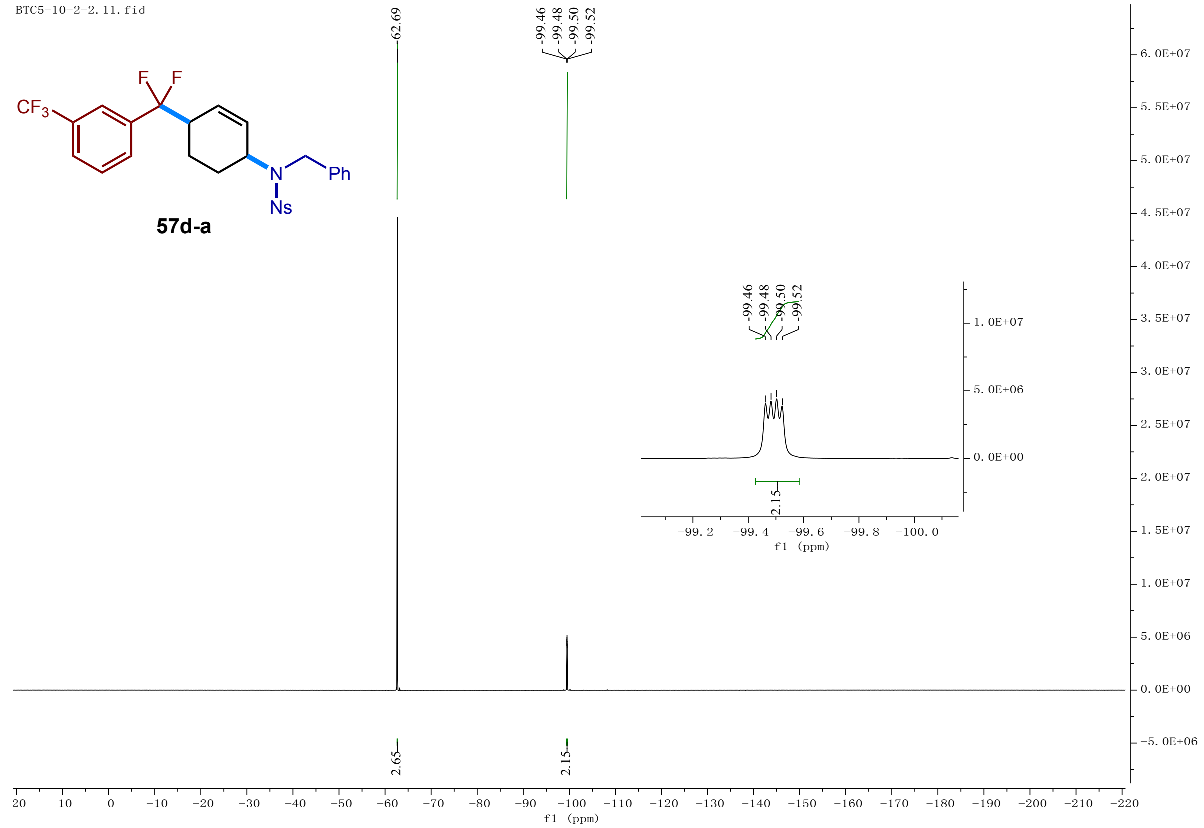
BTC5-10-2-2.11.fid
-62.69
-99.46
-99.48
-99.50
-99.52
CF3
F F
N
Ph
Ns
57d-a
2.15
2.65
f1 (ppm)

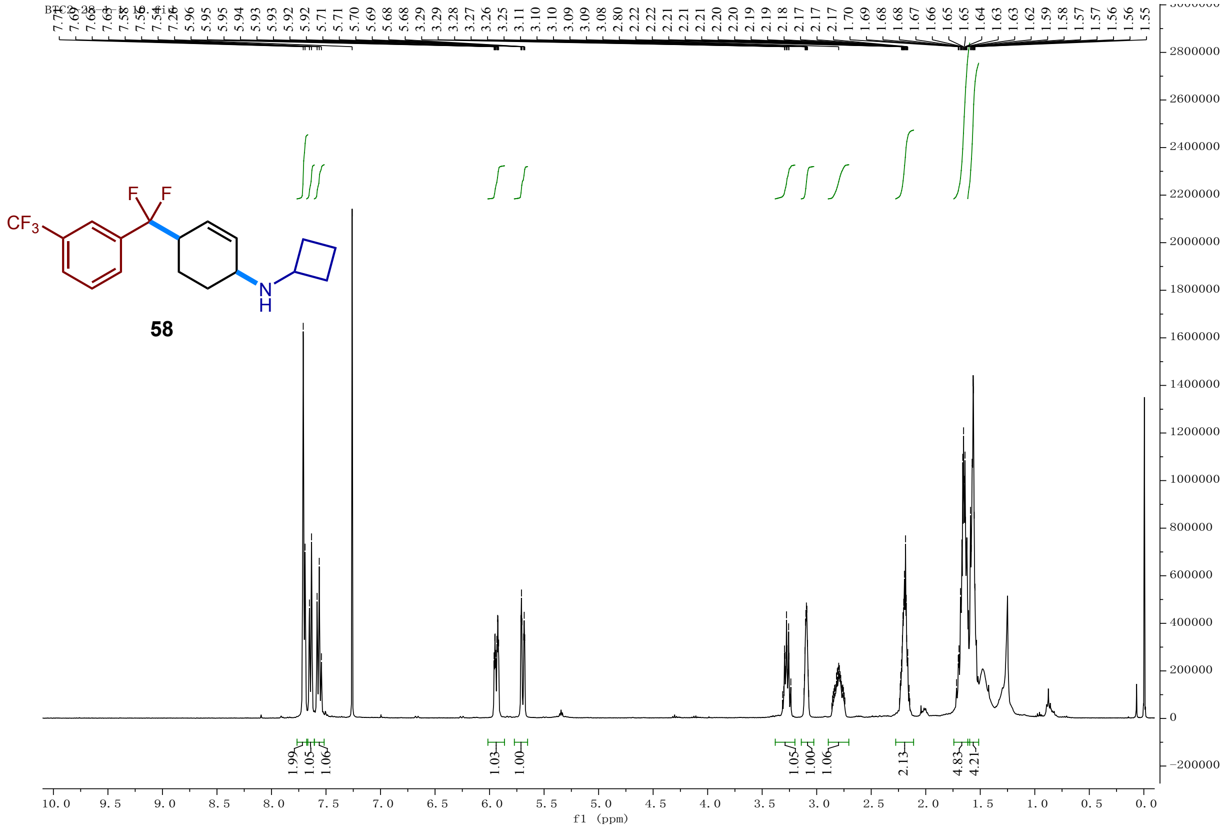

CF3
F F
N
H
58
7.69
7.58
7.56
7.54
7.26
5.96 5.95 5.95 5.94 5.93 5.93 5.92 5.92 5.71 5.71 5.70 5.69 5.68 5.68 3.29 3.29 3.28 3.27 3.26 3.25 3.11 3.10 3.10 3.09 3.09 3.08 2.80 2.22 2.22 2.21 2.21 2.21 2.20 2.20 2.19 2.19 2.18 2.17 2.17 2.17 1.70 1.69 1.68 1.68 1.67 1.66 1.65 1.65 1.64 1.63 1.63 1.62 1.59 1.58 1.57 1.57 1.56 1.55
1.99 1.05 1.06 1.03 1.00 1.05 1.00 1.06 2.13 4.83 4.21
f1 (ppm)

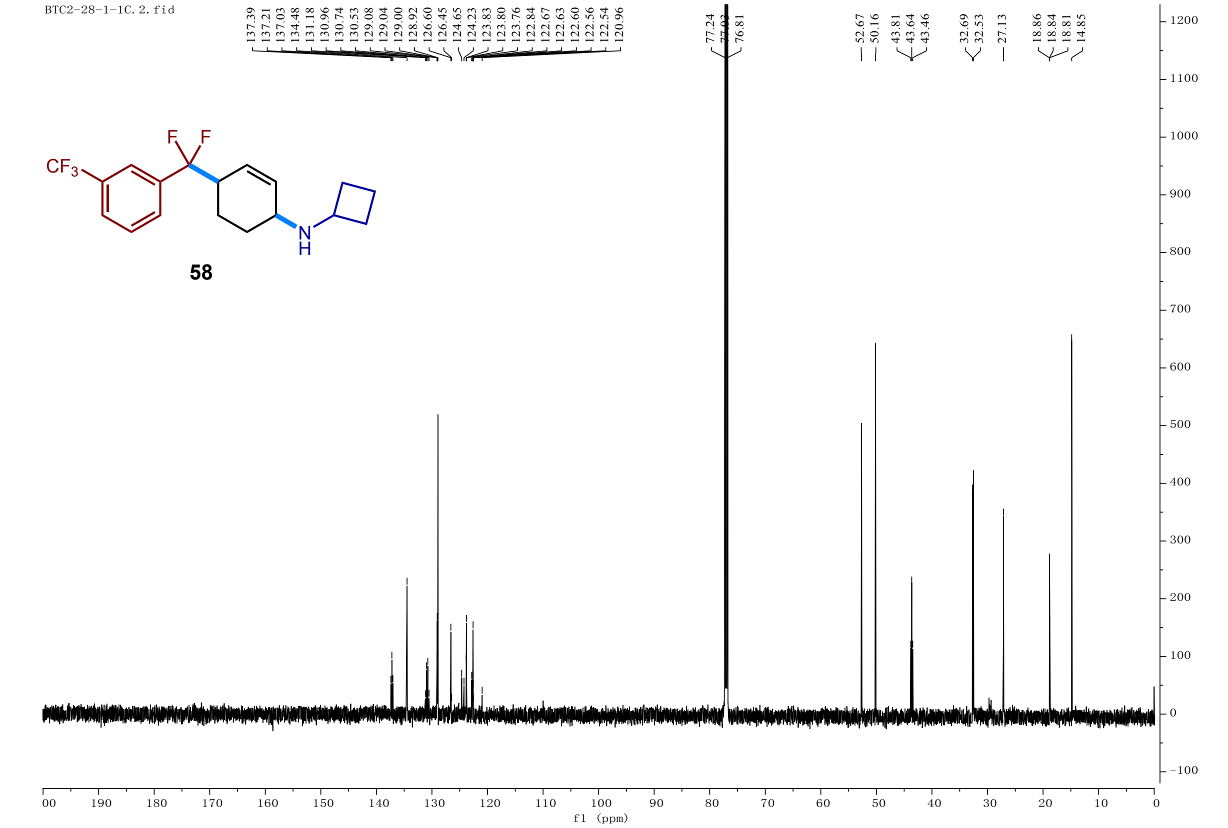

BTC2-28-1-1C.2.fid
137.39 137.21 137.03 134.48 131.18 130.96 130.74 130.53 129.08 129.04 129.00 128.92 126.60 126.45 124.65 124.23 123.83 123.80 123.76 122.84 122.67 122.63 122.60 122.56 122.54 120.96
77.24 76.81
52.67 50.16 43.81 43.64 43.46 32.69 32.53 27.13 18.86 18.84 18.81 14.85
CF3
F F
N
H
58
f1 (ppm)

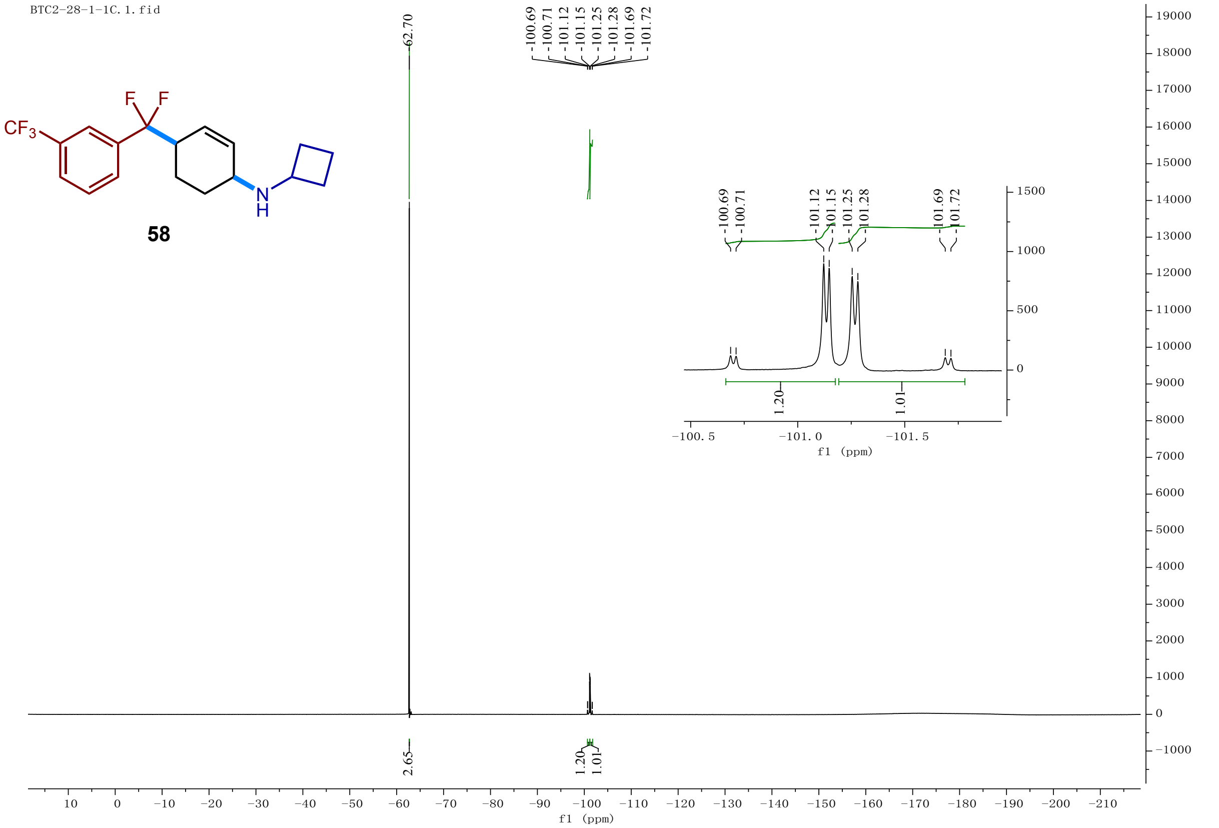
BTC2-28-1-1C.1.fid
CF3
F
F
N
H
58
-62.70
-100.69
-100.71
-101.12
-101.15
-101.25
-101.28
-101.69
-101.72
2.65
1.20
1.01
f1 (ppm)

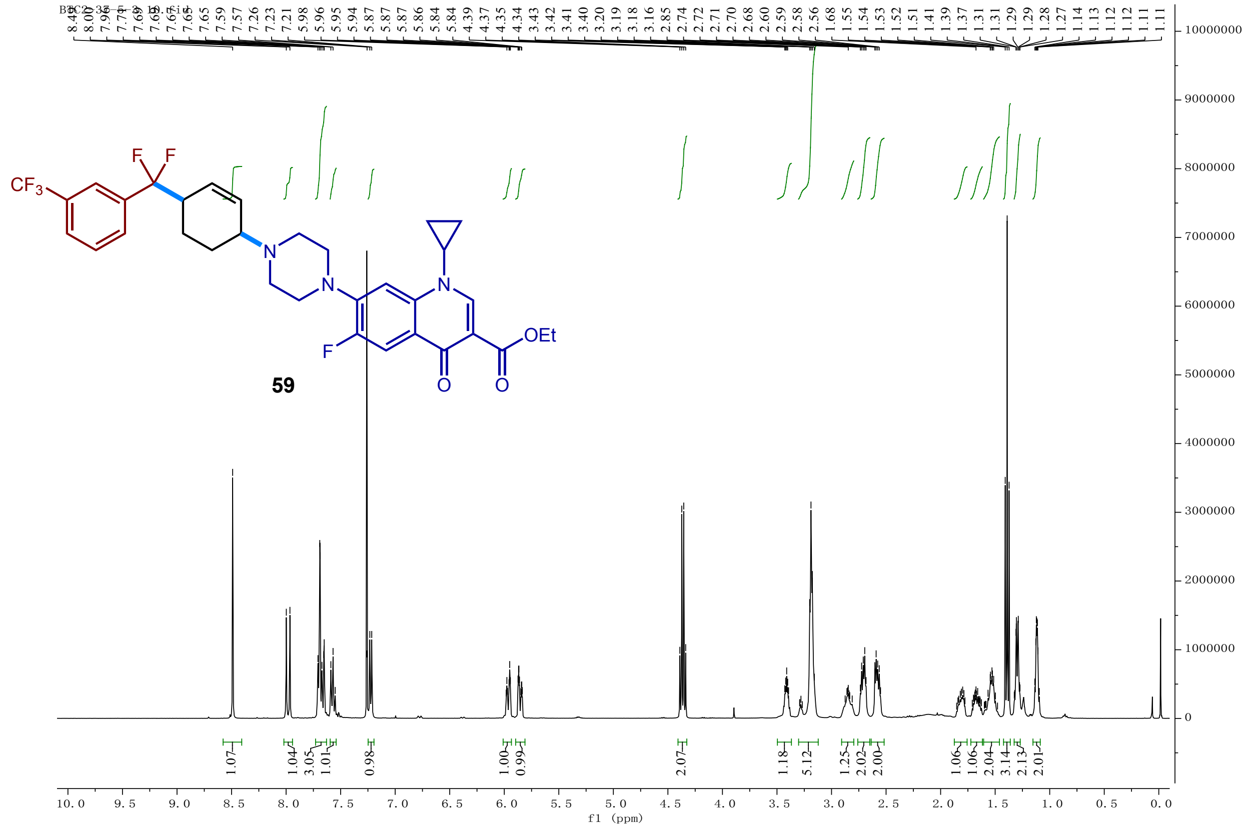
CF3
F
F
N
N
N
F
O
O
OEt
59
f1 (ppm)

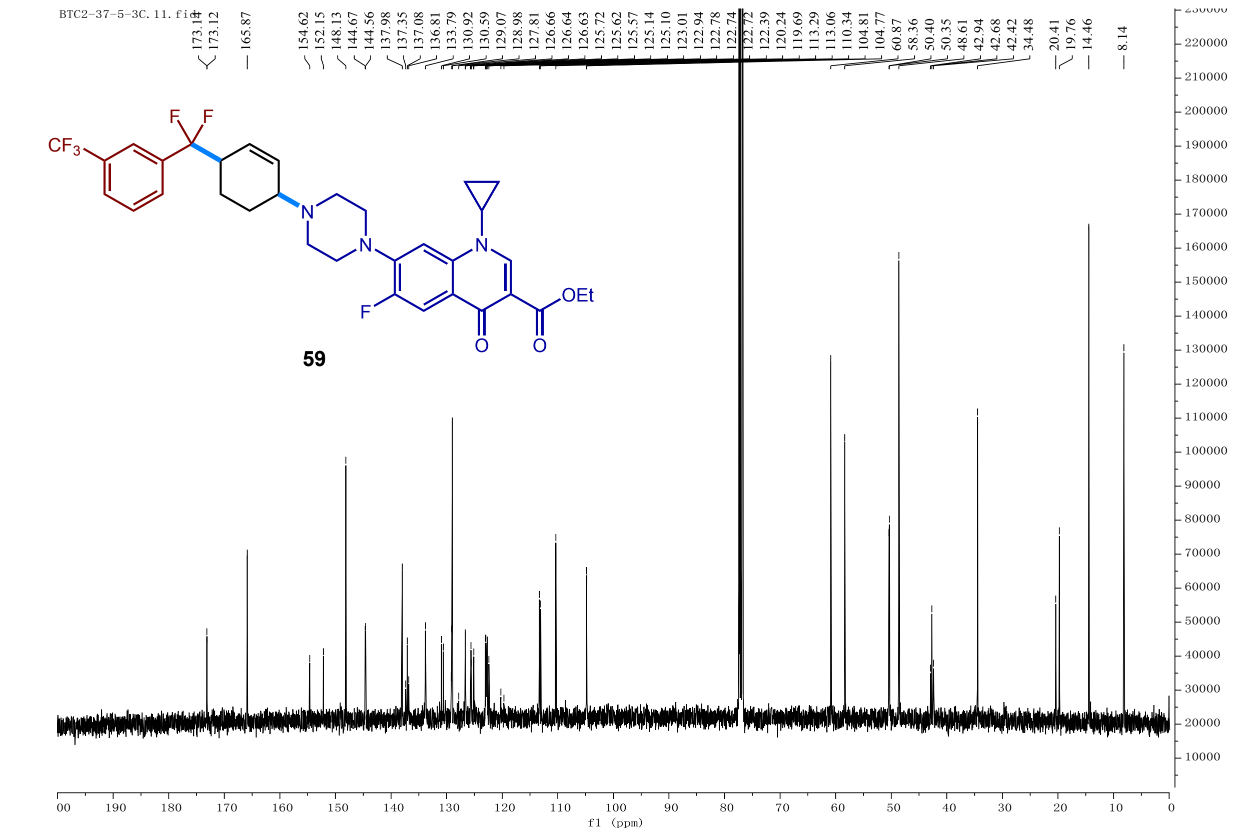

BTC2-37-5-3C.11.fid
173.14
173.12
165.87
154.62
152.15
148.13
144.67
144.56
137.98
137.35
137.08
136.81
133.79
130.92
130.59
129.07
128.98
127.81
126.66
126.64
126.63
125.72
125.62
125.57
125.14
125.10
123.01
122.94
122.78
122.74
122.72
122.39
120.24
119.69
113.29
113.06
110.34
104.81
104.77
60.87
58.36
50.40
50.35
48.61
42.94
42.68
42.42
34.48
20.41
19.76
14.46
8.14
CF3
F F
N
N
N
F
O
O
OEt
59
f1 (ppm)

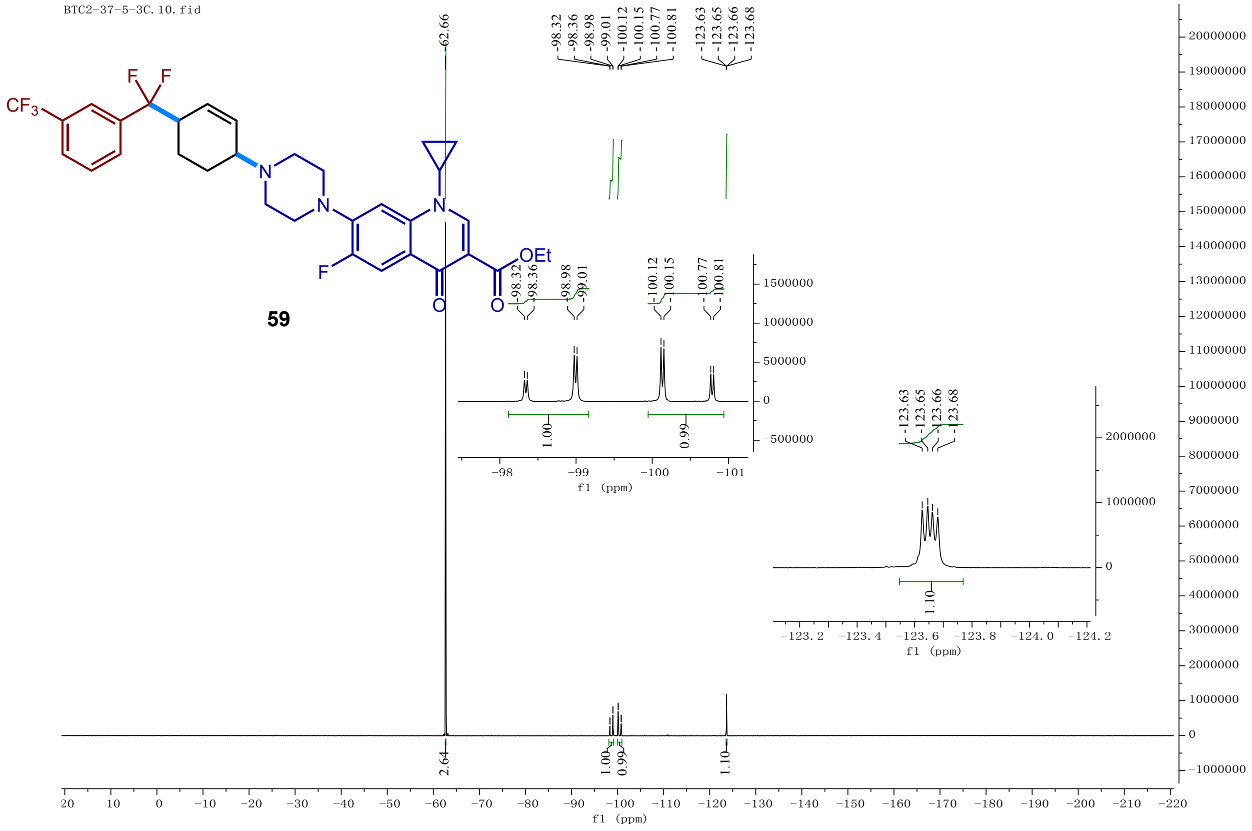

BTC2-37-5-3C.10.fid
-62.66
-98.32
-98.36
-98.98
-99.01
-100.12
-100.15
-100.77
-100.81
-123.63
-123.65
-123.66
-123.68
CF3
F F
N
N
N
F
O
O
OEt
59
1.00
0.99
1.10
2.64
f1 (ppm)

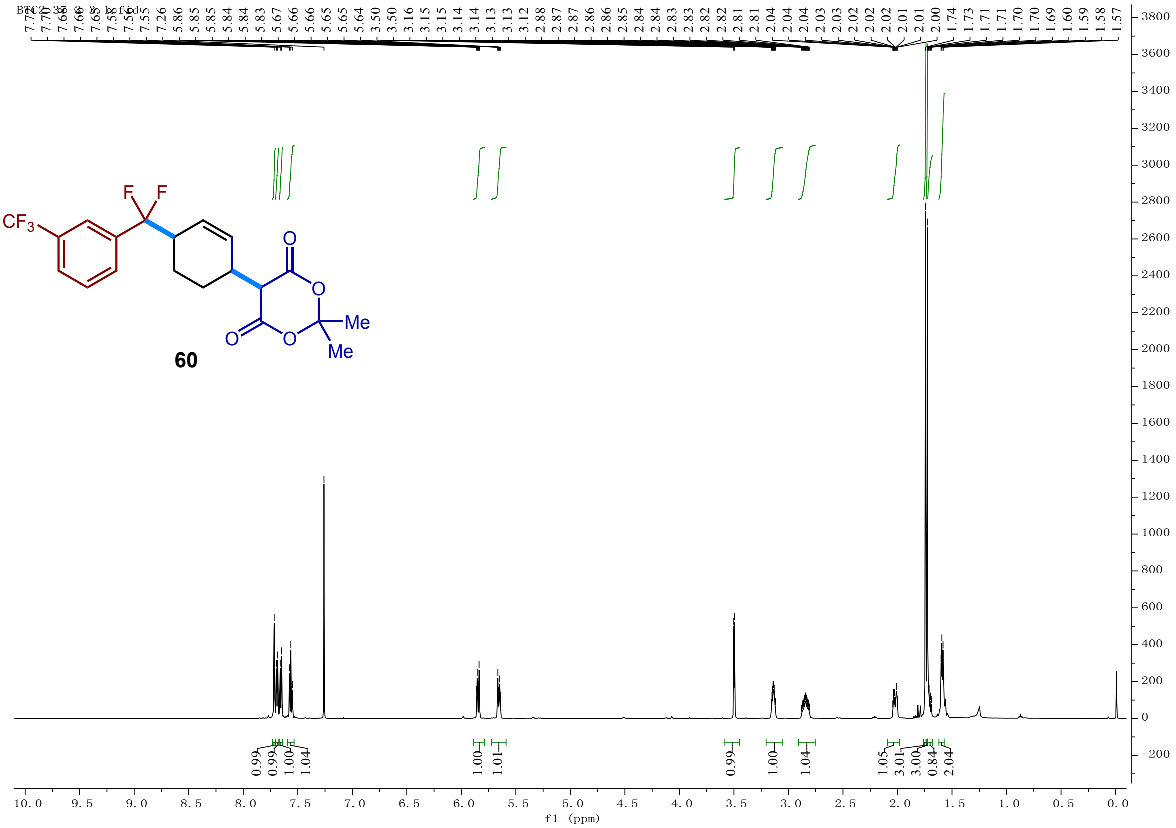
7.26
5.86
5.85
5.85
5.84
5.84
5.83
5.67
5.66
5.66
5.65
5.65
5.64
3.50
3.50
3.16
3.15
3.15
3.14
3.14
3.13
3.13
3.12
2.88
2.87
2.87
2.86
2.86
2.85
2.85
2.84
2.84
2.83
2.83
2.82
2.82
2.81
2.81
2.04
2.04
2.04
2.03
2.03
2.02
2.02
2.02
2.01
2.01
2.00
1.74
1.73
1.71
1.71
1.70
1.69
1.60
1.59
1.58
1.57
CF3
F
F
O
O
O
O
Me
Me
60
0.99
0.99
1.00
1.04
1.00
1.01
0.99
1.00
1.04
1.05
3.01
3.00
0.84
2.04
f1 (ppm)

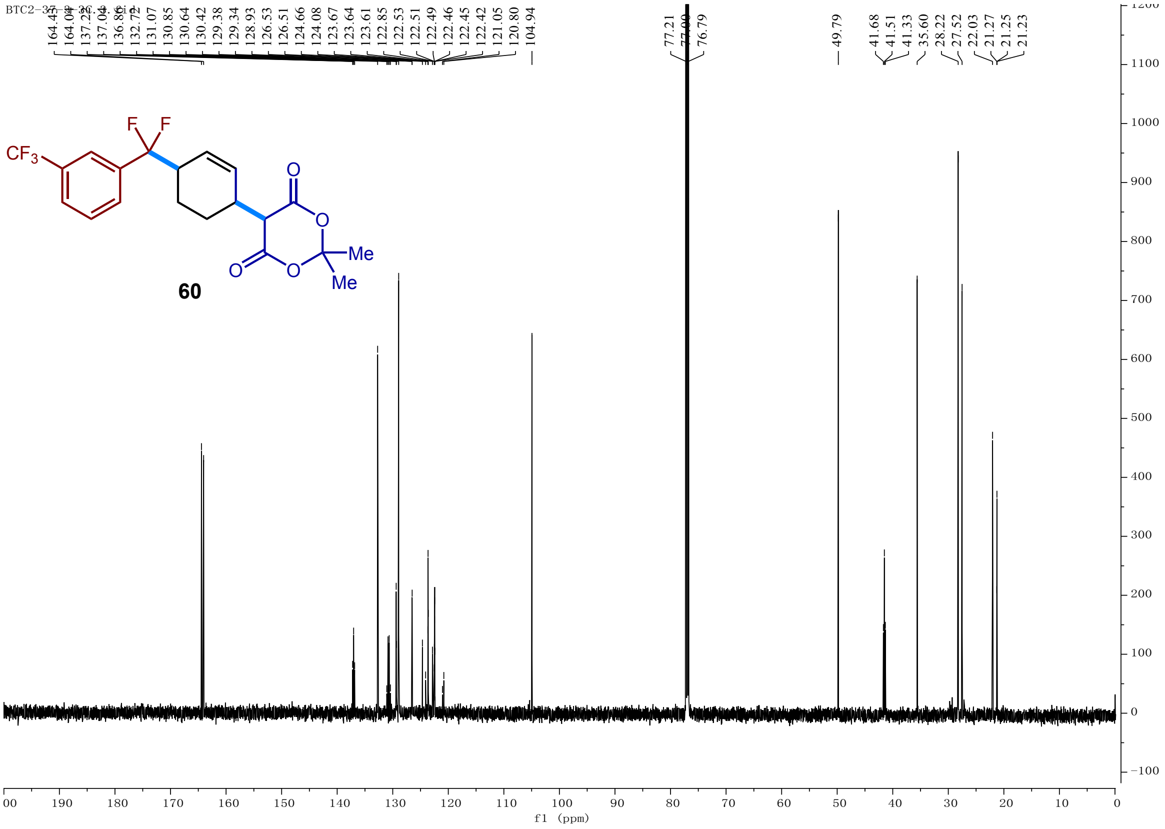
137.04
132.72
131.07
130.85
130.64
130.42
129.38
129.34
128.93
126.53
126.51
124.66
124.08
123.67
123.64
123.61
122.85
122.53
122.51
122.49
122.46
122.45
122.42
121.05
120.80
104.94
77.21
77.00
76.79
49.79
41.68
41.51
41.33
35.60
28.22
27.52
22.03
21.27
21.25
21.23
CF3
F
F
O
O
O
O
Me
Me
60
f1 (ppm)

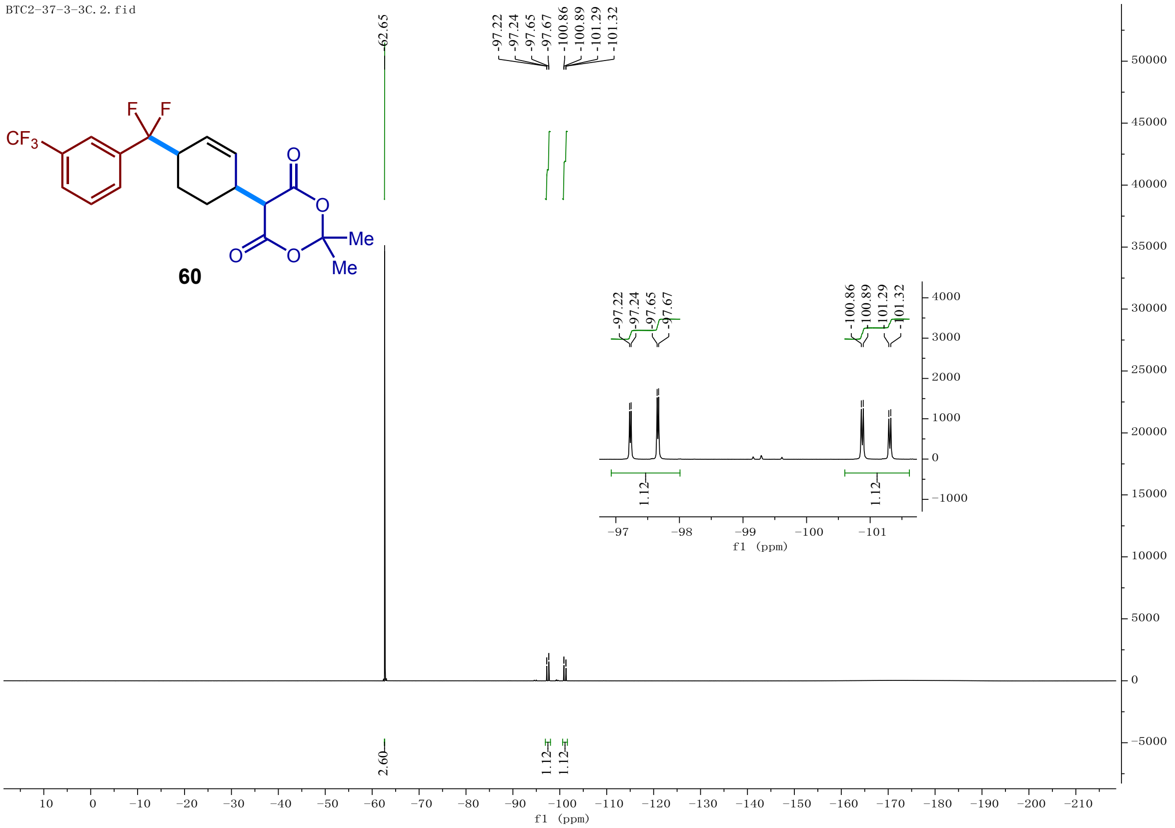
BTC2-37-3-3C.2.fid
-62.65
-97.22
-97.24
-97.65
-97.67
-100.86
-100.89
-101.29
-101.32
60
2.60
1.12
1.12
f1 (ppm)

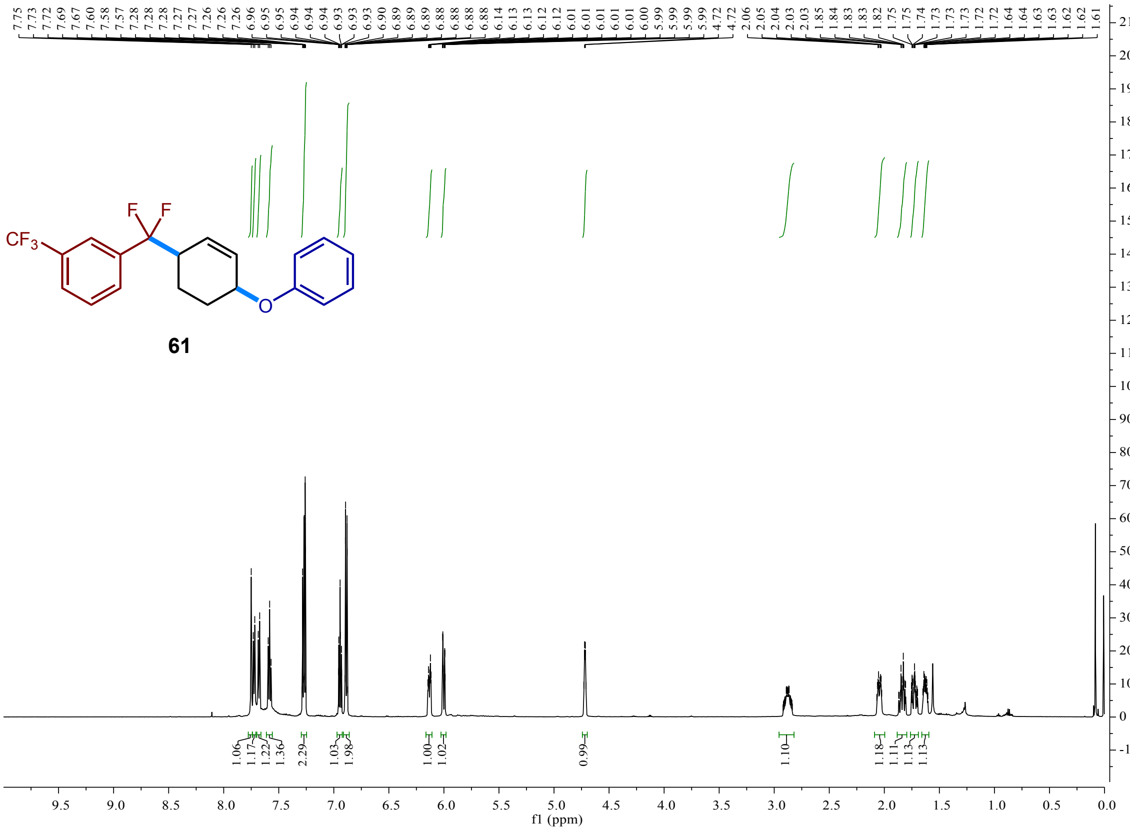
61
1.06
1.17
1.22
1.36
2.29
1.03
1.98
1.00
1.02
0.99
1.10
1.18
1.11
1.13
1.13
f1 (ppm)

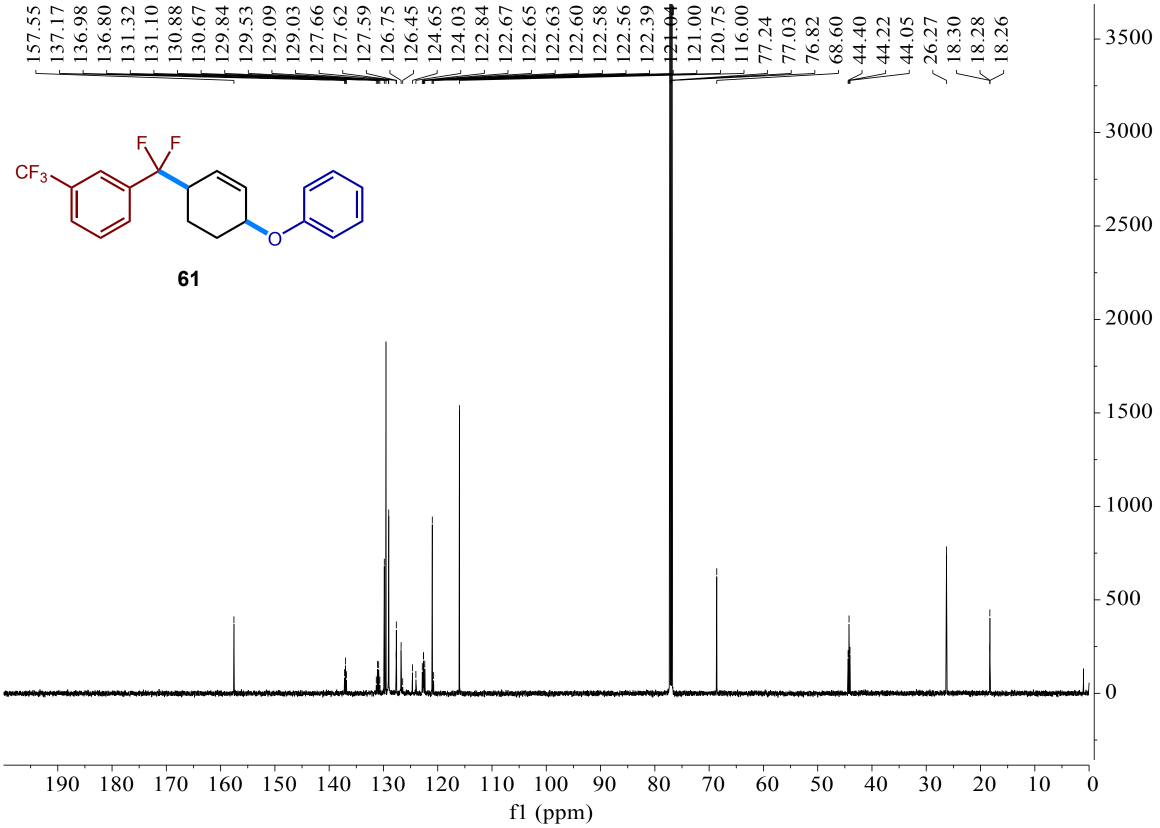
157.55
137.17
136.98
136.80
131.32
131.10
130.88
130.67
129.84
129.53
129.09
129.03
127.66
127.62
127.59
126.75
126.45
124.65
124.03
122.84
122.67
122.65
122.63
122.60
122.58
122.56
122.39
121.04
121.00
120.75
116.00
77.24
77.03
76.82
68.60
44.40
44.22
44.05
26.27
18.30
18.28
18.26
CF3
F
F
O
61
f1 (ppm)

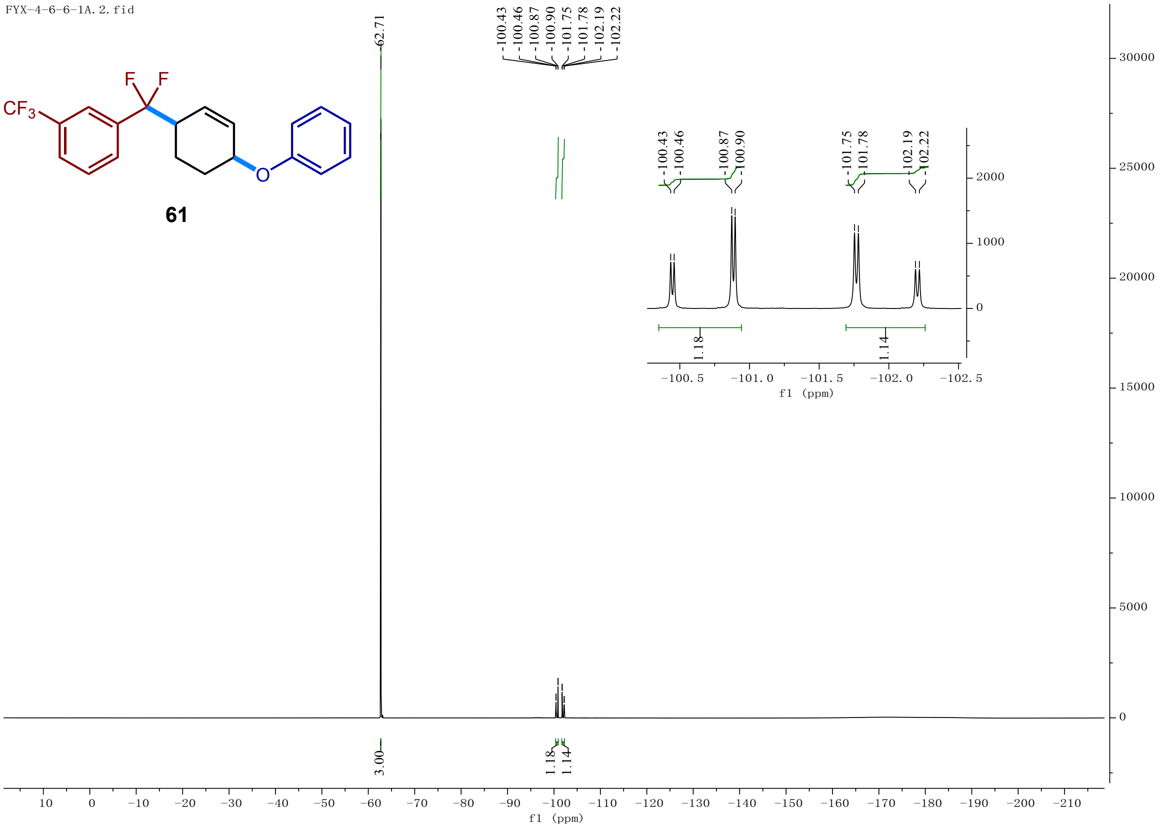
FYX-4-6-6-1A.2.fid
-62.71
-100.43
-100.46
-100.87
-100.90
-101.75
-101.78
-102.19
-102.22
CF3
F
F
O
61
1.18
1.14
3.00
f1 (ppm)

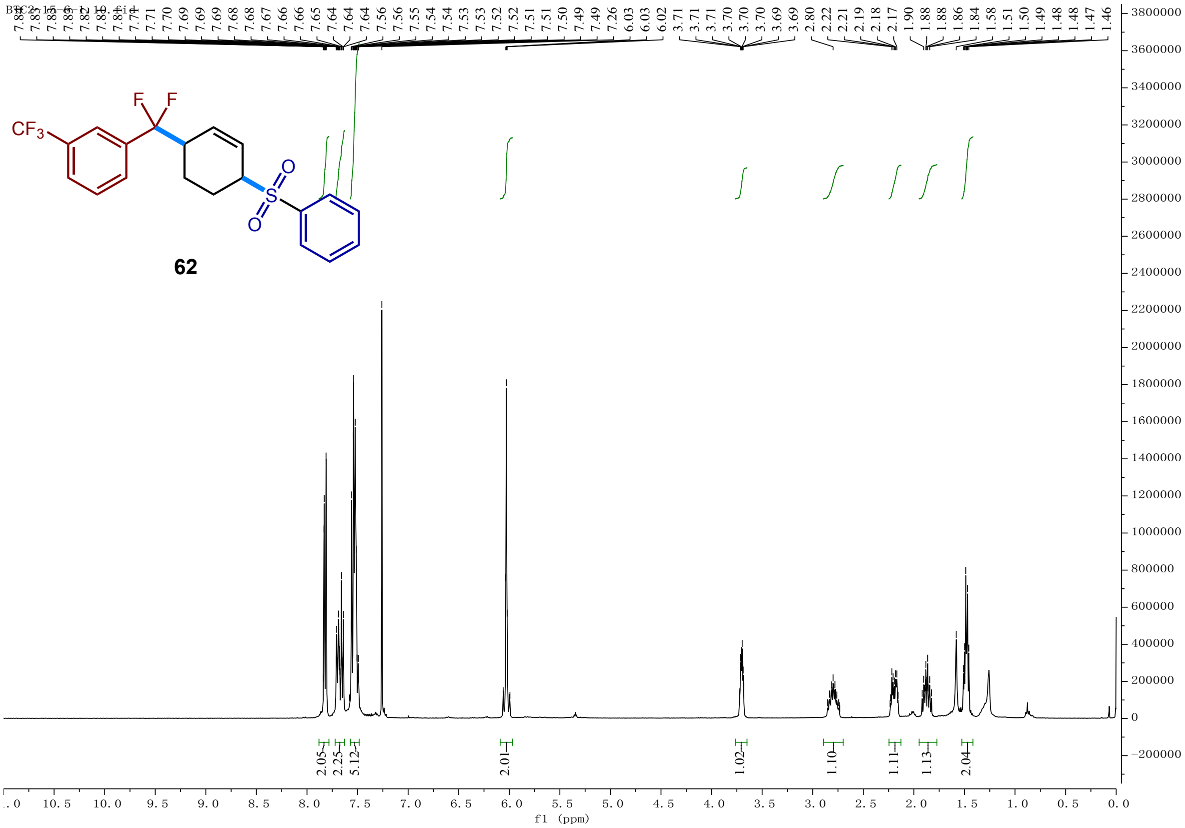
7.71 7.70 7.69 7.69 7.69 7.68 7.68 7.67 7.66 7.66 7.65 7.64 7.64 7.64 7.56 7.56 7.55 7.54 7.54 7.53 7.53 7.52 7.52 7.51 7.51 7.50 7.49 7.49 7.26 6.03 6.02 3.71 3.71 3.71 3.70 3.70 3.69 3.69 2.80 2.22 2.21 2.19 2.18 2.17 1.90 1.88 1.88 1.86 1.84 1.58 1.51 1.50 1.49 1.48 1.48 1.47 1.46
CF3
F
F
O
S
O
62
2.05
2.25
5.12
2.01
1.02
1.10
1.11
1.13
2.04
f1 (ppm)

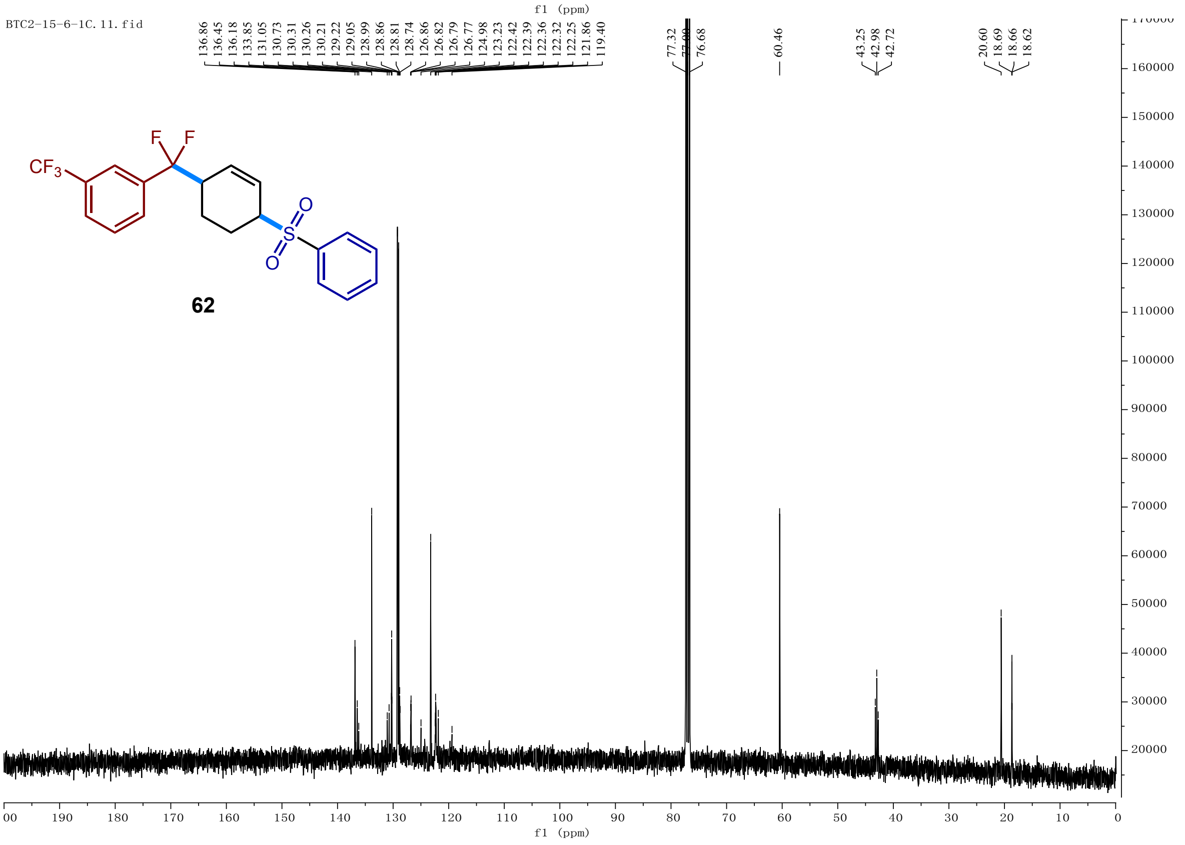
BTC2-15-6-1C. 11. fid
136.86 136.45 136.18 133.85 131.05 130.73 130.31 130.26 130.21 129.22 129.05 128.99 128.86 128.81 128.74 126.86 126.82 126.79 126.77 124.98 123.23 122.42 122.39 122.36 122.32 122.25 121.86 119.40
77.32 76.68
60.46
43.25 42.98 42.72
20.60 18.69 18.66 18.62
CF3
F
F
O
S
O
62
f1 (ppm)

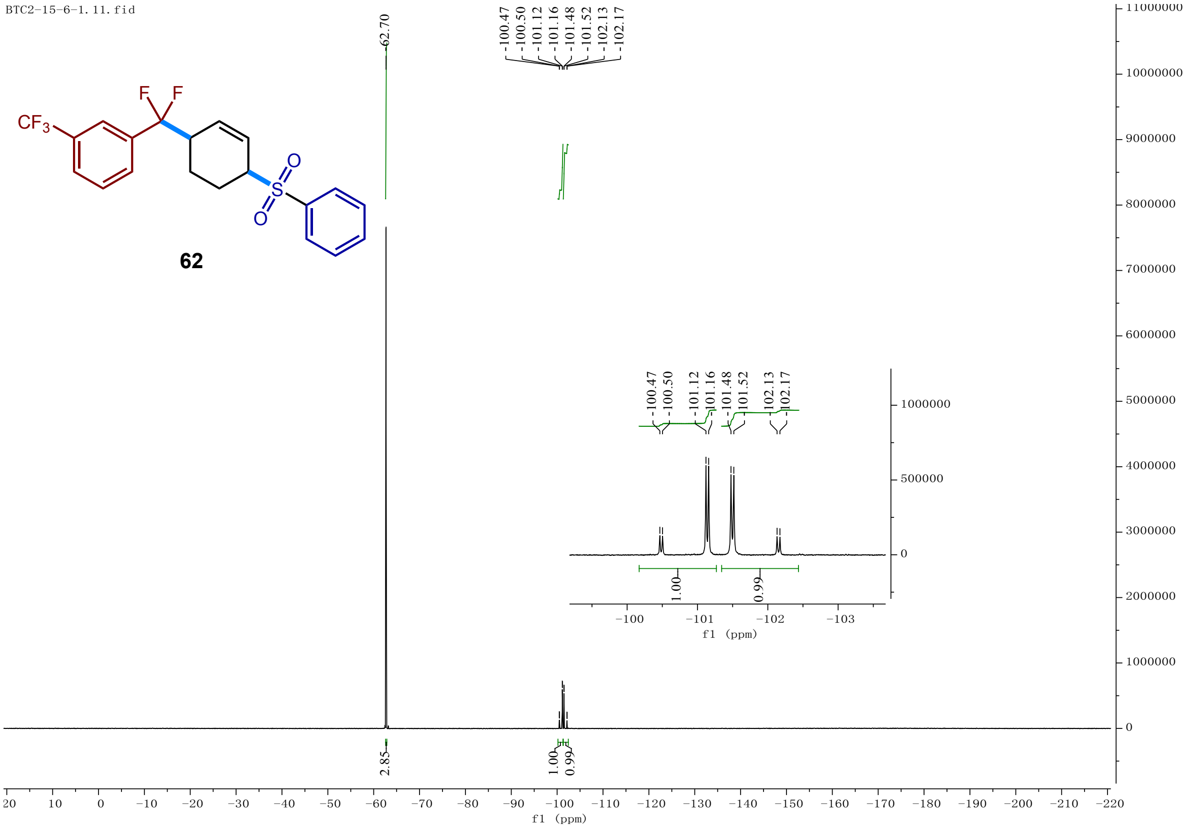
BTC2-15-6-1.11.fid
CF3
F F
O
S
O
62
-62.70
-100.47
-100.50
-101.12
-101.16
-101.48
-101.52
-102.13
-102.17
1.00
0.99
2.85
f1 (ppm)

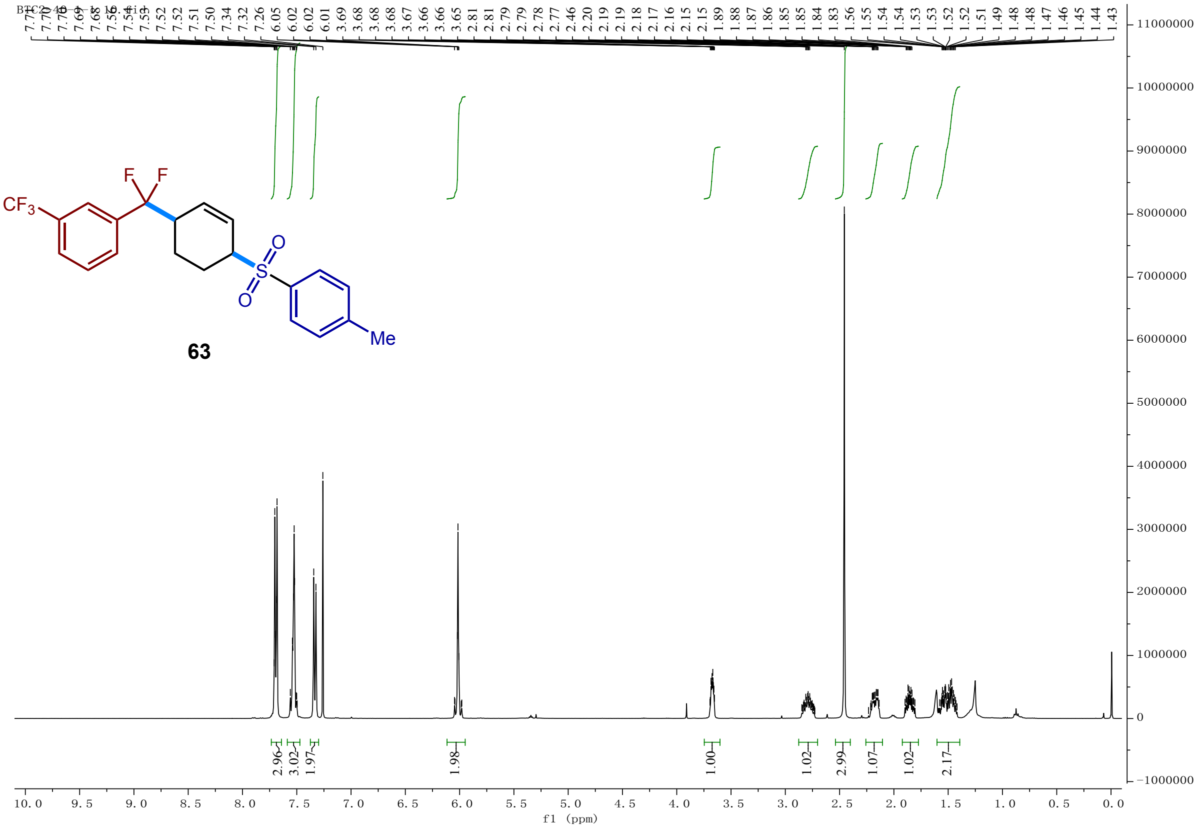
CF3
F F
O
S
O
Me
63
2.96
3.02
1.97
1.98
1.00
1.02
2.99
1.07
1.02
2.17
f1 (ppm)

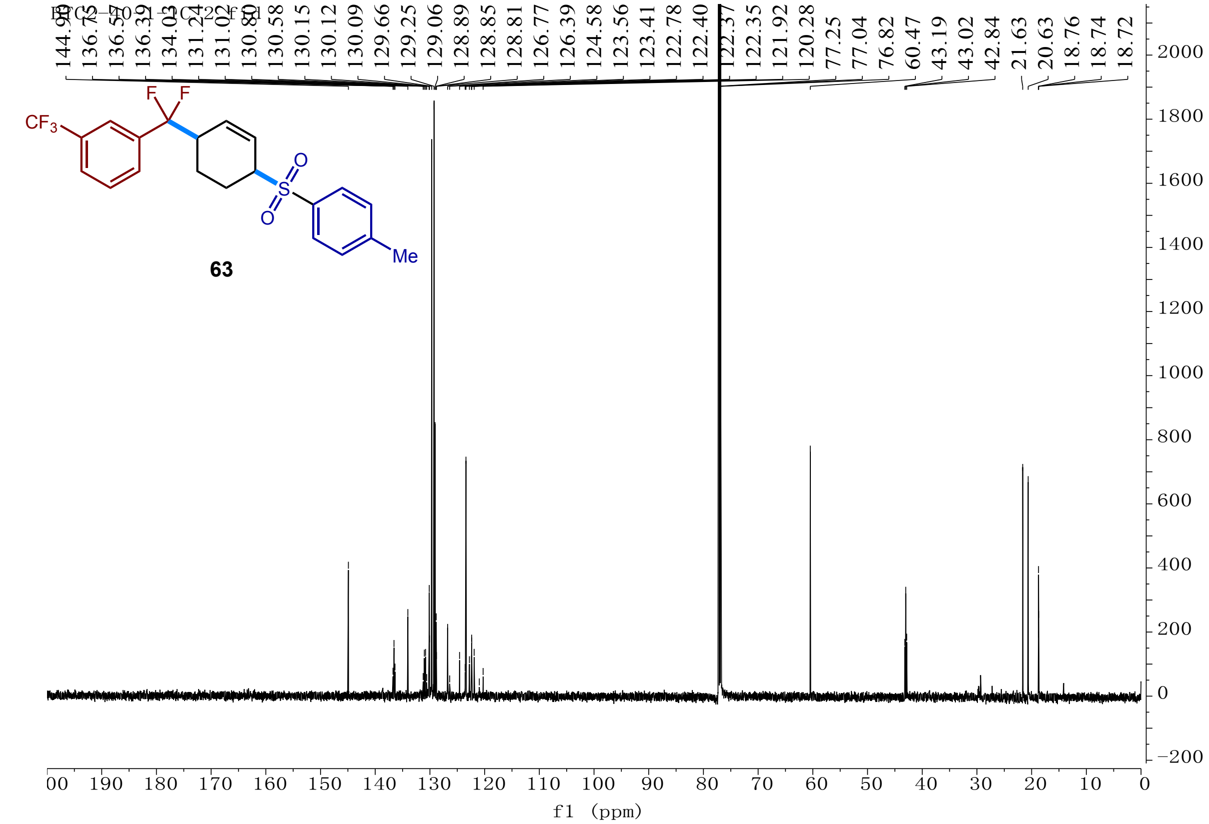
136.75
136.57
136.32
134.03
131.24
131.02
130.80
130.58
130.15
130.12
130.09
129.66
129.25
129.06
128.89
128.85
128.81
126.77
126.39
124.58
123.56
123.41
122.78
122.40
122.37
122.35
121.92
120.28
77.25
77.04
76.82
60.47
43.19
43.02
42.84
21.63
20.63
18.76
18.74
18.72
CF_3
F
F
O
S
O
Me
63
f1 (ppm)

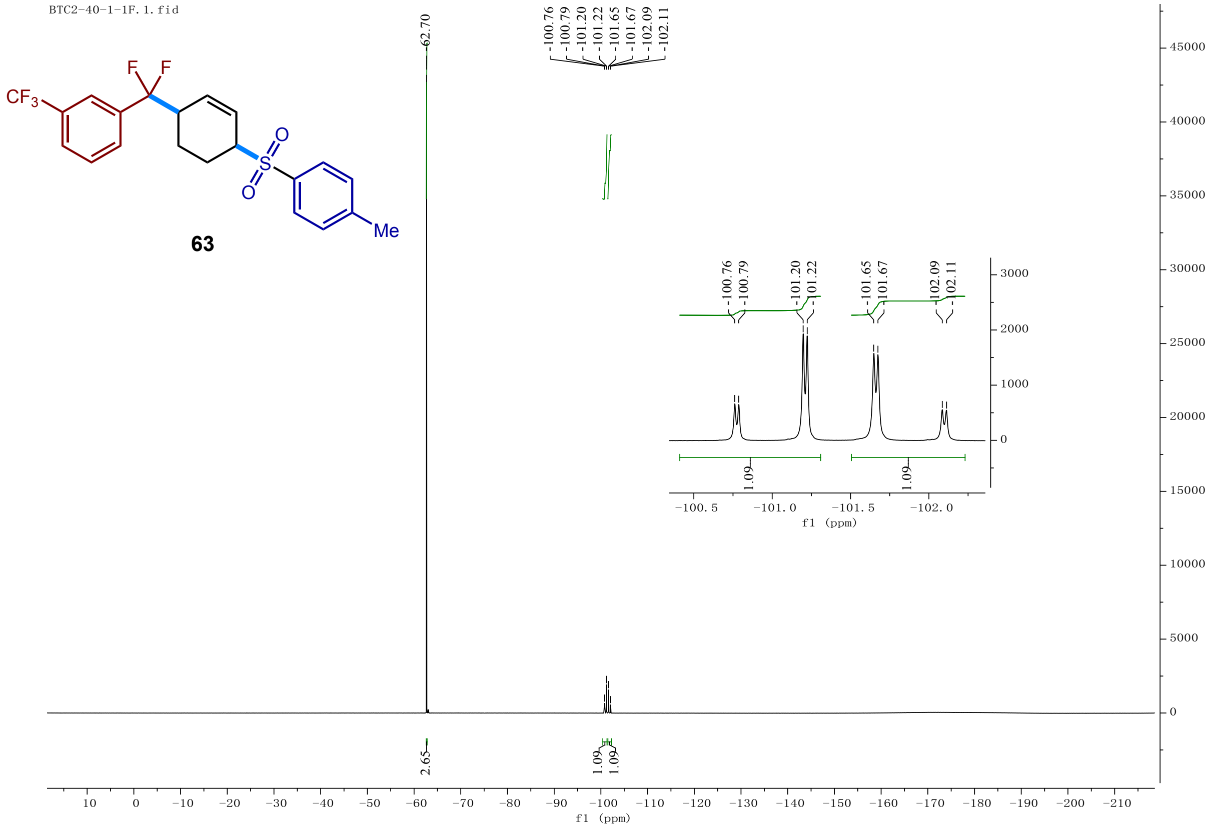
BTC2-40-1-1F.1.fid
-62.70
-100.76
-100.79
-101.20
-101.22
-101.65
-101.67
-102.09
-102.11
CF_3
F
F
O
S
O
Me
63
1.09
1.09
2.65
f1 (ppm)

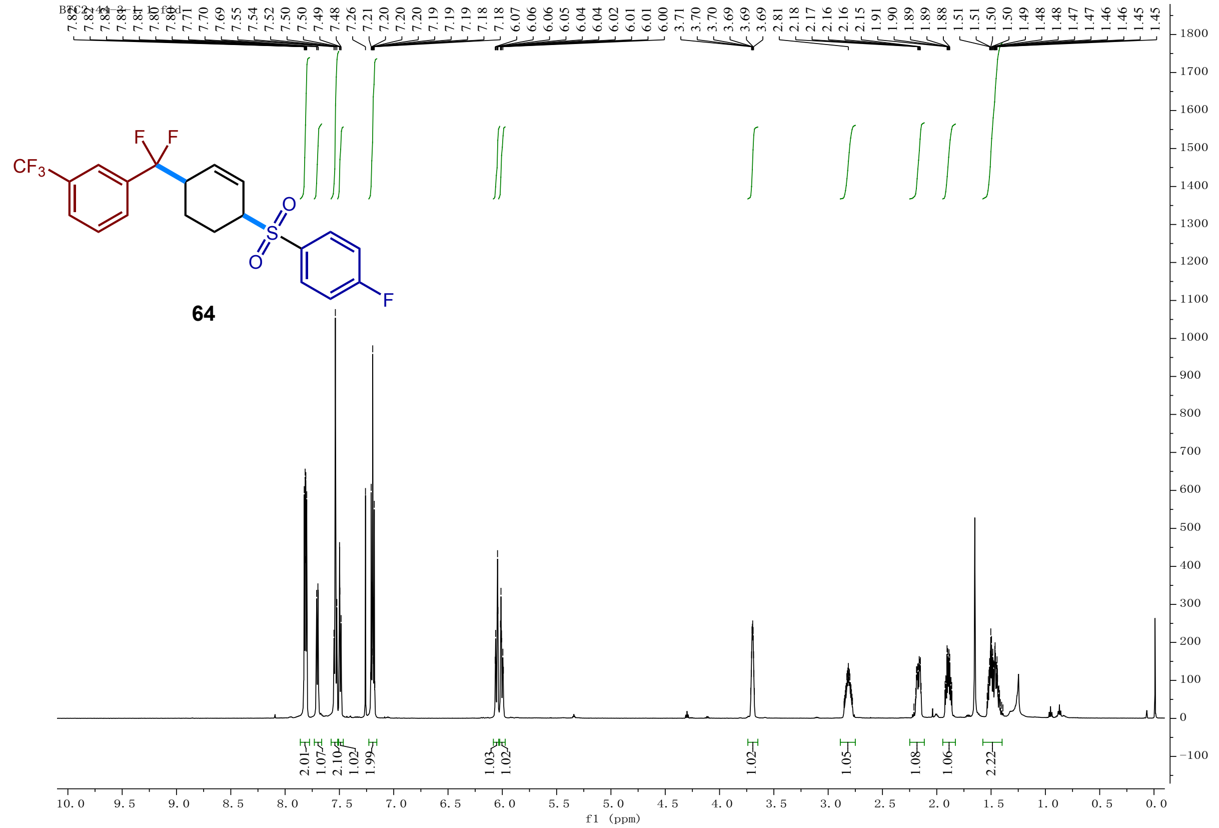
BTC2-144-3-1H
CF3
F F
O
S
O
F
64
f1 (ppm)

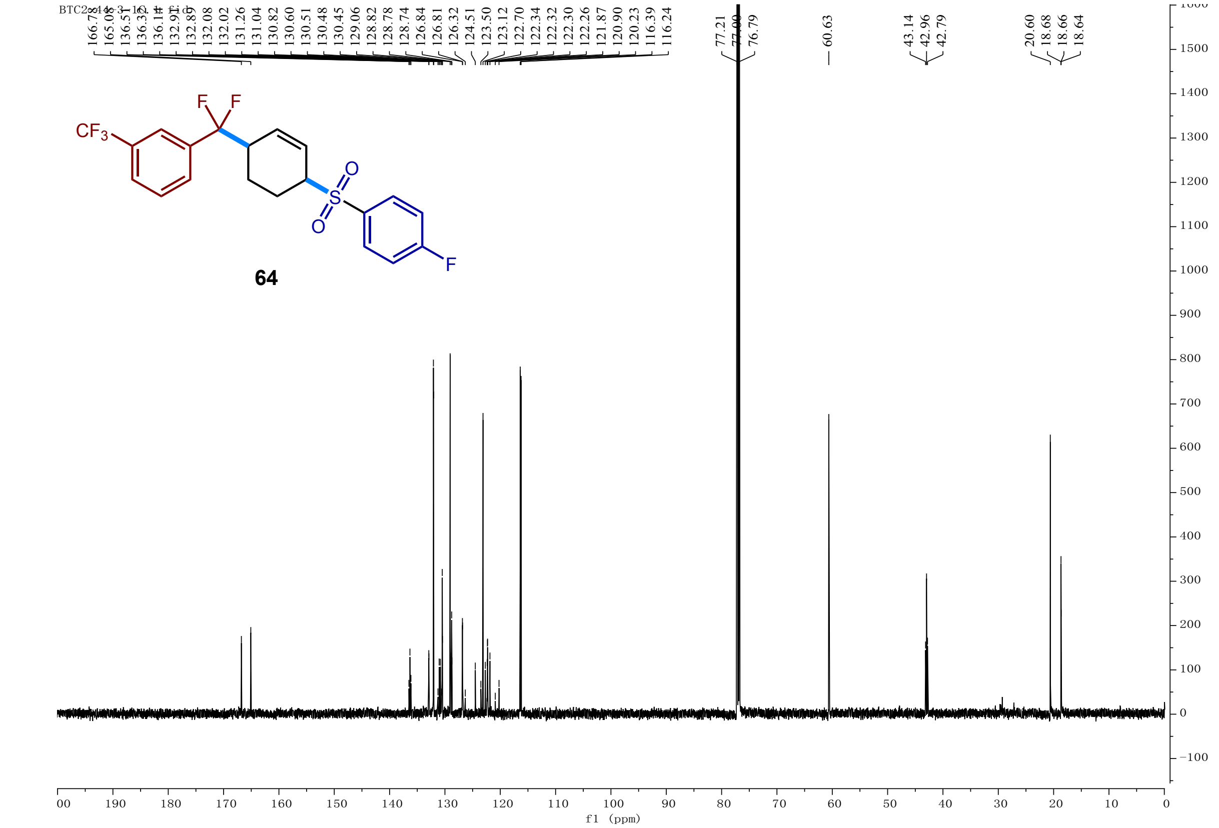
BTC2-144-3-13C
CF3
F F
O
S
O
F
64
f1 (ppm)

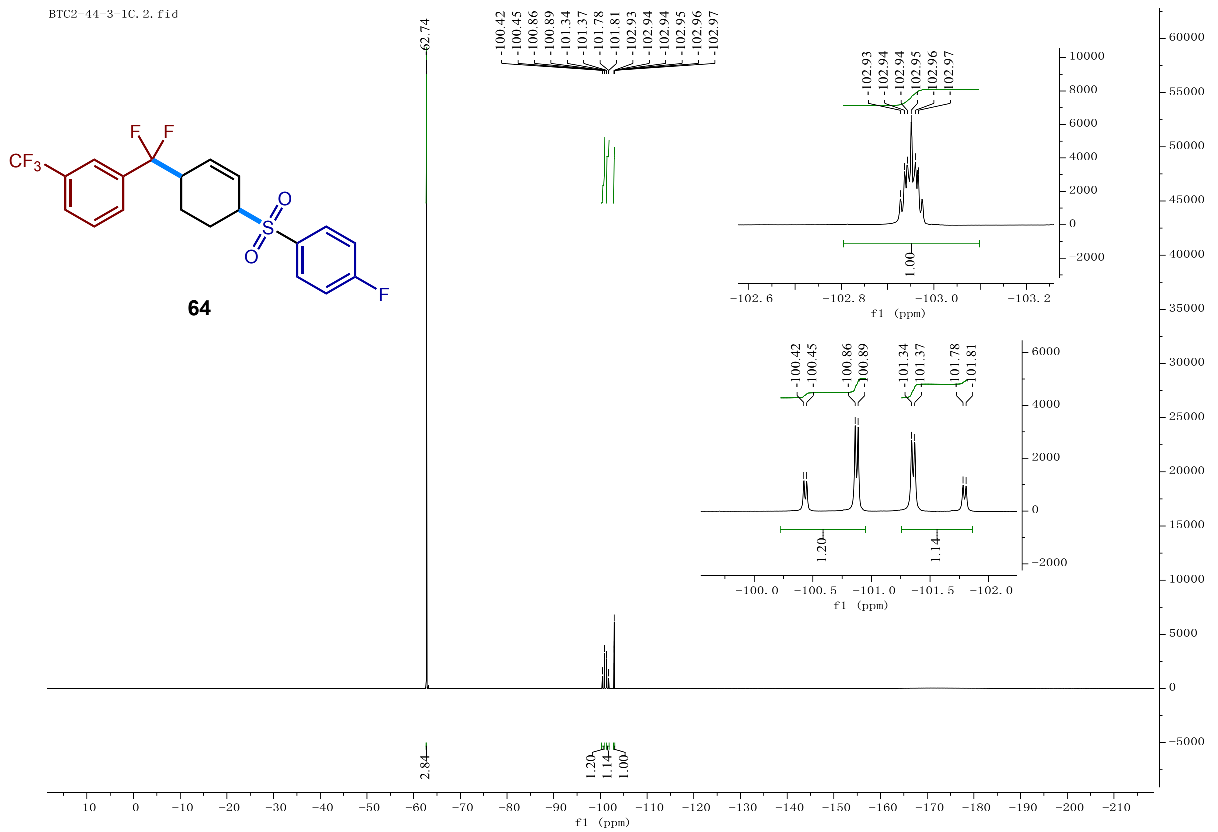
BTC2-44-3-1C.2.fid
64
-62.74
-100.42 -100.45 -100.86 -100.89 -101.34 -101.37 -101.78 -101.81 -102.93 -102.94 -102.94 -102.95 -102.96 -102.97
2.84
1.20
1.14
1.00
f1 (ppm)

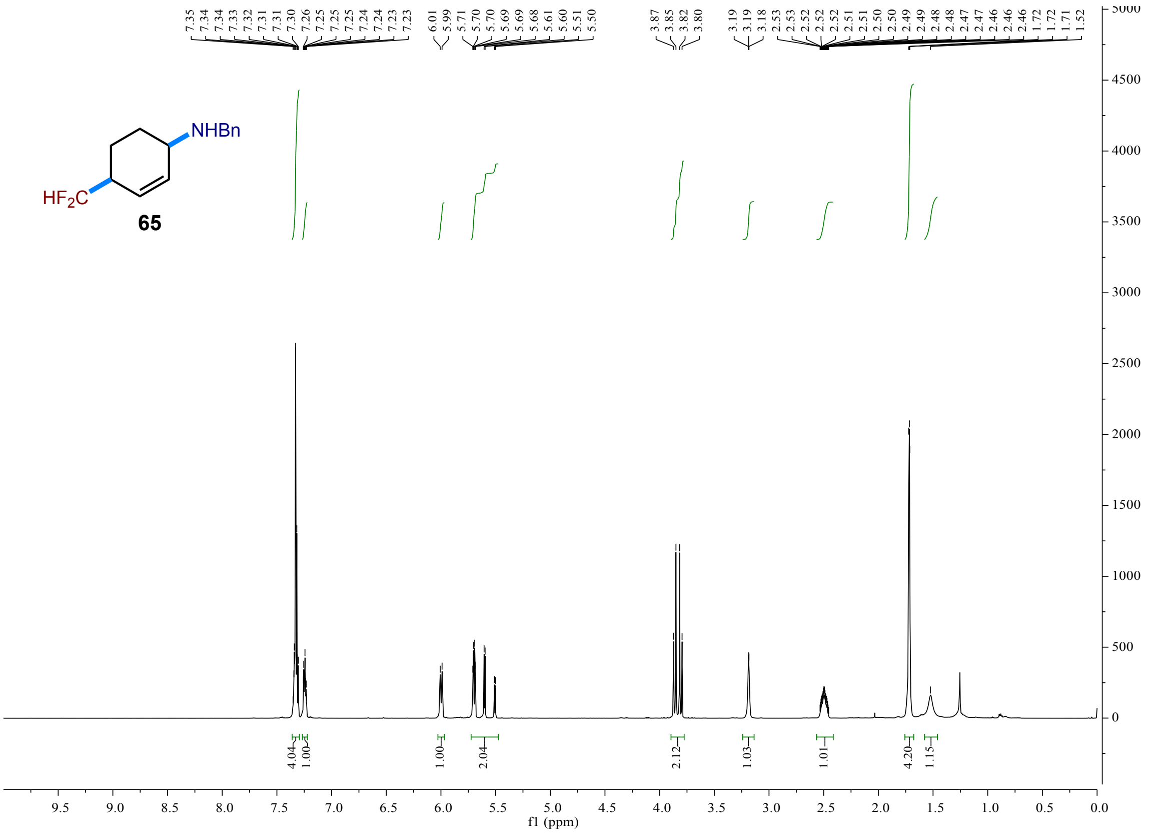
NHBn
HF_2C
65
7.35 7.34 7.34 7.33 7.32 7.31 7.31 7.30 7.26 7.25 7.25 7.25 7.24 7.24 7.23 7.23 6.01 5.99 5.71 5.70 5.70 5.69 5.69 5.68 5.61 5.60 5.51 5.50
3.87 3.85 3.82 3.80 3.19 3.19 3.18 2.53 2.53 2.52 2.52 2.52 2.51 2.51 2.50 2.50 2.49 2.49 2.48 2.48 2.47 2.46 2.46 2.46 1.72 1.72 1.71 1.52
4.04 1.00 1.00 2.04 2.12 1.03 1.01 4.20 1.15
f1 (ppm)

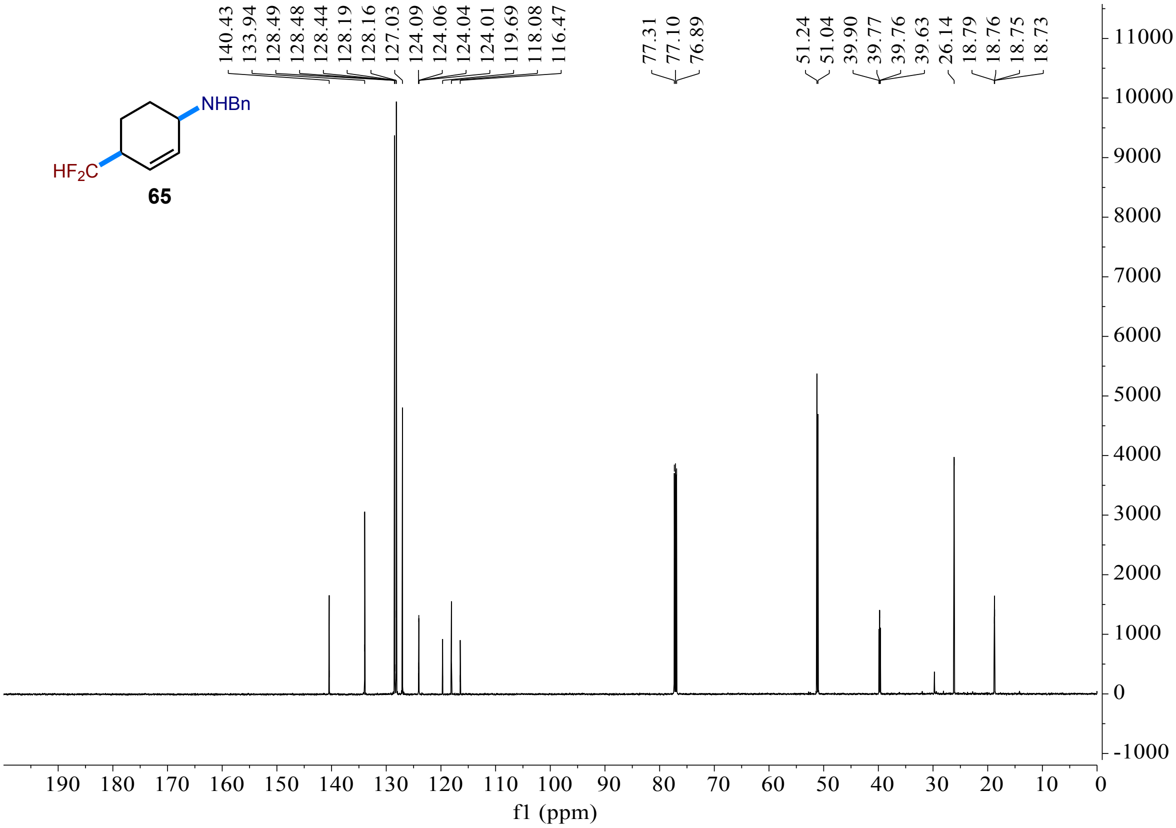
140.43
133.94
128.49
128.48
128.44
128.19
128.16
127.03
124.09
124.06
124.04
124.01
119.69
118.08
116.47
77.31
77.10
76.89
51.24
51.04
39.90
39.77
39.76
39.63
26.14
18.79
18.76
18.75
18.73
NHBn
HF2C
65
f1 (ppm)

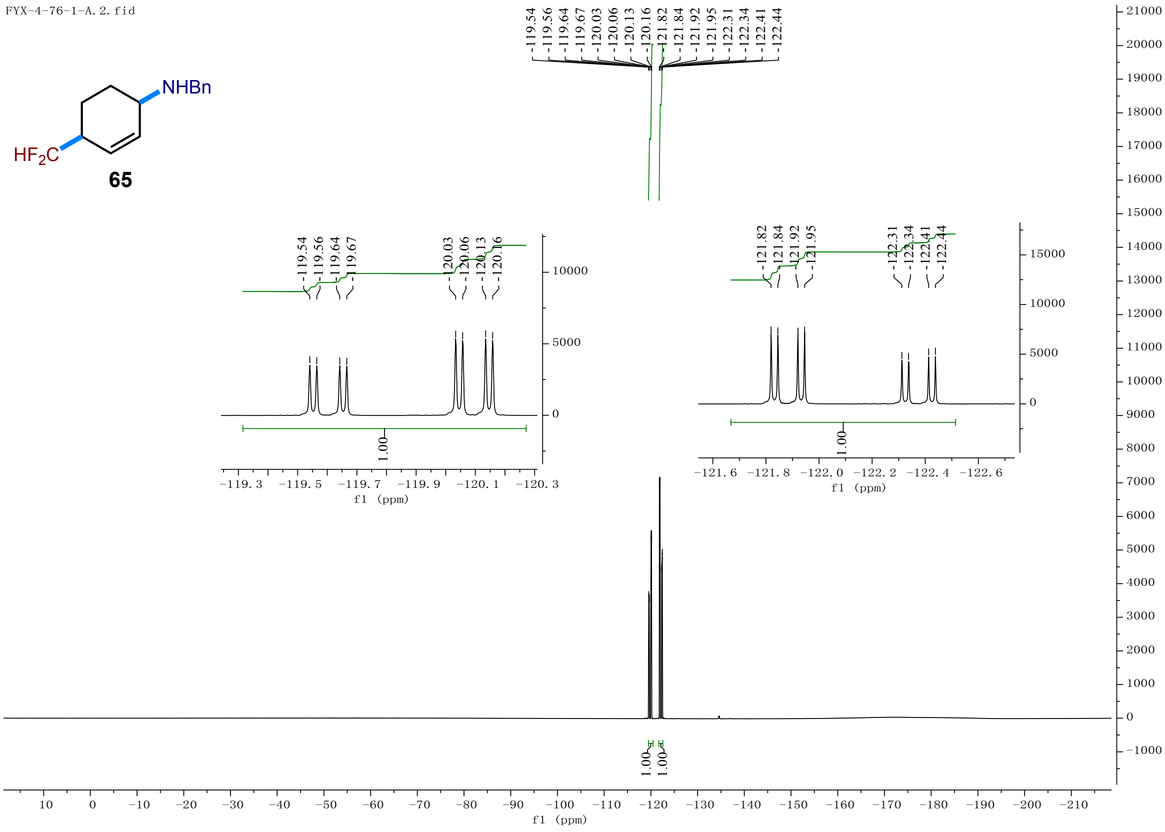
FYX-4-76-1-A.2.fid
-119.54
-119.56
-119.64
-119.67
-120.03
-120.06
-120.13
-120.16
-121.82
-121.84
-121.92
-121.95
-122.31
-122.34
-122.41
-122.44
NHBn
HF2C
65
1.00
1.00
f1 (ppm)

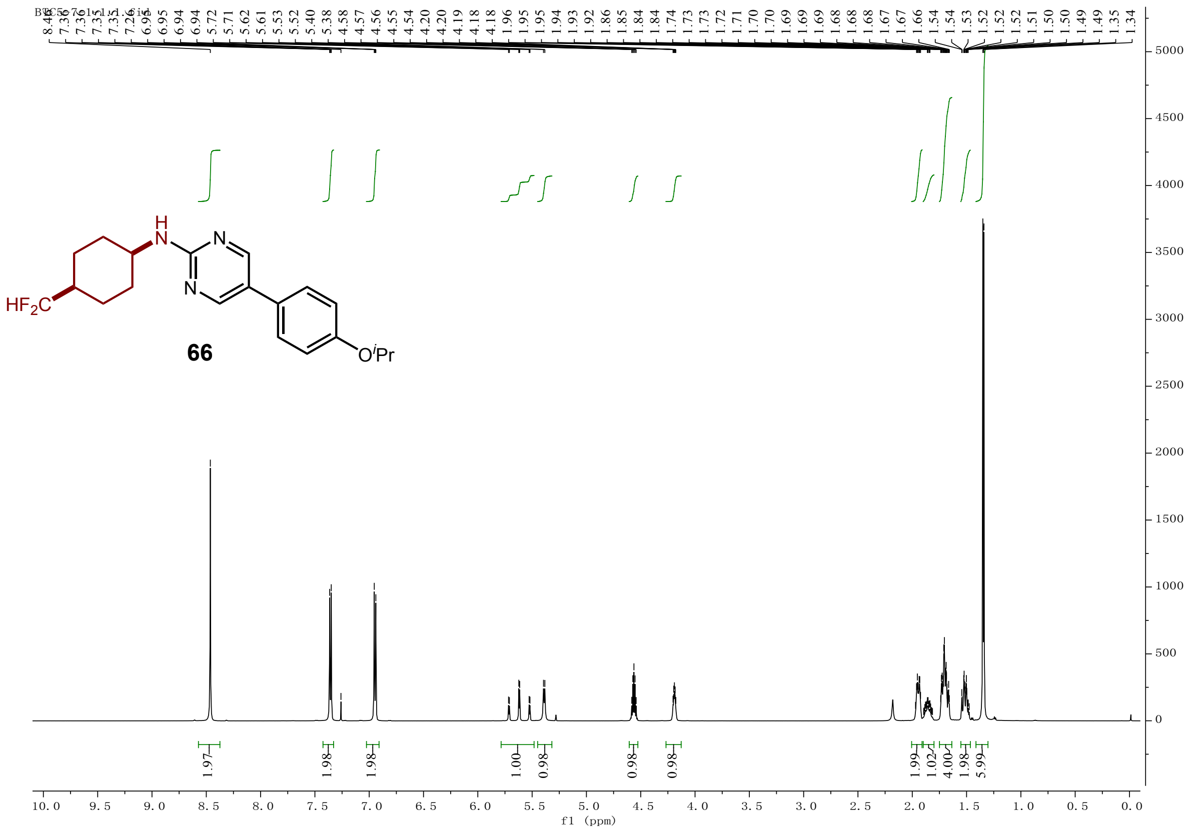
8.46
7.36
7.36
7.35
7.35
7.26
6.95
6.95
6.94
6.94
5.72
5.71
5.62
5.61
5.53
5.52
5.40
5.38
4.58
4.57
4.56
4.55
4.54
4.20
4.20
4.19
4.18
4.18
1.96
1.95
1.95
1.94
1.93
1.92
1.86
1.85
1.84
1.84
1.74
1.73
1.73
1.72
1.71
1.70
1.70
1.69
1.69
1.69
1.68
1.68
1.68
1.67
1.67
1.66
1.54
1.54
1.53
1.53
1.52
1.52
1.52
1.51
1.50
1.50
1.49
1.35
1.34
HF2C
H
N
N
N
O^{i}Pr
66
1.97
1.98
1.98
1.00
0.98
0.98
0.98
1.99
1.02
4.00
1.98
5.99
f1 (ppm)

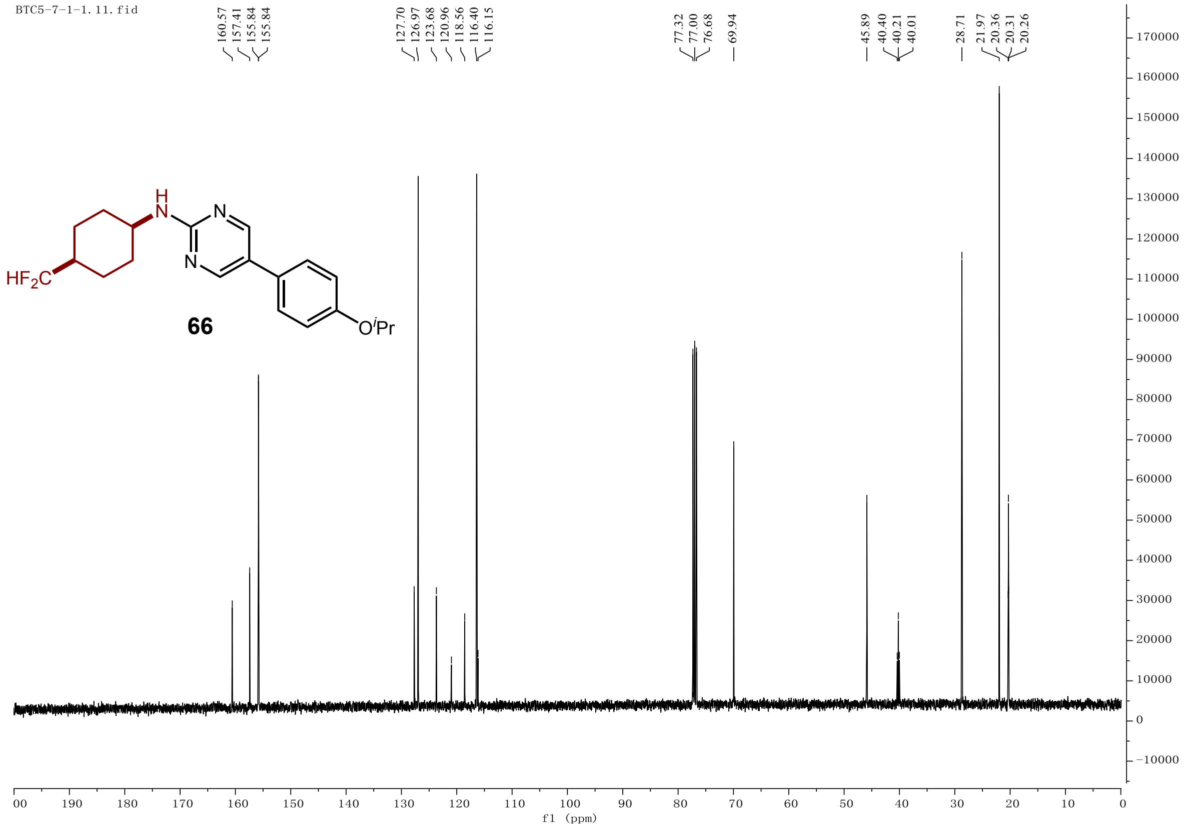
BTC5-7-1-1.11.fid
160.57
157.41
155.84
155.84
127.70
126.97
123.68
120.96
118.56
116.40
116.15
77.32
77.00
76.68
69.94
45.89
40.40
40.21
40.01
28.71
21.97
20.36
20.31
20.26
HF2C
H
N
N
N
O^{i}Pr
66
f1 (ppm)

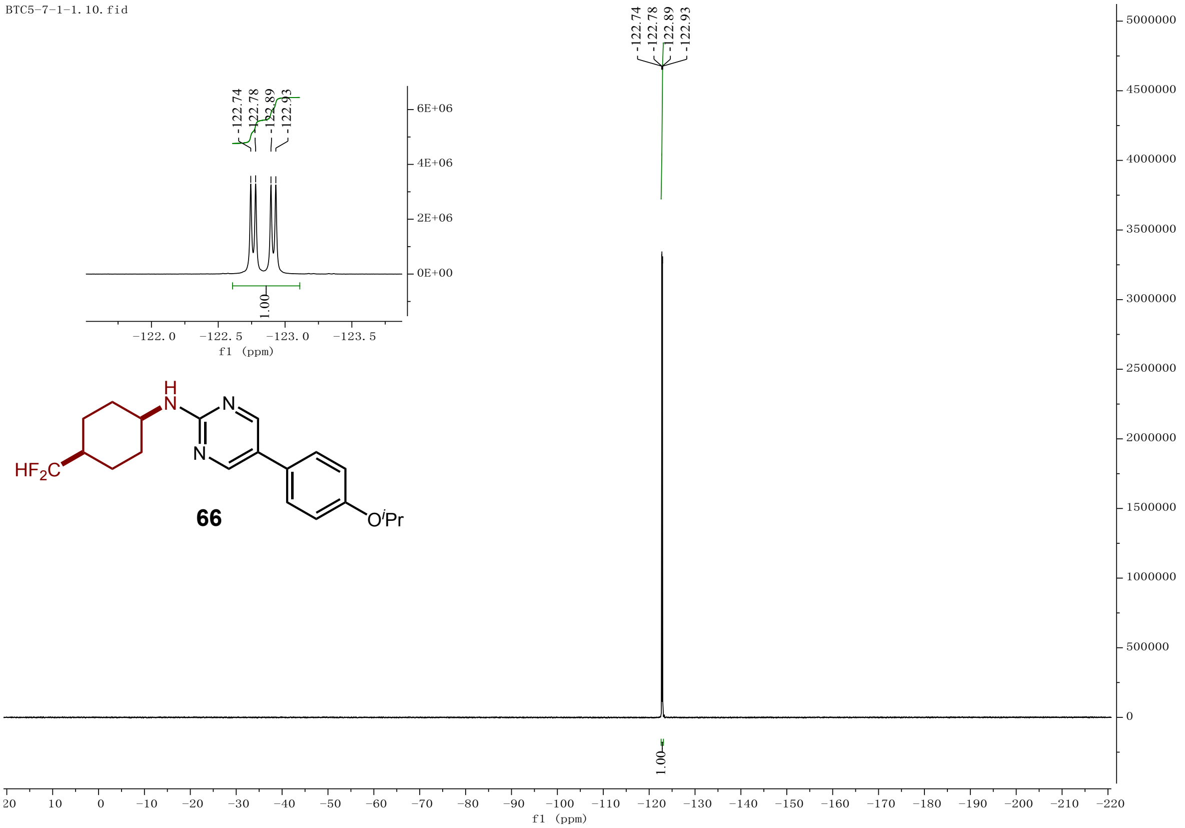
BTC5-7-1-1.10.fid
66
f1 (ppm)

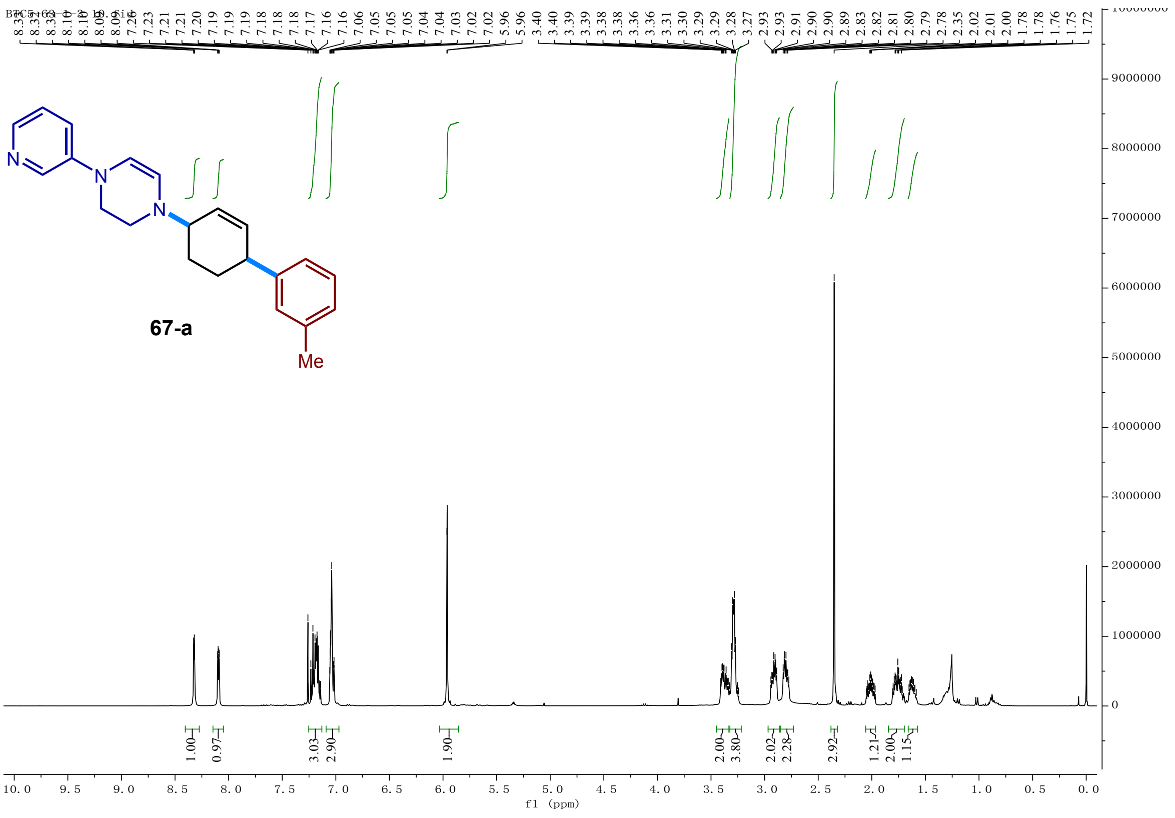
67-a
f1 (ppm)

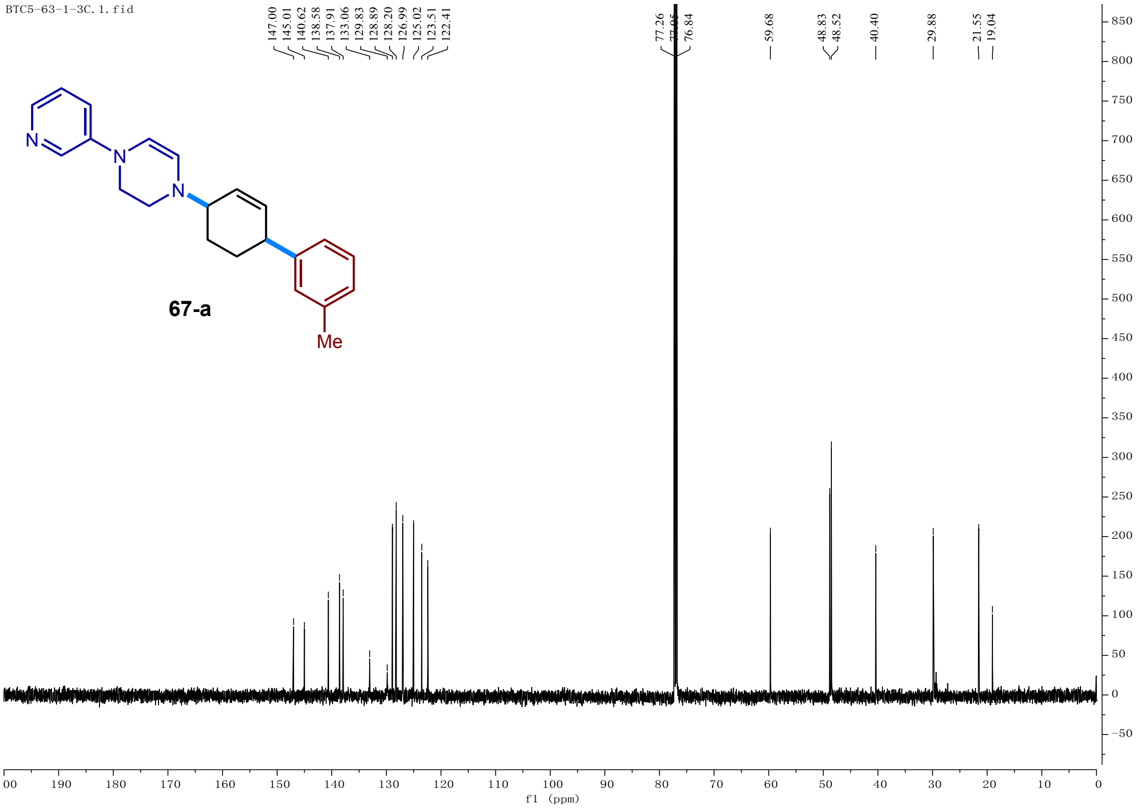
BTC5-63-1-3C.1.fid
147.00
145.01
140.62
138.58
137.91
133.06
129.83
128.89
128.20
126.99
125.02
123.51
122.41
77.26
77.05
76.84
59.68
48.83
48.52
40.40
29.88
21.55
19.04
N
N
N
Me
67-a
f1 (ppm)

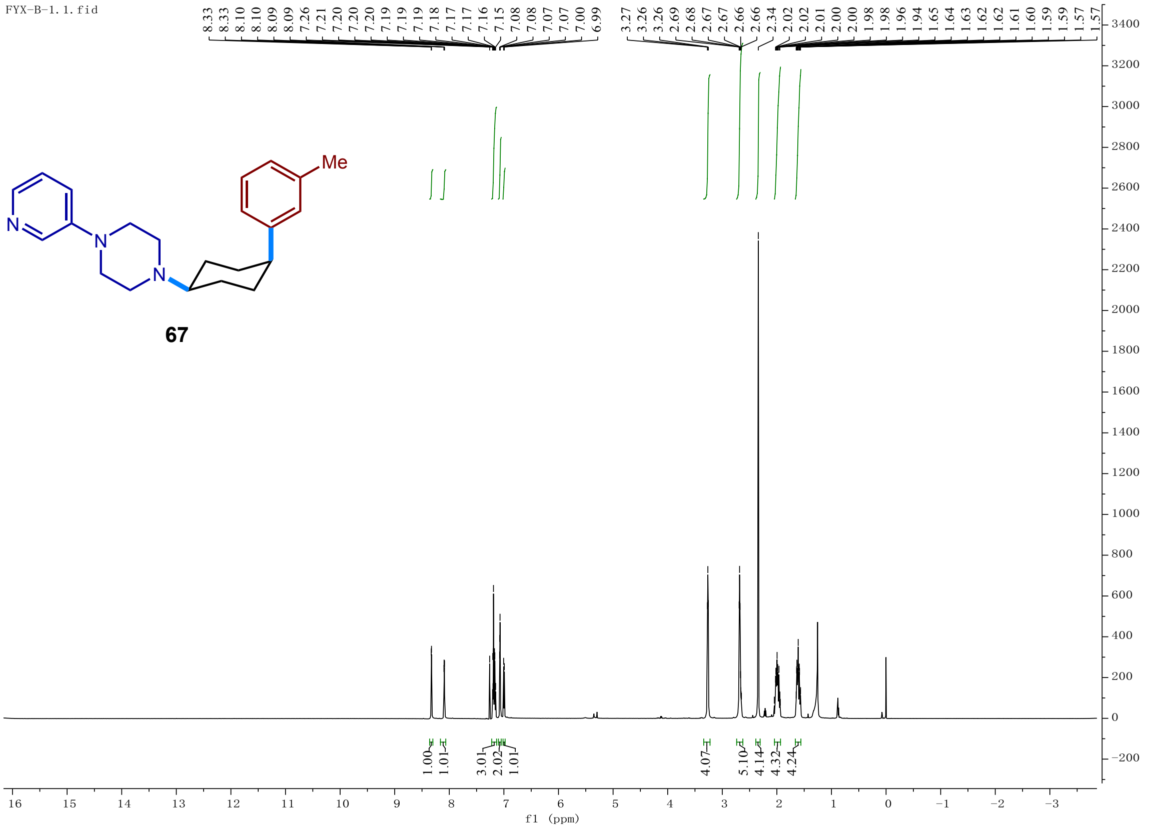
FYX-B-1.1.fid
8.33
8.33
8.10
8.10
8.09
7.26
7.21
7.20
7.20
7.20
7.19
7.19
7.19
7.18
7.17
7.17
7.16
7.15
7.08
7.08
7.07
7.07
7.00
6.99
3.27
3.26
3.26
2.69
2.68
2.67
2.67
2.66
2.66
2.34
2.02
2.02
2.01
2.00
2.00
1.98
1.96
1.94
1.65
1.64
1.63
1.62
1.62
1.61
1.60
1.59
1.57
1.57
Me
N
N
N
67
1.00
1.01
3.01
2.02
1.01
4.07
5.10
4.14
4.32
4.24
f1 (ppm)

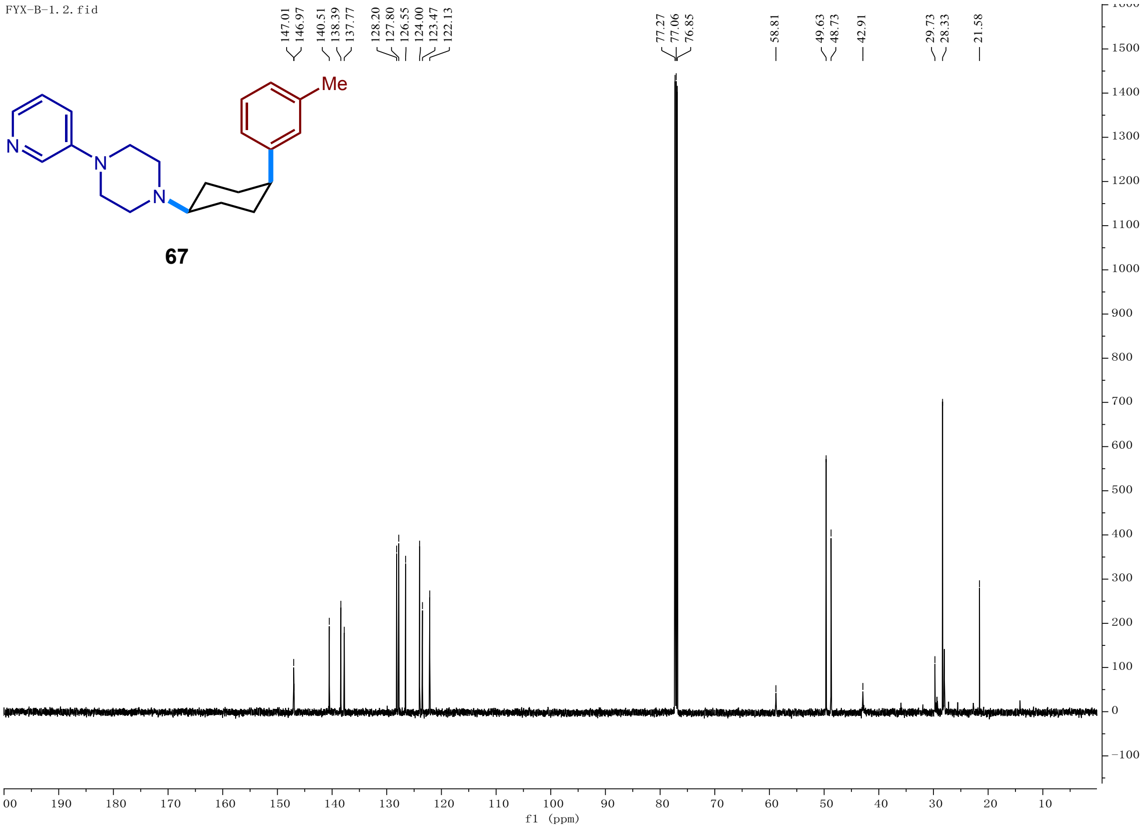
FYX-B-1.2.fid
147.01
146.97
140.51
138.39
137.77
128.20
127.80
126.55
124.00
123.47
122.13
77.27
77.06
76.85
58.81
49.63
48.73
42.91
29.73
28.33
21.58
Me
N
N
N
67
f1 (ppm)

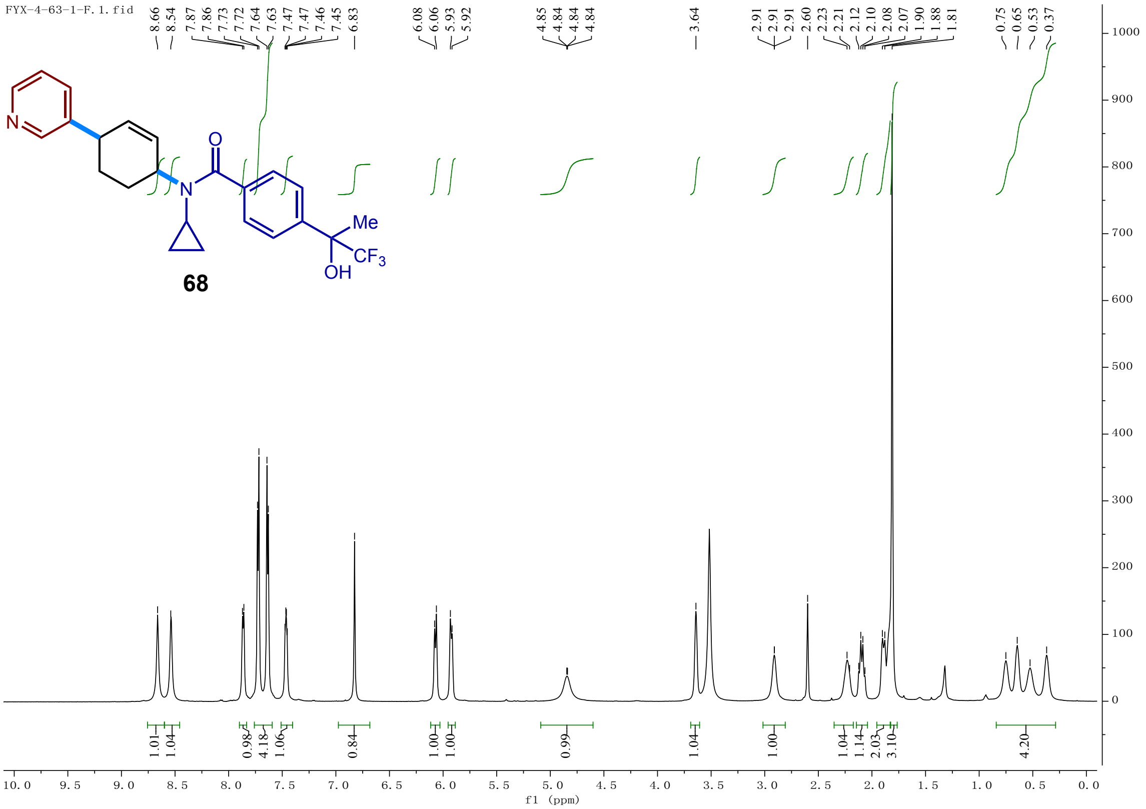
FYX-4-63-1-F.1.fid
8.66
8.54
7.87
7.86
7.73
7.72
7.64
7.63
7.47
7.46
7.45
6.83
6.08
6.06
5.93
5.92
4.85
4.84
4.84
4.84
3.64
2.91
2.91
2.91
2.60
2.23
2.21
2.12
2.10
2.08
2.07
1.90
1.88
1.81
0.75
0.65
0.53
0.37
N
O
N
Me
CF_3
OH
68
1.01
1.04
0.98
4.18
1.06
0.84
1.00
1.00
0.99
1.04
1.00
1.04
1.14
2.03
3.10
4.20
f1 (ppm)

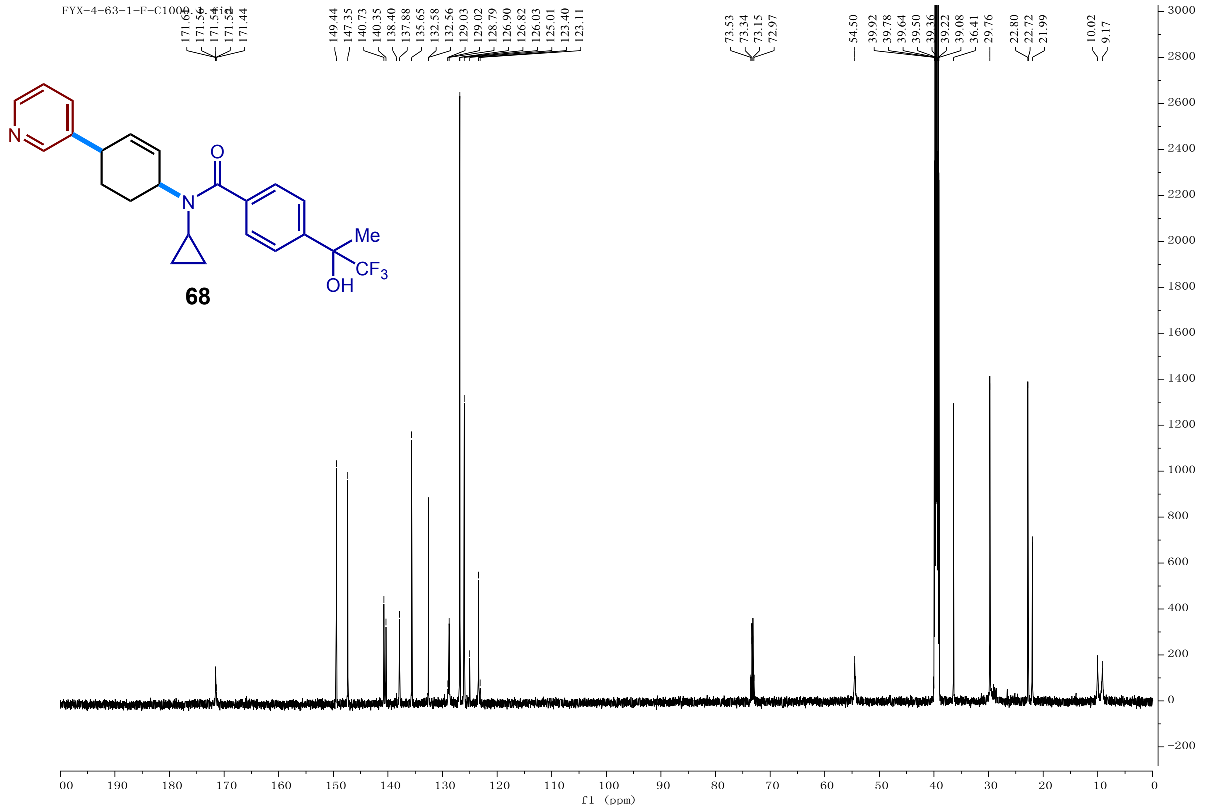
FYX-4-63-1-F-C1000.1.fid
68
Me
CF3
OH
N
O
171.62 171.56 171.54 171.52 171.44
149.44 147.35 140.73 140.35 138.40 137.88 135.65 132.58 132.56 129.03 129.02 128.79 126.90 126.82 126.03 125.01 123.40 123.11
73.53 73.34 73.15 72.97
54.50 39.92 39.78 39.64 39.50 39.36 39.22 39.08 36.41 29.76
22.80 22.72 21.99
10.02 9.17
f1 (ppm)

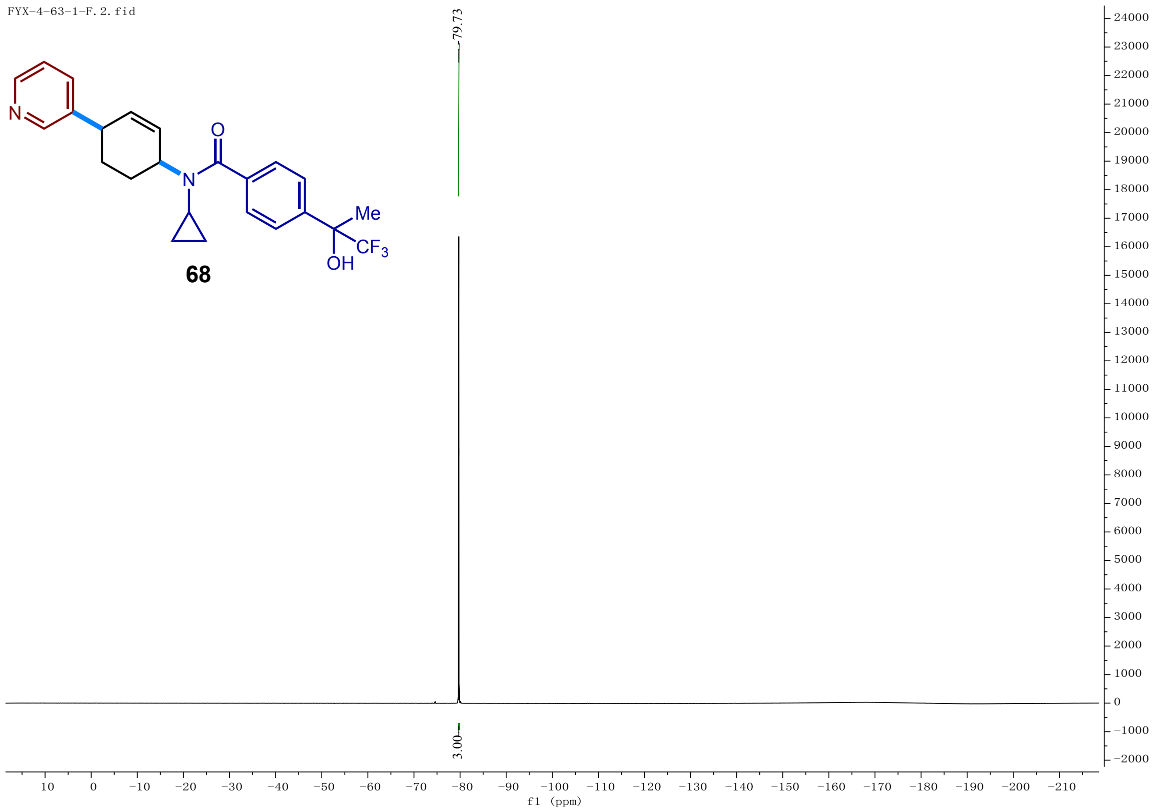
FYX-4-63-1-F.2.fid
68
Me
CF3
OH
N
O
-79.73
3.00
f1 (ppm)

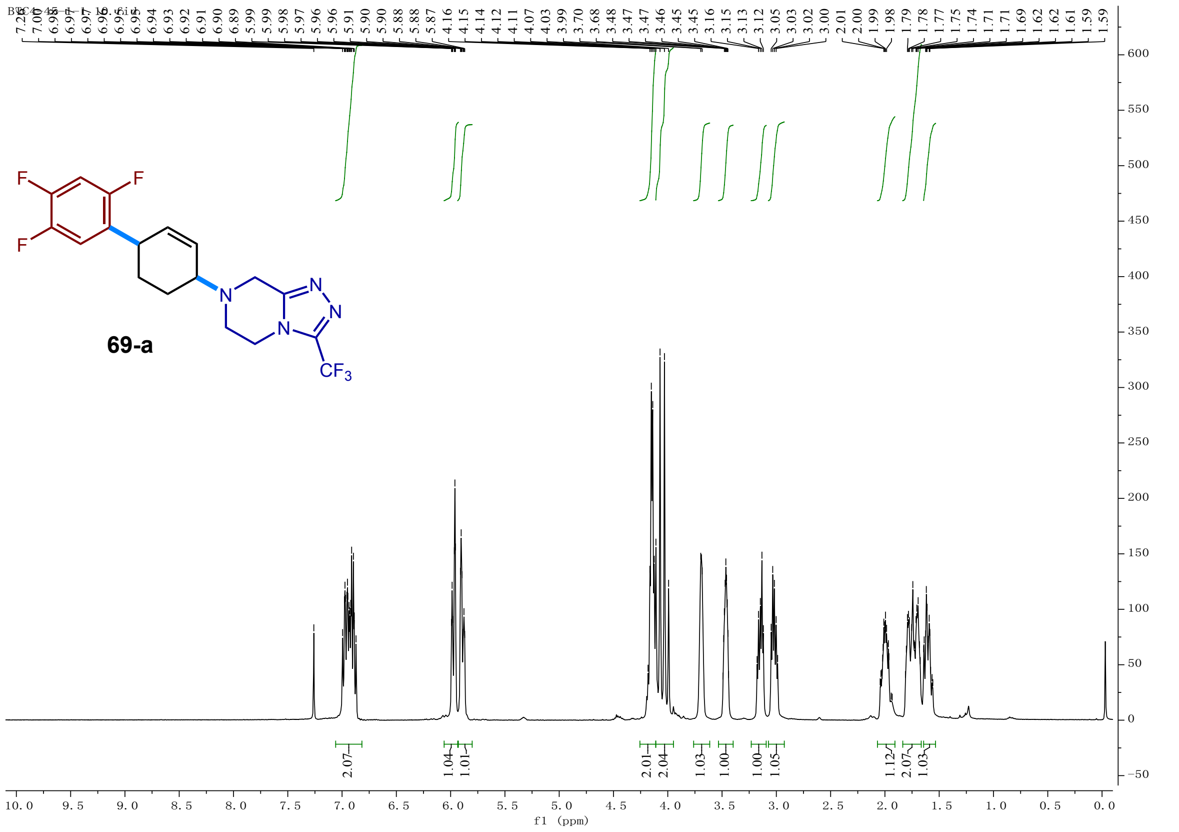

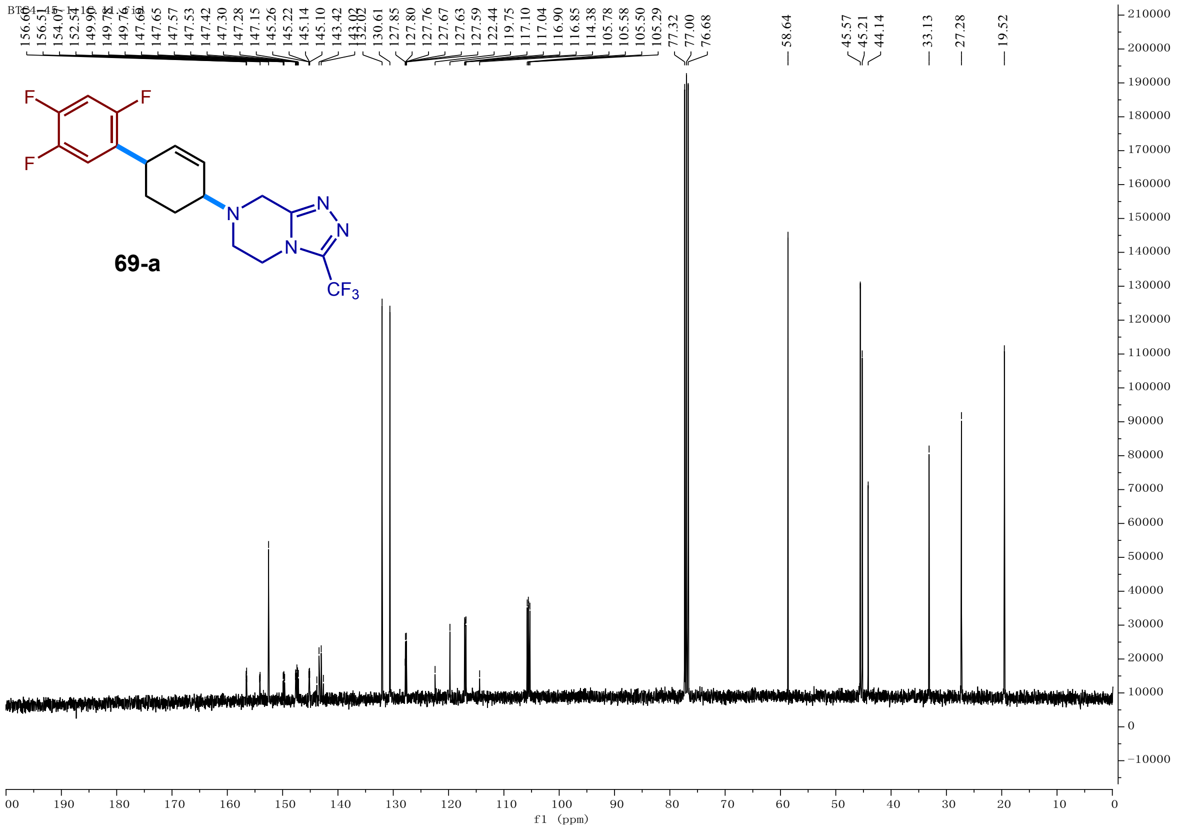

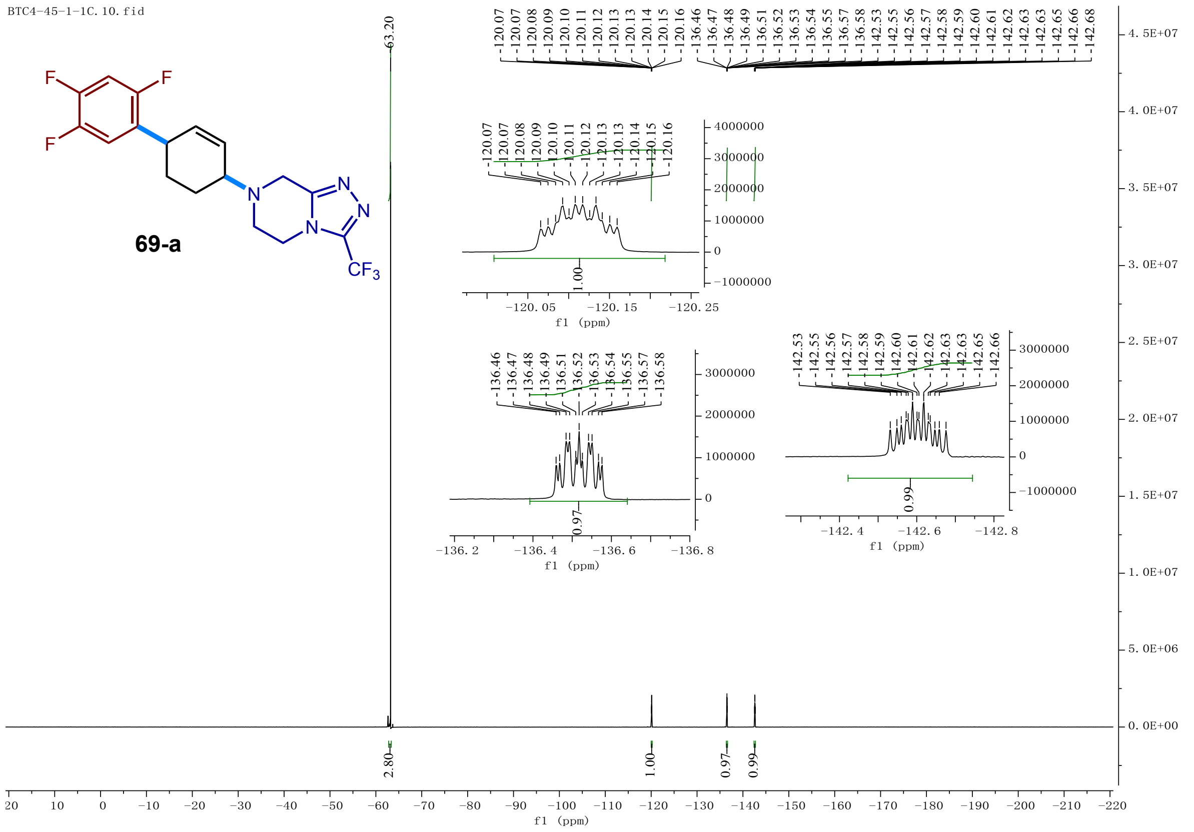
BTC4-45-1-1C.10.fid
69-a
-63.20
-120.07 -120.07 -120.08 -120.09 -120.10 -120.11 -120.12 -120.13 -120.13 -120.14 -120.15 -120.16
-136.46 -136.47 -136.48 -136.49 -136.51 -136.52 -136.53 -136.54 -136.55 -136.57 -136.58
-142.53 -142.55 -142.56 -142.57 -142.58 -142.59 -142.60 -142.61 -142.62 -142.63 -142.63 -142.65 -142.66 -142.68
2.80
1.00
0.97
0.99
f1 (ppm)

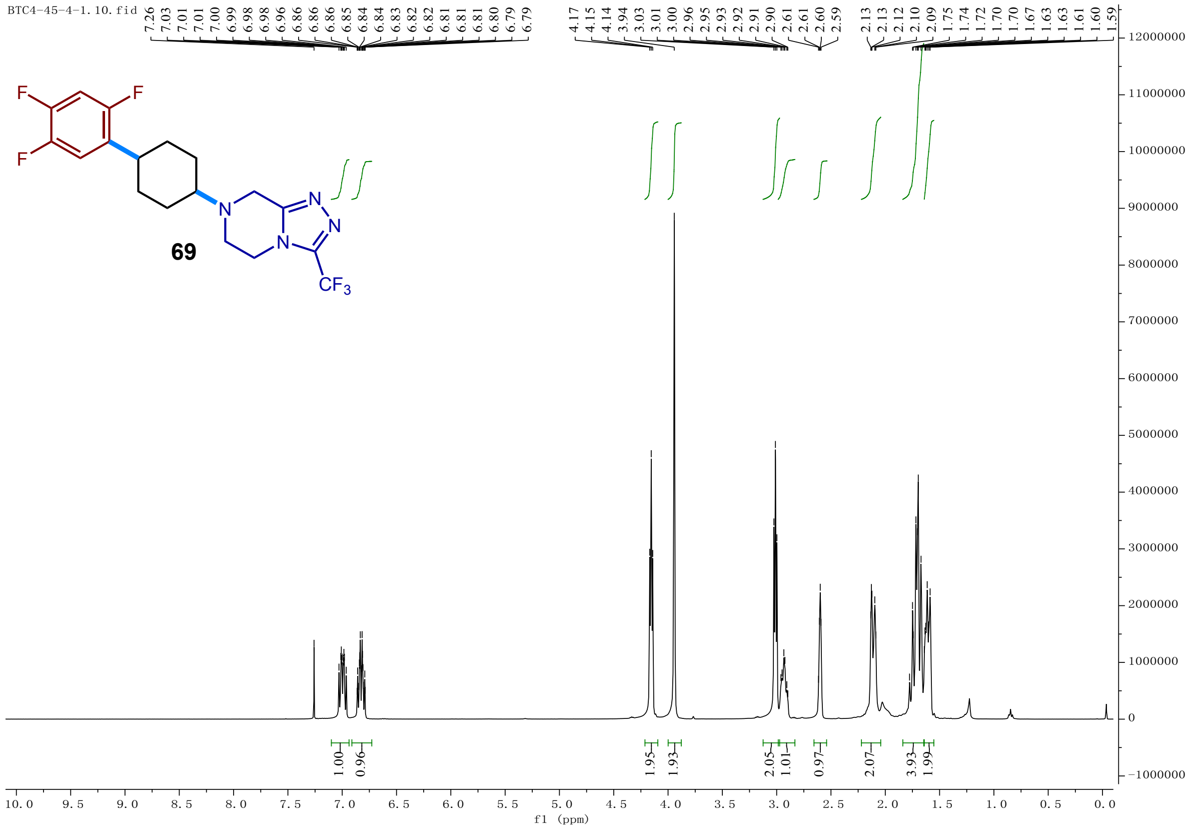
BTC4-45-4-1.10.fid
69
7.26 7.03 7.01 7.01 7.00 6.99 6.98 6.98 6.96 6.86 6.86 6.86 6.85 6.84 6.84 6.83 6.82 6.82 6.81 6.81 6.81 6.80 6.79 6.79
4.17 4.15 4.14 3.94 3.03 3.01 3.00 2.96 2.95 2.93 2.92 2.91 2.90 2.61 2.61 2.60 2.59
2.13 2.13 2.12 2.10 2.09 1.75 1.74 1.72 1.70 1.70 1.67 1.63 1.63 1.61 1.60 1.59
1.00 0.96 1.95 1.93 2.05 1.01 0.97 2.07 3.93 1.99
f1 (ppm)

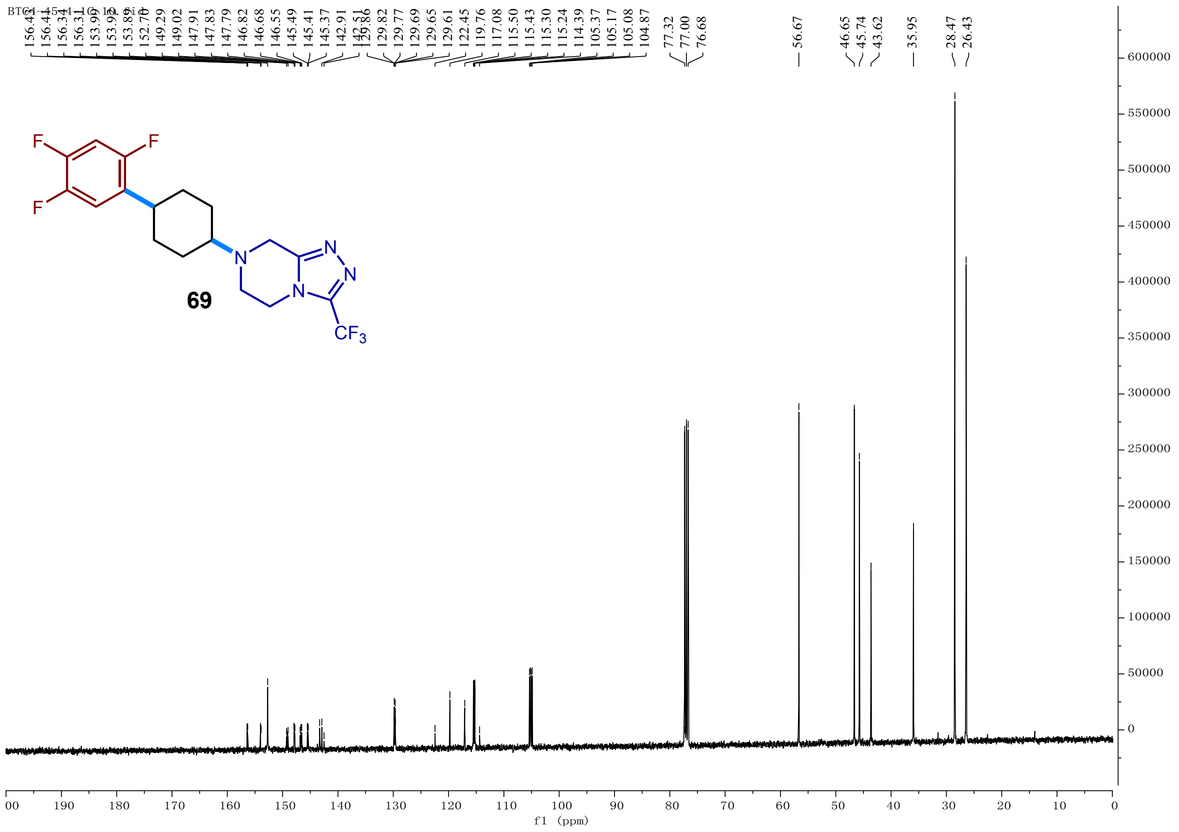

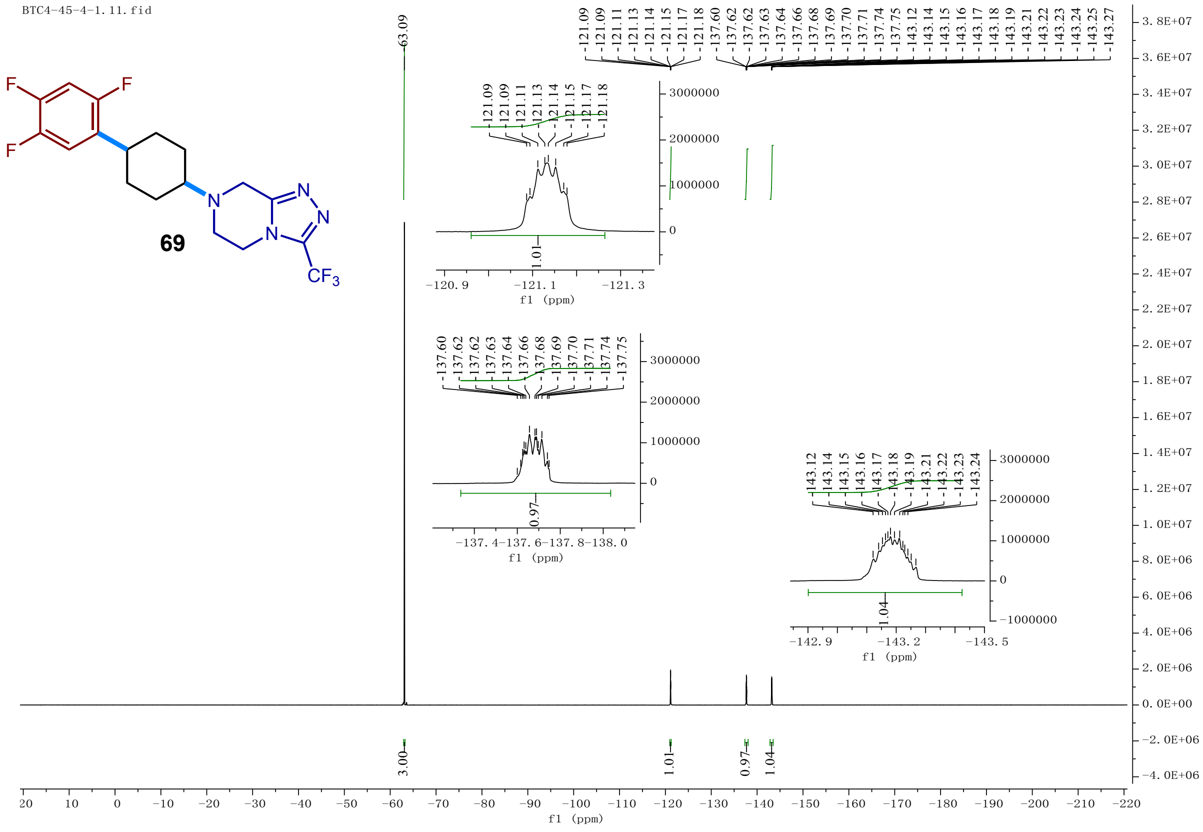

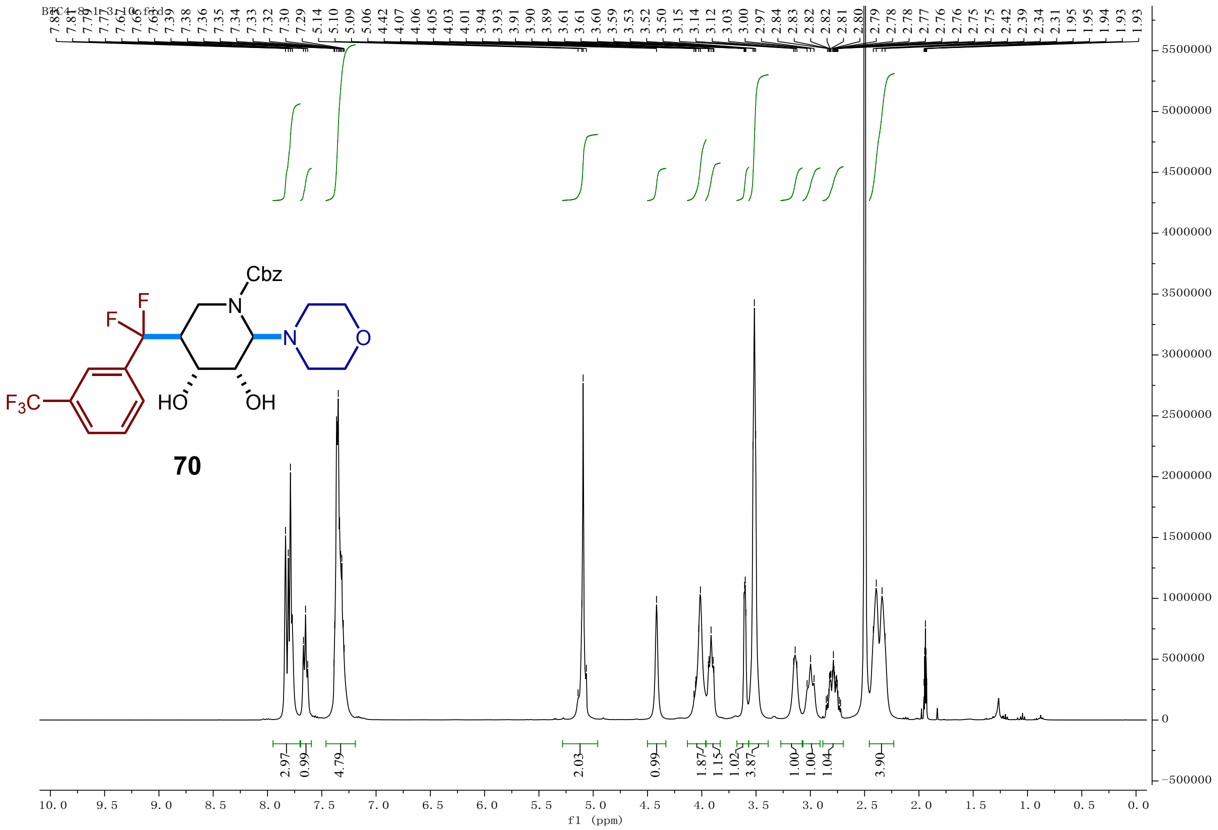
BTC4-8-1-3.10.fid
Cbz
F
F
N
N
O
F3C
HO
OH
f1 (ppm)

70

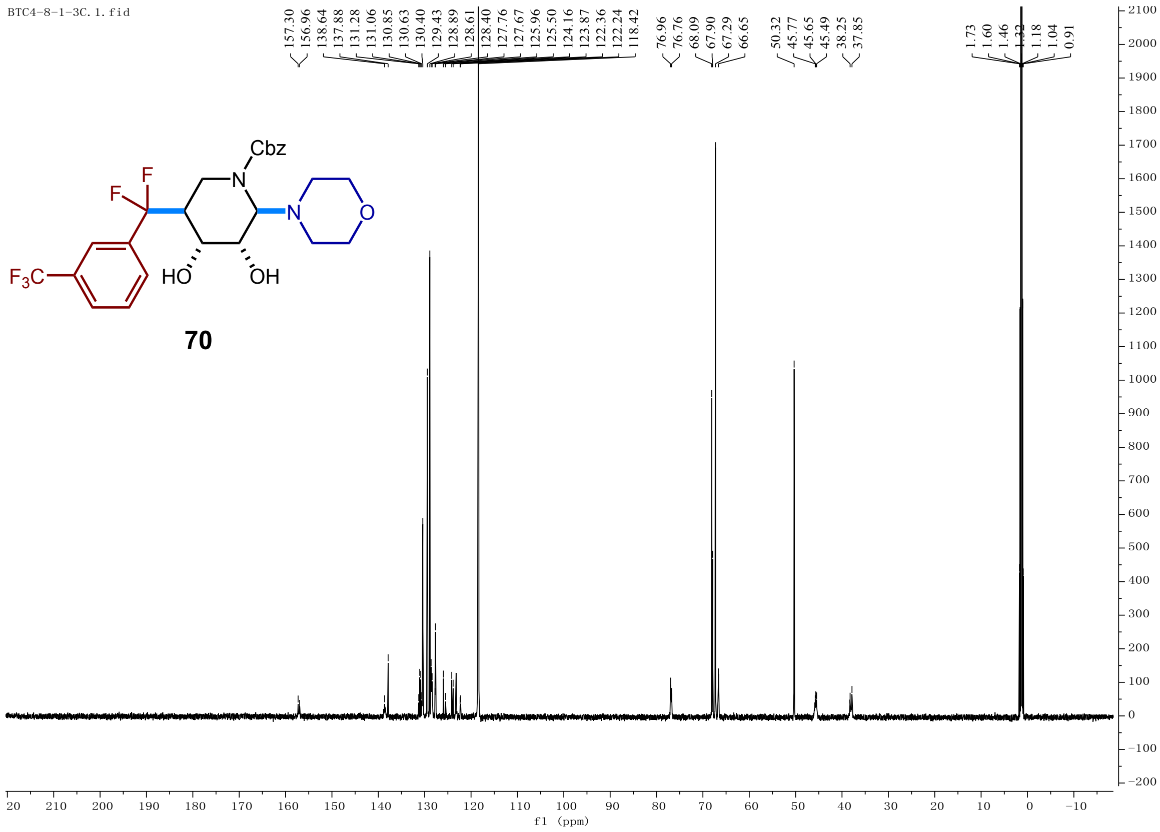
BTC4-8-1-3C.1.fid
Cbz
F
F
N
N
O
F3C
HO
OH
f1 (ppm)

70

r

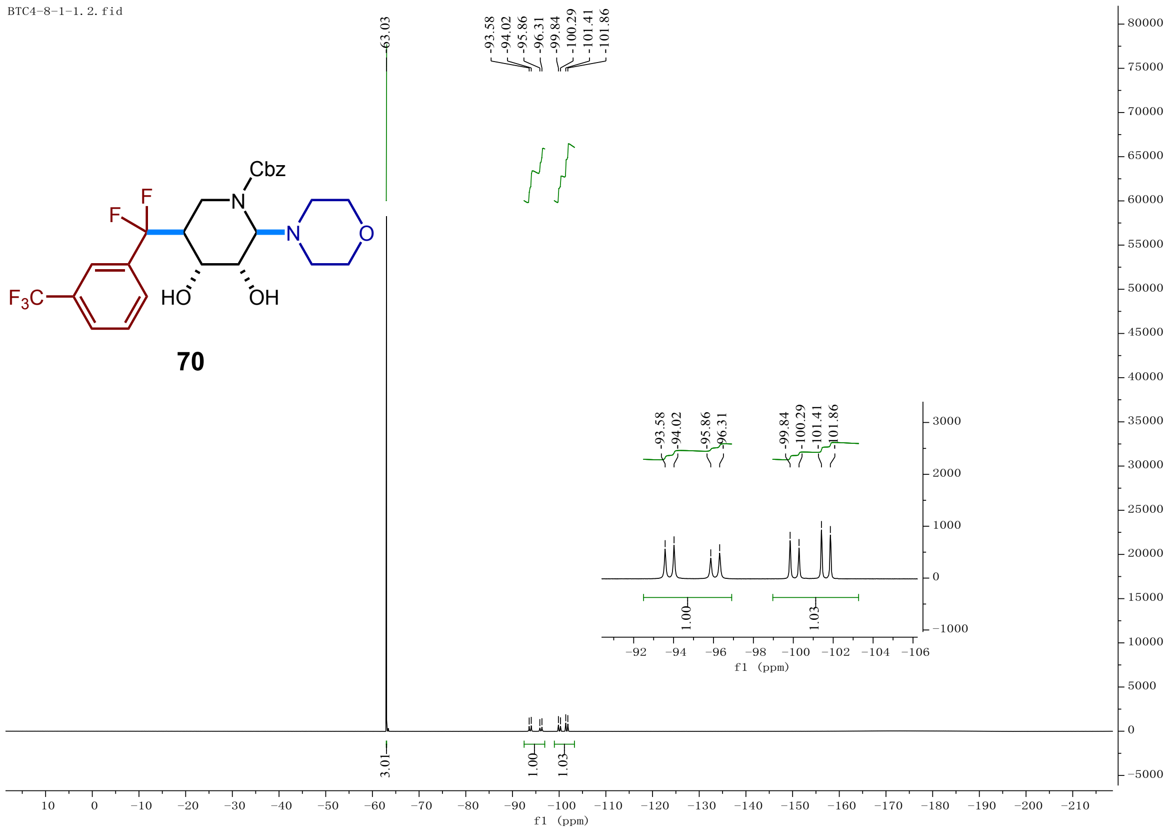

XII. References

1. Bayly, A. R., White, A. J. P. & Spivey, A. C. Design and Synthesis of a Prototype Scaffold for Five-Residue α-Helix Mimetics. Eur. J. Org. Chem. **2013**, 5566–5569 (2013).
2. Zhou, Y., Lu, Y., Hu, X., Mei, H., Lin, L., Liu, X. & Feng, X. Highly diastereo- and enantioselective synthesis of spirooxindole- cyclohexaneamides through N,N′-dioxide/Ni(II)-catalyzed Diels–Alder reactions. Chem. Commun. **53**, 2060–2063 (2017).
3. Mishra, S., Karabiyikoglu, S. & Fletcher, S. P. Catalytic Enantioselective Synthesis of 3-Piperidines from Arylboronic Acids and Pyridine. J. Am. Chem. Soc. **145**, 14221–14226 (2023).
4. Yu, B., Zou, S., Liu, H. & Huang, H. Palladium-catalyzed ring-closing reaction via C-N bond metathesis for rapid construction of saturated N-heterocycles. J. Am. Chem. Soc. **142**, 18341–18345 (2020).

5. Zhang, X., Ling, L., Luo, M. & Zeng, X. Accessing Difluoromethylated and Trifluoromethylated cis-Cycloalkanes and Saturated Heterocycles: Preferential Hydrogen Addition to the Substitution Sites for Dearomatization. Angew. Chem. Int. Ed. **58**, 16785–16789 (2019).
6. Sun, R., Granucci, G., Paul, A. K., Siebert, M., Liang, H. J., Cheong, G., Hase, W. L. & Persico, M. J. Chem. Phys., 2015, **142**(10)**,** 104302.
7. Shoji, A., Schanzenbach, D., Merrill, R., Zhang, J., Yang, L. & Sun, R. J. Phys. Chem. A, 2019, **123**(45), 9791-9799.
8. Luo, Y., Kreuscher, T., Kang, C., Hase, W. L., Weitzel, K. M. & Sun, R. Int. J. Mass Spectrom., 2021, **462**, 116515.
9. Fujioka, K., Weitzel, K. M. & Sun, R. J. Phys. Chem. A, 2022, **126**(9), 1465-1474.
10. Chen, Y. Q., Singh, S., Wu, Y., Wang, Z., Hao, W., Verma, P., Qiao, J. X., Sunoj, R. B. & Yu, J. Q. J. Am. Chem. Soc., 2020, **142**(22), 9966-9974.
11. Bag, S., Mondal, S. K, A., Jayarajan, R., Dutta, U., Porey, S., Sunoj, R. B. & Maiti, D., J. Am. Chem. Soc. 2020, **142**(28), 12453-12466.
12. Lázaro-Milla, C., Quirós, M. T., Cárdenas, D. J. & P. Almendros. Chem. Comm., 2020, **56**(45), 6070-6073.
13. Akporji, N., Thakore, R. R., Cortes-Clerget, M., Andersen, J., Landstrom, E., Aue, D. H., Gallou, F. & Lipshutz, B. H. Chem. Sci., 2020, **11**(20), 5205-5212.
14. Hu, L., Gao, H., Hu, Y., Lv, X., Wu, Y. B.. & Lu, G. J. Org. Chem., 2021, **86**(24), 18128-18138.
15. Ano, Y., Kawai, N. & Chatani, N. Chem. Sci., 2021, **12**(37), 12326-12332.
16. Li, Y., Zhang, P., Liu, Y. J., Yu, Z. X. & Shi, B. F. ACS Catalysis, 2020, **10**(15), 8212-8222.
17. Pati, B. V., Ghosh, A., Yadav, K., Banjare, S. K., Pandey, S., Lourderaj, U. & Ravikumar, P. C. Chem. Sci., 2022, **13**(9), 2692-2700.
18. Goyal, K., Kukier, G. A., Chen, X., Turlik, A., Houk, K. N. & Sarpong, R. Chem. Sci., 2023, **14**(42), 11809-11817.
19. Lee, C., Yang, W. & Parr, R. G. Phys. Rev. B: Condens. Matter Mater. Phys., 1988, **37**, 785–789.
20. Becke, A. D., J. Chem. Phys., 1993, **98**, 1372–1377.

21. Grimme, S., Antony, J., Ehrlich, S.. & Krieg, H., J. Chem. Phys., 2010, **132**, 154104.
22. Clark, T., Chandrasekhar, J., Spitznagel, G. W. & Schleyer, P. V. R., J. Comput. Chem., 1983, **4**, 294–301.
23. Fuentealba, P., Stoll, H., von Szentpaly, L., Schwerdtfeger, P., & Preuss, H., J. Phys. B: At. Mol. Opt. Phys. 1983, **16**, L323.
24. Tomasi, J., Mennucci, B., & Cammi, R., Chem. Rev. 2005, **105**, 2999–3094.
25. Grimme, S. J. Chem. Phys., 2006, **124**(3).
26. Frisch, M. J., Trucks, G. W., Schlegel, H. B., Scuseria, G. E., Robb, M. A., Cheeseman, J. R., Scalmani, G., Barone, V., Petersson, G. A., Nakatsuji, H., Li, X., Caricato, M., Marenich, A. V., Bloino, J., Janesko, B. G., Gomperts, R., Mennucci, B., Hratchian, H. P., Ortiz, J. V., Izmaylov, A. F., Sonnenberg, J. L., Williams-Young, D., Ding, F., Lipparini, F., Egidi, F., Goings, J., Peng, B., Petrone, A., Henderson, T., Ranasinghe, D., Zakrzewski, V. G., Gao, J., Rega, N., Zheng, G., Liang, W., Hada, M., Ehara, M., Toyota, K., Fukuda, R., Hasegawa, J., Ishida, M., Nakajima, T., Honda, Y., Kitao, O., Nakai, H., Vreven, T., Throssell, K., Montgomery, J. A., Peralta, J. E., Ogliaro, F., Bearpark, M. J., Heyd, J. J., Brothers, E. N., Kudin, K. N., Staroverov, V. N., Keith, T. A., Kobayashi, R., Normand, J., Raghavachari, K., Rendell, A. P., Burant, J. C., Iyengar, S. S., Tomasi, J., Cossi, M., Millam, J. M., Klene, M., Adamo, C., Cammi, R., J. W., Martin, R. L., Morokuma, K., Farkas, O., Foresman, J. B. & Fox, D. J., Gaussian, Inc., Wallingford CT, **2016**.
27. (a) Bäckvall, J. E.; Juntunen, S. K. 2-(Phenylsulfonyl)-1,3-dienes as versatile synthons in organic transformations. Multicoupling reagents and Diels-Alder dienes with a dual electron demand. *J. Am. Chem. Soc.* **1987**, *109*, 6396-6403. (b) Patel, B. A.; Kao, L. C.; Cortese, N. A.; Minkiewicz, J. V.; Heck, R. F. Palladium-Catalyzed Vinylation of Conjugated Dienes. *J. Org. Chem.* **1979**, *44*, 918-921. (c) Yu, B.; Zou, S.; Liu, H.; Huang, H. Palladium-catalyzed ring-closing reaction via C-N bond metathesis for rapid construction of saturated N-heterocycles. *J. Am. Chem. Soc*. **2020**, *142*, 18341–18345.
28. Fujioka, K., Kaiser, R. I., & Sun, R., J. Phys. Chem. A, 2022, **127**(4), 913-923.
29. Bhandari Neupane, J., Neupane, R. P., Luo, Y., Yoshida, W. Y., Sun, R., & Williams, P. G., Org. Lett., 2019, **21**(20), 8449-8453.

30. Eyring, H., J. Chem. Phys., 1935, **3**(2), 107–115.

31. Eyring, H. & Polanyi, M., Z. Phys. Chem., Abt. B, 1931 **12**, 279–311.

32. Carey, F. A.; Sundberg, R. J.; (1984). Advanced Organic Chemistry Part A Structure and Mechanisms (2nd ed.). New York N.Y.: Plenum Press. ISBN 0-306-41198-9
