# Supplementary material for: Selective 1,4-syn-Addition to Cyclic 1,3-Dienes via Hybrid Palladium Catalysis
Source: ACS Cent Sci. 2024 May 15;10(6):1191–200. doi: 10.1021/acscentsci.4c00094 (PMC11212138; doi:10.1021/acscentsci.4c00094)
Supplement: Supplementary file 2 — oc4c00094_si_002.pdf [file oc4c00094_si_002.pdf]

oc-2024-00094s.R1

Name: Peer Review Information for "Selective 1,4-syn-addition to Cyclic 1,3-Dienes via Hybrid Palladium Catalysis"

First Round of Reviewer Comments

Reviewer: 1

Comments to the Author

- This manuscript by Zhang et al presents a remarkably general protocol for the conversion of cyclic 1,3-dienes into cis-1,4-disubstituted cycloalkenes. The scope demonstrated is extraordinarily broad, with the choice of electrophile including both aryl and alkyl halides, and the choice of nucleophile including multiple examples of carbon and nitrogen, as well as a few examples of S and O nucleophiles. The authors also demonstrate their protocol in the preparation of several biologically interesting compounds. Judging from the SI, the broad claim of >20:1 dr for all reactions seems valid. I congratulate the authors on this beautiful chemistry and enthusiastically recommend publication in ACS Central Science after modification. I feel that the introduction needs to be rewritten. The length is appropriate, but it needs to do a better job of describing the most relevant science that has led up to this study. From what I can tell, this study is truly unique in the broad range of nucleophiles and electrophiles that successfully participate. The authors even describe in the SI examples that did not work, which I appreciated. But the references are almost impossible to sort out. There are far too many references that are not really relevant to the current work (and they are bunched up under a single reference number: reference 14 and reference 18 each have close to 20 references within them!) In some cases one or two reviews are sufficient. There are numerous papers listed dealing with chemistry of dienes, and numerous papers dealing with photocatalysis. But what are the closest examples to what the authors are demonstrating? With a bit of streamlining the introduction and a shirt discussion section at the end, this paper has the potential to be a seminal article that many will be guided by in the future. Perhaps the authors might include these two references for other approaches to cis-1,4-disubstituted cyclohexenes: Weatherford-Pratt, J.T., Smith, J.A., Bloch, J.M. et al. The double protonation of dihapto-coordinated benzene complexes enables dearomatization using aromatic nucleophiles. Nat Commun 14, 3145 (2023). Justin T. Weatherford-Pratt et al.,Tungsten-anisole complex provides 3,6-substituted cyclohexenes for highly diversified chemical libraries. Sci. Adv.10, eadl0885(2024). The DFT and the Eyring kinetics is a nice addition, but aside from a summarizing sentence or two, probably belongs in the SI. There is nothing unusual going on here.

Reviewer: 2

Comments to the Author

The manuscript by Zhang describes an excited Pd-catalyzed three-component reaction of cyclic 1,3-dienes, alkyl/aryl halides, and amines. The reaction forms 1,4-cis-substituted cyclic framework. Other nucleophiles in addition to amines, including C- and O-nucleophiles, were also compatible. A hybrid palladium-catalyzed radical-polar crossover mechanism was proposed for the reaction. A wide array of halides, including trifluoromethyl arenes, aryl bromides, and alkyl iodides, underwent the three-component reaction.

The reaction provides a modular approach for the synthesis of 1,4-cis-substituted cyclic compounds. I recommend this manuscript to be accepted for publication in ACS central science after some issues as below are addressed.

1, For the trifluoromethylarene scope, the substrates were limited to benzenes bearing two trifluoromethyl groups and trifluoromethylpyridine derivatives. How about trifluoromethylbenzene and its simple, and other heteroarenes.

2, For products 5-10, the evidences should be provided to prove the regioselectivity.

3, Some grammatical errors can be found. For example: On page 1: line 39, “selective constructing”. Line 31: “new strategy”. On page 4: line 8, “lead”. On page 8, line 18, “synthesis bioactive molecule analogs”.

Reviewer: 3

Comments to the Author

The present manuscript describes a novel catalytic method for the preparation of cis-1,4-disubstituted cycloalk-2-enes. The challenge is to add a non-stabilized carbon nucleophile and a stabilized nucleophile in a syn fashion to 1,3-cyclic dienes in a palladium-catalyzed reaction since the first nucleophile adds in a syn fashion and the second nucleophile (amines, stabilized carbon nucleophiles), adds in an anti-fashion resulting in an 1,4-anti-addition. The authors have solved this problem by adding the first (non-stabilized) carbon nucleophile in a radical addition leading to an allyl radical, and when this species is trapped by palladium a trans configuration between palladium and the added carbon is obtained. The subsequent amine attack on the (π-allyl)palladium intermediate formed occurs in an anti-fashion leading to an overall syn-addition of the two nucleophiles. This approach is a clever way of obtaining syn-addition when the first

nucleophile is a non-stabilized carbon nucleophile and the second nucleophile is an amine. A remarkable high diastereoselectivity is obtained in the reaction (dr > 20/1), considering that the Pd(I) attacking the allyl radical has the choice of attacking both facces of the allyl radical. Apparently, there is a high preference for Pd(I) attacking on the face opposite to that of the carbon substituent. I recommend publication of this novel and interesting paper after some revision.

1. The authors should comment on the high preference of Pd(I) to attack on the face opposite to that of the carbon substituent.

2. Could this reaction be extended to other nucleophiles than amines? Please discuss or provide preliminary results. An extension to other nucleophiles (in the second step) would further increase the importance of the method.

3. The palladium-catalyzed 1,4-addition to cyclic 1,3 dienes can provide a large number of cis-1,4-disubstituted cycloalk-2-enes via the 1,4-chloraacetoxylation. Here the author should refer to J. Am. Chem. Soc., 1985, 107, 3676. Also ref to "Palladium-Catalyzed 1,4-Additions to Conjugated Dienes", review in "Metal-catalyzed Cross-Coupling Reactions", Eds. A. de Meijere and F. Diederich, Wiley-VCH: Weinheim, 2004; pp 479-529 could be made.

4. Examples on palladium-catalyzed 1,4 addition to cyclic 1-3-dienes where an allyl silane and an Sn2 nucleophile are added in a syn-fashion are given in J. Am. Chem. Soc. 1995, 117, 560 and Chem. Eur. J. 1997, 3, 482.

4. In the title "1,4-syn-Addition of" should be "1,4-syn-Addition to"

5. On page 1, second column, 1st line: "soft nucleophiles" should be avoided since organic chemists often use it in the wrong sense. Here it is used correctly if they refer to a non-stabilized carbon nucleophile. However, I recommend that "soft nucleophiles" is replaced by "non-stabilized carbon nucleophiles".

6. On page 1, second column, 6th line: "new" should be "a new"

7. Page8, right column, 4th line: "10 folds activity than its" should be "10 folds activity compared to its"

Reviewer: 4

Comments to the Author

Journal: ACS Central Science

Manuscript ID : oc-2024-00094s
Title : Selective 1,4-syn addition of cyclic 1,3-dienes via hybrid palladium catalysis

Author(s): Liang, Y.; Bian, T.; Yadav, K.; Zhou, Q.; Zhou, L.; Sun, R.; Zhang, Z.

General Comments: Photoactivation of Pd-precatalysts is an emerging field of catalysis that opens the door to alternative hybrid Pd-radical species with potential new modes of reactivity as compared to ground-state Pd-complexes. In this work by Zhang and coworkers, the application of photoexcited Pd-catalysis to functionalized cyclic 1,3-dienes is explored with particular focus on accessing *cis*-1,4-disubstituted cyclohexane derivatives. The authors provide a logistical evolution of the initial discovery and optimization of the reaction to an impressive scope of electrophiles and nucleophiles with all examples demonstrating high diastereoselectivity (*dr* >20:1) for the *cis*-1,4disubstituted products. In an effort to understand the origin of this high diastereoselectivity, DFT calculations were performed to ascertain the energetic differences in the pathways leading to either the *cis* or *trans* stereoisomers. A mechanistic rationale was provided that involves an initial SET from the excited Pd-catalyst to the electrophile that generates an allyl radical upon addition to the 1,3-cyclic diene. Recombination of this allyl radical with the Pd-catalyst followed by outersphere S_N2' attack on the Pd-allyl species accounts for the *cis*-1,4-disubstituted products.

Overall, this method adds value and complements other approaches towards *cis*-1,4,disubstituted cyclohexane derivatives. Enthusiasm for this work, however, is diminished as the photochemically induced Pd-catalyzed 1,4-difunctionalization of 1,3-dienes is a well-established method as exemplified by the work of Glorius, Gevorgyan, and others. From this perspective, the work lacks the novelty and broad interest that would warrant publication in *ACS Central Science*. There are also additional concerns regarding the relevance of the DFT calculations provided that are not consistent with the proposed photoinduced catalytic pathway. Nonetheless, provided below are specific comments that the authors may wish to consider prior to publication elsewhere that I hope they find helpful.

Specific comments:

Manuscript formatting suggestions

1) Scheme 1 – The general reaction provided at the top of this scheme does not accurately reflect the scope of substrates listed. For example, morpholine (**S3**) is given in the general reaction scheme but products **11** and **12** obviously do not incorporate this nucleophile. Also "x" appears in **S1** and **S2** but clearly do not represent the same substituent in either of these substrates.

2) Scheme 2 – Same problem here....the general reaction at the top is not accurate. The product in the scheme contains a morpholine as the nucleophilic component but clearly this is not reflective of what's going on here.

3) Figure 1c – "migratory insertion" instead of "migration insertion" is more accurate here.

4) Figure 2 – There is no yield given for **66**. Also, in the footnotes for this figure, it appears that footnote "a" and "e" are exactly the same. There are also two "e" footnotes.

5) Figure 3 – It is extremely difficult to visualize the computed structures in this scheme as they are extremely small. Some clarity here by improving the size/resolution of these structures is recommended.

Technical suggestions and comments

1) The optimization studies provided in Table 1 identify both DMSO and DMF as the best solvents. However, the authors provide no rationale as to why the substrates in Scheme 1 were performed using DMF while the substrates presented in Scheme 2 employed DMSO. Some explanation is needed here.

2) The optimization data in Table 1 also points to K_2HPO_4 as the preferred base yet as far as I can tell, all the subsequent examples use K_3PO_4 as base in Scheme 1&2 and in Figure 2. No explanation is given for this switch.

3) Figure 2 - I can't understand why the authors present **68** and **69** as good examples of the application of this method when it produces diastereomers (*cis*-isomer) of the two APIs shown (both which are *trans*). What am I supposed to take away from these examples that give the undesired stereoisomer?

4) Figure 2 – It mentions "standard conditions" here for all the examples but does this mean DMF or DMSO as the solvent. What really are "standard conditions"? Some additional clarity here is needed.

5) Figure 3a – I have a big problem with the DFT calculations presented. First, it appears that all structures are energetically compared to the ground-state Pdcatalyst? For any of this to be relevant, these calculations need to start from the excited Pd-catalyst (most likely T_1 after

decay from the initial S_1). Plus, the barriers given from their calculations (45 kcal/mol) are completely inconsistent with a reaction that is complete in 30h at room temp (barrier ~24 kcal/mol @ rt). The authors should revamp the approach here.

6) Figure 3b – Unless I just completely missed it, I don't see any explanation in the text of the Stern-Volmer plot presented here in the text. The text also mentions radical scavenger experiments, but I cannot find any of this data in the actual manuscript itself. The reader is left to assume that all of this is in the SI (which it is) but a clear explanation as to how these data support their conclusions is lacking.

7) Despite the fact that catalysis may be photoinduced, I can imagine a mechanism where after the initial SET to the electrophile, the radical recombination with the Pd-catalyst to give a traditional Pd(II)-alkyl/aryl species is possible. This could then do an insertion into the 1,3-diene to give the corresponding Pd(II) π-allyl complex. Isomerization of this complex through Pd(0) displacement is a common mechanism of these complexes that could also explain the *cis*-1,4-disubstiution observed in this method. Do the authors have any experimental data that is inconsistent with this alternative mechanistic pathway?

Author's Response to Peer Review Comments:

DEPARTMENT OF CHEMISTRY
2545 McCarthy Mall
Honolulu, HAWAI'I, 96822
Zuxiao Zhang, PH.D.
ASSISTANT PROFESSOR
DEPARTMENT OF CHEMISTRY

Revision for Manuscript oc-2024-00094s

Dear Editor,

Selective 1,4-*syn*-Addition to Cyclic 1,3-Dienes *via* Hybrid Palladium Catalysis

I would like to extend my sincere gratitude for affording us the opportunity to revise our manuscript titled "Selective 1,4-syn-addition of Cyclic 1,3-Dienes via Hybrid Palladium Catalysis," previously submitted to "ACS Central Science" (Manuscript ID: oc-2024-00094s, disclosed at ChemRxiv while under reviewing, DOI:10.26434/chemrxiv-2024-v3dsv). I am immensely thankful to you, your esteemed colleagues, and the four expert referees for your efficient work and invaluable suggestions throughout the review process.

Please find enclosed revised versions of our manuscript and SI that address each of the comments provided by the four reviewers and editorial office. We are grateful to all the reviewers for taking the time to read and evaluate this manuscript. We sincerely appreciate their shared enthusiasm, along with their questions, comments, and helpful suggestions, which have resulted in an improved manuscript. The complete details of the manuscript revisions are described below. We hope you will agree that the additional experiments, newly revised text, and figures, along with newly added data properly address all revision requests and provide a better illustration of the significance and utility of this catalytic method for selective 1,4-*syn*-addition to cyclic 1,3-dienes.

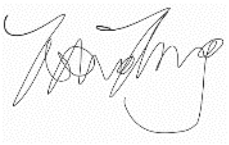

Zuxiao Zhang, Ph. D.
Assistant Professor of Chemistry

(E) zzhang9@hawaii.edu

DEPARTMENT OF CHEMISTRY
2545 McCarthy Mall
Honolulu, HAWAI'I, 96822
Zuxiao Zhang, PH.D.
ASSISTANT PROFESSOR
DEPARTMENT OF CHEMISTRY

Reviewer comments and detailed responses

Editor's comment:

Thank you for your recent submission to ACS Central Science. We have now received the reviews of your manuscript.
In its current form, your manuscript is not yet suitable for publication in ACS Central Science. The reviewers have raised points that require significant consideration and major revision that may include additional experiments/data and discussion. However, with adequate revisions, your manuscript may become acceptable for publication.
We would like to receive your revision no later than 25-Mar-2024. The revision should address the reviewers' comments and include a point-by-point response. Your manuscript may be subject to further peer review and likely sent back to one or more of the original referees.
***Note you need to directly address the following points:

Novelty relative to Glorius, Gevorgyan, and others (rev-3)

In comparison to the pioneering work by Glorius and Gevorgyan groups in the field of hybrid palladium-catalyzed difunctionalization, a notable gap in research pertains to the investigation of diastereoselectivity in the difunctionalization of cyclic conjugated dienes. While Glorius, as exemplified in the Nat Cat (ref18d) paper, has demonstrated 1,4-difunctionalization of cyclohexene, the observed diastereoselectivity remains moderate. On the other hand, Gevorgyan's contributions (ref18k) primarily revolve around the 1,2-difunctionalization of conjugated dienes. As of our current knowledge, there is a conspicuous absence of reports specifically addressing the distereoselectivity aspects of difunctionalization in cyclic 1,3-dienes within the works of Glorius, Gevorgyan, and others in the field (see the following detailed summary in hybrid palladium catalyzed difunctionalization of conjugated dienes). This represents an intriguing avenue for future research to explore and potentially enhance the stereochemical outcomes of such transformations.

(E) zzhang9@hawaii.edu

Department of Chemistry
2545 McCarthy Mall
Honolulu, Hawai'i, 96822
Zuxiao Zhang, Ph.D.
Assistant Professor
Department of Chemistry

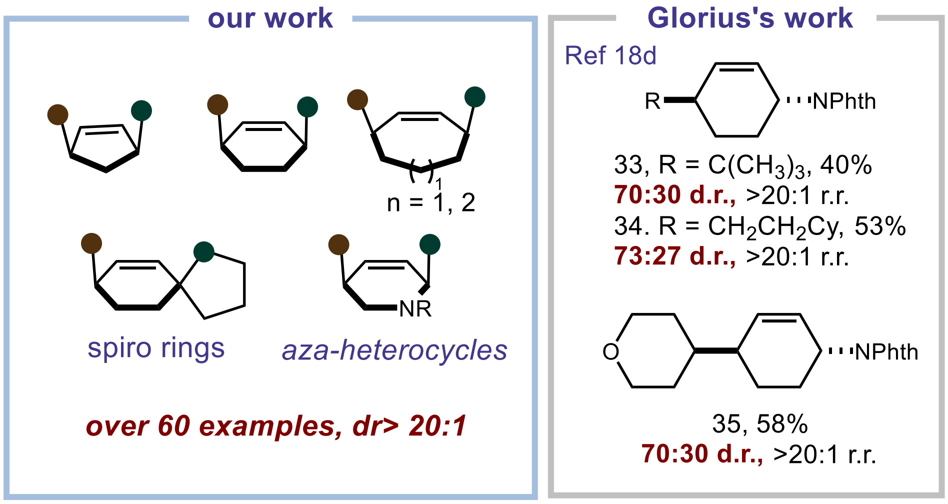

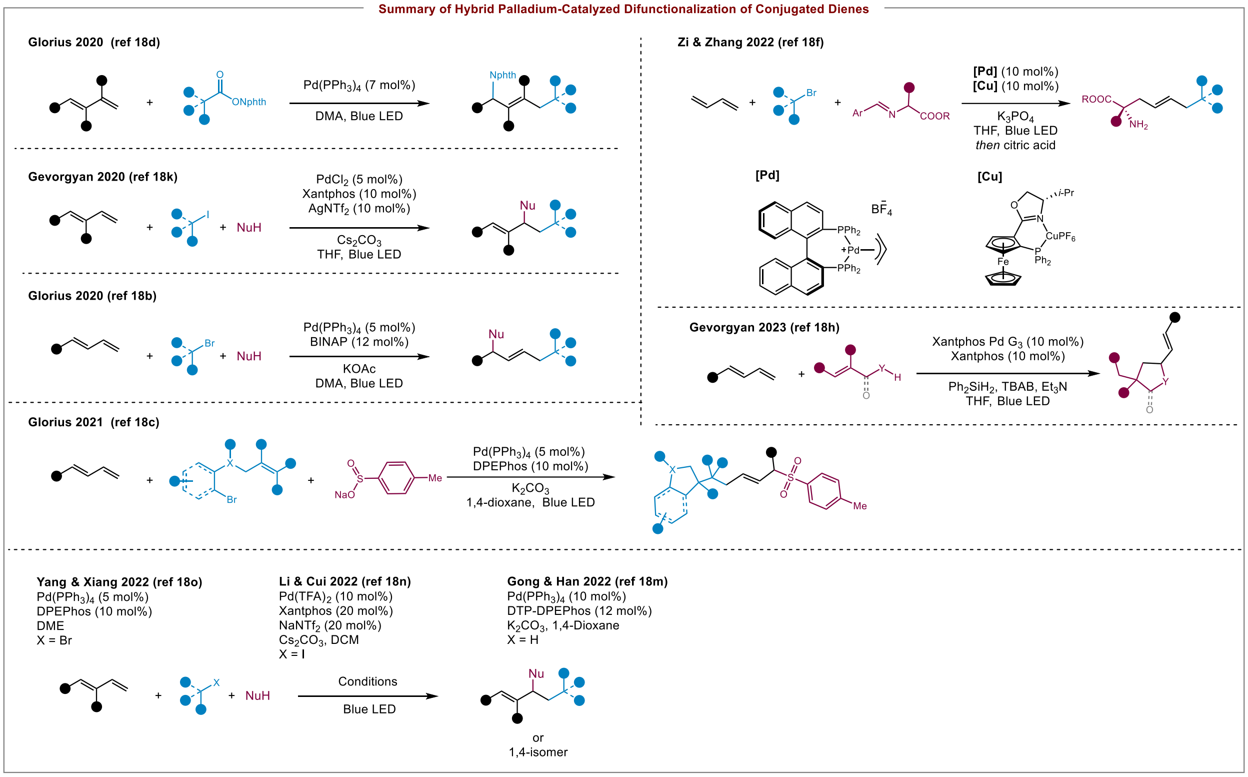

DFT calculations correctness (rev-3)

As reviewer #4 suggested, we have revamped our calculation with a more accurate method and considering the reaction starting from the excited state of the catalyst. The results are presented in the revised Fig. 3, and they tell a consistent story as before.

DEPARTMENT OF CHEMISTRY
2545 McCarthy Mall
Honolulu, HAWAI'I, 96822
Zuxiao Zhang, PH.D.
ASSISTANT PROFESSOR
DEPARTMENT OF CHEMISTRY

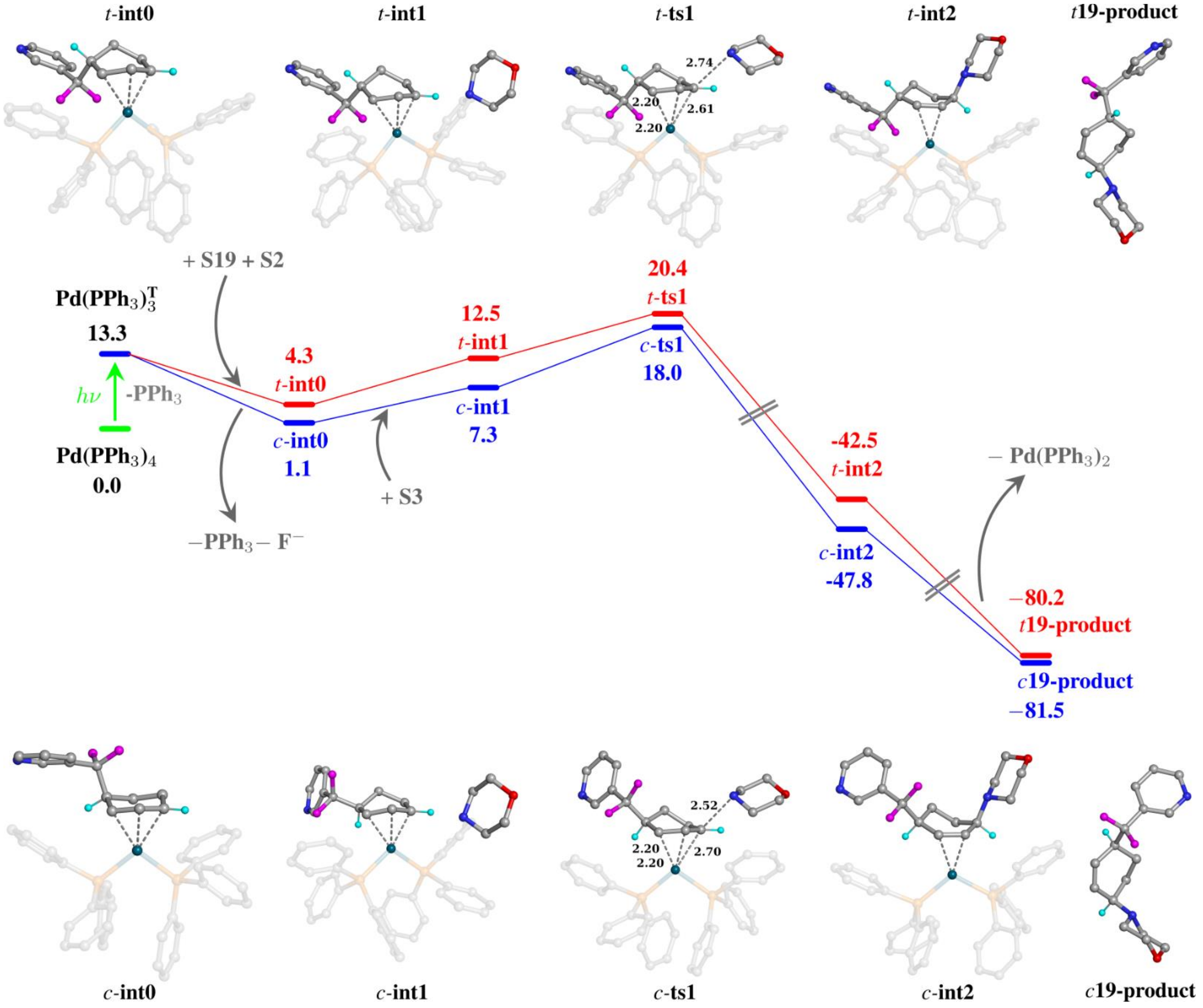

Formatting Needs:

SYNOPSIS MISSING: The- should be no more than 200 characters (including spaces) and should reasonably correlate with the TOC graphic. The synopsis is intended to explain the importance of the article to a broader readership across the sciences. Please place your synopsis in the manuscript file after the TOC graphic, and label it as "Synopsis."

The Synopsis has been added accordingly.

Department of Chemistry
2545 McCarthy Mall
Honolulu, HAWAI'I, 96822
Zuxiao Zhang, Ph.D.
Assistant Professor
Department of Chemistry

SI PG#S: The supporting information pages must be numbered consecutively, starting with page S1.

The Supporting Information pages number have been added accordingly.

Reviewer(s)' Comments to Author:

Reviewer: 1

Recommendation: **Publish in ACS Central Science after minor revisions noted.**

Comments:

- This manuscript by Zhang et al presents a remarkably general protocol for the conversion of cyclic 1,3-dienes into cis-1,4-disubstituted cycloalkenes. The scope demonstrated is extraordinarily broad, with the choice of electrophile including both aryl and alkyl halides, and the choice of nucleophile including multiple examples of carbon and nitrogen, as well as a few examples of S and O nucleophiles. The authors also demonstrate their protocol in the preparation of several biologically interesting compounds. Judging from the SI, the broad claim of >20:1 dr for all reactions seems valid. I congratulate the authors on this beautiful chemistry and enthusiastically recommend publication in ACS Central Science after modification.

Thank you for your positive comments and constructive feedback.

I feel that the introduction needs to be rewritten. The length is appropriate, but it needs to do a better job of describing the most relevant science that has led up to this study.

The introduction has been revised accordingly.

"The major challenge in achieving a general redox-neutral 1,4-syn-addition to cyclic 1,3-dienes involves reversing the conventional syn migratory insertion of the R-Pd(II)-X complex, as the S_N2' preferred anti-attack mode in the presence of amines complicates matters. Recent advancements in hybrid palladium catalysis have demonstrated notable reactivity to reduce of carbon-halogen (C-X) bonds and generate carbon-centered radicals. The formed Pd(I) species exhibit a pronounced affinity for engaging with subsequent carbon radicals, resulting in the formation of Pd(II) intermediates amenable to classic palladium chemistry.[18] Thus, we envisioned that the hybrid palladium catalysis would enable a formal stepwise anti-migratory insertion of cyclic 1,3-dienes since the steric effects favor the capture of the allylic carbon radical from the less hindered backside by Pd(I). Subsequently, the resulting allylic Pd(II) complex undergoes S_N2' nucleophilic substitution in the presence of amines, yielding 1,4-cis-carboamination products (see Figure 1d). Herein, we Present a reliable and modular protocol for synthesizing 1,4-cis-substituted cyclic

(E) zzhang9@hawaii.edu

DEPARTMENT OF CHEMISTRY
2545 McCarthy Mall
Honolulu, HAWAI'I, 96822
Zuxiao Zhang, PH.D.
ASSISTANT PROFESSOR
DEPARTMENT OF CHEMISTRY

compounds through excited palladium-catalyzed multicomponent reactions. This method could efficiently assemble a diverse array of amines, electrophiles, and cyclo-1,3-dienes into 1,4-syn-addition products with excellent regio- and diastereoselectivity."

From what I can tell, this study is truly unique in the broad range of nucleophiles and electrophiles that successfully participate. The authors even describe in the SI examples that did not work, which I appreciated.

Thank you for your positive comments and constructive feedback.

But the references are almost impossible to sort out. There are far too many references that are not really relevant to the current work (and they are bunched up under a single reference number: reference 14 and reference 18 each have close to 20 references within them!) In some cases one or two reviews are sufficient. There are numerous papers listed dealing with chemistry of dienes, and numerous papers dealing with photocatalysis. But what are the closest examples to what the authors are demonstrating?

We have streamlined the references by removing unrelated ones, retaining only the most pertinent sources as suggested.

With a bit of streamlining the introduction and a shirt discussion section at the end, this paper has the potential to be a seminal article that many will be guided by in the future. Perhaps the authors might include these two references for other approaches to cis-1,4-disubstituted cyclohexenes: Weatherford-Pratt, J.T., Smith, J.A., Bloch, J.M. et al. The double protonation of dihapto-coordinated benzene complexes enables dearomatization using aromatic nucleophiles. Nat Commun 14, 3145 (2023). Justin T. Weatherford-Pratt et al.,Tungsten-anisole complex provides 3,6-substituted cyclohexenes for highly diversified chemical libraries. Sci. Adv.10, eadl0885(2024).

We thank this reviewer very much for the valuable suggestion. The suggested references have been added as Ref 10b and 10c.

(E) zzhang9@hawaii.edu

DEPARTMENT OF CHEMISTRY
2545 McCarthy Mall
Honolulu, HAWAI'I, 96822
Zuxiao Zhang, PH.D.
ASSISTANT PROFESSOR
DEPARTMENT OF CHEMISTRY

The DFT and the Eyring kinetics is a nice addition, but aside from a summarizing sentence or two, probably belongs in the SI. There is nothing unusual going on here.

We thank this reviewer very much for the valuable suggestion. In the revised manuscript, we have shortened the computational section and moved the detailed methodologies to the Supplementary Information. This adjustment ensures a focused presentation of the main findings while providing access to additional technical details in the SI, as recommended. The revised computational section reads:

*"To understand the nature of the facial selectivity observed in the experiment, density functional theory (DFT) calculation was employed to calculate the energy profile of the reaction (details in the Supplementary Materials). As shown in Fig. 3, the trifluoromethylated arene carbon radical reacts with the 1,3-diene moiety to give the allylic carbon radical, which coor-dinates with Pd(PPh3)2 via π-π interaction (**int0**). The nucleo-phile attacks int0 from the opposite direction of the catalyst, via a classical SN2' mechanism that involves a pre-reaction complex (**int1**), a Walden inversion transition state (**ts1**), post-reaction complex (**int2**), and dissociated products (**product**). As the figure shows, the trans-pathway has overall higher en-ergies than the cis-pathway with the rate-limiting step (**t-ts1** vs. **c-ts1**) 2.4 kcal/mol above. According to the Curtin-Hammett principle and the Eyring-Polanyi equation, this level of differ-ence results in a ratio of t-**product** vs. c-**product** 1:57, aligning closely with experimental findings."*

Additional Questions:

Quality of experimental data, technical rigor: Top 5%

Significance to chemistry researchers in this and related fields: Top 5%

Broad interest to other researchers: Top 5%

Novelty: Top 5%

Is this research study suitable for media coverage or a First Reactions (a News & Views piece in

(E) zzhang9@hawaii.edu

DEPARTMENT OF CHEMISTRY
2545 McCarthy Mall
Honolulu, HAWAI'I, 96822
Zuxiao Zhang, PH.D.
ASSISTANT PROFESSOR
DEPARTMENT OF CHEMISTRY

the journal)?: Yes

Reviewer: 2

Recommendation: **Publish in ACS Central Science after minor revisions noted**.

Comments:

The manuscript by Zhang describes an excited Pd-catalyzed three-component reaction of cyclic 1,3-dienes, alkyl/aryl halides, and amines. The reaction forms 1,4-cis-substituted cyclic framework. Other nucleophiles in addition to amines, including C- and O-nucleophiles, were also compatible. A hybrid palladium-catalyzed radical-polar crossover mechanism was proposed for the reaction. A wide array of halides, including trifluoromethyl arenes, aryl bromides, and alkyl iodides, underwent the three-component reaction. The reaction provides a modular approach for the synthesis of 1,4-cis-substituted cyclic compounds. I recommend this manuscript to be accepted for publication in ACS central science after some issues as below are addressed.

Thank you for your positive comments and constructive feedback.

1, For the trifluoromethylarene scope, the substrates were limited to benzenes bearing two trifluoromethyl groups and trifluoromethylpyridine derivatives. How about trifluoromethylbenzene and its simple, and other heteroarenes.

Regrettably, trifluoromethylbenzene was attempted but proved ineffective under the optimized conditions, potentially due to unmatched reduction potential. Various heteroarenes were also explored, and the examples of unsuccessful substrate are provided in the Supplementary Information (part II, page S3).

2, For products **5-10**, the evidences should be provided to prove the regioselectivity.

The single crystal structure of compound **70**, derived from product **5**, has been successfully obtained. The X-ray analysis definitively confirms the regio- and diastereoselectivity of compounds **5**, along with its analogs **6-10**.

(E) zzhang9@hawaii.edu

DEPARTMENT OF CHEMISTRY
2545 McCarthy Mall
Honolulu, HAWAI'I, 96822
Zuxiao Zhang, PH.D.
ASSISTANT PROFESSOR
DEPARTMENT OF CHEMISTRY

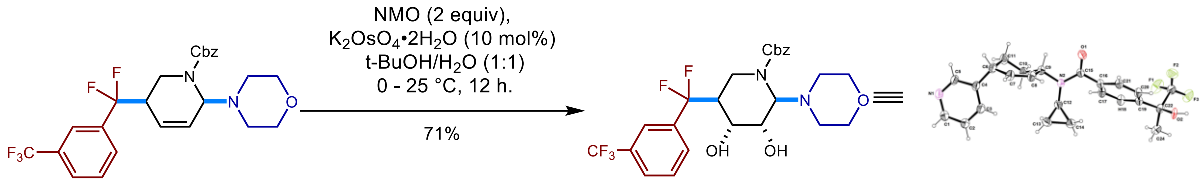

3, Some grammatical errors can be found. For example: On page 1: line 39, “selective constructing”. Line 31: “new strategy”. On page 4: line 8, “lead”. On page 8, line 18, “synthesis bioactive molecule analogs”.

The grammatical errors have been corrected accordingly.

Additional Questions:

Quality of experimental data, technical rigor: High

Significance to chemistry researchers in this and related fields: Top 5%

Broad interest to other researchers: Top 5%

Novelty: High

Is this research study suitable for media coverage or a First Reactions (a News & Views piece in the journal)?: No

Reviewer: 3

Recommendation: **Publish in ACS Central Science after minor revisions noted**.

Comments:

The present manuscript describes a novel catalytic method for the preparation of cis-1,4-disubstituted cycloalk-2-enes. The challenge is to add a non-stabilized carbon nucleophile and a stabilized nucleophile in a syn fashion to 1,3-cyclic dienes in a palladium-catalyzed reaction since the first nucleophile adds in a syn fashion and the second nucleophile (amines, stabilized carbon nucleophiles), adds in an anti-fashion resulting in an 1,4-anti-addition. The authors have solved this problem by adding the first (non-stabilized) carbon nucleophile in a radical addition leading to an allyl radical, and when this species is trapped by palladium a trans configuration between

(E) zzhang9@hawaii.edu

DEPARTMENT OF CHEMISTRY
2545 McCarthy Mall
Honolulu, HAWAI'I, 96822
Zuxiao Zhang, PH.D.
ASSISTANT PROFESSOR
DEPARTMENT OF CHEMISTRY

palladium and the added carbon is obtained. The subsequent amine attack on the (π-allyl)palladium intermediate formed occurs in an anti-fashion leading to an overall syn-addition of the two nucleophiles. This approach is a clever way of obtaining syn-addition when the first nucleophile is a non-stabilized carbon nucleophile and the second nucleophile is an amine. A remarkable high diastereoselectivity is obtained in the reaction (dr > 20/1), considering that the Pd(I) attacking the allyl radical has the choice of attacking both facces of the allyl radical. Apparently, there is a high preference for Pd(I) attacking on the face opposite to that of the carbon substituent. I recommend publication of this novel and interesting paper after some revision.

Thank you for your positive comments and constructive feedback.

1. The authors should comment on the high preference of Pd(I) to attack on the face opposite to that of the carbon substituent.

The pronounced preference of Pd(I) to attack the face opposite the carbon substituent is likely a result of steric effects, where the substituent blocks the same face of the cyclic diene. This insightful comment has been incorporated into the reaction design section.

2. Could this reaction be extended to other nucleophiles than amines? Please discuss or provide preliminary results. An extension to other nucleophiles (in the second step) would further increase the importance of the method.

Other nucleophiles, such as carbon, oxygen, and sulfur nucleophiles, were explored beyond amines (see Original Scheme 2, products **60-64**).

3. The palladium-catalyzed 1,4-addition to cyclic 1,3 dienes can provide a large number of cis-1,4-disubstituted cycloalk-2-enes via the 1,4-chloraacetoxylation. Here the author should refer to J. Am. Chem. Soc., 1985, 107, 3676. Also ref to “Palladium-Catalyzed 1,4-Additions to Conjugated Dienes”, review in “Metal-catalyzed Cross-Coupling Reactions”, Eds. A. de Meijere and F. Diederich, Wiley-VCH: Weinheim, 2004; pp 479-529 could be made.

The suggested references have been added as suggested. The suggested *J. Am. Chem. Soc.*, **1985**, *107*, 3676. has been cited as ref 15b. The suggested review in “Metal-catalyzed Cross-Coupling

(E) zzhang9@hawaii.edu

Department of Chemistry
2545 McCarthy Mall
Honolulu, Hawai'i, 96822
Zuxiao Zhang, Ph.D.
Assistant Professor
Department of Chemistry

Reactions", Eds. A. de Meijere and F. Diederich, Wiley-VCH: Weinheim, 2004; pp 479-529 has been added as ref 14d

4. Examples on palladium-catalyzed 1,4 addition to cyclic 1-3-dienes where an allyl silane and an Sn2 nucleophile are added in a syn-fashion are given in J. Am. Chem. Soc. 1995, 117, 560 and Chem. Eur. J. 1997, 3, 482.

The suggested references have now been added as ref 15d and ref 15e.

4. In the title "1,4-syn-Addition of" should be "1,4-syn-Addition to"

The error has been corrected as suggested.

5. On page 1, second column, 1st line: "soft nucleophiles" should be avoided since organic chemists often use it in the wrong sense. Here it is used correctly if they refer to a non-stabilized carbon nucleophile. However, I recommend that "soft nucleophiles" is replaced by "non-stabilized carbon nucleophiles".

The "soft nucleophiles" has been replaced by "non-stabilized carbon nucleophiles" as suggested.

6. On page 1, second column, 6th line: "new" should be "a new"

The error has been corrected as suggested.

7. Page8, right column, 4th line: "10 folds activity than its" should be "10 folds activity compared to its"

It has been corrected as suggested.

Additional Questions:

Quality of experimental data, technical rigor: High

Significance to chemistry researchers in this and related fields: High

Broad interest to other researchers: High

(E) zzhang9@hawaii.edu

DEPARTMENT OF CHEMISTRY
2545 McCarthy Mall
Honolulu, HAWAI'I, 96822
Zuxiao Zhang, PH.D.
ASSISTANT PROFESSOR
DEPARTMENT OF CHEMISTRY

Novelty: Top 5%

Is this research study suitable for media coverage or a First Reactions (a News & Views piece in the journal)?: No

Reviewer: 4

Recommendation: Does not meet the requirements of publishing in ACS Central Science.

Comments:

General Comments: Photoactivation of Pd-precatalysts is an emerging field of catalysis that opens the door to alternative hybrid Pd-radical species with potential new modes of reactivity as compared to ground-state Pd-complexes. In this work by Zhang and co-workers, the application of photoexcited Pd-catalysis to functionalized cyclic 1,3-dienes is explored with particular focus on accessing cis-1,4-disubstituted cyclohexane derivatives. The authors provide a logistical evolution of the initial discovery and optimization of the reaction to an impressive scope of electrophiles and nucleophiles with all examples demonstrating high diastereoselectivity (dr >20:1) for the cis-1,4-disubstituted products. In an effort to understand the origin of this high diastereoselectivity, DFT calculations were performed to ascertain the energetic differences in the pathways leading to either the cis or trans stereoisomers. A mechanistic rationale was provided that involves an initial SET from the excited Pd-catalyst to the electrophile that generates an allyl radical upon addition to the 1,3-cyclic diene. Recombination of this allyl radical with the Pd-catalyst followed by outersphere SN2' attack on the Pd-allyl species accounts for the cis-1,4-disubstituted products. Overall, this method adds value and complements other approaches towards cis-1,4,-disubstituted cyclohexane derivatives. Enthusiasm for this work, however, is diminished as the photochemically induced Pd-catalyzed 1,4-difunctionalization of 1,3-dienes is a well-established method as exemplified by the work of Glorius, Gevorgyan, and others. From this perspective, the work lacks the novelty and broad interest that would warrant publication in ACS Central Science. There are also additional concerns regarding the relevance of the DFT calculations provided that are not consistent with the proposed photoinduced catalytic pathway. Nonetheless, provided below are specific comments that the authors may wish to consider prior to publication elsewhere that I hope they find helpful.

(E) zzhang9@hawaii.edu

DEPARTMENT OF CHEMISTRY
2545 McCarthy Mall
Honolulu, HAWAI'I, 96822
Zuxiao Zhang, PH.D.
ASSISTANT PROFESSOR
DEPARTMENT OF CHEMISTRY

We appreciate the reviewer's comments and constructive feedback. However, we respectfully disagree with the assertion that our work, focusing on the selective 1,4-syn-addition to cyclic 1,3-dienes, lacks novelty in light of the contributions by Glorius, Gevorgyan, and others in the difunctionalization of conjugated dienes. In comparison to the pioneering work by Glorius and Gevorgyan groups in the field of hybrid palladium-catalyzed difunctionalization, a notable gap in research pertains to the investigation of diastereoselectivity in the difunctionalization of cyclic conjugated dienes. While Glorius, as exemplified in the Nat Cat paper (ref 18d), has demonstrated 1,4-difunctionalization of cyclohexene, the observed diastereoselectivity remains moderate. On the other hand, Gevorgyan's contributions (ref 18k) primarily revolve around the 1,2-difunctionalization of conjugated dienes. As of our current knowledge, there is a conspicuous absence of reports specifically addressing the distereoselectivity aspects of difunctionalization in cyclic 1,3-dienes within the works of Glorius, Gevorgyan, and others in the field. This represents an intriguing avenue for future research to explore and potentially enhance the stereochemical outcomes of such transformations.

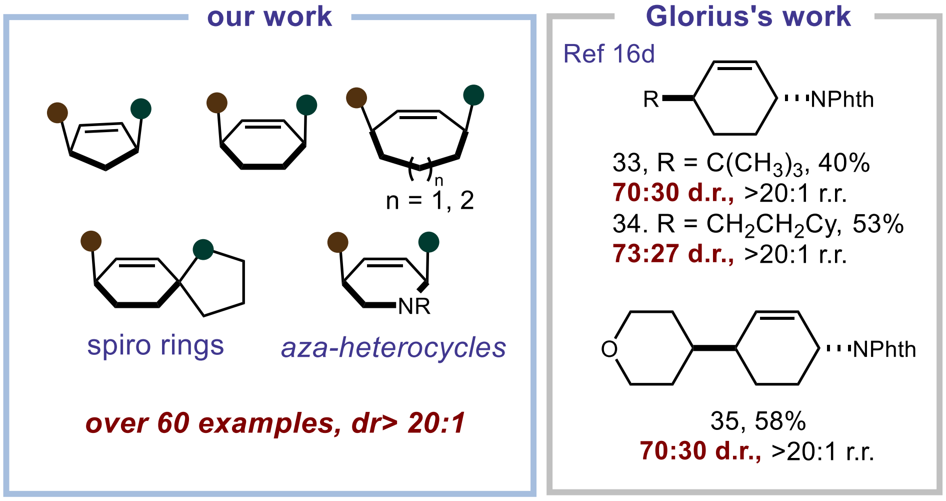

(E) zzhang9@hawaii.edu

DEPARTMENT OF CHEMISTRY
2545 McCarthy Mall
Honolulu, HAWAI'I, 96822
Zuxiao Zhang, PH.D.
ASSISTANT PROFESSOR
DEPARTMENT OF CHEMISTRY

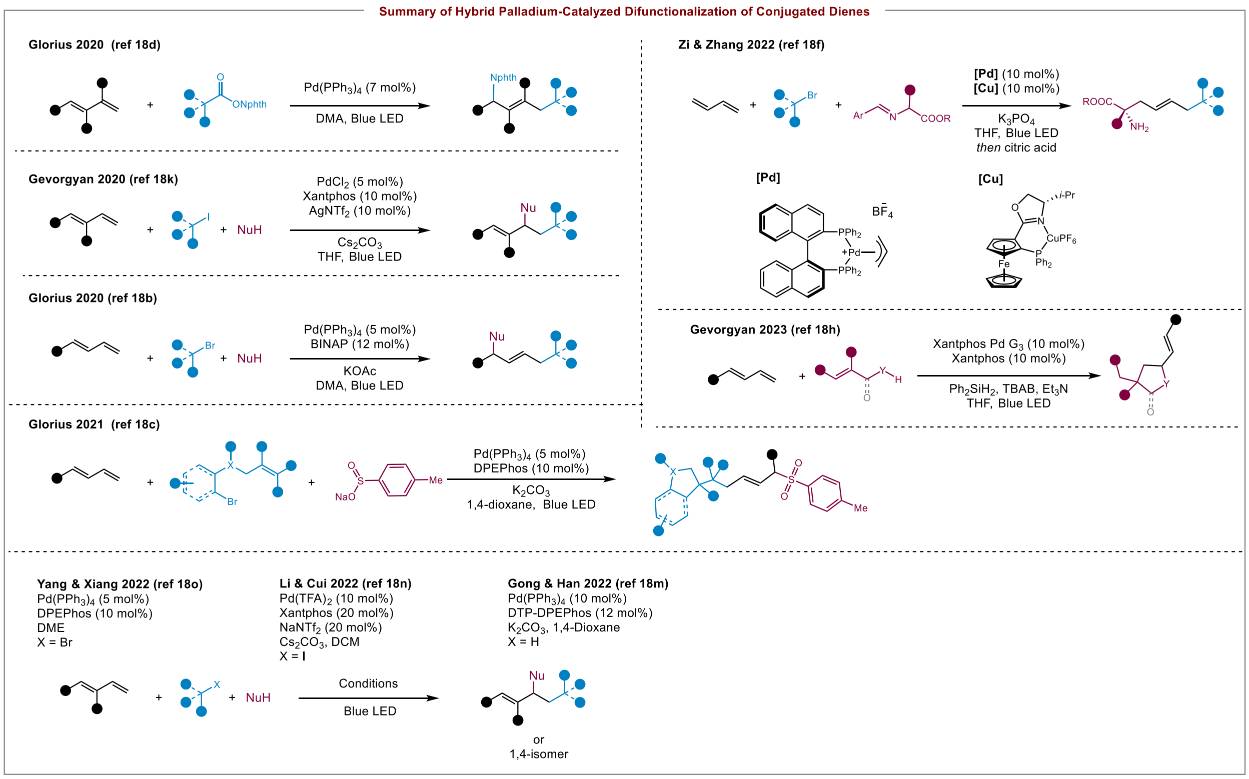

Specific comments:

Manuscript formatting suggestions

1) Scheme 1 – The general reaction provided at the top of this scheme does not accurately reflect the scope of substrates listed. For example, morpholine (S3) is given in the general reaction scheme but products 11 and 12 obviously do not incorporate this nucleophile. Also “x” appears in S1 and S2 but clearly do not represent the same substituent in either of these substrates.

We have addressed the discrepancy by providing specific details in note b for products 11 and 12 regarding the starting material. Additionally, we have rectified the potential confusion by replacing 'x' with 'z' in the ring structure to accurately represent the substituent, as detailed in the revised Scheme 1."

2) Scheme 2 – Same problem here....the general reaction at the top is not accurate. The product in the scheme contains a morpholine as the nucleophilic component but clearly this is not reflective of what’s going on here.

(E) zzhang9@hawaii.edu

Department of Chemistry
2545 McCarthy Mall
Honolulu, HAWAI'I, 96822
Zuxiao Zhang, Ph.D.
Assistant Professor
Department of Chemistry

We have rectified the discrepancy by replacing 'morpholine' with '**Nu**' in the general reaction at the top to accurately reflect the nucleophilic component in the Scheme 2.

3) Figure 1c – “migratory insertion” instead of “migration insertion” is more accurate here.
The “migration insertion” has been replaced by “migratory insertion” as suggested.

4) Figure 2 – There is no yield given for 66. Also, in the footnotes for this figure, it appears that footnote “a” and “e” are exactly the same. There are also two “e” footnotes.
The yield for compound 66 has now been included in Figure 2a. The duplicate footnote 'e' has been removed, and the footnotes 'a' and 'e' have been revised to ensure accurate and distinct information.

5) Figure 3 – It is extremely difficult to visualize the computed structures in this scheme as they are extremely small. Some clarity here by improving the size/resolution of these structures is recommended.
We have enhanced the visibility and clarity of the computed structures in the computational studies (see new Figure 3) by adjusting their size and resolution, as recommended.

Technical suggestions and comments
1) The optimization studies provided in Table 1 identify both DMSO and DMF as the best solvents. However, the authors provide no rationale as to why the substrates in Scheme 1 were performed using DMF while the substrates presented in Scheme 2 employed DMSO. Some explanation is needed here.
We apologize for any confusion. The standard conditions for the experiments involve utilizing DMSO as the solvent and K_2HPO_4 as the base. We have corrected both Scheme 1 and Scheme 2 to accurately reflect these standard conditions unless otherwise noted in footnotes.

(E) zzhang9@hawaii.edu

DEPARTMENT OF CHEMISTRY
2545 McCarthy Mall
Honolulu, HAWAI'I, 96822
Zuxiao Zhang, PH.D.
ASSISTANT PROFESSOR
DEPARTMENT OF CHEMISTRY

2) The optimization data in Table 1 also points to K2HPO4 as the preferred base yet as far as I can tell, all the subsequent examples use K3PO4 as base in Scheme 1&2 and in Figure 2. No explanation is given for this switch.

We apologize for any confusion. Both Schemes and Figure 2 have been rectified accordingly to reflect the consistent use of K_2HPO_4.

3) Figure 2 - I can't understand why the authors present 68 and 69 as good examples of the application of this method when it produces diastereomers (cis-isomer) of the two APIs shown (both which are trans). What am I supposed to take away from these examples that give the undesired stereoisomer?

We aim to highlight that our method offers a convenient route to analogs of the APIs, which might be challenging to access through alternative means.

4) Figure 2 – It mentions "standard conditions" here for all the examples but does this mean DMF or DMSO as the solvent. What really are "standard conditions"? Some additional clarity here is needed.

Depending on the different electrophiles, the conditions are slightly modified. We have replaced the standard conditions with more specified general procedures which could be found the details in SI.

5) Figure 3a – I have a big problem with the DFT calculations presented. First, it appears that all structures are energetically compared to the ground-state Pd-catalyst? For any of this to be relevant, these calculations need to start from the excited Pd-catalyst (most likely T1 after decay from the initial S1). Plus, the barriers given from their calculations (45 kcal/mol) are completely inconsistent with a reaction that is complete in 30h at room temp (barrier ~24 kcal/mol @ rt). The authors should revamp the approach here.

Department of Chemistry
2545 McCarthy Mall
Honolulu, HAWAI'I, 96822
Zuxiao Zhang, Ph.D.
Assistant Professor
Department of Chemistry

We thank the reviewer for this comment. As suggested, we have revamped our computational study with a more accurate method and incorporated the energy of the excited Pd(0) catalyst as a reference point. Our analysis now reveals an energy barrier of 18 kcal/mol, which aligns with the experimental observation of the reaction completed within 30 hours at room temperature.

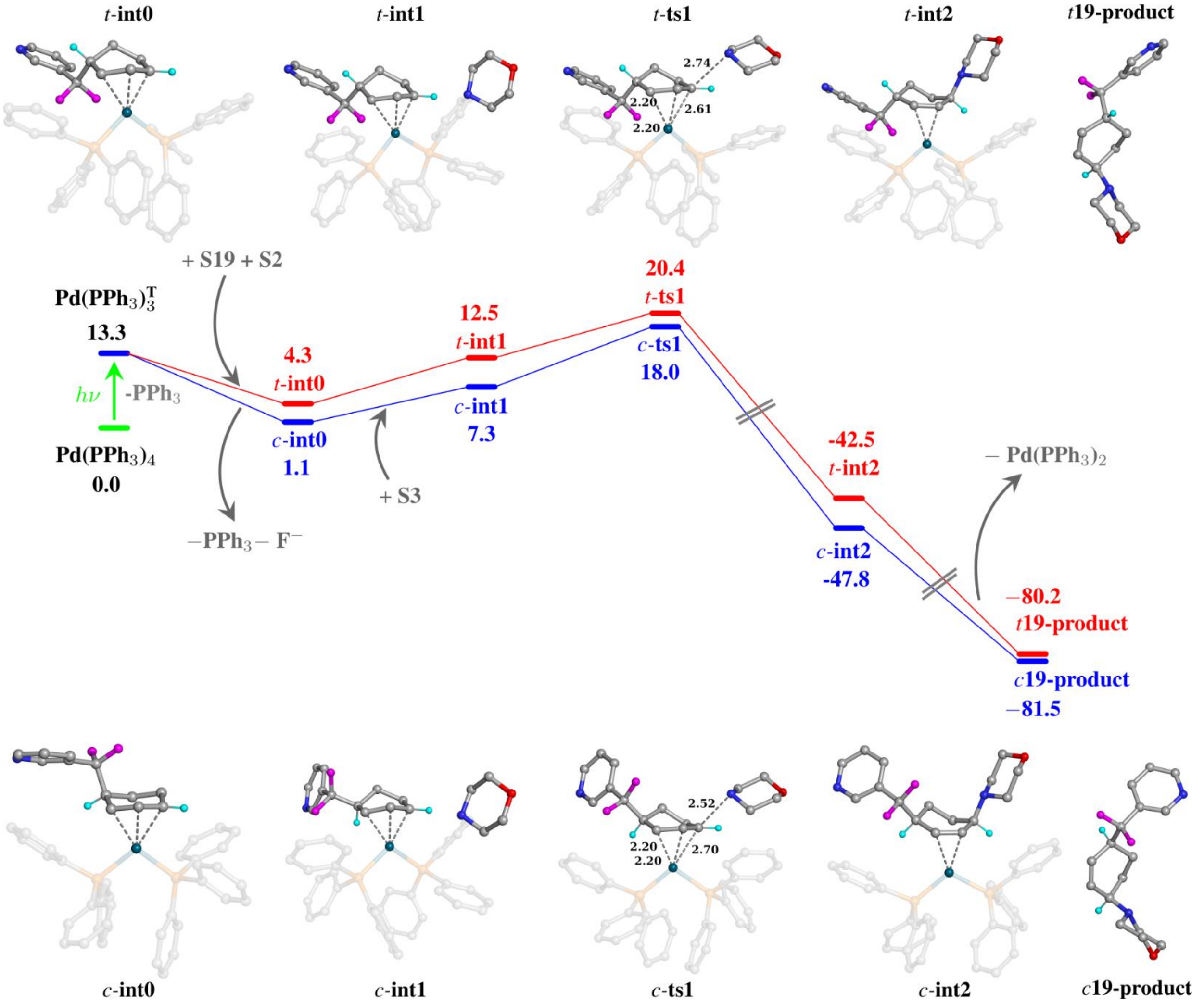

6) Figure 3b – Unless I just completely missed it, I don't see any explanation in the text of the Stern-Volmer plot presented here in the text. The text also mentions radical scavenger experiments, but I cannot find any of this data in the actual manuscript itself. The reader is left to assume that all of this is in the SI (which it is) but a clear explanation as to how these data support their conclusions is lacking.

DEPARTMENT OF CHEMISTRY
2545 McCarthy Mall
Honolulu, HAWAI'I, 96822
Zuxiao Zhang, PH.D.
ASSISTANT PROFESSOR
DEPARTMENT OF CHEMISTRY

We have incorporated an explanation of the Stern-Volmer plot, and the details of radical scavenger experiments are now provided in the manuscript as Figure 3a. This additional information clarifies the role of these experiments in supporting our conclusions, addressing any potential ambiguity in the original presentation.

"The computation supports the predominance of kinetically controlled cis configuration products, aligning closely with experimental findings. Control experiments utilizing TEMPO as a radical scavenger yielded no product formation, with the observation of the ArCF$_2$-TEMPO adduct (Figure 4a). Furthermore, Stern-Volmer quenching experiments (Figure 4b) revealed that only S1 effectively quenches the excited Pd(0) catalyst. Integrating these findings from radical scavenger experiments, Stern-Volmer results, and computational studies, we propose the following mechanism (see Figure 4c)."

7) Despite the fact that catalysis may be photoinduced, I can imagine a mechanism where after the initial SET to the electrophile, the radical recombination with the Pd-catalyst to give a traditional Pd(II)-alkyl/aryl species is possible. This could then do an insertion into the 1,3-diene to give the corresponding Pd(II) -allyl complex. Isomerization of this complex through Pd(0) displacement is a common mechanism of these complexes that could also explain the cis-1,4-disubstiution observed in this method. Do the authors have any experimental data that is inconsistent with this alternative mechanistic pathway?

While we cannot entirely rule out the possibility of a mechanism involving isomerization, our analysis considers previous reported 1,4-functionalization of cyclic 1,3-dienes (1. *J. Am. Chem. Soc*. **1987**, *109*, 6396-6403. 2. *J. Org. Chem.* **1979**, *44*(6):918-921. doi:10.1021/JO01320A005. 3. *J. Am. Chem. Soc*. **2020**, *142*, 18341–18345. doi:10.1021/jacs.0c10615.). Traditional Pd(II)-alkyl/aryl species insertion into the diene typically exhibits diastereoselectivity preferences for anti-addition or a mixture, which differs from our observed results. Although the catalysis may be photoinduced, there is currently no experimental data inconsistent with our proposed stepwise migratory insertion mechanism. We appreciate the insightful suggestion and will carefully

(E) zzhang9@hawaii.edu

DEPARTMENT OF CHEMISTRY
2545 McCarthy Mall
Honolulu, HAWAI'I, 96822
Zuxiao Zhang, PH.D.
ASSISTANT PROFESSOR
DEPARTMENT OF CHEMISTRY

consider additional experiments to further elucidate the mechanistic pathway.

(E) zzhang9@hawaii.edu

oc-2024-00094s.R2

Name: Peer Review Information for "Selective 1,4-syn-addition to Cyclic 1,3-Dienes via Hybrid Palladium Catalysis"

Second Round of Reviewer Comments

Reviewer: 3

Comments to the Author

The manuscript has been revised in a satisfactory manner and can now be published

Reviewer: 2

Comments to the Author

All the issues raised by the reviewers have been addressed. I recommend this manuscript to be accepted for publication in ACS central science.

Some minor errors: Page 1, right column, line 45, "to reduce of"; line 56, "Present" (should not be capital), Page 5, right column, line 7, "trans" (should be italic)

Reviewer: 1

Comments to the Author

With the suggested modifications to this manuscript, I feel that it meets the standards of publication in ACS Central Science. I stand by my earlier review that this work represents a general protocol for the conversion of cyclic 1,3-dienes into cis-1,4-disubstituted cycloalkenes. The scope demonstrated is extraordinarily broad, with the choice of electrophile including both aryl and alkyl halides, and the choice of nucleophile including multiple examples of carbon and nitrogen, as well as a few examples of S and O nucleophiles. The argument that this work is just an extension of Glorius and Gevorgyan's earlier studies dismisses the significant advance of achieving highly dependable cis-stereochemistry for cyclic dienes.

Reviewer: 4

Comments to the Author

The authors have made a good faith effort to address my previous concerns regarding several of the figures, confusions surrounding the "standard conditions", and the starting point for the DFT calculations. My harshest critique regarding the lack of novelty still remains. While I agree with the authors and the other reviewers that the method presented here has synthetic value, I do not feel that emphasis on controlling diastereoselectivity in the Pd-catalyzed 1,4-difunctionalization of cyclic dienes represents a huge leap forward. Again, the Pd-catalyzed syn 1,4-difunctionalization of cyclic 1,3-dienes was demonstrated by Backvall almost 30 years ago. Therefore, I view it as an incremental (yet valuable) advance that is worthy of publication in a more specialized journal.

Author's Response to Peer Review Comments:

DEPARTMENT OF CHEMISTRY
2545 McCarthy Mall
Honolulu, HAWAI'I, 96822
Zuxiao Zhang, PH.D.
ASSISTANT PROFESSOR
DEPARTMENT OF CHEMISTRY

Revision for Manuscript oc-2024-00094s.R1

Editor

Selective 1,4-syn-addition to Cyclic 1,3-Dienes via Hybrid Palladium Catalysis

Please find enclosed revised versions of our manuscript and SI that address each of the comments provided by the four reviewers and editorial office. We are grateful to all the reviewers for taking the time to read and evaluate this manuscript. We sincerely appreciate their shared enthusiasm, along with their questions, comments, and helpful suggestions, which have resulted in an improved manuscript. The complete details of the manuscript revisions are described below. We trust that our revisions adequately address the comments provided by the reviewers. We are optimistic that the modifications outlined above align with the publication standards set by ACS Central Science. We are deeply grateful for your continued assistance with our manuscript.

Sincerely,

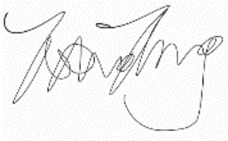

Zuxiao Zhang, Ph. D.
Assistant Professor of Chemistry

(E) zzhang9@hawaii.edu

DEPARTMENT OF CHEMISTRY
2545 McCarthy Mall
Honolulu, HAWAI'I, 96822
Zuxiao Zhang, PH.D.
ASSISTANT PROFESSOR
DEPARTMENT OF CHEMISTRY

Reviewer comments and detailed responses

Editor's comment:

Dear Dr. Zhang:

Thank you for your recent submission to ACS Central Science. We have now received the reviews for your manuscript and I am pleased to inform you they were quite positive.

However, the reviewers have raised important points that require attention and must be addressed before a final decision can be made.

Please make the appropriate changes to your manuscript and submit a revised manuscript no later than 14-May-2024. Your manuscript may be subject to further peer review but if the revision can thoroughly address the outstanding concerns, we aim to minimize further back-and-forth correspondence between authors and referees and make editorial decisions in house, which will save time and effort for all and expedite processing of your paper.

Formatting Needs:

AU EMAIL: Please include the email address of the corresponding author on the first page of the manuscript, and the Supporting Information if submitted, with an asterisk next to their name in the author list. Please be sure to label "email."

It has been addressed accordingly.

SECTION HEADERS: Please note that section headers are required for the sections in all manuscript types as discussed in the Author Guide. Abstract, Introduction, Results and Discussion, and Conclusions, Methods and Acknowledgment are usually acceptable section headers. However,

(E) zzhang9@hawaii.edu

DEPARTMENT OF CHEMISTRY
2545 McCarthy Mall
Honolulu, HAWAI'I, 96822
Zuxiao Zhang, PH.D.
ASSISTANT PROFESSOR
DEPARTMENT OF CHEMISTRY

if other section headers are more relevant for the material, authors are encouraged to use alternatives, with the exception of the ABSTRACT.

It has been addressed accordingly.

HIGHLIGHTING: Please remove the highlighting in the text of both the Manuscript and Supporting Information files, and upload "clean" copies for publication. You may upload annotated files separately as "Supporting Information for Review Only" files. It has been addressed accordingly.

SI PARAGRAPH: If the manuscript is accompanied by any supporting information for publication, a brief description of the supplementary material is required in the manuscript. The appropriate format is: Supporting Information. Brief statement in non-sentence format listing the contents of the material supplied as Supporting Information.

It has been addressed accordingly.

SI HEADER: The title and author list on the first page of the SI for Pub file must match the title and author list of the manuscript.

It has been addressed accordingly.

Reviewer(s)' Comments to Author:

Reviewer: 3

Recommendation: Publish in ACS Central Science without change.

(E) zzhang9@hawaii.edu

DEPARTMENT OF CHEMISTRY
2545 McCarthy Mall
Honolulu, HAWAI'I, 96822
Zuxiao Zhang, PH.D.
ASSISTANT PROFESSOR
DEPARTMENT OF CHEMISTRY

Comments:
The manuscript has been revised in a satisfactory manner and can now be published

Thank you for your positive comments.

Additional Questions:

Quality of experimental data, technical rigor: High

Significance to chemistry researchers in this and related fields: High

Broad interest to other researchers: High

Novelty: Top 5%

Is this research study suitable for media coverage or a First Reactions (a News & Views piece in the journal)?: No

Reviewer: 2

Recommendation: Publish in ACS Central Science after minor revisions noted.

Comments:
All the issues raised by the reviewers have been addressed. I recommend this manuscript to be accepted for publication in ACS central science.
Thank you for your positive comments.

(E) zzhang9@hawaii.edu

Department of Chemistry
2545 McCarthy Mall
Honolulu, HAWAI'I, 96822
Zuxiao Zhang, Ph.D.
Assistant Professor
Department of Chemistry

Some minor errors: Page 1, right column, line 45, "to reduce of"; line 56, "Present" (should not be capital), Page 5, right column, line 7, "trans" (should be italic)

We thank this reviewer very much for the effort to point out the minor errors. All the issues have been addressed accordingly.

Additional Questions:

Quality of experimental data, technical rigor: Top 5%

Significance to chemistry researchers in this and related fields: Top 5%

Broad interest to other researchers: Top 5%

Novelty: Top 5%

Is this research study suitable for media coverage or a First Reactions (a News & Views piece in the journal)?: No

Reviewer: 1

Recommendation: Publish in ACS Central Science without change.

Comments:
With the suggested modifications to this manuscript, I feel that it meets the standards of publication in ACS Central Science. I stand by my earlier review that this work represents a general protocol for the conversion of cyclic 1,3-dienes into cis-1,4-disubstituted cycloalkenes. The scope demonstrated is extraordinarily broad, with the choice of electrophile including both aryl and alkyl

(E) zzhang9@hawaii.edu

Department of Chemistry
2545 McCarthy Mall
Honolulu, Hawai'i, 96822
Zuxiao Zhang, Ph.D.
Assistant Professor
Department of Chemistry

halides, and the choice of nucleophile including multiple examples of carbon and nitrogen, as well as a few examples of S and O nucleophiles. The argument that this work is just an extension of Glorius and Gevorgyan's earlier studies dismisses the significant advance of achieving highly dependable cis-stereochemistry for cyclic dienes.

Thank you for your positive comments.

Additional Questions:

Quality of experimental data, technical rigor: Top 5%

Significance to chemistry researchers in this and related fields: Top 5%

Broad interest to other researchers: Top 5%

Novelty: Top 5%

Is this research study suitable for media coverage or a First Reactions (a News & Views piece in the journal)?: Yes

Reviewer: 4

Recommendation: Publish elsewhere

Comments:
The authors have made a good faith effort to address my previous concerns regarding several of the figures, confusions surrounding the "standard conditions", and the starting point for the DFT calculations. My harshest critique regarding the lack of novelty still remains. While I agree with

(E) zzhang9@hawaii.edu

Department of Chemistry
2545 McCarthy Mall
Honolulu, Hawai'i, 96822
Zuxiao Zhang, Ph.D.
Assistant Professor
Department of Chemistry

the authors and the other reviewers that the method presented here has synthetic value, I do not feel that emphasis on controlling diastereoselectivity in the Pd-catalyzed 1,4-difunctionalization of cyclic dienes represents a huge leap forward. Again, the Pd-catalyzed syn 1,4-difunctionalization of cyclic 1,3-dienes was demonstrated by Backvall almost 30 years ago. Therefore, I view it as an incremental (yet valuable) advance that is worthy of publication in a more specialized journal.

Thank you for your insightful comments. We respectfully disagree with this reviewer's assessment. It appears that there may be a misunderstanding regarding the novelty and significance of our work.

Initially, the concern raised was regarding the perceived lack of novelty due to the reported hybrid palladium-catalyzed difunctionalization of conjugated dienes. However, as we have previously addressed, our work fills a notable gap in research concerning the investigation of diastereoselectivity in the difunctionalization of cyclic conjugated dienes. This aspect has been acknowledged and supported by reviewer 1, who noted, *"The argument that this work is just an extension of Glorius and Gevorgyan's earlier studies dismisses the significant advance of achieving highly dependable cis-stereochemistry for cyclic dienes."*

Now, this reviewer's focus has shifted to the demonstration of Pd-catalyzed syn 1,4-difunctionalization of cyclic 1,3-dienes by Backvall almost 30 years ago. We have provided a clear comparison of Backvall's pioneering work and our own in the introduction (see Figure 1c and 1d). Additionally, we have included a figure to further elucidate the differences between the two transformations. It is important to emphasize that these transformations have distinct mechanisms and vastly different substrate scopes.

Considering these factors, we believe that this reviewer's comment may not fully capture the objective assessment of our work. We remain confident in the novelty and significance of our contributions to the field.

Department of Chemistry
2545 McCarthy Mall
Honolulu, Hawai'i, 96822
Zuxiao Zhang, Ph.D.
Assistant Professor
Department of Chemistry

a) Pioneer work by Backvall:

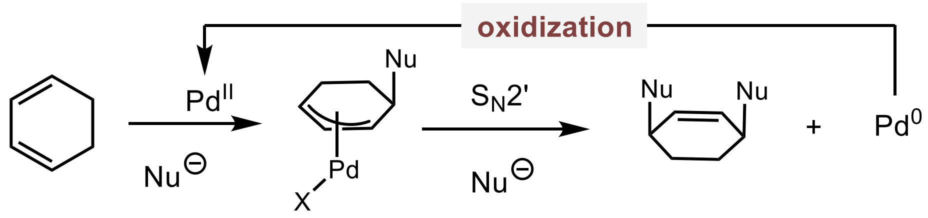

oxidative difunctionalization; Nu = OAc⁻, halide anions

b) Our work

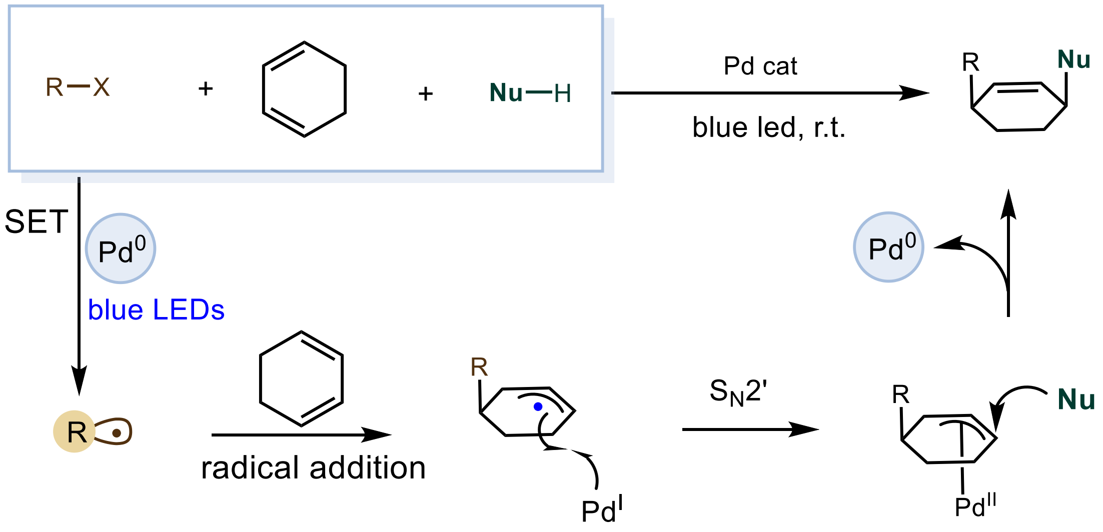

- **redox neutral carboamination of cyclic 1,3-diene**
- **electrophiles: $ArCF_2$-F, Ar-Br, Alkyl-I**
- **nucleophiles: amines, and C, S and O nucleophiles**

Additional Questions:

Quality of experimental data, technical rigor: High

Significance to chemistry researchers in this and related fields: Moderate

Broad interest to other researchers: Moderate

(E) zzhang9@hawaii.edu

Department of Chemistry
2545 McCarthy Mall
Honolulu, HAWAI'I, 96822
Zuxiao Zhang, Ph.D.
Assistant Professor
Department of Chemistry

Novelty: Moderate

Is this research study suitable for media coverage or a First Reactions (a News & Views piece in the journal)?: No

(E) zzhang9@hawaii.edu
